# Stereotaxic atlas of the infant rat brain at postnatal days 7–13

**DOI:** 10.3389/fnana.2022.968320

**Published:** 2022-08-12

**Authors:** Yu-Nong Chen, Xin Zheng, Hai-Lin Chen, Jin-Xian Gao, Xin-Xuan Li, Jun-Fan Xie, Yu-Ping Xie, Karen Spruyt, Yu-Feng Shao, Yi-Ping Hou

**Affiliations:** ^1^Departments of Neuroscience, Anatomy, Histology, and Embryology, Key Laboratory of Preclinical Study for New Drugs of Gansu Province, School of Basic Medical Sciences, Lanzhou University, Lanzhou, China; ^2^Sleep Medicine Center of Gansu Provincial Hospital, Lanzhou, China; ^3^NeuroDiderot – INSERM, Université de Paris, Paris, France; ^4^Key Lab of Neurology of Gansu Province, Lanzhou University, Lanzhou, China

**Keywords:** infant rat, brain, atlas, stereotaxic coordinates, neural development

## Abstract

Recently, researchers have paid progressively more attention to the study of neural development in infant rats. However, due to the lack of complete intracerebral localization information, such as clear nuclear cluster boundaries, identified main brain structures, and reliable stereotaxic coordinates, it is difficult and restricted to apply technical neuroscience to infant rat’s brain. The present study was undertaken to refine the atlas of infant rats. As such, we established a stereotaxic atlas of the infant rat’s brain at postnatal days 7–13. Furthermore, dye calibration surgery was performed in P7–P13 infant rats by injecting Methylene blue, and sections were incubated in Nissl solutions. From the panoramic images of the brain sections, atlases were made. Our article has provided the appearance and measurements of P7–P13 Sprague–Dawley rat pups. Whereas the atlas contains a series of about 530 coronal brain section images from olfactory bulbs to the brainstem, a list of abbreviations of the main brain structures, and reliable stereotaxic coordinates, which were demonstrated by vertical and oblique injections with fluorescent dye DiI. The present findings demonstrated that our study of P7–P13 atlases has reasonable nucleus boundaries and accurate and good repeatability of stereotaxic coordinates, which can make up for the shortage of postnatal rat brain atlas currently in the field.

## Introduction

Neural development is an important and mysterious question for a neuroscientist (Poldrack and Farah, 2015; Di Lullo and Kriegstein, 2017). As an indispensable reference book, the brain stereotaxic atlas is a guide for animal neurosurgery and is a wildly used book (Feng et al., 2022). The nervous system is highly active during the process of its development (Chaudhury et al., 2016). This development is an extremely complicated and precisely regulated process (Ecker et al., 2017; Bagni and Zukin, 2019) that includes the proliferation and differentiation of neural progenitor cells, migration and maturation of neurons, myelination of neuronal axons, and synaptogenesis and organization of the neural circuits (Suzuki, 2007; Jiang and Xu, 2019). Disruption of any of the overlapping steps that contribute to this process can result in a wide range of developmental disorders (Guerrini and Dobyns, 2014). Studying and understanding the timing and role of development in a particular part of the nervous system are important for us to understand and address the impact of early development on lifelong health problems (Thapar et al., 2017).

Rodent dams have large litters that are easy to care, generally disease-resistant, and have no agricultural uses. Rat pups are born after a short gestation (22.5 days) and have long been the general species of choice, such as rat macro- and micro-neuroanatomy and neurophysiology (Clancy et al., 2007). Rats have a number of clear advantages over mice, such as the relatively large size of their brains, which makes brain surgery much easier. Rats are also much easier to handle than mice and less easily stressed by human contact (Ellenbroek and Youn, 2016).

Since rodents are altricial, born in a far less mature condition than humans, it is estimated that the rat’s brain at postnatal day (P) 7 is equivalent to that of the human brain at birth or that rat’s neurodevelopment at P1–10 equates to the third trimester in humans (Dobbing and Sands, 1979; Clancy et al., 2007). At P11–12, the rodents’ cerebral cortex has completed most of its anatomical development (Cirelli and Tononi, 2015). From P7 to P14 in rats equates to human year 1 (Andrews and Fitzgerald, 1997). Rats are therefore good models to examine the development of the nervous system because more immature stages of developmental processes can be studied in postnatal life at a time when they are more experimentally accessible (Cirelli and Tononi, 2015). In particular, P0–P10 is a period during which the rat’s brain shows explosive growth. Meanwhile, P7–P14 represents a pivotal point in the transition from immature to mature that includes the cortex and many physiological functions (Cirelli and Tononi, 2015). Therefore, P7–P14 rat pups are very important experimental subjects for studying neural development (Jouvet-Mounier et al., 1970).

There are various technical applications of neuroscience, for example, neurotropic viruses circuit-tracing, polysomnographic recordings, optrode recording, optogenetic/chemogenetic manipulation, immunohistochemistry, fluorescence *in situ* hybridization, and so on. These techniques have greatly promoted our understanding of the types and functions of nerve cells in the process of brain development and have provided methods and approaches to reveal the pathogenesis of developmental brain diseases. Applying these techniques to P7–P14 rat pups requires a complete atlas of the infant rat brain.

A series of atlases of the developing rat’s brain in stereotaxic coordinates already have been published (see Table 1 for details; Valenstein et al., 1969; Sherwood and Timiras, 1970; Ramachandra and Subramanian, 2011; Calabrese et al., 2013; Khazipov et al., 2015; Paxinos and Ashwell, 2018). These publications include E11–E19, P0–P10, P14, P18, P21, P24, P39, P40, P40, and P80 developing rat’s brain. Among them, even though only P0–P10, P14, P21, and P39 have stereotaxic coordinates, they are not complete. These atlases published above are extremely important as a reference for the development and the advancement of the developing rat brain atlas. However, due to the incomplete atlases of P7–P13, the lack of the stereotaxic coordinate with bregma-lambda reference, the clear nuclear cluster boundary, and the detailed marking of brain structure, it is difficult and limited to apply neuroscientific approaches to an infant rat’s brain. To solve this problem, this study was designed to provide the appearance and measures of P7–P13 Sprague–Dawley rat pups and to establish the stereotaxic atlas of the infant rat’s brain at postnatal days 7–13. The atlas contains a series of about 530 coronal brain section images from olfactory bulbs to the brainstem, a list of abbreviations of the main brain structures, and creative and reliable stereotaxic coordinates, which were demonstrated by vertical and oblique injection. In that case, stereotaxic surgery and neurotropic viruses’ circuit-tracing for an infant rat brain can be realized, and hopefully, our atlas provides some reference value for research on the development of the nervous system.

**TABLE 1:** Atlases of the developing rat brain.

Title	Ages	Plane	Animal	Strength	Weakness	Doi
Stereotaxic atlas of the infant rat hypothalamus	P1, P7, P14	Coronal	Albino rats of the Holtzman strain	Stereotaxic coordinates of hypothalamus	No whole brain image, no data for P7-P13 rats	https://doi.org/10.1002/dev.420020206
A stereotaxic atlas of the developing rat brain	P10, P21, P39	Coronal and sagittal	Female long-evans rats	Stereotaxic coordinates, clear structures and boundaries	No whole brain image, coronal sections lack of olfactory bulbs and cerebellum, no data for P7-P13 rats	https://doi.org/10.1016/0022-510X(72)90178-5
Atlas of the neonatal rat brain	P1, P7, P14	Coronal and sagittal	Sprague–Dawley rat	Clear brain structures and boundaries	Only certain brain structures are labeled, no stereotaxic coordinates, no data for P7-P13 rats	https://doi.org/10.1201/b10500-3
A quantitative magnetic resonance histology atlas of postnatal rat brain development with regional estimates of growth and variability	P0, P2, P4, P8, P12, P18, P24, P40, P80	Multidimensionality	Wistar rats	Establishes the first magnetic resonance histology atlas of the developing rat brain, with an emphasis on quantitation	no stereotaxic coordinates, no clear brain structures and boundaries, no data for P7-P13 rats	https://doi.org/10.1016/j.neuroimage.2013.01.017
Atlas of the postnatal rat brain in stereotaxic coordinates	P0–P10, P14, P21	Coronal	Wistar rats	With an indication of the main brain structures, offering bregma and lambda as the reference points, lots of data for different days	Only certain brain structures are labeled without boundaries, and no data for P7-P13 rats	https://doi.org/10.3389/fnana.2015.00161
Atlas of the developing Rat Nervous System 4th edition	E11–E19	Coronal and sagittal	Wistar rats	Clear nervous system structures and boundaries	No stereotaxic coordinates and no data for postnatal rats	

## Materials and methods

### Animal preparation

Adult male and female Sprague–Dawley rats (6–8 weeks old, weighing = 250 ± 35 g) were purchased from the Experimental Animal Center of Lanzhou University (Lanzhou, China). A male rat with two female rats were housed in a plastic cage (485 mm L × 350 mm W × 225 mm H) for mating and kept in an automatically controlled room in a 12:12-h light/dark cycle (lights on 8:00–20:00 h, illumination intensity ≈ 100 lux) at an ambient temperature (23 ± 1°C) and 50% relative humidity with food and water available *ad libitum*. On the second day of mating, we checked whether the female rats have the vaginal plugs. Once the vaginal plug was confirmed, the pregnant rats were housed individually in cages, and the whole process was recorded with a wireless infrared camera (MS-226ZR-IPH-1/C/M, MS, Dongguan, China). The pregnant rats were raised in litters that were culled to 8 pups within 3 days of birth (day of birth = P0) (Mohns and Blumberg, 2010). The present study used 76 Sprague–Dawley rat pups with both male and female rats. All animals were cared for, and experiments were conducted in accordance with the National Institutes of Health Guide for the Care and Use of Laboratory Animals (2011 revision). The experimental protocol was approved by the Ethics Committee of Lanzhou University (permit number: SCXK Gan 2018–0002, Lanzhou, China). All possible efforts were made to reduce the number of animals used and the discomfort to the animals.

### Stereotaxic surgery

**FIGURE 1:**
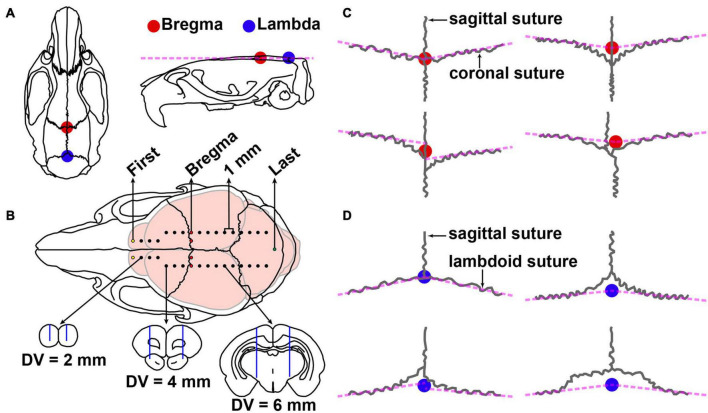
Schematic diagram of stereotactic surgery and variations of bregma and lambda. **(A)** Dorsal and lateral views of the skull of adult Sprague–Dawley rat. The colorful circles represent the position of bregma (red) and lambda (blue). **(B)** Top, injection site of methylene blue in P9 Sprague–Dawley rat pups. The coordinate sites were related to the size of different parts of the brain: the medial and lateral were 0, 1, or 2 mm, the step distance between adjacent anterior and posterior was 1 mm, and the dorsal and ventral (DV) were 2, 4, or 6 mm. The first, bregma, and last sites are shown in yellow, red, and green, respectively. Bottom, the schematic drawing shows the representative brain sections and the needle track (blue line) marked by methylene blue. **(C)** Four variations of the bregma point. **(D)** Four variations of lambda point.

In order to obtain the exact coordinate section, dye calibration surgery was performed as follows. Under isoflurane anesthesia (1–1.5%, flow rate of 0.4 L/min; R510-22, RWD, Shenzhen, China), pups (P7–P13) were weighted and photographed and then placed in a small-animal stereotaxic instrument (Item: 68030, RWD, Shenzhen, China). The method for brain stereotaxic was similar to that of adult rats and mice (Shao et al., 2021). Briefly, disinfection, skin incision, and adequate exposure of the skull surface were performed. The position of the head was adjusted so that the height of the skull surfaces of Bregma (the junction of the coronal suture and the sagittal suture of the skull) and Lambda (the point of intersection of the best fit lines passing through the sagittal suture and the left and right portions of the lambdoid suture) (Figure 1A), and the skull surfaces on 2 mm left and right sides of Bregma in the same plane (Figure 1B). In the present study, we found four variations of bregma (Figure 1C) and lambda (Figure 1D), respectively. The coordinate site of the skull surface of bregma was defined as the zero points (anteroposterior/mediolateral, AP/ML). The skull above the injection sites was thinned with a dental drill and removed carefully (Figure 1B). Injections were conducted with a syringe pump (OEMTJ2A-01, Longer Precision Pump, Hebei, China) connected to a glass micropipette (φ = 10–15 μm) with a volume of 50 nl and a speed of 25 nl/min. Meanwhile, the glass micropipette was retreated slowly. Methylene blue was left in the needle track in this process. The number of injection sites varies depending on the age of the pups. In this way, the first, bregma, and last sites can be distinguished, and the coordinates of each brain slice can be calculated from the data of the injection site.

### Tissue preparation

The pups treated with methylene blue were decapitated and were fixed in 0.1 M sodium phosphate buffer (PBS) containing 4% of paraformaldehyde (PFA) for 7 days at 4°C. The brains were carefully removed and post-fixed in the same fixative for 7 days at 4°C. This allowed the preservation of the brain structures and made it easier to isolate the brains from the delicate skulls (Ramachandra and Subramanian, 2011). After fixation, the brain tissues were immersed in 30% of sucrose solutions in 0.1 M PB at 4°C until the brain was dewatering completely. The 50-μm thick coronal sections were obtained using the cryostat microtome (CM1860, Leica Microsystems, Heidelberg, Germany) and placed in wells with section preservation solution. It is worth noting that when the brain was fixed on the freezing base, this made the bilateral needle track parallel to the base and therefore, adjusted the base angle to make sure that the section was vertical to the line between bregma and lambda.

### Nissl staining

Brain sections were mounted on gelatin-coated microscope slides in sequence, air dried, and incubated in Nissl solutions (0.1% cresyl violet (C5042-10 G, Sigma-Aldrich, Saint Louis, MO, United States) and 1% glacial acetic acid) at 37°C for 30 min. They then were immersed for 5–10 s in each of the following solutions: 65% alcohol, 75% alcohol, 85% alcohol, 95% alcohol, 100% alcohol, 100% alcohol, xylene I, and xylene II. The slides were covered with a cover slip, using Dibutylphthalate Polystyrene Xylene (DPX) mounting media and left to dry for 2 days before image capture.

### Image acquisition and processing

**FIGURE 2:**
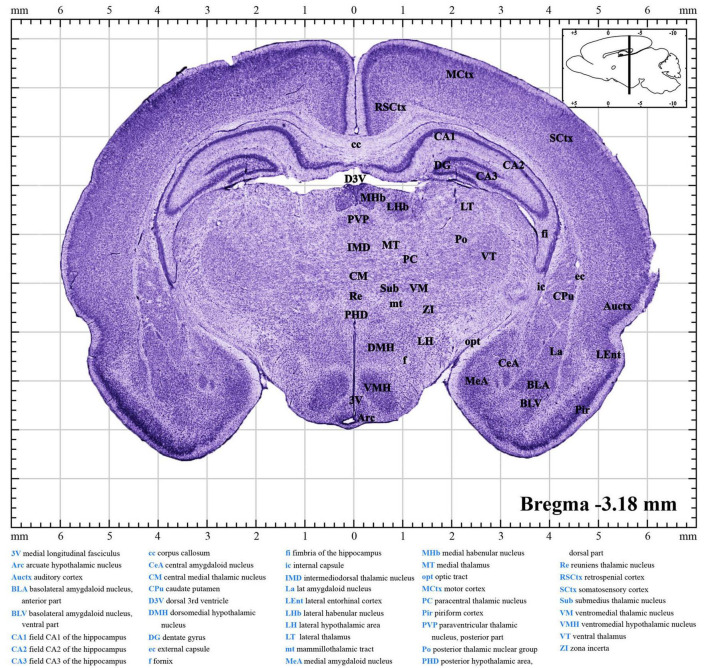
A brief introduction to the atlas. A random figure of the P10 atlas (Supplementary material 4) shows the anteroposterior (AP) of –3.18 mm section. The midline of the brain is defined as mediolateral (ML) of 0.00 mm, and the dorsal surface is defined as dorsoventral (DV) of 0.00 mm. The upper right inset illustrates the sagittal position (black line) of this section. Explanation of abbreviations of the main structures is at the bottom of the figure.

**FIGURE 3:**
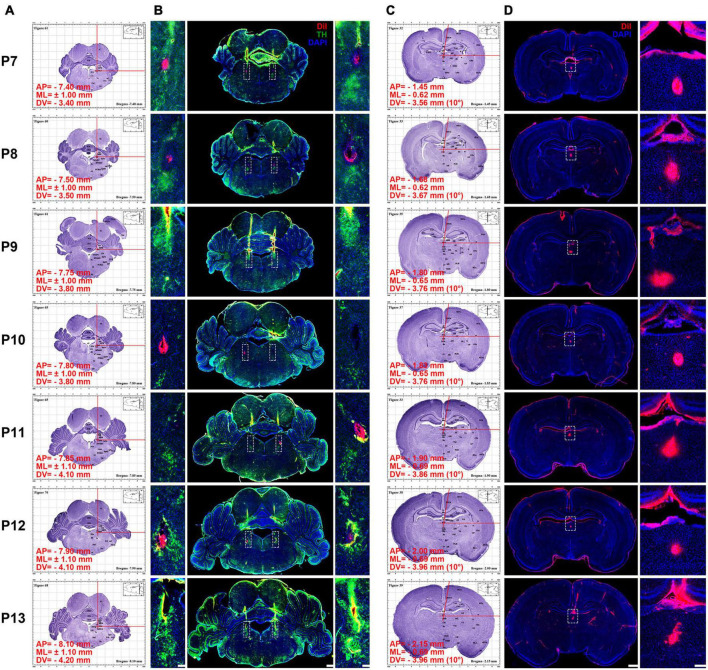
Using the atlas to calculate and inject DiI into P7–P13 pups’ locus coeruleus LC) and paraventricular thalamic nucleus (PVT). **(A)** Using the atlas to calculate the coordinates of the LC (see Supplementary materials 1–7 for clear figures). **(B)** Middle column, coronal section of pups’ brain at the LC stained with DiI (red), tyrosine hydroxylase (TH) (green), and 4′,6-diamidino-2-phenylindole (DAPI) (blue). Left and right column, magnifying insets in dotted square in the middle column. Bar = 500 μm (middle column), bar = 100 μm (left and right column). **(C)** Using the atlas to calculate the coordinates of the PVT (see Supplementary materials 1–7 for clear figures). In order to avoid the damage of the superior sagittal sinus during the surgery, an angle of 10° to the right is used in the calculation (see Supplementary materials 1–7 for clear figures). **(D)** Left column, coronal section of pups’ brain at the PVT stained with DiI (red) and DAPI (blue). Right, the enlarged images of the white dotted box in the left column, bar = 1,000 μm (left column), bar = 100 μm (right column). The red words in **(A,C)** show the coordinates of the DiI injection site.

Panoramic images of the brain sections were captured with the ScanScope Virtual Slides (AperioCS2, Leica, Heidelberg, Germany) and upright brightfield and fluorescence cytometers (Tissue FAXS PLUS, Tissue Gnostics, Vienna, Austria). Images were further processed using Adobe Illustrator CC 2018 software. Established the stereotaxic coordinates that contained the reference of bregma, labeled the primary structures and boundaries, and converted them to 300 dpi images in PDF (Figures 2, 3 and Supplementary materials 1–7; Paxinos and Watson, 2007; Ramachandra and Subramanian, 2011).

### Dye microinjection and immunofluorescence staining

To better illustrate the use of this atlas, the fluorescent dye was exemplarily applied for microinjection into bilateral locus coeruleus (LC) and mesal paraventricular thalamic nucleus (PVT). The steps are as follows: (1) the coordinates of the brain sections corresponding to the P7–P13 were calculated. In our atlases, we can quickly find or caculate the anteroposterior (AP), mediolateral (ML) and dorsoventral (DV) coordinates of LC. In order to avoid the superior sagittal sinus, the ML and DV of PVT can be calculated by tilting 10° to the right or left along the midline according to the Pythagorean Theorem (Figure 3 and Supplementary materials 1–7). (2) Under isoflurane anesthesia (1–1.5%, flow rate of 0.4 L/min; R510-22, RWD, Shenzhen, China), pups were injected with 0.5 μmol/L 50 nl DiI (Item: 42364, Sigma-Aldrich, Saint Louis, MO, United States) into LC and PVT with a speed at 25 nl/min according to coordinates. (3) The pups were anesthetized with pentobarbital sodium (100 mg/kg) and perfused *via* the ascending aorta with 30 ml of saline containing heparin (1 U/ml) and followed by 4% of PFA in 0.1 M PB. Brains were removed, post-fixed in the same fixative for 7 days, and immersed in 30% sucrose solution in 0.1 M PB at 4°C until the samples dewatering completely. The 50-μm thick coronal slices were obtained using the cryostat microtome (CM1860, Leica Microsystems, Heidelberg, Germany) and stored at −20°C in a section preservation solution.

Brain sections that contained LC were rinsed in 0.01 M of PBS and incubated in blocking solution (10% bovine serum in PBS) for 1 h. Sections were incubated with a rabbit polyclonal to Anti-Tyrosine Hydroxylase (TH) Antibody (1:1,000, ab6211, Abcam, Cambridge, England), diluted in PBS containing 1% bovine serum for 24 h at 4°C on an agitator. After rinsing in PBS, sections were incubated with an Alexa Fluor 488-conjugated D-R IgG (1:2,000, 711-545-152, Jackson ImmunoResearch Laboratories, Inc., PA, United States) and 4’,6-diamidino-2-phenylindole (DAPI; 1:8,000, D8417, Sigma-Aldrich, Saint Louis, MO, United States) for 4 h at room temperature. Selected the brain sections where the PVT of P7–P13 is located. The floating sections were rinsed in 0.01 M PBS (pH 7.4) and incubated in DAPI for 4 h at room temperature. Finally, sections were mounted on slides, covered with a coverslip, using 90% glycerol in 0.1 M PB, and observed under a fluorescence microscope (BX53, Olympus, Tokyo, Japan), and images were captured with upright brightfield and fluorescence cytometers (Tissue FAXS PLUS, Tissue Gnostics, Vienna, Austria).

### Data analysis

The physical appearance of each animal was observed and recorded before anesthesia. The increase in body size and a change in the body color of representative pups with aging were recorded by photographing. In addition, their weight was measured and recorded. During the stereotaxic surgery, the distance between bregma and lambda was measured by a stereotaxic instrument under a stereo microscope (SZ61, OLYMPUS, Tokyo, Japan) after the skull surface was leveled. All data were expressed as means ± SEM.

For identifying brain structures, sections were systematically compared with images from the existing developing rat brain atlases and adult rat brain atlas, and the abbreviations of the main structures were labeled and explained (Paxinos and Watson, 2007; Ramachandra and Subramanian, 2011; Khazipov et al., 2015). About stereotaxic coordinates, for the AP, the AP distance from the bregma point, we set the bregma point at zero; for the ML, the left and the right distance from midline, we set the midline at zero; and for the DV, the depth from the brain surface, we set the brain surface as zero planes. The stereotactic coordinates were calculated as follows: bregma was defined as the zero point (AP/ML = 0 mm). Rostral was positive, while caudal was negative. Each needle track corresponded to an integer, and the distance between the two adjacent needle tracks was 1 mm. The coordinates of the brain slices between the needle tracks were converted by multiplying the ratio of the total number of brain slices between the two adjacent needle tracks by 1 mm. Actually, due to the differences generated during the slicing process, the total number of slices per 1 mm is between 18 and 22, with an error of ≤10%. The results have been annotated in our brain atlas.

## Results

### Appearance and measurements of P7–P13 pups

**FIGURE 4:**
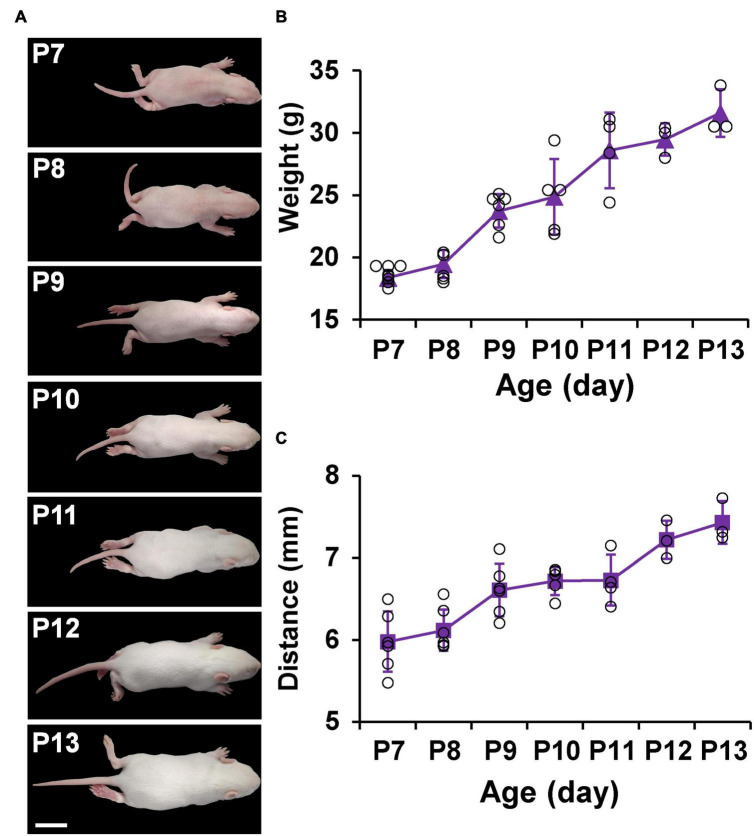
Appearance and measurements of P7–P13 pups. **(A)** The dorsal view of the representative pups shows an increase in body size and a change in body color from pink to white. **(B)** Average body weight change in pups. **(C)** Average distance between bregma and lambda change in pups. Values are means ± SEM (*n* = 3–7). Bar = 2 cm.

Figure 4 shows the appearance and measurements of P7–P13 pups. P7 pups are pink in body color and grow a very thin layer of golden fur, and their eyes are closed. As they age, the pups begin to grow more white fur and increase in body size. Until P13, the pups are covered with white fur and some of their eyes are open (Figure 4A). The physical characteristics of P7–P13 pups are described in Table 2. With the increase of age, the weight and the distance between bregma and lambda of the pups also gradually increased (Figures 4B,C).

**TABLE 2:** The physical characteristics of P7–P13 Sprague–Dawley rat pups.

Age	Physical characteristics
P7 (*n* = 6)	The color of the body from blood red to flesh pink; in addition to the limbs, abdomen and tail, the whole body grows a very thin layer of fur with golden floc
P8 (*n* = 7)	Fur in belly
P9 (*n* = 6)	Fur thickens; a coat of short silvery white fur grows all over the body; females show nipples
P10 (*n* = 5)	Fur grew completely; lower teeth appear; pups became more active
P11 (*n* = 4)	White fur; small lower teeth; high mobility
P12 (*n* = 3)	Cleft appears in the center of the eye bubble; small lower teeth
P13 (*n* = 3)	Eyes have not yet fully opened; stools granular; lower teeth obvious

### Atlas with a coordinate system

Brain sectional images were loaded into the coordinate system as shown in Figure 2 and Supplementary materials 1–7. The collection of atlases prepared from postnatal days P7–P13 rats is deposited in Supplementary materials 1–7. Each file contains a series of about 68–82 coronal brain section images from olfactory bulbs to the brainstem and a list of abbreviations of the main brain structures. Every image was separated by 200–500 μm. Data for the daily atlas came from one infant rat brain whose coordinates were within the average range (Figure 4). In each image (Figure 2), the middle shows the coronal drawing that can be used to identify the brain structures by Nissl bodies. The major parts of the brain were labeled *via* Adobe illustrator in the drawing and full names are available at the bottom. The large number at the bottom right shows the AP distance of the coronal plate from the bregma, and the image in the upper right corner shows the pattern drawing of the sagittal plane 0.5 mm from the middle suture. When readers use our atlas, according to the required nucleus, they may find and calculate the corresponding AP, ML, and DV coordinates.

The comparison of all atlases showed that the brain of P7 was smaller, the cerebral cortex was thinner, and the boundary of the nucleus was not obvious. With the increase of age, the brain size of the pups was gradually increasing, the cerebral cortex gradually thickened, and the nucleus boundary became gradually more clear.

### Immunohistochemistry

Figure 3B shows that TH is labeled in bilateral LC of P7–P13 pups and DiI is also labeled in bilateral LC. Figure 3D shows that DiI is labeled in mesal PVT of P7–P13 pups. When injecting LC, the vertical arm of the brain stereotaxic instrument remained vertical, while injecting PVT, the vertical arm of the instrument should be deflected 10° outward along the midline. The results show that the location of LC and PVT can be accurately marked by using the coordinates of the atlas, and the reliability of stereotaxic coordinates of our brain atlas was demonstrated by vertical and oblique injections. At every age, we had 6 pups to be injected, and the success rate of correct coordinate injection respectively was 85.7% in LC and 95.2% in PVT.

## Discussion

The present study demonstrated that the appearance of the P7–P13 pups is consistent with what Sulagna Dutta and Pallav Sengupta had described (Dutta and Sengupta, 2016). More specifically, the body color of P7–P13 pups changed from pink to being covered with white fur, their closed were eyes opened, their weight was gradually increased, and the distance between bregma and lambda was also gradually increasing as they aged (Table 2 and Figure 4). More importantly, our study established the stereotaxic atlas of the infant rat brain at postnatal days 7–13 (Supplementary materials 1–7). The continuous day measures are conducive to observe the subtle changes while developing. From the atlas, we can intuitively observe that the brain size of the pups is gradually increasing, the cerebral cortex is gradually thickening, and the nucleus boundary is gradually clearer while growing (Figure 3 and Supplementary materials 1–7). Noteworthy, main brain structures that were not stipulated in other atlases (Ramachandra and Subramanian, 2011; Khazipov et al., 2015) are marked in our atlas (Figure 2 and Supplementary materials 1–7), and the reliability of creative stereotaxic coordinates (the coordinates are calculated separately every 1 mm, and the error is smaller) of our brain atlas is demonstrated by vertical and oblique injection (Figure 3). In the present study, we chose PVT and LC because they play a critical role in multiple biological functions (Uematsu et al., 2015; Liu et al., 2017; Ren et al., 2018; Liang et al., 2020). More importantly, the anatomical structure of PVT and LC is symmetrically distributed and located in a constant position, which is easy to identify and locate (Nieuwenhuys et al., 2008; Fernandes et al., 2012; Kirouac, 2015; Vertes et al., 2015; Sacchini et al., 2018). Therefore, we chose these two nuclei as verification nuclei.

However, for better use of our atlas, here are three things to be noted: (1) in order to make stereotaxic coordinates of this brain atlas, which can be used in most stereotactic surgery, the key is to make sure the animals are consistent in weight at the same age. We have suggested to use the mean body weight in Figure 4B. Because the pups’ weight changes quickly in the early growth, body and brain sizes can vary significantly depending on pups’ weight. As a consequence, this requires keeping the number of pups in each litter to about eight, so that there is no significant difference in the weight of the pups fed by mothers from different litters. A litter of eight is just enough to ensure that each animal thrives and does not become too small or fat due to nutritional imbalances. It is not recommended to be used in species with large body size differences due to the large deviation of nuclear location between infant rats of different ages and different body weights. (2) It is difficult to measure the distance from the surface of the skull to the nucleus due to the large curvature of the pups’ skull, so we take the surface of the brain as the zero point of the ordinate. However, due to the influence of individual differences and cerebral hemorrhage, we need to clean the bleeding site frequently during the surgery and adjust the brain surface repeatedly to obtain a stable and uniform height. This is why potential errors may occur in LC and PVT injection and, in particular, the LC’s error. The AP and ML coordinates are generally correct, while DV coordinates may show some errors. This is because the brain surface at LC will overflow more cerebrospinal fluid and blood, which needs to be cleaned repeatedly. (3) The reader’s coronal slice may be slightly different from the atlas, which has to do with the angle at which the brain is fixed to the frozen base. This required us to maintain a uniform standard when coronal sections were established.

In summary, our P7–P13 atlases have many advantages, such as the more comprehensive data of P7–P13, complete brain structure, reasonable nucleus boundaries, detailed marking of nuclei, and accurate and good repeatability of stereotaxic coordinates. Our findings can make up for the shortage of postnatal rat brain atlas currently available. Given that our atlas researchers may get more confident in the study of newborn rats and execute stereotaxic surgery and neurotropic viruses circuit-tracing for infant rat brain.

## Data availability statement

The original contributions presented in this study are included in the article/Supplementary material, further inquiries can be directed to the corresponding authors.

## Ethics statement

The animal study was reviewed and approved by the Ethics Committee of Lanzhou University.

## Author contributions

Y-PH, Y-NC, and Y-FS designed the study. Y-NC, XZ, and X-XL conducted the experiments. H-LC, J-XG, J-FX, and Y-PX collected and analyzed the data. Y-PH, Y-NC, Y-FS, and KS wrote the manuscript. All authors approved the final version and evaluated the accuracy and integrity of the work.
