# Supplementary material for: Stereotaxic atlas of the infant rat brain at postnatal days 7–13
Source: Front Neuroanat. 2022 Aug 12;16:968320. doi: 10.3389/fnana.2022.968320 (PMC9412974; doi:10.3389/fnana.2022.968320)
Supplement: Supplementary file 1 [file Data_Sheet_1.PDF]

Supplementary Material 1

Stereotaxic Atlas of the Infant Rat Brain

P7 (# G-14-1, 17.5 g)

Yu-Nong Chen[1], Xin Zheng[1], Hai-Lin Chen[1], Jin-Xian Gao[1], Xin-Xuan Li[1], Jun-Fan Xie[1], Yu-Ping Xie[3], Karen Spruyt[4], Yu-Feng Shao[1,2]* and Yi-Ping Hou[1,2]*

[1]Departments of Neuroscience, Anatomy, Histology, and Embryology, Key Laboratory of Preclinical Study for New Drugs of Gansu Province, School of Basic Medical Sciences, Lanzhou University, Lanzhou, China
[2]Key Lab of Neurology of Gansu Province, Lanzhou University, Lanzhou, China
[3]Sleep Medicine Center of Gansu Provincial Hospital, Lanzhou, China
[4]Université de Paris, NeuroDiderot – INSERM, Paris, France.

* Correspondence: Yu-Feng Shao (shaoyf@lzu.edu.cn); Yi-Ping Hou (houyiping@lzu.edu.cn)

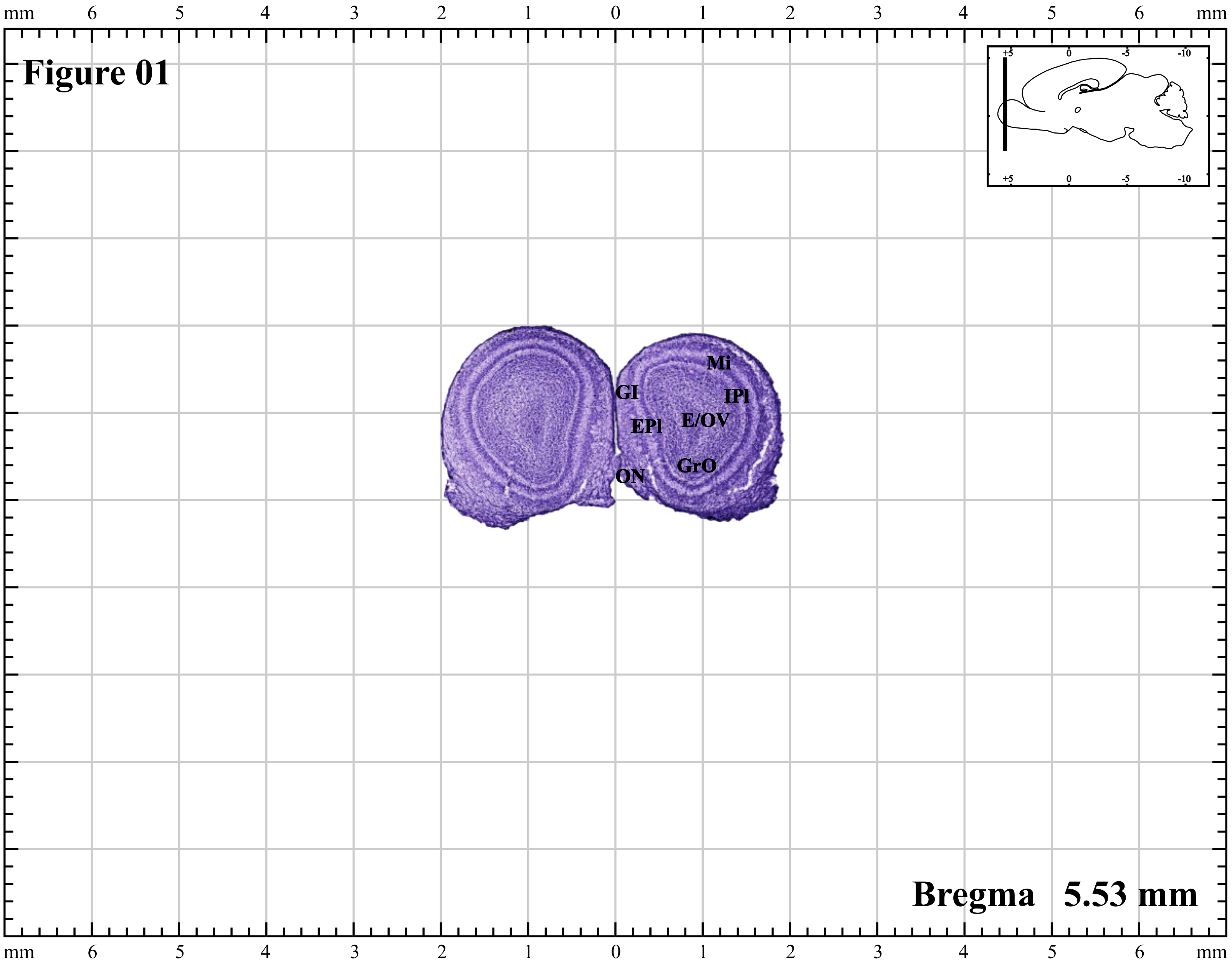

EPl external plexiform layer of the olfactory bulb
E/OV ependymal and subendymal layer/olfactory ventricle
GrO granular cell layer of the olfactory bulb
GI granular insular cortex
IPl internal plexiform layer of the olfactory bulb
Mi mitral cell layer of the olfactory bulb
ON olfactory nerve layer

Figure 02

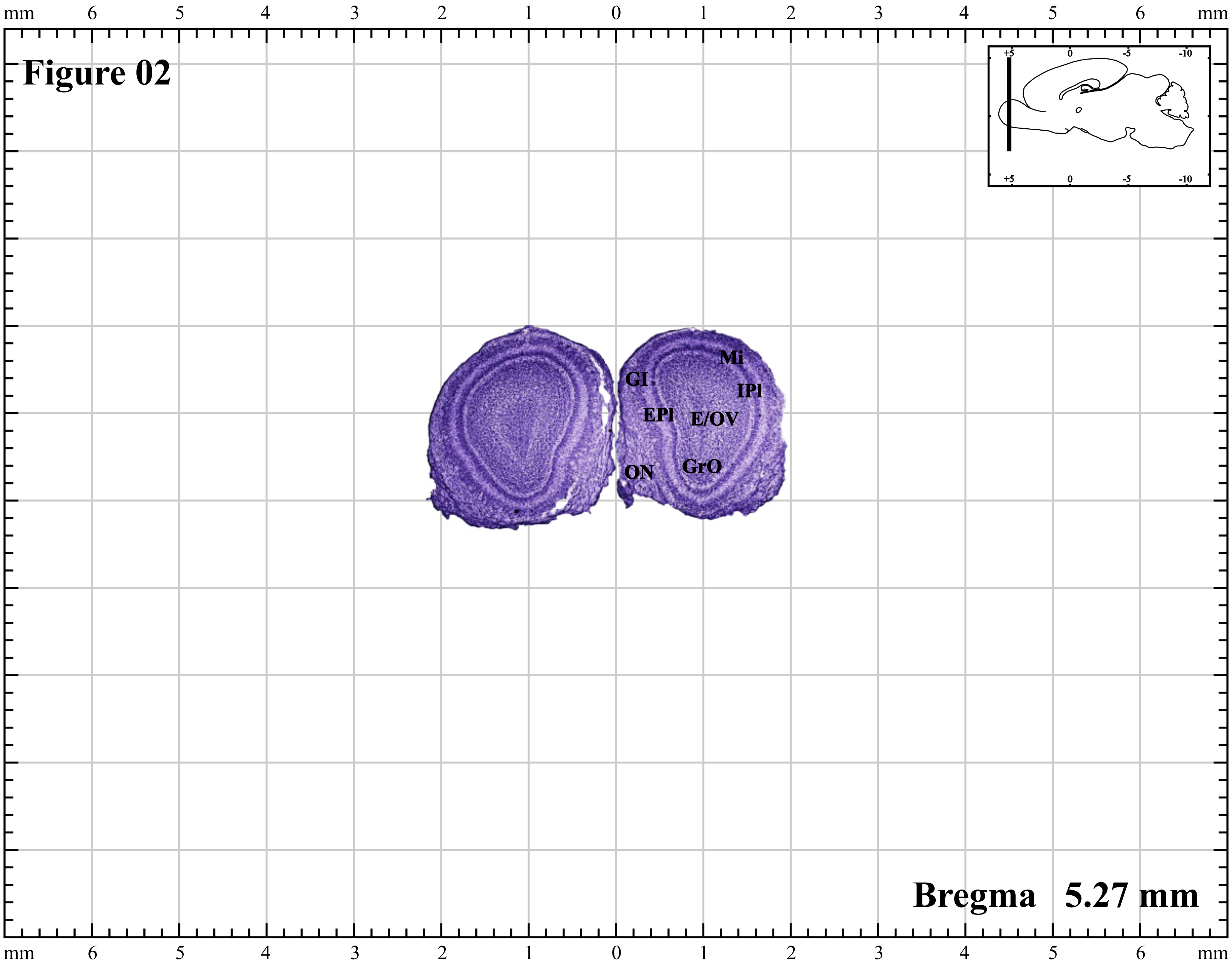

EPl external plexiform layer of the olfactory bulb

E/OV ependymal and subendymal layer/olfactory ventricle

GrO granular cell layer of the olfactory bulb

GI granular insular cortex

IPl internal plexiform layer of the olfactory bulb

Mi mitral cell layer of the olfactory bulb

ON olfactory nerve layer

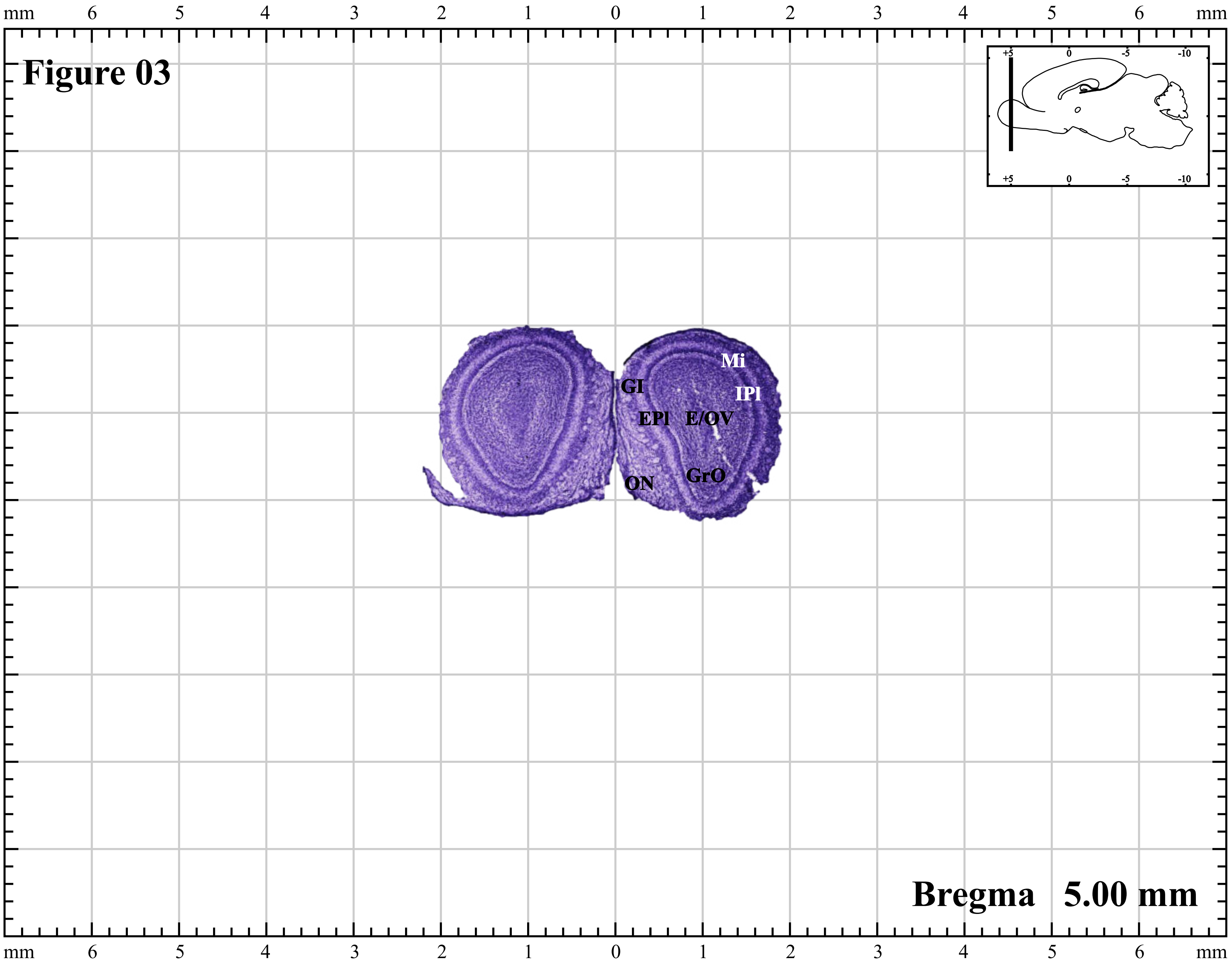

EPl external plexiform layer of the olfactory bulb

E/OV ependymal and subendymal layer/olfactory ventricle

GrO granular cell layer of the olfactory bulb

GI granular insular cortex

IPl internal plexiform layer of the olfactory bulb

Mi mitral cell layer of the olfactory bulb

ON olfactory nerve layer

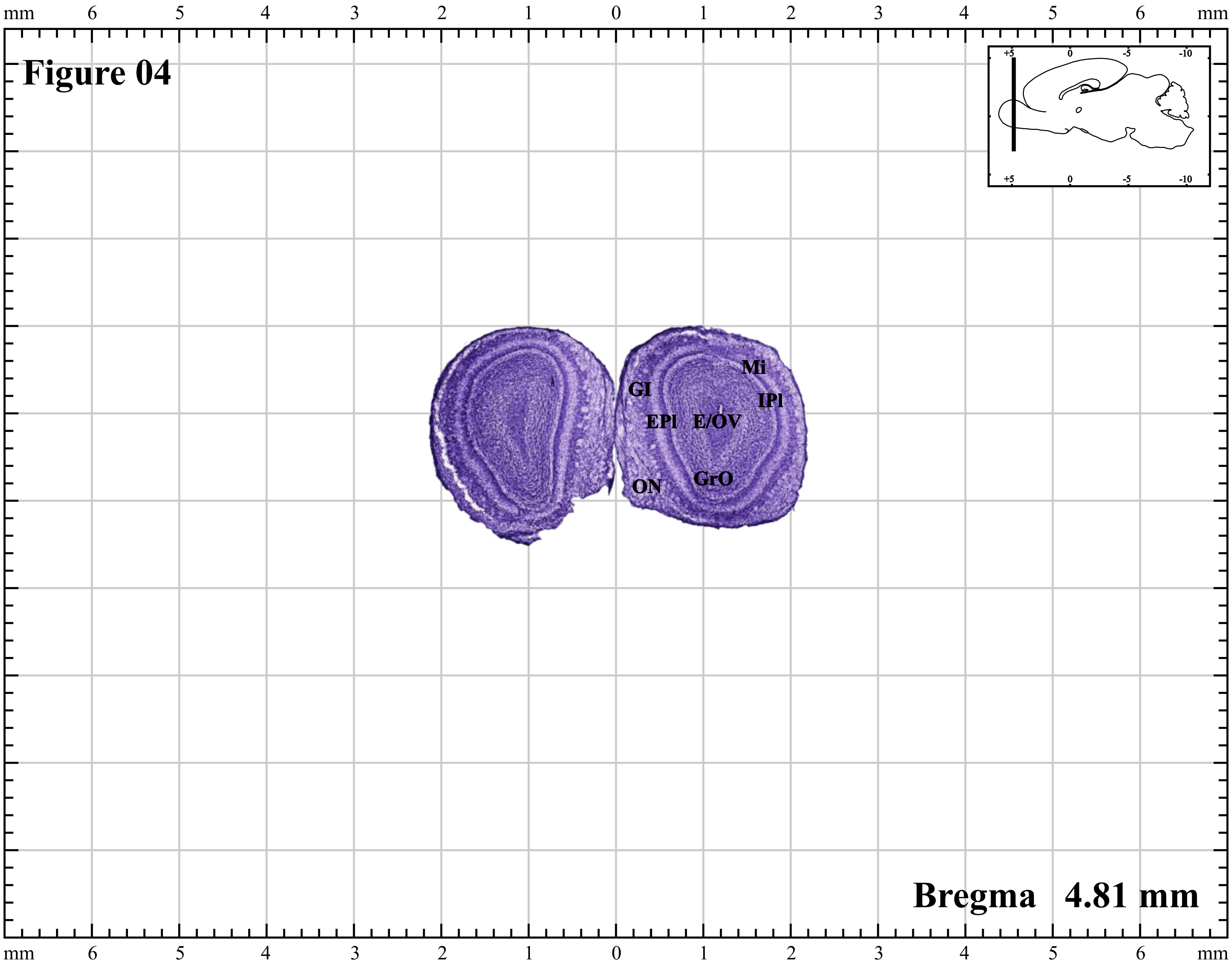

EPl external plexiform layer of the olfactory bulb
E/OV ependymal and subendymal layer/olfactory ventricle
GrO granular cell layer of the olfactory bulb
GI granular insular cortex
IPl internal plexiform layer of the olfactory bulb
Mi mitral cell layer of the olfactory bulb
ON olfactory nerve layer

Figure 05

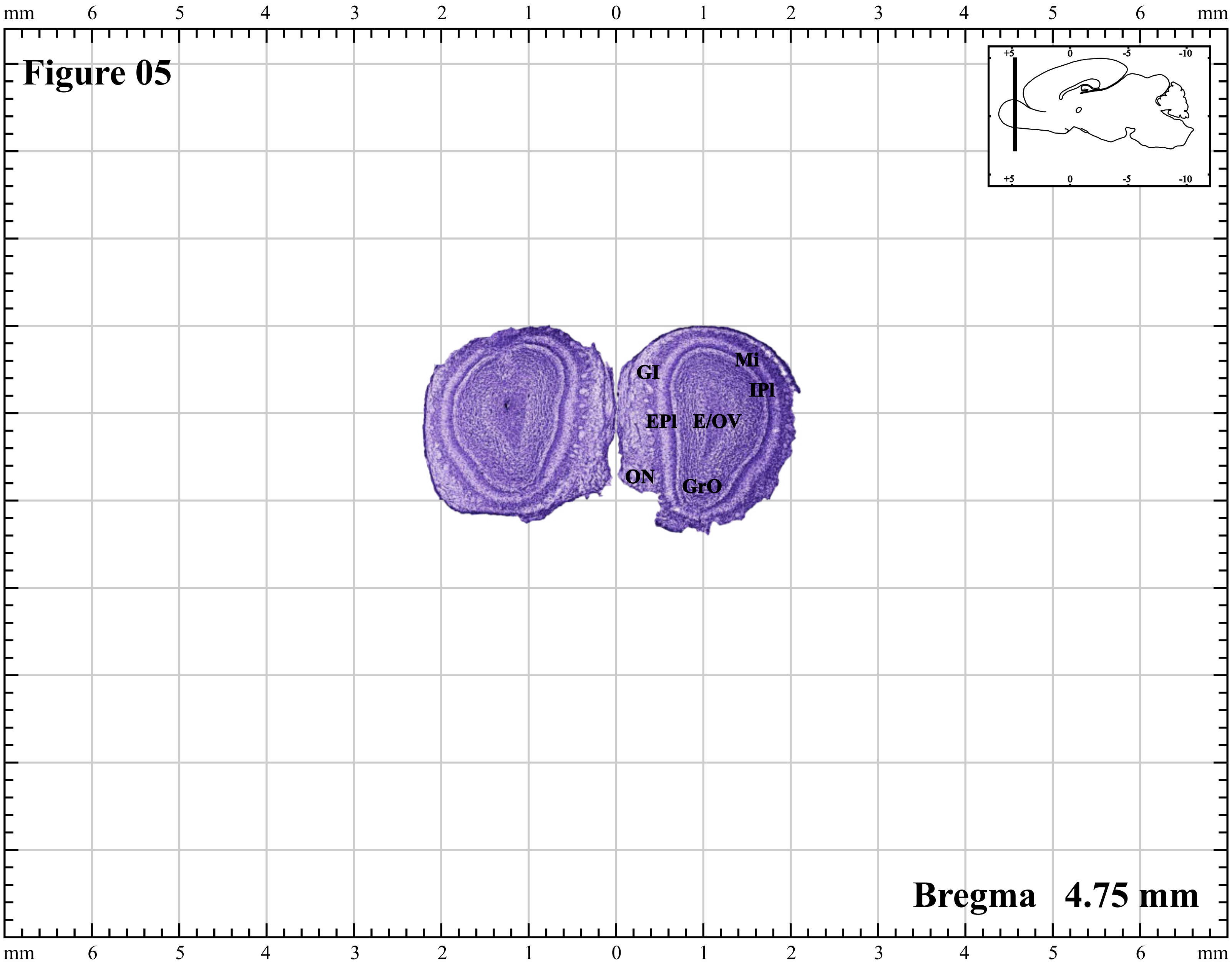

Bregma 4.75 mm

EPl external plexiform layer of the olfactory bulb

E/OV ependymal and subendymal layer/olfactory ventricle

GrO granular cell layer of the olfactory bulb

GI granular insular cortex

IPl internal plexiform layer of the olfactory bulb

Mi mitral cell layer of the olfactory bulb

ON olfactory nerve layer

Figure 06

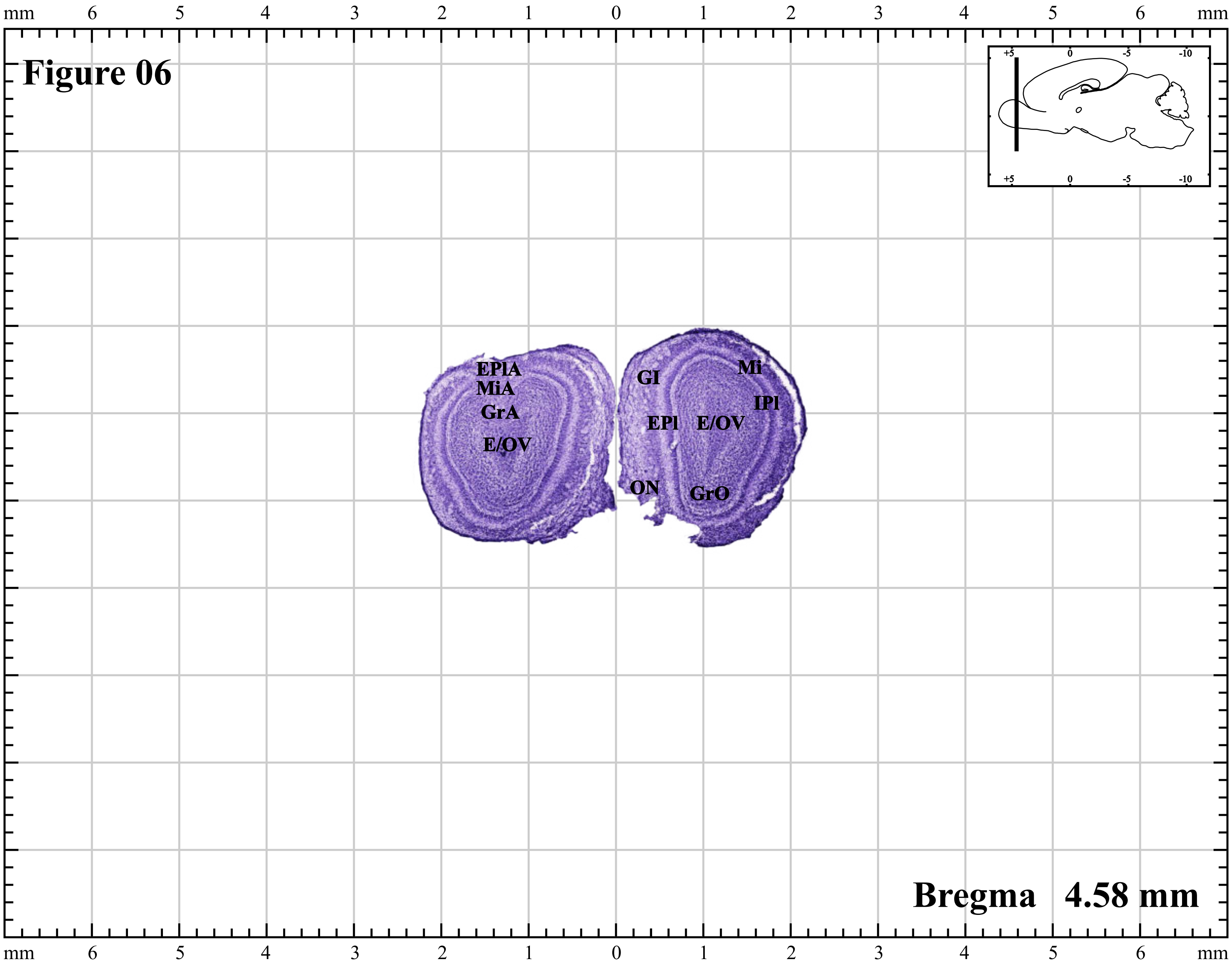

Bregma 4.58 mm

EPl external plexiform layer of the olfactory bulb
E/OV ependymal and subendymal layer/olfactory ventricle
EPlA external plexiform layer of the accessory olfactory bulb
GrO granular cell layer of the olfactory bulb
GI granular insular cortex
GrA granule cell layer of the accessory olfactory bulb
IPl internal plexiform layer of the olfactory bulb
MiA mitral cell layer of the accessory olfactory bulb
Mi mitral cell layer of the olfactory bulb
ON olfactory nerve layer

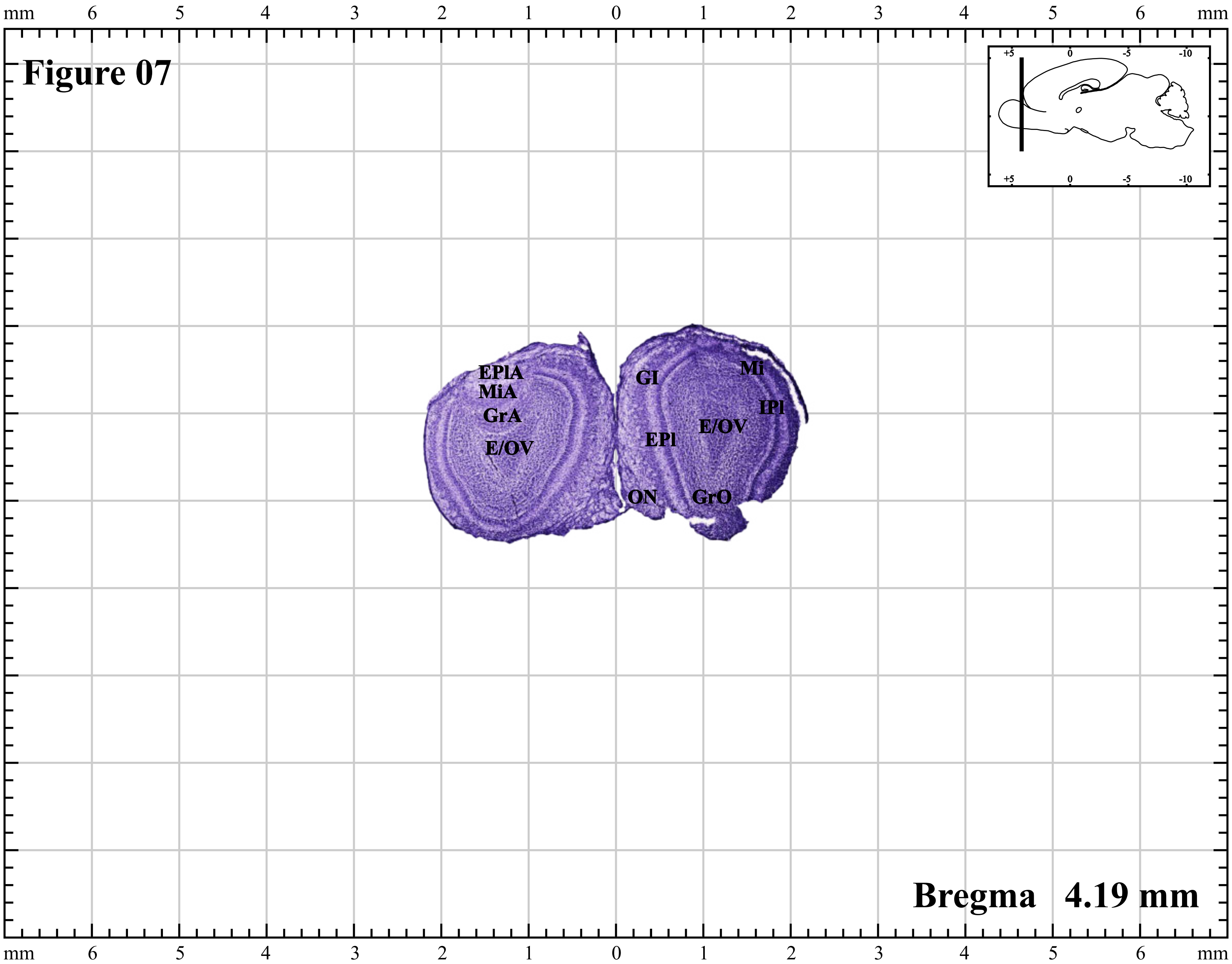

EPl external plexiform layer of the olfactory bulb
E/OV ependymal and subendymal layer/olfactory ventricle
EPlA external plexiform layer of the accessory olfactory bulb
GrO granular cell layer of the olfactory bulb
Gl granular insular cortex
GrA granule cell layer of the accessory olfactory bulb
IPl internal plexiform layer of the olfactory bulb
Mi mitral cell layer of the olfactory bulb
MiA mitral cell layer of the accessory olfactory bulb
ON olfactory nerve layer

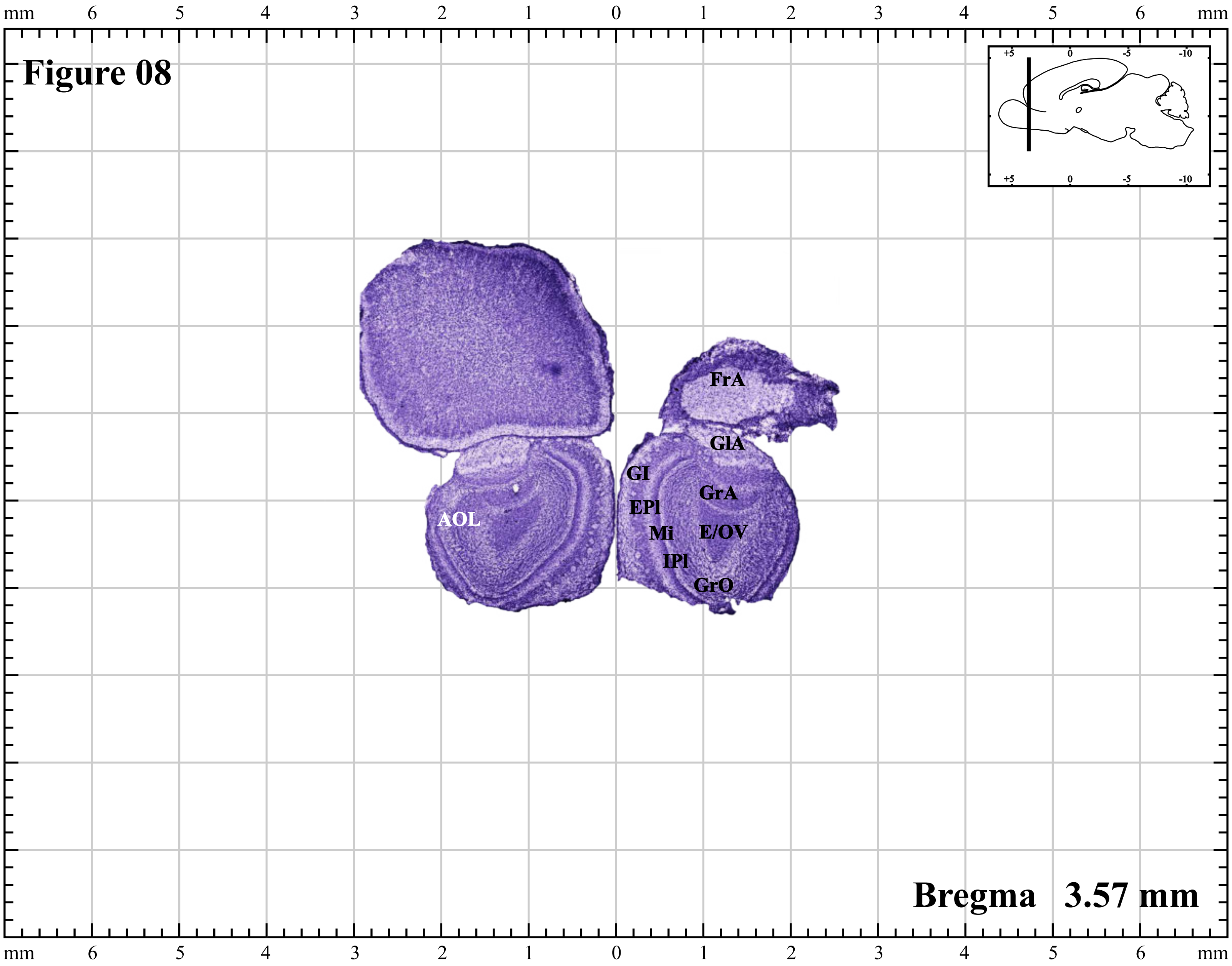

AOL anterior olfactory nucleus, lateral part

EPl external plexiform layer of the olfactory bulb

E/OV ependymal and subendymal layer/olfactory ventricle

FrA frontal assocn cortex

GlA glomerular layer of the accessory olfactory bulb

GrO granular cell layer of the olfactory bulb

GI granular insular cortex

GrA granule cell layer of the accessory olfactory bulb

IPl internal plexiform layer of the olfactory bulb

Mi mitral cell layer of the olfactory bulb

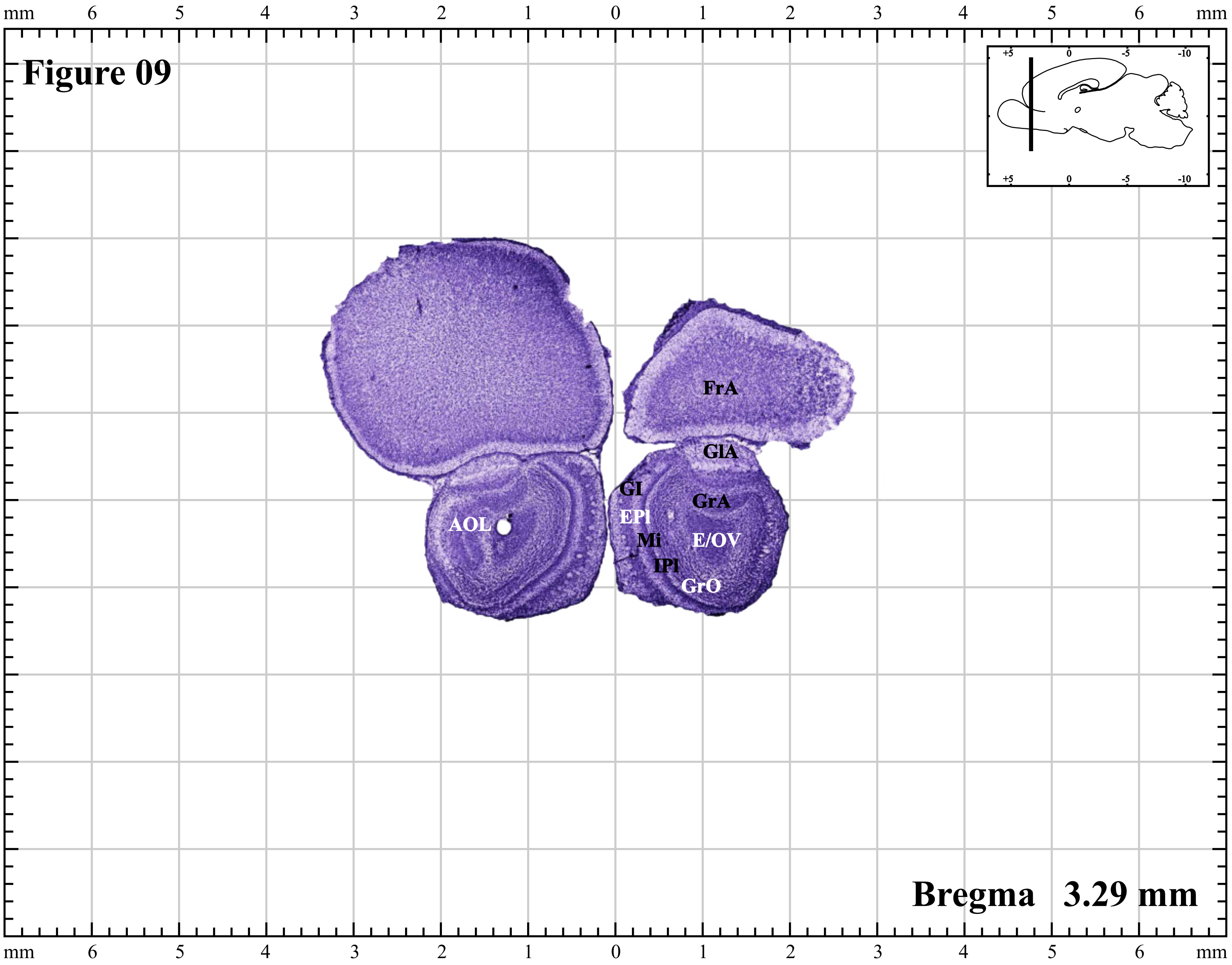

AOL anterior olfactory nucleus, lateral part

EPl external plexiform layer of the olfactory bulb

E/OV ependymal and subendymal layer/olfactory ventricle

FrA frontal assocn cortex

GlA glomerular layer of the accessory olfactory bulb

GrO granular cell layer of the olfactory bulb

GI granular insular cortex

GrA granule cell layer of the accessory olfactory bulb

IPl internal plexiform layer of the olfactory bulb

Mi mitral cell layer of the olfactory bulb

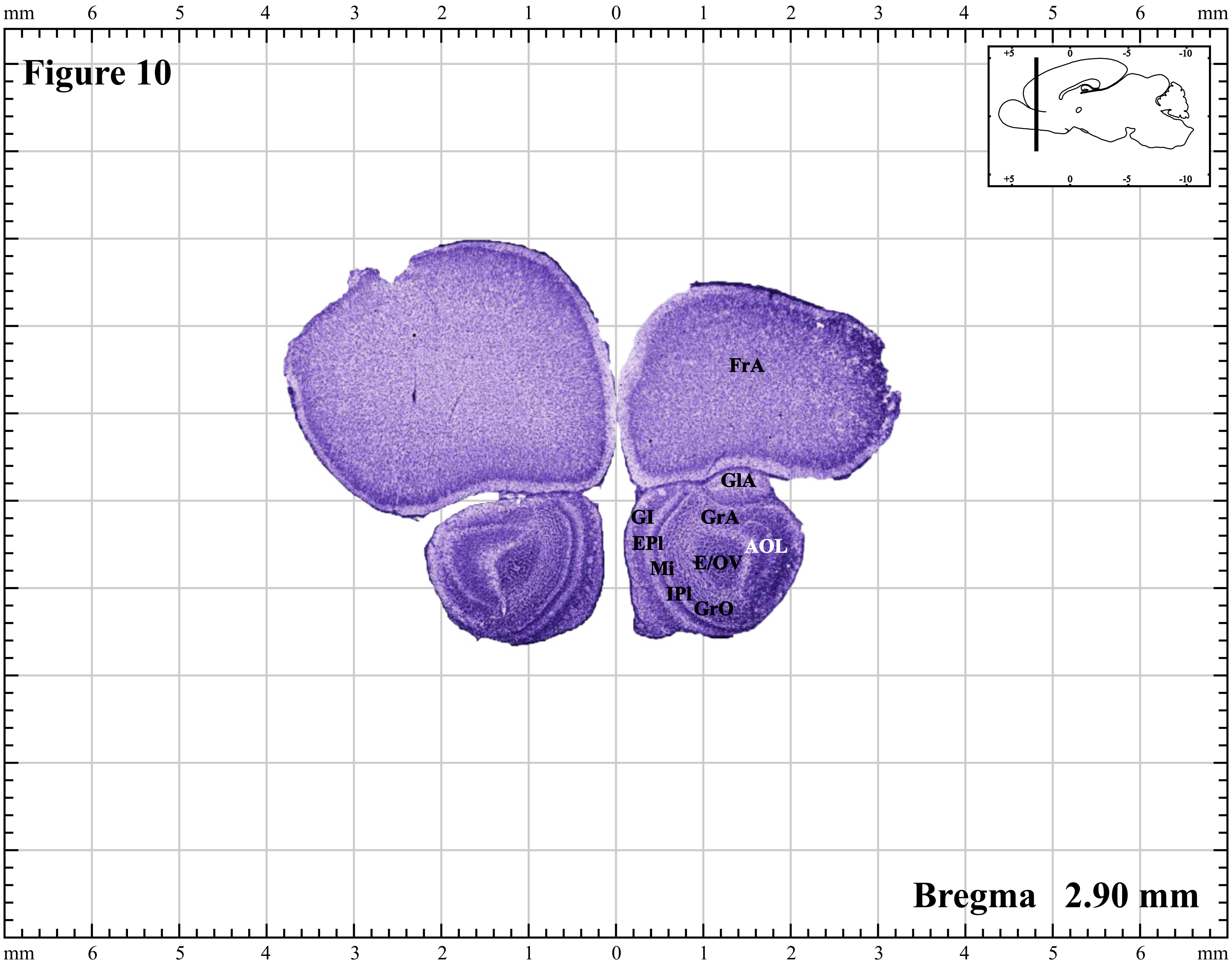

AOL anterior olfactory nucleus, lateral part
EPl external plexiform layer of the olfactory bulb
E/OV ependymal and subendymal layer/olfactory ventricle
FrA frontal assocn cortex
GlA glomerular layer of the accessory olfactory bulb
GrO granular cell layer of the olfactory bulb
GI granular insular cortex
GrA granule cell layer of the accessory olfactory bulb
IPl internal plexiform layer of the olfactory bulb
Mi mitral cell layer of the olfactory bulb

Figure 11

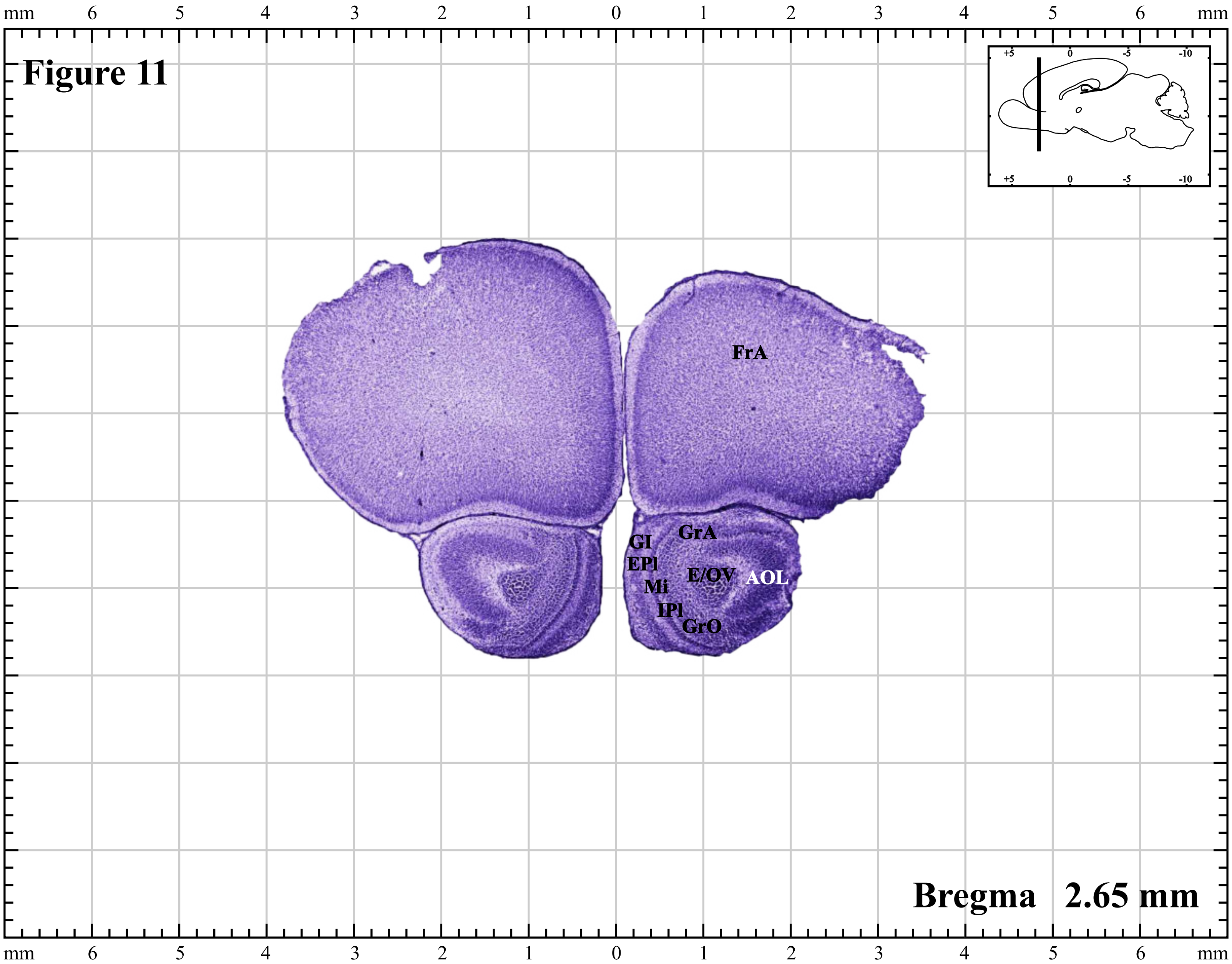

AOL anterior olfactory nucleus, lateral part
EPl external plexiform layer of the olfactory bulb
E/OV ependymal and subendymal layer/olfactory ventricle
FrA frontal assocn cortex
GrO granular cell layer of the olfactory bulb
GI granular insular cortex
GrA granule cell layer of the accessory olfactory bulb
IPl internal plexiform layer of the olfactory bulb
Mi mitral cell layer of the olfactory bulb

Figure 12

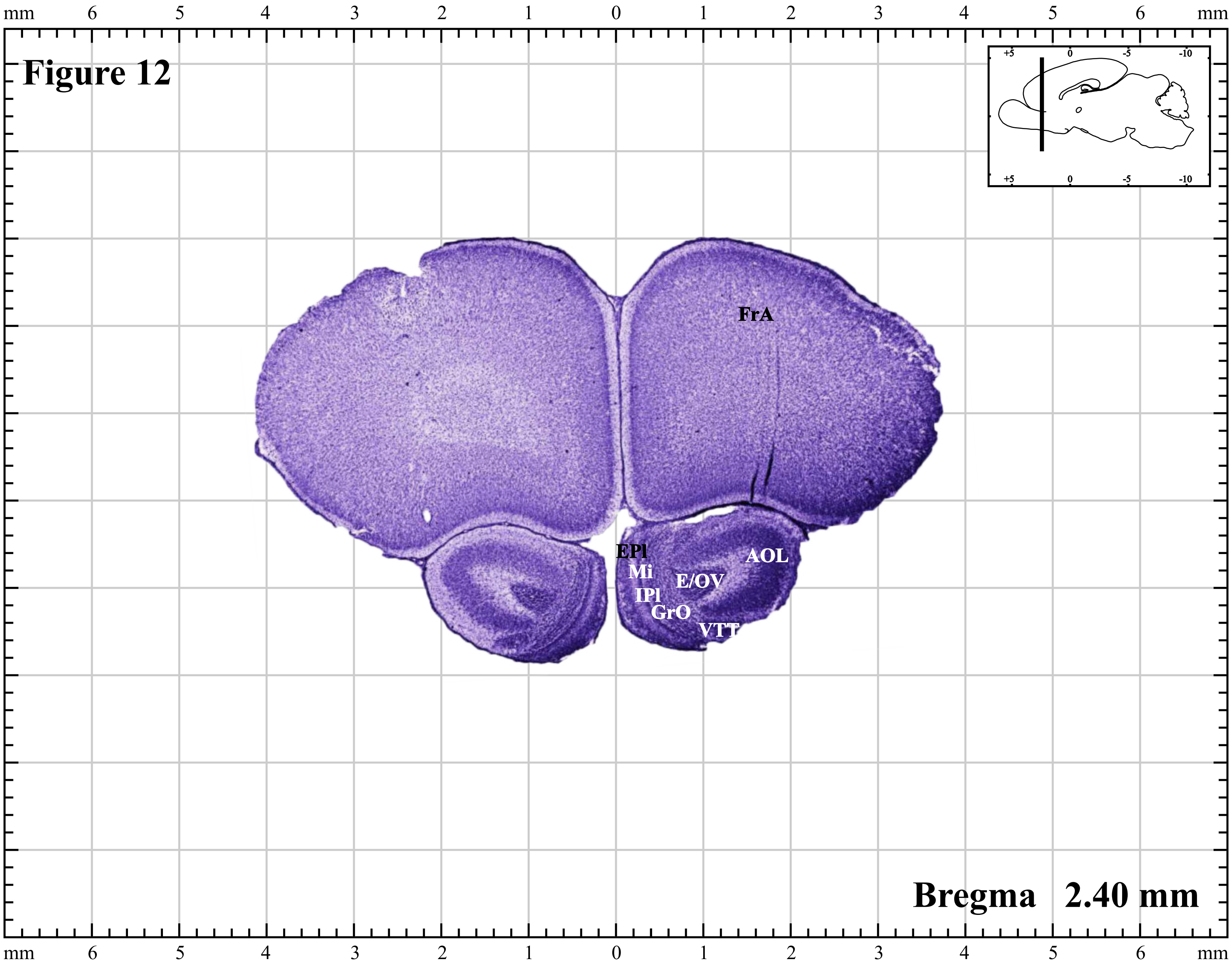

Bregma 2.40 mm

AOL anterior olfactory nucleus, lateral part
EPl external plexiform layer of the olfactory bulb
E/OV ependymal and subendymal layer/olfactory ventricle
FrA frontal assocn cortex
GrO granular cell layer of the olfactory bulb
IPl internal plexiform layer of the olfactory bulb
Mi mitral cell layer of the olfactory bulb
VTT ventral tenia tecta

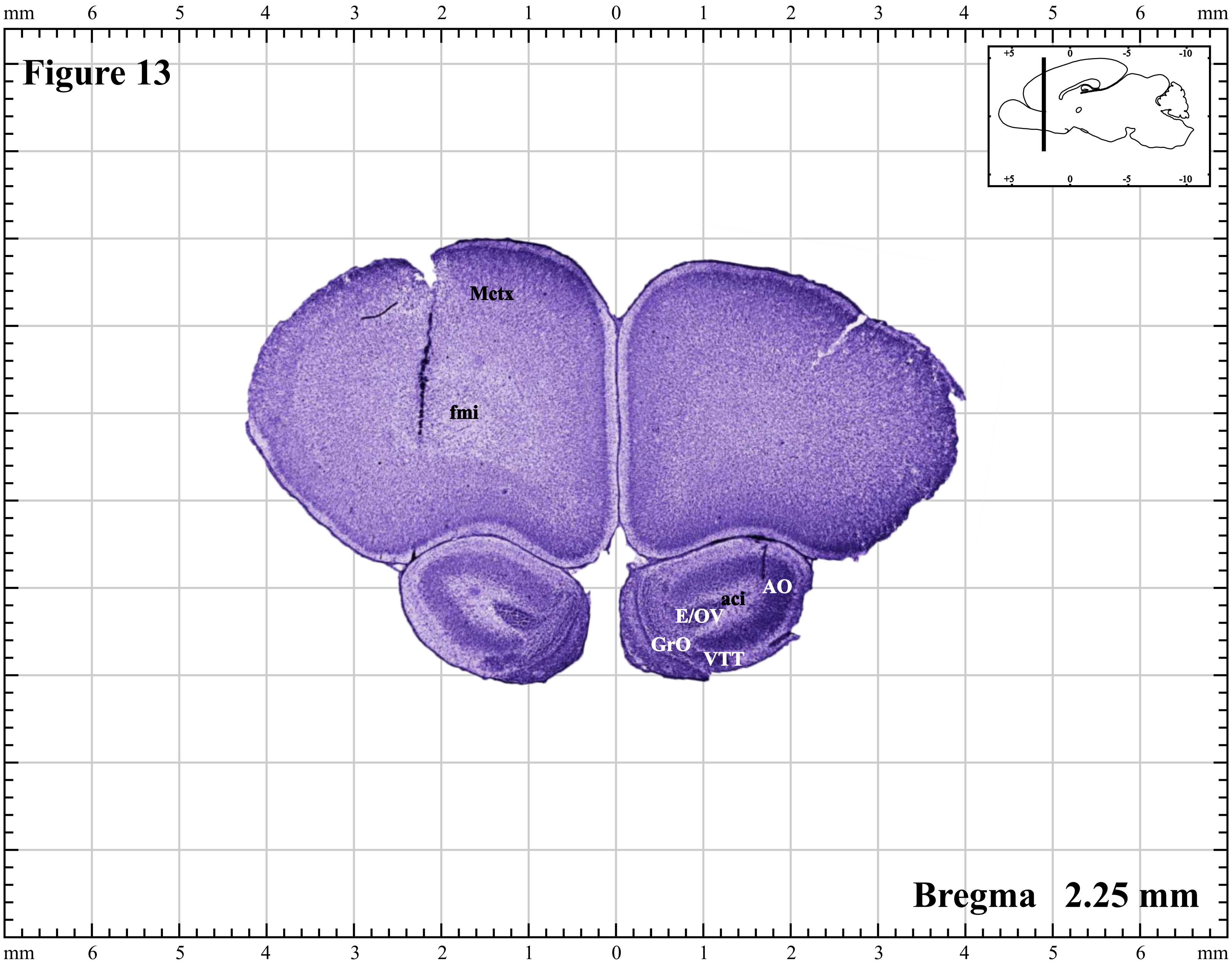

aci anterior commissure, intrabulbar part
AO anterior olfactory nuclues
E/OV ependymal and subendymal layer/ olfactory ventricle
fmi forceps major of corpus callosum
GrO granular cell layer of the olfactory bulb
MCtx motor cortex
VTT ventral tenia tecta

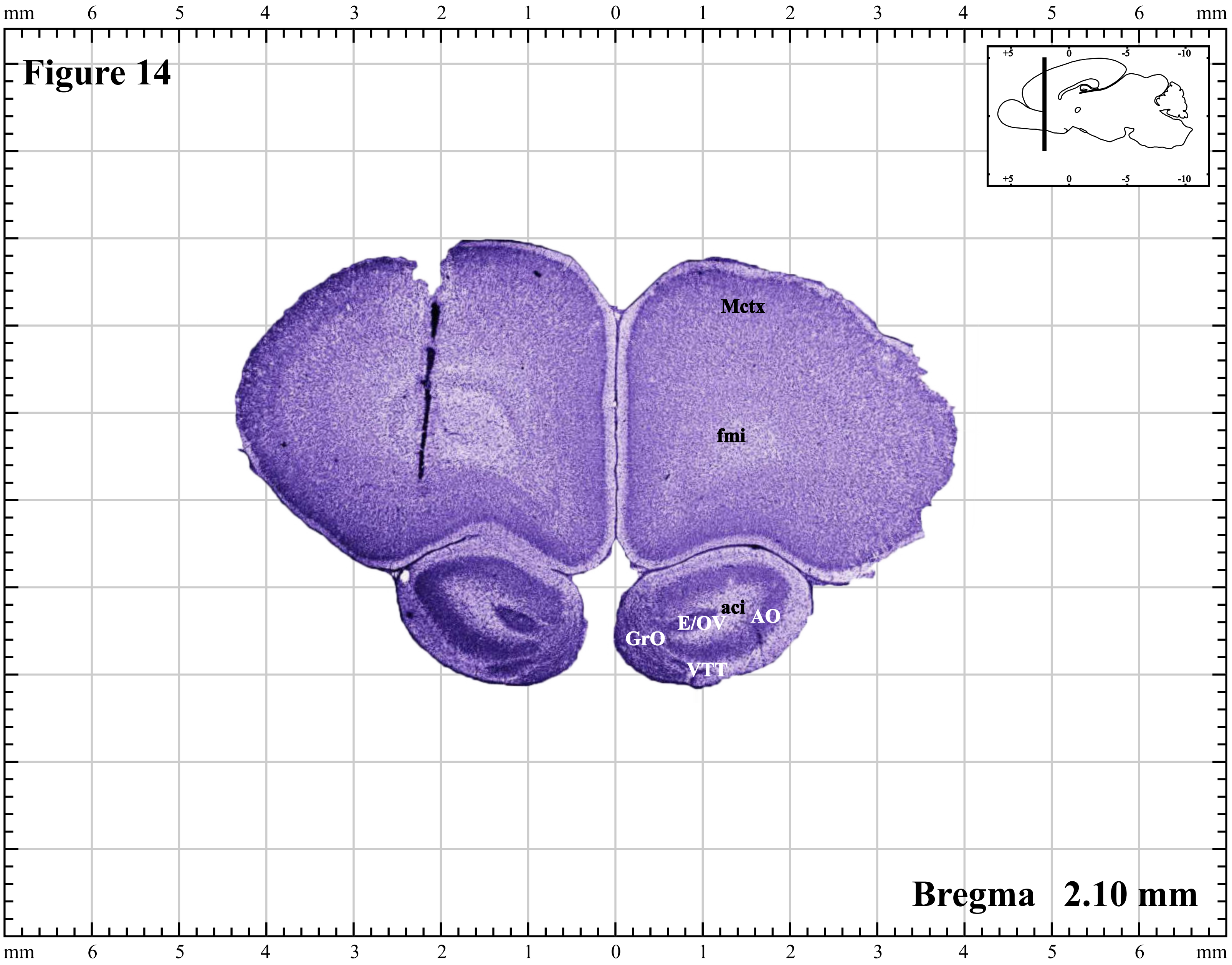

aci anterior commissure, intrabulbar part
AO anterior olfactory nuclues
E/OV ependymal and subendymal layer/ olfactory ventricle
fmi forceps major of corpus callosum
GrO granular cell layer of the olfactory bulb
MCtx motor cortex
VTT ventral tenia tecta

Figure 15

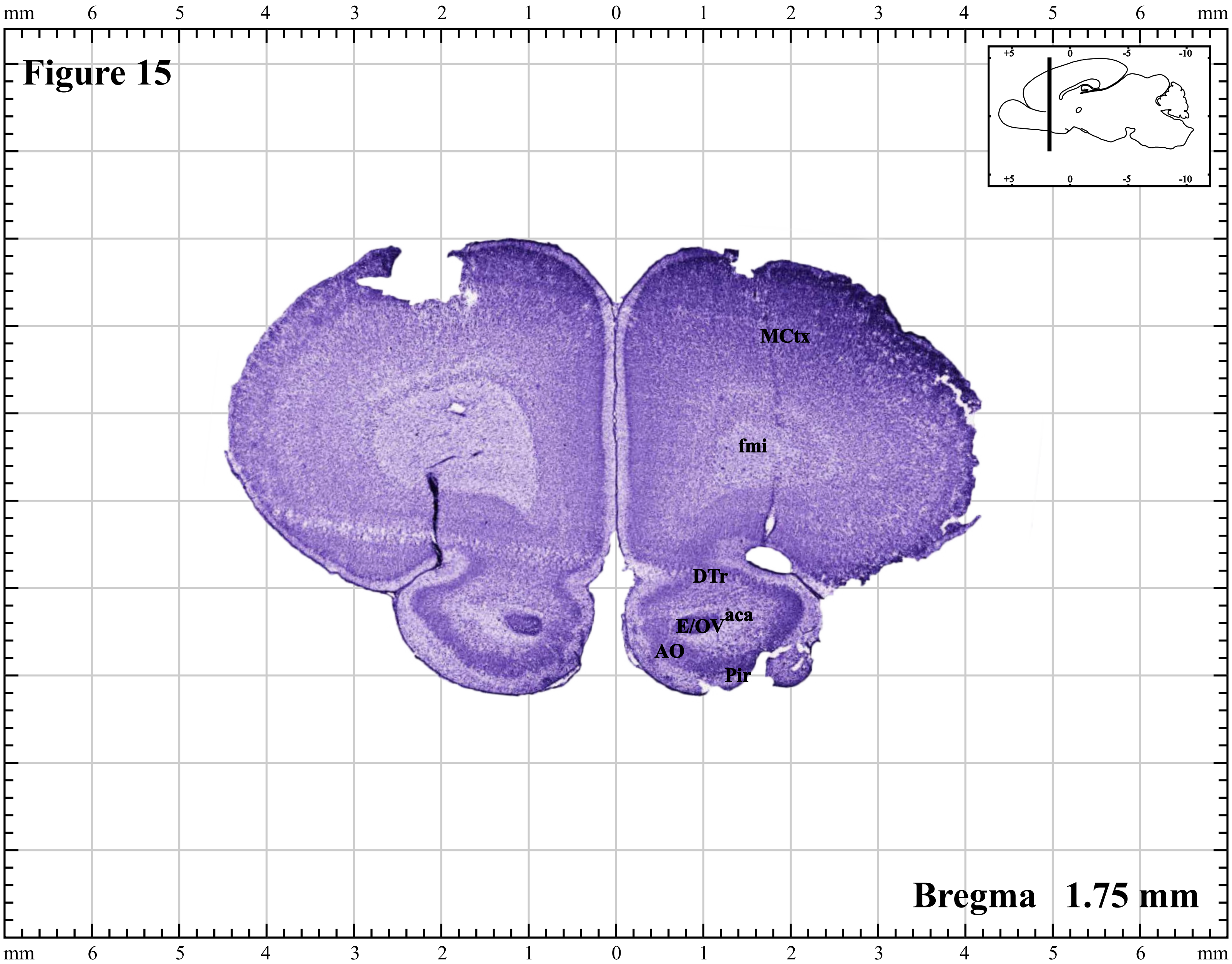

Bregma 1.75 mm

aca anterior commissure, anterior part
AO anterior olfactory nuclues
DTr dorsal transition zone
E/OV ependyma and subependymal layer
/olfactory ventricle
fmi forceps major of corpus callosum
MCtx motor cortex
Pir piriform cortex

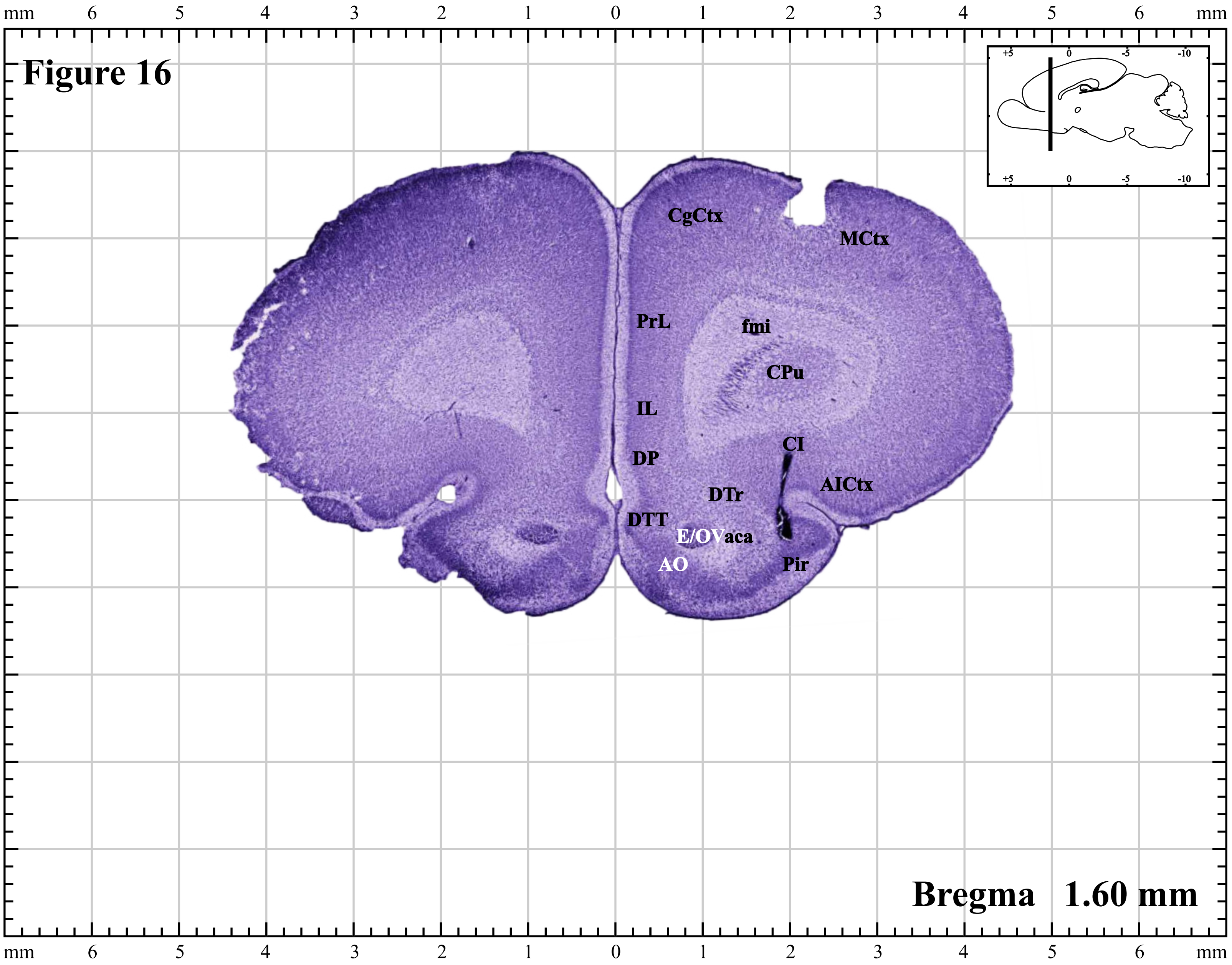

aca anterior commissure, anterior part
AO anterior olfactory nuclues
AICtx agranular insular cortex
CI claustrum
CgCtx cingulate cortex
CPu caudate putamen (striatum)
DP dorsal peduncular cortex
DTT dorsal tenia tecta
DTr dorsal transition zone
E/OV ependyma and subependymal layer /olfactory ventricle
fmi forceps major of corpus callosum
IL infralimbic cortex
MCtx motor cortex
Pir piriform cortex
PrL prelimbic cortex

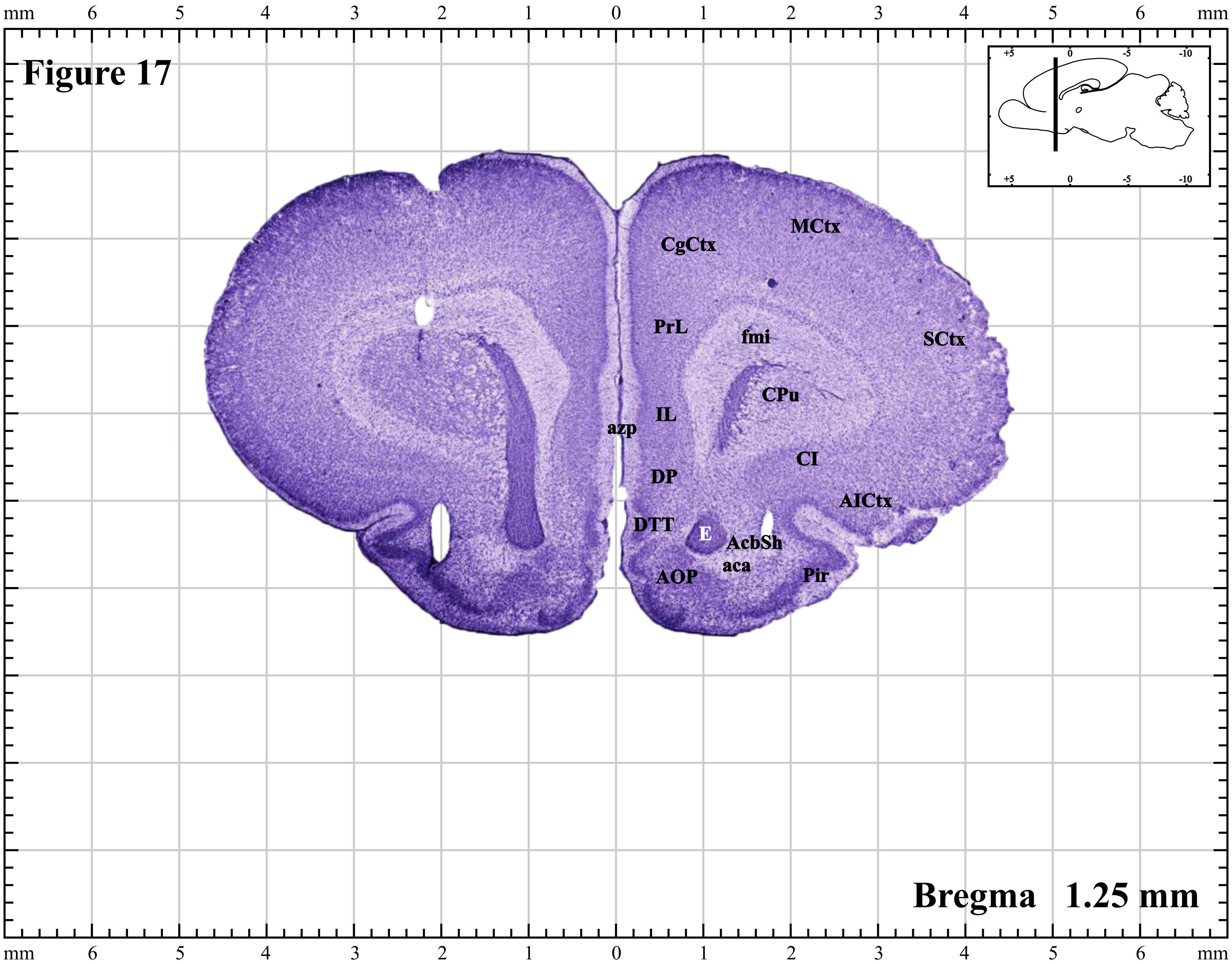

azp azygous pericallosal artery
aca anterior commissure, anterior part
AcbSh accumbens shell
AOP anterior olfactory nucleus, posterior part
AICtx agranular insular cortex
CgCtx cingulate cortex
CI claustrum
CPu caudate putamen (striatum)
DP dorsal peduncular cortex
DTT dorsal tenia tecta
IL infralimbic cortex
E ependyma and subependymal layer
fmi forceps major of corpus callosum
MCtx motor cortex
Pir piriform cortex
PrL prelimbic cortex
SCtx somatosensory cortex

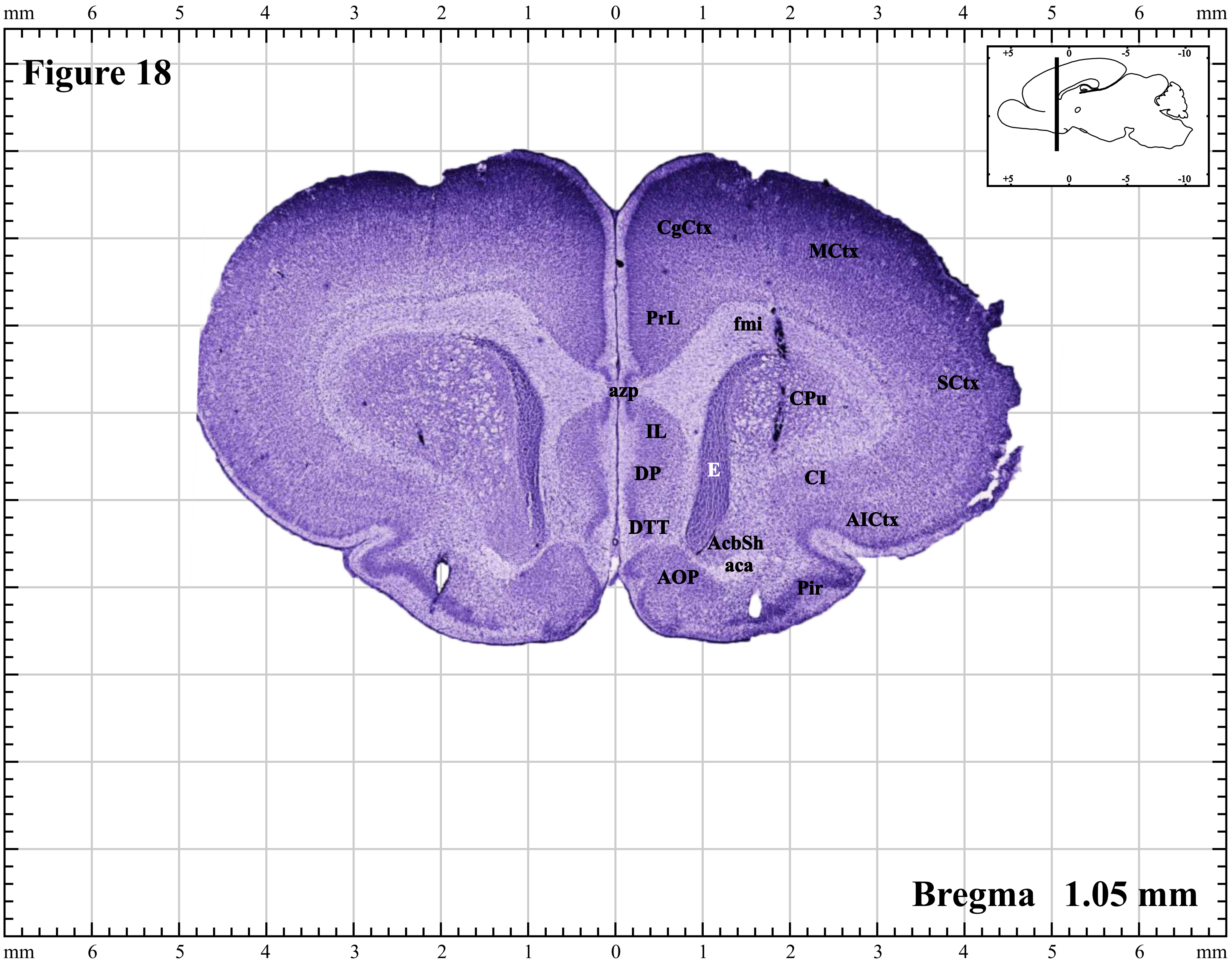

azp azygous pericallosal artery
aca anterior commissure, anterior part
AcbSh accumbens shell
AOP anterior olfactory nucleus, posterior part
AICtx agranular insular cortex
CgCtx cingulate cortex
CI claustrum
CPu caudate putamen (striatum)
DP dorsal peduncular cortex
DTT dorsal tenia tecta
E ependyma and subependymal layer
fmi forceps major of corpus callosum
IL infralimbic cortex
MCtx motor cortex
Pir piriform cortex
PrL prelimbic cortex
SCtx somatosensory cortex

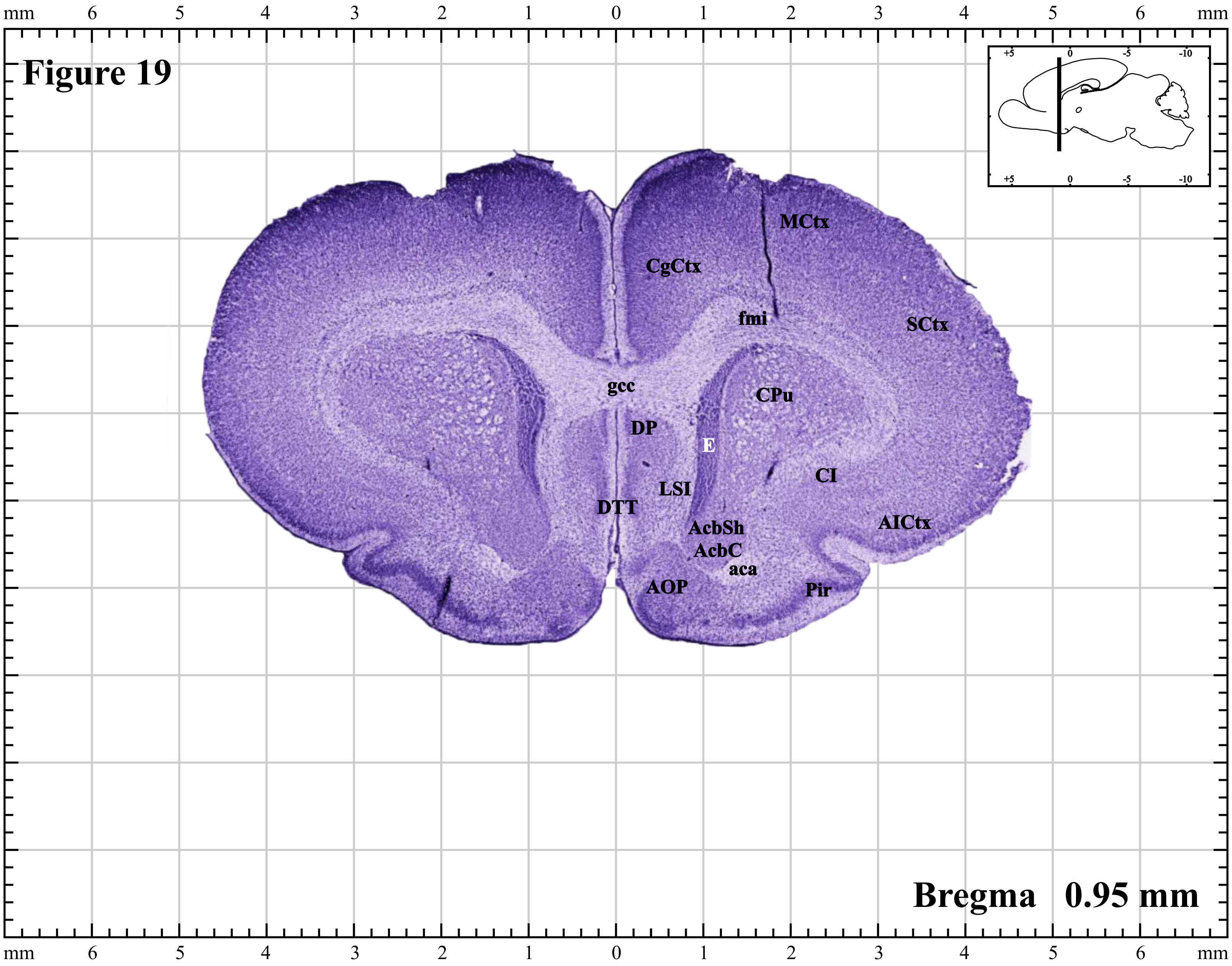

azp azygous pericallosal artery
AcbC accumbens nucleus, core
AcbSh accumbens shell
AOP anterior olfactory nucleus, posterior part
AICtx agranular insular cortex
CgCtx cingulate cortex
CI claustrum
CPu caudate putamen (striatum)
DP dorsal peduncular cortex
DTT dorsal tenia tecta
E ependyma and subependymal layer
fmi forceps major of corpus callosum
gcc genu of the corpus callosum
LSI lateral septal nucleus, intermediate part
MCtx motor cortex
Pir piriform cortex
SCtx somatosensory cortex

Figure 20

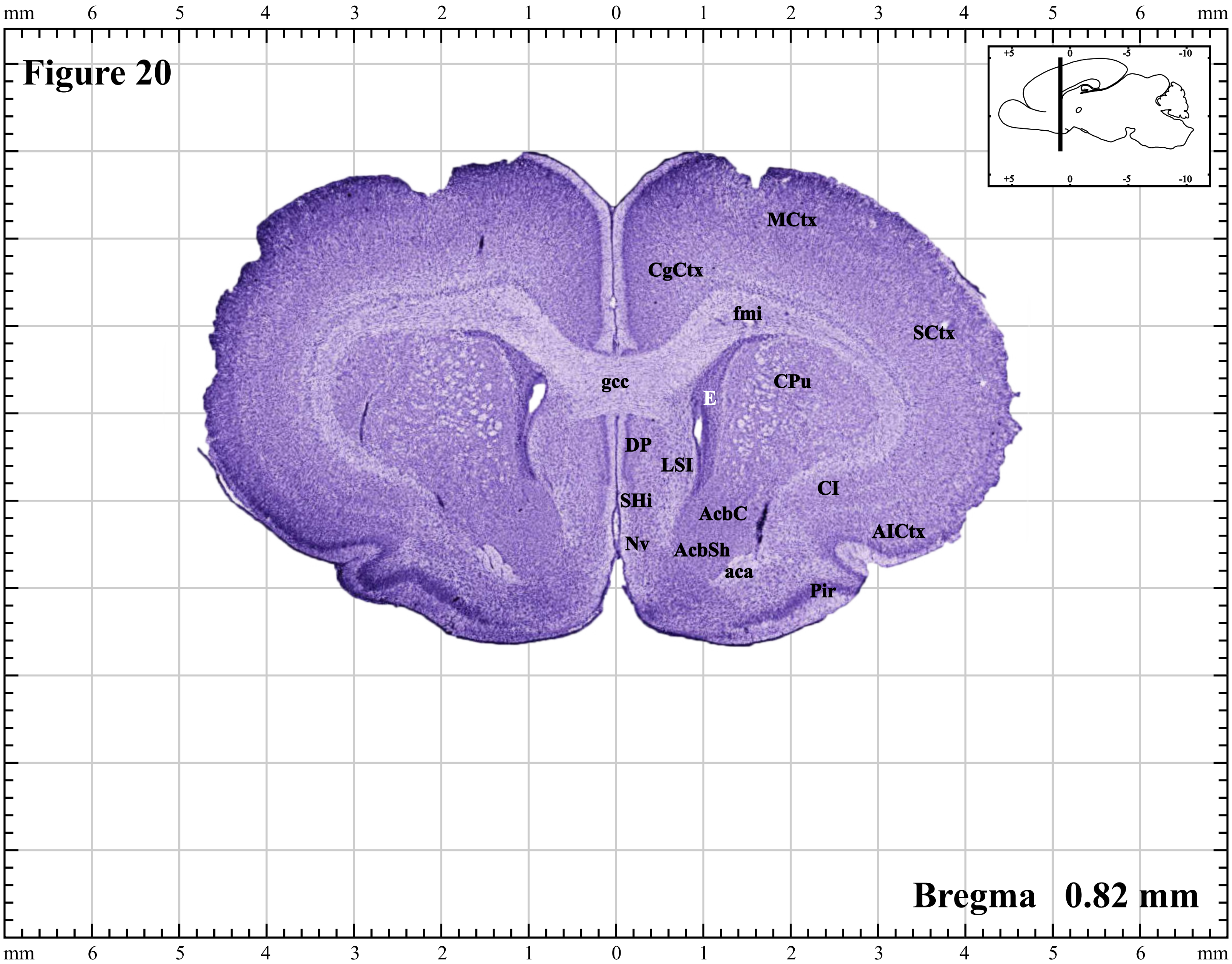

Bregma 0.82 mm

azp azygous pericallosal artery
AcbC accumbens nucleus, core
AcbSh accumbens shell
AICtx agranular insular cortex
CgCtx cingulate cortex
CI claustrum
CPu caudate putamen (striatum)
DP dorsal peduncular cortex
E ependyma and subependymal layer
fmi forceps major of corpus callosum
gcc genu of the corpus callosum
LSI lateral septal nucleus, intermediate part
MCtx motor cortex
Nv navicular nucleus of the basal forebrain
Pir piriform cortex
SHi septohippocampal nucleus
SCtx somatosensory cortex

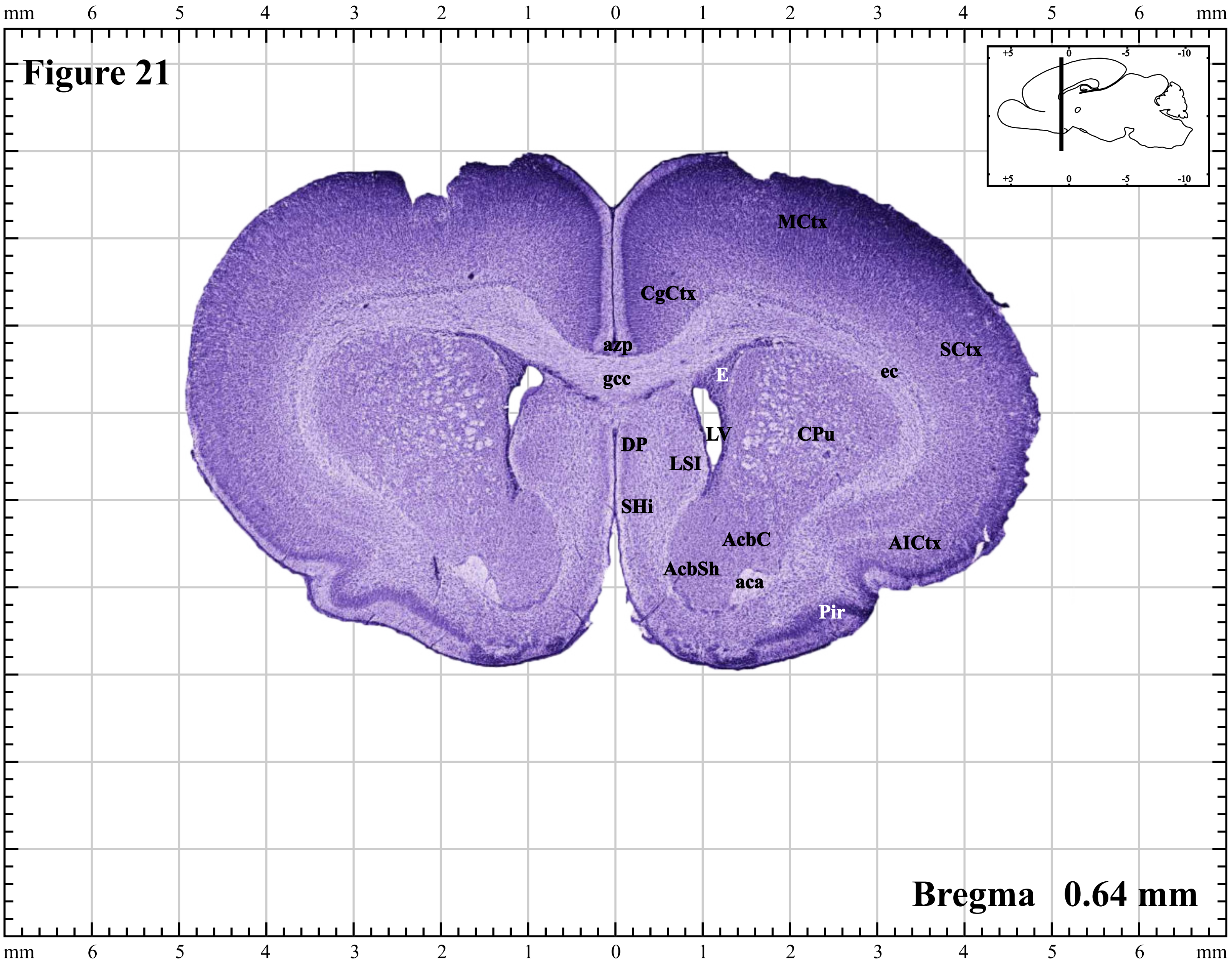

aca anterior commissure, anterior part
azp azygous pericallosal artery
AcbC accumbens nucleus, core
AcbSh accumbens shell
AICtx agranular insular cortex
CgCtx cingulate cortex
CPu caudate putamen (striatum)
DP dorsal peduncular cortex
ec external capsule
E ependyma and subependymal layer
gcc genu of the corpus callosum
fmi forceps major of corpus callosum
LV lateral ventricle
LSI lateral septal nucleus, intermediate part
MCtx motor cortex
Pir piriform cortex
SCtx somatosensory cortex
SHi septohippocampal nucleus

Figure 22

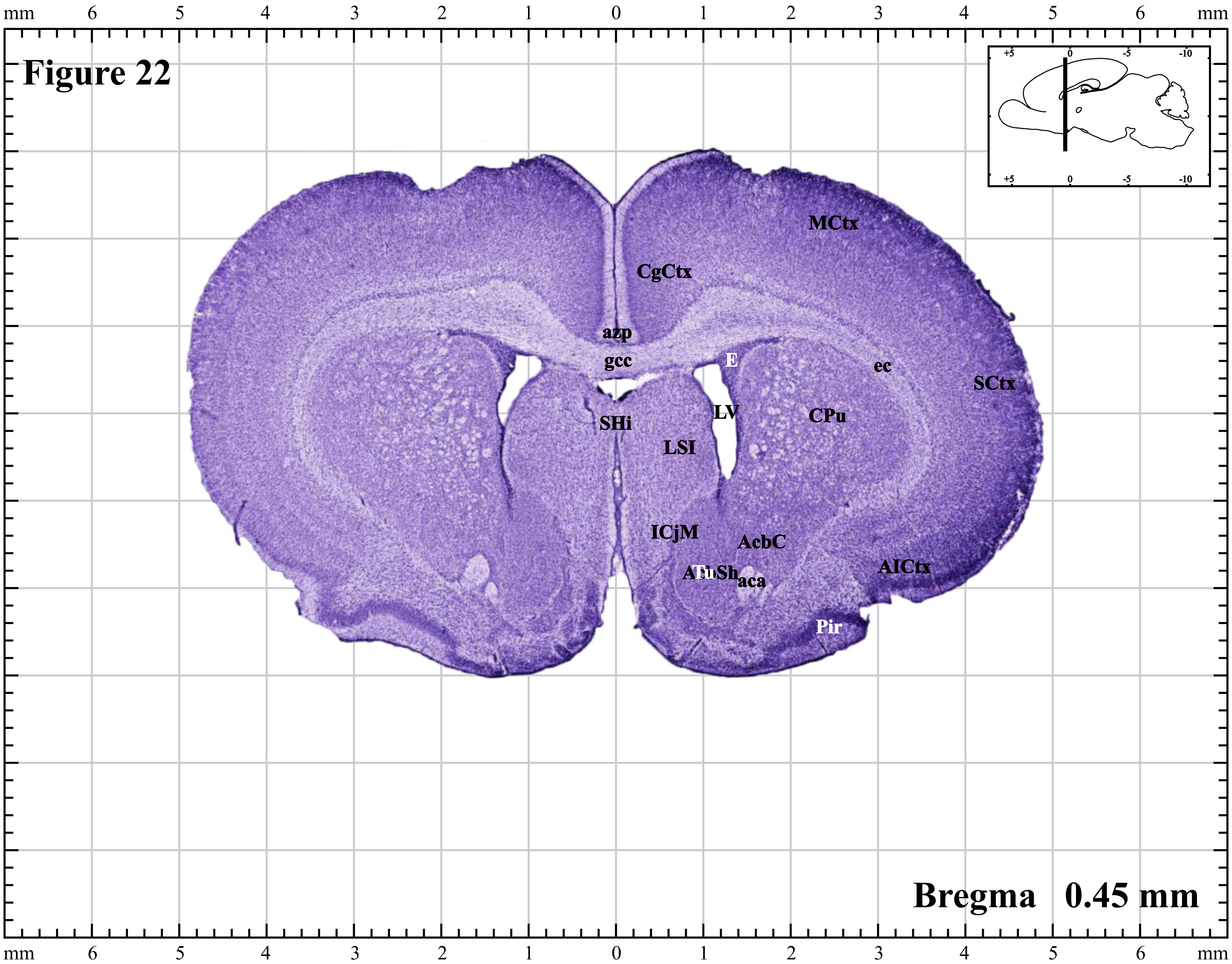

Bregma 0.45 mm

aca anterior commissure, anterior part
azp azygous pericallosal artery
AcbC accumbens nucleus, core
AcbSh accumbens shell
AICtx agranular insular cortex
CgCtx cingulate cortex
CPu caudate putamen (striatum)
ec external capsule
E ependyma and subependymal layer
gcc genu of the corpus callosum
ICjM islands of Calleja, major island
LV lateral ventricle
LSI lateral septal nucleus, intermediate part
MCtx motor cortex
Pir piriform cortex
SCtx somatosensory cortex
SHi septohippocampal nucleus
Tu olfactory tubercle

Figure 23

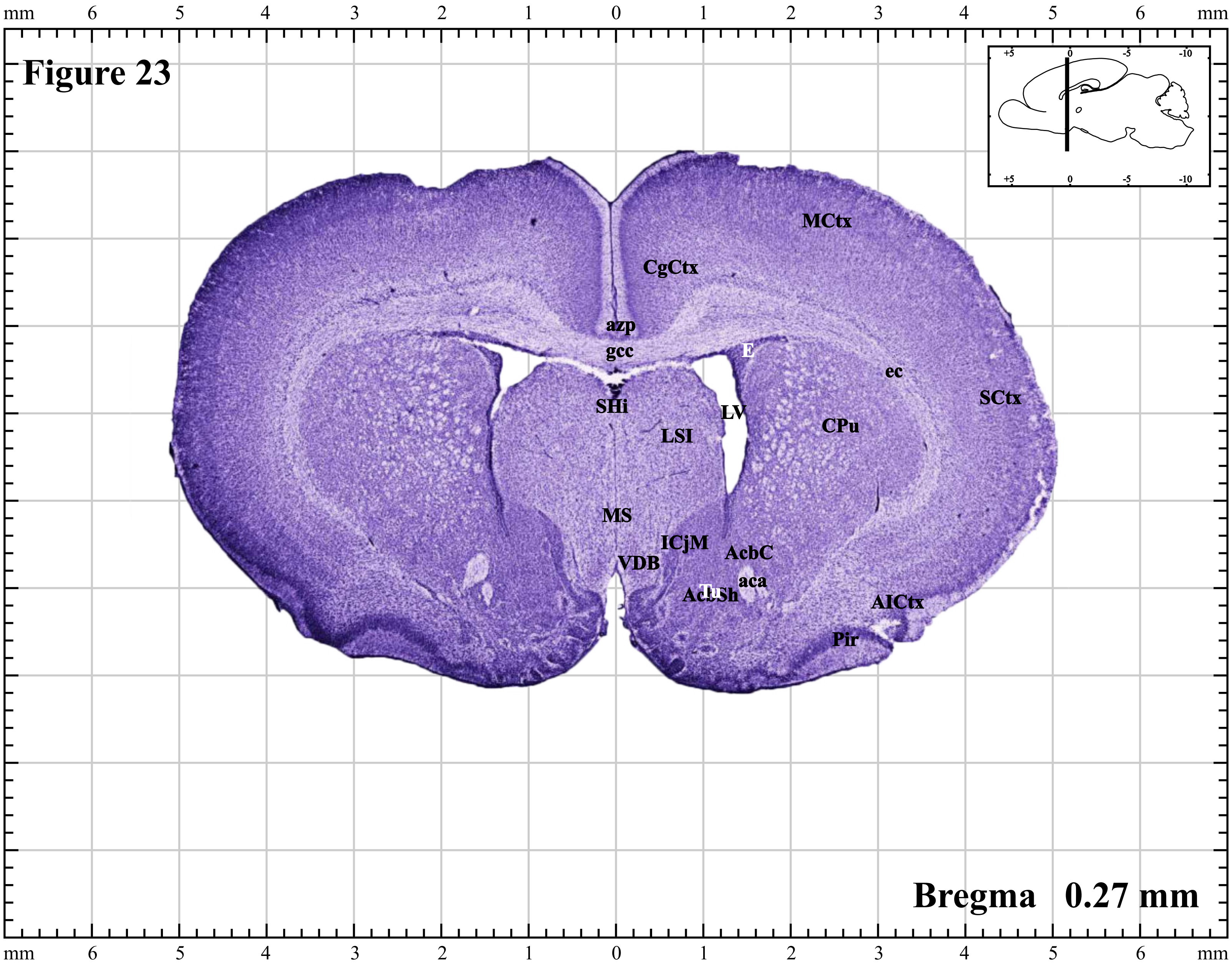

Bregma 0.27 mm

aca anterior commissure, anterior part
azp azygous pericallosal artery
AcbC accumbens nucleus, core
AcbSh accumbens shell
AICtx agranular insular cortex
CgCtx cingulate cortex
CPu caudate putamen (striatum)
ec external capsule
E ependyma and subependymal layer
gcc genu of the corpus callosum
ICjM islands of Calleja, major island
LV lateral ventricle
LSI lateral septal nucleus, intermediate part
MCtx motor cortex
MS medial septal nucleus
Pir piriform cortex
SCtx somatosensory cortex
SHi septohippocampal nucleus
Tu olfactory tubercle
VDB nucleus of the vertical limb of the diagonal band

Figure 24

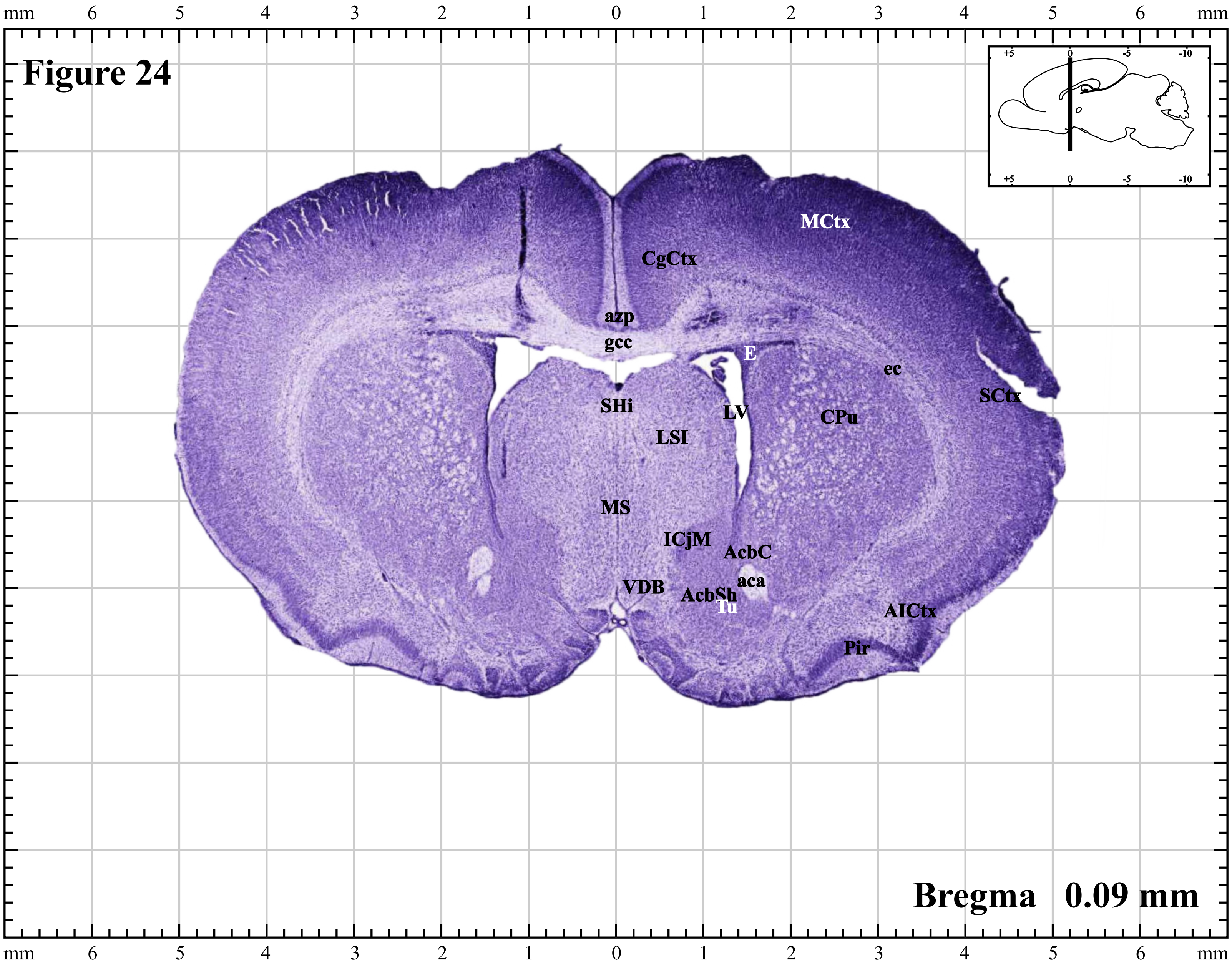

Bregma 0.09 mm

aca anterior commissure, anterior part
azp azygous pericallosal artery
AcbC accumbens nucleus, core
AcbSh accumbens shell
AICtx agranular insular cortex
CgCtx cingulate cortex
CPu caudate putamen (striatum)
ec external capsule
E ependyma and subependymal layer
gcc genu of the corpus callosum
ICjM islands of Calleja, major island
LV lateral ventricle
LSI lateral septal nucleus, intermediate part
MCtx motor cortex
MS medial septal nucleus
Pir piriform cortex
SCtx somatosensory cortex
SHi septohippocampal nucleus
Tu olfactory tubercle
VDB nucleus of the vertical limb of the diagonal band

Figure 25

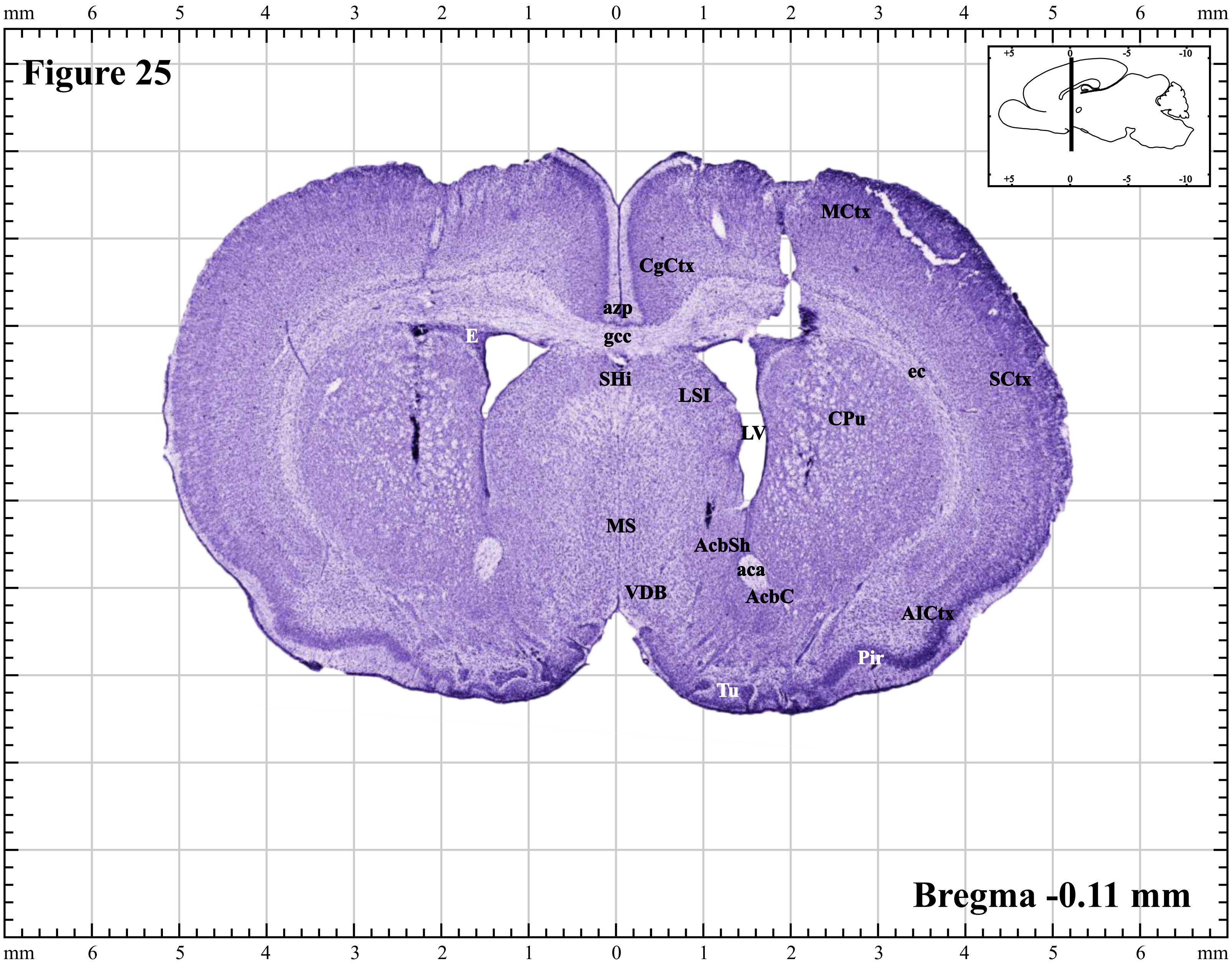

Bregma -0.11 mm

aca anterior commissure, anterior part
azp azygous pericallosal artery
AcbC accumbens nucleus, core
AcbSh accumbens shell
AICtx agranular insular cortex
CgCtx cingulate cortex
CPu caudate putamen (striatum)
ec external capsule
E ependyma and subependymal layer
gcc genu of the corpus callosum
LV lateral ventricle
LSI lateral septal nucleus, intermediate part
MS medial septal nucleus
MCtx motor cortex
Pir piriform cortex
SCtx somatosensory cortex
SHi septohippocampal nucleus
Tu olfactory tubercle
VDB nucleus of the vertical limb of the diagonal band

Figure 26

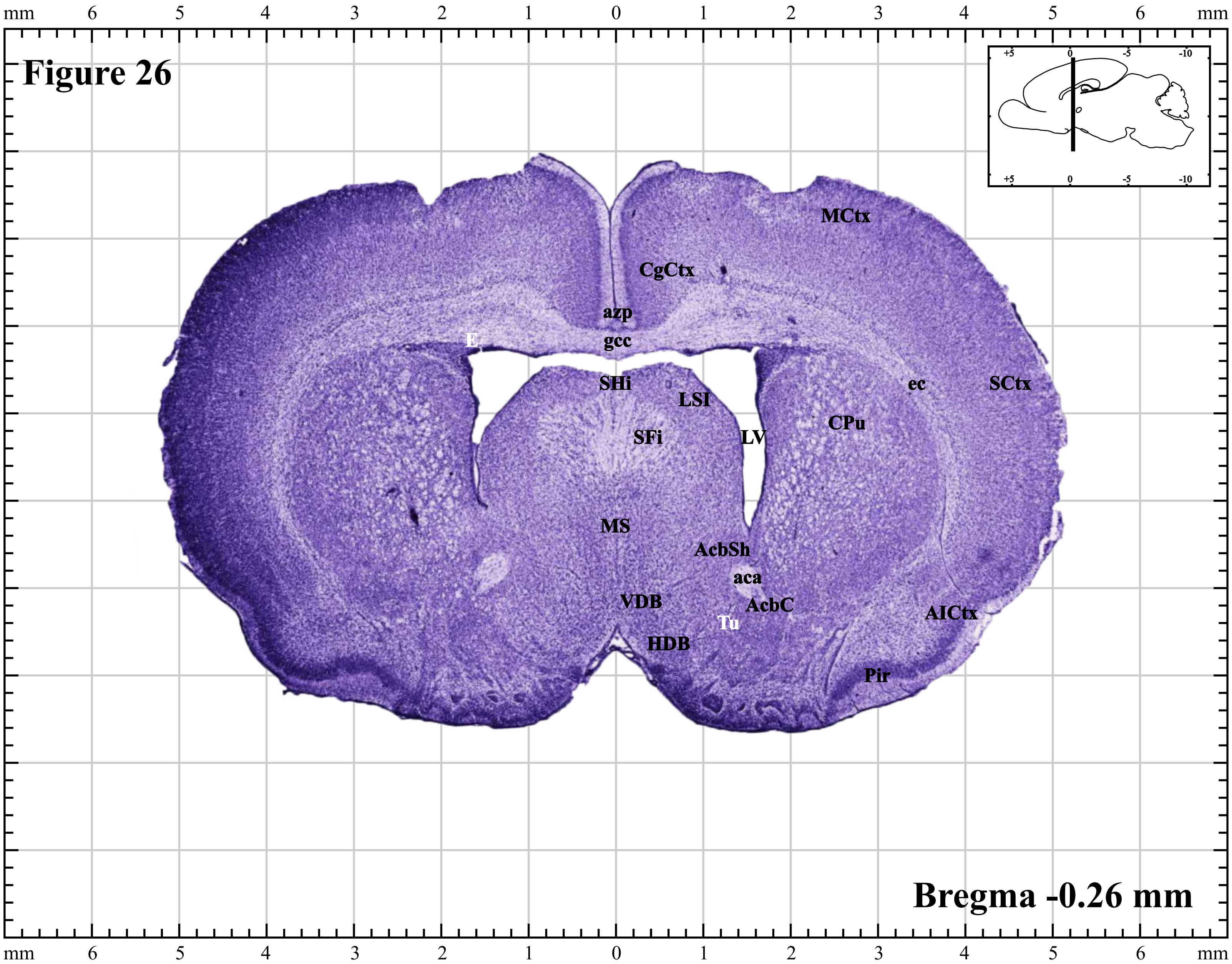

Bregma -0.26 mm

aca anterior commissure, anterior part
azp azygous pericallosal artery
AcbC accumbens nucleus, core
AcbSh accumbens shell
AICtx agranular insular cortex
CgCtx cingulate cortex
CPu caudate putamen (striatum)
ec external capsule
E ependyma and subependymal layer
gcc genu of the corpus callosum
HDB nucleus of the horizontal limb of the diagonal band
LV lateral ventricle
LSI lateral septal nucleus, intermediate part
MS medial septal nucleus
MCtx motor cortex
Pir piriform cortex
SCtx somatosensory cortex
SHi septohippocampal nucleus
SFi septofimbrial nucleus
Tu olfactory tubercle
VDB nucleus of the vertical limb of the diagonal band

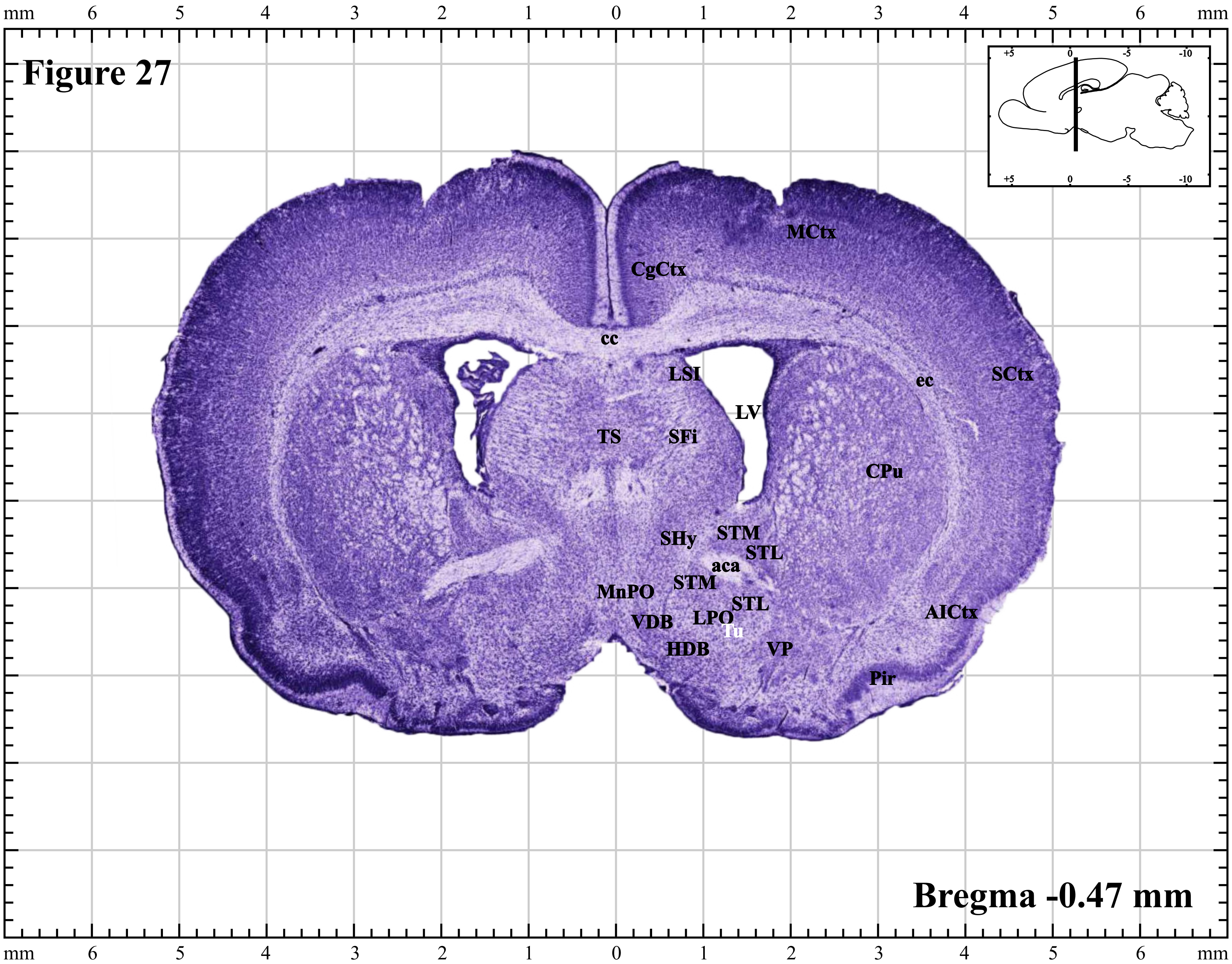

aca anterior commissure, anterior part
AICtx agranular insular cortex
cc corpus callosum
CPu caudate putamen
Cgctx cingulate cortex
ec external capsule
HDB nucleus of the horizontal limb of the diagonal band
LPO lateral preoptic area
LSI lateral septal nucleus, intermediate part
LV lateral ventricle
MnPO median preoptic nucleus
MCtx motor cortex
MnPO median preoptic nucleus
Pir piriform cortex
SCtx somatosensory cortex
SHy septohypothalamic nucleus
SFi septofimbrial nucleus
STM bed nucleus of the stria terminalis, medial division
STL bed nucleus of the stria terminalis, lateral division
TS triangular septal nucleus
Tu olfactory tubercle
VP ventral pallidum
VDB nucleus of the vertical limb of the diagonal band

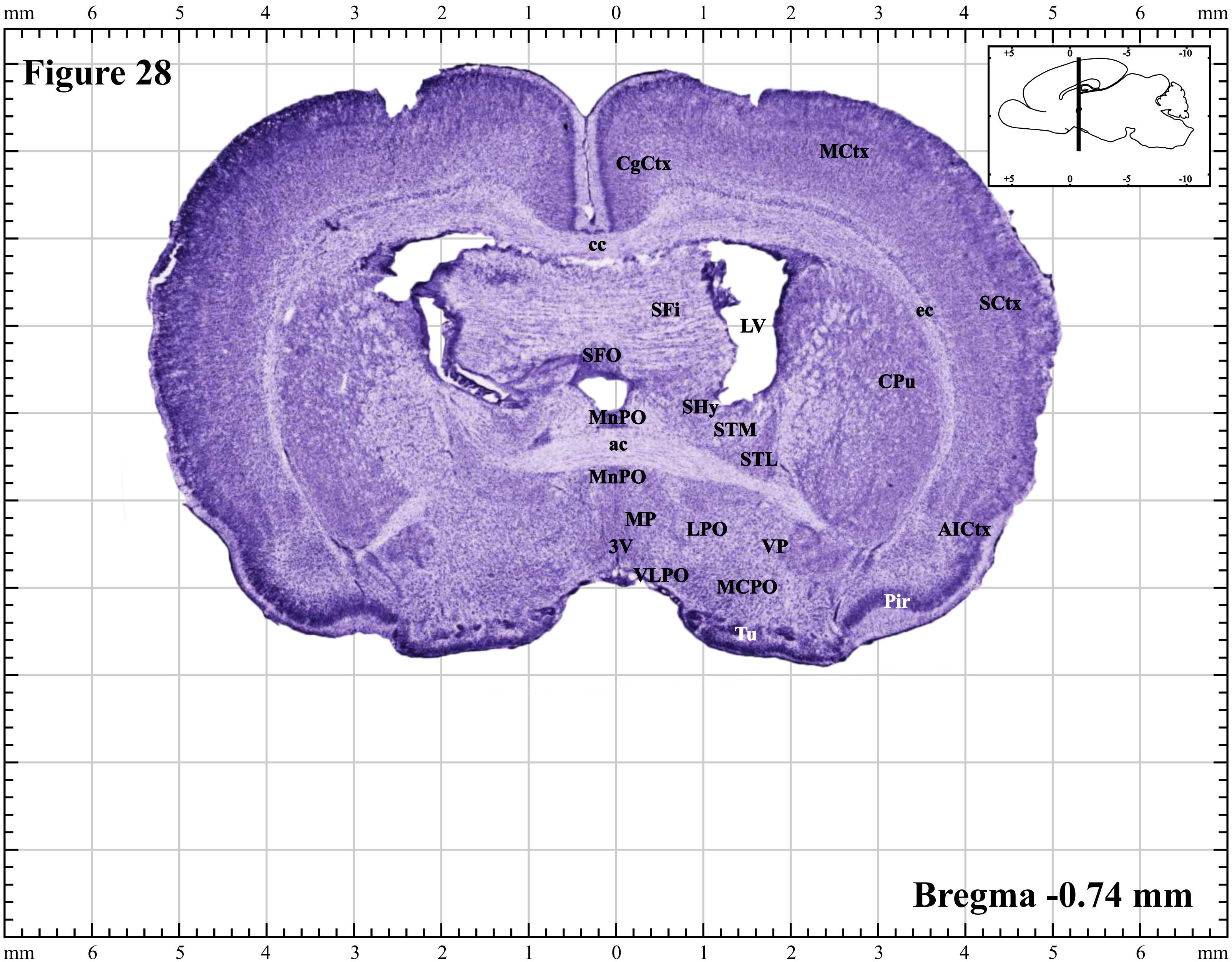

3V 3rd ventricle
ac anterior commissure
AICtx agranular insular cortex
cc corpus callosum
CPu caudate putamen
Cgctx cingulate cortex
ec external capsule
LPO lateral preoptic area
LV lateral ventricle
MP medial preoptic nucleus
MnPO median preoptic nucleus
MCtx motor cortex
MS medial septal nucleus
och optic chiasm
Pir piriform cortex
MCPO magnocellular preoptic nucleus
SCtx somatosensory cortex
SFO subfornical organ
SHy septohypothalamic nucleus
SFi septofimbrial nucleus
STL bed nucleus of the stria terminalis, lateral division
STM bed nucleus of the stria terminalis, medial division
Tu olfactory tubercle
VP ventral pallidum
VLPO ventrolateral preoptic nucleus

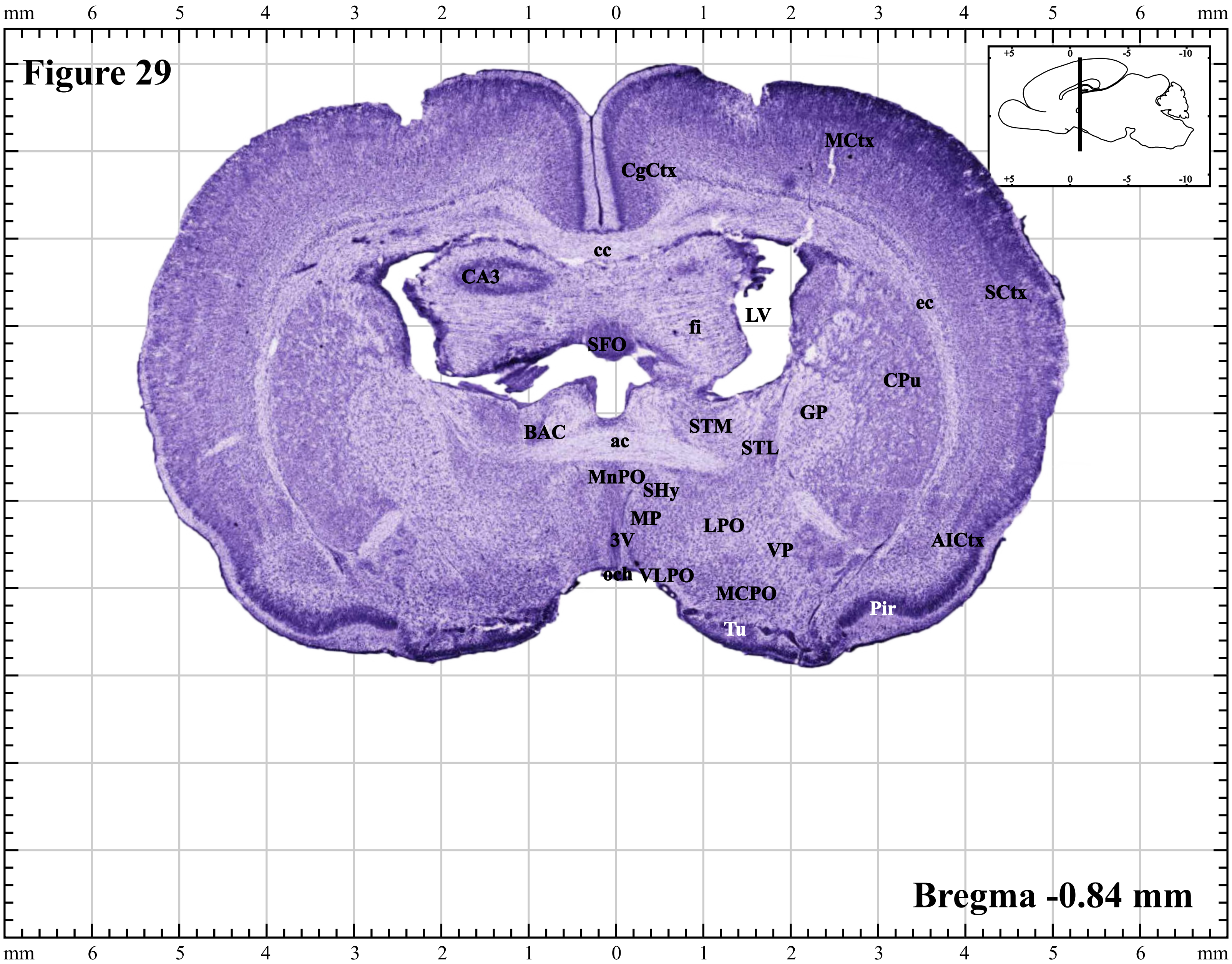

3V 3rd ventricle
ac anterior commissure
AICtx agranular insular cortex
BAC bed nucleus of the anterior commissure
cc corpus callosum
CPu caudate putamen
Cgctx cingulate cortex
CA3 field CA3 of the hippocampus
ec external capsule
fi fimbria of the hippocampus
GP globus pallidus
LV lateral ventricle
LPO lateral preoptic area
MP medial preoptic nucleus
MnPO median preoptic nucleus
MCtx motor cortex
MS medial septal nucleus
och optic chiasm
Pir piriform cortex
MCPO magnocellular preoptic nucleus
SCtx somatosensory cortex
SFO subfornical organ
STL bed nucleus of the stria terminalis, lateral division
STM bed nucleus of the stria terminalis, medial division
SHy septohypothalamic nucleus
Tu olfactory tubercle
VP ventral pallidum
VLPO ventrolateral preoptic nucleus

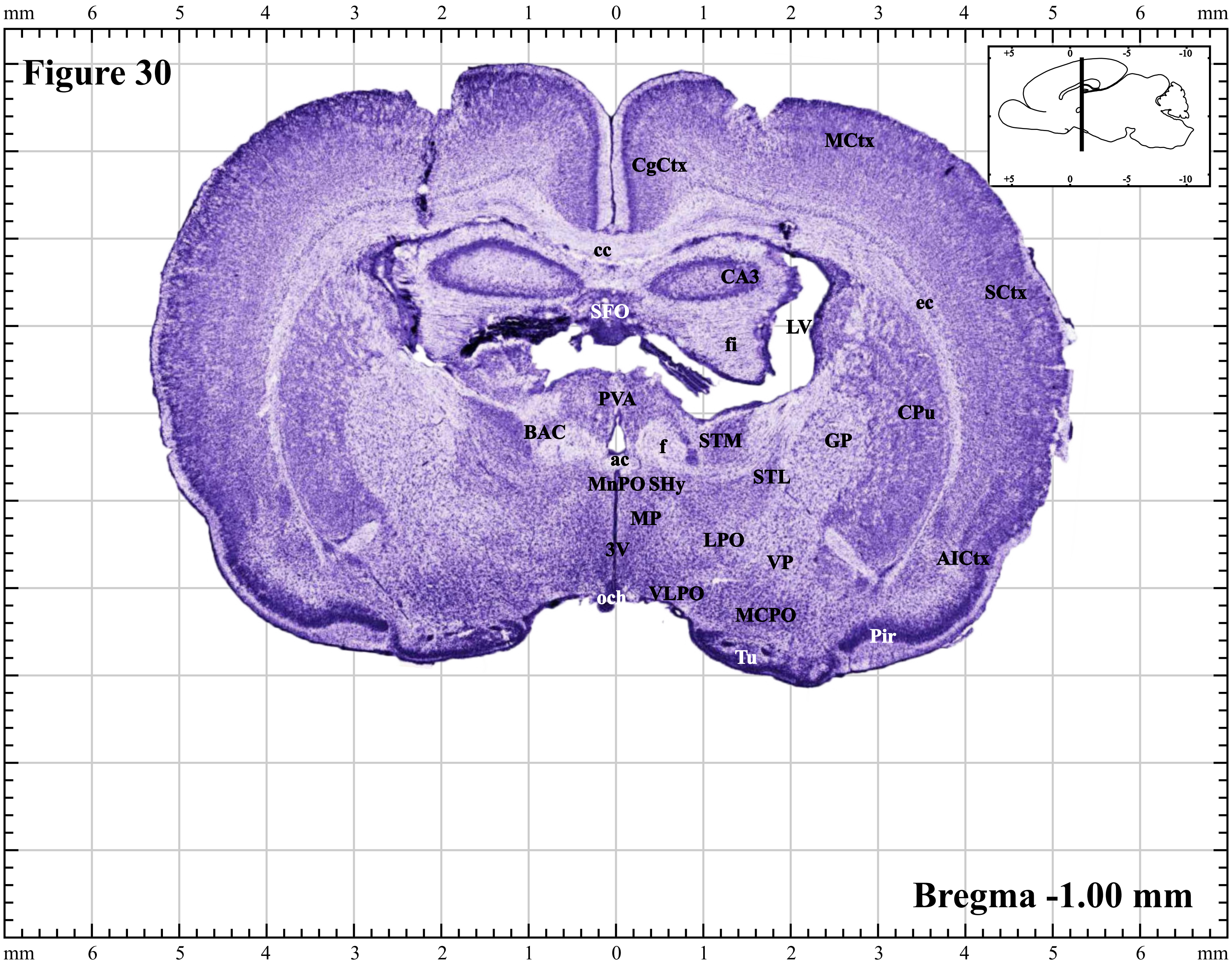

3V 3rd ventricle
ac anterior commissure
AICtx agranular insular cortex
BAC bed nucleus of the anterior commissure
cc corpus callosum
CPu caudate putamen
Cgctx cingulate cortex
CA3 field CA3 of the hippocampus
ec external capsule
f fornix
fi fimbria of the hippocampus
GP globus pallidus
LV lateral ventricle
LPO lateral preoptic area
MP medial preoptic nucleus
MnPO median preoptic nucleus
MCtx motor cortex
MS medial septal nucleus
och optic chiasm
Pir piriform cortex
MCPO magnocellular preoptic nucleus
SCtx somatosensory cortex
STL bed nucleus of the stria terminalis, lateral division
STM bed nucleus of the stria terminalis, medial division
SFO subfornical organ
SHy septohypothalamic nucleus
Tu olfactory tubercle
VP ventral pallidum
VLPO ventrolateral preoptic nucleus

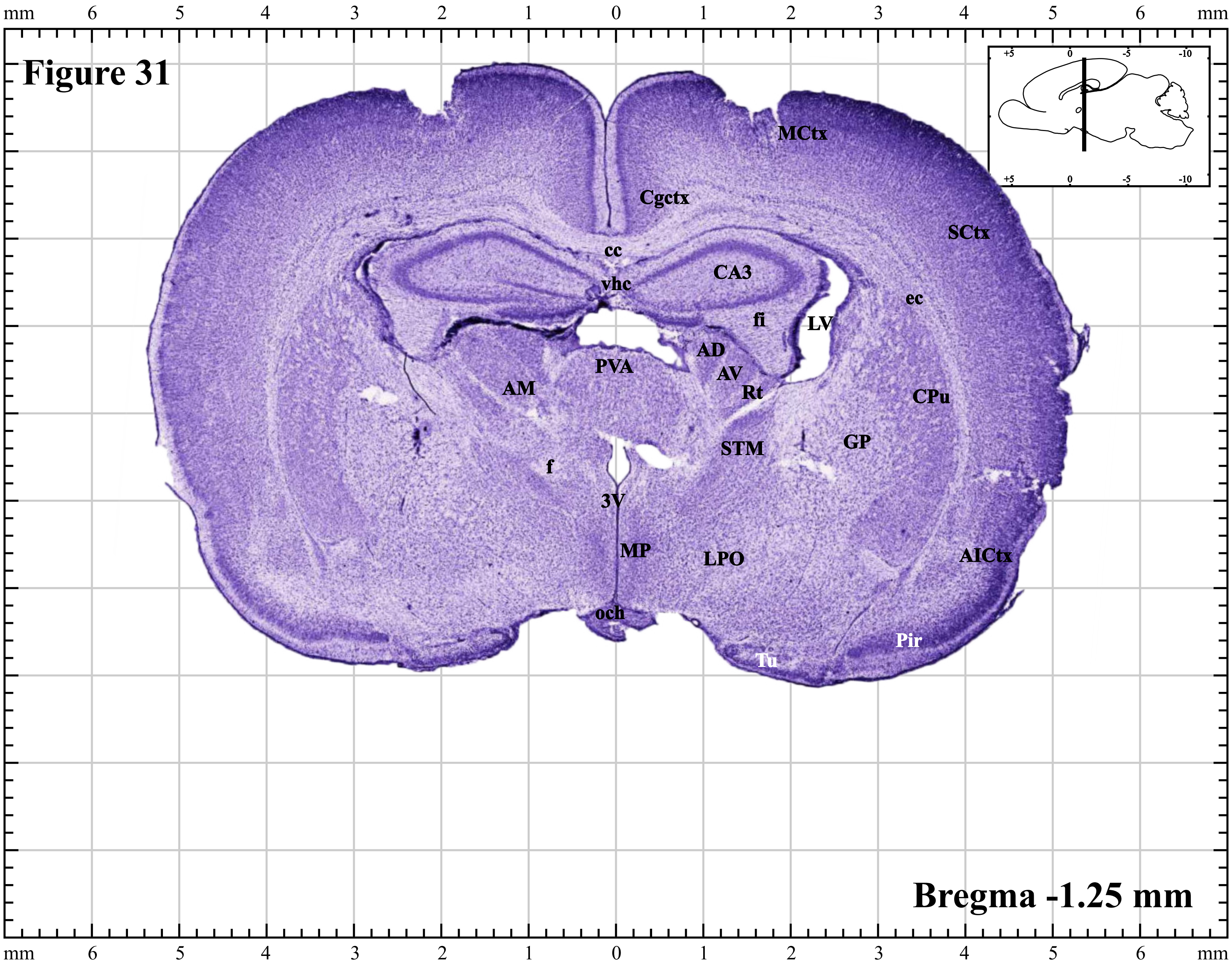

3V 3rd ventricle
AICtx agranular insular cortex
AD anterodorsal thalamic nucleus
AM anteromedial thalamic nucleus
AV anteroventral thalamic nucleus
cc corpus callosum
CPu caudate putamen
Cgctx cingulate cortex
CA3 field CA3 of the hippocampus
ec external capsule
GP globus pallidus
f fornix
fi fimbria of the hippocampus
LPO lateral preoptic area
LV lateral ventricle
MCtx motor cortex
MP medial preoptic nucleus
och optic chiasm
PVA paraventricular thalamic nucleus, anterior part
Pir piriform cortex
Rt reticular thalamic nucleus
SCtx somatosensory cortex
STM bed nucleus of the stria terminalis, medial division
Tu olfactory tubercle
vhc ventral hippocampal commissure

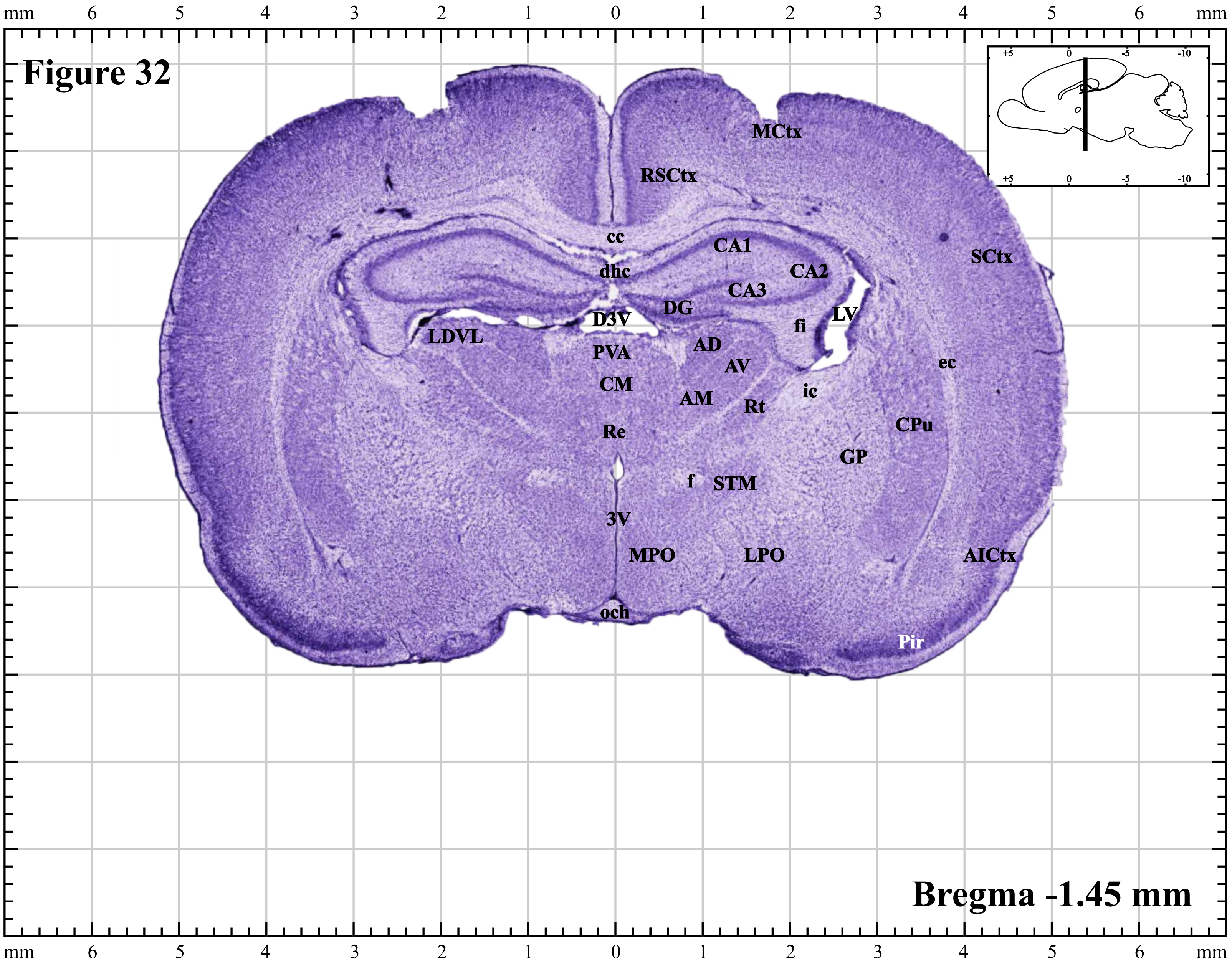

3V 3rd ventricle
AICtx agranular insular cortex
AD anterodorsal thalamic nucleus
AM anteromedial thalamic nucleus
AV anteroventral thalamic nucleus
cc corpus callosum
CPu caudate putamen
CA1 field CA1 of the hippocampus
CA2 field CA2 of the hippocampus
CA3 field CA3 of the hippocampus
CM central medial thalamic nucleus
dhc dorsol hippocampal commissure
D3V dorsal 3rd ventricle
ec external capsule
fi fimbria of the hippocampus
f fornix
GP globus pallidus
ic internal capsule
LV lateral ventricle
LDVL laterodorsal thalamic nucleus, ventrolateral part
LPO lateral preoptic area
MCtx motor cortex
MPO medial preoptic nucleus
och optic chiasm
PVA paraventricular thalamic nucleus, anterior part
Pir piriform cortex
Rt reticular thalamic nucleus
Re reuniens thalamic nucleus
SCtx somatosensory cortex
STM bed nucleus of the stria terminalis, medial division
RSCtx retrospenial cortex

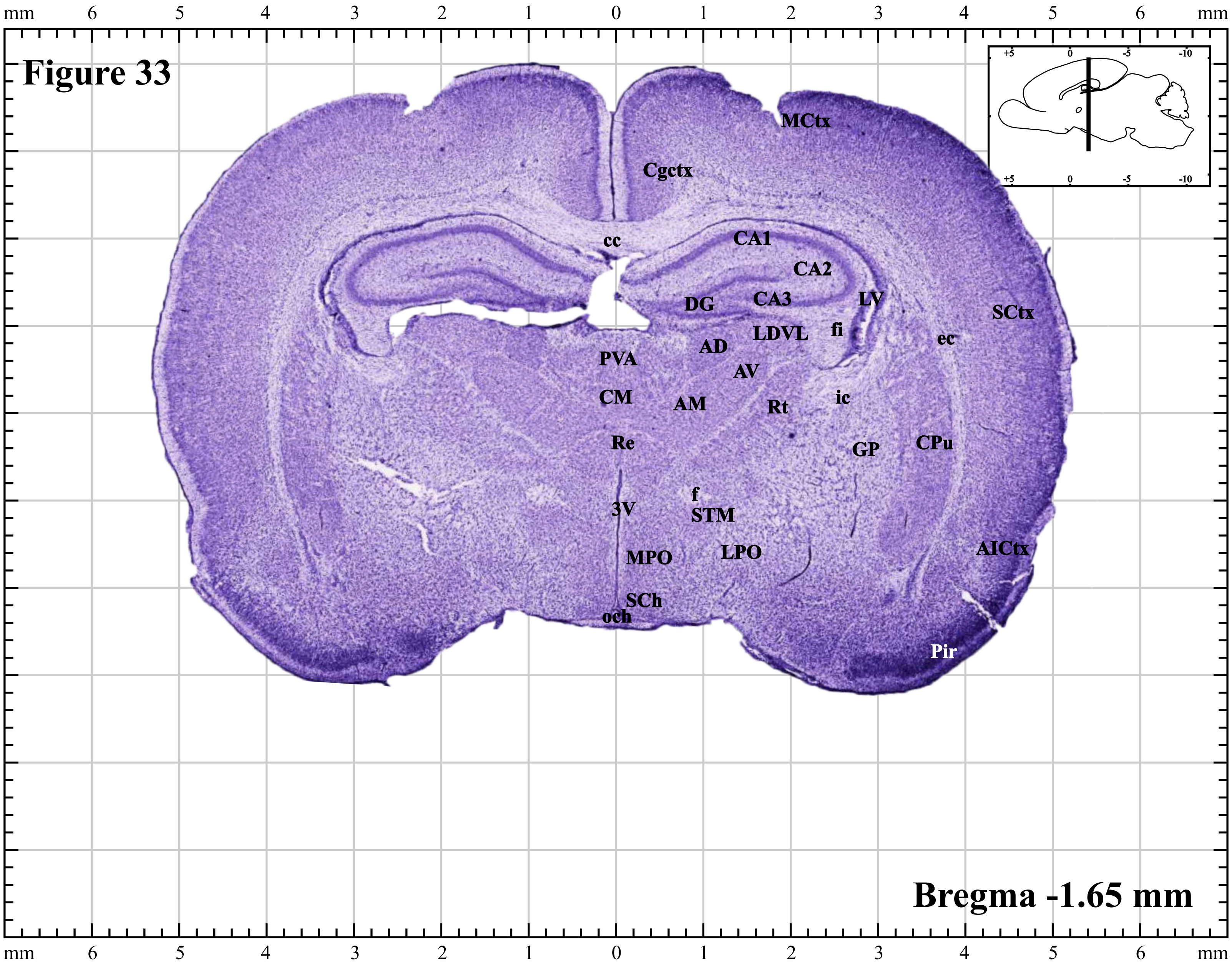

3V 3rd ventricle
AD anterodorsal thalamic nucleus
AM anteromedial thalamic nucleus
AV anteroventral thalamic nucleus
AICtx agranular insular cortex
cc corpus callosum
CPu caudate putamen
Cgctx cingulate cortex
CM central medial thalamic nucleus
CA1 field CA1 of the hippocampus
CA2 field CA2 of the hippocampus
CA3 field CA3 of the hippocampus
DG dentate gyrus
ec external capsule
fi fimbria of the hippocampus
f fornix
GP globus pallidus
ic internal capsule
LPO lateral preoptic area
LV lateral ventricle
LDVL laterodorsal thalamic nucleus, ventrolateral part
MPO medial preoptic nucleus
MCtx motor cortex
Rt reticular thalamic nucleus
och optic chiasm
PVA paraventricular thalamic nucleus, anterior part
Pir piriform cortex
SCh suprachiasmatic nucleus
STM bed nucleus of the stria terminalis, medial division
SCtx somatosensory cortex
Re reuniens thalamic nucleus

Figure 34

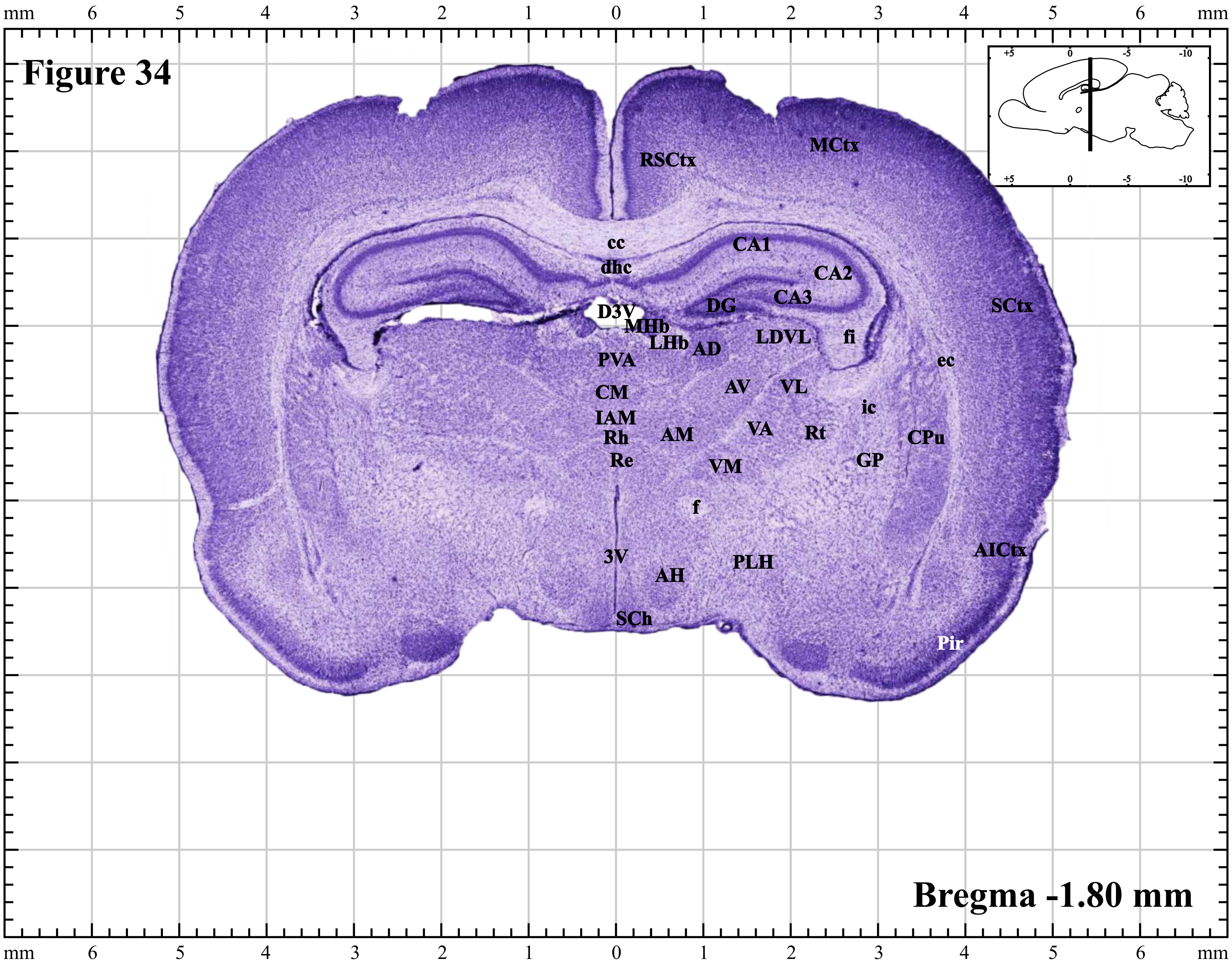

Bregma -1.80 mm

3V 3rd ventricle
AICtx agranular insular cortex
AD anterodorsal thalamic nucleus
AM anteromedial thalamic nucleus
AV anteroventral thalamic nucleus
AH anterior hypothalamic area
cc corpus callosum
CPu caudate putamen
CA1 field CA1 of the hippocampus
CA2 field CA2 of the hippocampus
CA3 field CA3 of the hippocampus
CM central medial thalamic nucleus
DG dentate gyrus
dhc dorsol hippocampal commissure
D3V dorsal 3rd ventricle
ec external capsule
fi fimbria of the hippocampus
f fornix
GP globus pallidus
ic internal capsule
IAM interanteromedial thalamic nucleus
LDVL laterodorsal thalamic nucleus, ventrolateral part
LHb lateral habenular nucleus
MCtx motor cortex
MHb medial habenular nucleus
PVA paraventricular thalamic nucleus, anterior part
Pir piriform cortex
PLH peduncular part of lateral hypothalamus
Rt reticular thalamic nucleus
Re reuniens thalamic nucleus
SCtx somatosensory cortex
SCh suprachiasmatic nucleus
RSCtx retrospenial cortex
Rh rhomboid thalamic nucleus
VA ventral anterior thalamic nucleus
VM ventromedial thalamic nucleus
VL ventrolateral thalamic nucleus

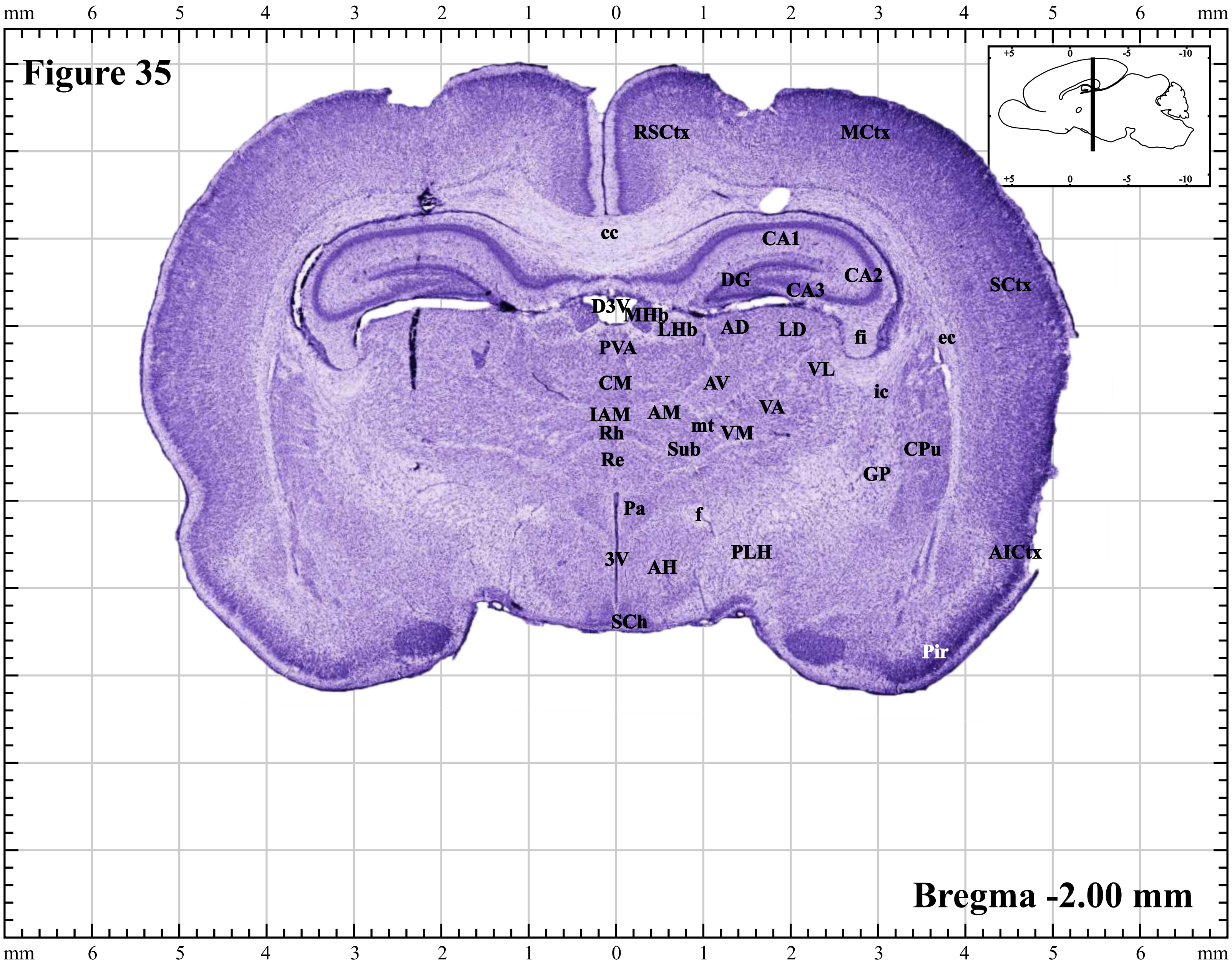

Figure 35

Bregma -2.00 mm

3V 3rd ventricle
AICtx agranular insular cortex
AH anterior hypothalamic area
AM anteromedial thalamic nucleus
AV anteroventral thalamic nucleus
AIctx anteroventral thalamic nucleus
AV anteroventral thalamic nucleus
cc corpus callosum
CPu caudate putamen
CA1 field CA1 of the hippocampus
CA2 field CA2 of the hippocampus
CA3 field CA3 of the hippocampus
CM central medial thalamic nucleus
D3V dorsal 3rd ventricle
ec external capsule
f fornix
fi fimbria of the hippocampus
GP globus pallidus
ic internal capsule
DG dentate gyrus
IAM interanteromedial thalamic nucleus
LHb lateral habenular nucleus
LD laterodorsal thalamic nucleus,
mt mammillothalamic tract
MHb medial habenular nucleus
MCtx motor cortex
Pir piriform cortex
PVA paraventricular thalamic nucleus, anterior part
Pa paraventricular hypoth nucleus
PLH peduncular part of lateral hypothalamus
Rh rhomboid thalamic nucleus
Re reuniens thalamic nucleus
RSCtx retrospenial cortex
SCh suprachiasmatic nucleus
SCtx somatosensory cortex
Sub submedius thalamic nucleus
VA ventral anterior thalamic nucleus
VM ventromedial thalamic nucleus
VL ventrolateral thalamic nucleus

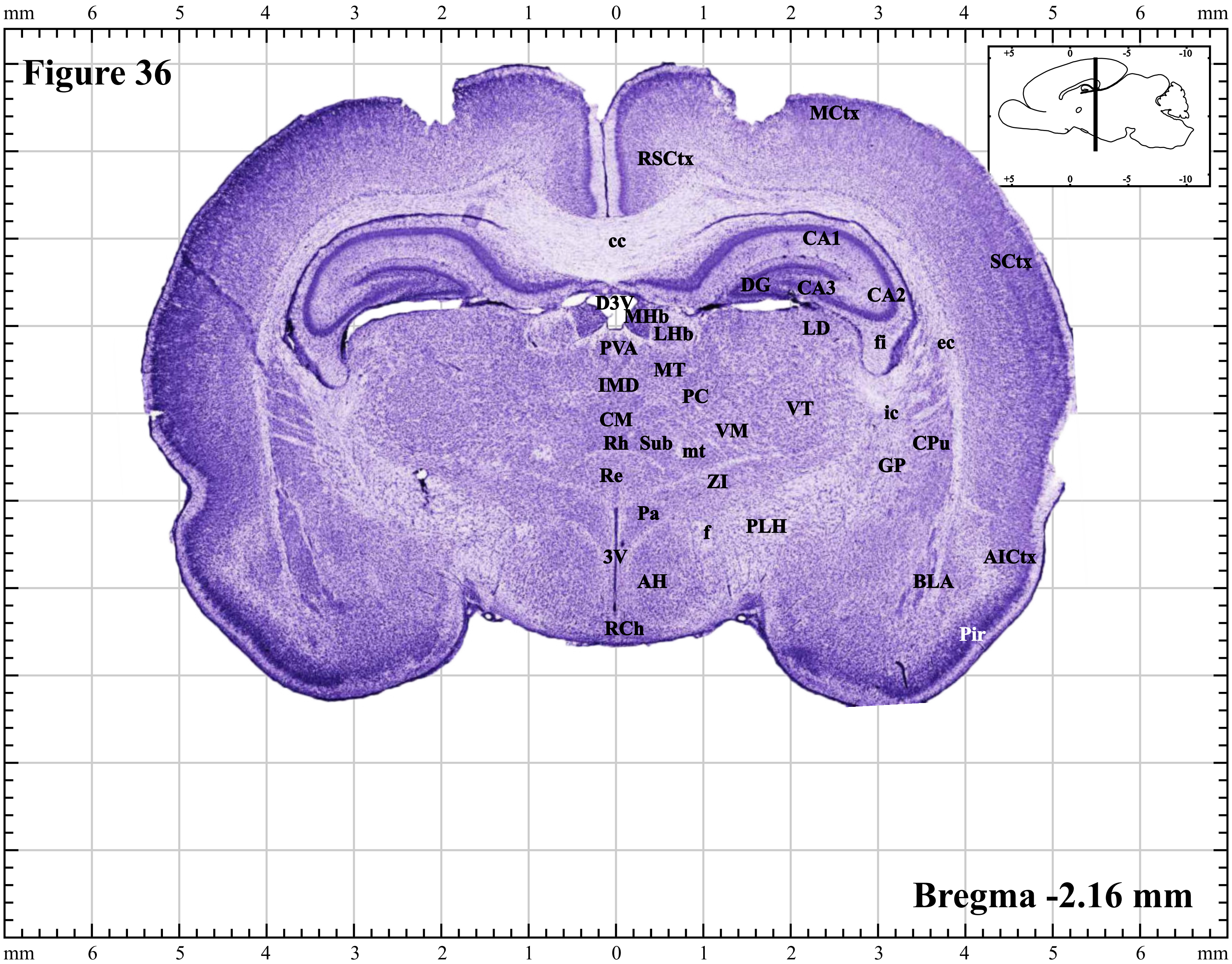

3V 3rd ventricle
AH anterior hypothalamic area
AICtx agranular insular cortex
BLA basolateral amygdaloid nucleus, anterior part
cc corpus callosum
CA1 field CA1 of the hippocampus
CA2 field CA2 of the hippocampus
CA3 field CA3 of the hippocampus
CPu caudate putamen
CM central medial thalamic nucleus
D3V dorsal 3rd ventricle
DG dentate gyrus
ec external capsule
f fornix
fi fimbria of the hippocampus
GP globus pallidus
ic internal capsule
IMD intermediodorsal thalamic nucleus
LHb lateral habenular nucleus
LD laterodorsal thalamic nucleus,
mt mammillothalamic tract
MHb medial habenular nucleus
MT medial thalamus
MCtx motor cortex
PC paracentral thalamic nucleus
Pir piriform cortex
PVA paraventricular thalamic nucleus, anterior part
Pa paraventricular hypoth nucleus
PLH peduncular part of lateral hypothalamus
Rh rhomboid thalamic nucleus
RCh retrochiasmatic area
RSCtx retrospenial cortex
Re reuniens thalamic nucleus
SCtx somatosensory cortex
Sub submedius thalamic nucleus
VT ventral thalamus
VM ventromedial thalamic nucleus
ZI zona incerta

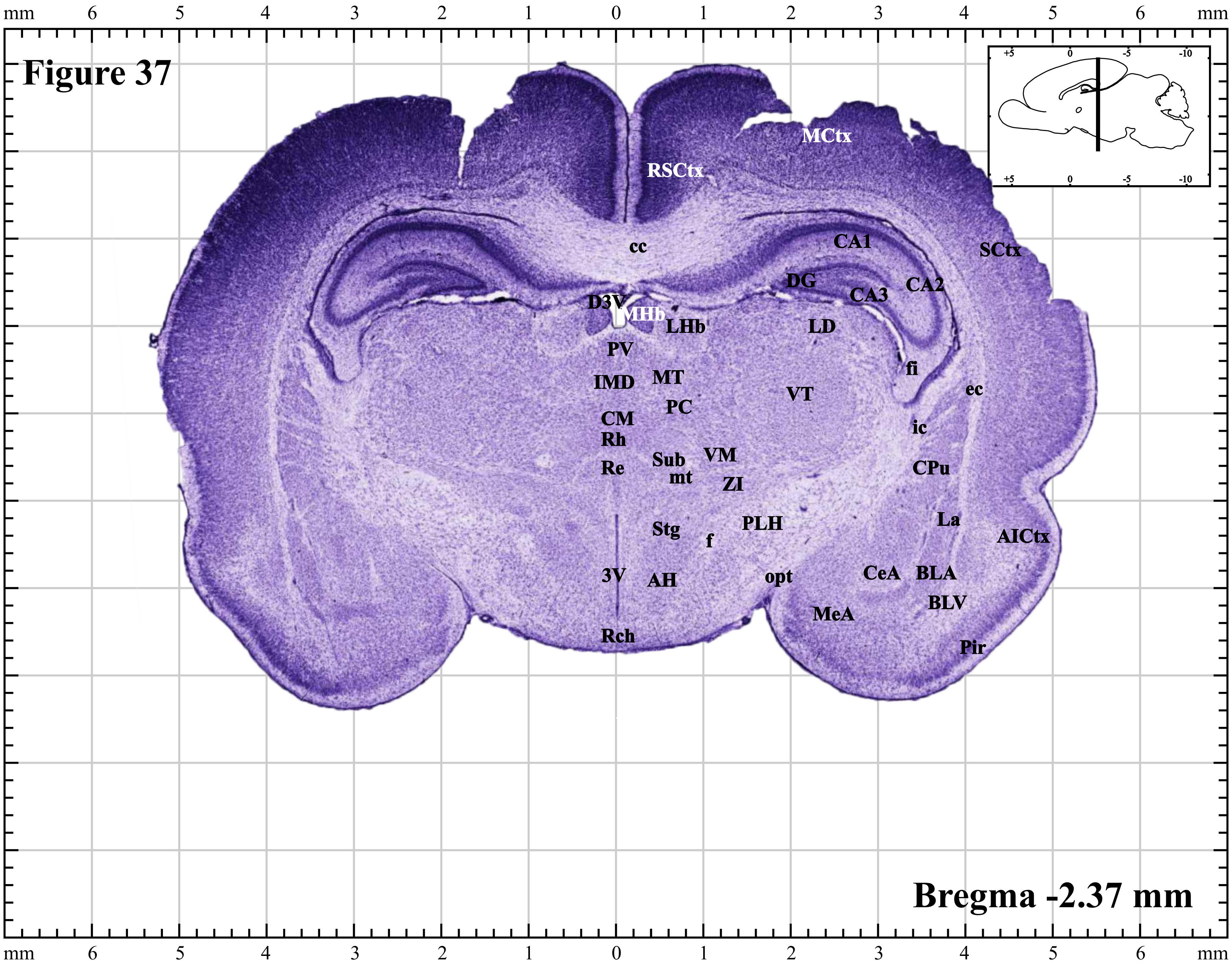

3V 3rd ventricle
AH anterior hypothalamic area
AICtx agranular insular cortex
Arc arcuate hypothalamic nucleus
BLA basolateral amygdaloid nucleus, anterior part
BLV basolateral amygdaloid nucleus, ventral part
cc corpus callosum
CPu caudate putamen
CA1 field CA1 of the hippocampus
CA2 field CA2 of the hippocampus
CA3 field CA3 of the hippocampus
CM central medial thalamic nucleus
CeA central amygdaloid nucleus
D3V dorsal 3rd ventricle
DG dentate gyrus
ec external capsule
f fornix
fi fimbria of the hippocampus
ic internal capsule
IMD intermediodorsal thalamic nucleus
LHb lateral habenular nucleus
LD laterodorsal thalamic nucleus
mt mammillothalamic tract
MT medial thalamus
MHb medial habenular nucleus
MCtx motor cortex
MeA medial amygdaloid nucleus
opt optic tract
Pir piriform cortex
PC paracentral thalamic nucleus
PLH peduncular part of lateral hypothalamus
Rh rhomboid thalamic nucleus
Re reuniens thalamic nucleus
RSCtx retrospenial cortex
RCh retrochiasmatic area
SCtx somatosensory cortex
Sub submedius thalamic nucleus
Stg stigmoid hypothalamic nucleus
VT ventral thalamus
VM ventromedial thalamic nucleus
ZI zona incerta

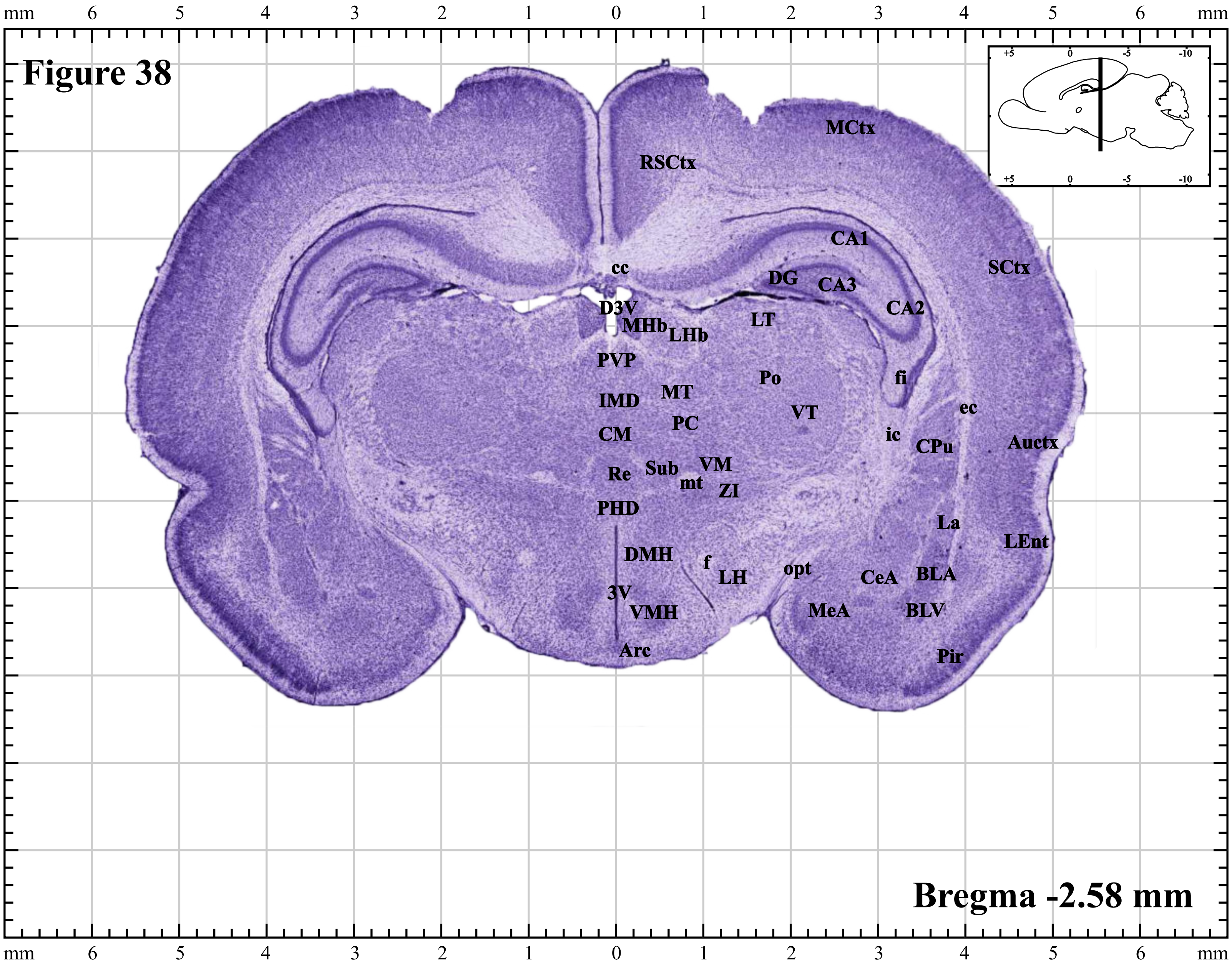

3V medial longitudinal fasciculus
Arc arcuate hypothalamic nucleus
Auctx auditory cortex
BLA basolateral amygdaloid nucleus, anterior part
BLV basolateral amygdaloid nucleus, ventral part
CA1 field CA1 of the hippocampus
CA2 field CA2 of the hippocampus
CA3 field CA3 of the hippocampus
cc corpus callosum
CeA central amygdaloid nucleus
CM central medial thalamic nucleus
CPu caudate putamen
D3V dorsal 3rd ventricle
DMH dorsomedial hypothalamic nucleus
DG dentate gyrus
ec external capsule
f fornix
fi fimbria of the hippocampus
ic internal capsule
IMD intermediodorsal thalamic nucleus
La lat amygdaloid nucleus
LEnt lateral entorhinal cortex
LHb lateral habenular nucleus
LH lateral hypothalamic area
LT lateral thalamus
MeA medial amygdaloid nucleus
mt mammillothalamic tract
MHb medial habenular nucleus
MT medial thalamus
opt optic tract
MCtx motor cortex
PC paracentral thalamic nucleus
Pir piriform cortex
PVP paraventricular thalamic nucleus, posterior part
Po posterior thalamic nuclear group
PHD posterior hypothalamic area, dorsal part
Re reuniens thalamic nucleus
RSCtx retrospenial cortex
SCtx somatosensory cortex
Sub submedius thalamic nucleus
VM ventromedial thalamic nucleus
VMH ventromedial hypothalamic nucleus
VT ventral thalamus
ZI zona incerta

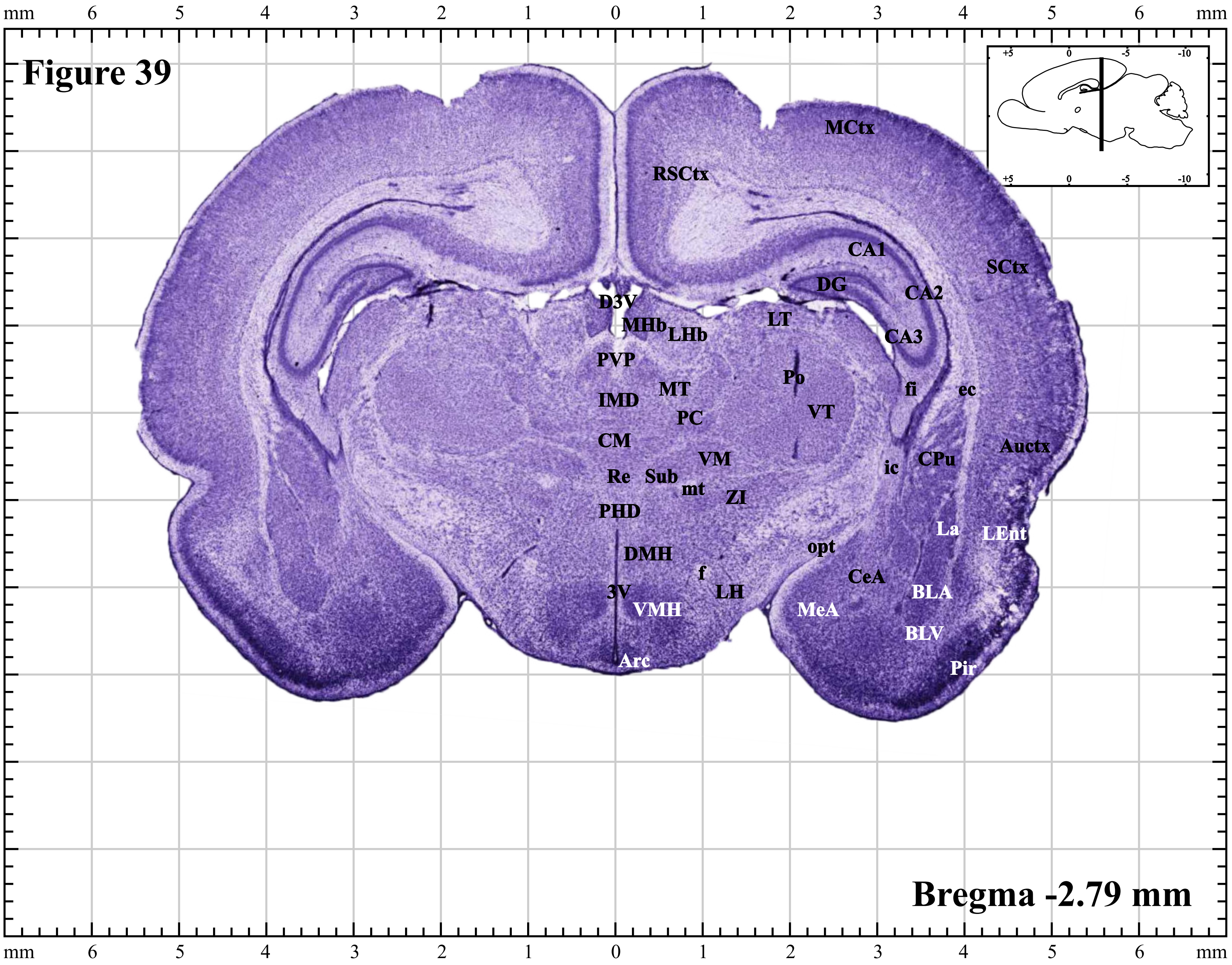

3V medial longitudinal fasciculus
Arc arcuate hypothalamic nucleus
Auctx auditory cortex
BLA basolateral amygdaloid nucleus, anterior part
BLV basolateral amygdaloid nucleus, ventral part
CA1 field CA1 of the hippocampus
CA2 field CA2 of the hippocampus
CA3 field CA3 of the hippocampus
CeA central amygdaloid nucleus
CM central medial thalamic nucleus
CPu caudate putamen
D3V dorsal 3rd ventricle
DMH dorsomedial hypothalamic nucleus
DG dentate gyrus
ec external capsule
f fornix
fi fimbria of the hippocampus
ic internal capsule
IMD intermediodorsal thalamic nucleus
La lat amygdaloid nucleus
LEnt lateral entorhinal cortex
LHb lateral habenular nucleus
LH lateral hypothalamic area
LT lateral thalamus
MeA medial amygdaloid nucleus
mt mammillothalamic tract
MHb medial habenular nucleus
MT medial thalamus
opt optic tract
MCtx motor cortex
PC paracentral thalamic nucleus
Pir piriform cortex
PVP paraventricular thalamic nucleus, posterior part
Po posterior thalamic nuclear group
PHD posterior hypothalamic area, dorsal part
Re reuniens thalamic nucleus
RSCtx retrospenial cortex
SCtx somatosensory cortex
Sub submedius thalamic nucleus
VM ventromedial thalamic nucleus
VMH ventromedial hypothalamic nucleus
VT ventral thalamus
ZI zona incerta

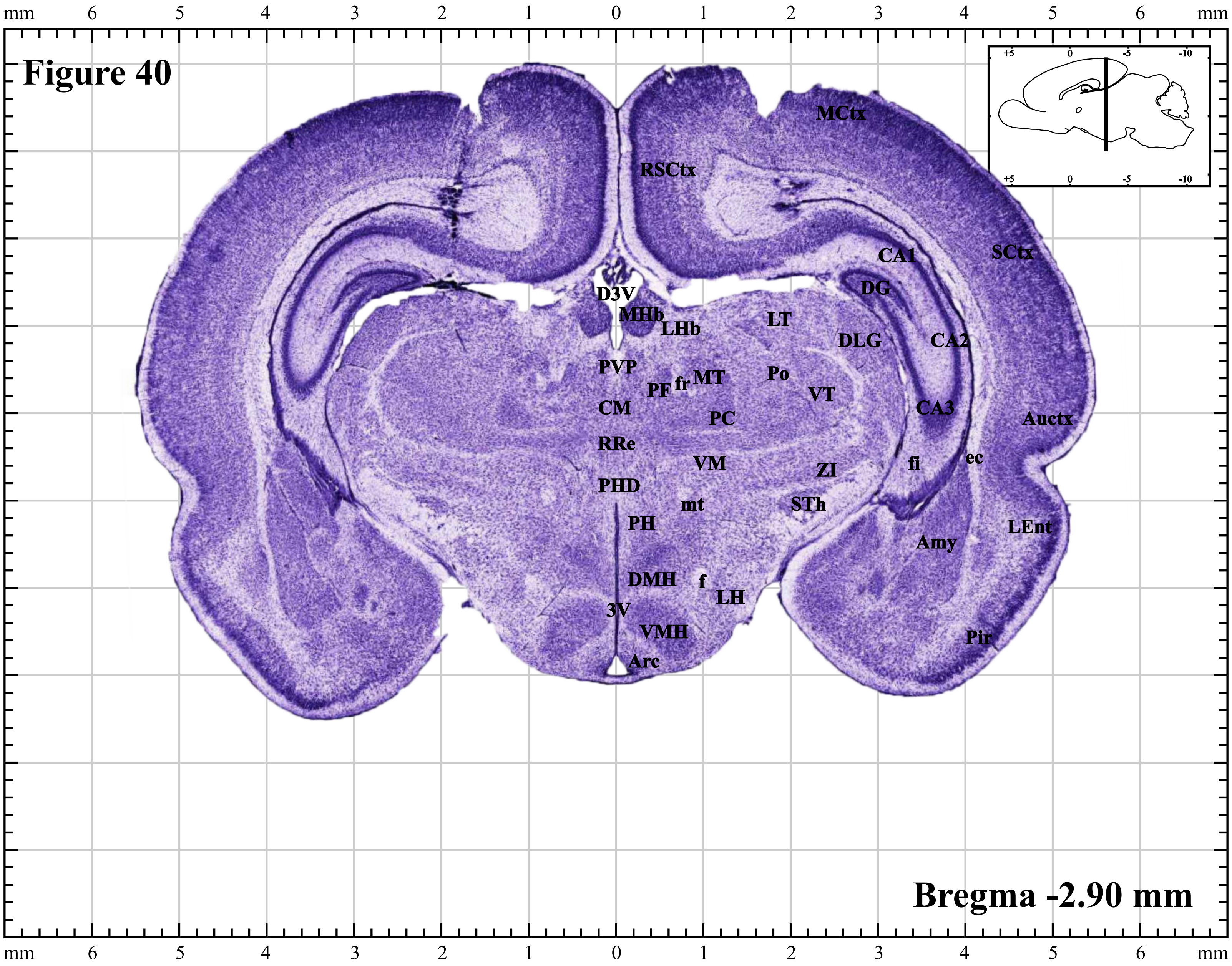

Figure 40

Bregma -2.90 mm

3V medial longitudinal fasciculus
Auctx auditory cortex
CA1 field CA1 of the hippocampus
CA2 field CA2 of the hippocampus
CA3 field CA3 of the hippocampus
cc corpus callosum
CeA central amygdaloid nucleus
CM central medial thalamic nucleus
D3V dorsal 3rd ventricle
DMH dorsomedial hypothalamic nucleus
DG dentate gyrus
DLG dorsal lateral geniculate nucleus
ec external capsule
f fornix
fi fimbria of the hippocampus
fr fasciculus retroflexus
IMD intermediodorsal thalamic nucleus
LEnt lateral entorhinal cortex
LHb lateral habenular nucleus
LH lateral hypothalamic area
LT lateral thalamus
mt mammillothalamic tract
MHb medial habenular nucleus
MT medial thalamus
PC paracentral thalamic nucleus
Pir piriform cortex
PHD posterior hypothalamic area, dorsal part
PF parafascicular thalamic nucleus
PVP paraventricular thalamic nucleus, posterior part
Po posterior thalamic nuclear group
PH posterior hypothalamic nucleus
PtActx parietal association cortex
RRe retrouniens area
RSCtx retrospenial cortex
SCtx somatosensory cortex
STh subthalamic nucleus
VM ventromedial thalamic nucleus
VT ventral thalamus
VMH ventromedial hypothalamic nucleus
ZI zona incerta

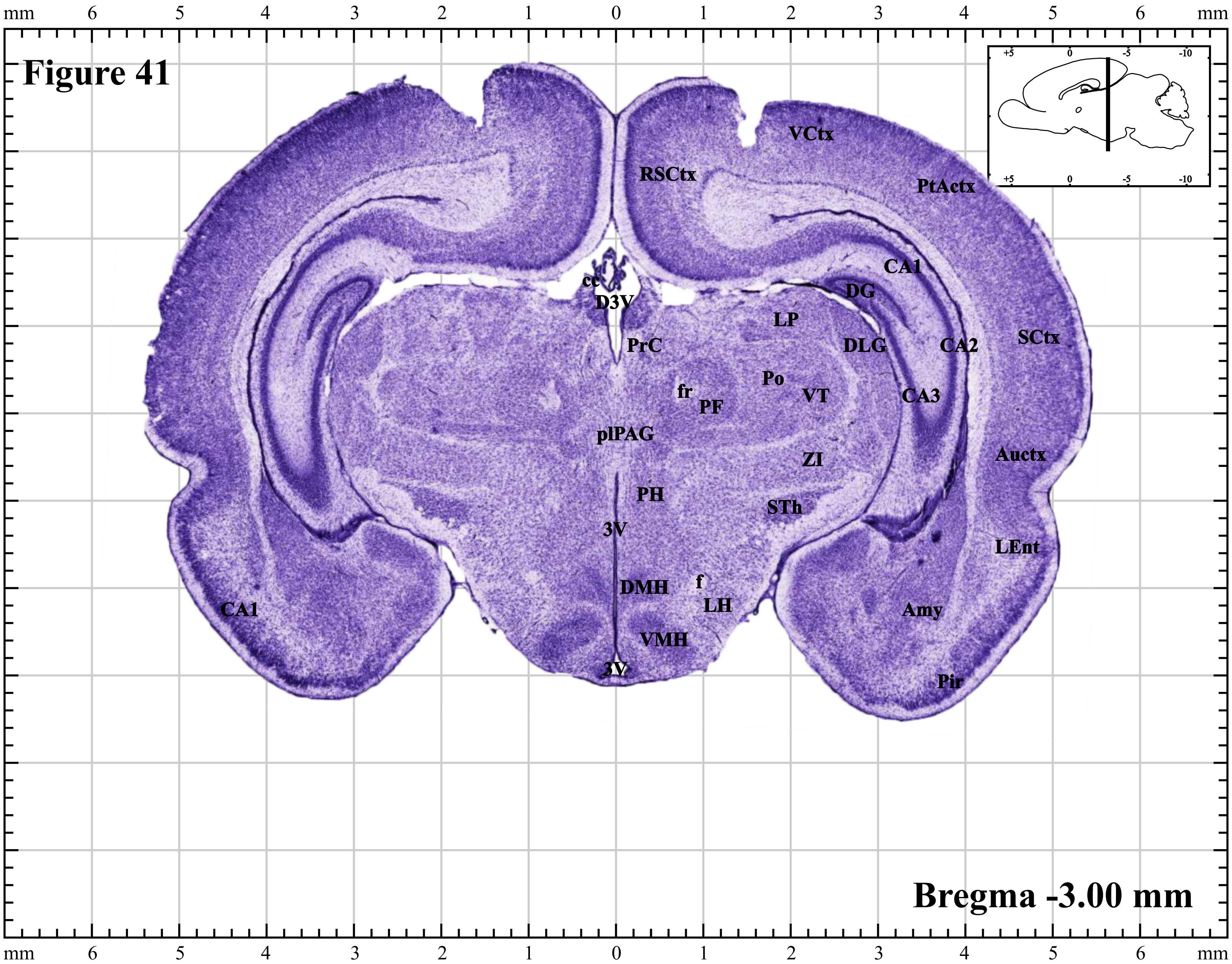

3V medial longitudinal fasciculus
Auctx auditory cortex
Amy amygdaloid nuclei
CA1 field CA1 of the hippocampus
CA2 field CA2 of the hippocampus
CA3 field CA3 of the hippocampus
cc corpus callosum
DMH dorsomedial hypothalamic nucleus
DLG dorsal lateral geniculate nucleus
D3V dorsal 3rd ventricle
DG dentate gyrus
f fornix
fr fasciculus retroflexus
ic internal capsule
LHb lateral habenular nucleus
LH lateral hypothalamic area
LP lateral posterior thalamic nucleus
LEnt lateral entorhinal cortex
mt mammillothalamic tract
MHb medial habenular nucleus
PrC precommissural nucleus
Pir piriform cortex
Po posterior thalamic nuclear group
plPAG pleomorphic part of periaqueductai gray
PMV premammillary nucleus, ventral part
PF parafascicular thalamic nucleus
PH posterior hypothalamic nucleus
PtActx parietal association cortex
RSCtx retrospenial cortex
SCtx somatosensory cortex
STh subthalamic nucleus
VCtx visual cortex
VT ventral thalamus
VMH ventromedial hypothalamic nucleus
ZI zona incerta

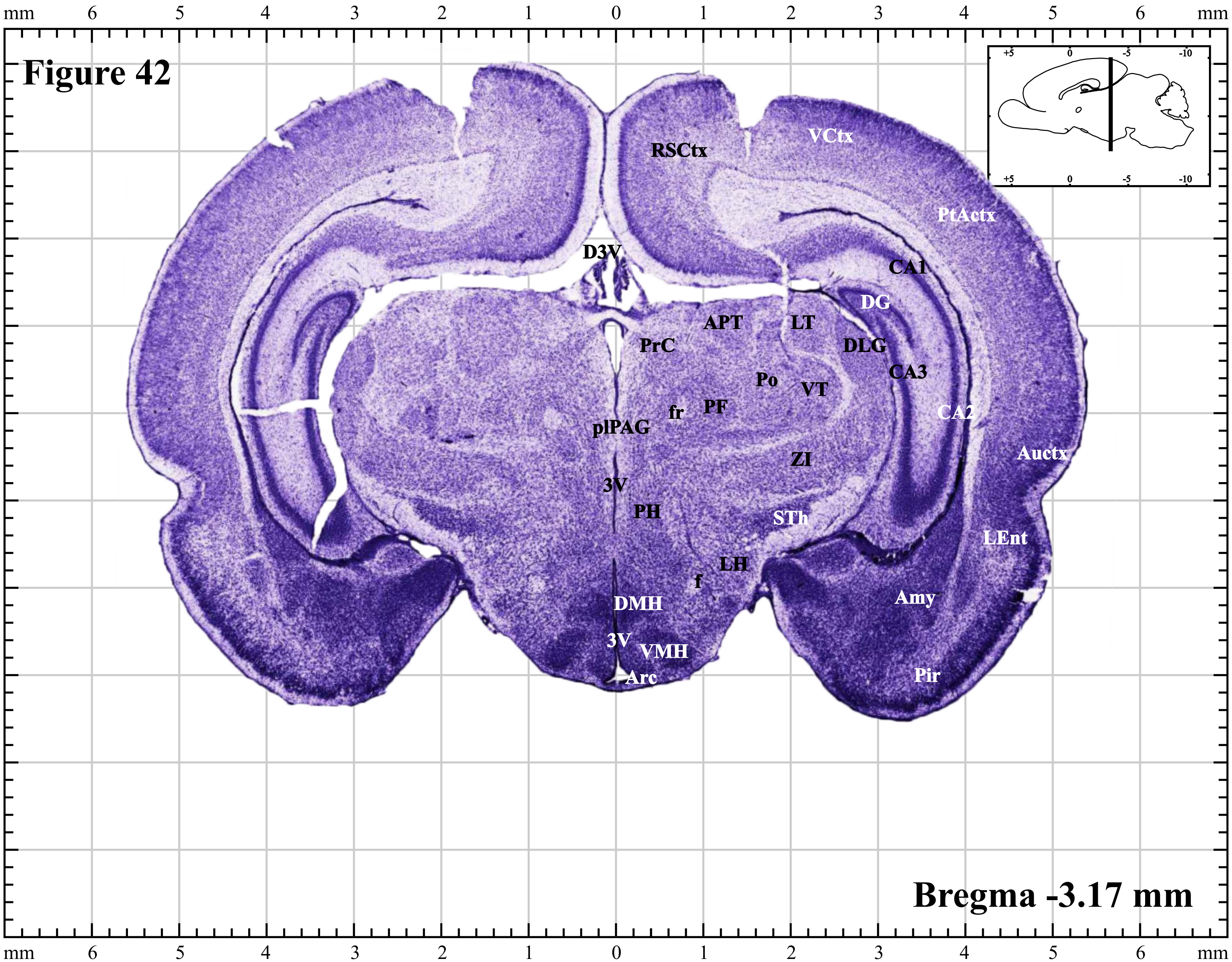

3V medial longitudinal fasciculus
Auctx auditory cortex
Arc arcuate hypothalamic nucleus
Amy amygdaloid nuclei
APT anterior pretectal nucleus
CA1 field CA1 of the hippocampus
CA2 field CA2 of the hippocampus
CA3 field CA3 of the hippocampus
DLG dorsal lateral geniculate nucleus
D3V dorsal 3rd ventricle
DG dentate gyrus
DMH dorsomedial hypothalamic nucleus
f fornix
fr fasciculus retroflexus
ic internal capsule
LH lateral hypothalamic area
LP lateral posterior thalamic nucleus
LEnt lateral entorhinal cortex
mt mammillothalamic tract
PrC precommissural nucleus
Pir piriform cortex
Po posterior thalamic nuclear group
plPAG pleomorphic part of periaqueductai gray
PF parafascicular thalamic nucleus
PH posterior hypothalamic nucleus
PtActx parietal association cortex
RSCtx retrospenial cortex
SCtx somatosensory cortex
STh subthalamic nucleus
VCtx visual cortex
VT ventral thalamus
VMH ventromedial hypothalamic nucleus
ZI zona incerta

Figure 43

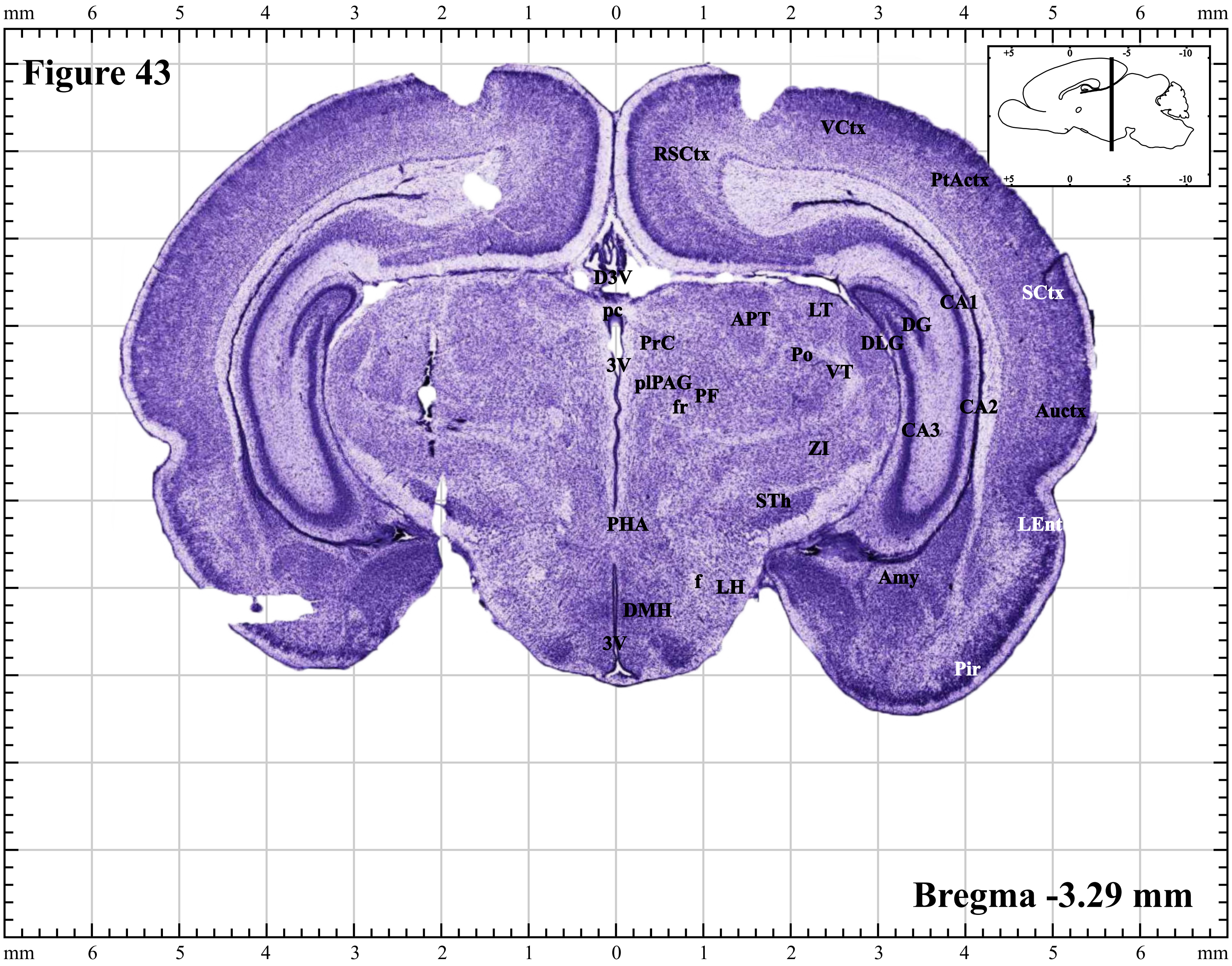

Bregma -3.29 mm

3V medial longitudinal fasciculus
Auctx auditory cortex
Amy amygdaloid nuclei
APT anterior pretectal nucleus
CA1 field CA1 of the hippocampus
CA2 field CA2 of the hippocampus
CA3 field CA3 of the hippocampus
D3V dorsal 3rd ventricle
DG dentate gyrus
DLG dorsal lateral geniculate nucleus
DMH dorsomedial hypothalamic nucleus
f fornix
fr fasciculus retroflexus
LH lateral hypothalamic area
LT lateral thalamus
LEnt lateral entorhinal cortex
pc posterior commissure
PF parafascicular thalamic nucleus
PrC precommissural nucleus
Pir piriform cortex
Po posterior thalamic nuclear group
plPAG pleomorphic part of periaqueductai gray
PHA posterior hypothalamic area
PtActx parietal association cortex
RSCtx retrospenial cortex
SCtx somatosensory cortex
STh subthalamic nucleus
VCtx visual cortex
VT ventral thalamus
VMH ventromedial hypothalamic nucleus
ZI zona incerta

Figure 44

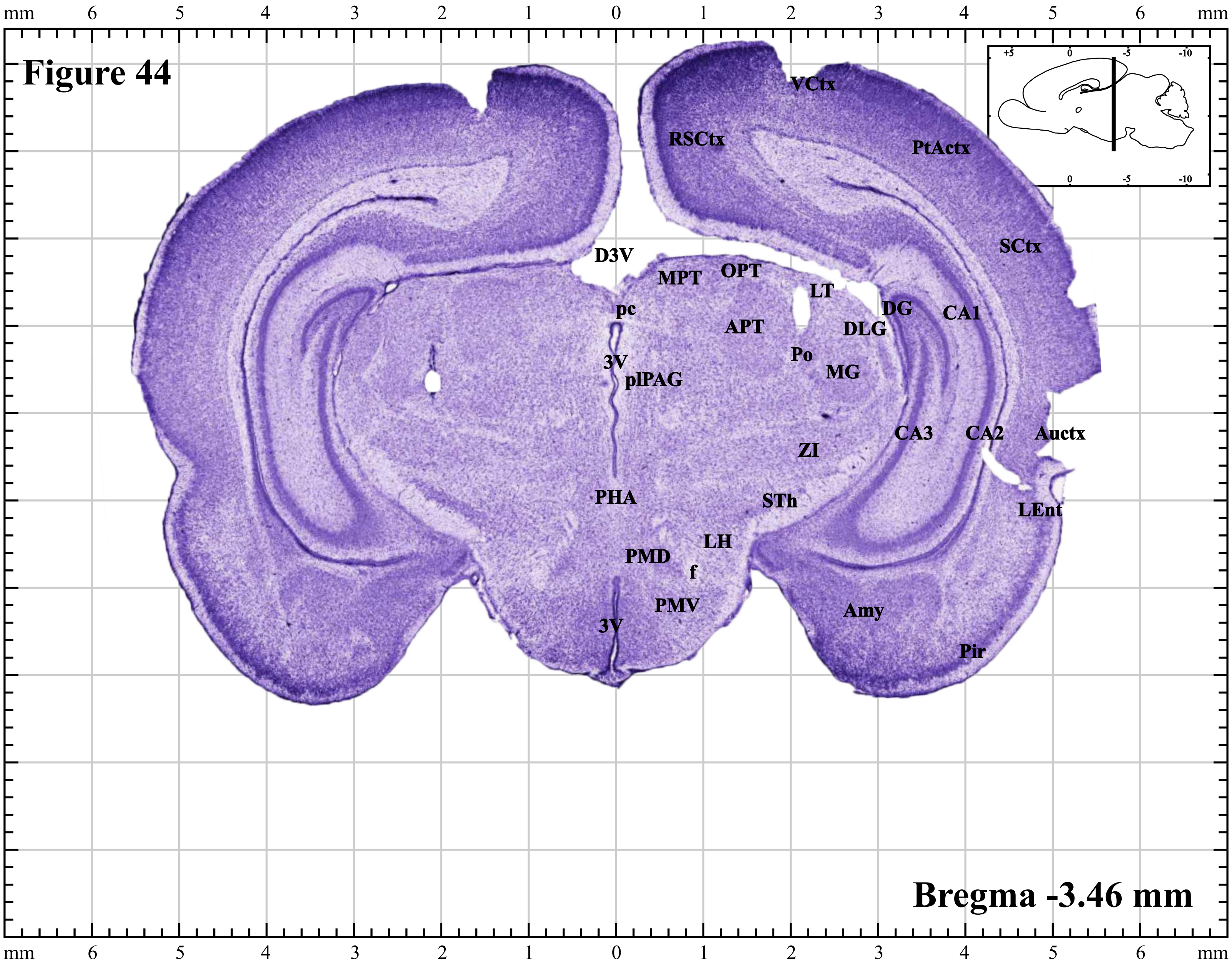

Bregma -3.46 mm

3V medial longitudinal fasciculus
Auctx auditory cortex
Amy amygdaloid nuclei
APT anterior pretectal nucleus
CA1 field CA1 of the hippocampus
CA2 field CA2 of the hippocampus
CA3 field CA3 of the hippocampus
D3V dorsal 3rd ventricle
DG dentate gyrus
DLG dorsal lateral geniculate nucleus
f fornix
LT lateral thalamus
LEnt lateral entorhinal cortex
LH lateral hypothalamic area
MG medial geniculate nucleus
MPT medial pretectal nucleus
OPT olivary pretectal nucleus
Pir piriform cortex
PMV premammillary nucleus, ventral part
PMD premammillary nucleus, dorsal part
Po posterior thalamic nuclear group
pc posterior commissure
PHA posterior hypothalamic area
plPAG pleomorphic part of periaqueductai gray
PtActx parietal association cortex
RSCtx retrospenial cortex
SCtx somatosensory cortex
STh subthalamic nucleus
VCtx visual cortex
ZI zona incerta

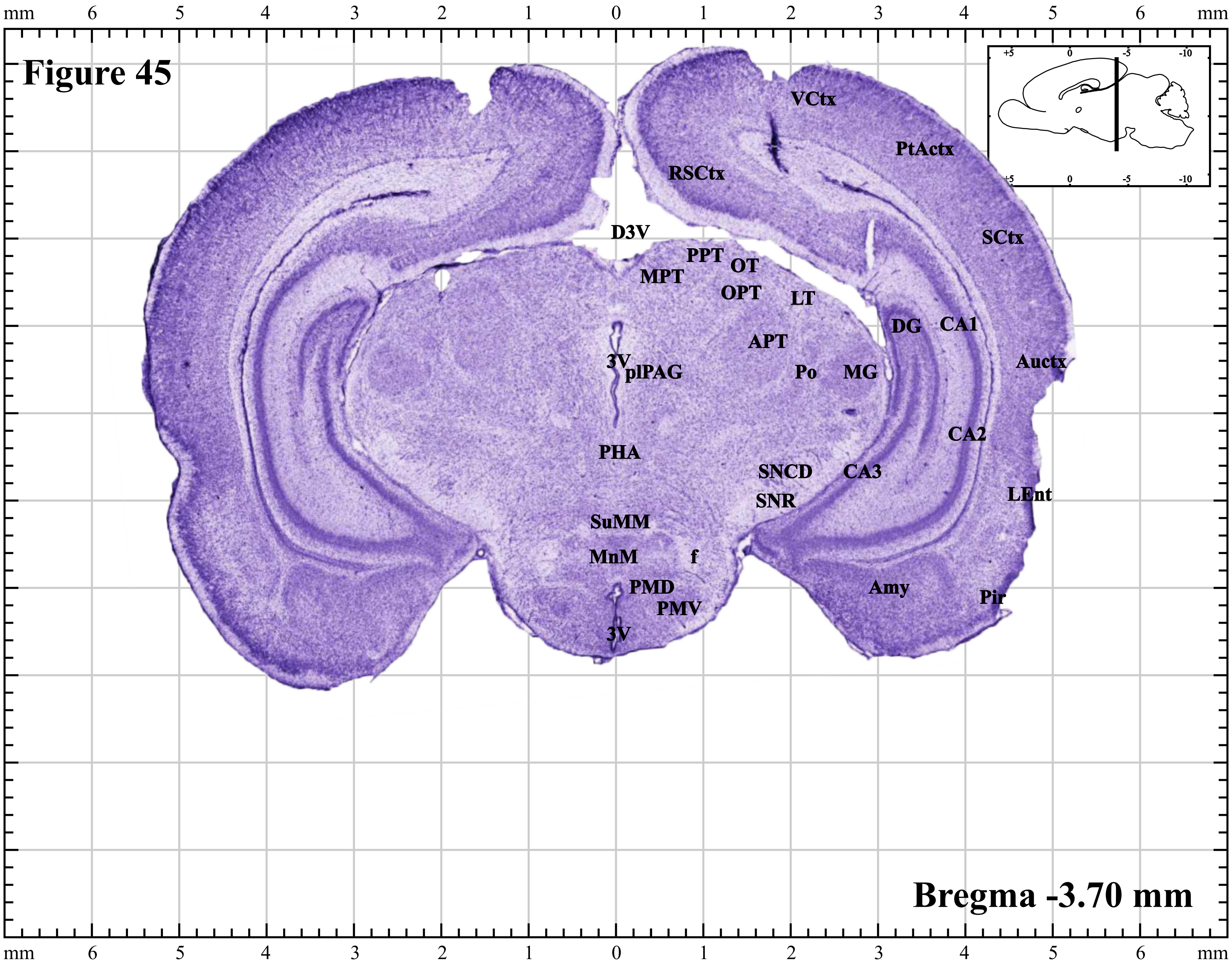

3V medial longitudinal fasciculus
Auctx auditory cortex
Amy amygdaloid nuclei
APT anterior pretectal nucleus
CA1 field CA1 of the hippocampus
CA2 field CA2 of the hippocampus
CA3 field CA3 of the hippocampus
D3V dorsal 3rd ventricle
DG dentate gyrus
f fornix
LT lateral thalamus
LEnt lateral entorhinal cortex
LH lateral hypothalamic area
MG medial geniculate nucleus
MPT medial pretectal nucleus
MnM medial mammillary nucleus, median part
OPT olivary pretectal nucleus
OT nucleus of the optic
PPT posterior pretectal nucleus
Pir piriform cortex
PMV premammillary nucleus, ventral part
PMD premammillary nucleus, dorsal part
Po posterior thalamic nuclear group
PHA posterior hypothalamic area
plPAG pleomorphic part of periaqueductai gray
PtActx parietal association cortex
RSCtx retrospenial cortex
SCtx somatosensory cortex
SNR substantia nigra, reticular part
SNCD substantia nigra, compact part, dorsal tier
SuMM supramammillary nucleus, medial part
VCtx visual cortex

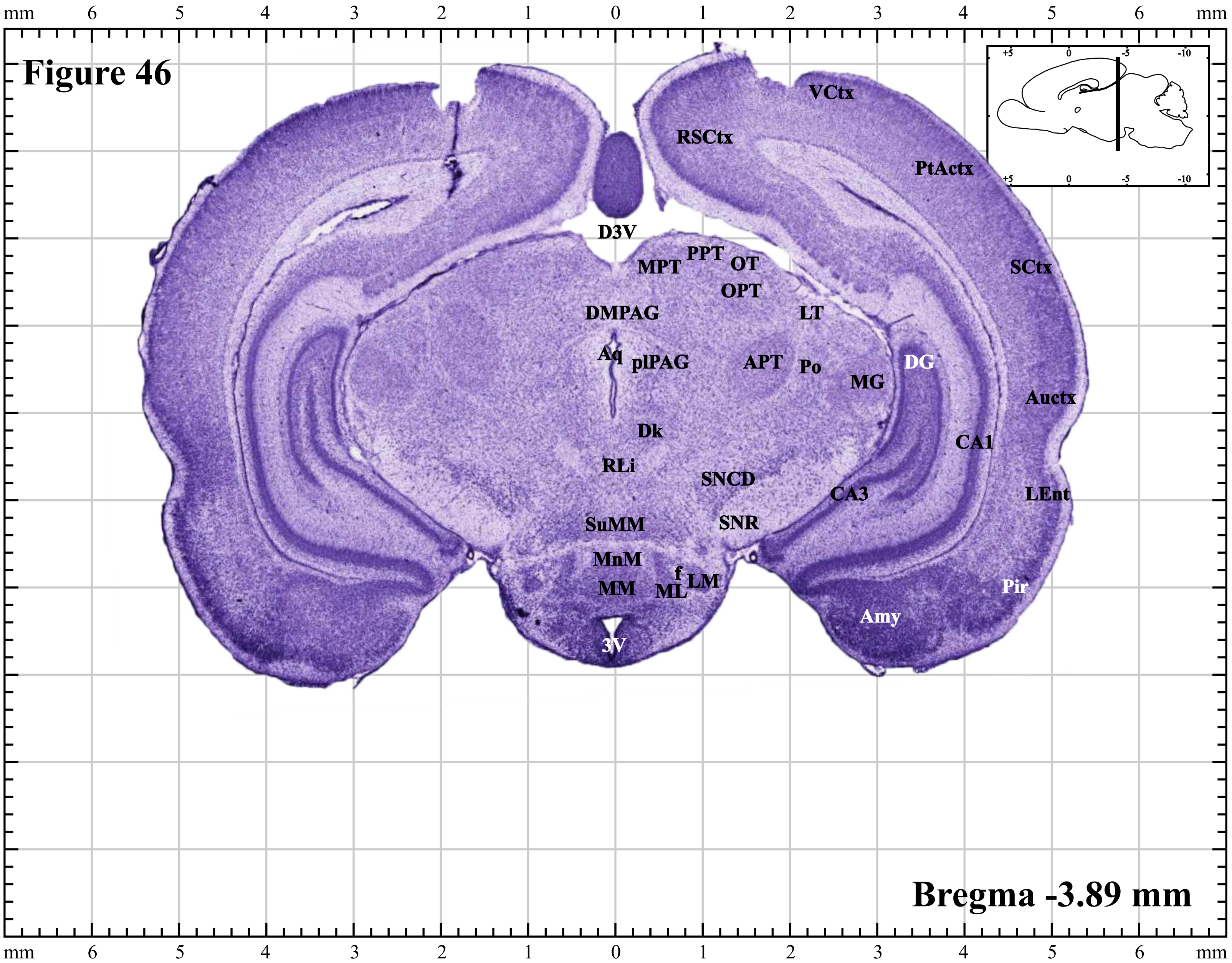

- **Aq** aqueduct
- **Auctx** auditory cortex
- **Amy** amygdaloid nuclei
- **APT** anterior pretectal nucleus
- **CA1** field CA1 of the hippocampus
- **CA3** field CA3 of the hippocampus
- **D3V** dorsal 3rd ventricle
- **DG** dentate gyrus
- **Dk** nucleus of Darkschewitsch
- **DMPAG** dorsomedial periaqueductal gray
- **DLG** dorsal lateral geniculate nucleus
- **f** fornix
- **LT** lateral thalamus
- **LEnt** lateral entorhinal cortex
- **LH** lateral hypothalamic area
- **LM** lateral mammillary nucleus
- **MG** medial geniculate nucleus
- **MPT** medial pretectal nucleus
- **MnM** medial mammillary nucleus, median part
- **ML** medial mammillary nucleus, lateral part
- **MM** medial mammillary nucleus, medial part
- **OPT** olivary pretectal nucleus
- **OT** nucleus of the optic
- **PPT** posterior pretectal nucleus
- **Pir** piriform cortex
- **Po** posterior thalamic nuclear group
- **PHA** posterior hypothalamic area
- **plPAG** pleomorphic part of periaqueductai gray
- **PtActx** parietal association cortex
- **RSCtx** retrospenial cortex
- **RLi** rostral linear nucleus of the raphe
- **SCtx** somatosensory cortex
- **SNR** substantia nigra, reticular part
- **SNCD** substantia nigra, compact part, dorsal tier
- **SuMM** supramammillary nucleus, medial part
- **VCtx** visual cortex

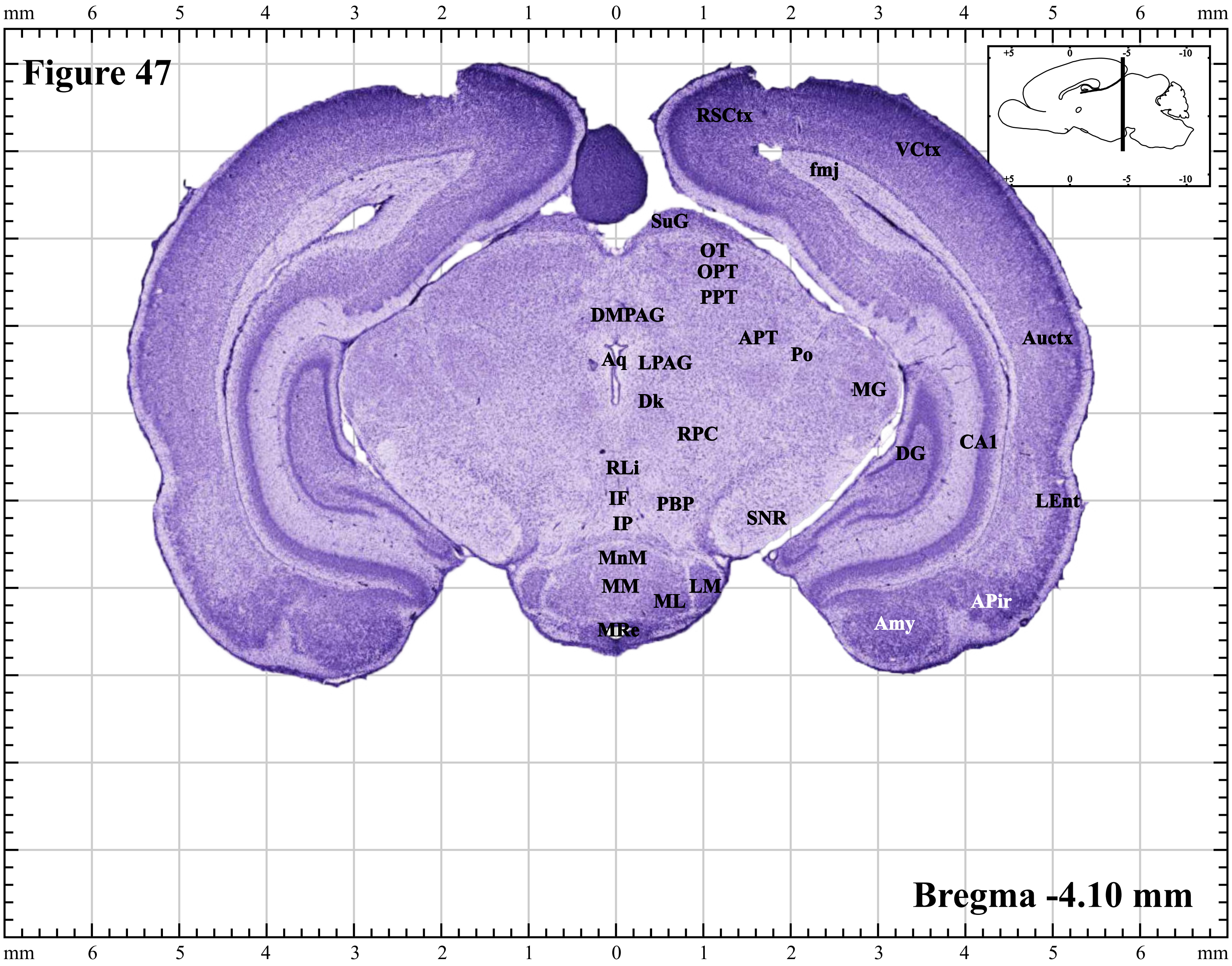

Aq aqueduct
Auctx auditory cortex
APir amygdalopiriform transition area
APT anterior pretectal nucleus
Amy amygdaloid nuclei
CA1 field CA1 of the hippocampus
DMPAG dorsomedial periaqueductal gray
DG dentate gyrus
Dk nucleus of Darkschewitsch
fmj forceps major of the corpus callosum
IC inferior colliculus
IF interfascicular nucleus
IP interpeduncular nucleus
LEnt lateral entorhinal cortex
LPAG lateral periaqueductal gray
LM lateral mammillary nucleus
MG medial geniculate nucleus
ML medial mammillary nucleus, lateral part
MnM medial mammillary nucleus, median part
MM medial mammillary nucleus, medial part
MRe mammillary recess of the 3rd ventricle
OT nucleus of the optic
OPT olivary pretectal nucleus
Po posterior thalamic nuclear group
PBP parabrachial pigmented nucleus of the VTA
Pn pontine nuclei
PPT posterior pretectal nucleus
RPC red nucleus, parvicellular part
RSCtx retrospenial cortex
RLi rostral linear nucleus of the raphe
RPC red nucleus, parvicellular part
SuG superficial gray layer of the superior colliculus
SNR substantia nigra, reticular part
VCtx visual cortex

Figure 48

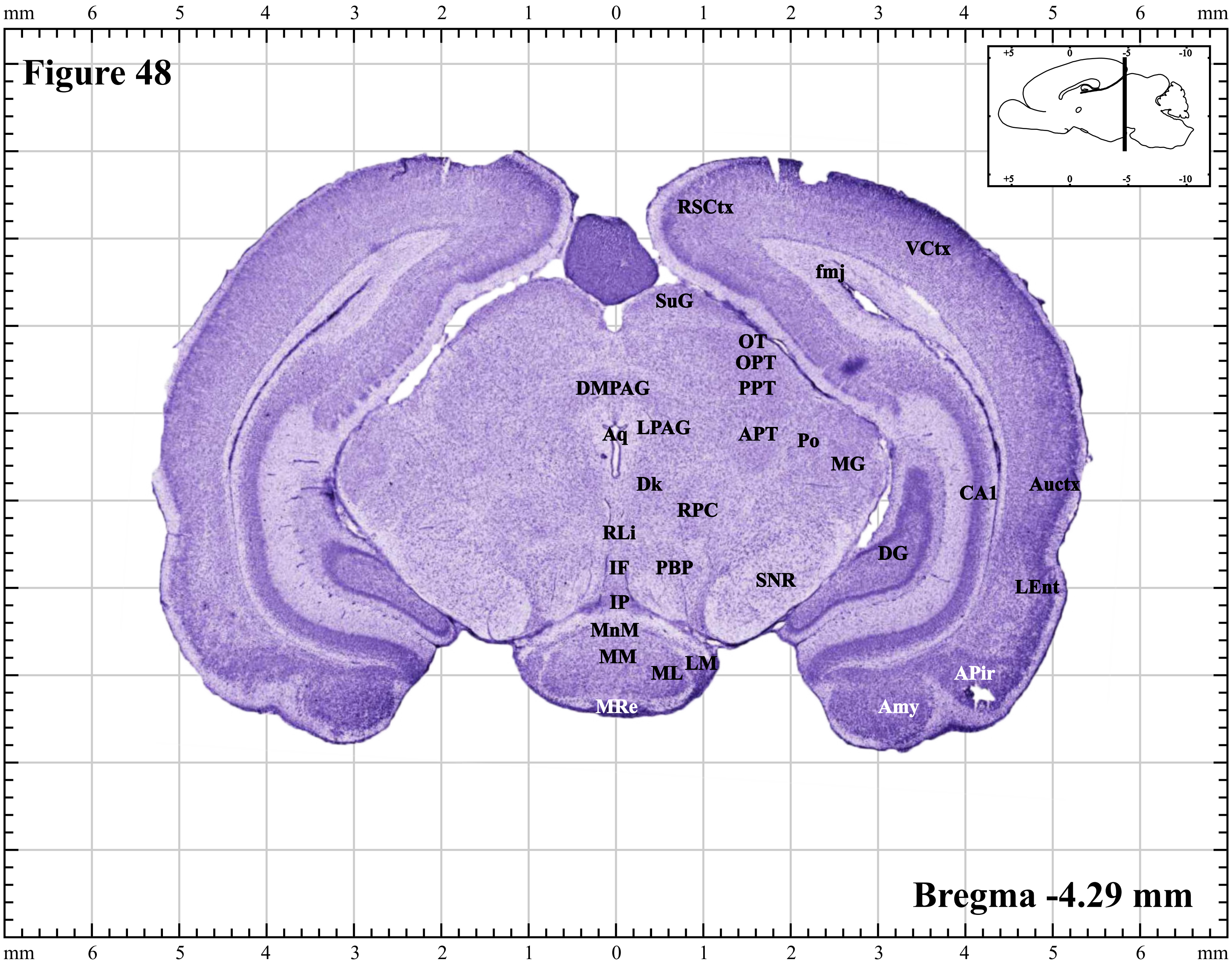

Bregma -4.29 mm

Aq aqueduct
Auctx auditory cortex
APT anterior pretectal nucleus
APir amygdalopiriform transition area
Amy amygdaloid nuclei
CA1 field CA1 of the hippocampus
DMPAG dorsomedial periaqueductal gray
DG dentate gyrus
Dk nucleus of Darkschewitsch
fmj forceps major of the corpus callosum
IC inferior colliculus
IF interfascicular nucleus
IP interpeduncular nucleus
LEnt lateral entorhinal cortex
LPAG lateral periaqueductal gray
LM lateral mammillary nucleus
MG medial geniculate nucleus
ML medial mammillary nucleus, lateral part
MnM medial mammillary nucleus, median part
MM medial mammillary nucleus, medial part
MRe mammillary recess of the 3rd ventricle
OT nucleus of the optic
OPT olivary pretectal nucleus
Po posterior thalamic nuclear group
PBP parabrachial pigmented nucleus of the VTA
Pn pontine nuclei
PPT posterior pretectal nucleus
RPC red nucleus, parvicellular part
RSCtx retrospenial cortex
RLi rostral linear nucleus of the raphe
RPC red nucleus, parvicellular part
SuG superficial gray layer of the superior colliculus
SNR substantia nigra, reticular part
VCtx visual cortex

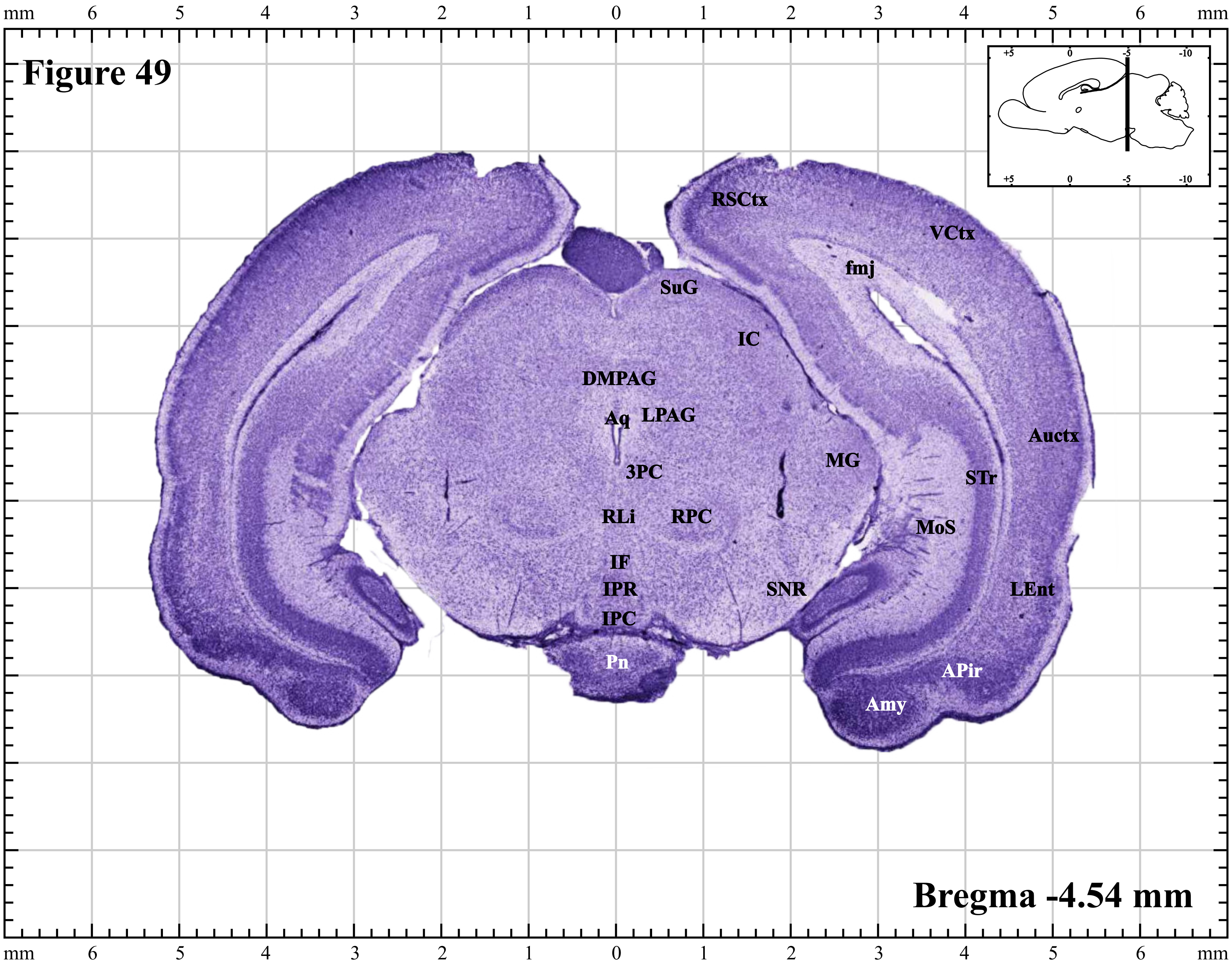

3PC oculomotor nucleus, parvicellular part
APir amygdalopiriform transition area
Amy amygdaloid nuclei
Aq aqueduct
Auctx auditory cortex
APT anterior pretectal nucleus
CA1 field CA1 of the hippocampus
DMPAG dorsomedial periaqueductal gray
DG dentate gyrus
fmj forceps major of the corpus callosum
IC inferior colliculus
IF interfascicular nucleus
IPC interpeduncular nucleus, caudal subnucleus
IPR interpeduncular nucleus, rostral subnucleus
LEnt lateral entorhinal cortex
LPAG lateral periaqueductal gray
MG medial geniculate nucleus
MoS molecular layer of the subiculum
Pn pontine nuclei
RSCtx retrospenial cortex
RLi rostral linear nucleus of the raphe
RPC red nucleus, parvicellular part
STr subiculum, transition area
SNR substantia nigra, reticular part
SuG superficial gray layer of the superior colliculus
VCtx visual cortex

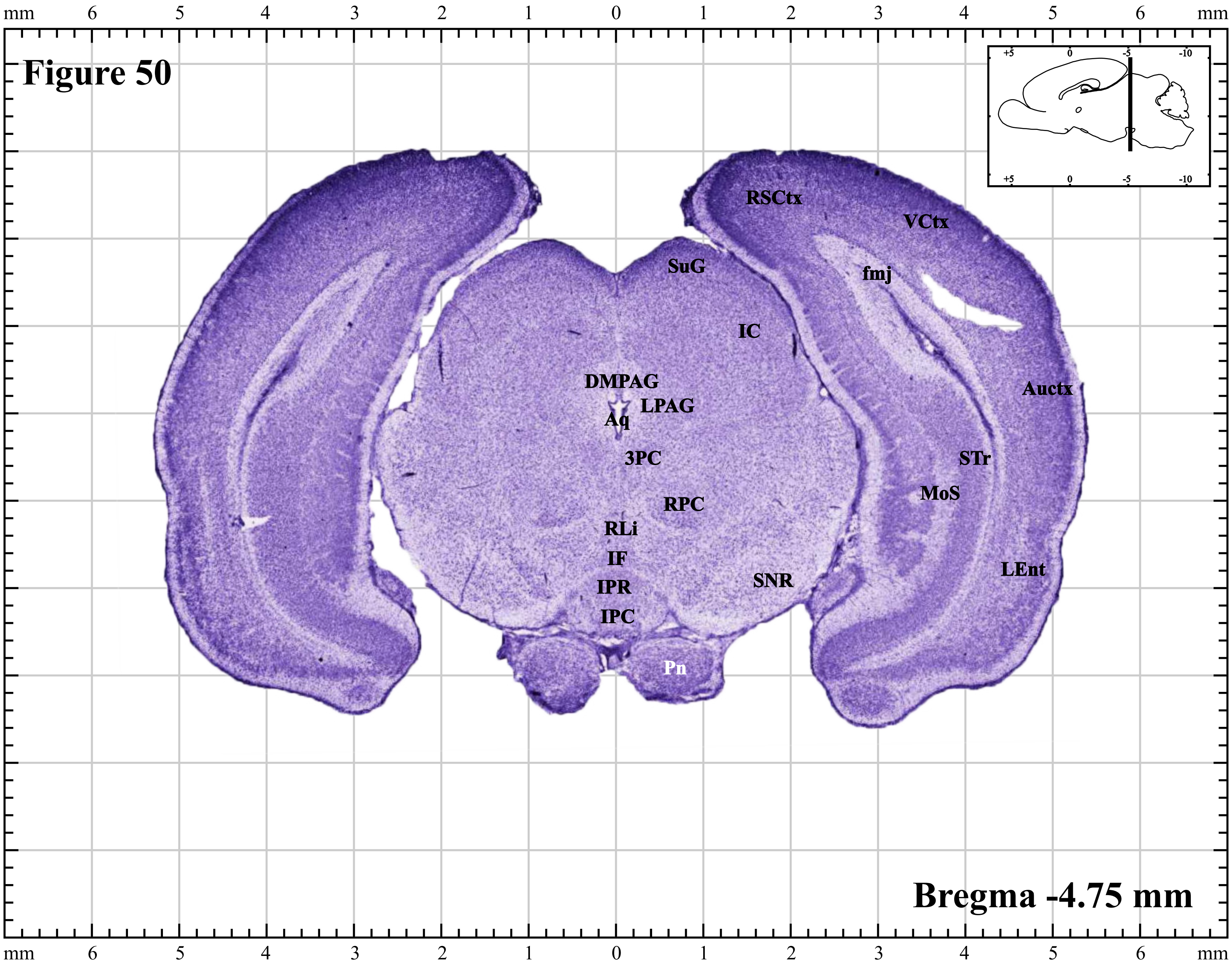

3PC oculomotor nucleus, parvicellular part
Aq aqueduct
Auctx auditory cortex
APT anterior pretectal nucleus
CA1 field CA1 of the hippocampus
DMPAG dorsomedial periaqueductal gray
DG dentate gyrus
fmj forceps major of the corpus callosum
IC inferior colliculus
IF interfascicular nucleus
IPC interpeduncular nucleus, caudal subnucleus
IPR interpeduncular nucleus, rostral subnucleus
LEnt lateral entorhinal cortex
LPAG lateral periaqueductal gray
MoS molecular layer of the subiculum
Pn pontine nuclei
RSCtx retrospenial cortex
RLi rostral linear nucleus of the raphe
RPC red nucleus, parvicellular part
STr subiculum, transition area
SNR substantia nigra, reticular part
SuG superficial gray layer of the superior colliculus
VCtx visual cortex

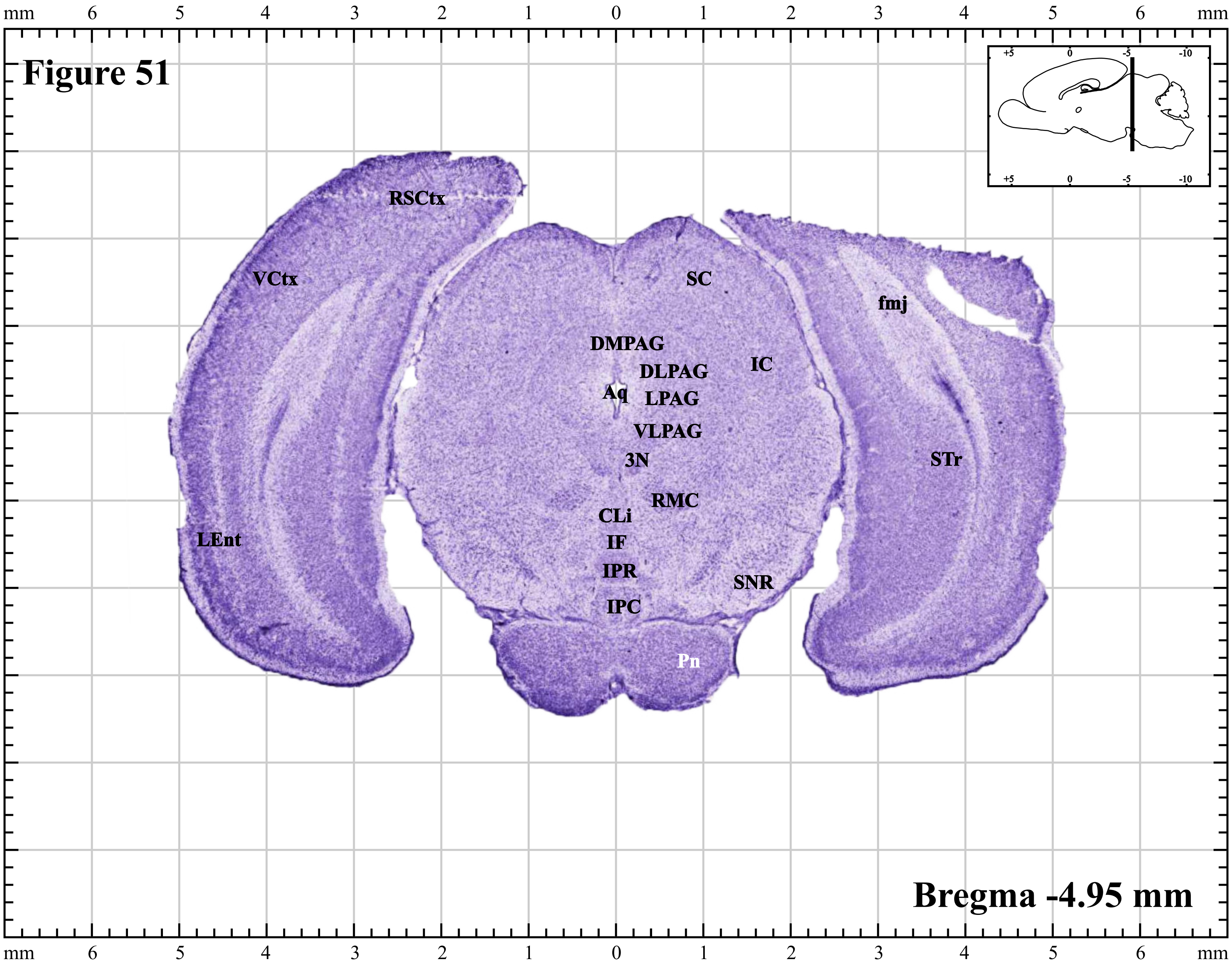

3N oculomotor nucleus
Aq aqueduct
CLi caudal linear nucleus of the raphe
DMPAG dorsomedial periaqueductal gray
fmj forceps major of the corpus callosum
IC inferior colliculus
DLPAG dorsolateral periaqueductal gray
IF interfascicular nucleus
IPC interpeduncular nucleus, caudal subnucleus
IPR interpeduncular nucleus, rostral subnucleus
LEnt lateral entorhinal cortex
LPAG lateral periaqueductal gray
Pn pontine nuclei
RSCtx retrospenial cortex
RMC red nucleus, magnocellular part
SC superior colliculus
SuG superficial gray layer of the superior colliculus
SNR substantia nigra, reticular part
STr subiculum, transition area
VCtx visual cortex
VLPAG ventrolateral periaqueductal gray

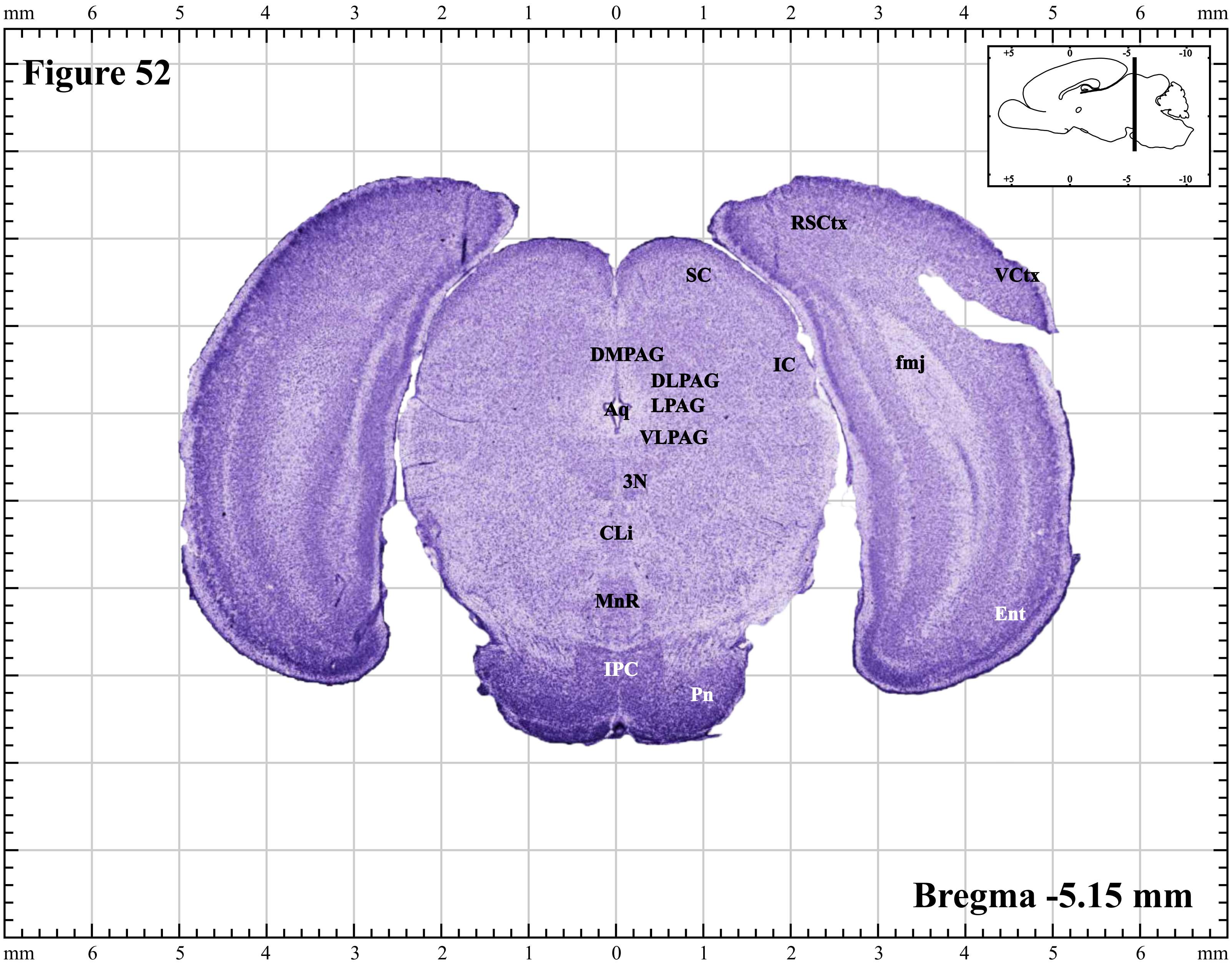

Figure 52

Bregma -5.15 mm

3N oculomotor nucleus
Aq aqueduct
CLi caudal linear nucleus of the raphe
DMPAG dorsomedial periaqueductal gray
DLPAG dorsolateral periaqueductal gray
Ent entorhinal cortex
fmj forceps major of the corpus callosum
IC inferior colliculus
IPC interpeduncular nucleus, caudal subnucleus
LPAG lateral periaqueductal gray
MnR median raphe nucleus
Pn pontine nuclei
RSCtx retrospenial cortex
SC superior colliculus
VCtx visual cortex
VLPAG ventrolateral periaqueductal gray

Figure 53

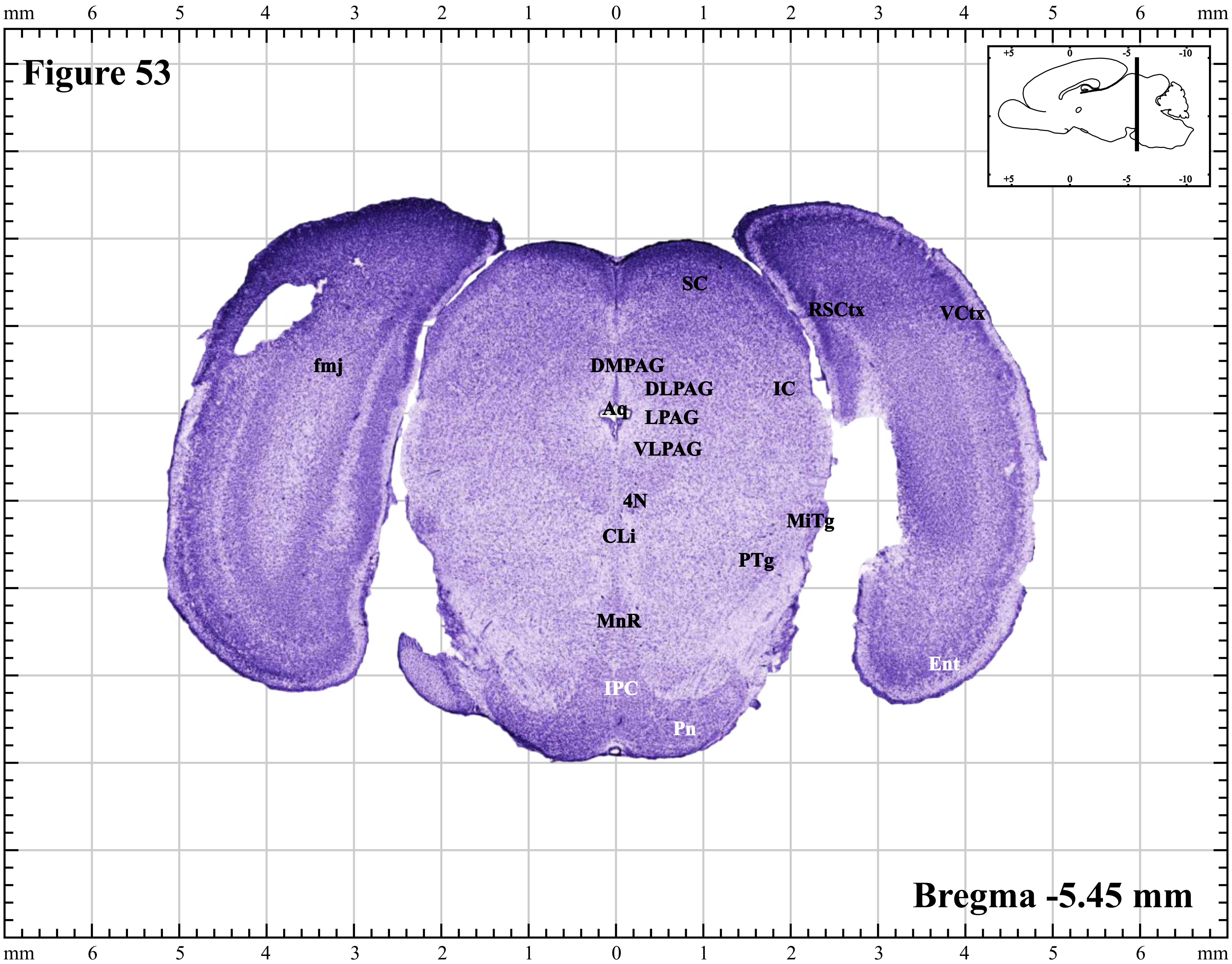

Bregma -5.45 mm

4N trochlear nucleus
Aq aqueduct
CLi caudal linear nucleus of the raphe
DMPAG dorsomedial periaqueductal gray
DLPAG dorsolateral periaqueductal gray
Ent entorhinal cortex
fmj forceps major of the corpus callosum
IC inferior colliculus
IPC interpeduncular nucleus, caudal subnucleus
LPAG lateral periaqueductal gray
MiTg microcellular tegmental nucleus
MnR median raphe nucleus
Pn pontine nuclei
PTg pedunculopontine tegmental nucleus
RSCtx retrospenial cortex
SC superior colliculus
VCtx visual cortex
VLPAG ventrolateral periaqueductal gray

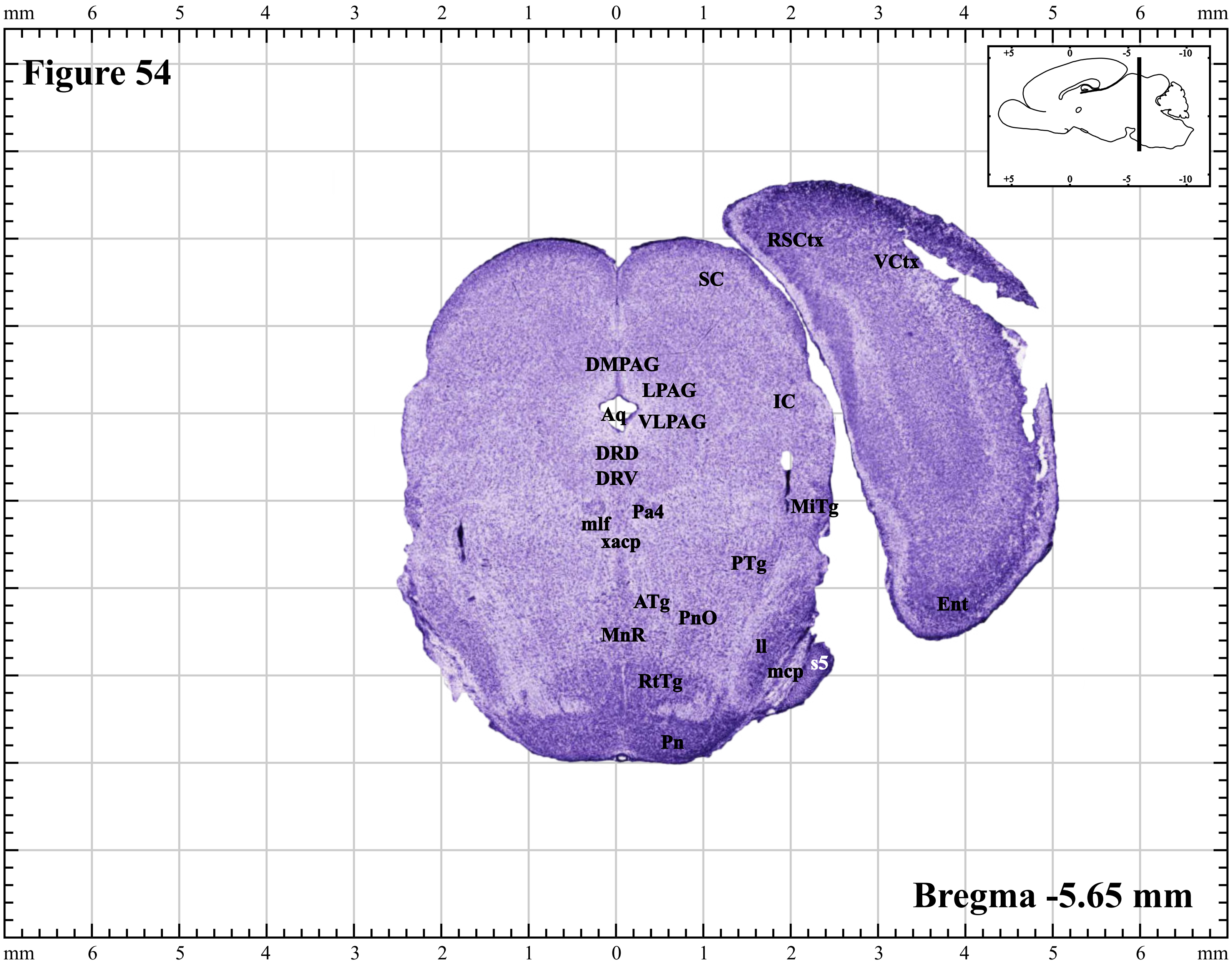

Figure 54

Bregma -5.65 mm

- **ATg** anterior tegmental nucleus
- **Aq** aqueduct
- **DMPAG** dorsomedial periaqueductal gray
- **DRD** dorsomedial hypothalamic nucleus, dorsal part
- **DRV** dorsomedial hypothalamic nucleus, ventral part
- **Ent** entorhinal cortex
- **IC** inferior colliculus
- **ll** lateral lemniscus
- **LPAG** lateral periaqueductal gray
- **MnR** median raphe nucleus
- **mlf** medial longitudinal fasciculus
- **mcp** middle cerebellar peduncle
- **MiTg** microcellular tegmental nucleus
- **PnO** pontine reticular nucleus, oral part
- **Pn** pontine nuclei
- **PTg** pedunculopontine tegmental nucleus
- **Pa4** paratrochlear nucleus
- **RSCtx** retrospenial cortex
- **RtTg** reticulotegmental nucleus of the pons
- **s5** sensory root of the trigeminal nerve
- **VCtx** visual cortex
- **SC** superior colliculus
- **VLPAG** ventrolateral periaqueductal gray
- **xscp** decussation of the superior cerebellar peduncle

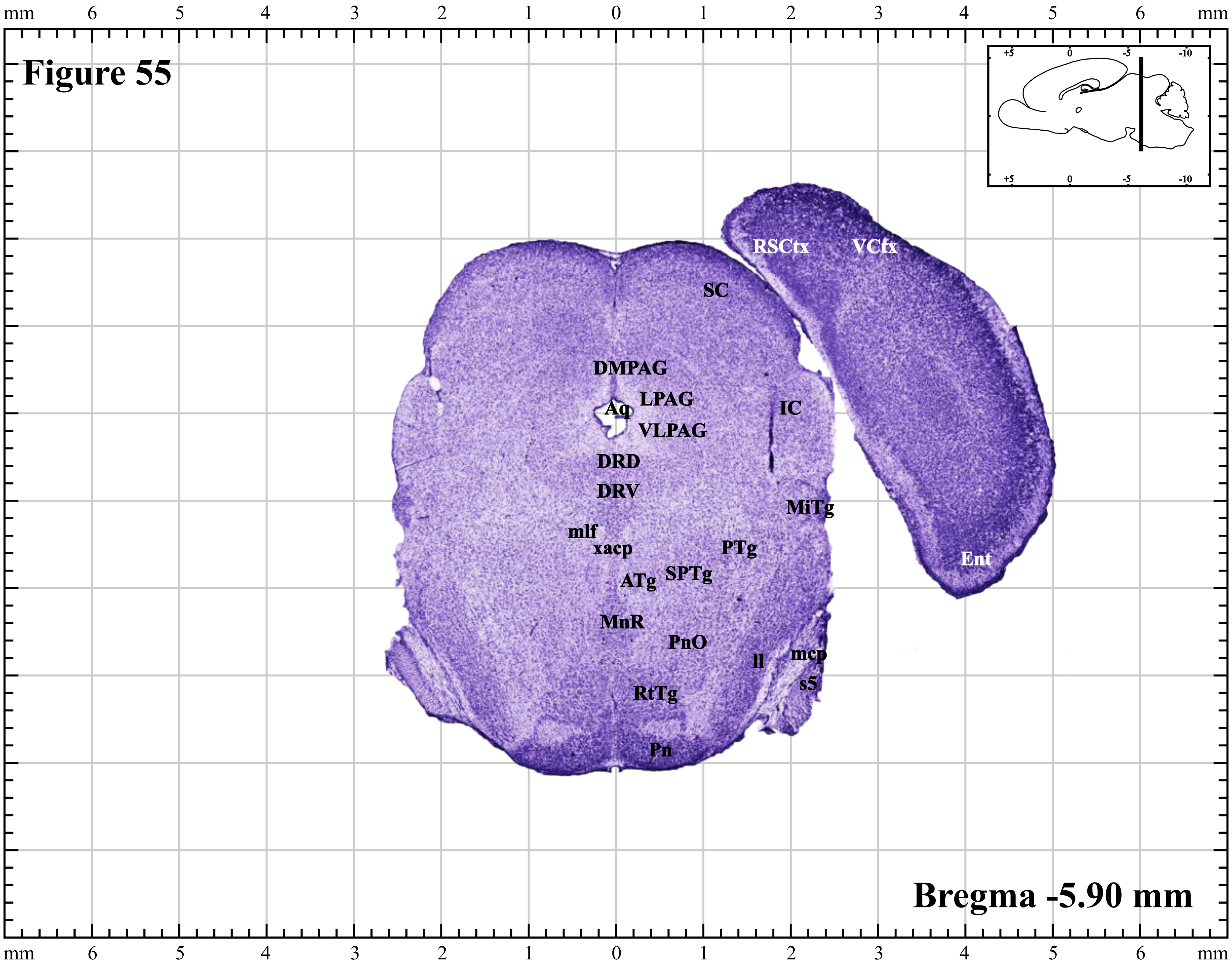

Aq aqueduct
ATg anterior tegmental nucleus
DMPAG dorsomedial periaqueductal gray
DRD dorsomedial hypothalamic nucleus, dorsal part
DRV dorsomedial hypothalamic nucleus, ventral part
Ent entorhinal cortex
IC inferior colliculus
ll lateral lemniscus
LPAG lateral periaqueductal gray
MnR median raphe nucleus
mlf medial longitudinal fasciculus
mcp middle cerebellar peduncle
MiTg microcellular tegmental nucleus
PnO pontine reticular nucleus, oral part
Pn pontine nuclei
PTg pedunculopontine tegmental nucleus
RSCtx retrospenial cortex
RtTg reticulotegmental nucleus of the pons
s5 sensory root of the trigeminal nerve
SC superior colliculus
SPTg subpeduncular tegmental nucleus
VCtx visual cortex
VLPAG ventrolateral periaqueductal gray

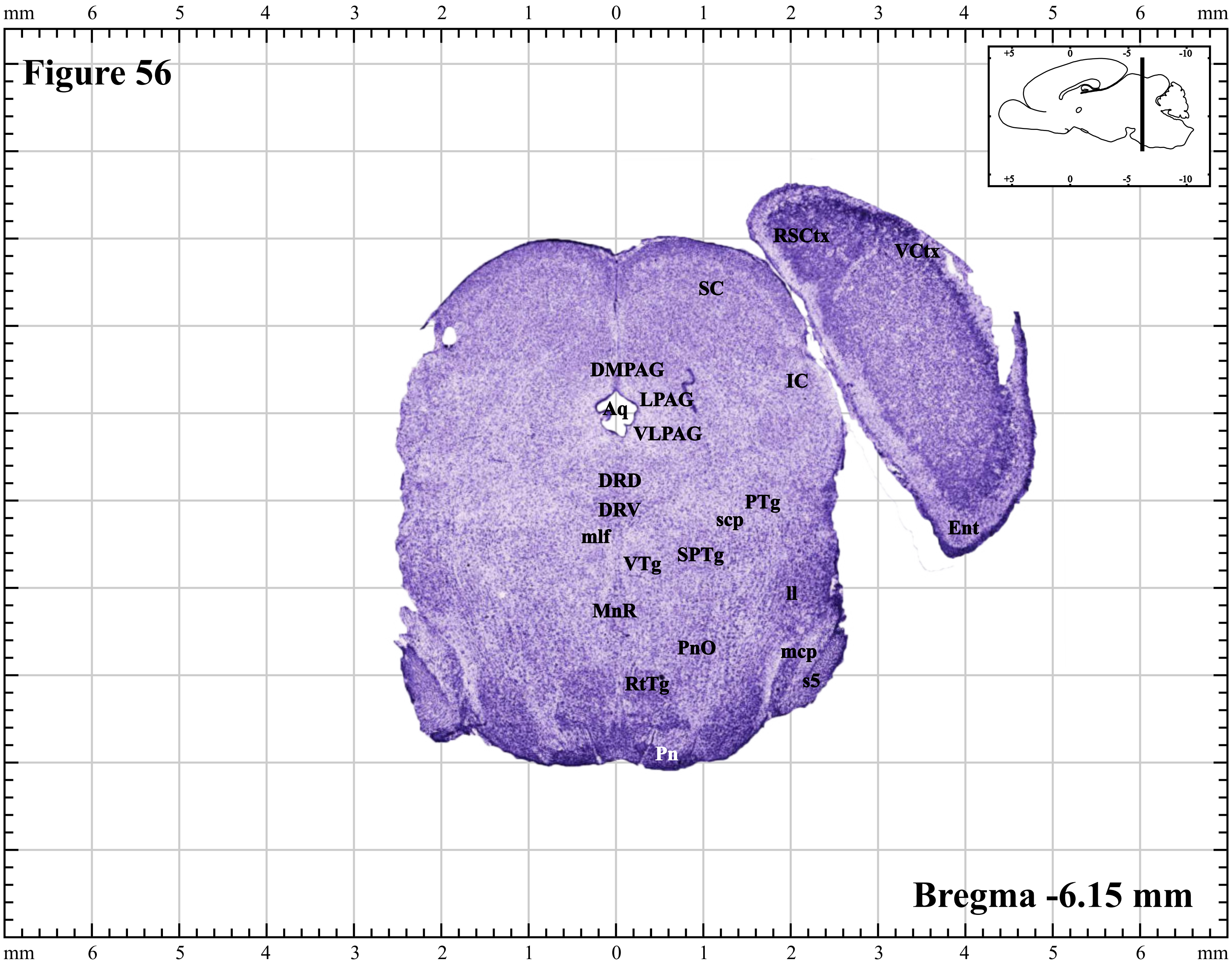

Aq aqueduct
DMPAG dorsomedial periaqueductal gray
DRD dorsomedial hypothalamic nucleus, dorsal part
DRV dorsomedial hypothalamic nucleus, ventral part
Ent entorhinal cortex
IC inferior colliculus
ll lateral lemniscus
LPAG lateral periaqueductal gray
MnR median raphe nucleus
mlf medial longitudinal fasciculus
mcp middle cerebellar peduncle
PnO pontine reticular nucleus, oral part
Pn pontine nuclei
PTg pedunculopontine tegmental nucleus
RSCtx retrospenial cortex
RtTg reticulotegmental nucleus of the pons
scp superior cerebellar peduncle
s5 sensory root of the trigeminal nerve
SC superior colliculus
SPTg subpeduncular tegmental nucleus
VCtx visual cortex
VLPAG ventrolateral periaqueductal gray
VTg ventral tegmental nucleus

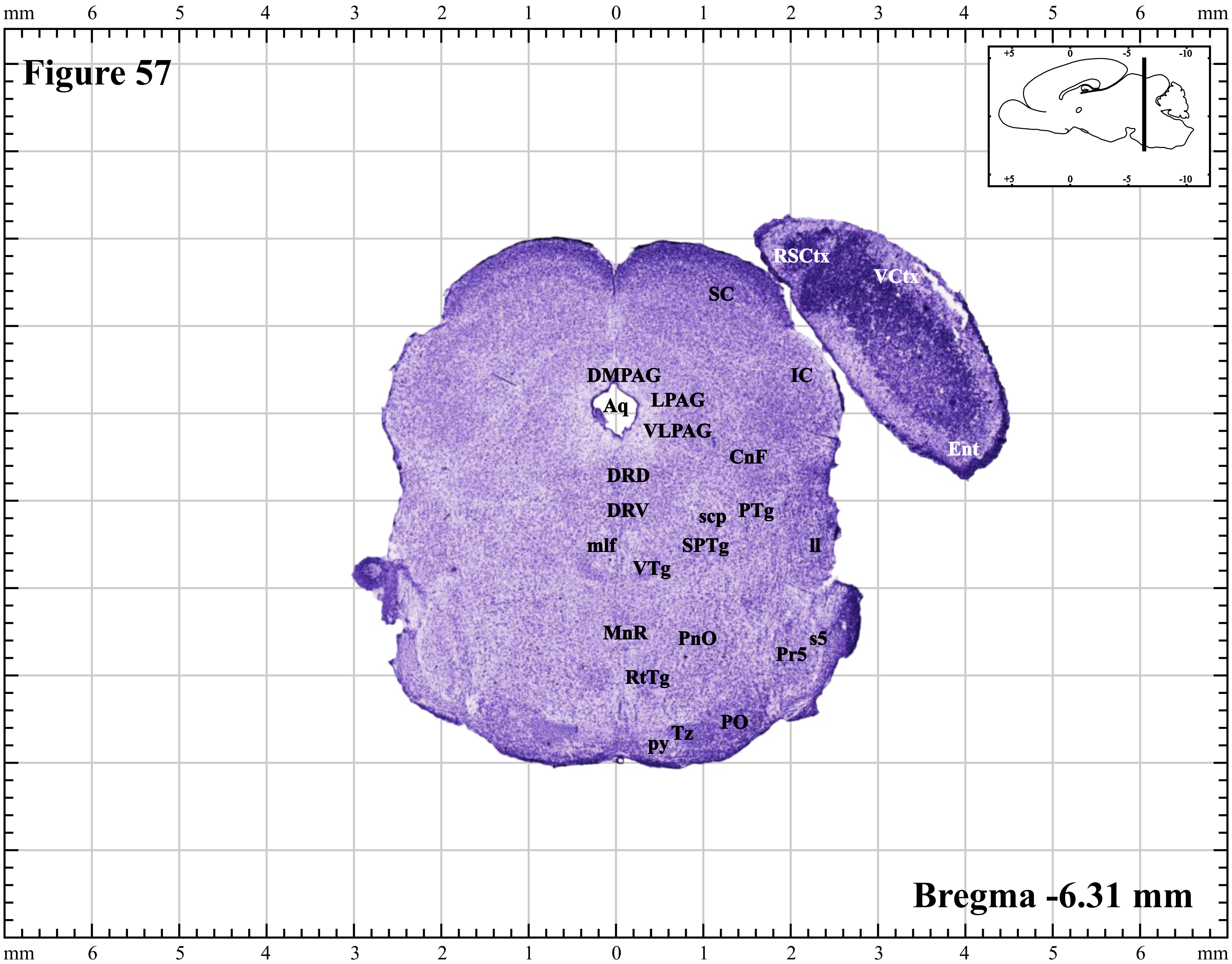

Aq aqueduct
CnF cuneiform nucleus
DMPAG dorsomedial periaqueductal gray
DRD dorsomedial hypothalamic nucleus, dorsal part
DRV dorsomedial hypothalamic nucleus, ventral part
Ent entorhinal cortex
IC inferior colliculus
ll lateral lemniscus
LPAG lateral periaqueductal gray
mlf medial longitudinal fasciculus
MnR median raphe nucleus
py pyramidal tract
PO paraolivary nucleus
PnO pontine reticular nucleus, oral part
PTg pedunculopontine tegmental nucleus
RSCtx retrospenial cortex
RtTg reticulotegmental nucleus of the pons
s5 sensory root of the trigeminal nerve
scp superior cerebellar peduncle
SC superior colliculus
SPTg subpeduncular tegmental nucleus
Tz nucleus of the trapezoid body
VCtx visual cortex
VLPAG ventrolateral periaqueductal gray
VTg ventral tegmental nucleus

Figure 58

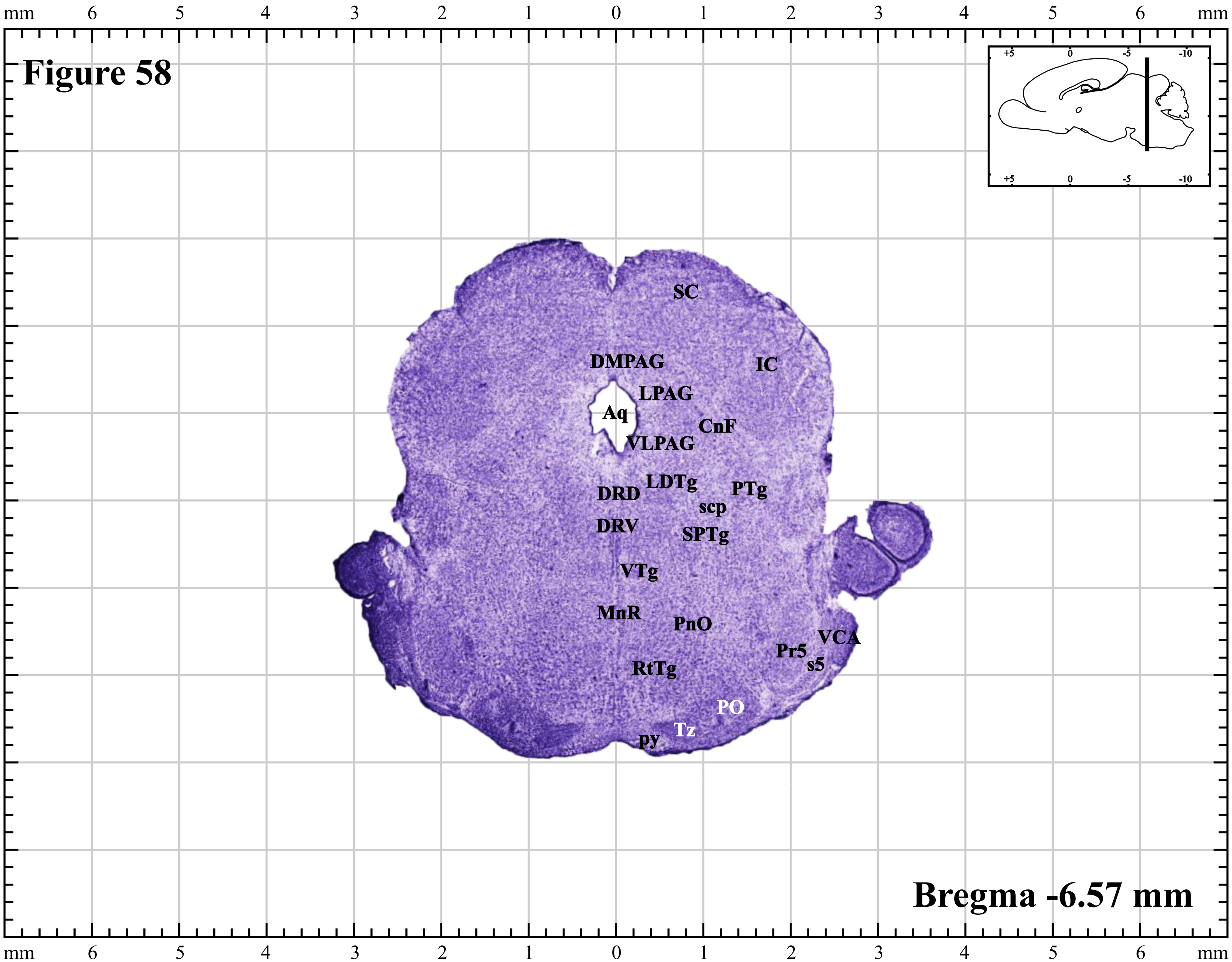

Bregma -6.57 mm

Aq aqueduct
CnF cuneiform nucleus
DMPAG dorsomedial periaqueductal gray
DRD dorsomedial hypothalamic nucleus, dorsal part
DRV dorsomedial hypothalamic nucleus, ventral part
Ent entorhinal cortex
IC inferior colliculus
LDTg laterodorsal tegmental nucleus
LPAG lateral periaqueductal gray
MnR median raphe nucleus
py pyramidal tract
PO paraolivary nucleus
PTg pedunculopontine tegmental nucleus
PnO pontine reticular nucleus, oral part
Pr5 principal sensory trigeminal nucleus
RtTg reticulotegmental nucleus of the pons
s5 sensory root of the trigeminal nerve
scp superior cerebellar peduncle
SC superior colliculus
SPTg subpeduncular tegmental nucleus
Tz nucleus of the trapezoid body
VLPAG ventrolateral periaqueductal gray
VTg ventral tegmental nucleus
VCA ventral cochlear nucleus, anterior part

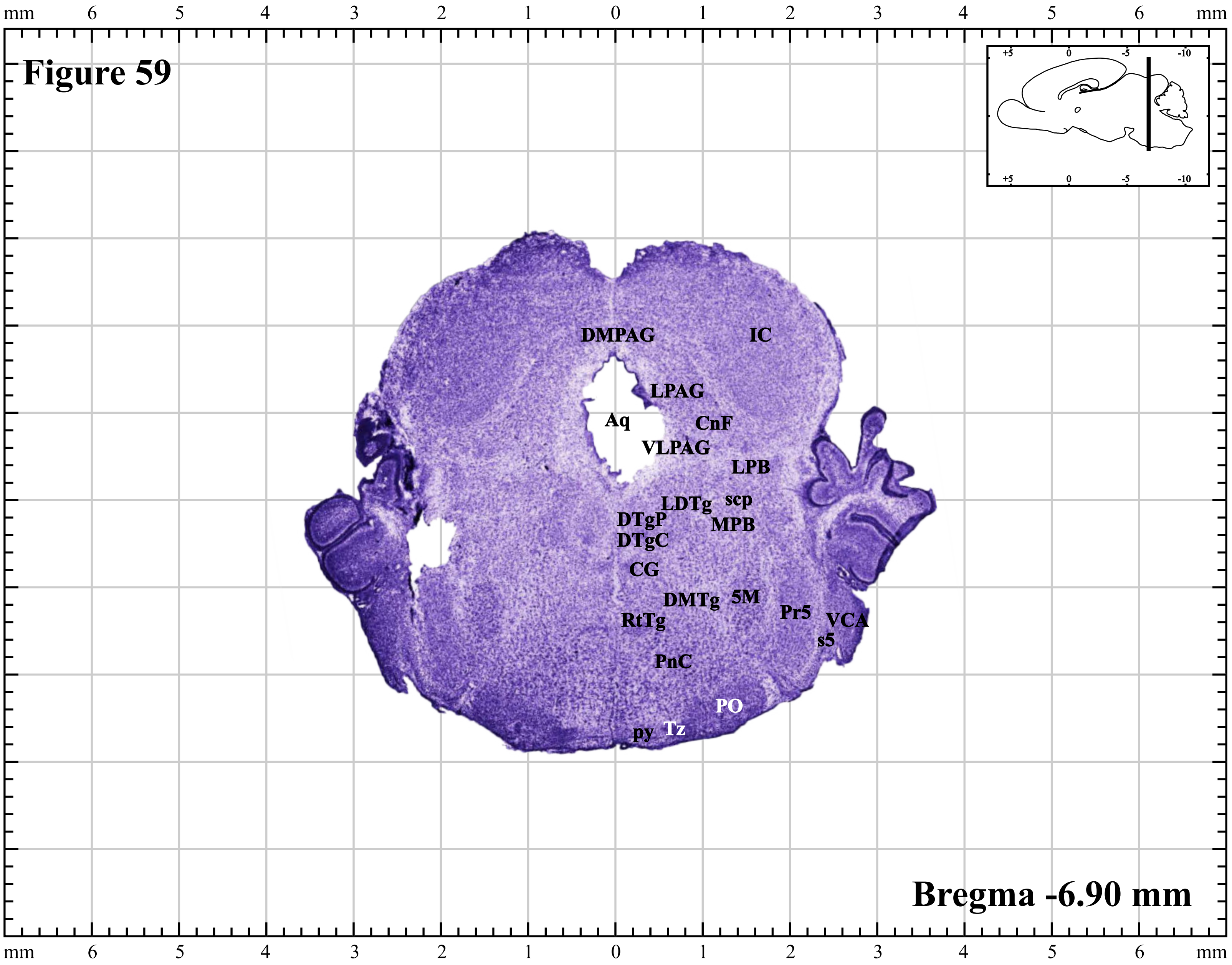

5M motor trigeminal nucleus
Aq aqueduct
CG central gray
CnF cuneiform nucleus
DMPAG dorsomedial periaqueductal gray
DTgP dorsal tegmental nucleus, pericentral part
DTgC dorsal tegmental nucleus, central part
DMTg dorsomedial tegmental area
IC inferior colliculus
LDTg laterodorsal tegmental nucleus
LPAG lateral periaqueductal gray
LPB lateral parabrachial nucleus
MPB medial parabrachial nucleus
py pyramidal tract
PO paraolivary nucleus
PnC pontine reticular nucleus,caudal part
Pr5 principal sensory trigeminal nucleus
s5 sensory root of the trigeminal nerve
scp superior cerebellar peduncle
RtTg reticulotegmental nucleus of the pons
VLPAG ventrolateral periaqueductal gray
VTg ventral tegmental nucleus
VCA ventral cochlear nucleus, anterior part
Tz nucleus of the trapezoid body

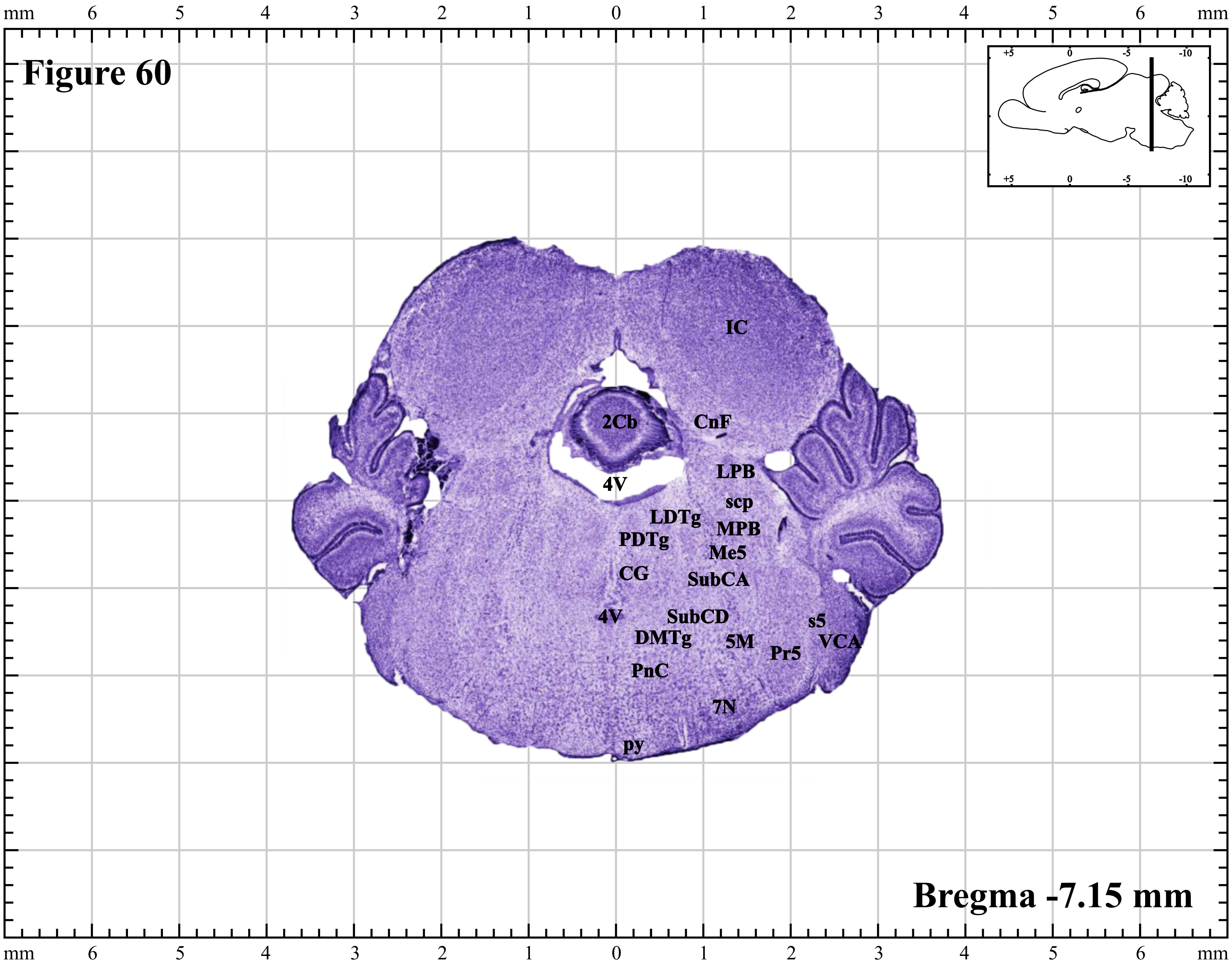

2Cb 2nd cerebellar lobule
4V 4th ventricle
5M motor trigeminal nucleus
7N facial nuclues
CG central gray
CnF cuneiform nucleus
DTgP dorsal tegmental nucleus, pericentral part
DMTg dorsomedial tegmental area
IC inferior colliculus
LDTg laterodorsal tegmental nucleus
Me5 mesencephalic trigeminal nucleus
LPB lateral parabrachial nucleus
MPB medial parabrachial nucleus
py pyramidal tract
PDTg posterodorsal tegmental nucleus
PnC pontine reticular nucleus,caudal part
Pr5 principal sensory trigeminal nucleus
s5 sensory root of the trigeminal nerve
scp superior cerebellar peduncle
SubCA subcoeruleus nucleus, alpha part
SubCD subcoeruleus nucleus, dorsal part
VLPAG ventrolateral periaqueductal gray
VCA ventral cochlear nucleus, anterior part
VTg ventral tegmental nucleus

Figure 61

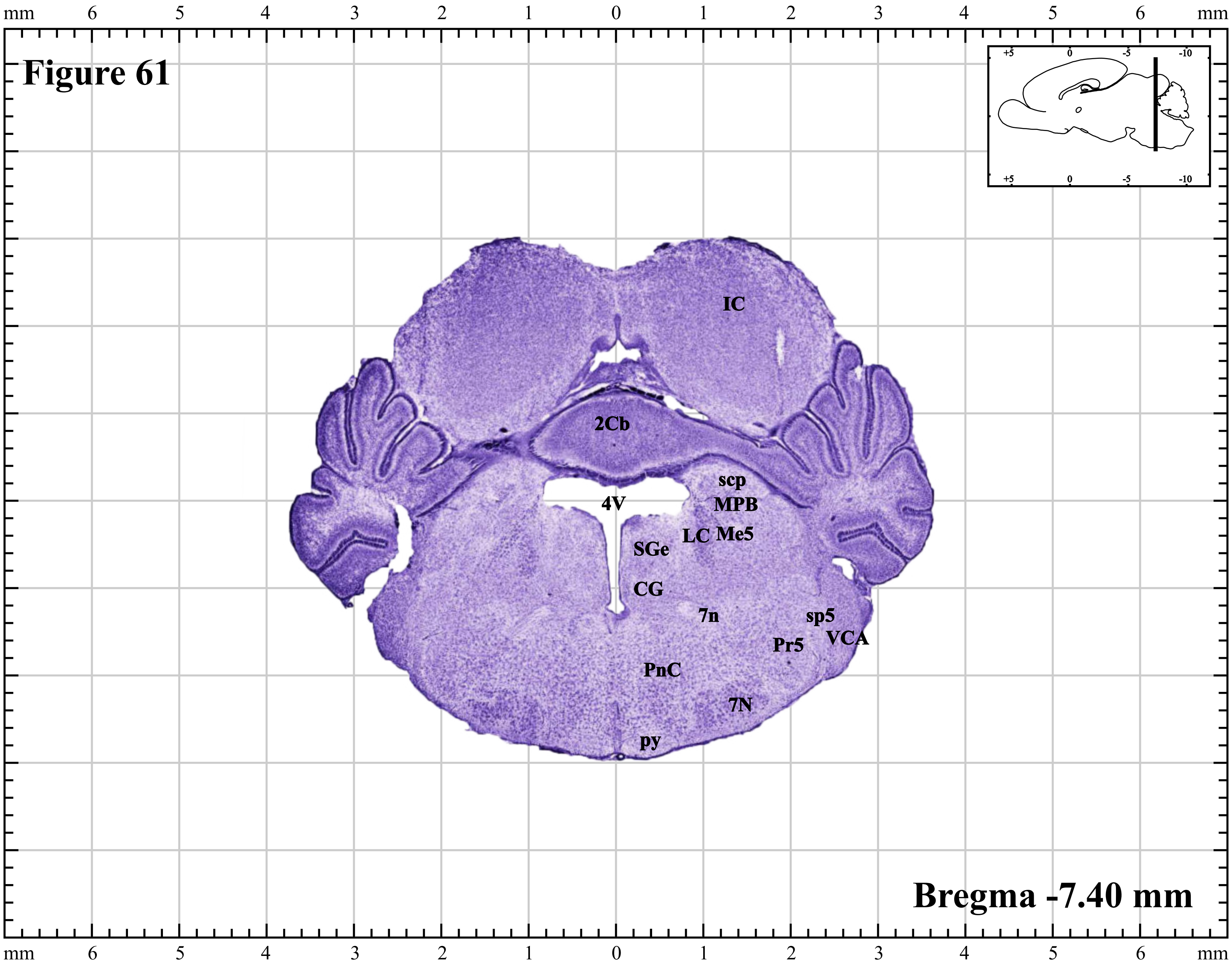

2Cb 2nd cerebellar lobule
4V 4th ventricle
7n facial nerve
7N facial nuclues
CG central gray
IC inferior colliculus
LC locus coeruleus
Me5 mesencephalic trigeminal nucleus
MPB medial parabrachial nucleus
py pyramidal tract
PnC pontine reticular nucleus, caudal part
scp superior cerebellar peduncle
sp5 spinal trigeminal tract
SGe supragenual nucleus
VCA ventral cochlear nucleus, anterior part
Pr5 principal sensory trigeminal nucleus

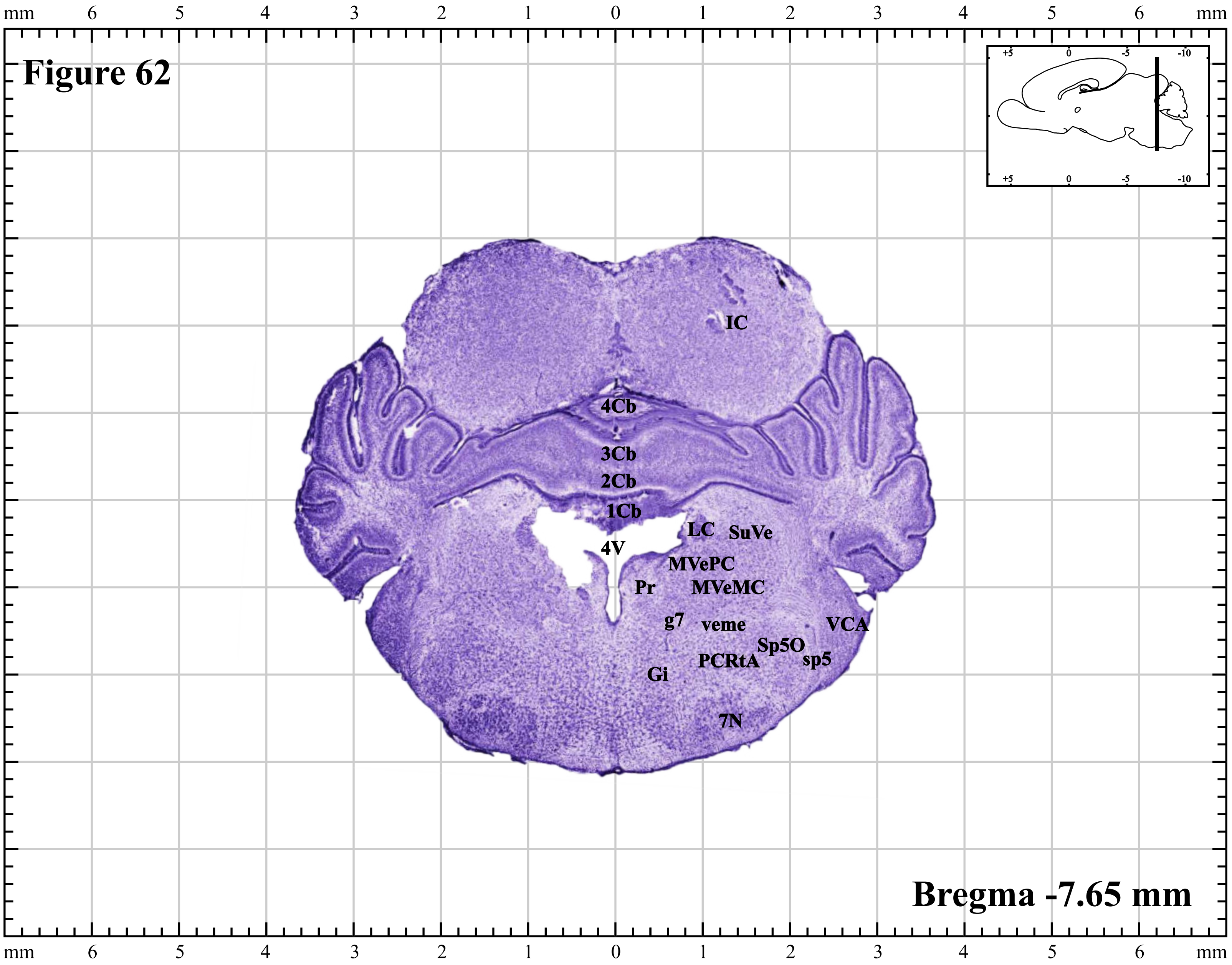

1Cb 1st cerebellar lobule (lingula)
2Cb 2nd cerebellar lobule
3Cb 3rd cerebellar lobule
4Cb 3rd cerebellar lobule
4V 4th ventricle
7N facial nuclues
g7 genu of the facial nerve
Gi granular insular cortex
IC inferior colliculus
LC locus coeruleus
MVeMC medial vestibular nucleus, magnocellular part
MVePC medial vestibular nucleus, parvicellular part
PCRtA parvicellular reticular nucleus, alpha part
Pr prepositus nucleus
SuVe superior vestibular nucleus
Sp5O spinal trigeminal nucleus, oral part
sp5 spinal trigeminal tract
VCA ventral cochlear nucleus, anterior part
veme vestibulomesencephalic tract

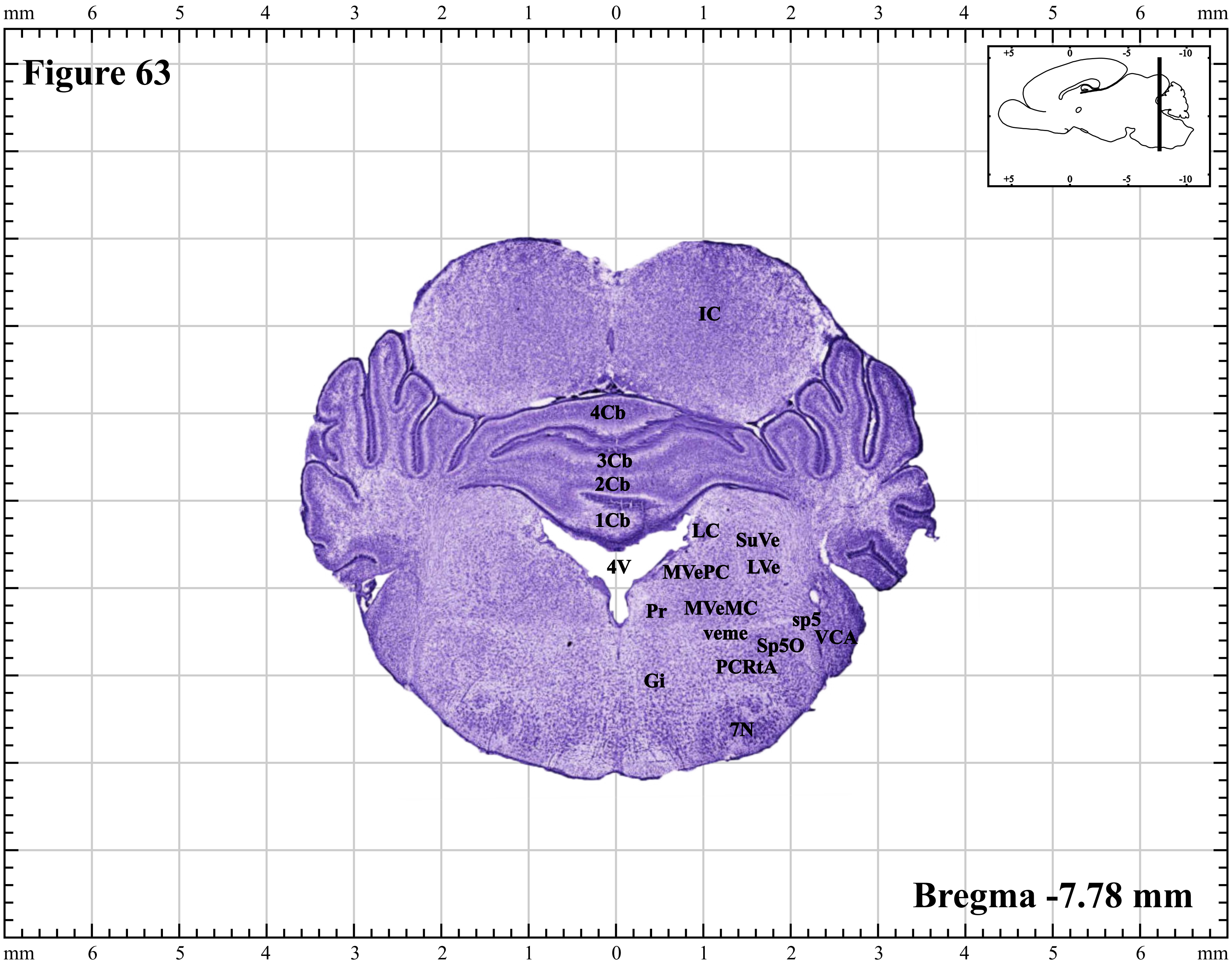

1Cb 1st cerebellar lobule (lingula)
2Cb 2nd cerebellar lobule
3Cb 3rd cerebellar lobule
4Cb 4th cerebellar lobule
4V 4th ventricle
7N facial nuclues
Gi granular insular cortex
LC locus coeruleus
IC inferior colliculus
LVe lateral vestibular nucleus
MVeMC medial vestibular nucleus, magnocellular part
MVePC medial vestibular nucleus, parvicellular part
PCRtA parvicellular reticular nucleus, alpha part
Pr prepositus nucleus
SuVe superior vestibular nucleus
Sp5O spinal trigeminal nucleus, oral part
sp5 spinal trigeminal tract
VCA ventral cochlear nucleus, anterior part
veme vestibulomesencephalic tract

Figure 64

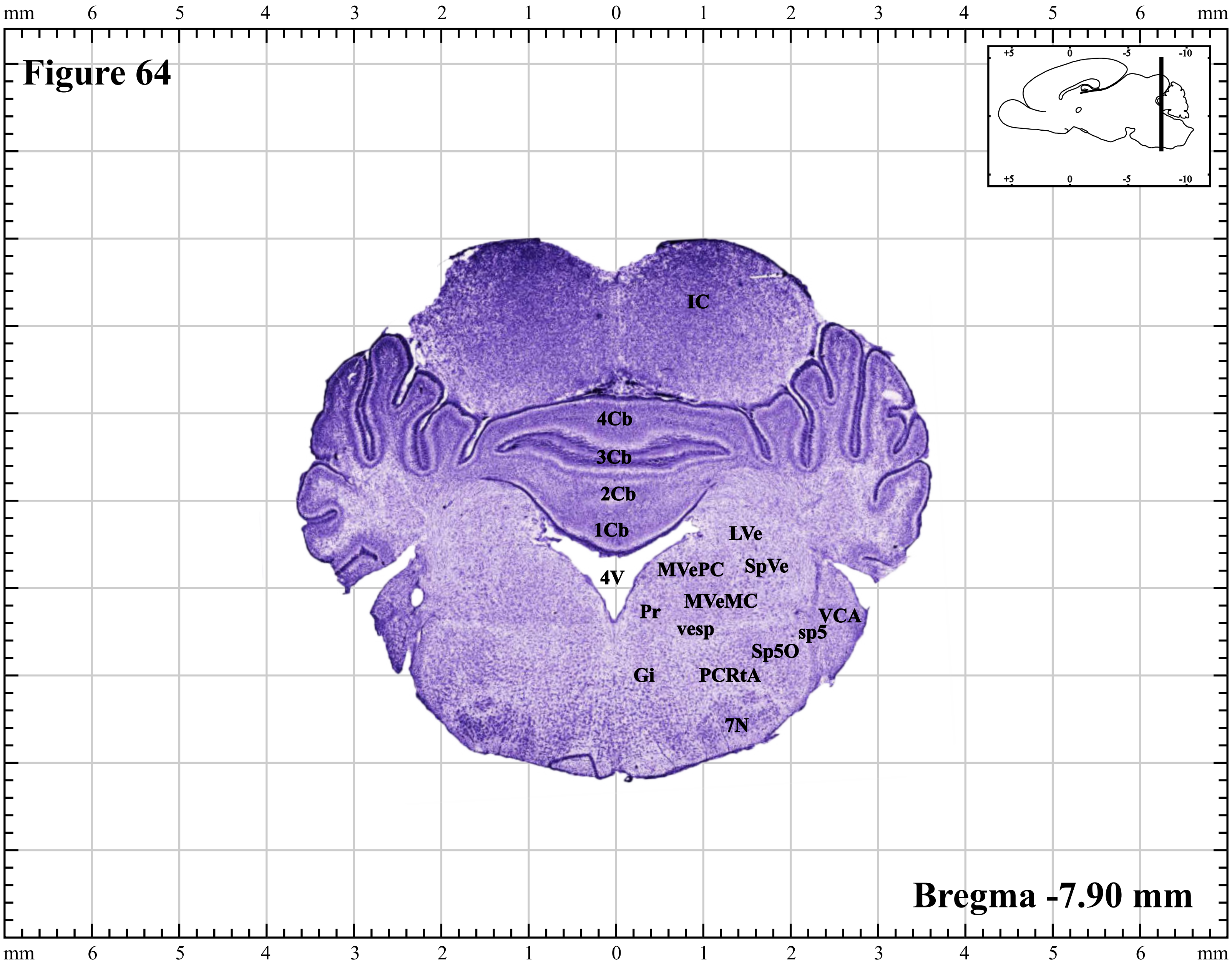

Bregma -7.90 mm

1Cb 1st cerebellar lobule (lingula)
2Cb 2nd cerebellar lobule
3Cb 3rd cerebellar lobule
4Cb 4th cerebellar lobule
4V 4th ventricle
7N facial nuclues
Gi granular insular cortex
IC inferior colliculus
LVe lateral vestibular nucleus
MVeMC medial vestibular nucleus, magnocellular part
MVePC medial vestibular nucleus, parvicellular part
PCRtA parvicellular reticular nucleus, alpha part
Pr prepositus nucleus
SpVe spinal vestibular nucleus
Sp5O spinal trigeminal nucleus, oral part
sp5 spinal trigeminal tract
VCA ventral cochlear nucleus, anterior part
vesp vestibulospinal tract

Figure 65

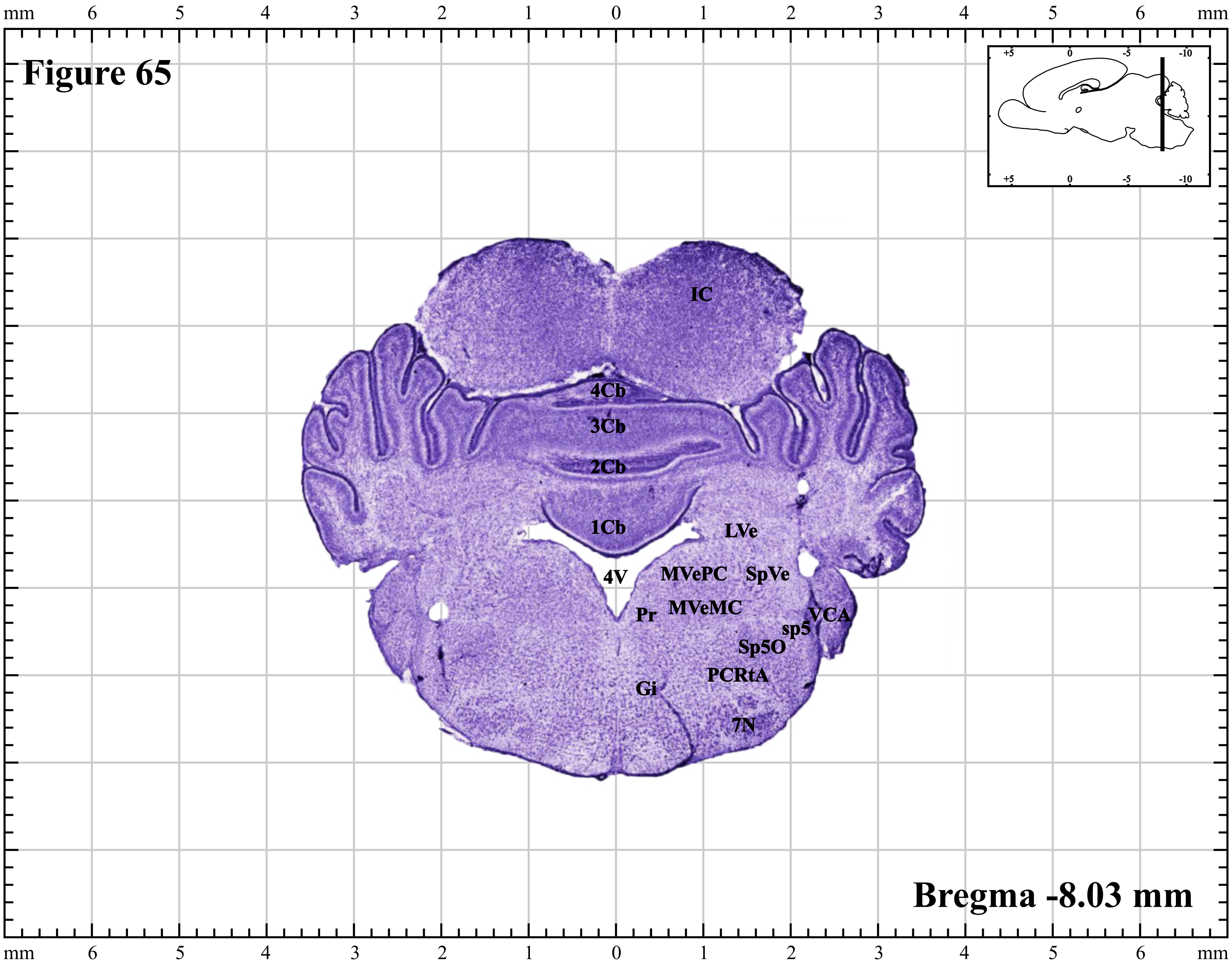

Bregma -8.03 mm

1Cb 1st cerebellar lobule (lingula)
2Cb 2nd cerebellar lobule
3Cb 3rd cerebellar lobule
4Cb 4th cerebellar lobule
4V 4th ventricle
7N facial nuclues
Gi granular insular cortex
IC inferior colliculus
LC locus coeruleus
LVe lateral vestibular nucleus
Me5 mesencephalic trigeminal nucleus
MVeMC medial vestibular nucleus, magnocellular part
MVePC medial vestibular nucleus, parvicellular part
PCRtA parvicellular reticular nucleus, alpha part
Pr prepositus nucleus
sp5 spinal trigeminal tract
SpVe spinal vestibular nucleus
Sp5O spinal trigeminal nucleus, oral part
VCA ventral cochlear nucleus, anterior part

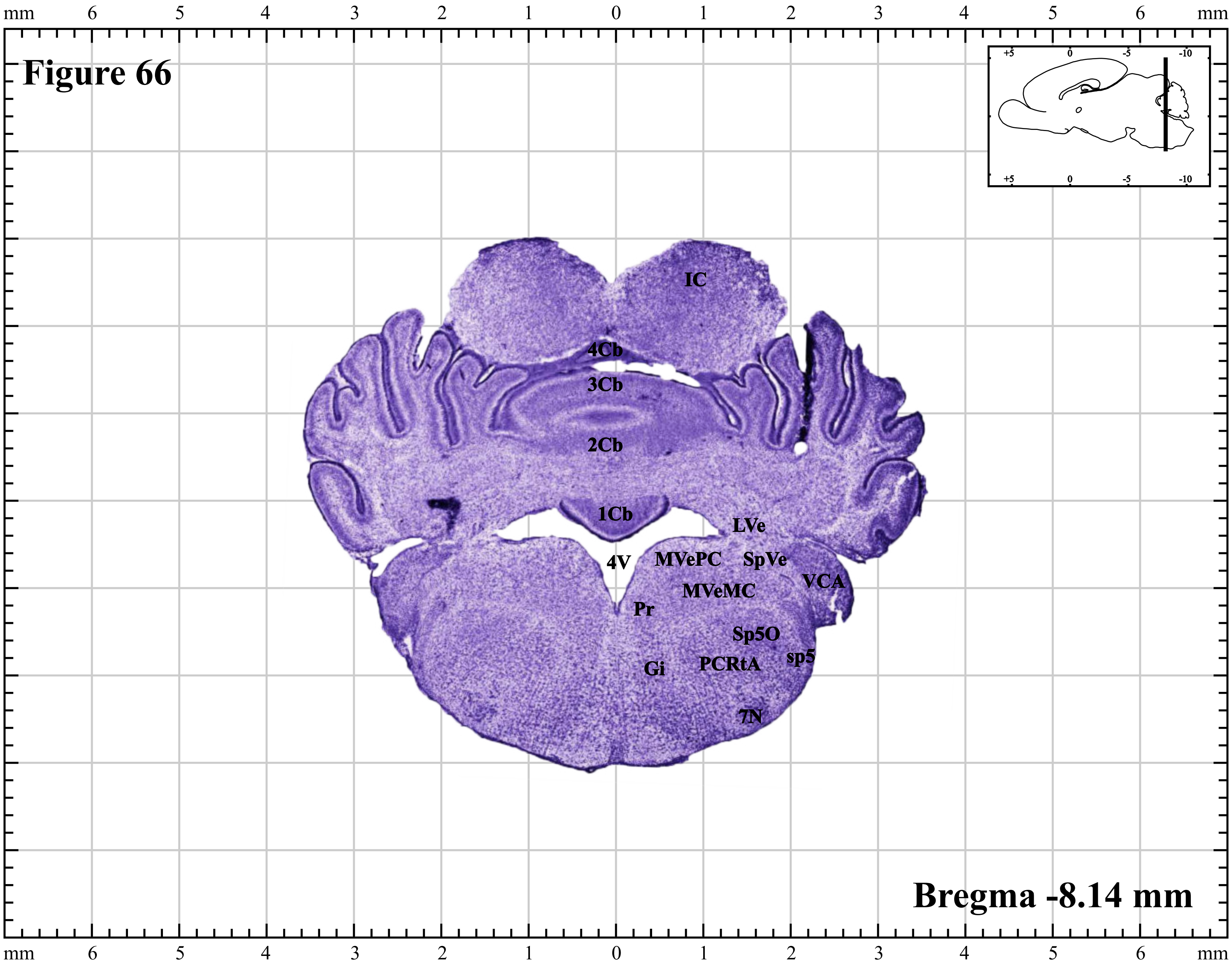

1Cb 1st cerebellar lobule (lingula)
2Cb 2nd cerebellar lobule
3Cb 3rd cerebellar lobule
4Cb 4th cerebellar lobule
4V 4th ventricle
7N facial nuclues
Gi granular insular cortex
IC inferior colliculus
LC locus coeruleus
LVe lateral vestibular nucleus
Me5 mesencephalic trigeminal nucleus
MVeMC medial vestibular nucleus, magnocellular part
MVePC medial vestibular nucleus, parvicellular part
PCRtA parvicellular reticular nucleus, alpha part
Pr prepositus nucleus
sp5 spinal trigeminal tract
SpVe spinal vestibular nucleus
Sp5O spinal trigeminal nucleus, oral part
VCA ventral cochlear nucleus, anterior part

Figure 67

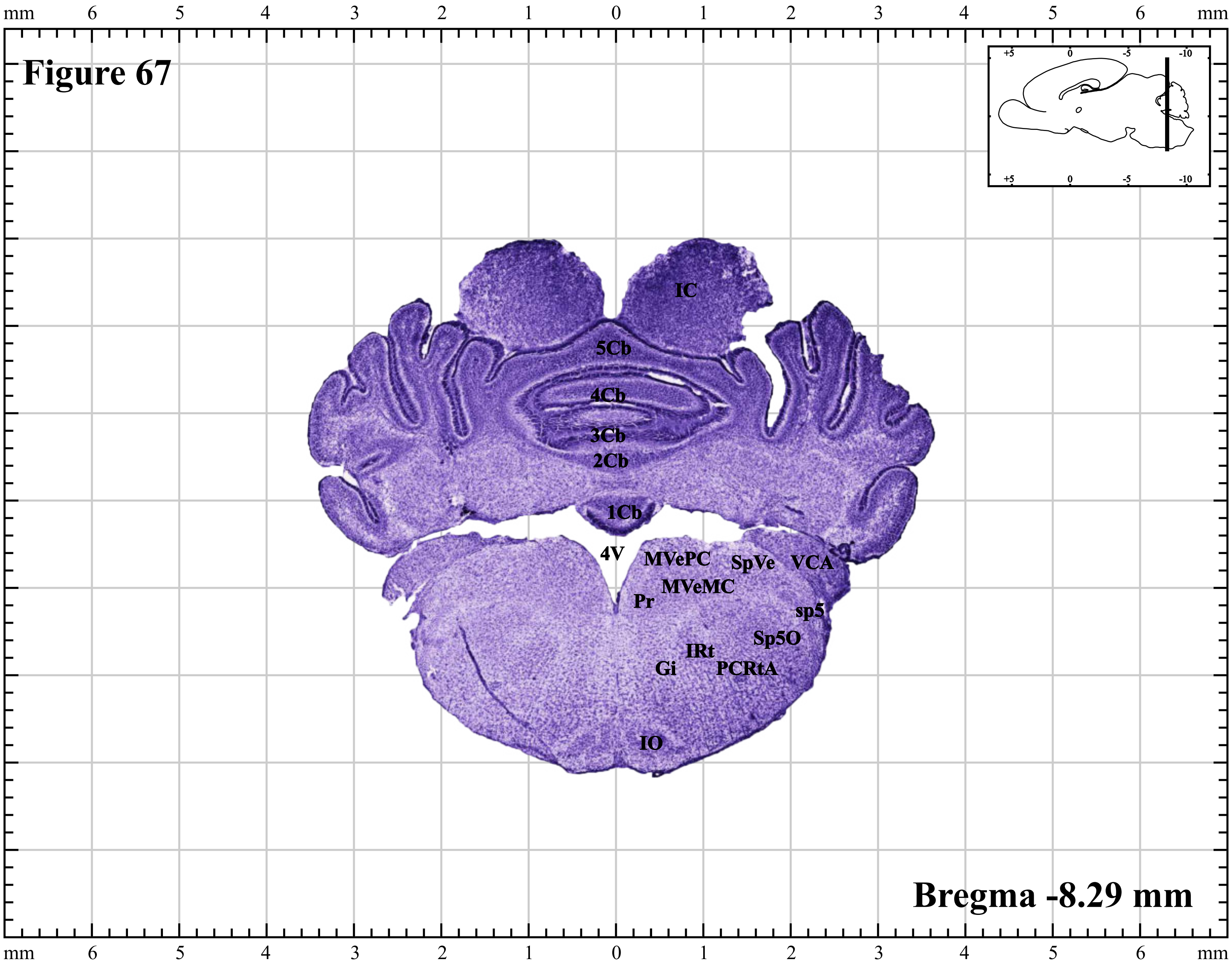

Bregma -8.29 mm

1Cb 1st cerebellar lobule (lingula)
2Cb 2nd cerebellar lobule
3Cb 3rd cerebellar lobule
4Cb 4th cerebellar lobule
4V 4th ventricle
5Cb 5th cerebellar lobule
Gi granular insular cortex
IO inferior olive
IRt intermediate reticular nucleus
IC inferior colliculus
LVe lateral vestibular nucleus
Me5 mesencephalic trigeminal nucleus
MVeMC medial vestibular nucleus, magnocellular part
MVePC medial vestibular nucleus, parvicellular part
PCRtA parvicellular reticular nucleus, alpha part
Pr prepositus nucleus
sp5 spinal trigeminal tract
SpVe spinal vestibular nucleus
Sp5O spinal trigeminal nucleus, oral part
VCA ventral cochlear nucleus, anterior part

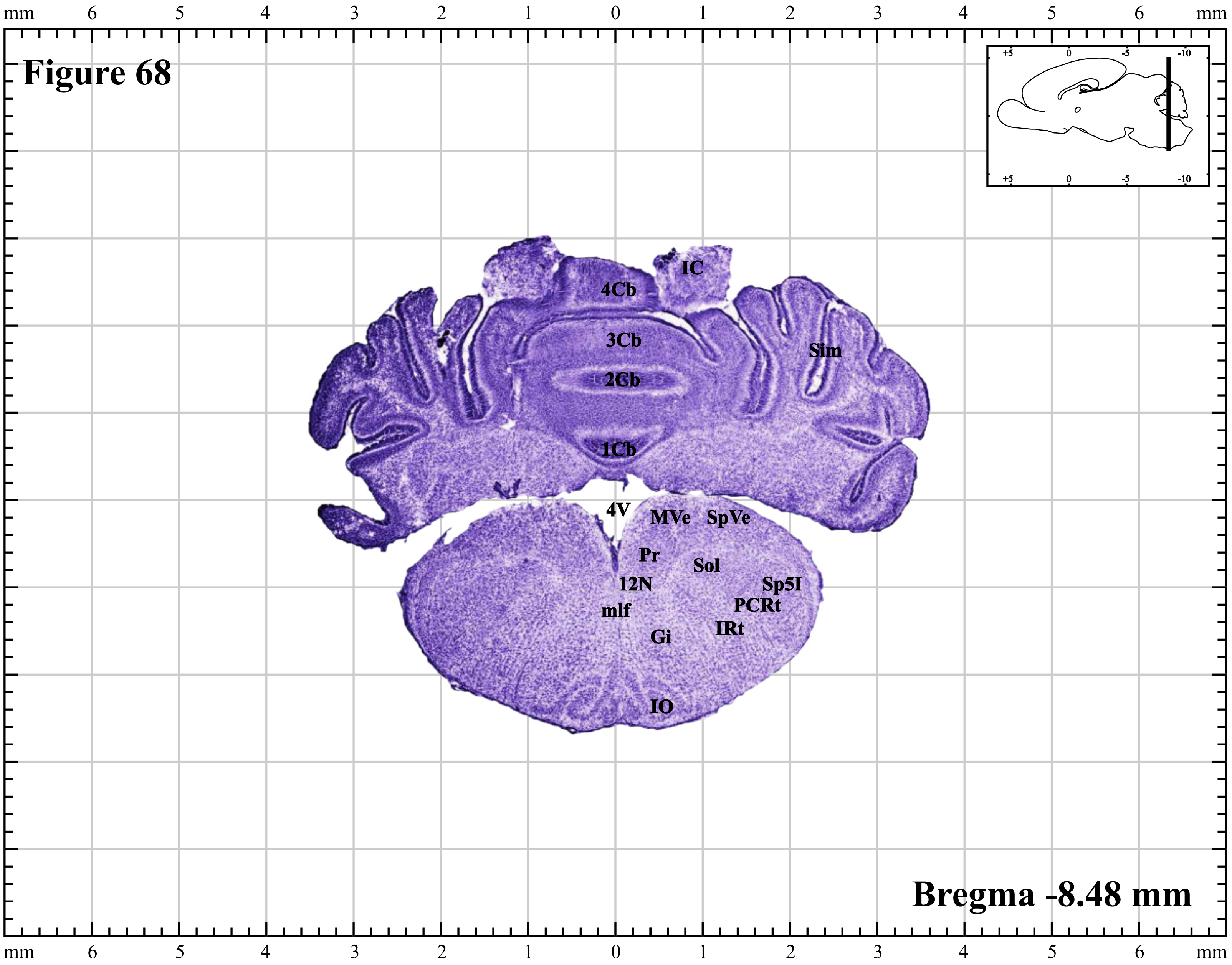

1Cb 1st cerebellar lobule (lingula)
2Cb 2nd cerebellar lobule
3Cb 3rd cerebellar lobule
4Cb 4th cerebellar lobule
4V 4th ventricle
12N hypoglossal nucleus
Gi granular insular cortex
IO inferior olive
IC inferior colliculus
IRt intermediate reticular nucleus
mlf medial longitudinal fasciculus
MVe medial vestibular nucleus
PCRt parvicellular reticular nucleus
Pr prepositus nucleus
Sol nucleus of the solitary tract
Sim simple lobule
SpVe spinal vestibular nucleus
Sp5I spinal trigeminal nucleus, interpolar part

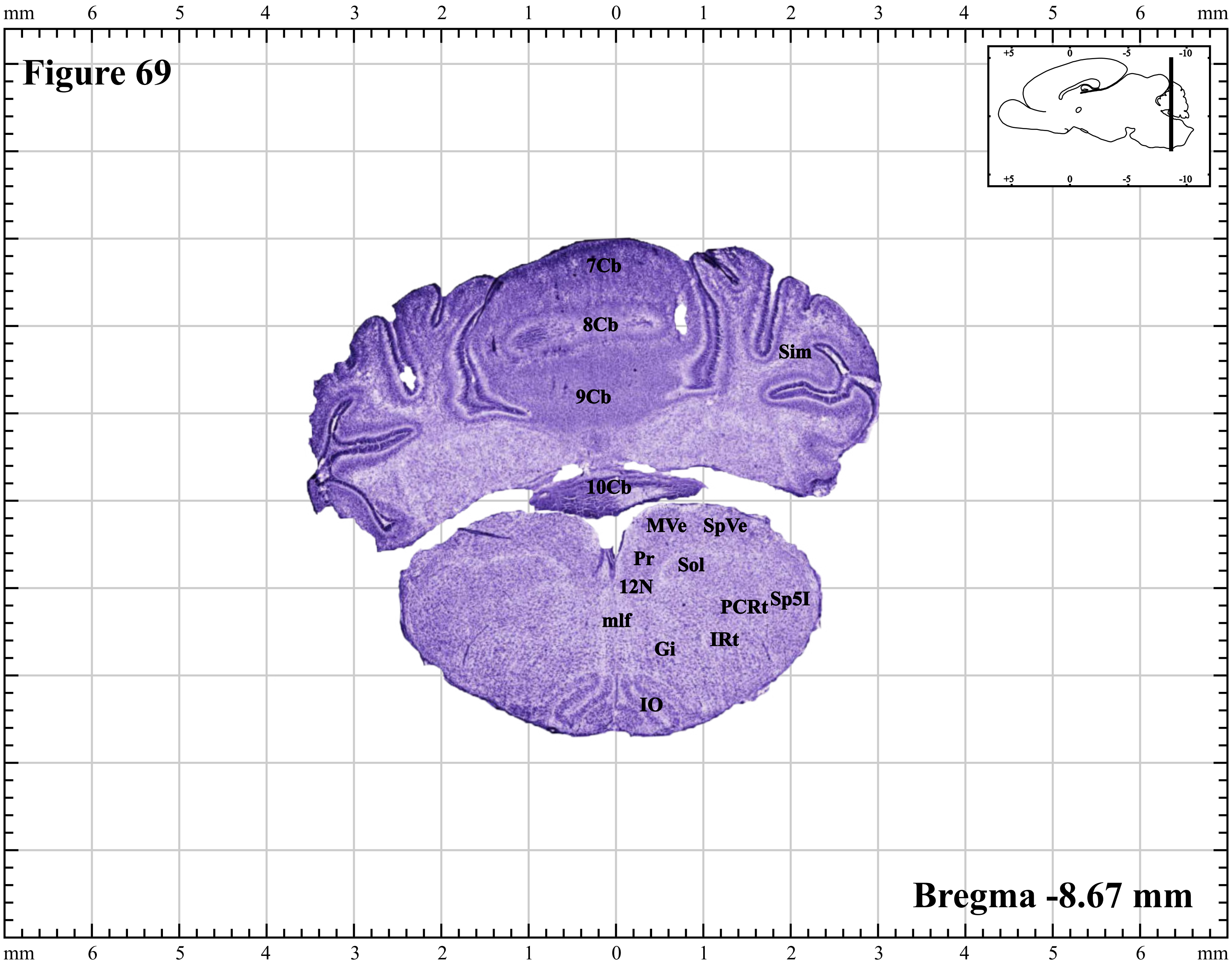

4V 4th ventricle
7Cb 7th cerebellar lobule
8Cb 8th cerebellar lobule
9Cb 9th cerebellar lobules
10Cb 10th cerebellar lobule
12N hypoglossal nucleus
Gi granular insular cortex
IO inferior olive
IRt intermediate reticular nucleus
mlf medial longitudinal fasciculus
MVe medial vestibular nucleus
Pr prepositus nucleus
PCRt parvicellular reticular nucleus
Sol nucleus of the solitary tract
Sim simple lobule
SpVe spinal vestibular nucleus
Sp5I spinal trigeminal nucleus, interpolar part

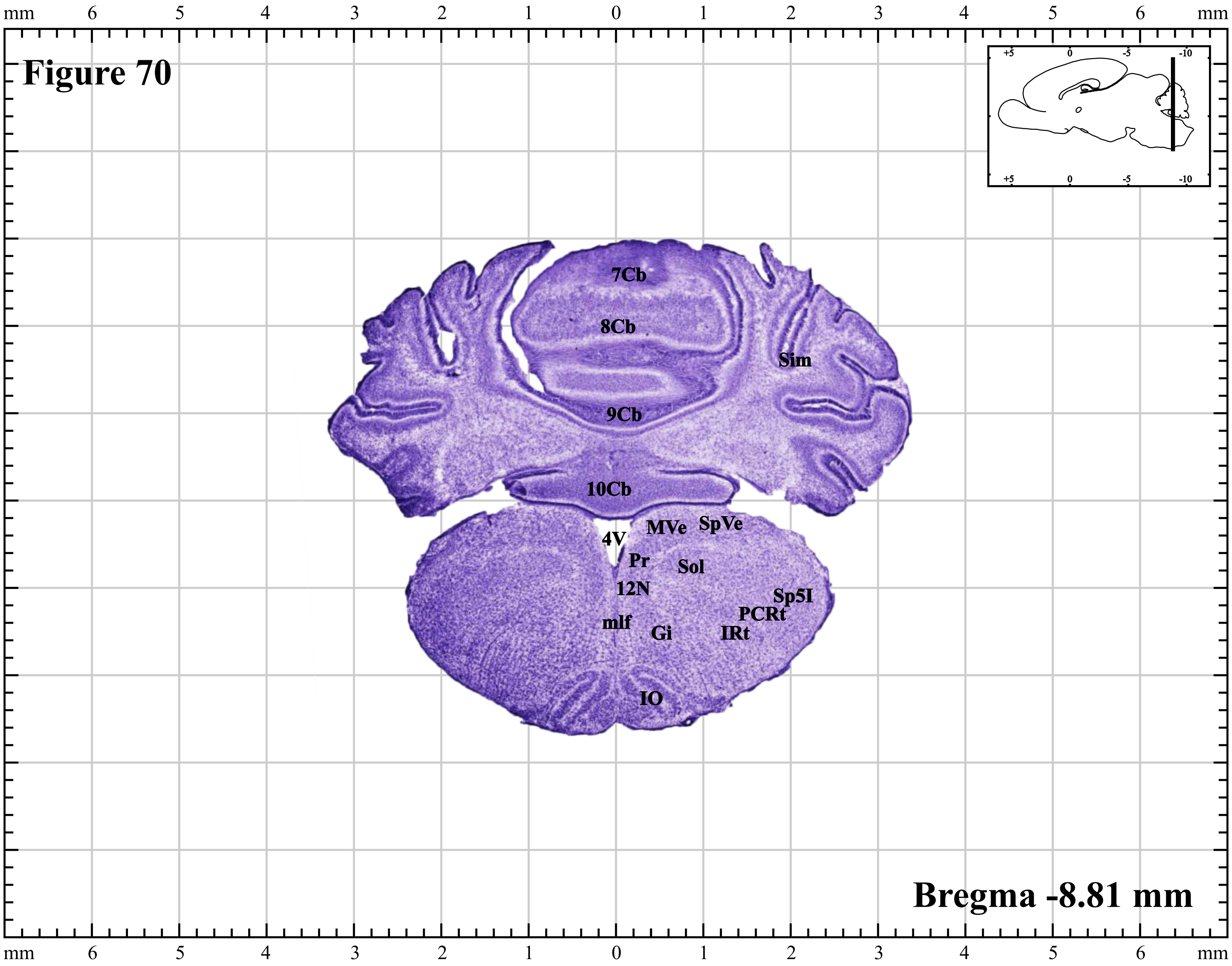

4V 4th ventricle
7Cb 7th cerebellar lobule
8Cb 8th cerebellar lobule
9Cb 9th cerebellar lobules
10Cb 10th cerebellar lobule
12N hypoglossal nucleus
Gi granular insular cortex
IO inferior olive
IRt intermediate reticular nucleus
mlf medial longitudinal fasciculus
MVe medial vestibular nucleus
Pr prepositus nucleus
PCRt parvicellular reticular nucleus
Sol nucleus of the solitary tract
Sim simple lobule
SpVe spinal vestibular nucleus
Sp5I spinal trigeminal nucleus, interpolar part

Figure 71

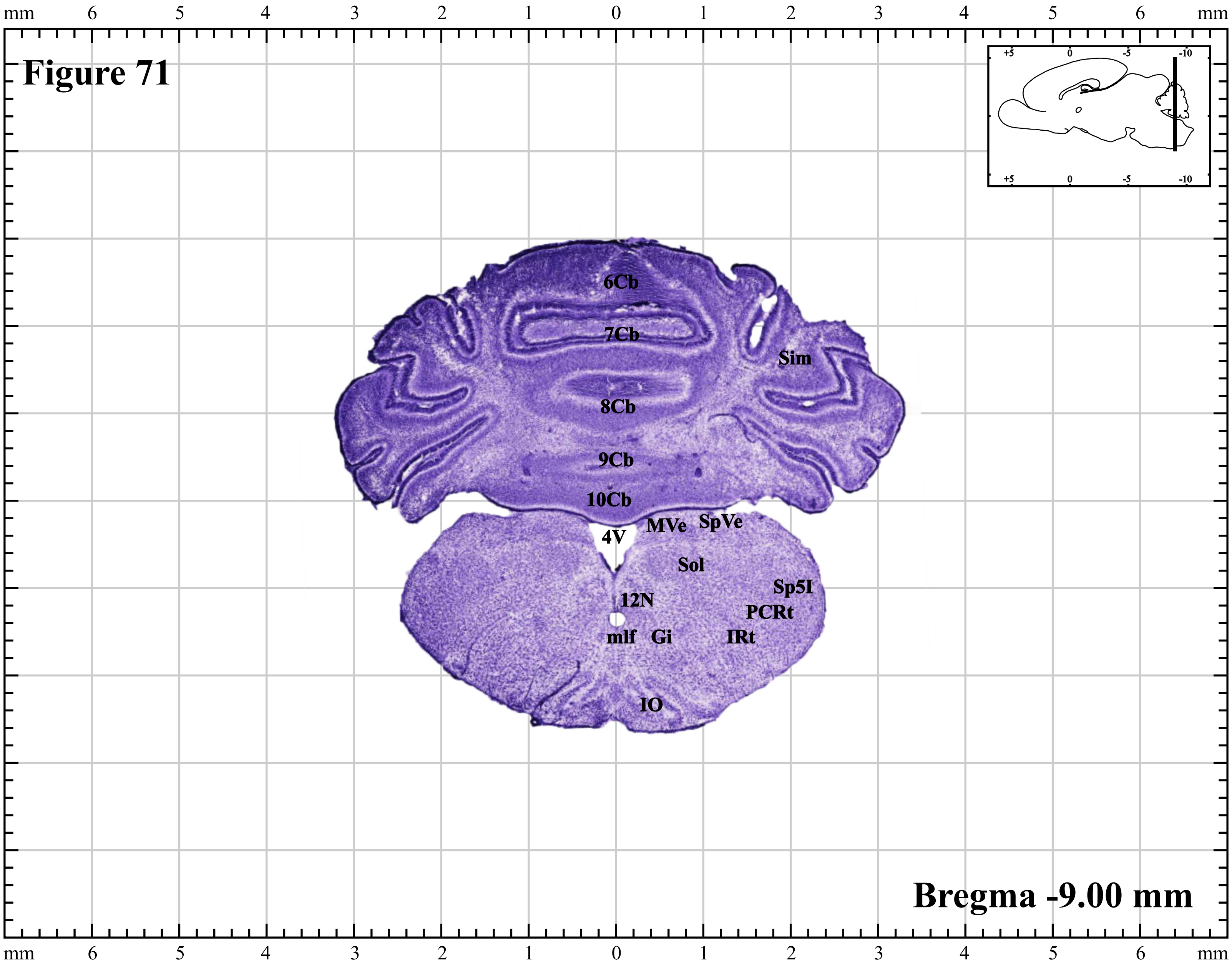

Bregma -9.00 mm

4V 4th ventricle
6Cb 6th cerebellar lobule
7Cb 7th cerebellar lobule
8Cb 8th cerebellar lobule
9Cb 9th cerebellar lobules
10Cb 10th cerebellar lobule
12N hypoglossal nucleus
Gi granular insular cortex
IO inferior olive
IRt intermediate reticular nucleus
mlf medial longitudinal fasciculus
MVe medial vestibular nucleus
PCRt parvicellular reticular nucleus
Sol nucleus of the solitary tract
Sim simple lobule
SpVe spinal vestibular nucleus
Sp5I spinal trigeminal nucleus, interpolar part

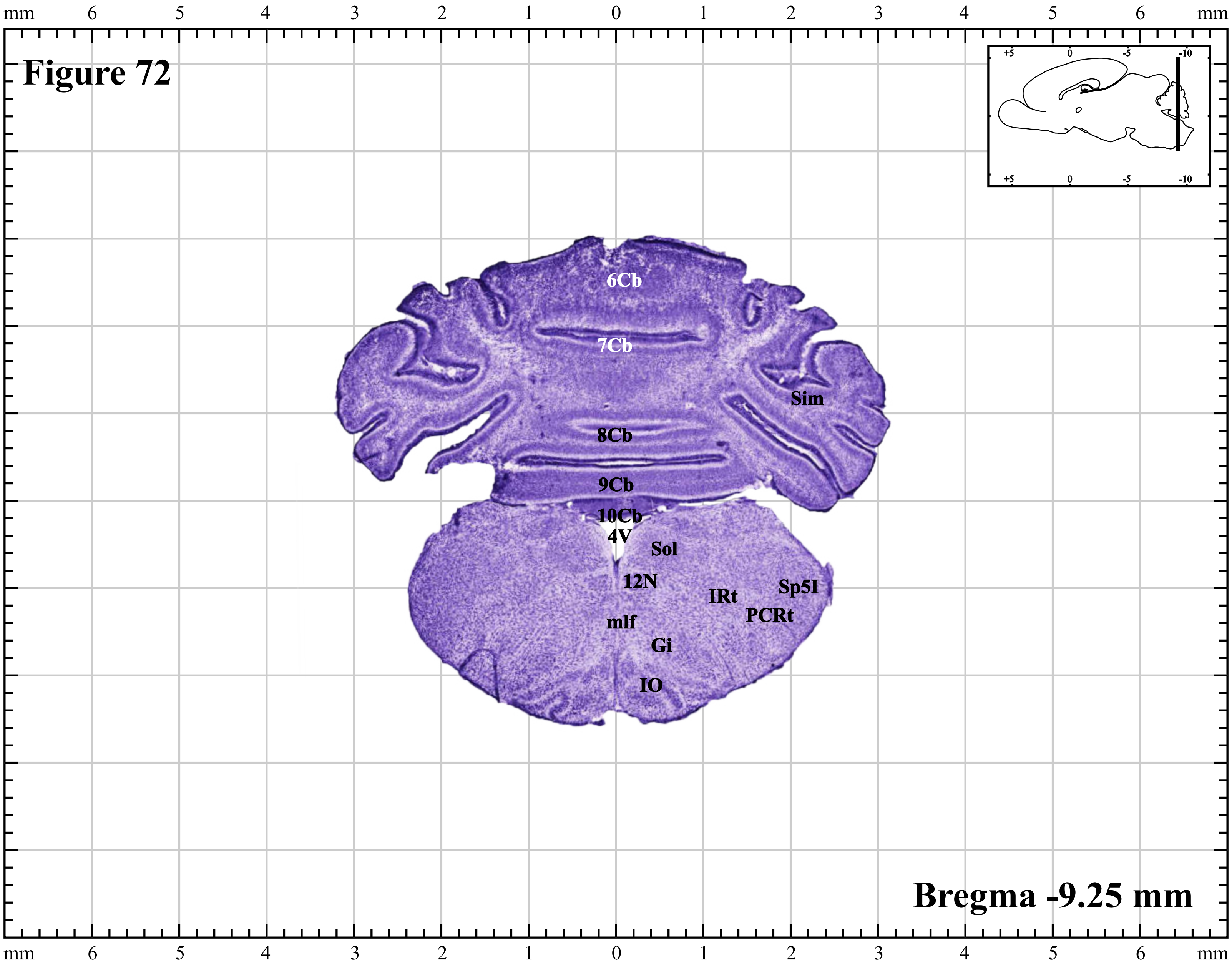

- **4V** 4th ventricle
- **6Cb** 6th cerebellar lobule
- **7Cb** 7th cerebellar lobule
- **8Cb** 8th cerebellar lobule
- **9Cb** 9th cerebellar lobules
- **10Cb** 10th cerebellar lobule
- **12N** hypoglossal nucleus
- **Gi** granular insular cortex
- **IO** inferior olive
- **IRt** intermediate reticular nucleus
- **mlf** medial longitudinal fasciculus
- **PCRt** parvicellular reticular nucleus
- **Sol** nucleus of the solitary tract
- **Sim** simple lobule
- **Sp5I** spinal trigeminal nucleus, interpolar part

Figure 73

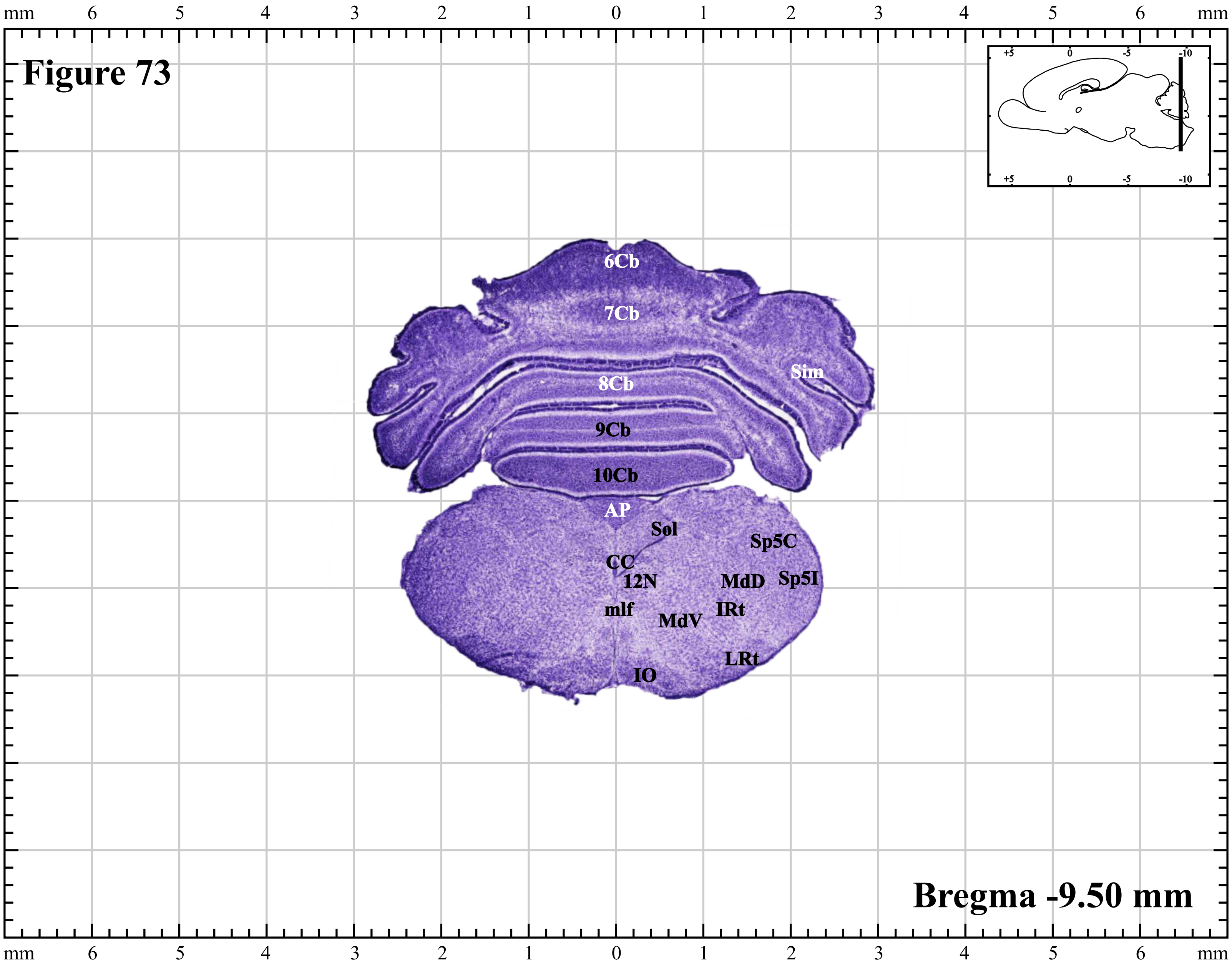

Bregma -9.50 mm

7Cb 7th cerebellar lobule
8Cb 8th cerebellar lobule
9Cb 9th cerebellar lobules
10Cb 10th cerebellar lobule
12N hypoglossal nucleus
AP area postrema
CC central canal
Gi granular insular cortex
IO inferior olive

IRt intermediate reticular nucleus
LRt lateral reticular nucleus
mlf medial longitudinal fasciculus
MdD medullary reticular nucleus, dorsal part
MdV medullary reticular nucleus, ventral part
Sol nucleus of the solitary tract
Sim simple lobule
Sp5I spinal trigeminal nucleus, interpolar part
Sp5C spinal trigeminal nucleus, caudal part

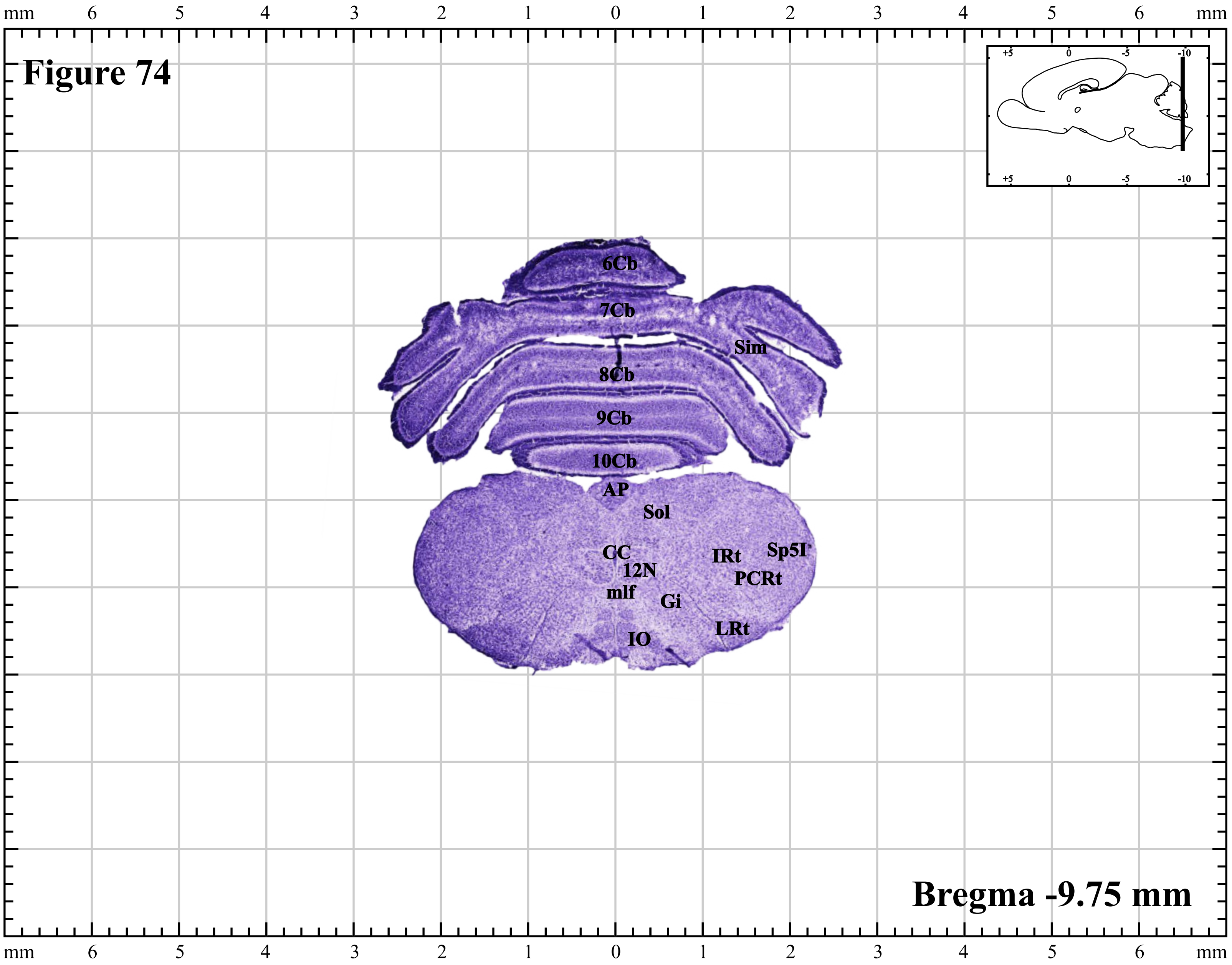

7Cb 7th cerebellar lobule
8Cb 8th cerebellar lobule
9Cb 9th cerebellar lobules
10Cb 10th cerebellar lobule
12N hypoglossal nucleus
CC central canal
AP area postrema
Gi granular insular cortex
IO inferior olive
IRt intermediate reticular nucleus
LRt lateral reticular nucleus
mlf medial longitudinal fasciculus
PCRt parvicellular reticular nucleus
Sol nucleus of the solitary tract
Sim simple lobule
Sp5I spinal trigeminal nucleus, interpolar part
