# Supplementary material for: Stereotaxic atlas of the infant rat brain at postnatal days 7–13
Source: Front Neuroanat. 2022 Aug 12;16:968320. doi: 10.3389/fnana.2022.968320 (PMC9412974; doi:10.3389/fnana.2022.968320)
Supplement: Supplementary file 3 [file Data_Sheet_3.PDF]

Supplementary Material 3

Stereotaxic Atlas of the Infant Rat Brain

P9 (# G-14-4, 21.6 g)

Yu-Nong Chen[1], Xin Zheng[1], Hai-Lin Chen[1], Jin-Xian Gao[1], Xin-Xuan Li[1], Jun-Fan Xie[1], Yu-Ping Xie[3], Karen Spruyt[4], Yu-Feng Shao[1,2]* and Yi-Ping Hou[1,2]*

[1]Departments of Neuroscience, Anatomy, Histology, and Embryology, Key Laboratory of Preclinical Study for New Drugs of Gansu Province, School of Basic Medical Sciences, Lanzhou University, Lanzhou, China
[2]Key Lab of Neurology of Gansu Province, Lanzhou University, Lanzhou, China
[3]Sleep Medicine Center of Gansu Provincial Hospital, Lanzhou, China
[4]Université de Paris, NeuroDiderot – INSERM, Paris, France.

* Correspondence: Yu-Feng Shao (shaoyf@lzu.edu.cn); Yi-Ping Hou (houyiping@lzu.edu.cn)

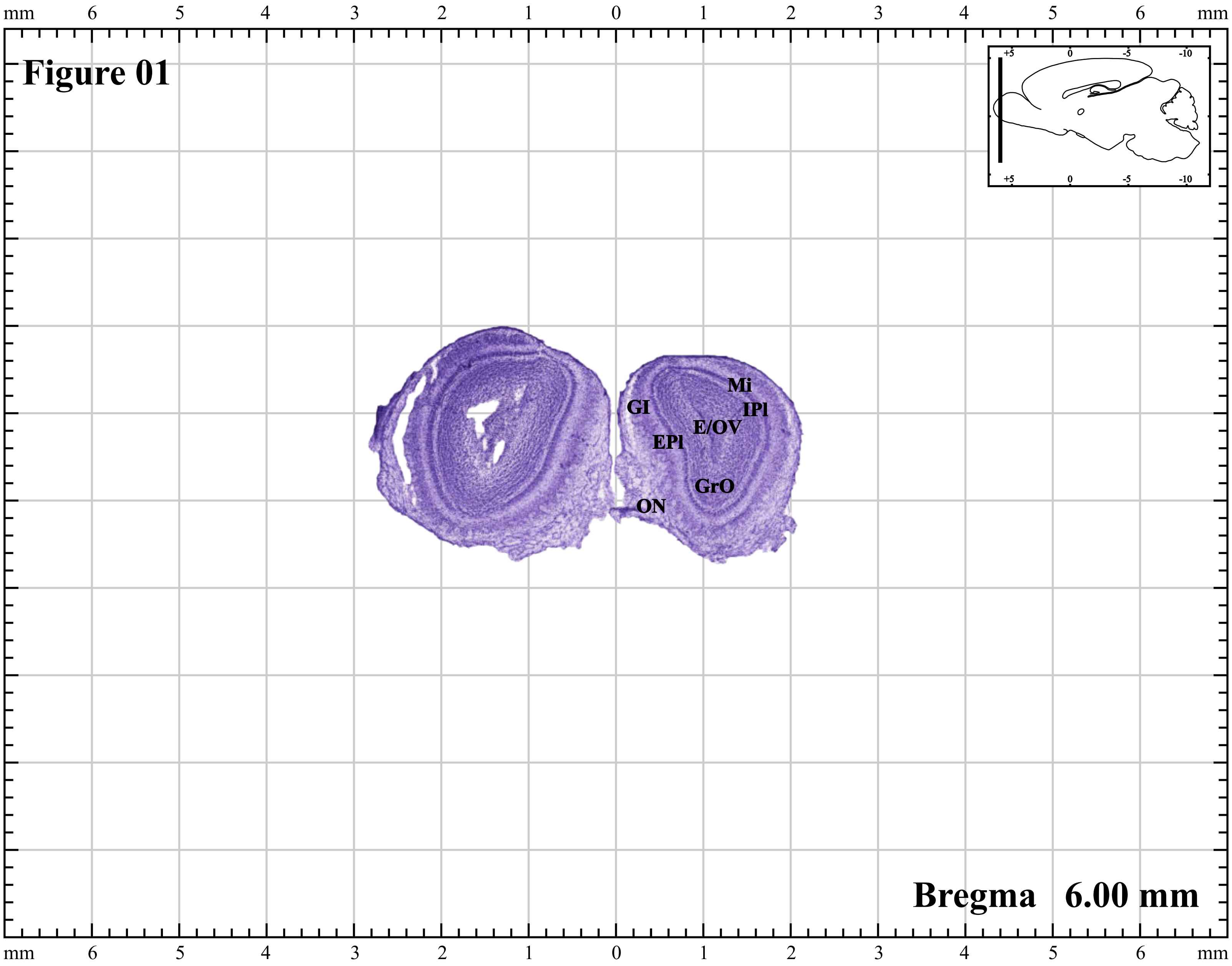

EPl external plexiform layer of the olfactory bulb

E/OV ependymal and subendymal layer/olfactory ventricle

GrO granular cell layer of the olfactory bulb

GI granular insular cortex

IPl internal plexiform layer of the olfactory bulb

Mi mitral cell layer of the olfactory bulb

ON olfactory nerve layer

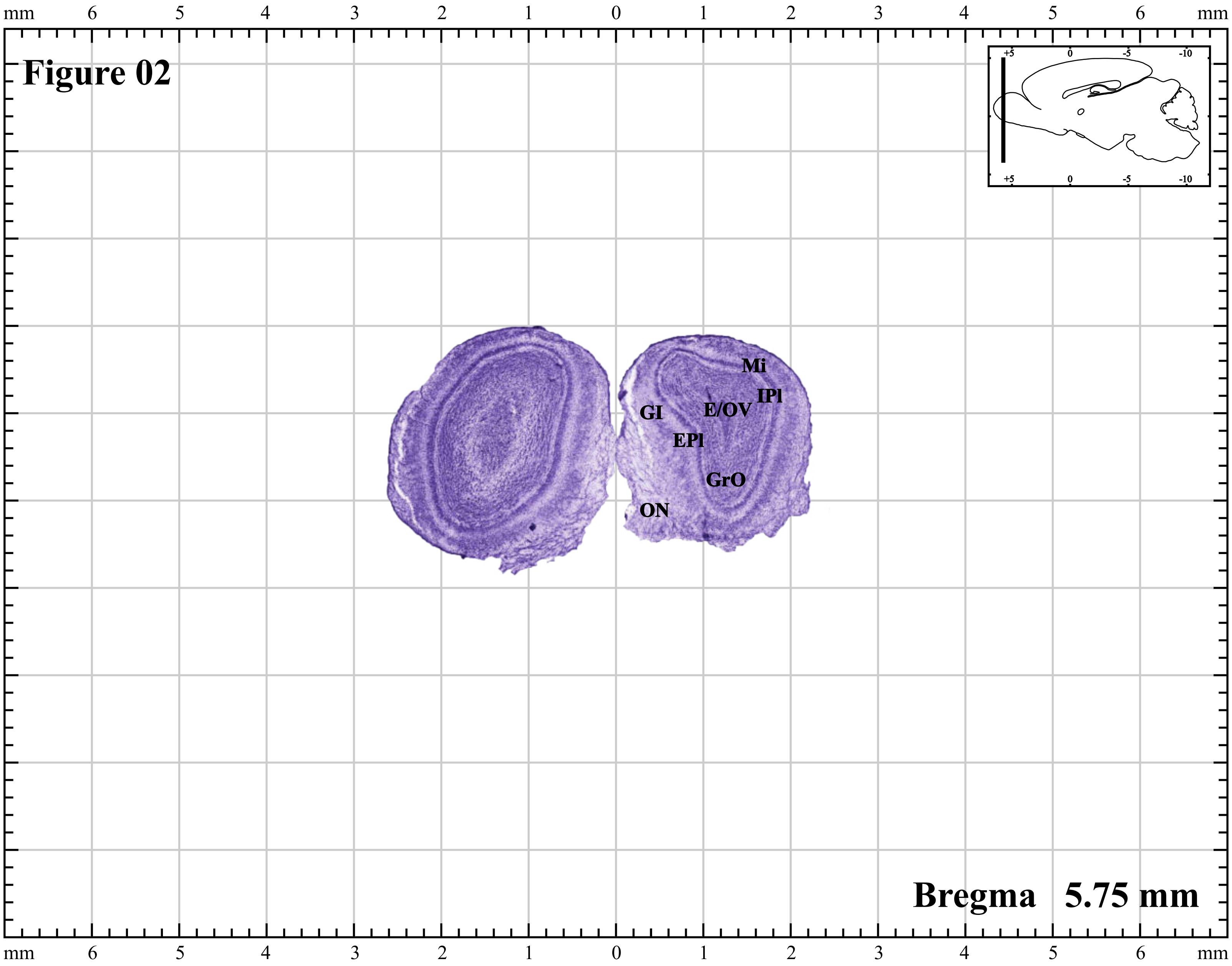

EPl external plexiform layer of the olfactory bulb

E/OV ependymal and subendymal layer/olfactory ventricle

GrO granular cell layer of the olfactory bulb

GI granular insular cortex

IPl internal plexiform layer of the olfactory bulb

Mi mitral cell layer of the olfactory bulb

ON olfactory nerve layer

Figure 03

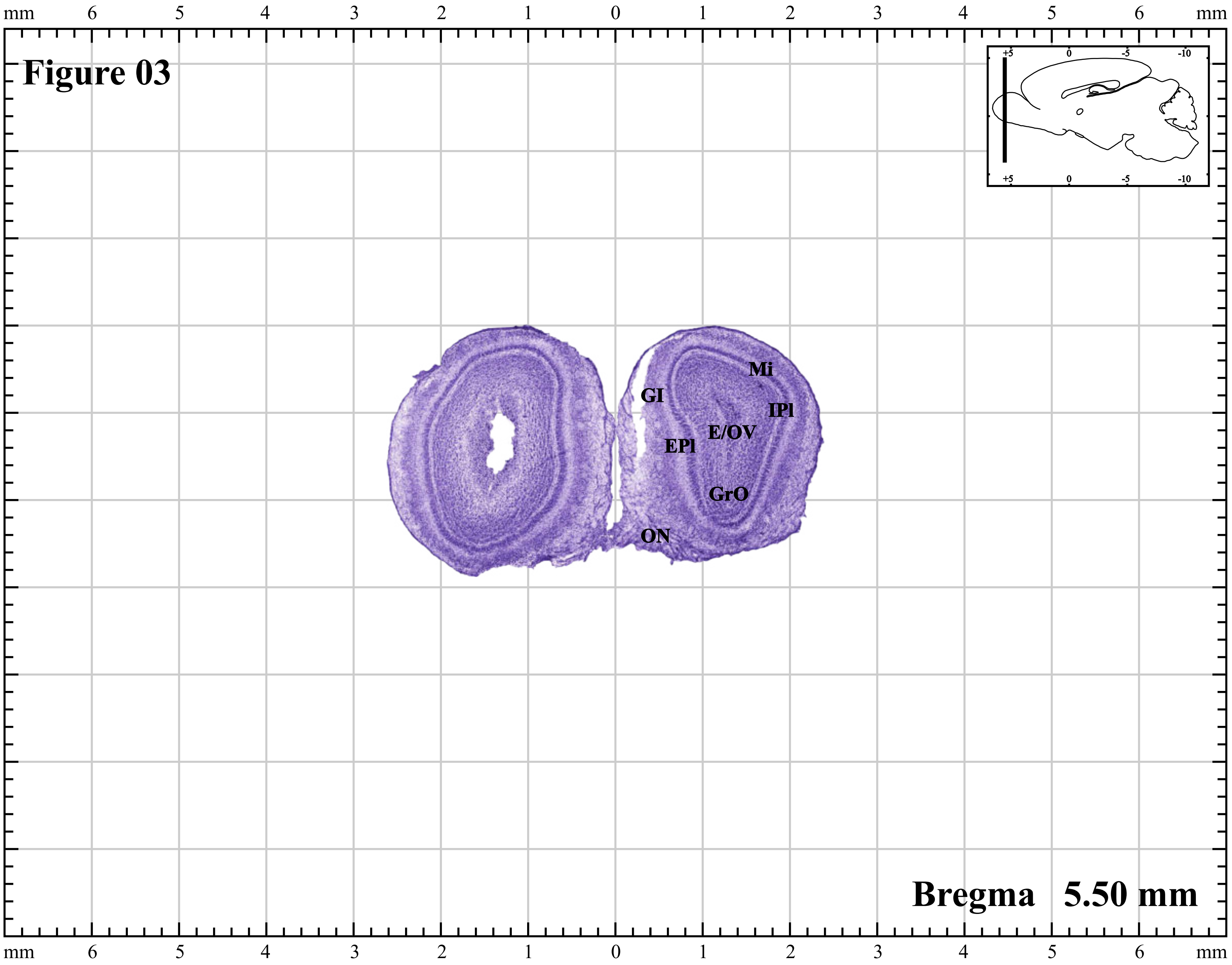

EPl external plexiform layer of the olfactory bulb
E/OV ependymal and subendymal layer/olfactory ventricle
GrO granular cell layer of the olfactory bulb
GI granular insular cortex
IPl internal plexiform layer of the olfactory bulb
Mi mitral cell layer of the olfactory bulb
ON olfactory nerve layer

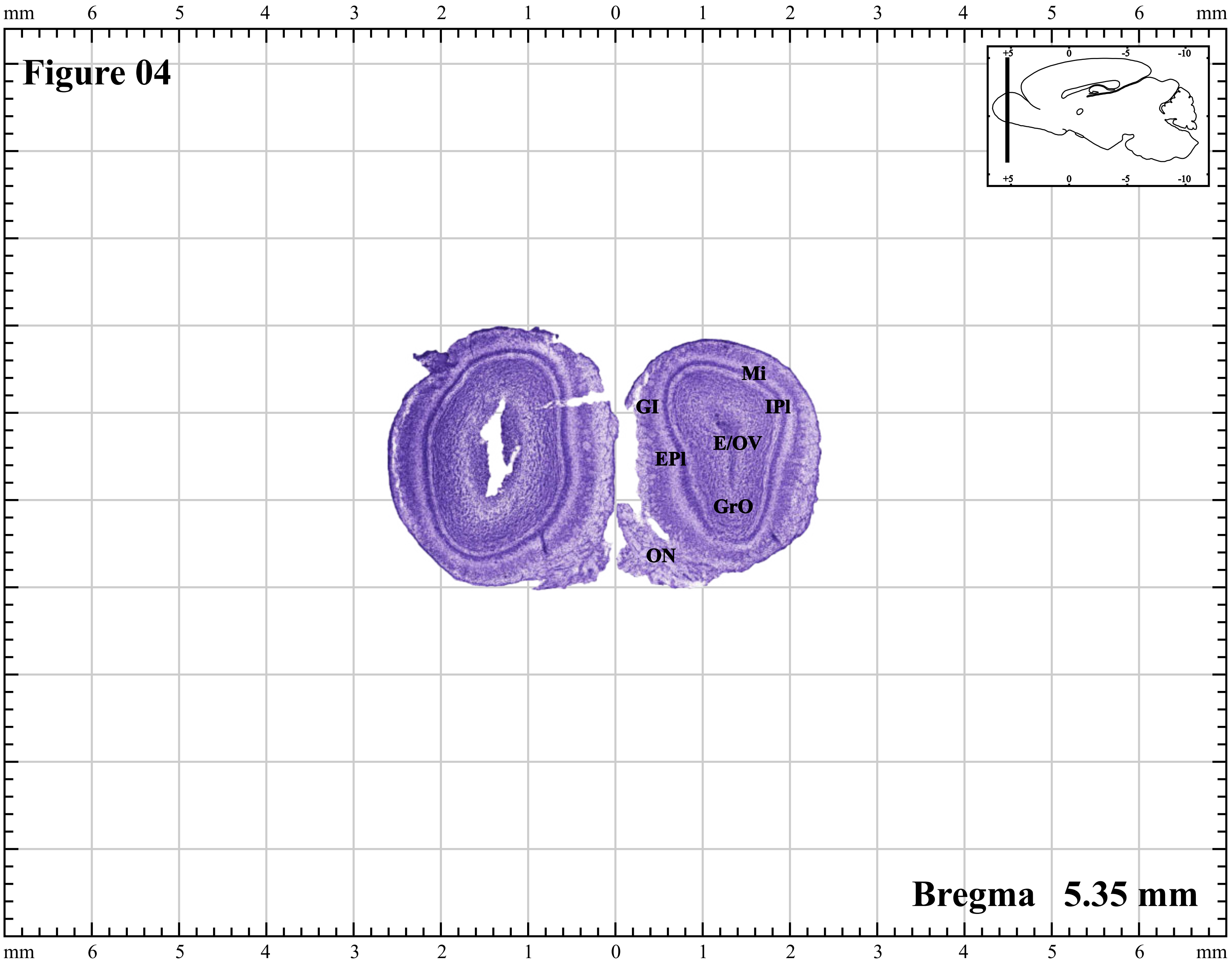

EPl external plexiform layer of the olfactory bulb
E/OV ependymal and subendymal layer/olfactory ventricle
GrO granular cell layer of the olfactory bulb
GI granular insular cortex
IPl internal plexiform layer of the olfactory bulb
Mi mitral cell layer of the olfactory bulb
ON olfactory nerve layer

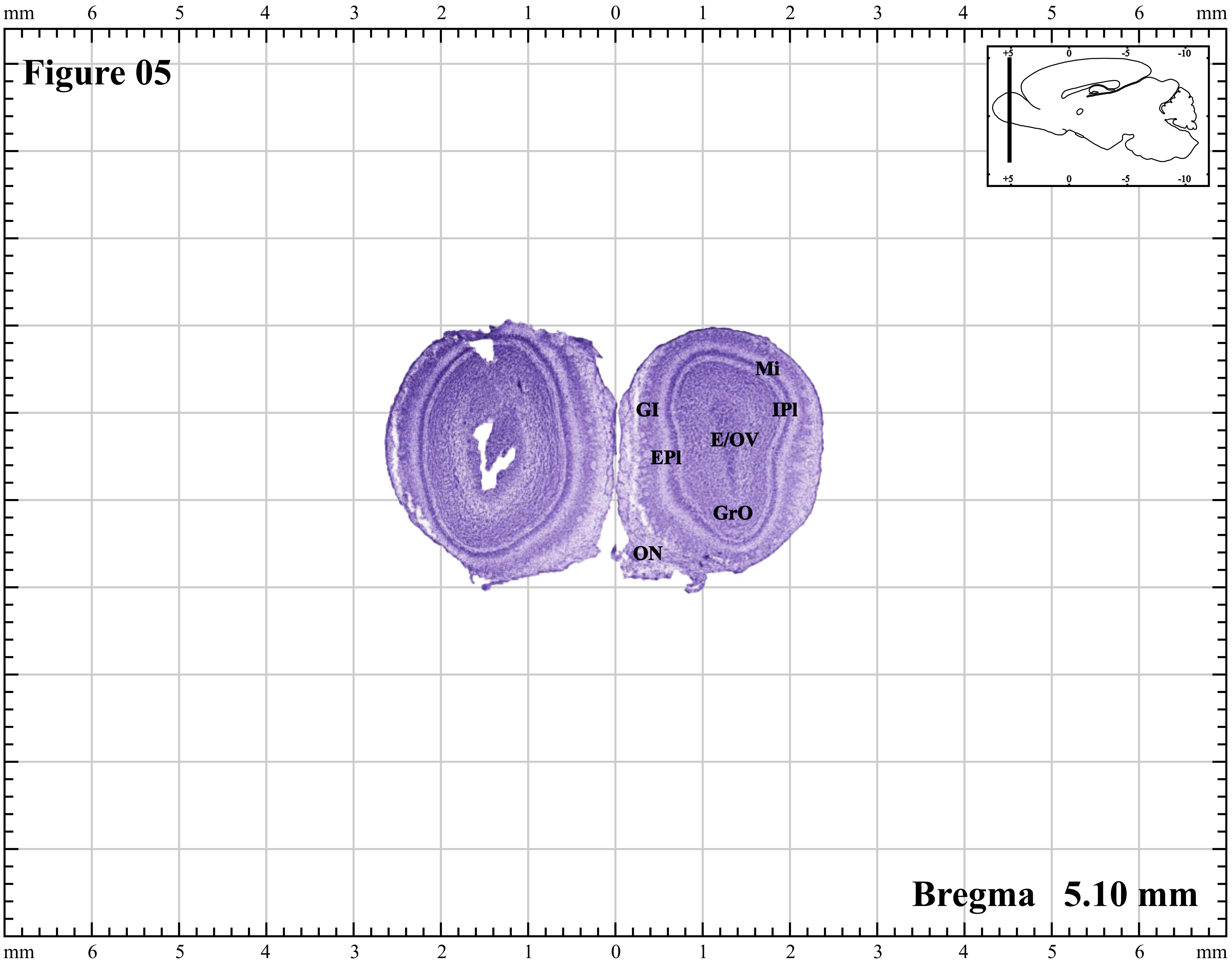

EPl external plexiform layer of the olfactory bulb
E/OV ependymal and subendymal layer/olfactory ventricle
GrO granular cell layer of the olfactory bulb
GI granular insular cortex
IPl internal plexiform layer of the olfactory bulb
Mi mitral cell layer of the olfactory bulb
ON olfactory nerve layer

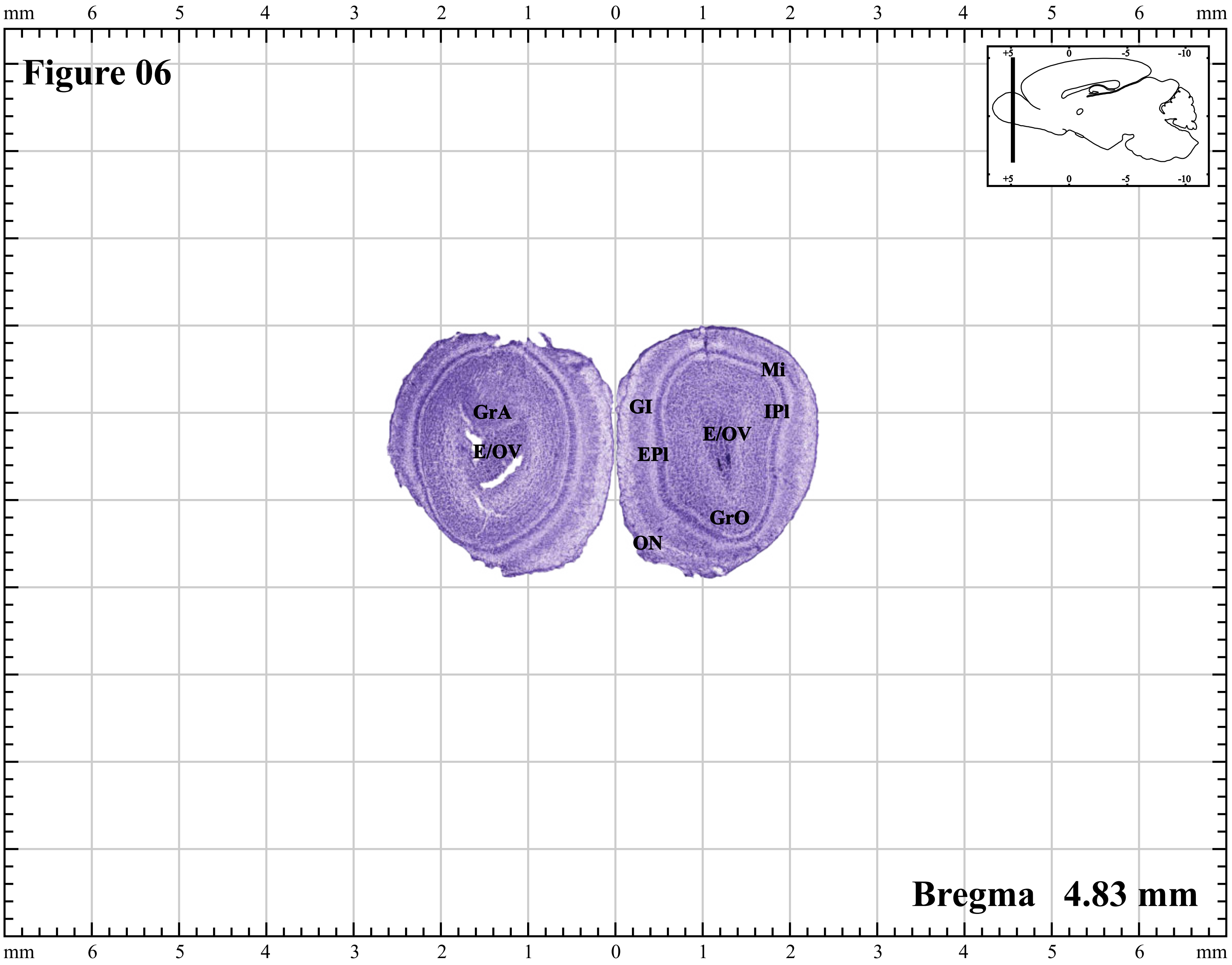

E/OV ependymal and subendymal layer/olfactory ventricle
EPl external plexiform layer of the olfactory bulb
GrO granular cell layer of the olfactory bulb
GI granular insular cortex
GrA granule cell layer of the accessory olfactory bulb
IPl internal plexiform layer of the olfactory bulb
Mi mitral cell layer of the olfactory bulb
ON olfactory nerve layer

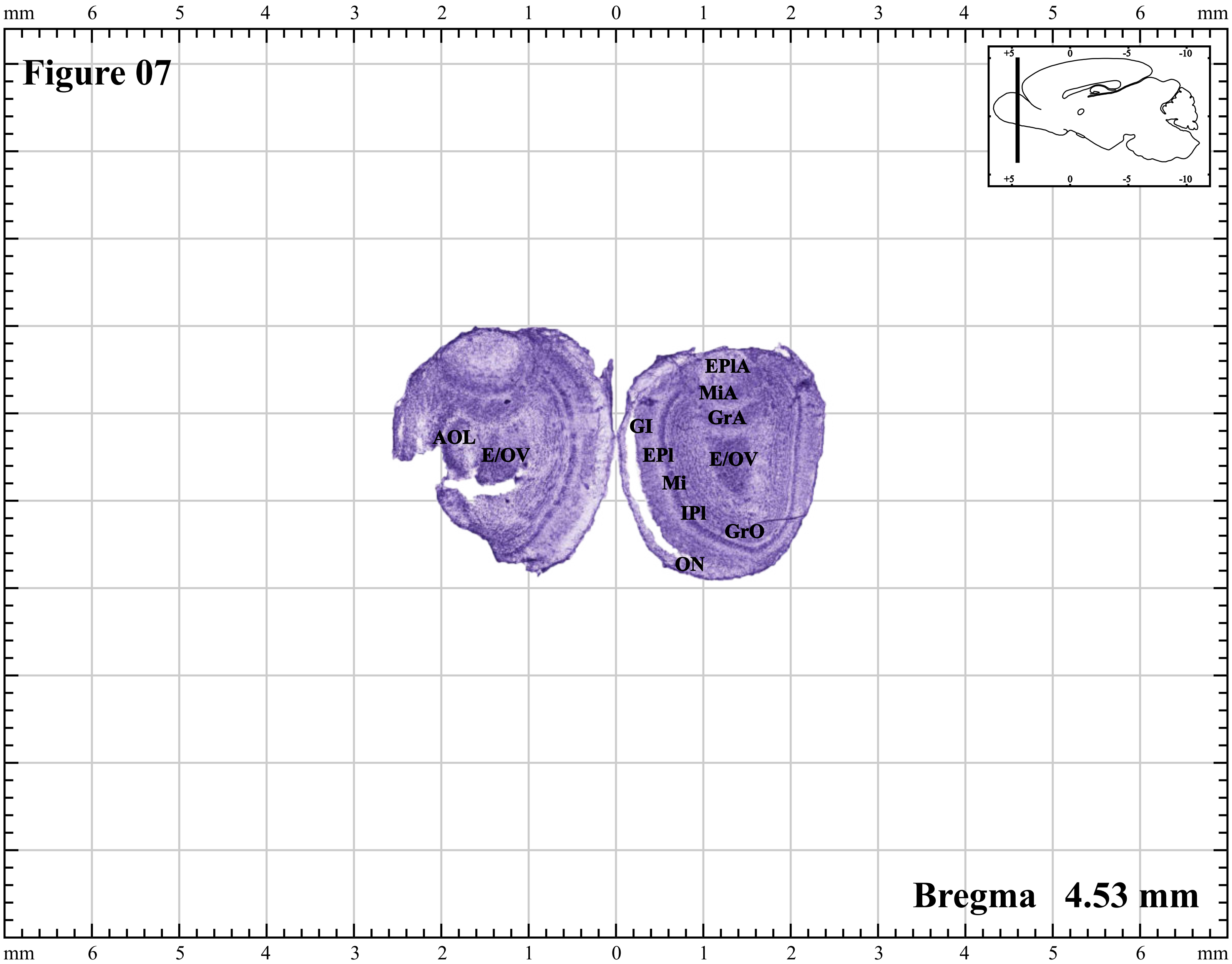

AOL anterior olfactory nucleus, lateral part

EPl external plexiform layer of the olfactory bulb

E/OV ependymal and subendymal layer/olfactory ventricle

EPlA external plexiform layer of the accessory olfactory bulb

GrO granular cell layer of the olfactory bulb

GI granular insular cortex

GrA granule cell layer of the accessory olfactory bulb

IPl internal plexiform layer of the olfactory bulb

Mi mitral cell layer of the olfactory bulb

MiA mitral cell layer of the accessory olfactory bulb

ON olfactory nerve layer

Figure 08

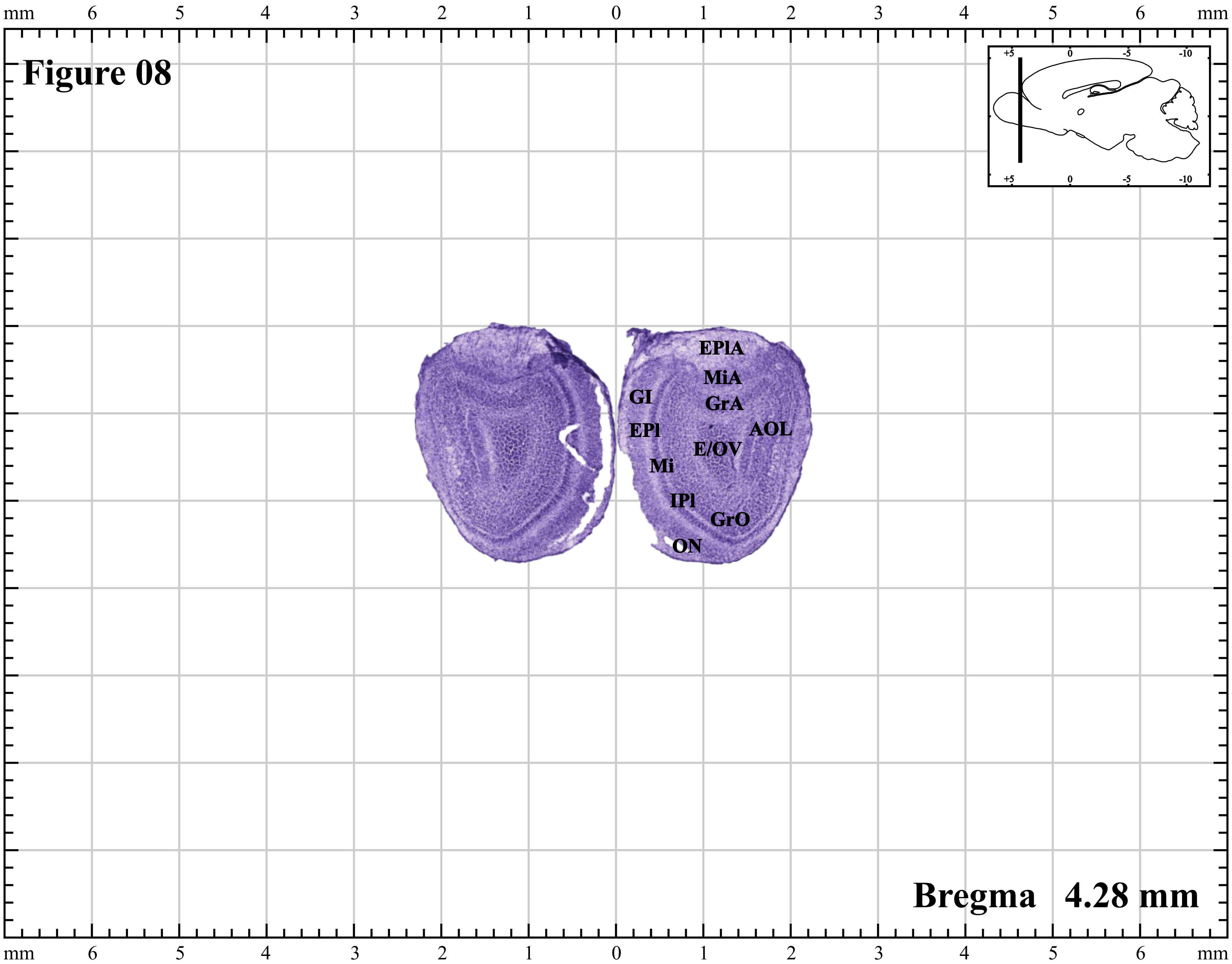

Bregma 4.28 mm

AOL anterior olfactory nucleus, lateral part
EPl external plexiform layer of the olfactory bulb
E/OV ependymal and subendymal layer/olfactory ventricle
EPlA external plexiform layer of the accessory olfactory bulb
GrO granular cell layer of the olfactory bulb
GI granular insular cortex
GrA granule cell layer of the accessory olfactory bulb
IPl internal plexiform layer of the olfactory bulb
Mi mitral cell layer of the olfactory bulb
MiA mitral cell layer of the accessory olfactory bulb
ON olfactory nerve layer

Figure 09

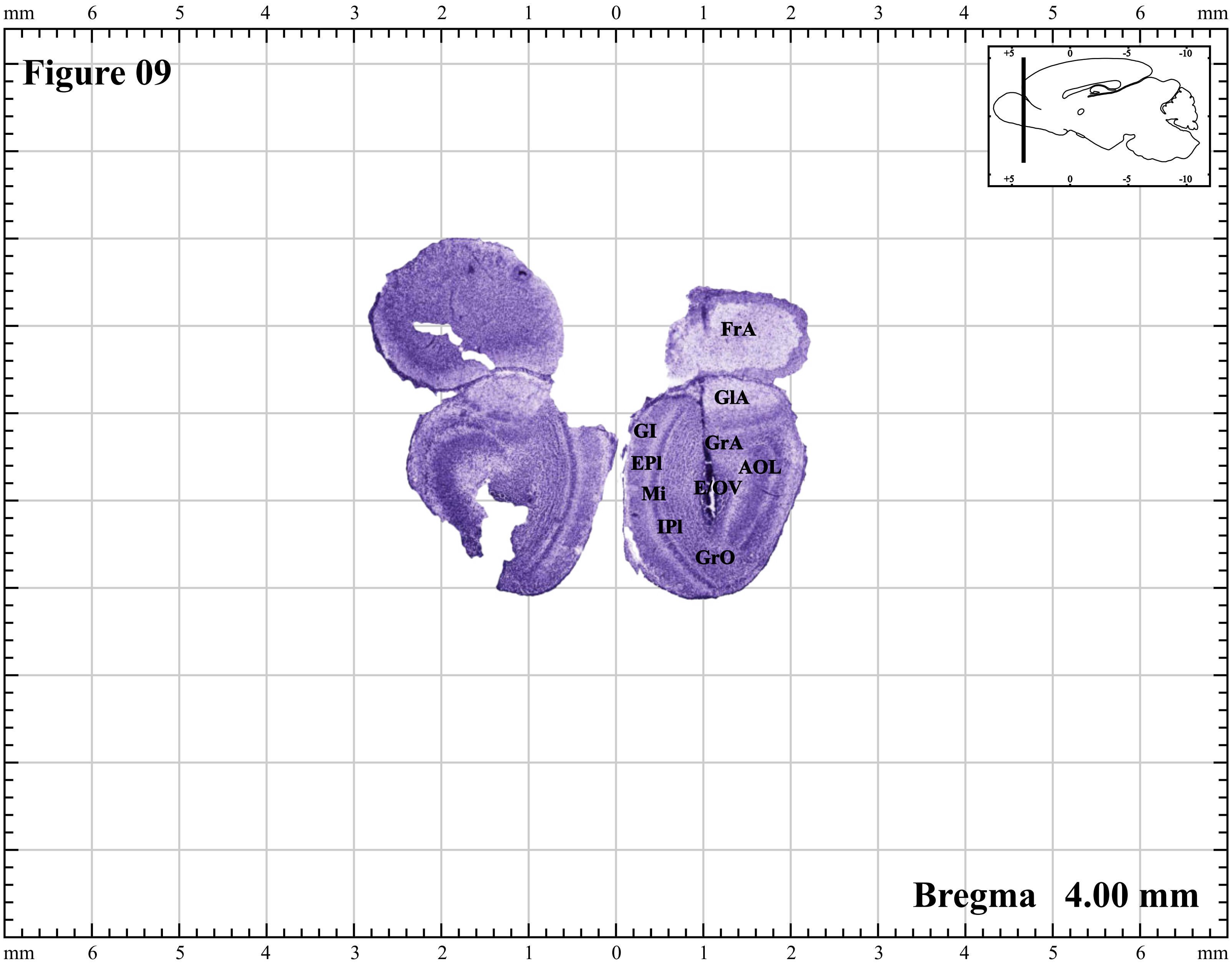

Bregma 4.00 mm

AOL anterior olfactory nucleus, lateral part
EPl external plexiform layer of the olfactory bulb
E/OV ependymal and subendymal layer/olfactory ventricle
FrA frontal assocn cortex
GlA glomerular layer of the accessory olfactory bulb
GrO granular cell layer of the olfactory bulb
GI granular insular cortex
GrA granule cell layer of the accessory olfactory bulb
IPl internal plexiform layer of the olfactory bulb
Mi mitral cell layer of the olfactory bulb

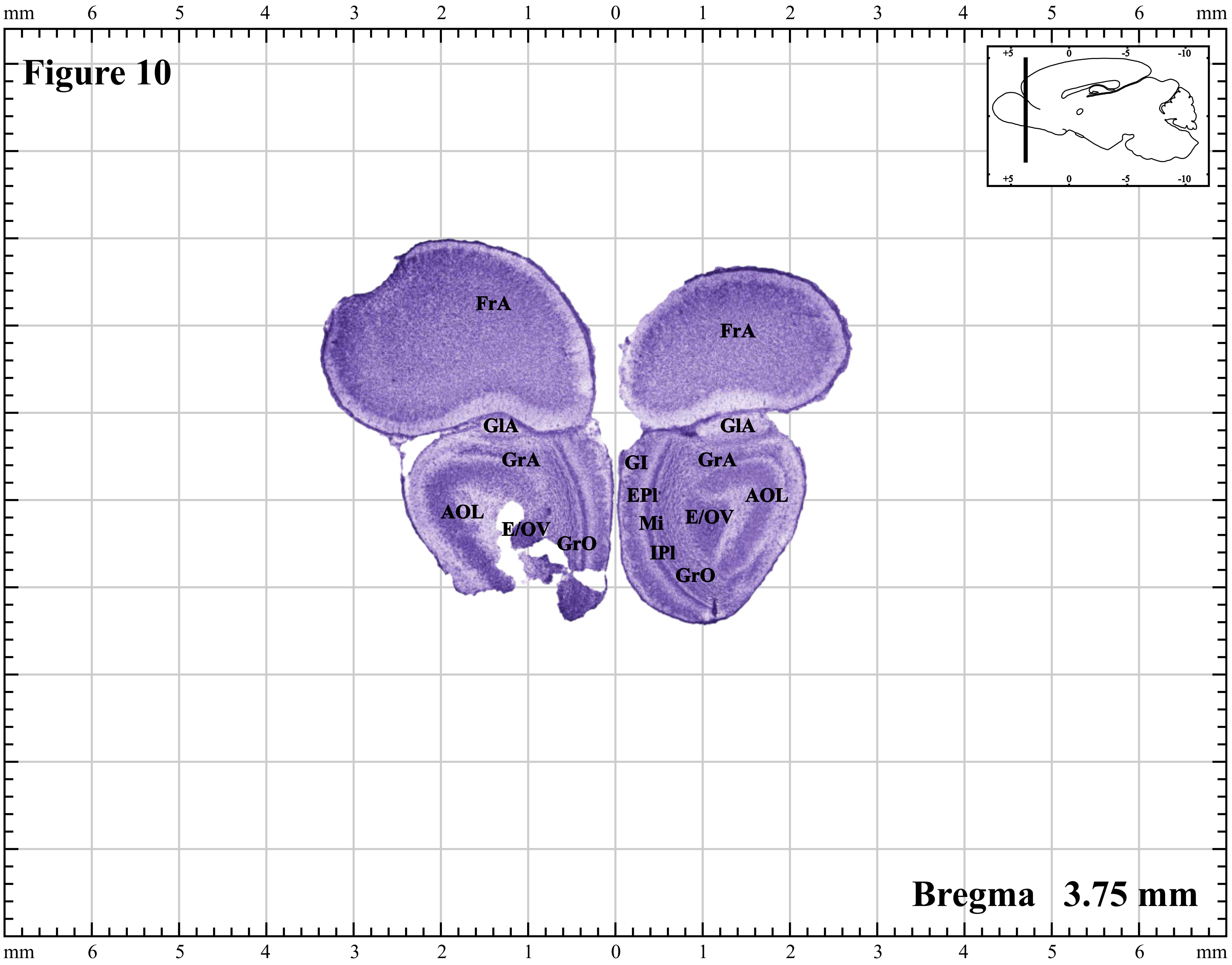

AOL anterior olfactory nucleus, lateral part

EPl external plexiform layer of the olfactory bulb

E/OV ependymal and subendymal layer/olfactory ventricle

FrA frontal assocn cortex

GlA glomerular layer of the accessory olfactory bulb

GrO granular cell layer of the olfactory bulb

GI granular insular cortex

GrA granule cell layer of the accessory olfactory bulb

IPl internal plexiform layer of the olfactory bulb

Mi mitral cell layer of the olfactory bulb

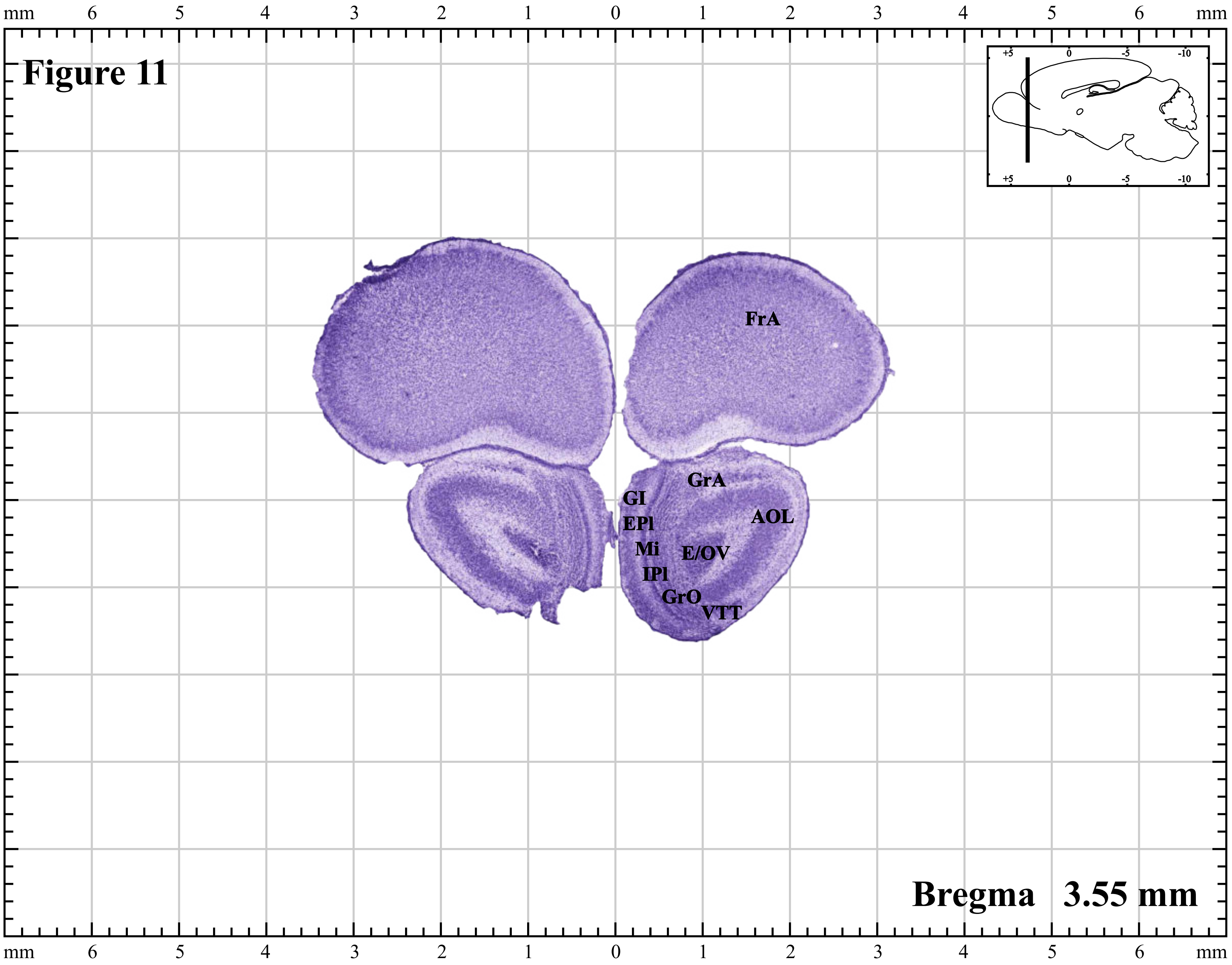

AOL anterior olfactory nucleus, lateral part
EPl external plexiform layer of the olfactory bulb
E/OV ependymal and subendymal layer/olfactory ventricle
FrA frontal assocn cortex
GrA granule cell layer of the accessory olfactory bulb
GrO granular cell layer of the olfactory bulb
GI granular insular cortex
GrA granule cell layer of the accessory olfactory bulb
IPl internal plexiform layer of the olfactory bulb
Mi mitral cell layer of the olfactory bulb
VTT ventral tenia tecta

Figure 12

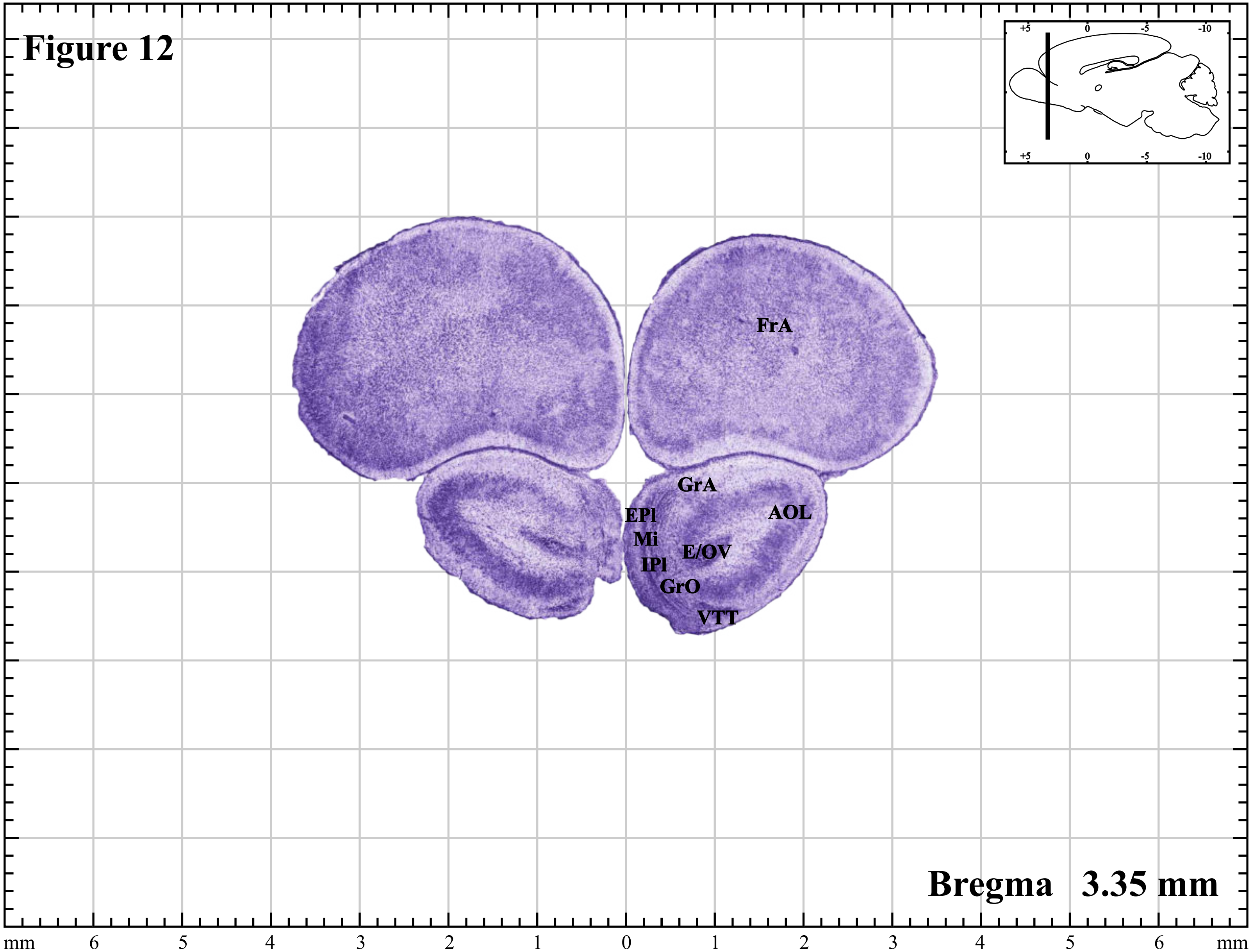

Bregma 3.35 mm

AOL anterior olfactory nucleus, lateral part
EPl external plexiform layer of the olfactory bulb
E/OV ependymal and subendymal layer/olfactory ventricle
FrA frontal assocn cortex
GrA granule cell layer of the accessory olfactory bulb
GrO granular cell layer of the olfactory bulb
IPl internal plexiform layer of the olfactory bulb
Mi mitral cell layer of the olfactory bulb
VTT ventral tenia tecta

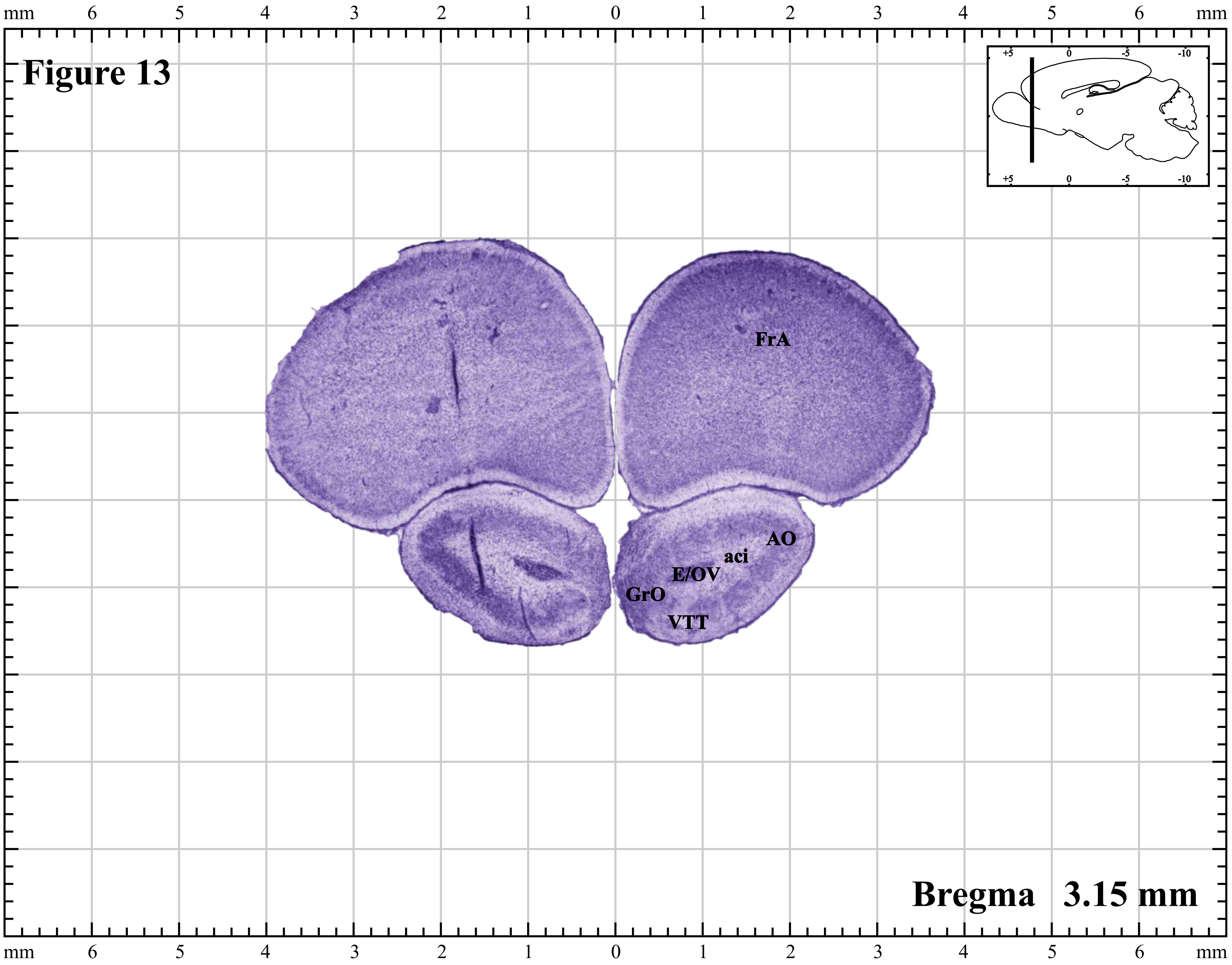

aci anterior commissure, intrabulbar part

AO anterior olfactory nuclues

E/OV ependymal and subendymal layer/
olfactory ventricle

FrA frontal assocn cortex

GrO granular cell layer of
the olfactory bulb

VTT ventral tenia tecta

Figure 14

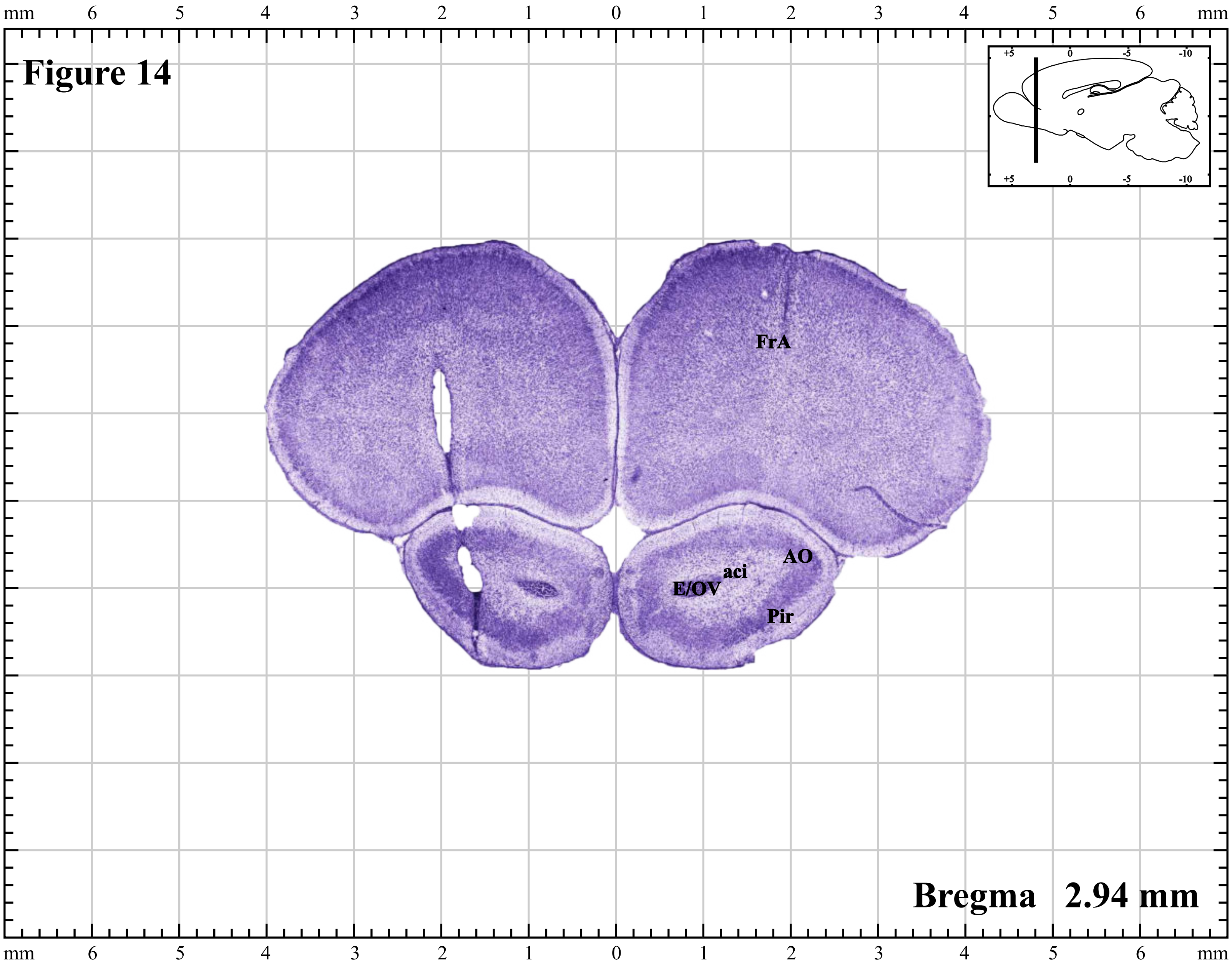

Bregma 2.94 mm

aci anterior commissure, intrabulbar part
AO anterior olfactory nuclues
E/OV ependymal and subendymal layer/ olfactory ventricle
GrO granular cell layer of the olfactory bulb
FrA frontal assocn cortex
Pir piriform cortex

Figure 15

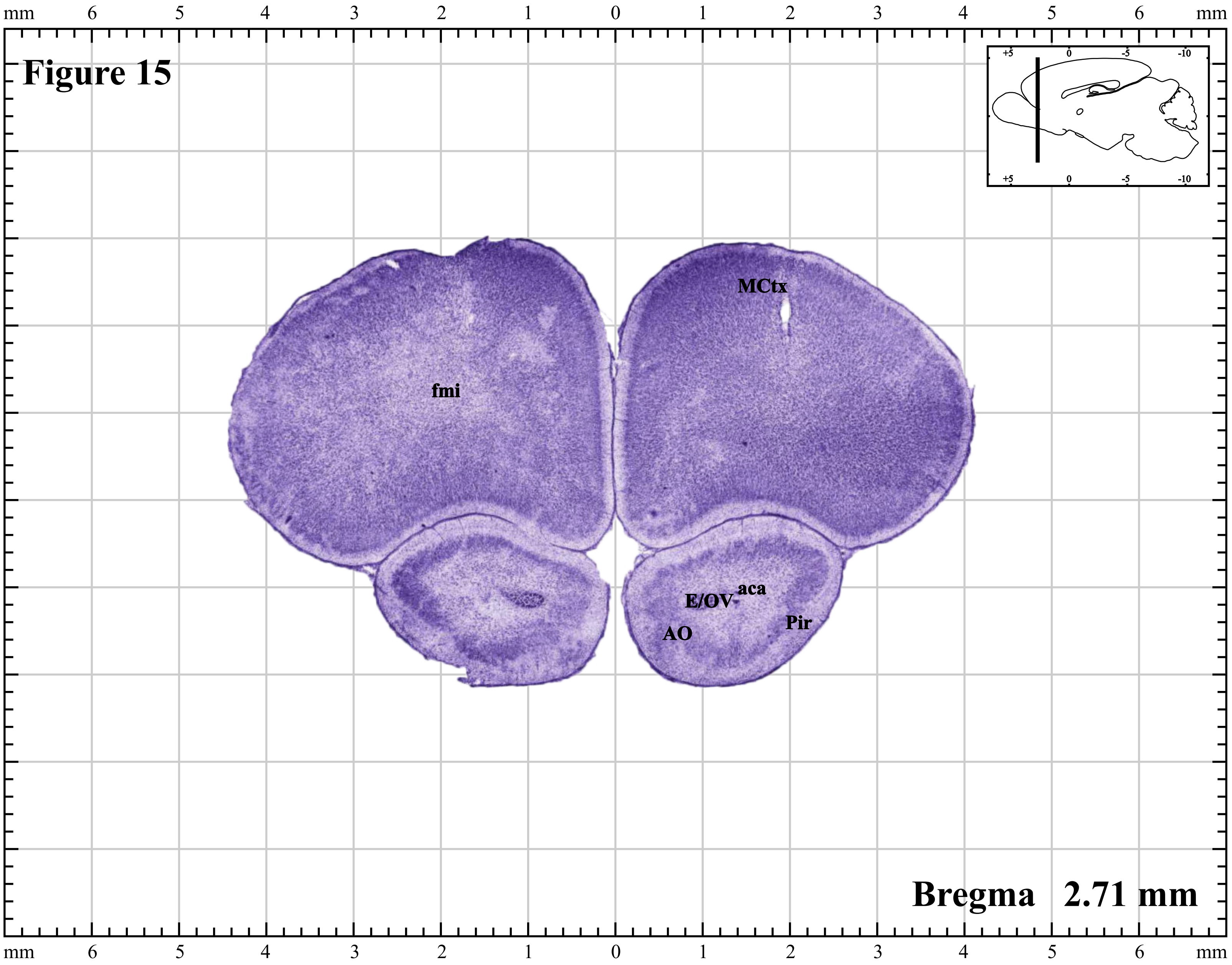

aca anterior commissure, anterior part
AO anterior olfactory nuclues
E/OV ependyma and subependymal layer /olfactory ventricle
fmi forceps major of corpus callosum
MCtx motor cortex
Pir piriform cortex

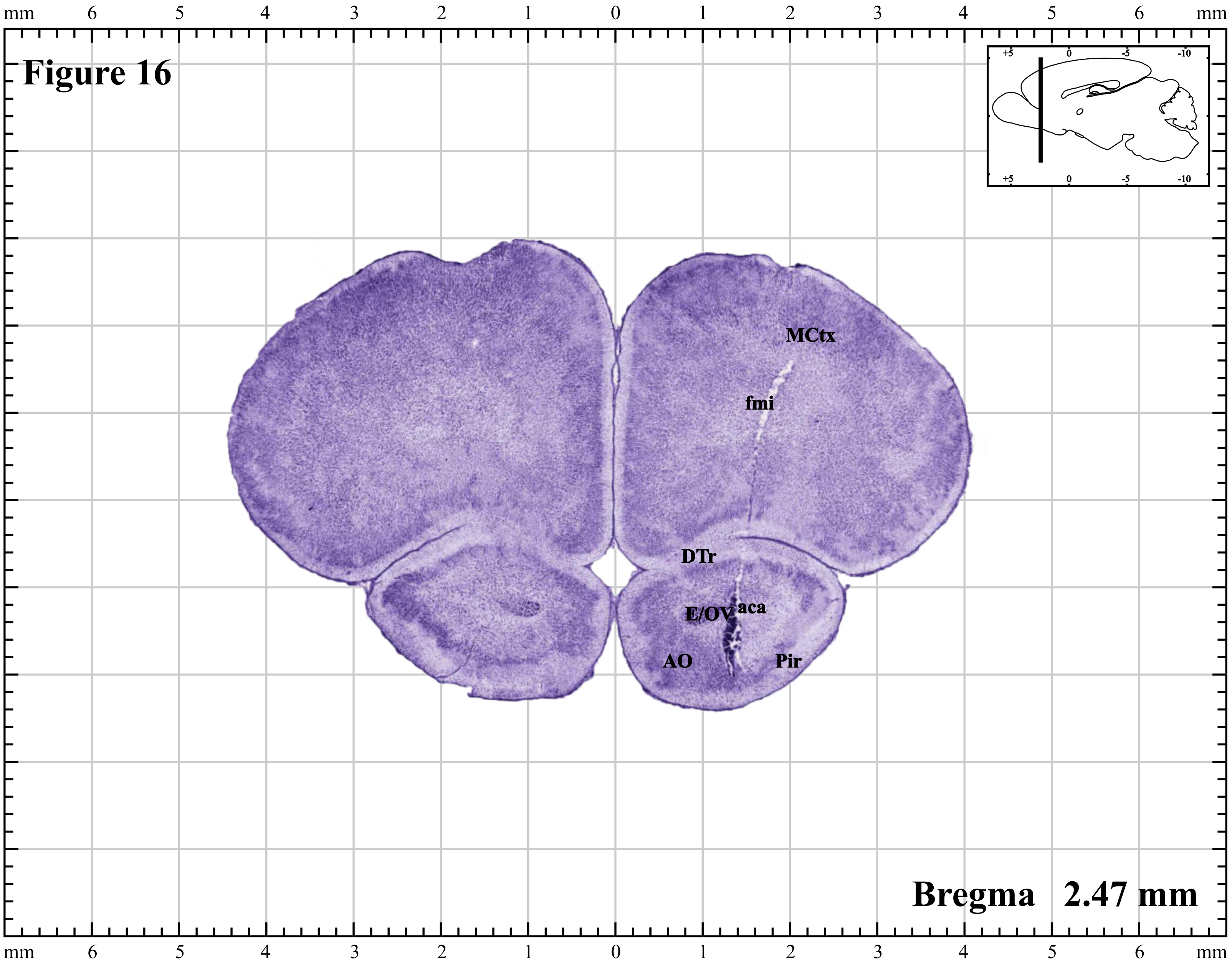

aca anterior commissure, anterior part
AO anterior olfactory nuclues
DTr dorsal transition zone
E/OV ependyma and subependymal layer
/olfactory ventricle
fmi forceps major of corpus callosum
MCtx motor cortex
Pir piriform cortex

Figure 17

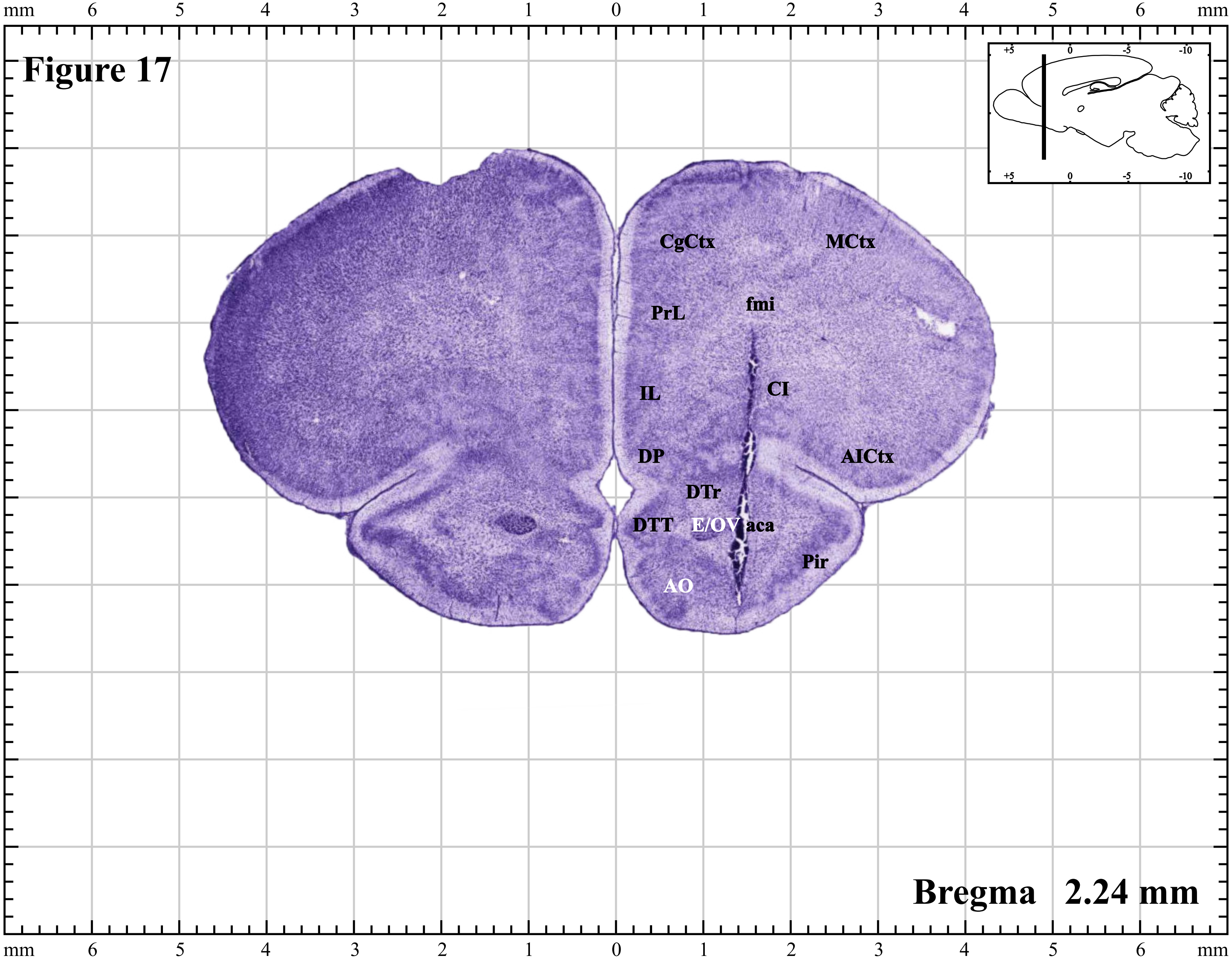

Bregma 2.24 mm

aca anterior commissure, anterior part
AO anterior olfactory nuclues
AICtx agranular insular cortex
CgCtx cingulate cortex
CI claustrum
DP dorsal peduncular cortex
DTT dorsal tenia tecta
DTr dorsal transition zone
E/OV ependyma and subependymal layer /olfactory ventricle
IL infralimbic cortex
fmi forceps major of corpus callosum
MCtx motor cortex
Pir piriform cortex
PrL prelimbic cortex

Figure 18

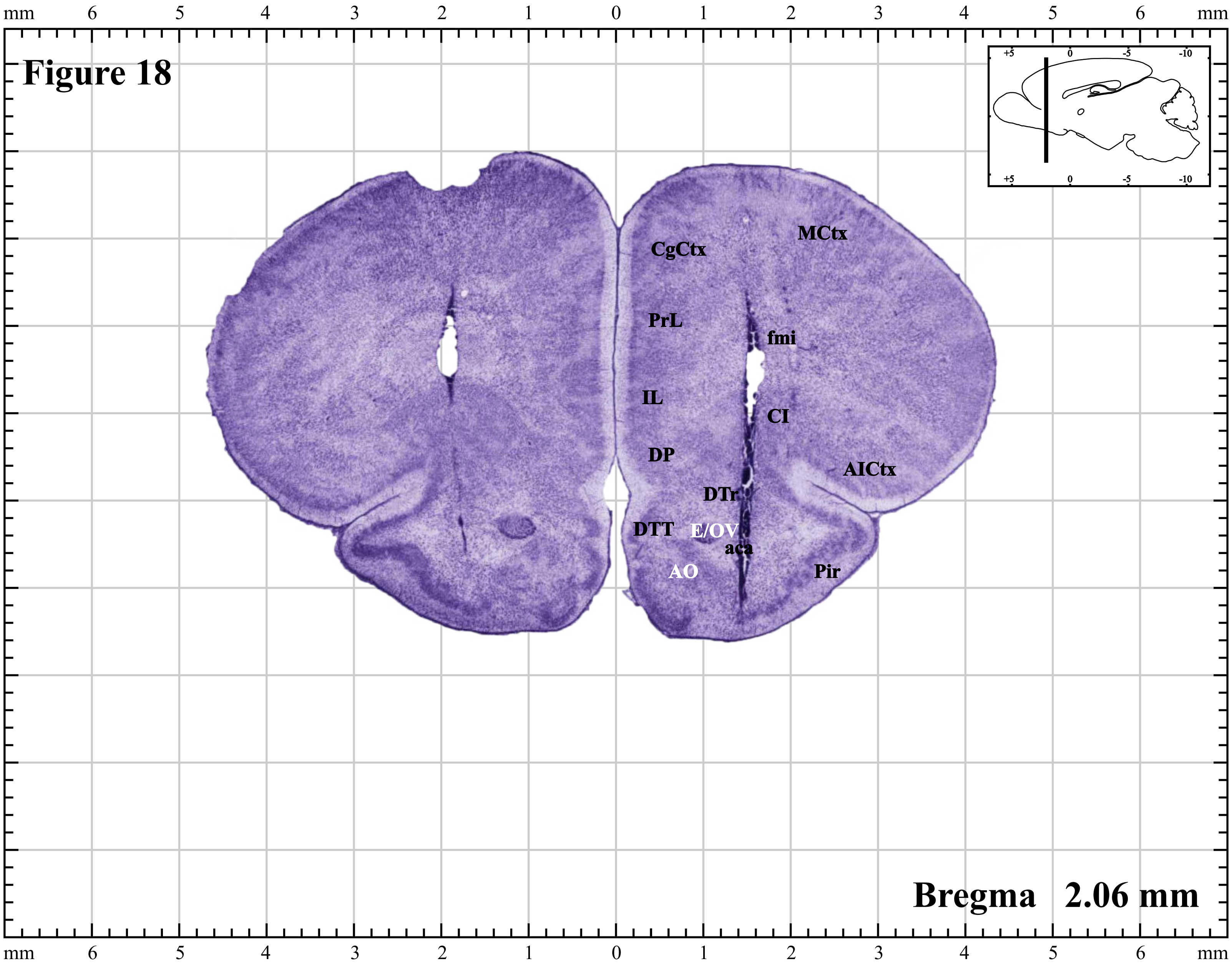

Bregma 2.06 mm

aca anterior commissure, anterior part
AO anterior olfactory nuclues
AICtx agranular insular cortex
CgCtx cingulate cortex
CI claustrum
DTT dorsal tenia tecta
DP dorsal peduncular cortex
DTr dorsal transition zone
E/OV ependyma and subependymal layer /olfactory ventricle
IL infralimbic cortex
fmi forceps major of corpus callosum
MCtx motor cortex
Pir piriform cortex
PrL prelimbic cortex

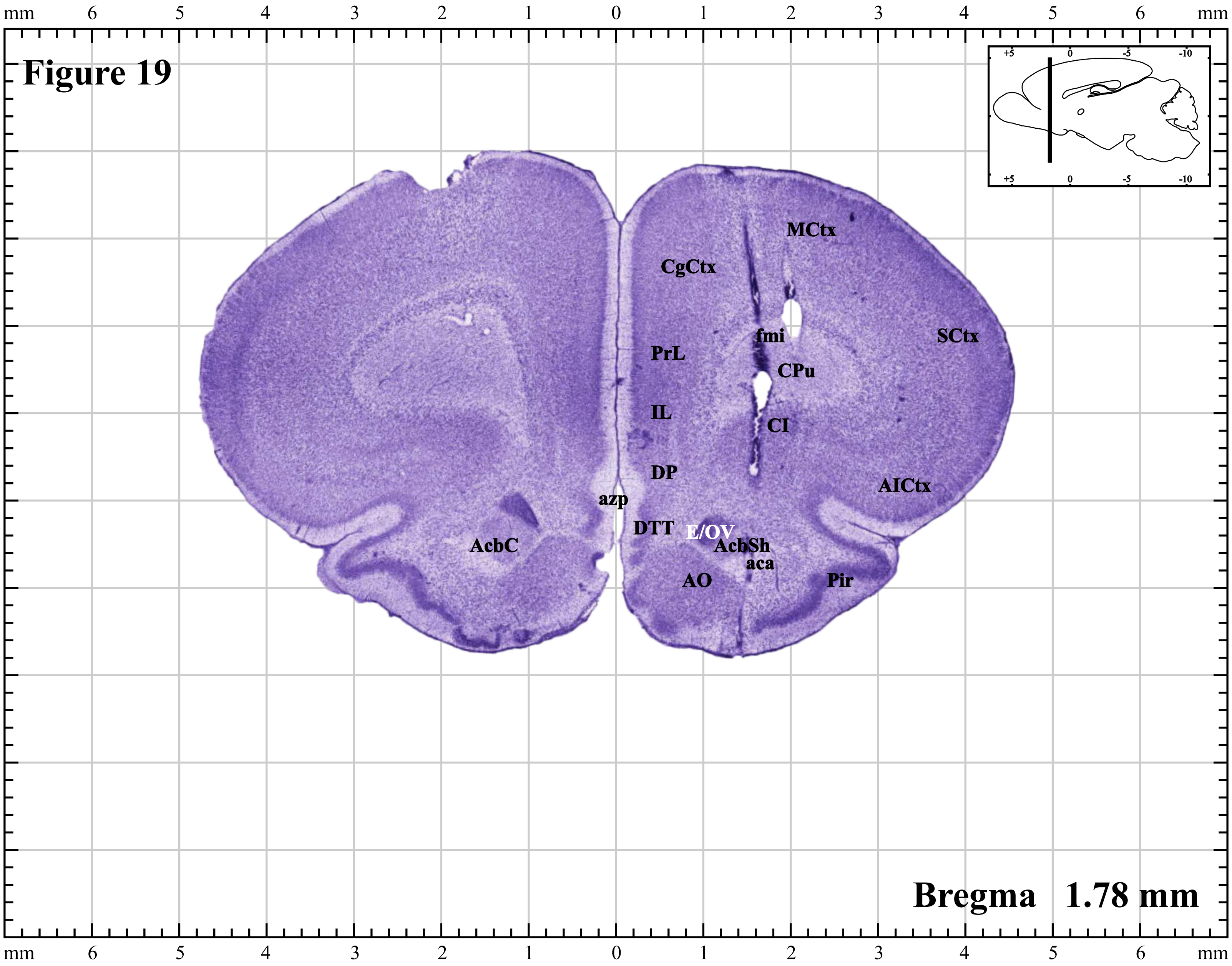

Figure 19

Bregma 1.78 mm

- **azp** azygous pericallosal artery
- **aca** anterior commissure, anterior part
- **AcbC** accumbens nucleus, core
- **AcbSh** accumbens shell
- **AO** anterior olfactory nuclues
- **AICtx** agranular insular cortex
- **CgCtx** cingulate cortex
- **CI** claustrum
- **CPu** caudate putamen (striatum)

- **DP** dorsal peduncular cortex
- **DTT** dorsal tenia tecta
- **IL** infralimbic cortex
- **OV** olfactory ventricle
- **fmi** forceps major of corpus callosum
- **MCtx** motor cortex
- **Pir** piriform cortex
- **PrL** prelimbic cortex
- **SCtx** somatosensory cortex

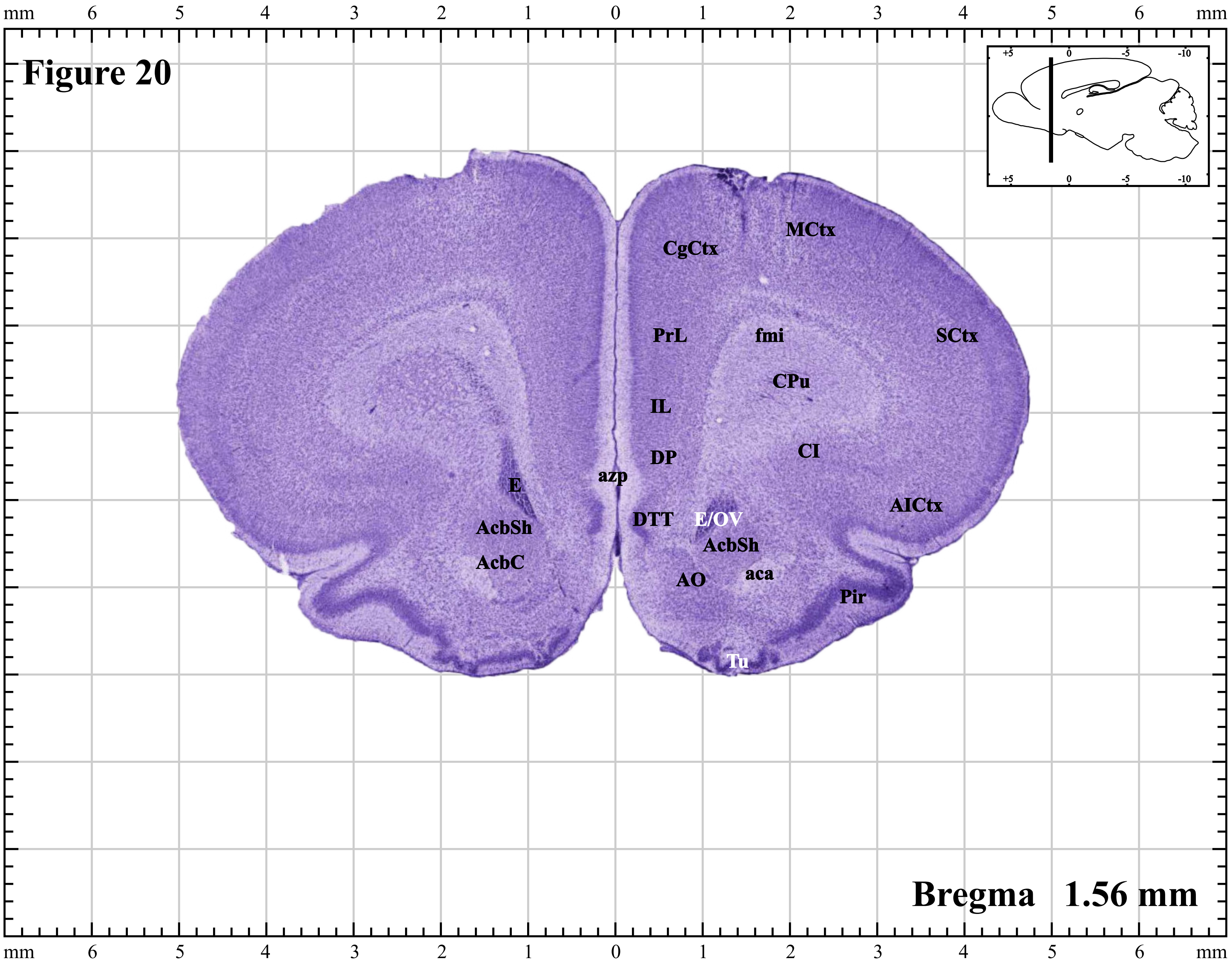

azp azygous pericallosal artery
aca anterior commissure, anterior part
AcbC accumbens nucleus, core
AcbSh accumbens shell
AO anterior olfactory nuclues
AICtx agranular insular cortex
CgCtx cingulate cortex
CI claustrum
CPu caudate putamen (striatum)
DP dorsal peduncular cortex
DTT dorsal tenia tecta
IL infralimbic cortex
E/OV ependyma and subependymal layer /olfactory ventricle
fmi forceps major of corpus callosum
MCtx motor cortex
Pir piriform cortex
PrL prelimbic cortex
SCtx somatosensory cortex
Tu olfactory tubercle

Figure 21

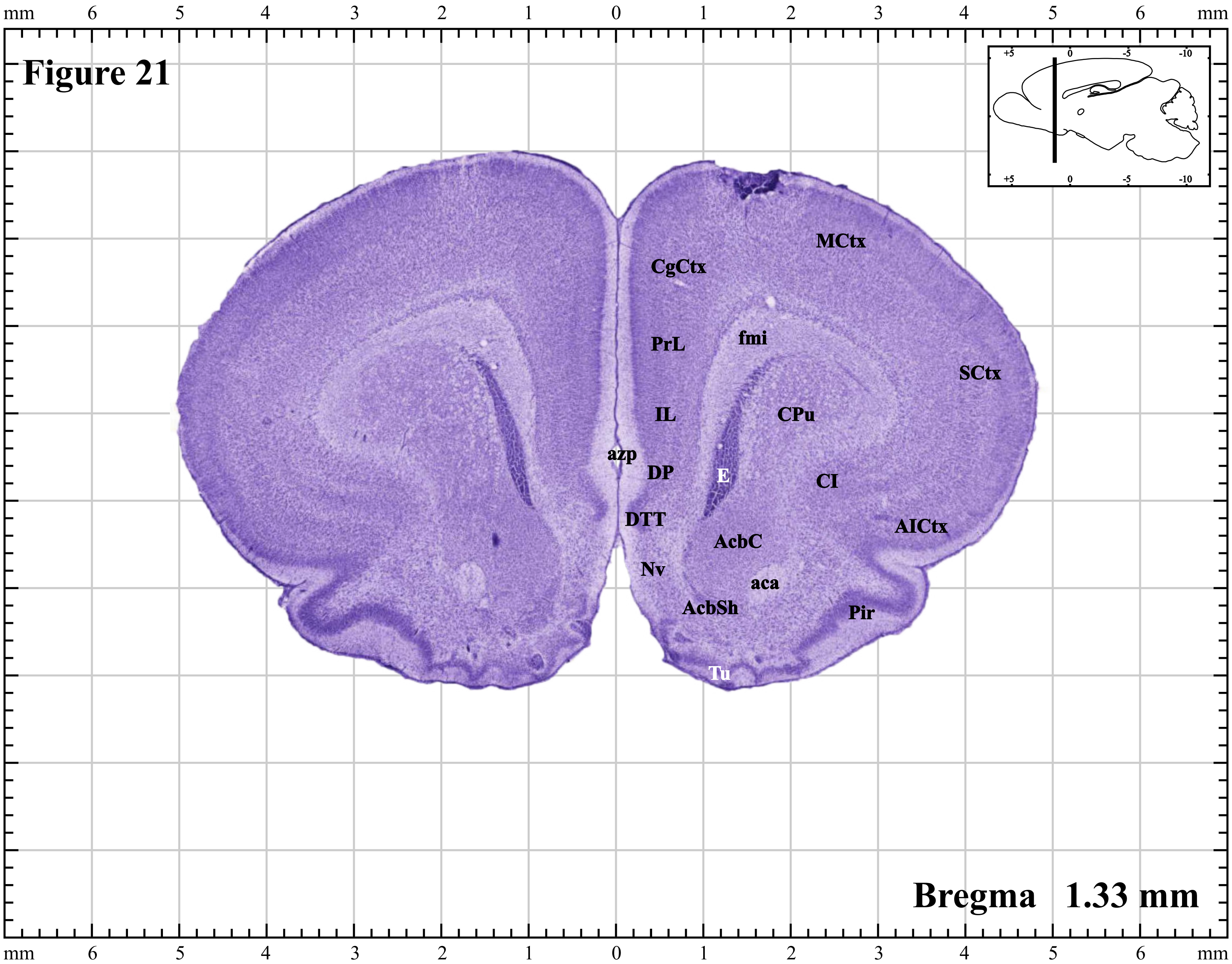

Bregma 1.33 mm

azp azygous pericallosal artery
aca anterior commissure, anterior part
AcbC accumbens nucleus, core
AcbSh accumbens shell
AICtx agranular insular cortex
CgCtx cingulate cortex
CI claustrum
CPu caudate putamen (striatum)
DP dorsal peduncular cortex
DTT dorsal tenia tecta
IL infralimbic cortex
E ependyma and subependymal layer
fmi forceps major of corpus callosum
MCtx motor cortex
Nv navicular nucleus of the basal forebrain
Pir piriform cortex
PrL prelimbic cortex
SCtx somatosensory cortex
Tu olfactory tubercle

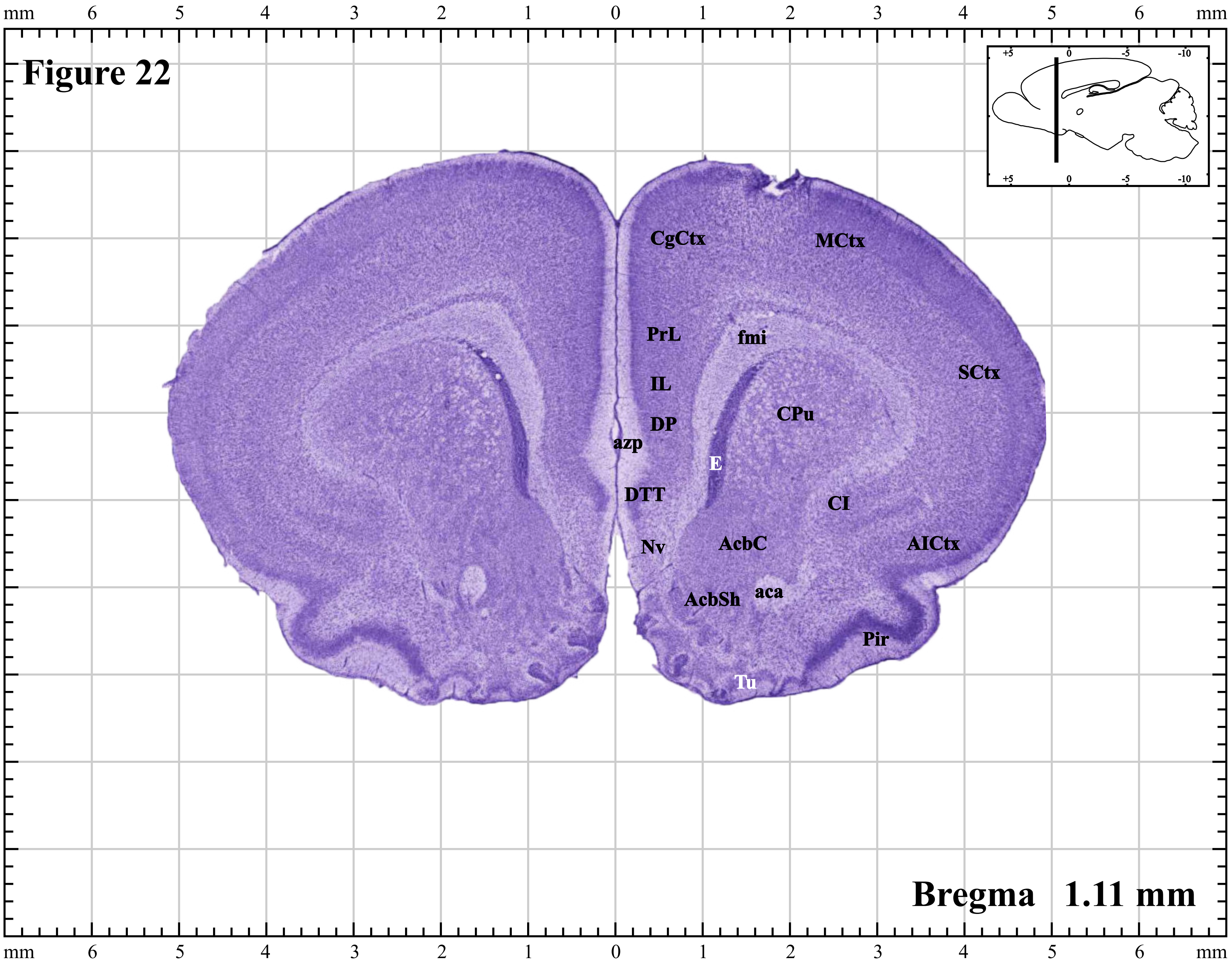

azp azygous pericallosal artery
aca anterior commissure, anterior part
AcbC accumbens nucleus, core
AcbSh accumbens shell
AICtx agranular insular cortex
CgCtx cingulate cortex
CI claustrum
CPu caudate putamen (striatum)
DP dorsal peduncular cortex
DTT dorsal tenia tecta
IL infralimbic cortex
E ependyma and subependymal layer
fmi forceps major of corpus callosum
MCtx motor cortex
Pir piriform cortex
PrL prelimbic cortex
SCtx somatosensory cortex
Nv navicular nucleus of the basal forebrain
Tu olfactory tubercle

Figure 23

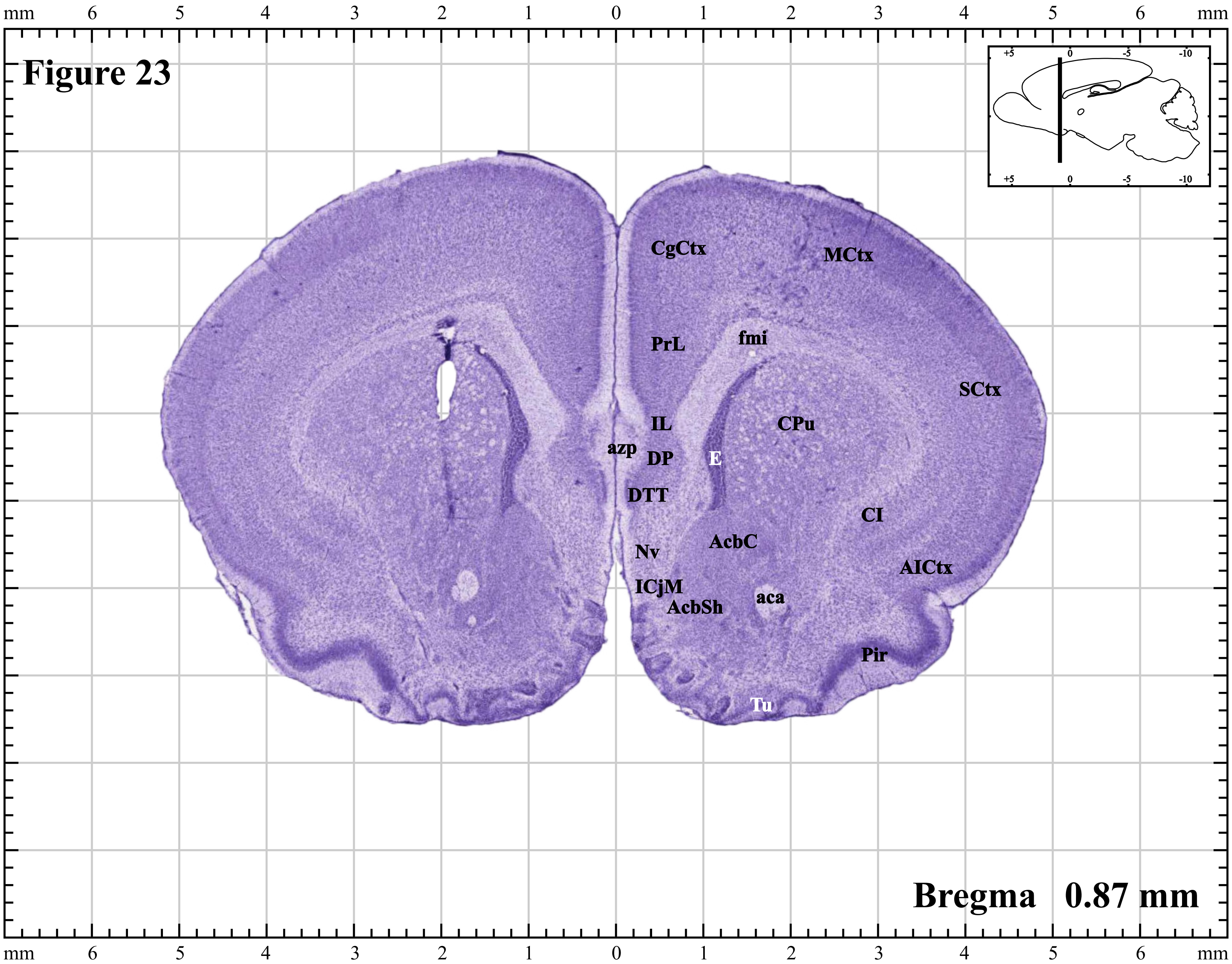

Bregma 0.87 mm

azp azygous pericallosal artery
aca anterior commissure, anterior part
AcbC accumbens nucleus, core
AcbSh accumbens shell
AICtx agranular insular cortex
CgCtx cingulate cortex
CI claustrum
CPu caudate putamen (striatum)
DP dorsal peduncular cortex
DTT dorsal tenia tecta
IL infralimbic cortex
ICjM islands of Calleja, major island
E ependyma and subependymal layer
fmi forceps major of corpus callosum
MCtx motor cortex
Nv navicular nucleus of the basal forebrain
Pir piriform cortex
PrL prelimbic cortex
SCtx somatosensory cortex
Tu olfactory tubercle

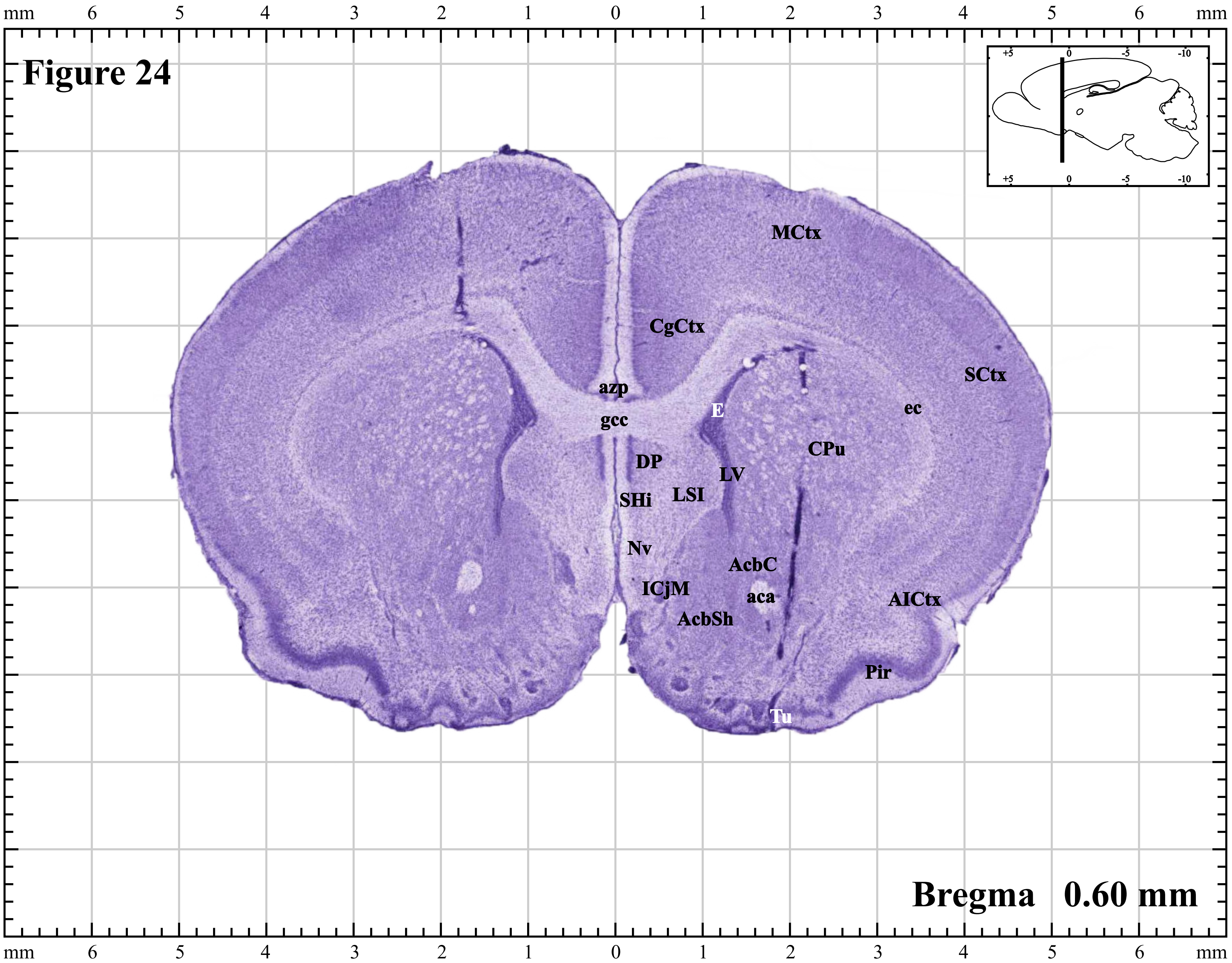

aca anterior commissure, anterior part
azp azygous pericallosal artery
AcbC accumbens nucleus, core
AcbSh accumbens shell
AICtx agranular insular cortex
CgCtx cingulate cortex
CPu caudate putamen (striatum)
DP dorsal peduncular cortex
ec external capsule
E ependyma and subependymal layer
gcc genu of the corpus callosum
ICjM islands of Calleja, major island
fmi forceps major of corpus callosum
LV lateral ventricle
LSI lateral septal nucleus, intermediate part
MCtx motor cortex
Nv navicular nucleus of the basal forebrain
Pir piriform cortex
SCtx somatosensory cortex
SHi septohippocampal nucleus
Tu olfactory tubercle

Figure 25

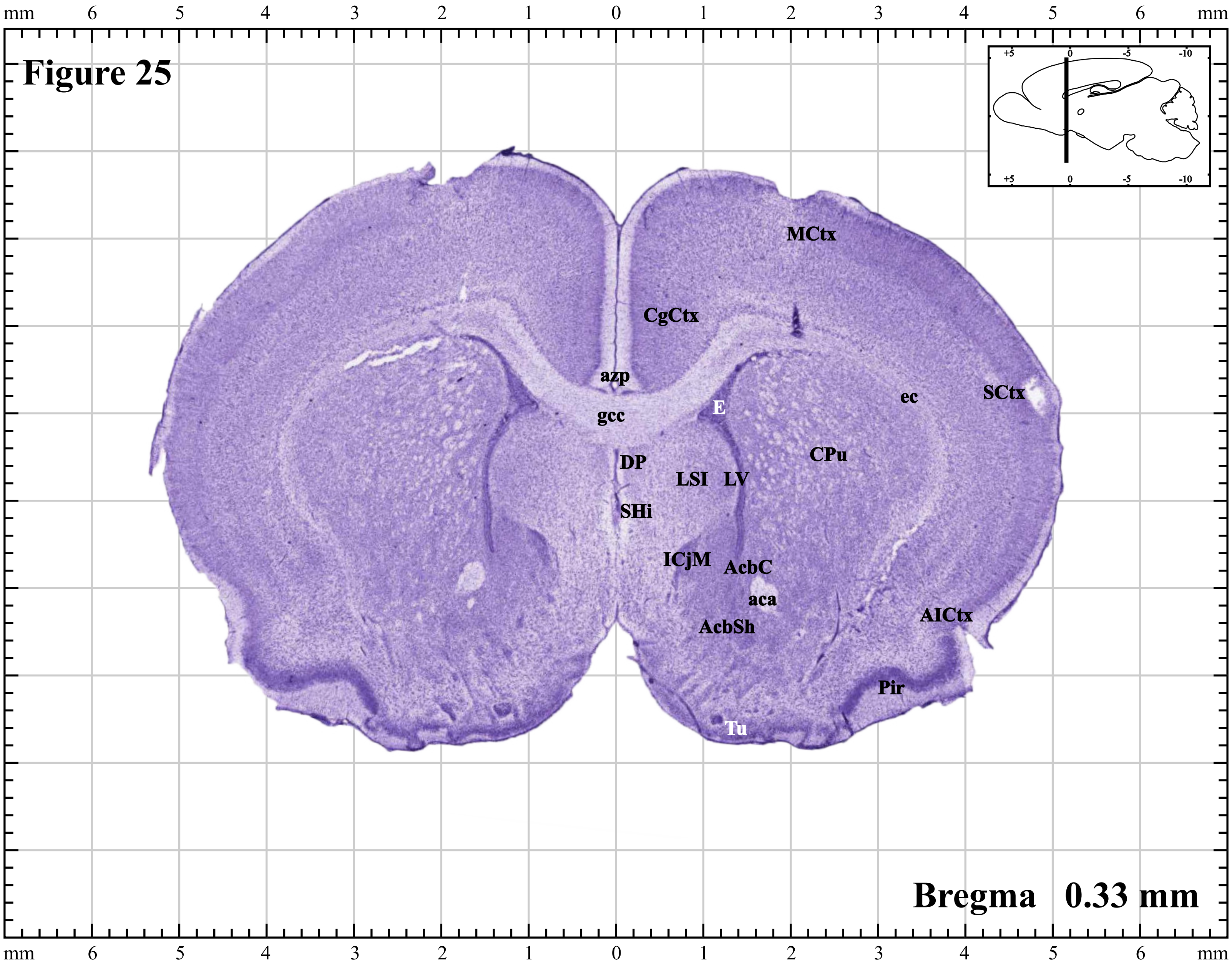

Bregma 0.33 mm

aca anterior commissure, anterior part
azp azygous pericallosal artery
AcbC accumbens nucleus, core
AcbSh accumbens shell
AICtx agranular insular cortex
CgCtx cingulate cortex
CPu caudate putamen (striatum)
DP dorsal peduncular cortex
ec external capsule
E ependyma and subependymal layer
gcc genu of the corpus callosum
ICjM islands of Calleja, major island
fmi forceps major of corpus callosum
LV lateral ventricle
LSI lateral septal nucleus, intermediate part
MCtx motor cortex
Pir piriform cortex
SCtx somatosensory cortex
SHi septohippocampal nucleus
Tu olfactory tubercle

Figure 26

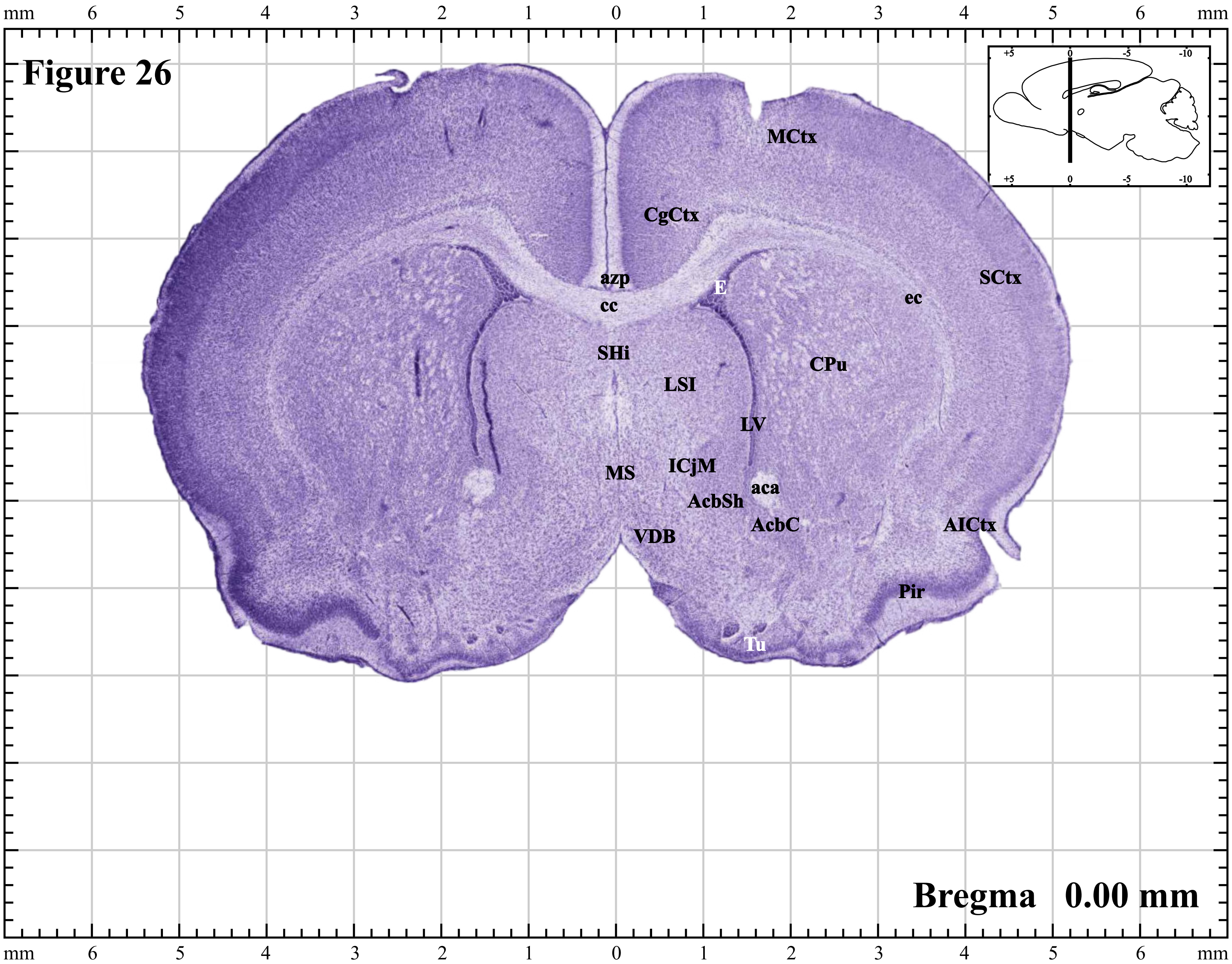

Bregma 0.00 mm

aca anterior commissure, anterior part
azp azygous pericallosal artery
AcbC accumbens nucleus, core
AcbSh accumbens shell
AICtx agranular insular cortex
cc corpus callosum
CgCtx cingulate cortex
CPu caudate putamen (striatum)
ec external capsule
E ependyma and subependymal layer
ICjM islands of Calleja, major island
LV lateral ventricle
LSI lateral septal nucleus, intermediate part
MS medial septal nucleus
MCtx motor cortex
Pir piriform cortex
SCtx somatosensory cortex
SHi septohippocampal nucleus
Tu olfactory tubercle
VDB nucleus of the vertical limb of the diagonal band

Figure 27

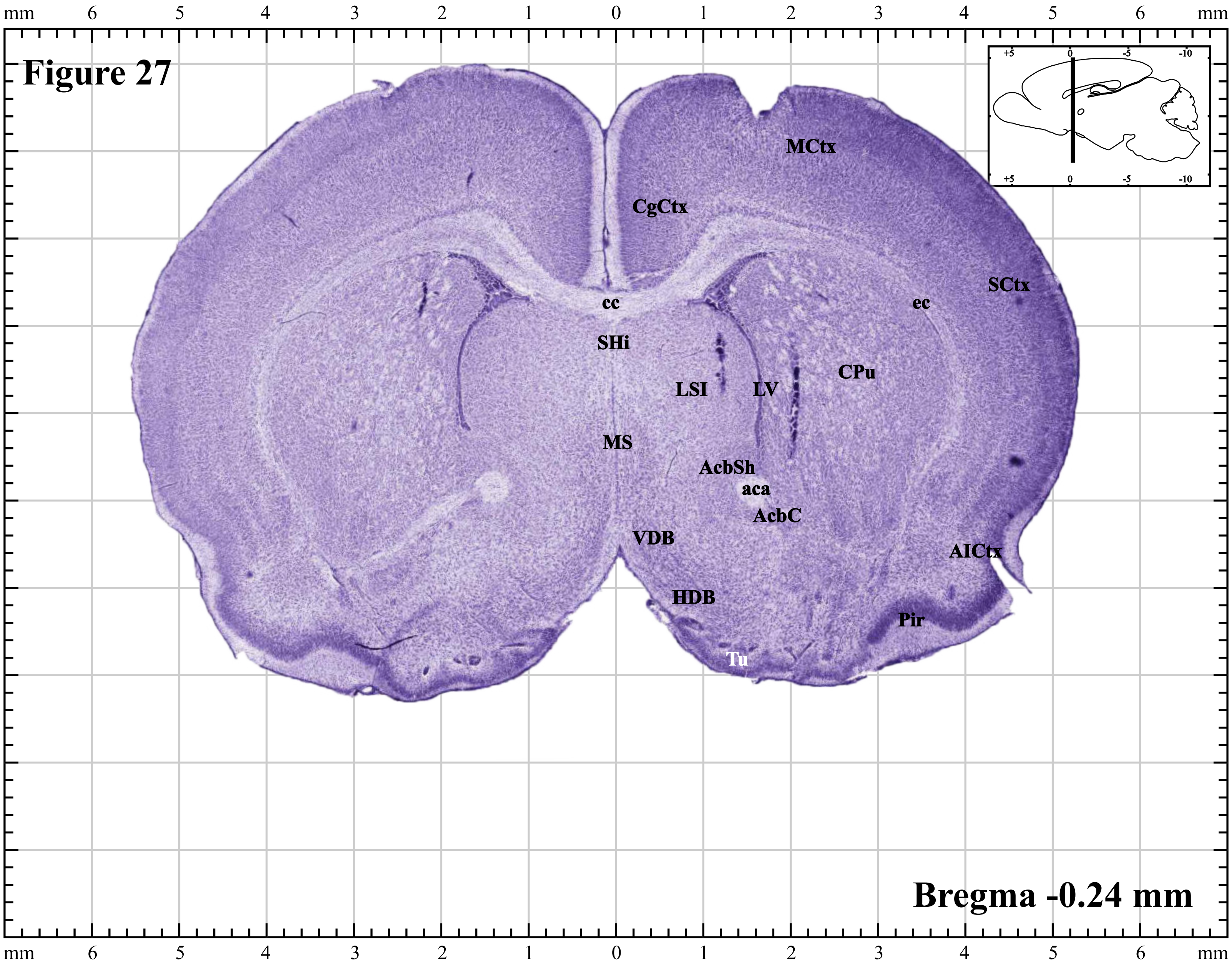

Bregma -0.24 mm

aca anterior commissure, anterior part
AcbC accumbens nucleus, core
AcbSh accumbens shell
AICtx agranular insular cortex
cc corpus callosum
CgCtx cingulate cortex
CPu caudate putamen (striatum)
ec external capsule
HDB nucleus of the horizontal limb
LV lateral ventricle
LSI lateral septal nucleus, intermediate part
MCtx motor cortex
MS medial septal nucleus
Pir piriform cortex
SCtx somatosensory cortex
SHi septohippocampal nucleus
VDB nucleus of the vertical limb of the diagonal band
Tu olfactory tubercle

Figure 28

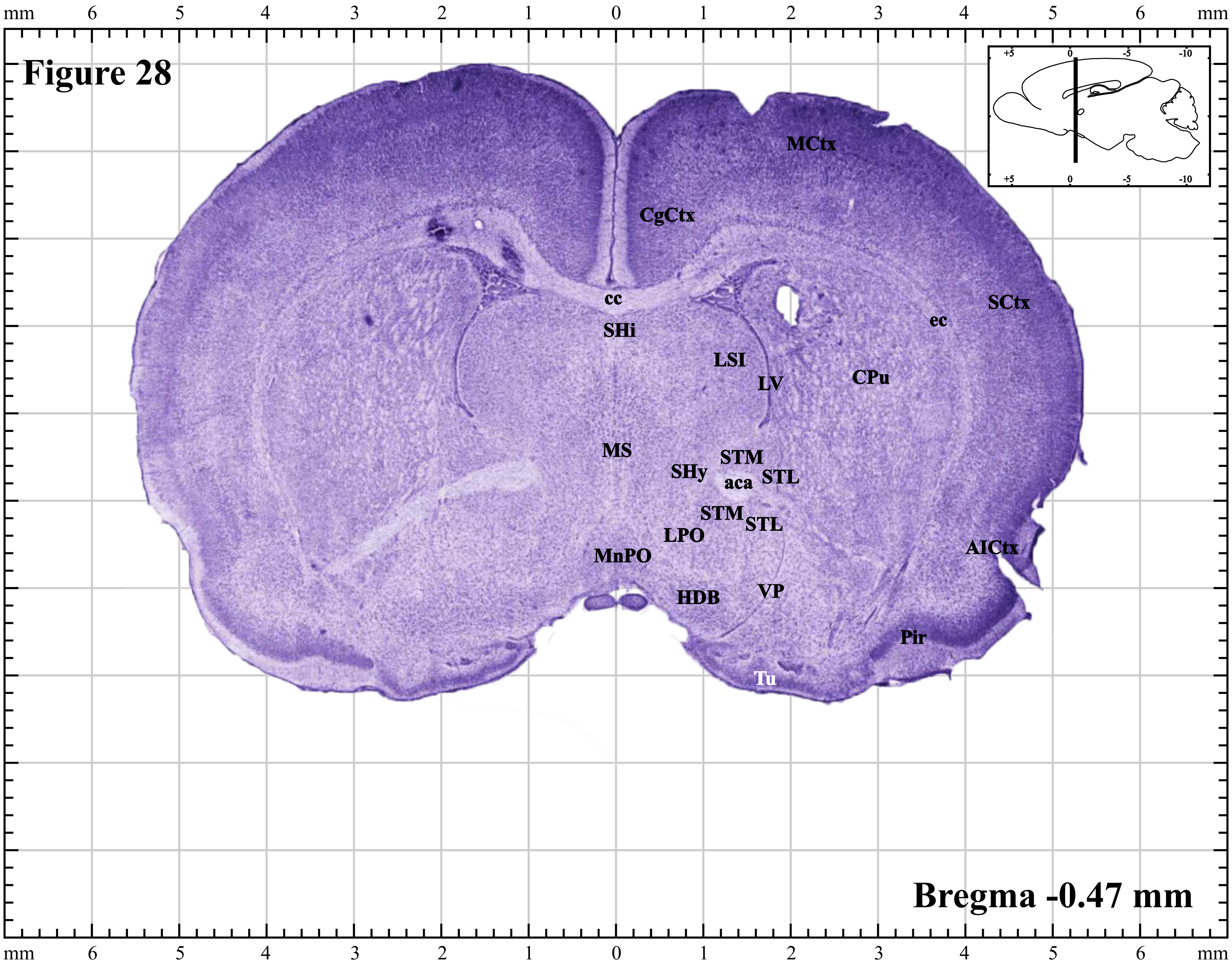

aca anterior commissure, anterior part
AICtx agranular insular cortex
cc corpus callosum
CPu caudate putamen
Cgctx cingulate cortex
ec external capsule
HDB nucleus of the horizontal limb of the diagonal band
LPO lateral preoptic area
LSI lateral septal nucleus, intermediate part
LV lateral ventricle
MnPO median preoptic nucleus
MCtx motor cortex
MS medial septal nucleus
Pir piriform cortex
SHi septohippocampal nucleus
SCtx somatosensory cortex
SHy septohypothalamic nucleus
STM bed nucleus of the stria terminalis, medial division
STL bed nucleus of the stria terminalis, lateral division
Tu olfactory tubercle
VP ventral pallidum

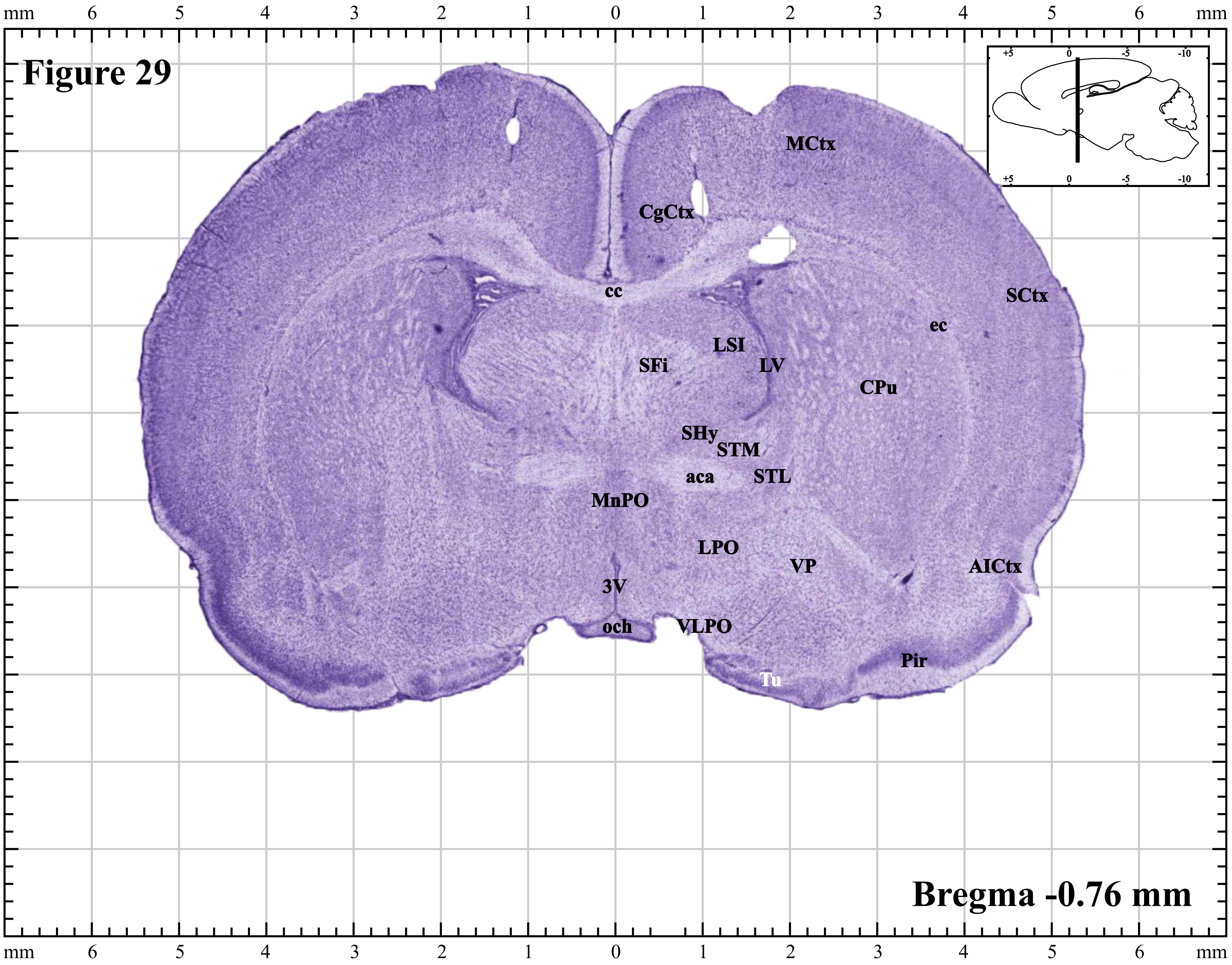

3V 3rd ventricle
aca anterior commissure,anterior part
AICtx agranular insular cortex
cc corpus callosum
CPu caudate putamen
Cgctx cingulate cortex
ec external capsule
LPO lateral preoptic area
LSI lateral septal nucleus, intermediate part
LV lateral ventricle
MnPO median preoptic nucleus
MCtx motor cortex
och optic chiasm
Pir piriform cortex
SCtx somatosensory cortex
SHy septohypothalamic nucleus
SFi septofimbrial nucleus
STL bed nucleus of the stria terminalis, lateral division
STM bed nucleus of the stria terminalis, medial division
Tu olfactory tubercle
VP ventral pallidum
VLPO ventrolateral preoptic nucleus

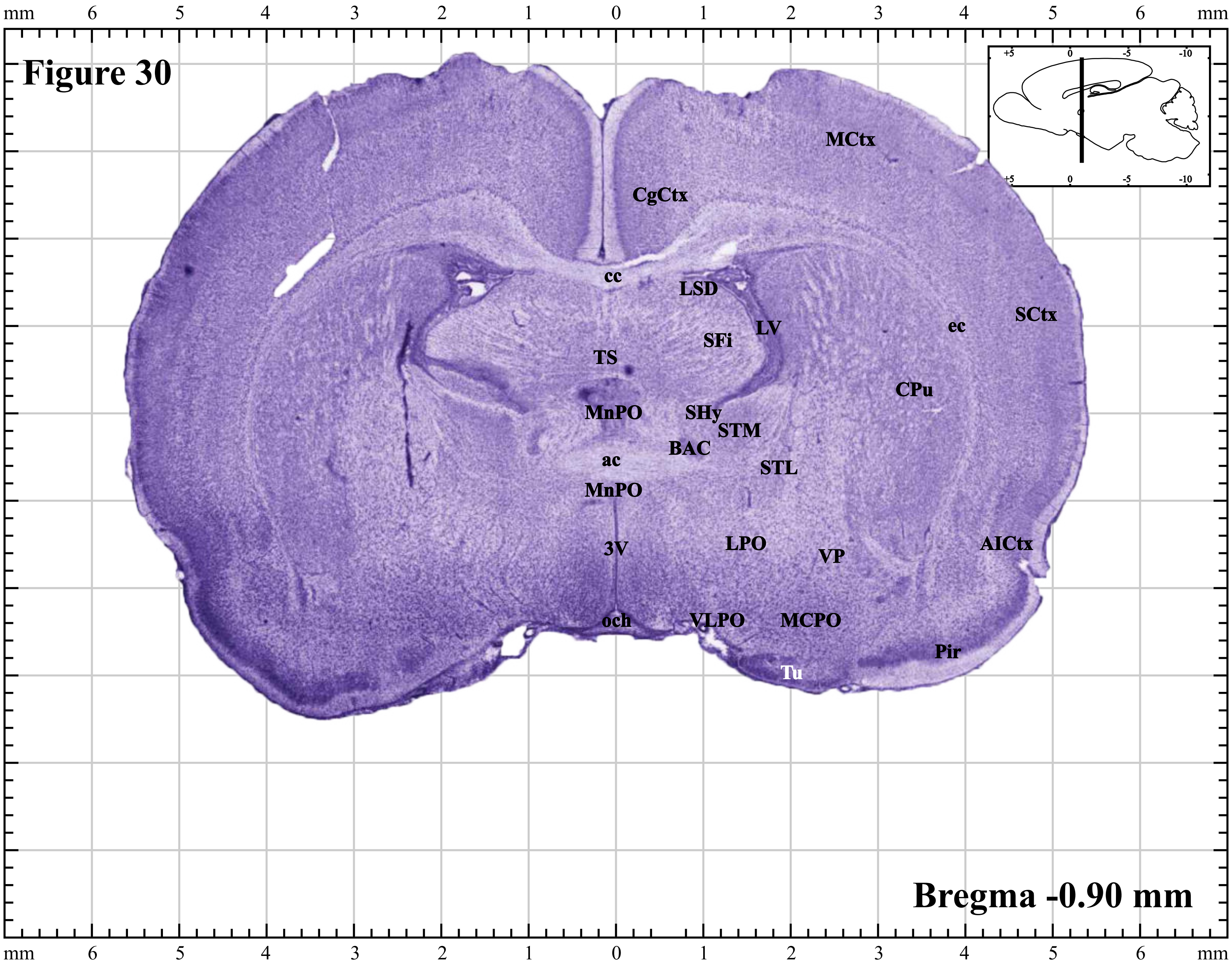

- **3V** 3rd ventricle
- **ac** anterior commissure
- **AICtx** agranular insular cortex
- **BAC** bed nucleus of the anterior commissure
- **cc** corpus callosum
- **CPu** caudate putamen
- **Cgctx** cingulate cortex
- **ec** external capsule
- **LPO** lateral preoptic area
- **LSD** lateral septal nucleus, dorsal part
- **LV** lateral ventricle
- **MnPO** median preoptic nucleus
- **MCtx** motor cortex
- **och** optic chiasm
- **Pir** piriform cortex
- **MCPO** magnocellular preoptic nucleus
- **SCtx** somatosensory cortex
- **SHy** septohypothalamic nucleus
- **SFi** septofimbrial nucleus
- **STL** bed nucleus of the stria terminalis, lateral division
- **STM** bed nucleus of the stria terminalis, medial division
- **Tu** olfactory tubercle
- **VP** ventral pallidum
- **VLPO** ventrolateral preoptic nucleus

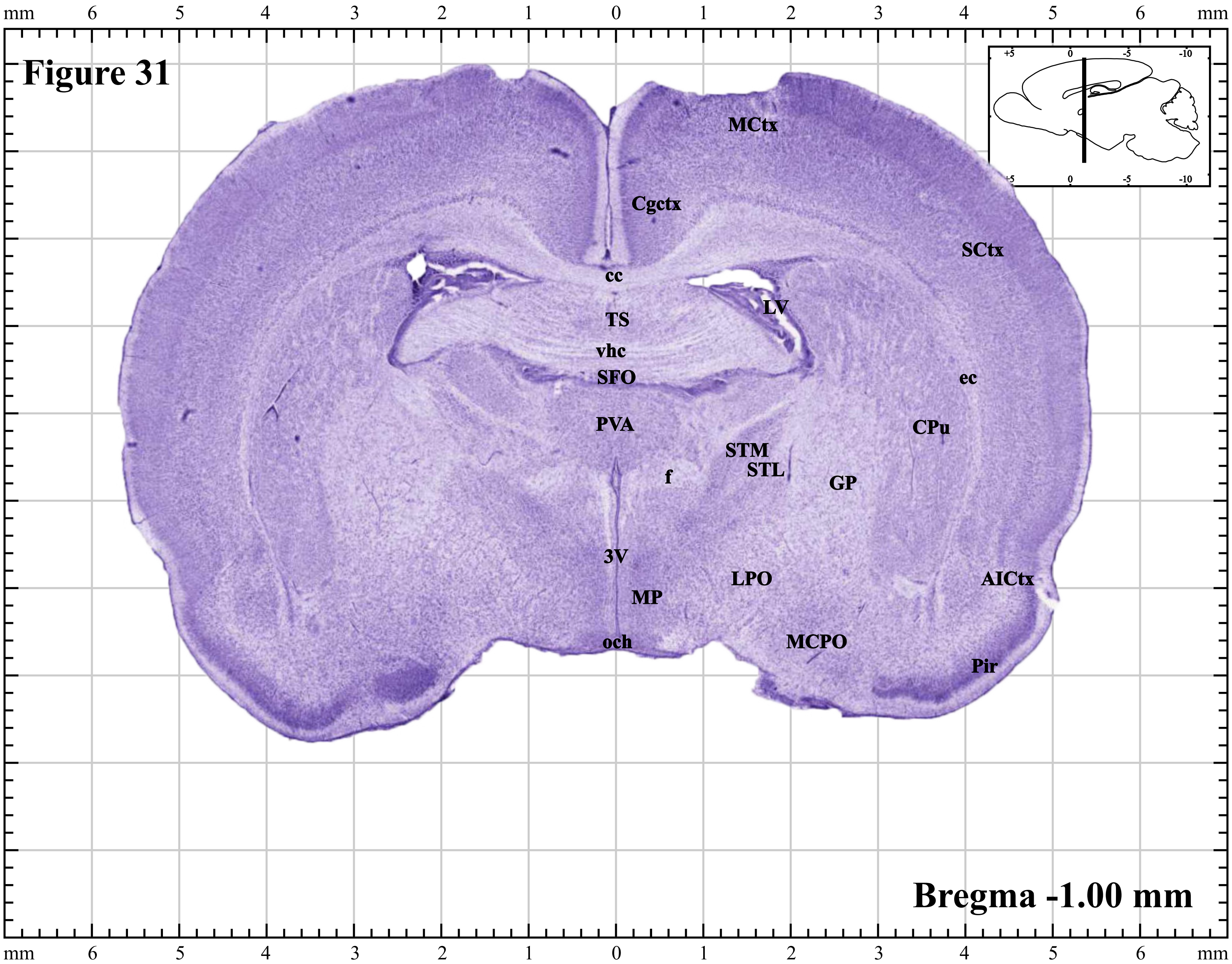

3V 3rd ventricle
AICtx agranular insular cortex
cc corpus callosum
CPu caudate putamen
Cgctx cingulate cortex
ec external capsule
f fornix
GP globus pallidus
LPO lateral preoptic area
LV lateral ventricle
MCtx motor cortex
MP medial preoptic nucleus
MCPO magnocellular preoptic nucleus
och optic chiasm
PVA paraventricular thalamic nucleus, anterior part
Pir piriform cortex
SCtx somatosensory cortex
STM bed nucleus of the stria terminalis, medial division
STL bed nucleus of the stria terminalis, lateral division
SFO subfornical organ
TS triangular septal nucleus
VLPO ventrolateral preoptic nucleus
vhc ventral hippocampal commissure

Figure 32

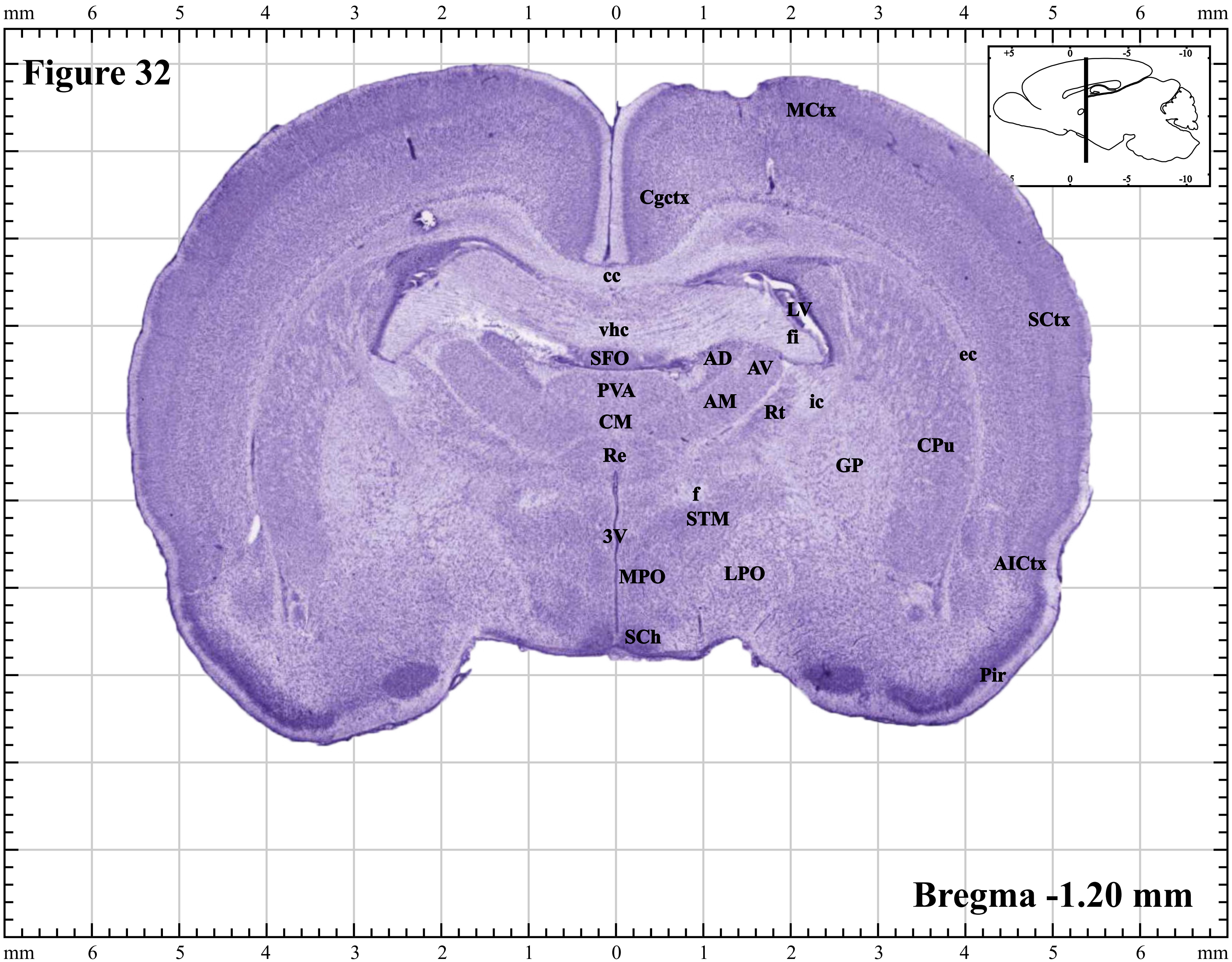

Bregma -1.20 mm

3V 3rd ventricle
AD anterodorsal thalamic nucleus
AM anteromedial thalamic nucleus
AICtx agranular insular cortex
AV anteroventral thalamic nucleus
cc corpus callosum
CPu caudate putamen
Cgctx cingulate cortex
CM central medial thalamic nucleus
ec external capsule
fi fimbria of the hippocampus
f fornix
GP globus pallidus
ic internal capsule
LPO lateral preoptic area
LV lateral ventricle
MPO medial preoptic nucleus
MCtx motor cortex
Rt reticular thalamic nucleus
och optic chiasm
PVA paraventricular thalamic nucleus, anterior part
Pir piriform cortex
SCh suprachiasmatic nucleus
SFO subfornical organ
STM bed nucleus of the stria terminalis, medial division
SCtx somatosensory cortex
vhc ventral hippocampal commissure
Re reuniens thalamic nucleus

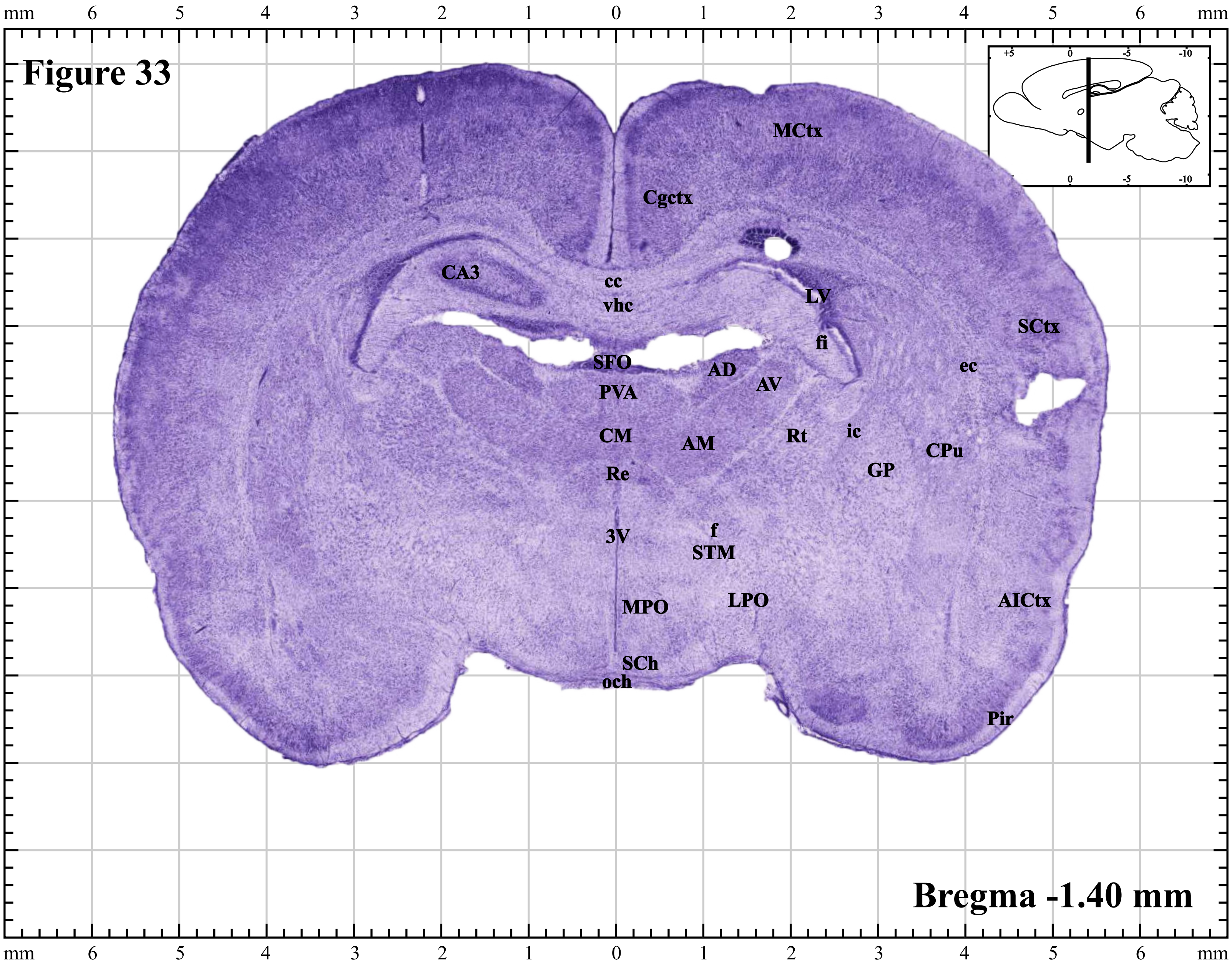

3V 3rd ventricle
AD anterodorsal thalamic nucleus
AM anteromedial thalamic nucleus
AICtx agranular insular cortex
AV anteroventral thalamic nucleus
cc corpus callosum
CPu caudate putamen
Cgctx cingulate cortex
CA3 field CA3 of the hippocampus
CM central medial thalamic nucleus
ec external capsule
fi fimbria of the hippocampus
f fornix
GP globus pallidus
ic internal capsule
LPO lateral preoptic area
LV lateral ventricle
MPO medial preoptic nucleus
MCtx motor cortex
Rt reticular thalamic nucleus
och optic chiasm
PVA paraventricular thalamic nucleus, anterior part
Pir piriform cortex
SCh suprachiasmatic nucleus
SFO subfornical organ
STM bed nucleus of the stria terminalis, medial division
SCtx somatosensory cortex
vhc ventral hippocampal commissure
Re reuniens thalamic nucleus

Figure 34

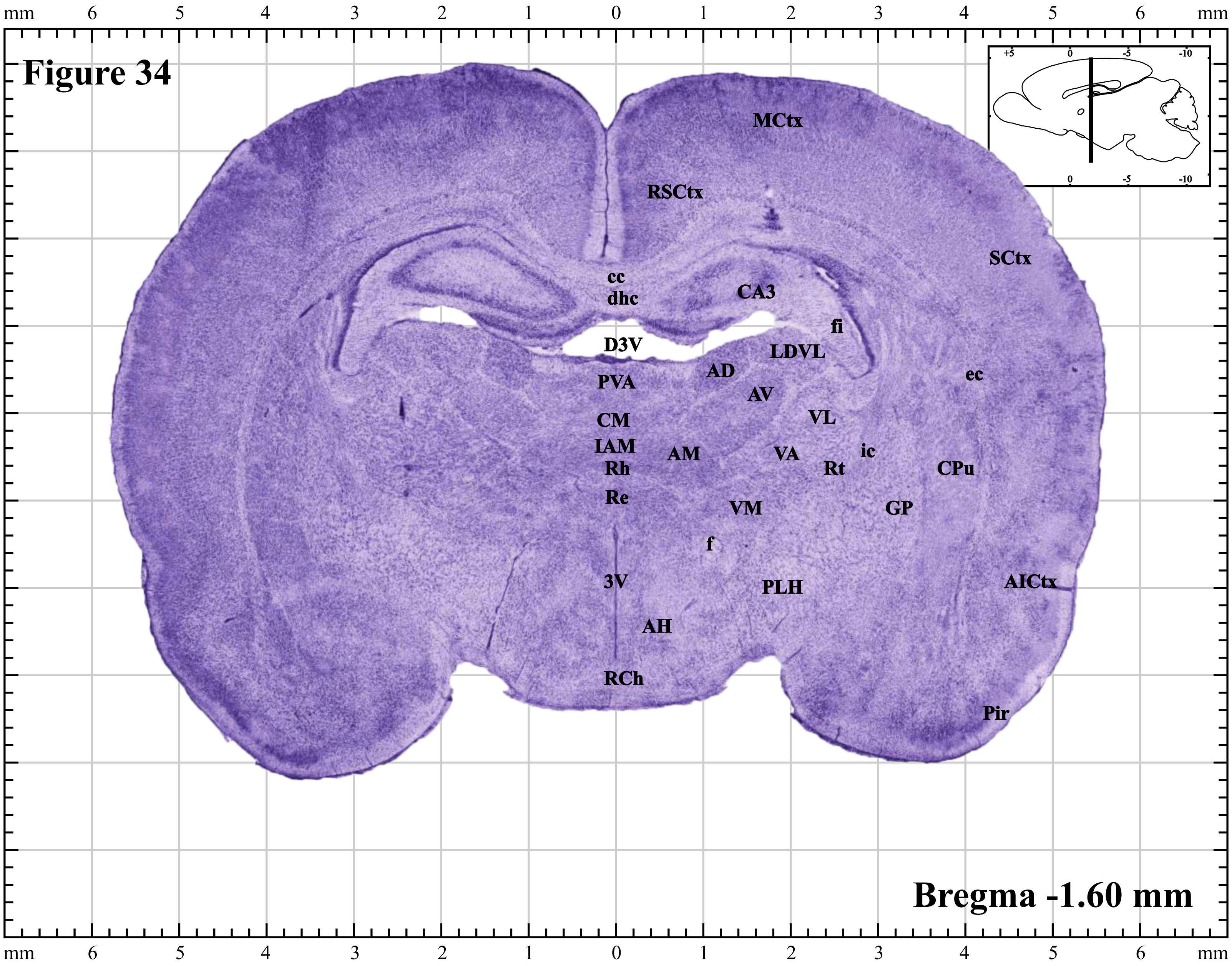

Bregma -1.60 mm

3V 3rd ventricle
AICtx agranular insular cortex
AD anterodorsal thalamic nucleus
AM anteromedial thalamic nucleus
AV anteroventral thalamic nucleus
AH anterior hypothalamic area
cc corpus callosum
CPu caudate putamen
CA3 field CA3 of the hippocampus
CM central medial thalamic nucleus
dhc dorsol hippocampal commissure
D3V dorsal 3rd ventricle
ec external capsule
fi fimbria of the hippocampus
f fornix
GP globus pallidus
ic internal capsule
IAM interanteromedial thalamic nucleus
LDVL laterodorsal thalamic nucleus, ventrolateral part
MCtx motor cortex
PVA paraventricular thalamic nucleus, anterior part
Pir piriform cortex
PLH peduncular part of lateral hypothalamus
Rt reticular thalamic nucleus
Re reuniens thalamic nucleus
SCtx somatosensory cortex
RCh retrochiasmatic area
RSCtx retrospenial cortex
Rh rhomboid thalamic nucleus
VA ventral anterior thalamic nucleus
VM ventromedial thalamic nucleus
VL ventrolateral thalamic nucleus

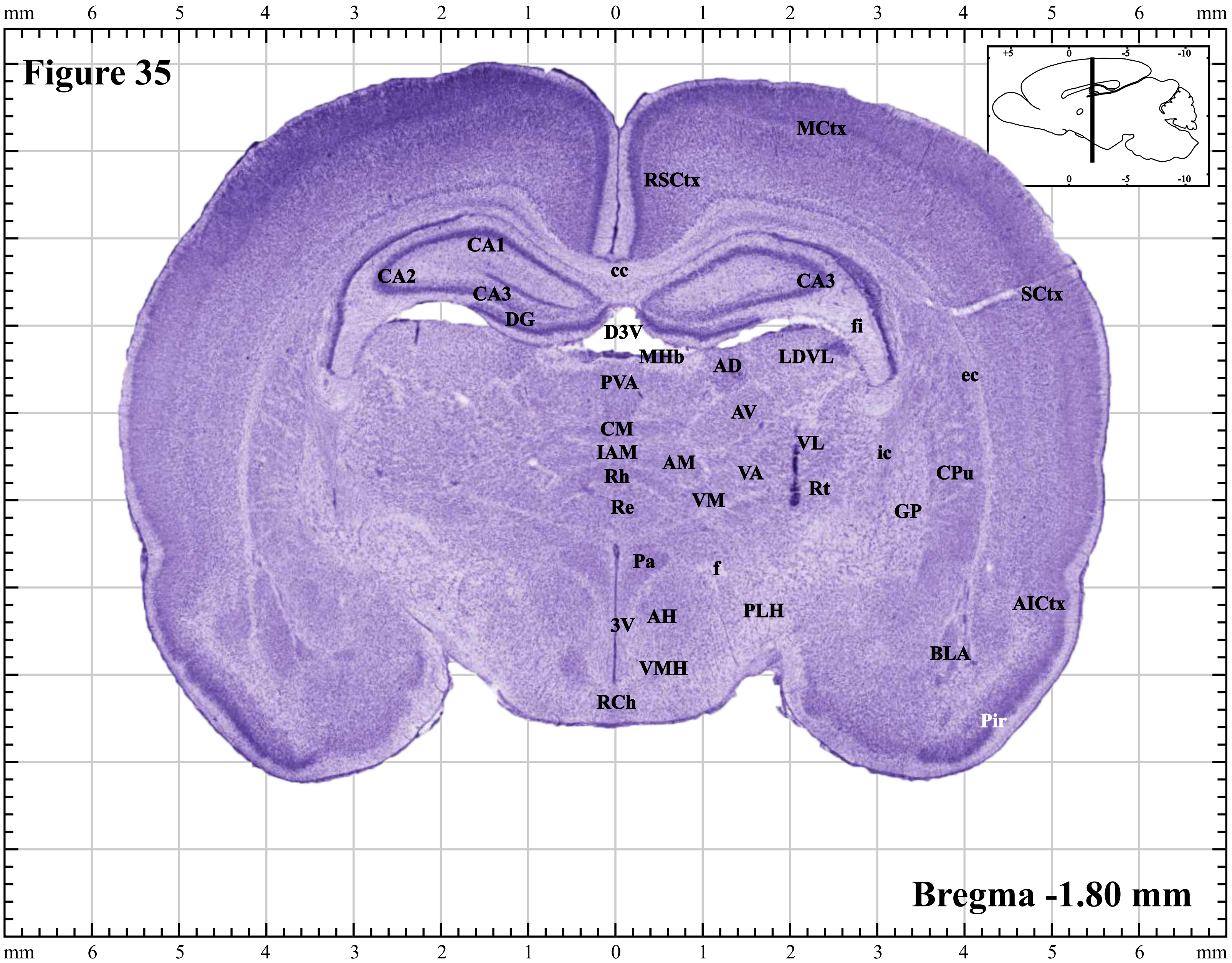

3V 3rd ventricle
AD anterodorsal thalamic nucleus
AH anterior hypothalamic area
AM anteromedial thalamic nucleus
AV anteroventral thalamic nucleus
AICtx agranular insular cortex
BLA basolateral amygdaloid nucleus, anterior part
cc corpus callosum
CA1 field CA1 of the hippocampus
CA2 field CA2 of the hippocampus
CA3 field CA3 of the hippocampus
CPu caudate putamen
CM central medial thalamic nucleus
D3V dorsal 3rd ventricle
ec external capsule
f fornix
fi fimbria of the hippocampus
GP globus pallidus
ic internal capsule
DG dentate gyrus
IAM interanteromedial thalamic nucleus
LDVL laterodorsal thalamic nucleus, ventrolateral part
MHb medial habenular nucleus
MCtx motor cortex
Pir piriform cortex
PVA paraventricular thalamic nucleus, anterior part
Pa paraventricular hypoth nucleus
PLH peduncular part of lateral hypothalamus
Rt reticular thalamic nucleus
Rh rhomboid thalamic nucleus
RCh retrochiasmatic area
Re reuniens thalamic nucleus
RSCtx retrospenial cortex
SCtx somatosensory cortex
Sub submedius thalamic nucleus
VA ventral anterior thalamic nucleus
VM ventromedial thalamic nucleus
VL ventrolateral thalamic nucleus
VMH ventromedial hypothalamic nucleus

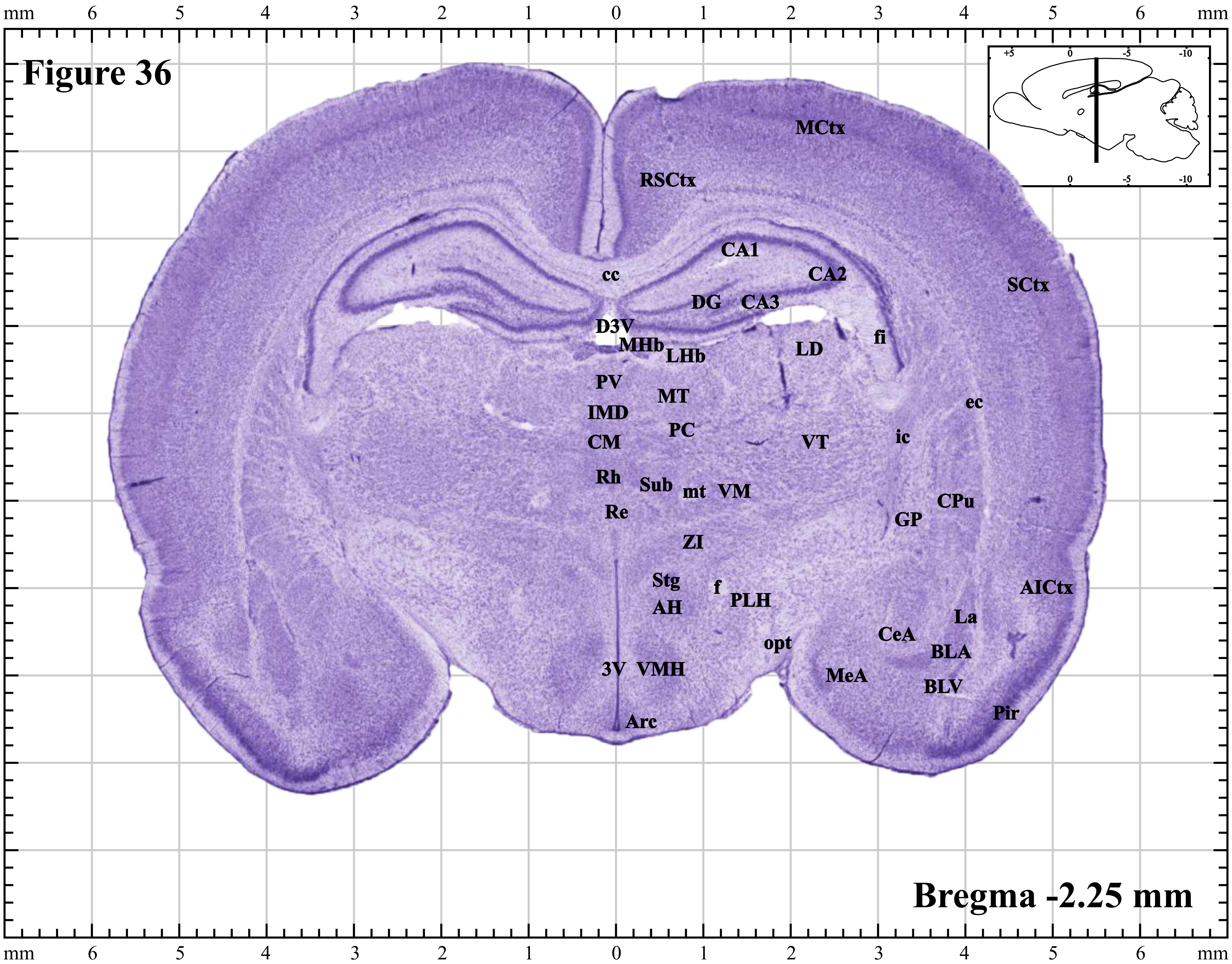

- **3V** 3rd ventricle
- **AH** anterior hypothalamic area
- **AICtx** agranular insular cortex
- **Arc** arcuate hypothalamic nucleus
- **BLA** basolateral amygdaloid nucleus, anterior part
- **BLV** basolateral amygdaloid nucleus, ventral part
- **cc** corpus callosum
- **CPu** caudate putamen
- **CA1** field CA1 of the hippocampus
- **CA2** field CA2 of the hippocampus
- **CA3** field CA3 of the hippocampus
- **CM** central medial thalamic nucleus
- **CeA** central amygdaloid nucleus
- **D3V** dorsal 3rd ventricle
- **DG** dentate gyrus
- **ec** external capsule
- **f** fornix
- **fi** fimbria of the hippocampus
- **GP** globus pallidus
- **ic** internal capsule
- **IMD** intermediodorsal thalamic nucleus
- **LHb** lateral habenular nucleus
- **LD** laterodorsal thalamic nucleus
- **mt** mammillothalamic tract
- **MT** medial thalamus
- **MHb** medial habenular nucleus
- **MCtx** motor cortex
- **MeA** medial amygdaloid nucleus
- **opt** optic tract
- **Pir** piriform cortex
- **PC** paracentral thalamic nucleus
- **PLH** peduncular part of lateral hypothalamus
- **Rh** rhomboid thalamic nucleus
- **Re** reuniens thalamic nucleus
- **RSCtx** retrospenial cortex
- **SCtx** somatosensory cortex
- **Sub** submedius thalamic nucleus
- **Stg** stigmoid hypothalamic nucleus
- **VT** ventral thalamus
- **VM** ventromedial thalamic nucleus
- **VMH** ventromedial hypothalamic nucleus
- **ZI** zona incerta

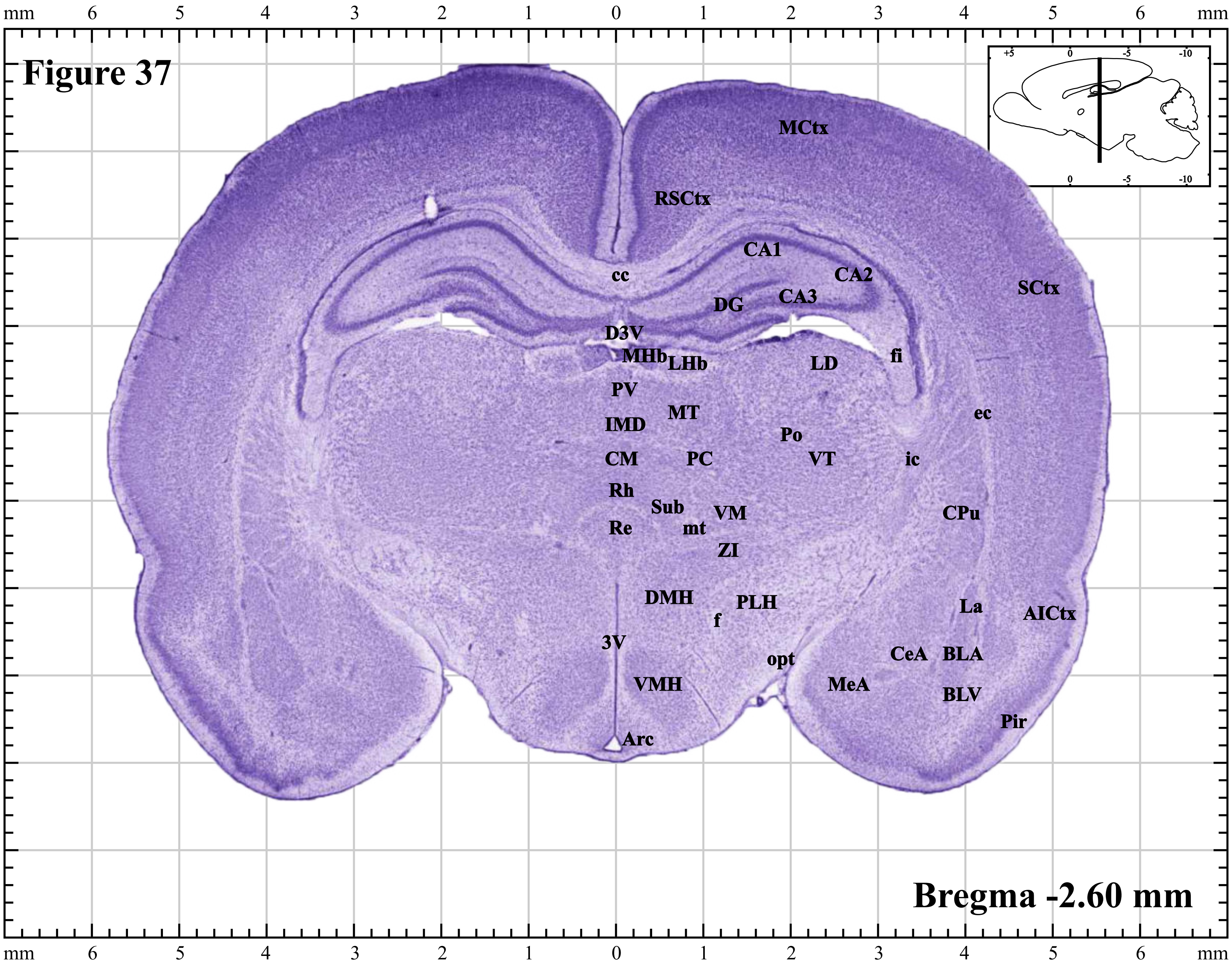

3V 3rd ventricle
AICtx agranular insular cortex
Arc arcuate hypothalamic nucleus
BLA basolateral amygdaloid nucleus, anterior part
BLV basolateral amygdaloid nucleus, ventral part
cc corpus callosum
CPu caudate putamen
CA1 field CA1 of the hippocampus
CA2 field CA2 of the hippocampus
CA3 field CA3 of the hippocampus
CM central medial thalamic nucleus
CeA central amygdaloid nucleus
DMH dorsomedial hypothalamic nucleus
D3V dorsal 3rd ventricle
DG dentate gyrus
ec external capsule
f fornix
fi fimbria of the hippocampus
ic internal capsule
IMD intermediodorsal thalamic nucleus
LHb lateral habenular nucleus
LD laterodorsal thalamic nucleus
mt mammillothalamic tract
MT medial thalamus
MHb medial habenular nucleus
MCtx motor cortex
MeA medial amygdaloid nucleus
opt optic tract
Pir piriform cortex
PC paracentral thalamic nucleus
PLH peduncular part of lateral hypothalamus
Po posterior thalamic nuclear group
Rh rhomboid thalamic nucleus
Re reuniens thalamic nucleus
RSCtx retrospenial cortex
SCtx somatosensory cortex
Sub submedius thalamic nucleus
VT ventral thalamus
VM ventromedial thalamic nucleus
VMH ventromedial hypothalamic nucleus
ZI zona incerta

Figure 38

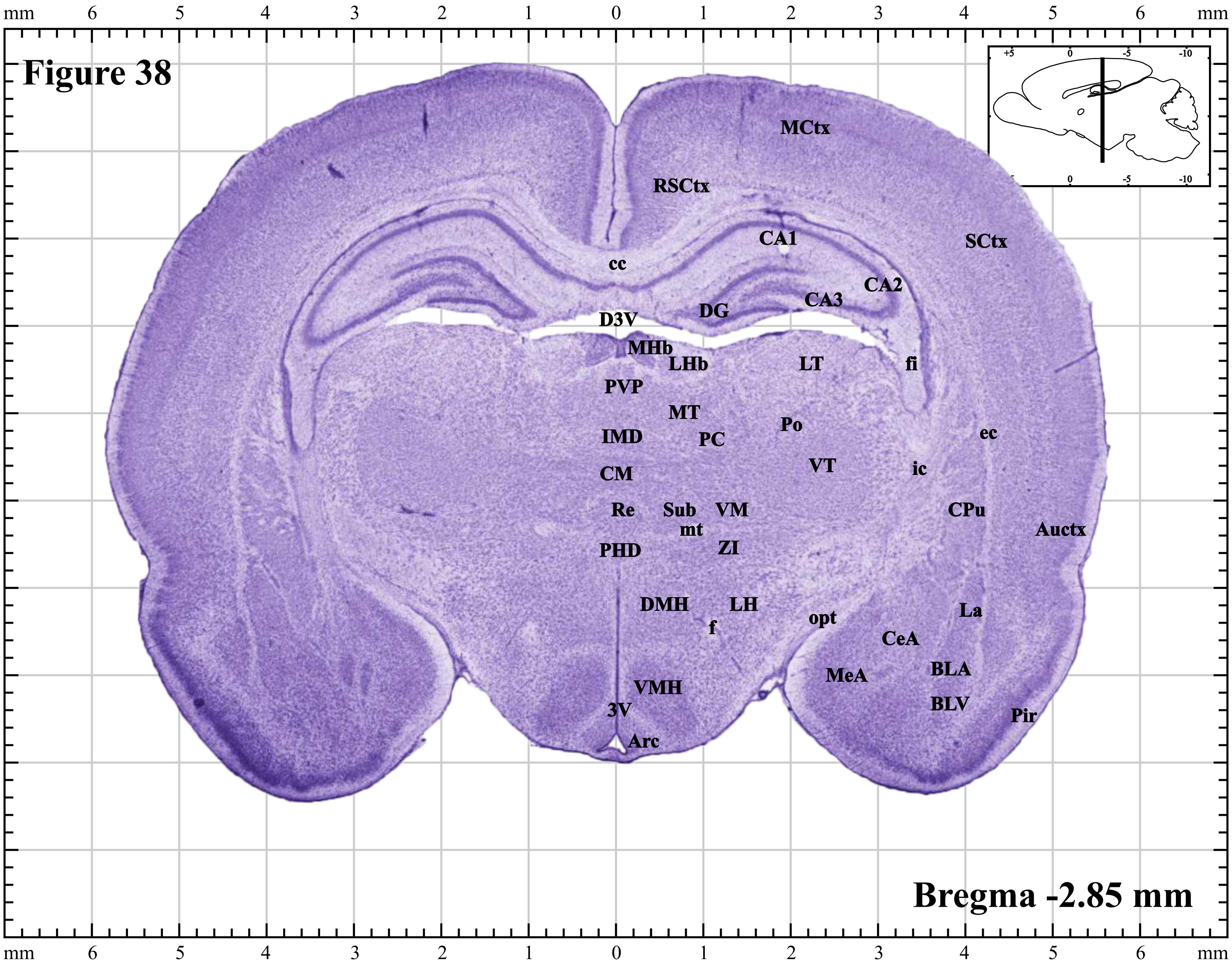

Bregma -2.85 mm

- **3V** medial longitudinal fasciculus
- **Arc** arcuate hypothalamic nucleus
- **Auctx** auditory cortex
- **BLA** basolateral amygdaloid nucleus, anterior part
- **BLV** basolateral amygdaloid nucleus, ventral part
- **CA1** field CA1 of the hippocampus
- **CA2** field CA2 of the hippocampus
- **CA3** field CA3 of the hippocampus
- **cc** corpus callosum
- **CeA** central amygdaloid nucleus
- **CM** central medial thalamic nucleus
- **CPu** caudate putamen
- **D3V** dorsal 3rd ventricle
- **DMH** dorsomedial hypothalamic nucleus
- **DG** dentate gyrus
- **ec** external capsule
- **f** fornix
- **fi** fimbria of the hippocampus
- **ic** internal capsule
- **IMD** intermediodorsal thalamic nucleus
- **La** lat amygdaloid nucleus
- **LHb** lateral habenular nucleus
- **LH** lateral hypothalamic area
- **LT** lateral thalamus
- **MeA** medial amygdaloid nucleus
- **mt** mammillothalamic tract
- **MHb** medial habenular nucleus
- **MT** medial thalamus
- **opt** optic tract
- **MCtx** motor cortex
- **PC** paracentral thalamic nucleus
- **Pir** piriform cortex
- **PVP** paraventricular thalamic nucleus, posterior part
- **Po** posterior thalamic nuclear group
- **PHD** posterior hypothalamic area, dorsal part
- **Re** reuniens thalamic nucleus
- **RSCtx** retrospenial cortex
- **SCtx** somatosensory cortex
- **Sub** submedius thalamic nucleus
- **VM** ventromedial thalamic nucleus
- **VMH** ventromedial hypothalamic nucleus
- **VT** ventral thalamus
- **ZI** zona incerta

Figure 39

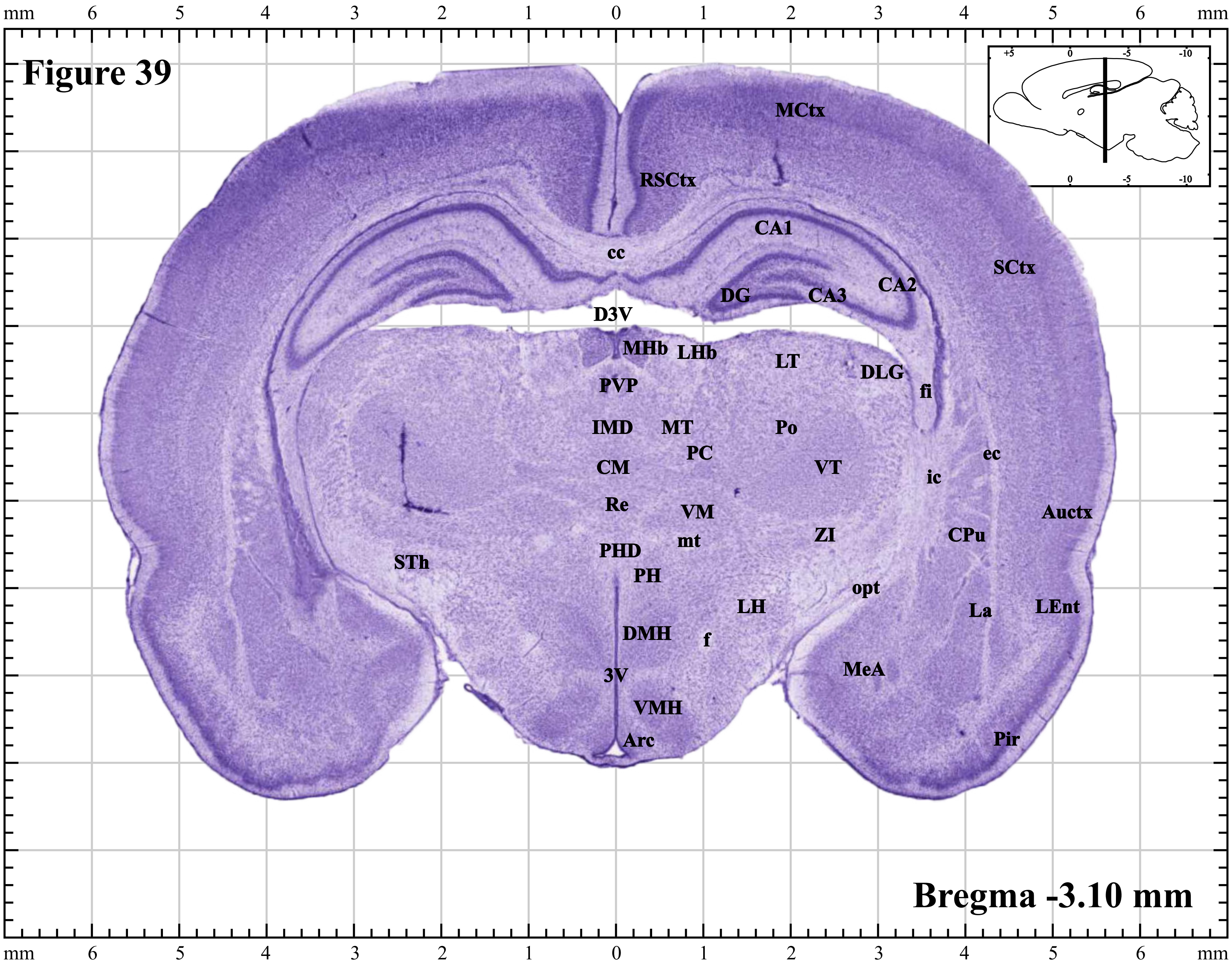

Bregma -3.10 mm

3V medial longitudinal fasciculus
Arc arcuate hypothalamic nucleus
Auctx auditory cortex
cc corpus callosum
CA1 field CA1 of the hippocampus
CA2 field CA2 of the hippocampus
CA3 field CA3 of the hippocampus
CM central medial thalamic nucleus
CPu caudate putamen
D3V dorsal 3rd ventricle
DMH dorsomedial hypothalamic nucleus
DG dentate gyrus
DLG dorsal lateral geniculate nucleus
ec external capsule
f fornix
ic internal capsule
IMD intermediodorsal thalamic nucleus
La lat amygdaloid nucleus
LEnt lateral entorhinal cortex
LHb lateral habenular nucleus
LH lateral hypothalamic area
LT lateral thalamus
LV lateral ventricle
mt mammillothalamic tract
MeA medial amygdaloid nucleus
MHb medial habenular nucleus
MT medial thalamus
opt optic tract
MCtx motor cortex
PC paracentral thalamic nucleus
Pir piriform cortex
PVP paraventricular thalamic nucleus, posterior part
Po posterior thalamic nuclear group
PH posterior hypothalamic nucleus
PHD posterior hypothalamic area, dorsal part
Re reuniens thalamic nucleus
RSCtx retrospenial cortex
SCtx somatosensory cortex
STh subthalamic nucleus
VM ventromedial thalamic nucleus
VMH ventromedial hypothalamic nucleus
VT ventral thalamus
ZI zona incerta

Figure 40

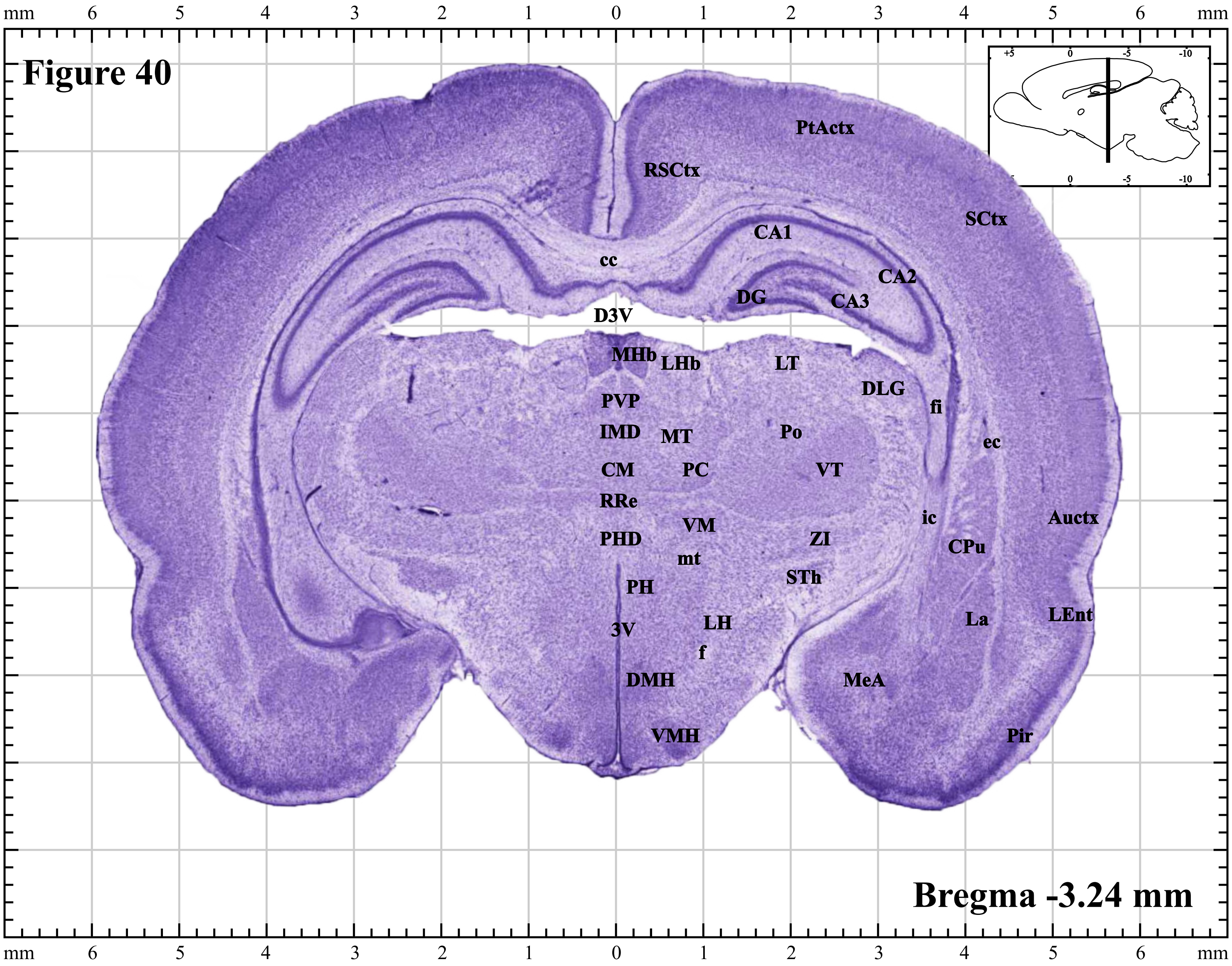

3V medial longitudinal fasciculus
Auctx auditory cortex
CA1 field CA1 of the hippocampus
CA2 field CA2 of the hippocampus
CA3 field CA3 of the hippocampus
cc corpus callosum
CeA central amygdaloid nucleus
CM central medial thalamic nucleus
CPu Caudate putamen
D3V dorsal 3rd ventricle
DMH dorsomedial hypothalamic nucleus
DG dentate gyrus
DLG dorsal lateral geniculate nucleus
ec external capsule
f fornix
fi fimbria of the hippocampus
ic internal capsule
IMD intermediodorsal thalamic nucleus
La lat amygdaloid nucleus
LEnt lateral entorhinal cortex
LHb lateral habenular nucleus
LH lateral hypothalamic area
LT lateral thalamus
LV lateral ventricle
mt mammillothalamic tract
MHb medial habenular nucleus
MT medial thalamus
MeA medial amygdaloid nucleus
PC paracentral thalamic nucleus
Pir piriform cortex
PVP paraventricular thalamic nucleus, posterior part
Po posterior thalamic nuclear group
PH posterior hypothalamic nucleus
PtActx parietal association cortex
PHD posterior hypothalamic area, dorsal part
RRe retrouniens area
RSCtx retrospenial cortex
SCtx somatosensory cortex
STh subthalamic nucleus
VM ventromedial thalamic nucleus
VT ventral thalamus
VMH ventromedial hypothalamic nucleus
ZI zona incerta

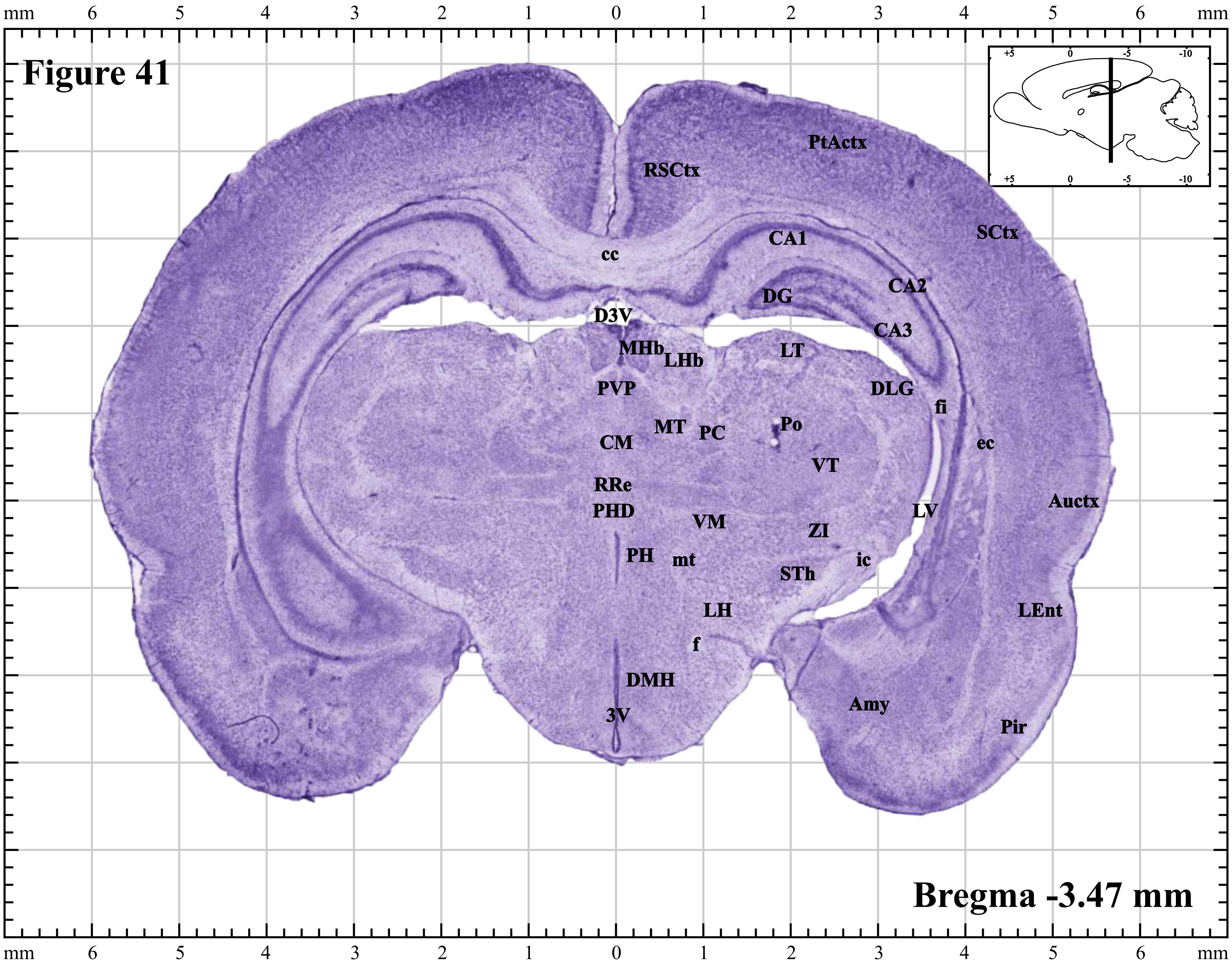

3V medial longitudinal fasciculus
Auctx auditory cortex
CA1 field CA1 of the hippocampus
CA2 field CA2 of the hippocampus
CA3 field CA3 of the hippocampus
cc corpus callosum
CeA central amygdaloid nucleus
CM central medial thalamic nucleus
D3V dorsal 3rd ventricle
DMH dorsomedial hypothalamic nucleus
DG dentate gyrus
DLG dorsal lateral geniculate nucleus
ec external capsule
f fornix
fi fimbria of the hippocampus
ic internal capsule
IMD intermediodorsal thalamic nucleus
LEnt lateral entorhinal cortex
LHb lateral habenular nucleus
LH lateral hypothalamic area
LT lateral thalamus
LV lateral ventricle
mt mammillothalamic tract
MHb medial habenular nucleus
MT medial thalamus
PC paracentral thalamic nucleus
Pir piriform cortex
PVP paraventricular thalamic nucleus, posterior part
Po posterior thalamic nuclear group
PH posterior hypothalamic nucleus
PtActx parietal association cortex
PHD posterior hypothalamic area, dorsal part
RRe retrouniens area
RSCtx retrospenial cortex
SCtx somatosensory cortex
STh subthalamic nucleus
VM ventromedial thalamic nucleus
VT ventral thalamus
ZI zona incerta

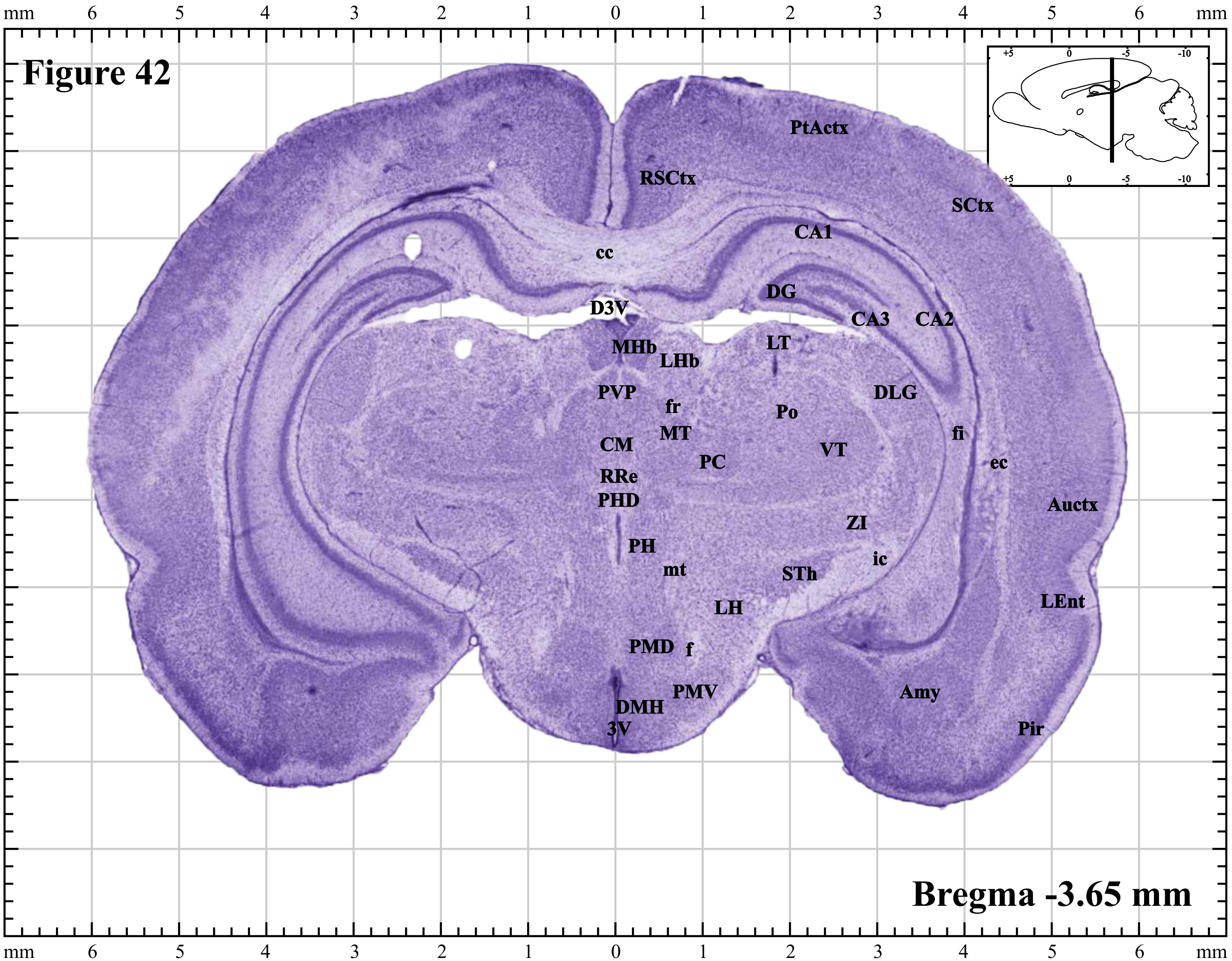

3V medial longitudinal fasciculus
Auctx auditory cortex
CA1 field CA1 of the hippocampus
CA2 field CA2 of the hippocampus
CA3 field CA3 of the hippocampus
cc corpus callosum
CM central medial thalamic nucleus
D3V dorsal 3rd ventricle
DG dentate gyrus
DMH dorsomedial hypothalamic nucleus
DLG dorsal lateral geniculate nucleus
ec external capsule
f fornix
fi fimbria of the hippocampus
fr fasciculus retroflexus
ic internal capsule
LHb lateral habenular nucleus
LEnt lateral entorhinal cortex
LH lateral hypothalamic area
LT lateral thalamus
mt mammillothalamic tract
MHb medial habenular nucleus
MT medial thalamus
PC paracentral thalamic nucleus
Pir piriform cortex
Po posterior thalamic nuclear group
PH posterior hypothalamic nucleus
PVP paraventricular thalamic nucleus, posterior part
PtActx parietal association cortex
PHD posterior hypothalamic area, dorsal part
PMV premammillary nucleus, ventral part
PMD premammillary nucleus, dorsal part
RRe retrouniens area
RSCtx retrospenial cortex
SCtx somatosensory cortex
STh subthalamic nucleus
VT ventral thalamus
ZI zona incerta

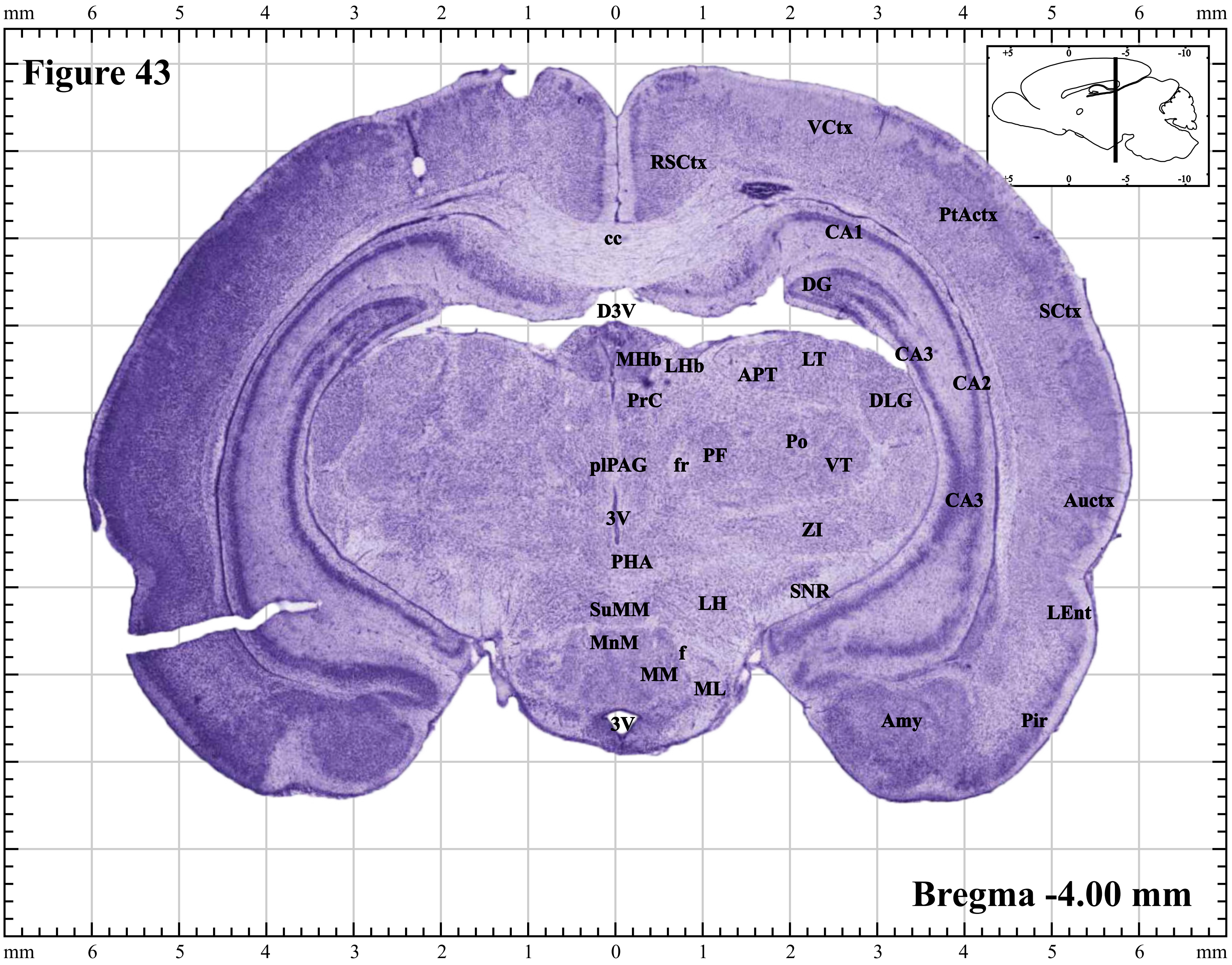

3V medial longitudinal fasciculus
Auctx auditory cortex
Amy amygdaloid nuclei
APT anterior pretectal nucleus
cc corpus callosum
CA1 field CA1 of the hippocampus
CA2 field CA2 of the hippocampus
CA3 field CA3 of the hippocampus
D3V dorsal 3rd ventricle
DG dentate gyrus
DLG dorsal lateral geniculate nucleus
f fornix
fr fasciculus retroflexus
LH lateral hypothalamic area
LT lateral thalamus
LEnt lateral entorhinal cortex
LHb lateral habenular nucleus
MnM medial mammillary nucleus, median part
ML medial mammillary nucleus, lateral part
MHb medial habenular nucleus median part
MM medial mammillary nucleus, medial part
PF parafascicular thalamic nucleus
PrC precommissural nucleus
Pir piriform cortex
Po posterior thalamic nuclear group
plPAG pleomorphic part of periaqueductai gray
PHA posterior hypothalamic area
PtActx parietal association cortex
RSCtx retrospenial cortex
SCtx somatosensory cortex
SNR substantia nigra, reticular part
SuMM supramammillary nucleus, medial part
VCtx visual cortex
VT ventral thalamus
ZI zona incerta

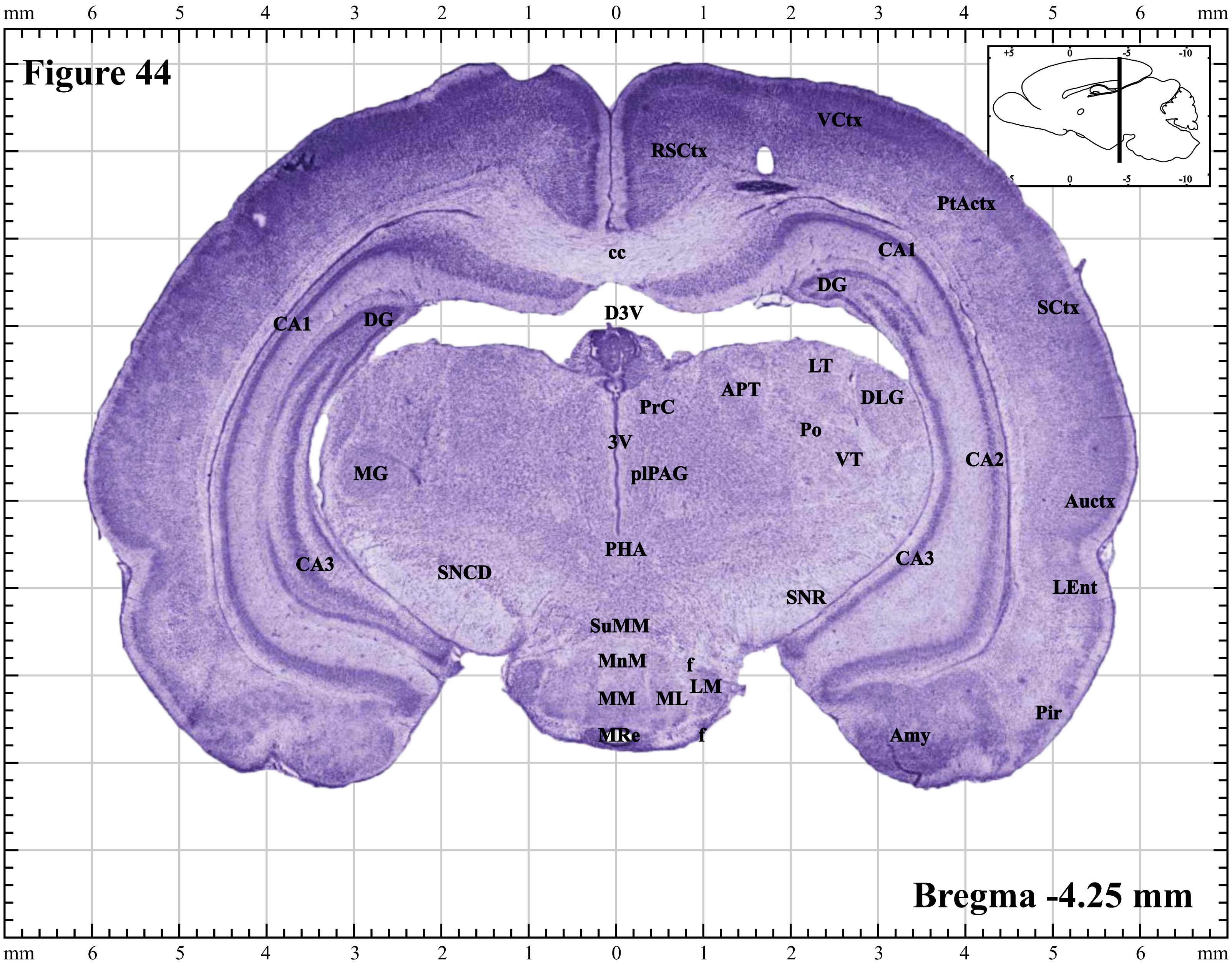

3V medial longitudinal fasciculus
Auctx auditory cortex
Amy amygdaloid nuclei
APT anterior pretectal nucleus
CA1 field CA1 of the hippocampus
CA3 field CA3 of the hippocampus
D3V dorsal 3rd ventricle
DG dentate gyrus
DLG dorsal lateral geniculate nucleus
f fornix
LM lateral mammillary nucleus
LT lateral thalamus
LEnt lateral entorhinal cortex
ML medial mammillary nucleus, lateral part
MnM medial mammillary nucleus, median part
MG medial geniculate nucleus
MM medial mammillary nucleus, medial part
MRe mammillary recess of the 3rd ventricle
Pir piriform cortex
Po posterior thalamic nuclear group
PrC precommissural nucleus
PHA posterior hypothalamic area
PtActx parietal association cortex
plPAG pleomorphic part of periaqueductai gray
RSCtx retrospenial cortex
SCtx somatosensory cortex
SNR substantia nigra, reticular part
SuMM supramammillary nucleus, medial part
SNCD substantia nigra, compact part, dorsal tier
VCtx visual cortex
VT ventral thalamus

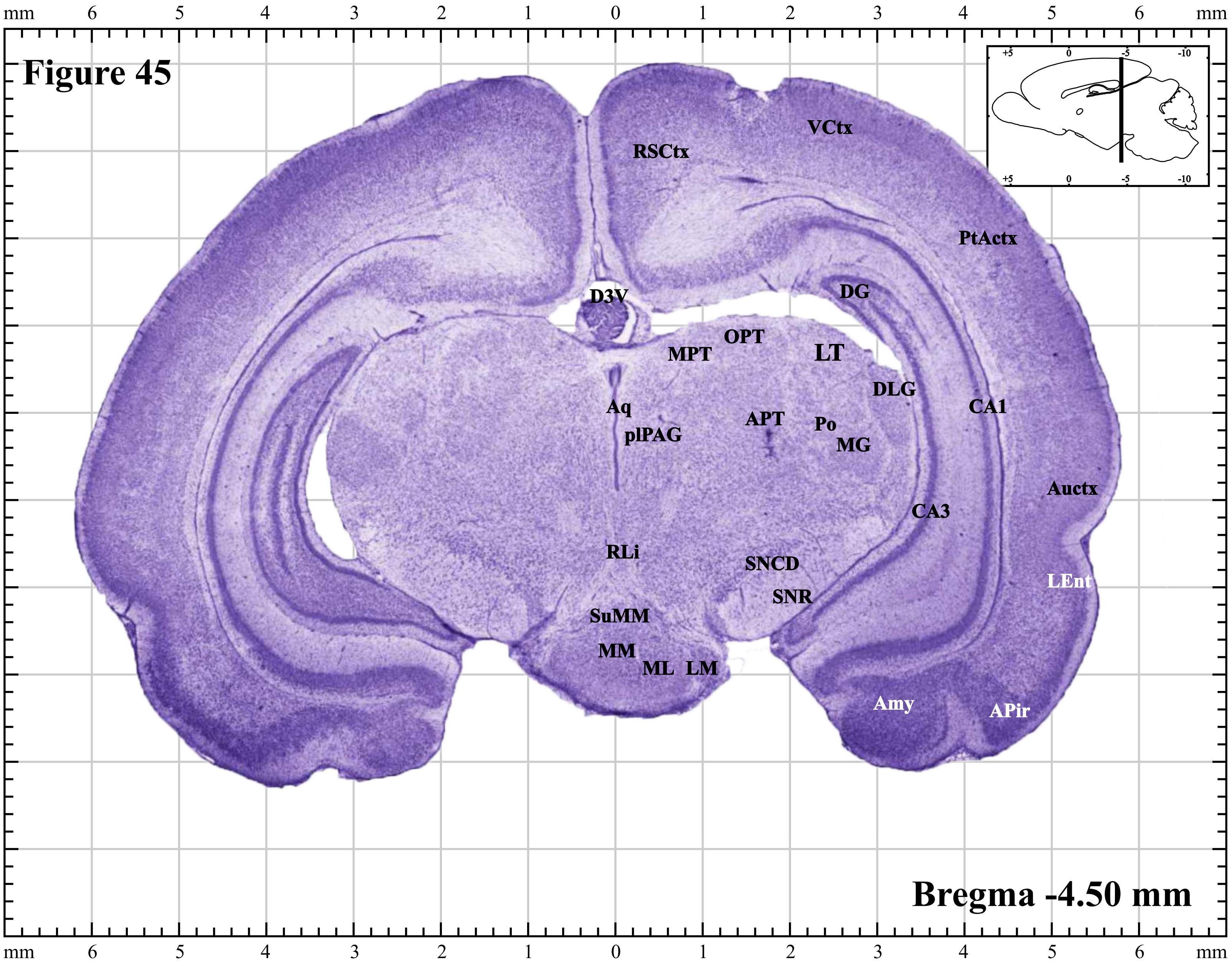

Aq aqueduct
Auctx auditory cortex
Amy amygdaloid nuclei
APir amygdalopiriform transition area
APT anterior pretectal nucleus
CA1 field CA1 of the hippocampus
CA2 field CA2 of the hippocampus
CA3 field CA3 of the hippocampus
D3V dorsal 3rd ventricle
DG dentate gyrus
DLG dorsal lateral geniculate nucleus
f fornix
LT lateral thalamus
LM lateral mammillary nucleus
LEnt lateral entorhinal cortex
ML medial mammillary nucleus, lateral part
MM medial mammillary nucleus, medial part
MG medial geniculate nucleus
MPT medial pretectal nucleus
OPT olivary pretectal nucleus
pc posterior commissure
plPAG pleomorphic part of periaqueductai gray
PtActx parietal association cortex
Po posterior thalamic nuclear group
RSCtx retrospenial cortex
RLi rostral linear nucleus of the raphe
SuMM supramammillary nucleus, medial part
SNR substantia nigra, reticular part
SNCD substantia nigra, compact part, dorsal tier
VCtx visual cortex

Figure 46

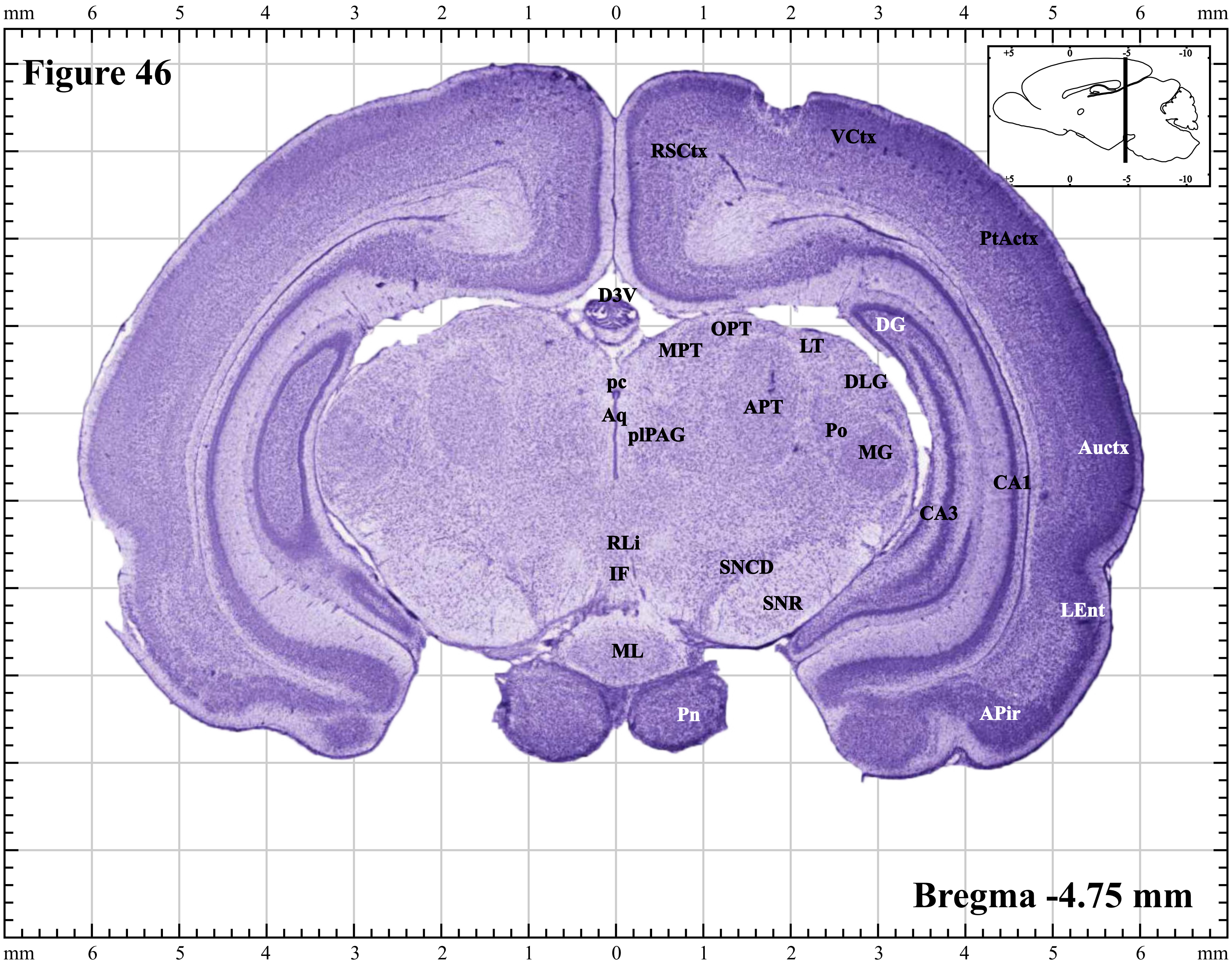

Aq aqueduct
Auctx auditory cortex
APir amygdalopiriform transition area
APT anterior pretectal nucleus
CA1 field CA1 of the hippocampus
CA3 field CA3 of the hippocampus
D3V dorsal 3rd ventricle
DG dentate gyrus
DLG dorsal lateral geniculate nucleus
IF interfascicular nucleus
LT lateral thalamus
LEnt lateral entorhinal cortex
ML medial mammillary nucleus, lateral part
MG medial geniculate nucleus
MPT medial pretectal nucleus
OPT olivary pretectal nucleus
PtActx parietal association cortex
Po posterior thalamic nuclear group
plPAG pleomorphic part of periaqueductai gray
pc posterior commissure
Pn pontine nuclei
RSCtx retrospenial cortex
RLi rostral linear nucleus of the raphe
SNR substantia nigra, reticular part
SNCD substantia nigra, compact part, dorsal tier
VCtx visual cortex

Figure 47

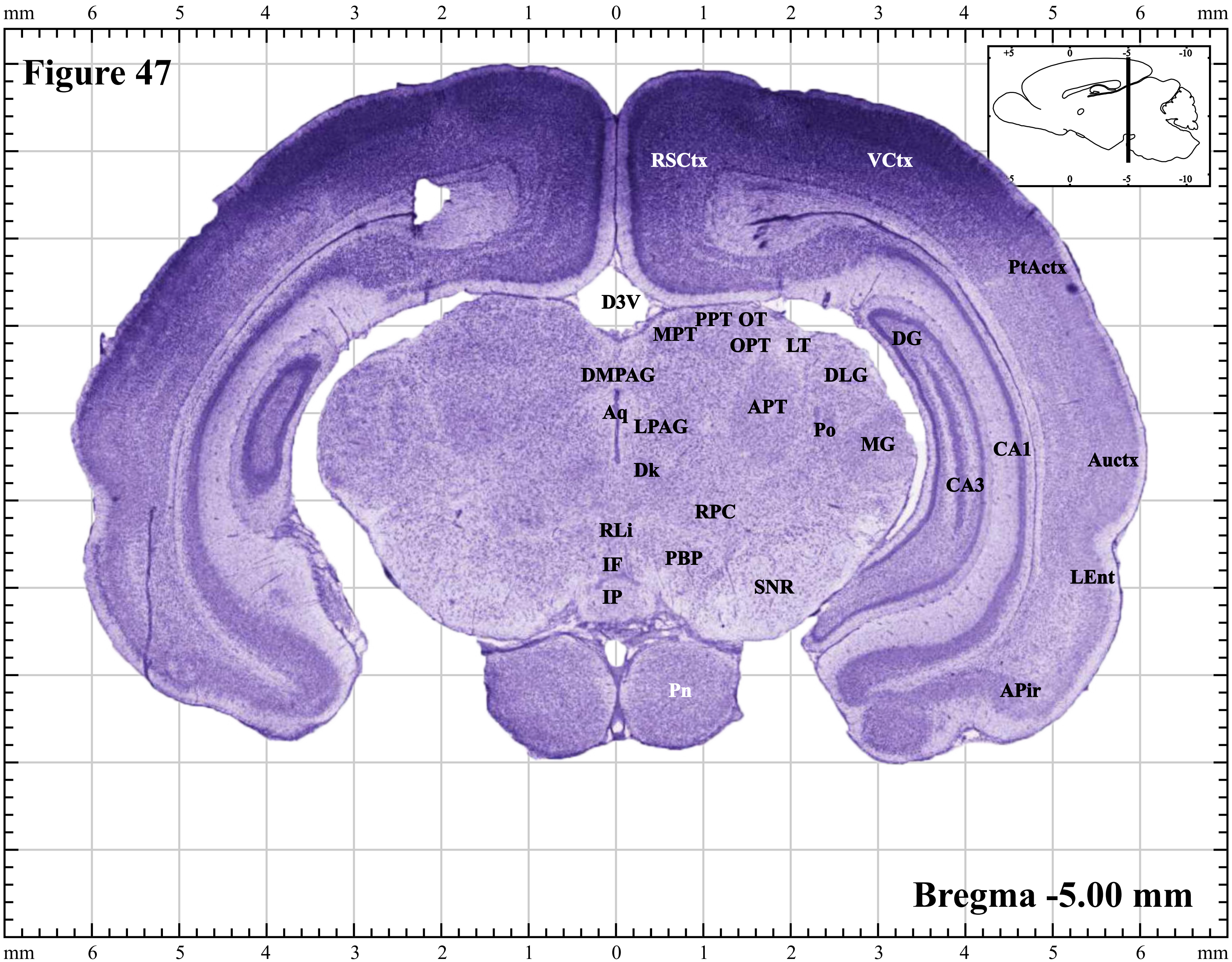

Bregma -5.00 mm

Aq aqueduct
Auctx auditory cortex
APT anterior pretectal nucleus
APir amygdalopiriform transition area
CA1 field CA1 of the hippocampus
CA3 field CA3 of the hippocampus
DMPAG dorsomedial periaqueductal gray
DG dentate gyrus
DLG dorsal lateral geniculate nucleus
Dk nucleus of Darkschewitsch
D3V dorsal 3rd ventricle
IC inferior colliculus
IF interfascicular nucleus
IP interpeduncular nucleus
LEnt lateral entorhinal cortex
LPAG lateral periaqueductal gray
LT lateral thalamus
MG medial geniculate nucleus
MPT medial pretectal nucleus
OT nucleus of the optic
OPT olivary pretectal nucleus
Pn pontine nuclei
Po posterior thalamic nuclear group
PBP parabrachial pigmented nucleus of the VTA
PtActx parietal association cortex
PPT posterior pretectal nucleus
RPC red nucleus, parvicellular part
RSCtx retrospenial cortex
RLi rostral linear nucleus of the raphe
RPC red nucleus, parvicellular part
SNR substantia nigra, reticular part
VCtx visual cortex

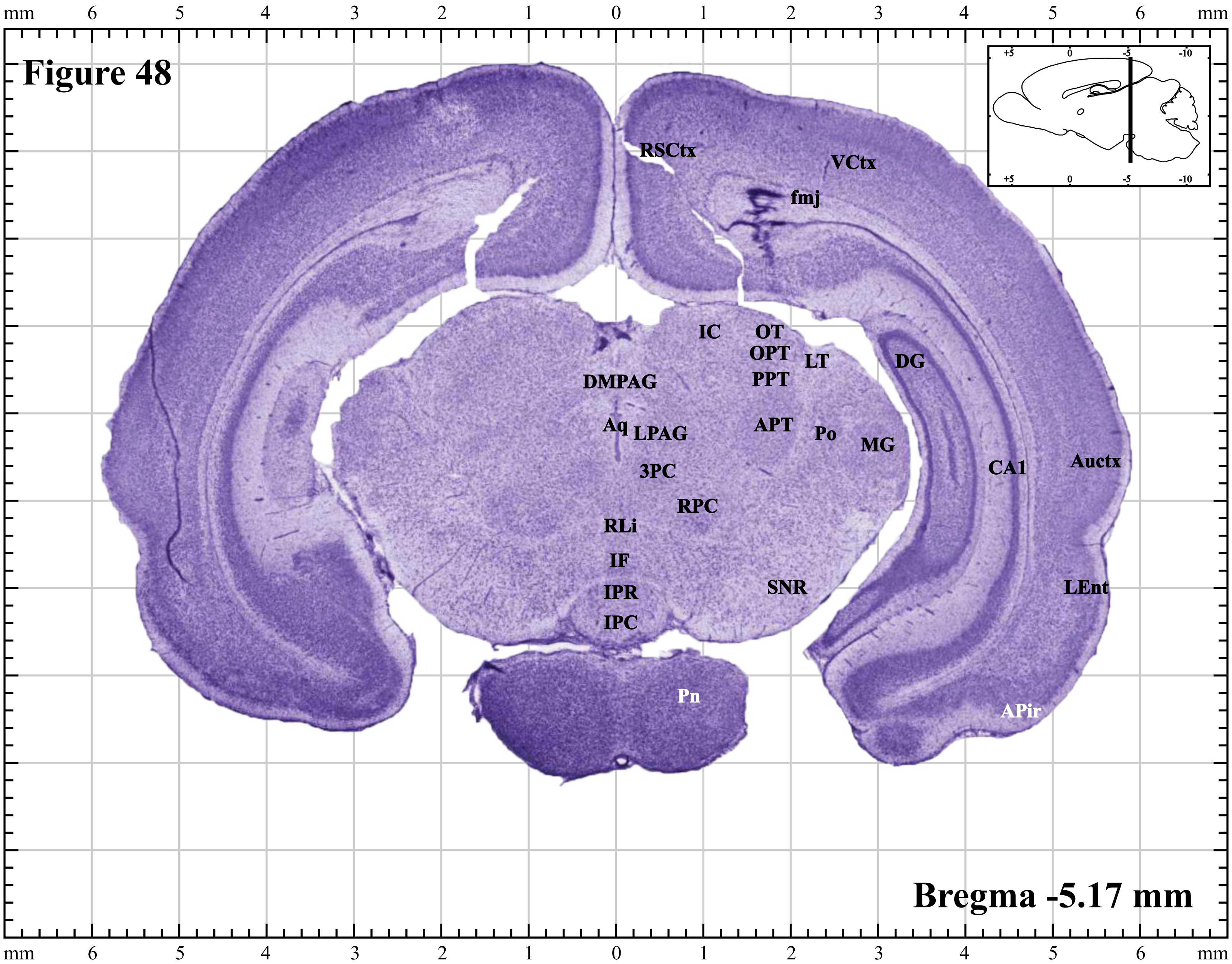

3PC oculomotor nucleus, parvicellular part
APir amygdalopiriform transition area
APT anterior pretectal nucleus
Aq aqueduct
Auctx auditory cortex
APT anterior pretectal nucleus
CA1 field CA1 of the hippocampus
DMPAG dorsomedial periaqueductal gray
DG dentate gyrus
fmj forceps major of the corpus callosum
IC inferior colliculus
IF interfascicular nucleus
IPC interpeduncular nucleus, caudal subnucleus
IPR interpeduncular nucleus, rostral subnucleus
LEnt lateral entorhinal cortex
LPAG lateral periaqueductal gray
LT lateral thalamus
MG medial geniculate nucleus
OT nucleus of the optic
OPT olivary pretectal nucleus
Po posterior thalamic nuclear group
Pn pontine nuclei
PPT posterior pretectal nucleus
RSCtx retrospenial cortex
RLi rostral linear nucleus of the raphe
RPC red nucleus, parvicellular part
SNR substantia nigra, reticular part
VCtx visual cortex

Figure 49

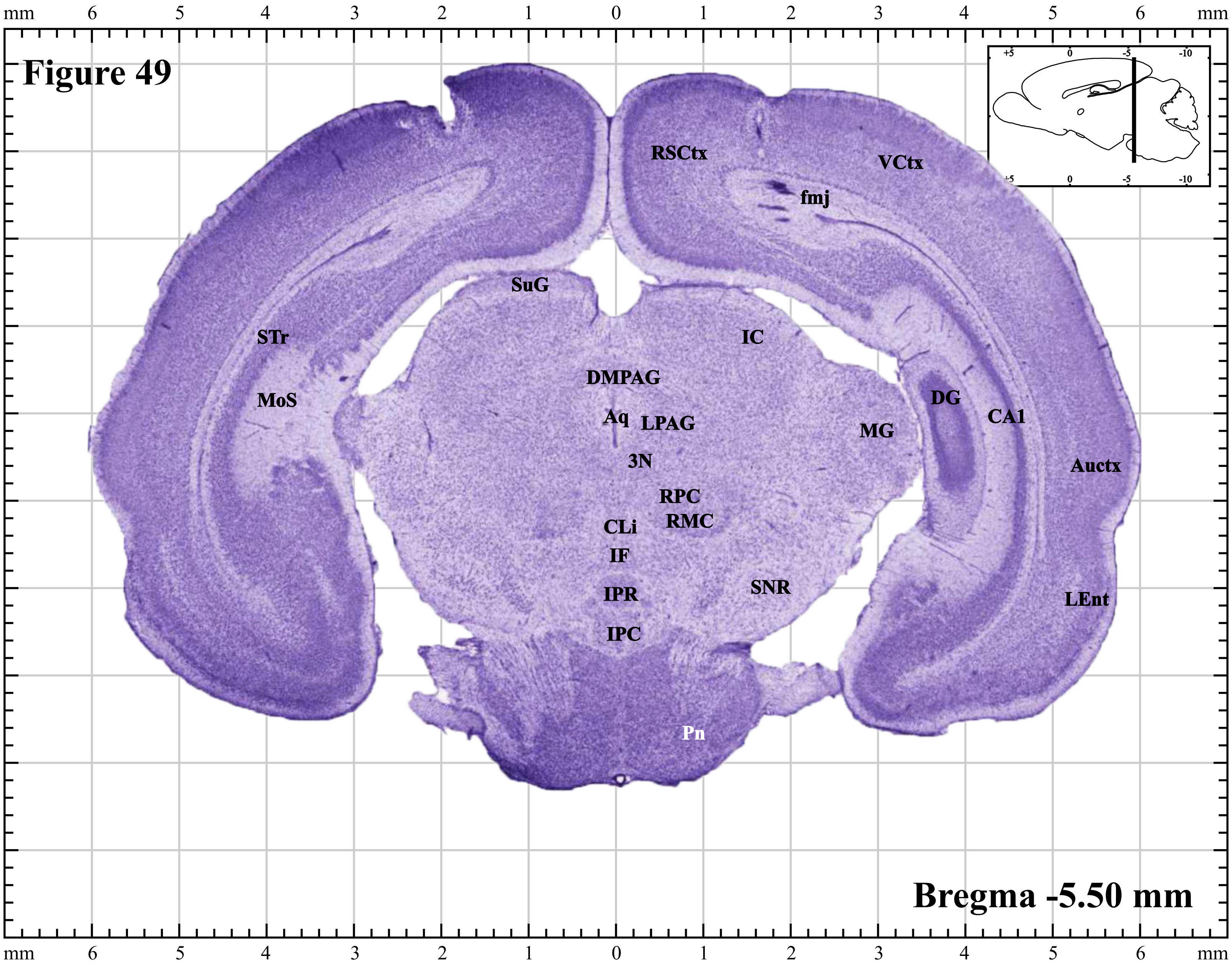

Bregma -5.50 mm

3N oculomotor nucleus
Aq aqueduct
Auctx auditory cortex
CLi caudal linear nucleus of the raphe
CA1 field CA1 of the hippocampus
DMPAG dorsomedial periaqueductal gray
DG dentate gyrus
fmj forceps major of the corpus callosum
IC inferior colliculus
IF interfascicular nucleus
IPC interpeduncular nucleus, caudal subnucleus
IPR interpeduncular nucleus, rostral subnucleus
LEnt lateral entorhinal cortex
LPAG lateral periaqueductal gray
MG medial geniculate nucleus
MoS molecular layer of the subiculum
Pn pontine nuclei
RSCtx retrospenial cortex
RMC red nucleus, magnocellular part
RPC red nucleus, parvicellular part
SuG superficial gray layer of the superior colliculus
SNR substantia nigra, reticular part
STr subiculum, transition area
VCtx visual cortex

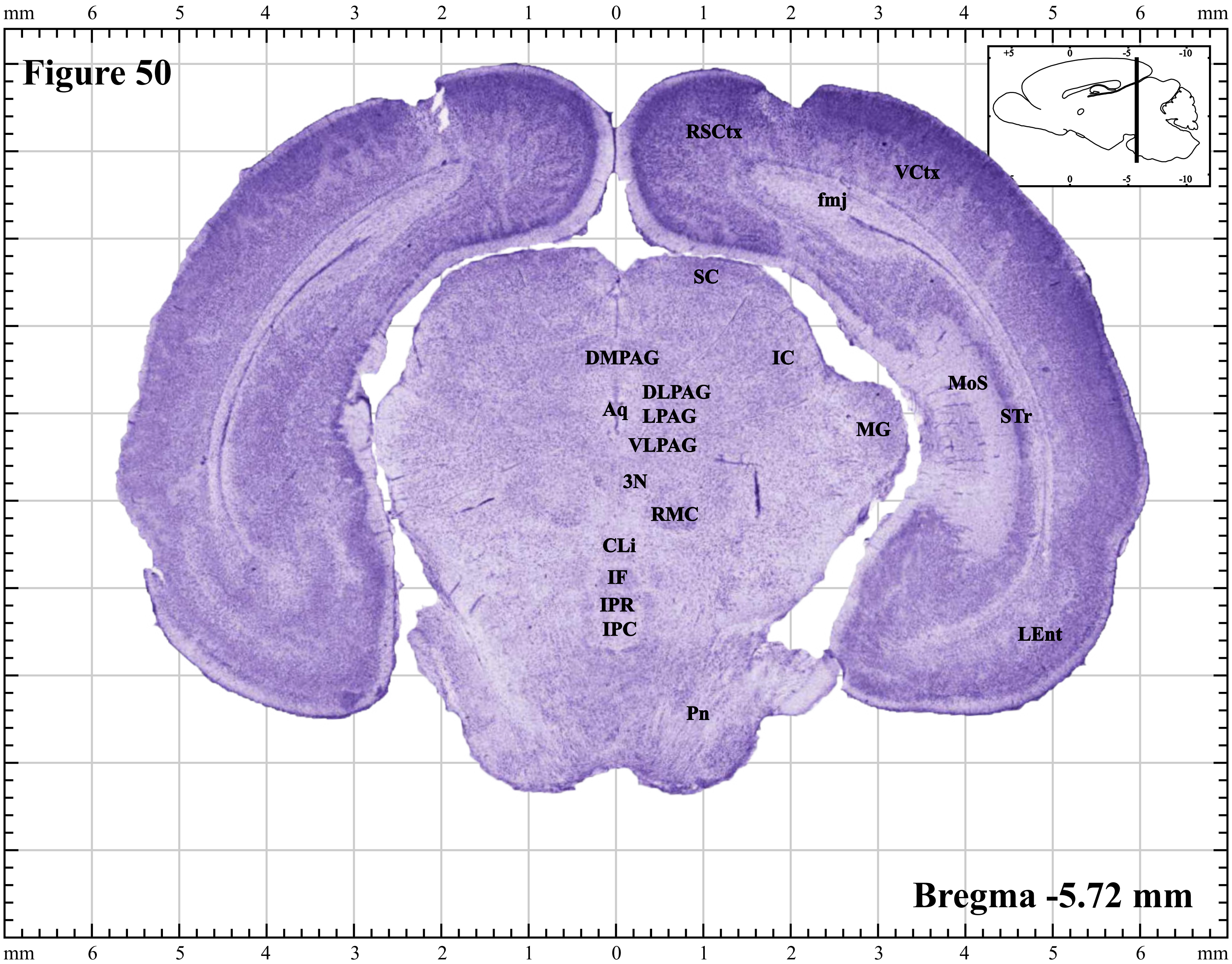

- **3N** oculomotor nucleus
- **Aq** aqueduct
- **CLi** caudal linear nucleus of the raphe
- **DMPAG** dorsomedial periaqueductal gray
- **DLPAG** dorsolateral periaqueductal gray
- **fmj** forceps major of the corpus callosum
- **IC** inferior colliculus
- **IPC** interpeduncular nucleus, caudal subnucleus
- **IF** interfascicular nucleus
- **IPC** interpeduncular nucleus, caudal subnucleus
- **IPR** interpeduncular nucleus, rostral subnucleus
- **LEnt** lateral entorhinal cortex
- **LPAG** lateral periaqueductal gray
- **MG** medial geniculate nucleus
- **MoS** molecular layer of the subiculum
- **Pn** pontine nuclei
- **RSCtx** retrospenial cortex
- **RMC** red nucleus, magnocellular part
- **STr** subiculum, transition area
- **SC** superior colliculus
- **VCtx** visual cortex
- **VLPAG** ventrolateral periaqueductal gray

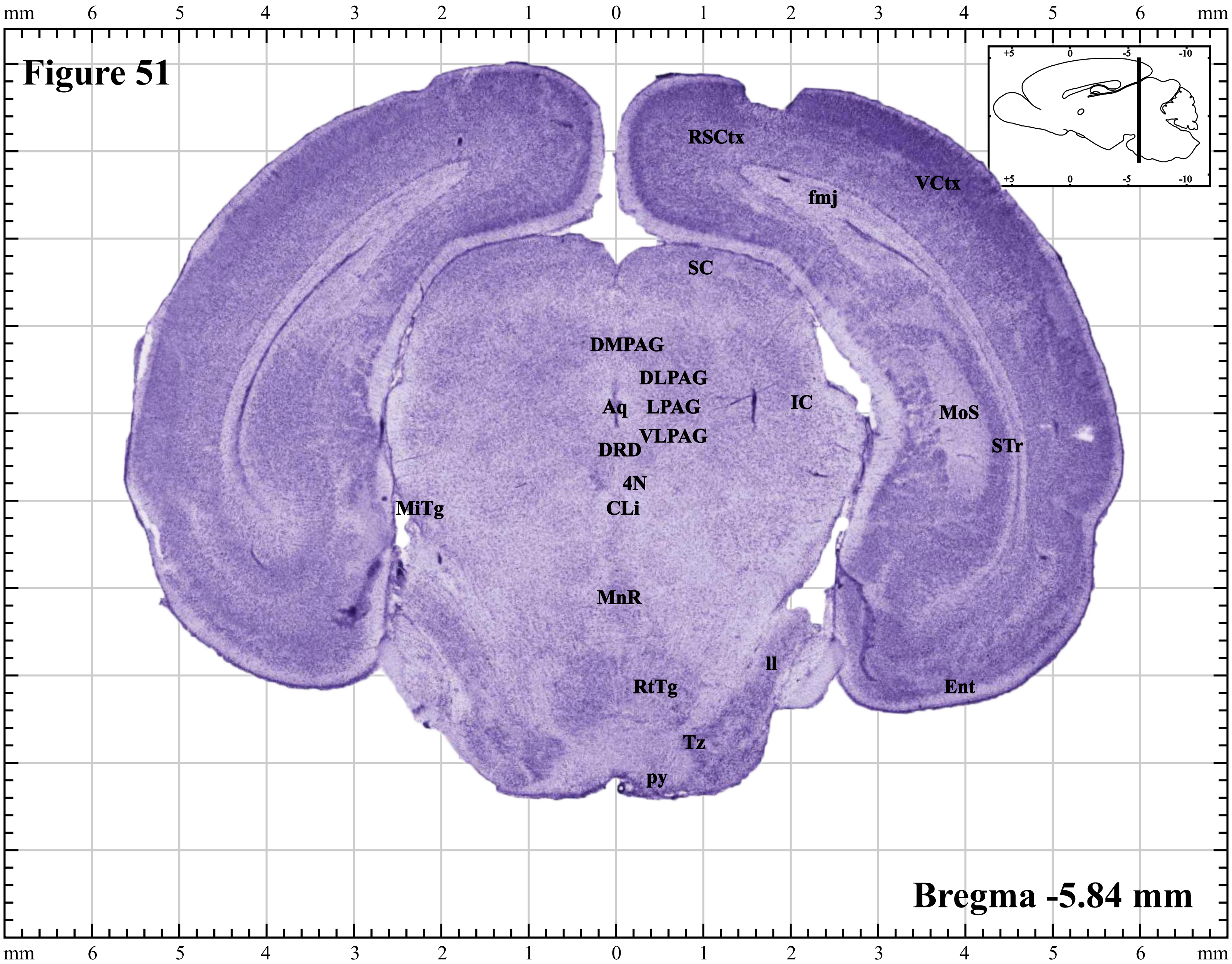

4N trochlear nucleus
Aq aqueduct
CLi caudal linear nucleus of the raphe
DMPAG dorsomedial periaqueductal gray
DLPAG dorsolateral periaqueductal gray
DRD dorsomedial hypothalamic nucleus, dorsal part
Ent entorhinal cortex
fmj forceps major of the corpus callosum
IC inferior colliculus
ll lateral lemniscus
LPAG lateral periaqueductal gray
MiTg microcellular tegmental nucleus
MnR median raphe nucleus
py pyramidal tract
MoS molecular layer of the subiculum
RSCtx retrospenial cortex
RtTg reticulotegmental nucleus of the pons
STr subiculum, transition area
SC superior colliculus
Tz nucleus of the trapezoid body
VCtx visual cortex
VLPAG ventrolateral periaqueductal gray

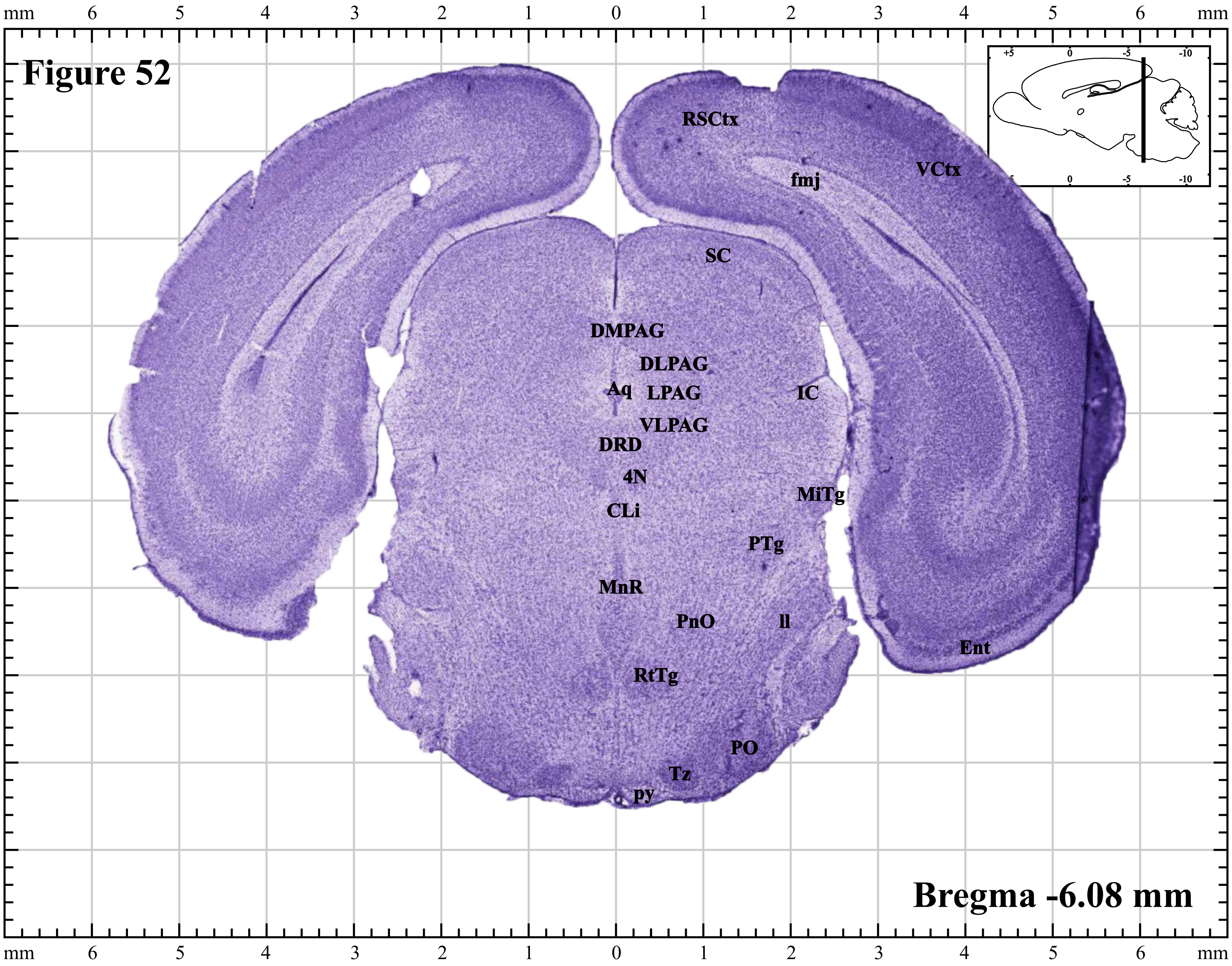

4N trochlear nucleus
Aq aqueduct
CLi caudal linear nucleus of the raphe
DMPAG dorsomedial periaqueductal gray
DLPAG dorsolateral periaqueductal gray
DRD dorsomedial hypothalamic nucleus, dorsal part
Ent entorhinal cortex
fmj forceps major of the corpus callosum
IC inferior colliculus
ll lateral lemniscus
LPAG lateral periaqueductal gray
MiTg microcellular tegmental nucleus
MnR median raphe nucleus
py pyramidal tract
PO paraolivary nucleus
PTg pedunculopontine tegmental nucleus
RtTg reticulotegmental nucleus of the pons
RSCtx retrospenial cortex
SC superior colliculus
Tz nucleus of the trapezoid body
VCtx visual cortex
VLPAG ventrolateral periaqueductal gray

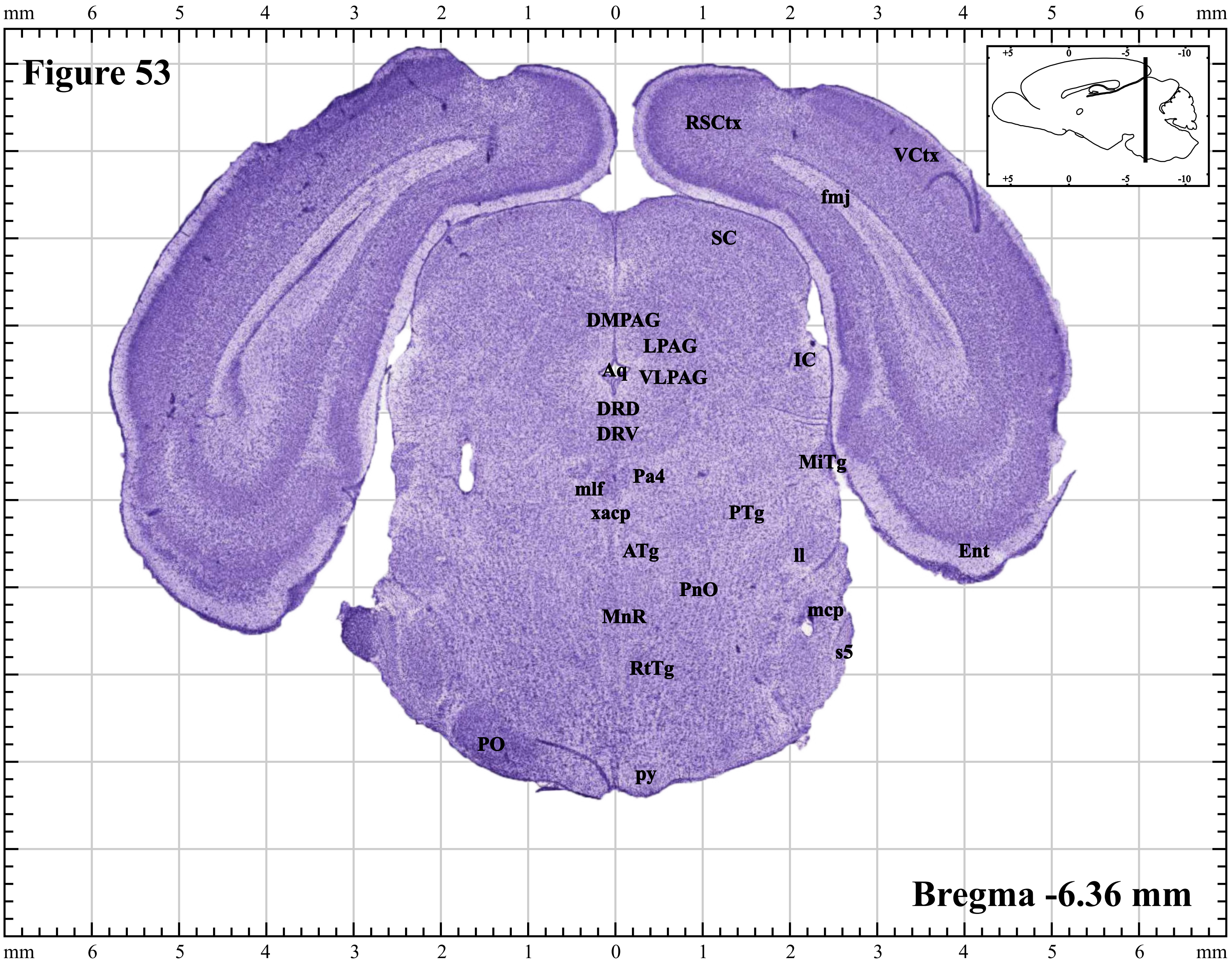

Figure 53

ATg anterior tegmental nucleus
Aq aqueduct
DMPAG dorsomedial periaqueductal gray
DRD dorsomedial hypothalamic nucleus, dorsal part
DRV dorsomedial hypothalamic nucleus, ventral part
Ent entorhinal cortex
fmj forceps major of the corpus callosum
IC inferior colliculus
ll lateral lemniscus
LPAG lateral periaqueductal gray
MnR median raphe nucleus
mlf medial longitudinal fasciculus
mcp middle cerebellar peduncle
MiTg microcellular tegmental nucleus
py pyramidal tract
PO paraolivary nucleus
PTg pedunculopontine tegmental nucleus
PnO pontine reticular nucleus, oral part
Pa4 paratrochlear nucleus
RtTg reticulotegmental nucleus of the pons
RSCtx retrospenial cortex
s5 sensory root of the trigeminal nerve
SC superior colliculus
VCtx visual cortex
VLPAG ventrolateral periaqueductal gray
xscp decussation of the superior cerebellar peduncle

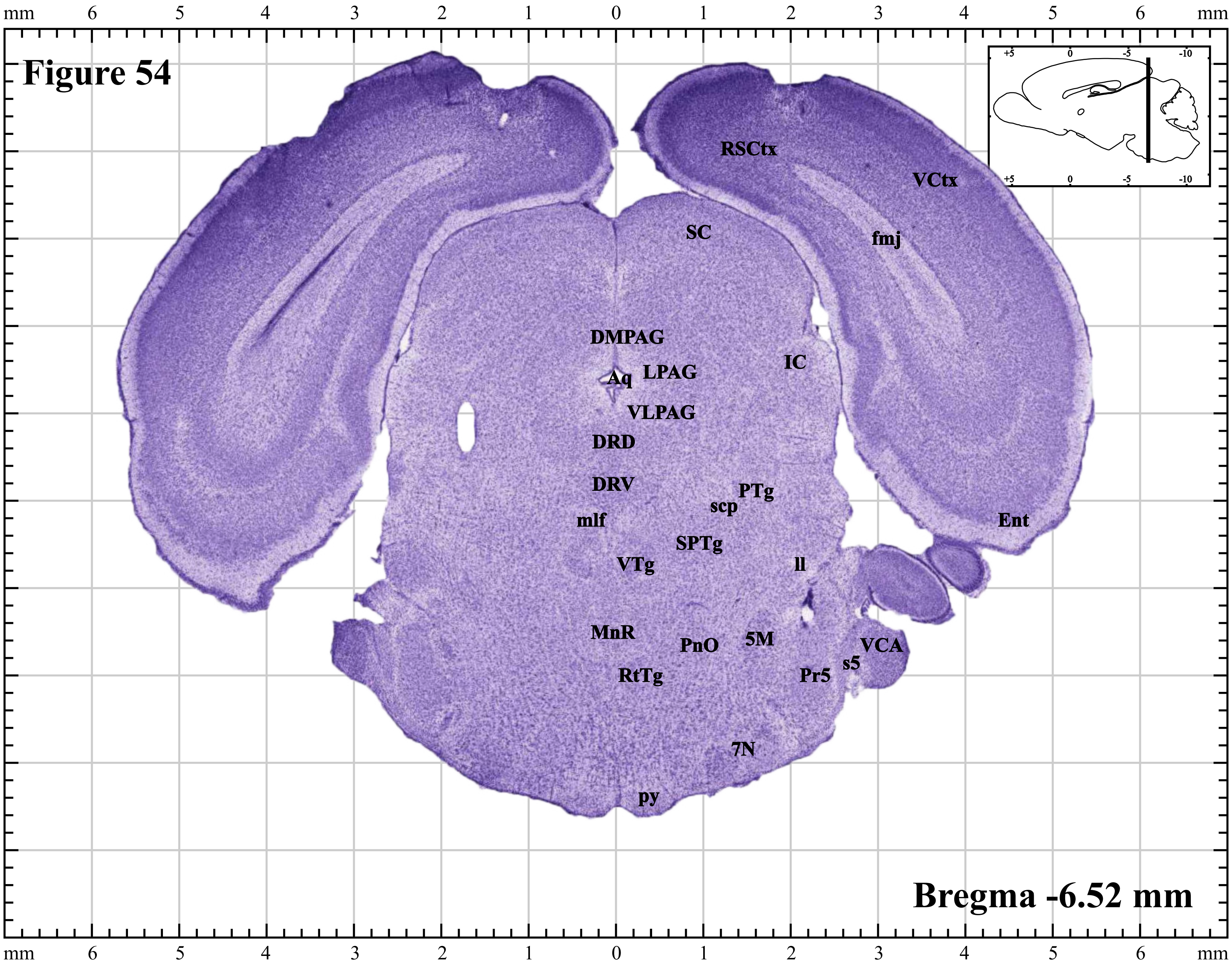

5M motor trigeminal nucleus
7N facial nuclues
Aq aqueduct
DMPAG dorsomedial periaqueductal gray
DRD dorsomedial hypothalamic nucleus, dorsal part
DRV dorsomedial hypothalamic nucleus, ventral part
Ent entorhinal cortex
fmj forceps major of the corpus callosum
IC inferior colliculus
ll lateral lemniscus
LPAG lateral periaqueductal gray
mlf medial longitudinal fasciculus
MnR median raphe nucleus
py pyramidal tract
PnO pontine reticular nucleus, oral part
PTg pedunculopontine tegmental nucleus
Pr5 principal sensory trigeminal nucleus
RSCtx retrospenial cortex
RtTg reticulotegmental nucleus of the pons
s5 sensory root of the trigeminal nerve
scp superior cerebellar peduncle
SC superior colliculus
SPTg subpeduncular tegmental nucleus
VCtx visual cortex
VLPAG ventrolateral periaqueductal gray
VTg ventral tegmental nucleus
VCA ventral cochlear nucleus, anterior part

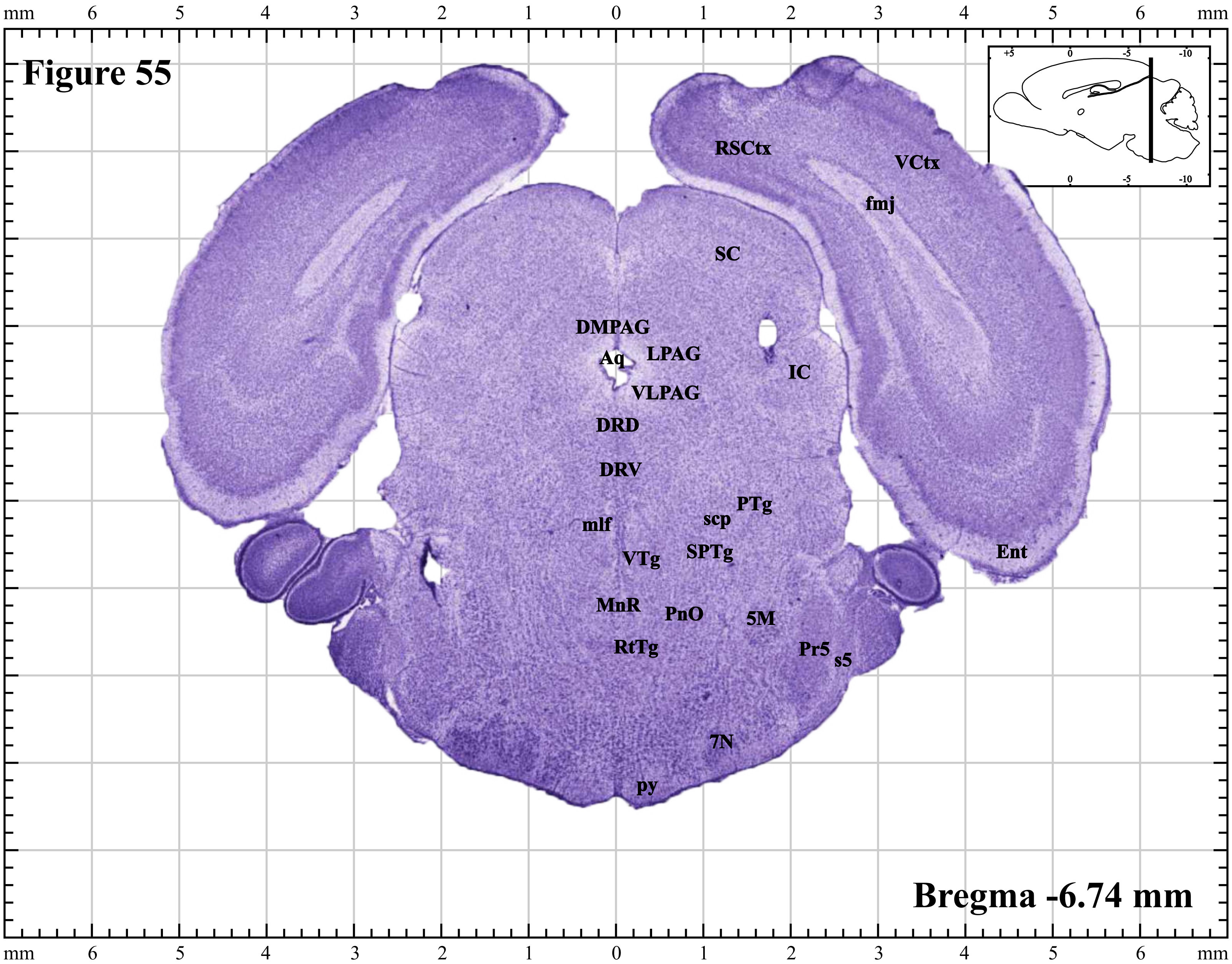

5M motor trigeminal nucleus
7N facial nuclues
Aq aqueduct
DMPAG dorsomedial periaqueductal gray
DRD dorsomedial hypothalamic nucleus, dorsal part
DRV dorsomedial hypothalamic nucleus, ventral part
Ent entorhinal cortex
fmj forceps major of the corpus callosum
IC inferior colliculus
LPAG lateral periaqueductal gray
MnR median raphe nucleus
mlf medial longitudinal fasciculus
py pyramidal tract
PTg pedunculopontine tegmental nucleus
Pr5 principal sensory trigeminal nucleus
PnO pontine reticular nucleus, oral part
RSCtx retrospenial cortex
RtTg reticulotegmental nucleus of the pons
s5 sensory root of the trigeminal nerve
scp superior cerebellar peduncle
SC superior colliculus
SPTg subpeduncular tegmental nucleus
VCtx visual cortex
VLPAG ventrolateral periaqueductal gray
VTg ventral tegmental nucleus

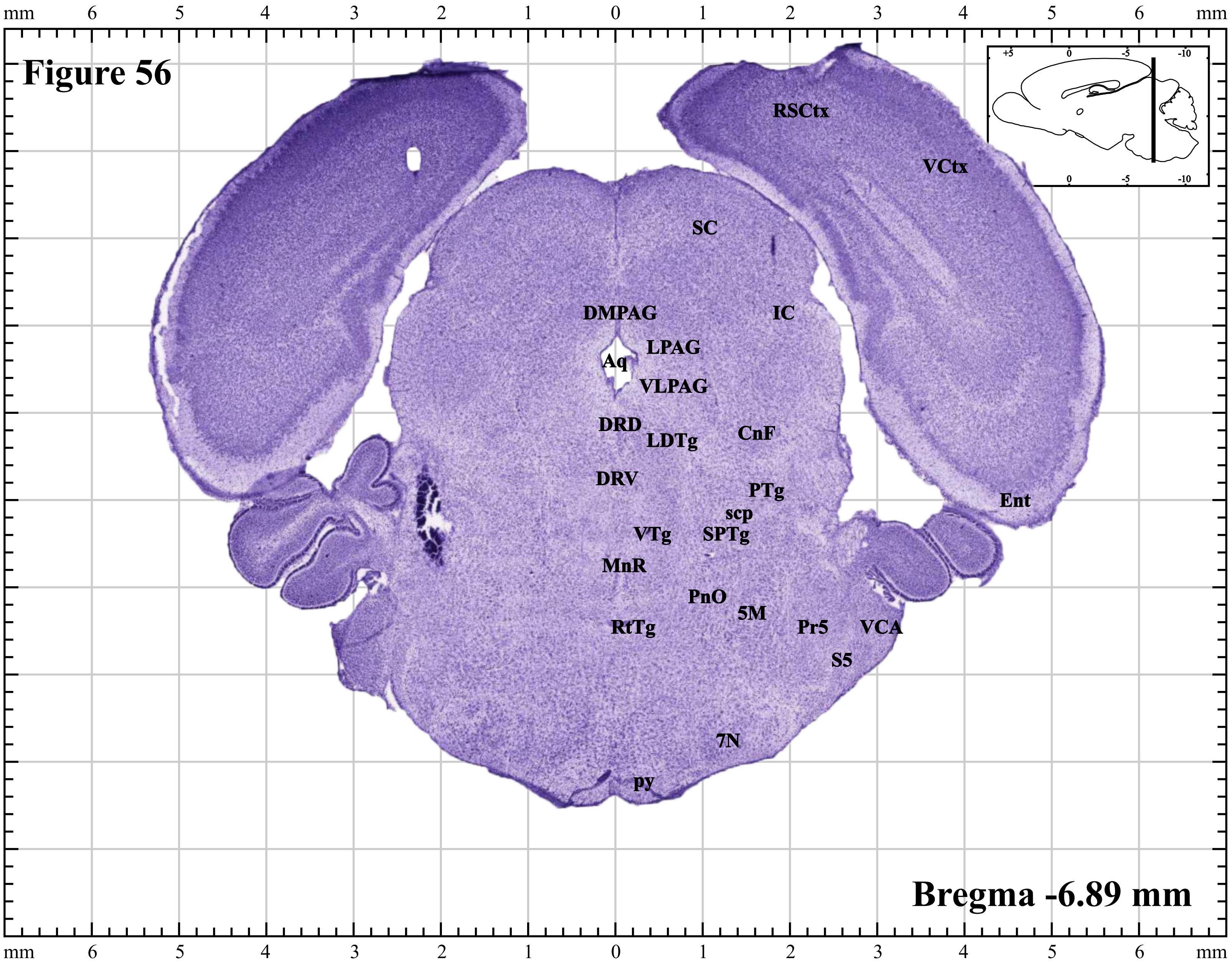

5M motor trigeminal nucleus
7N facial nuclues
Aq aqueduct
CnF cuneiform nucleus
DMPAG dorsomedial periaqueductal gray
DRD dorsomedial hypothalamic nucleus, dorsal part
DRV dorsomedial hypothalamic nucleus, ventral part
Ent entorhinal cortex
IC inferior colliculus
LDTg laterodorsal tegmental nucleus
LPAG lateral periaqueductal gray
MnR median raphe nucleus
py pyramidal tract
PnO pontine reticular nucleus, oral part
Pr5 principal sensory trigeminal nucleus
PTg pedunculopontine tegmental nucleus
RSCtx retrospenial cortex
RtTg reticulotegmental nucleus of the pons
s5 sensory root of the trigeminal nerve
scp superior cerebellar peduncle
SC superior colliculus
SPTg subpeduncular tegmental nucleus
VCtx visual cortex
VCA ventral cochlear nucleus, anterior part
VLPAG ventrolateral periaqueductal gray
VTg ventral tegmental nucleus

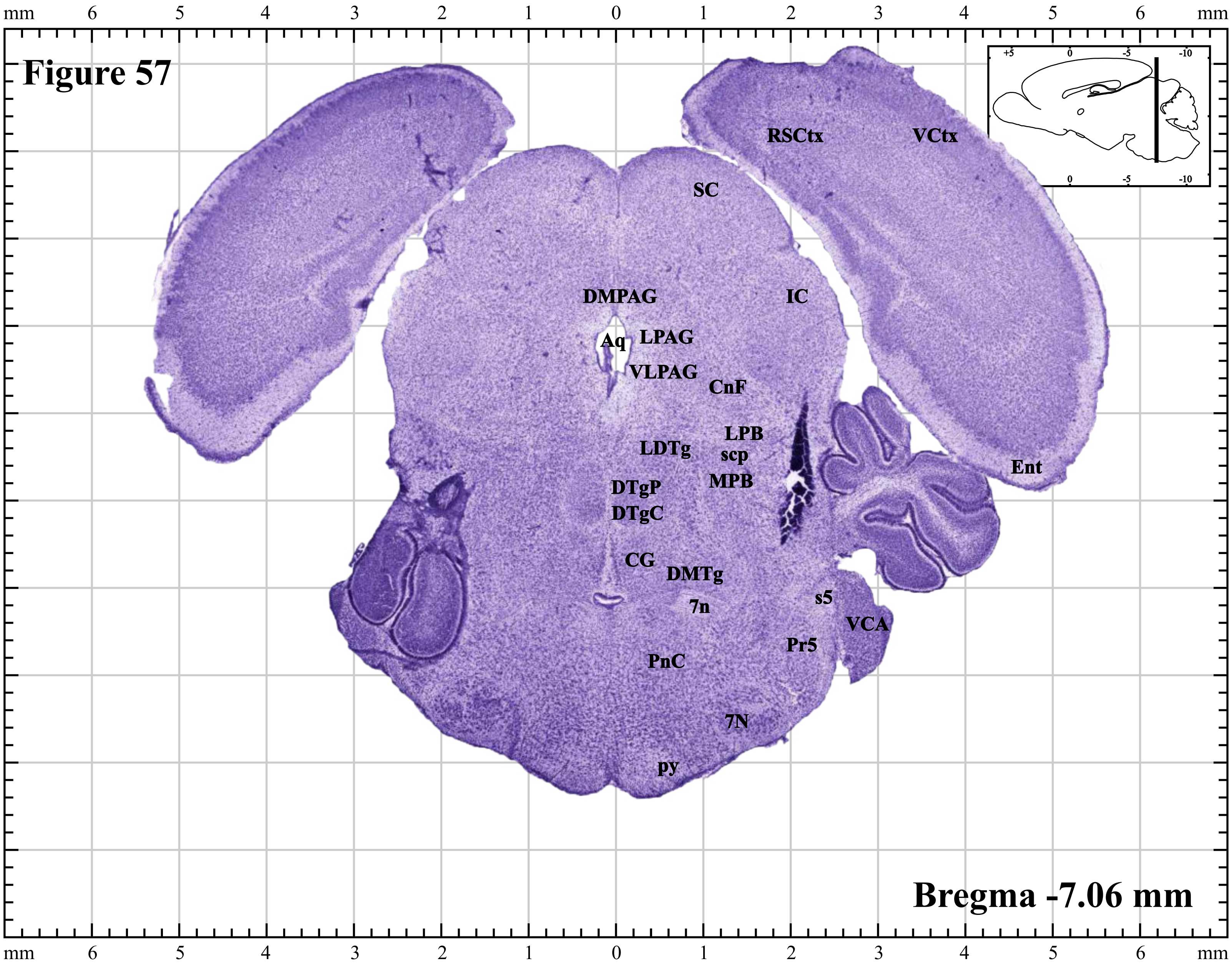

7n facial nerve
7N facial nuclues
Aq aqueduct
CG central gray
CnF cuneiform nucleus
DMPAG dorsomedial periaqueductal gray
DTgC dorsal tegmental nucleus, central part
DTgP dorsal tegmental nucleus, pericentral part
DMTg dorsomedial tegmental area
Ent entorhinal cortex
IC inferior colliculus
LDTg laterodorsal tegmental nucleus
LPAG lateral periaqueductal gray
LPB lateral parabrachial nucleus
MPB medial parabrachial nucleus
py pyramidal tract
PnC pontine reticular nucleus,caudal part
Pr5 principal sensory trigeminal nucleus
RSCtx retrospenial cortex
s5 sensory root of the trigeminal nerve
scp superior cerebellar peduncle
SC superior colliculus
VLPAG ventrolateral periaqueductal gray
VCtx visual cortex
VCA ventral cochlear nucleus, anterior part

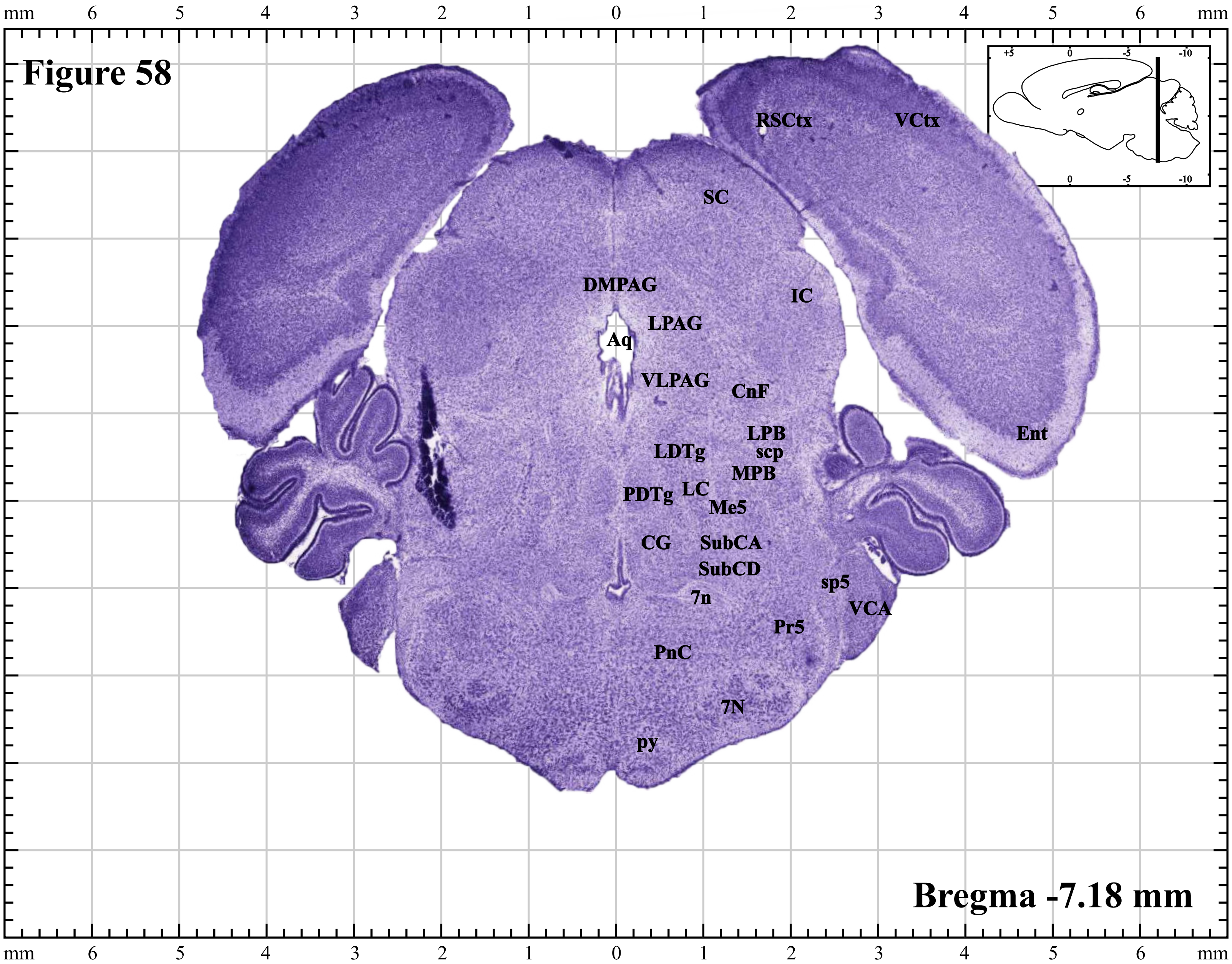

Aq aqueduct
7n facial nerve
7N facial nuclues
CG central gray
CnF cuneiform nucleus
DMPAG dorsomedial periaqueductal gray
Ent entorhinal cortex
IC inferior colliculus
LDTg laterodorsal tegmental nucleus
LC locus coeruleus
LPAG lateral periaqueductal gray
LPB lateral parabrachial nucleus
MPB medial parabrachial nucleus
Me5 mesencephalic trigeminal nucleus
py pyramidal tract
PDTg posterodorsal tegmental nucleus
Pr5 principal sensory trigeminal nucleus
PnC pontine reticular nucleus, caudal part
RSCtx retrospenial cortex
scp superior cerebellar peduncle
sp5 spinal trigeminal tract
SC superior colliculus
SubCA subcoeruleus nucleus, alpha part
SubCD subcoeruleus nucleus, dorsal part
VCA ventral cochlear nucleus, anterior part
VLPAG ventrolateral periaqueductal gray
VCtx visual cortex

Figure 59

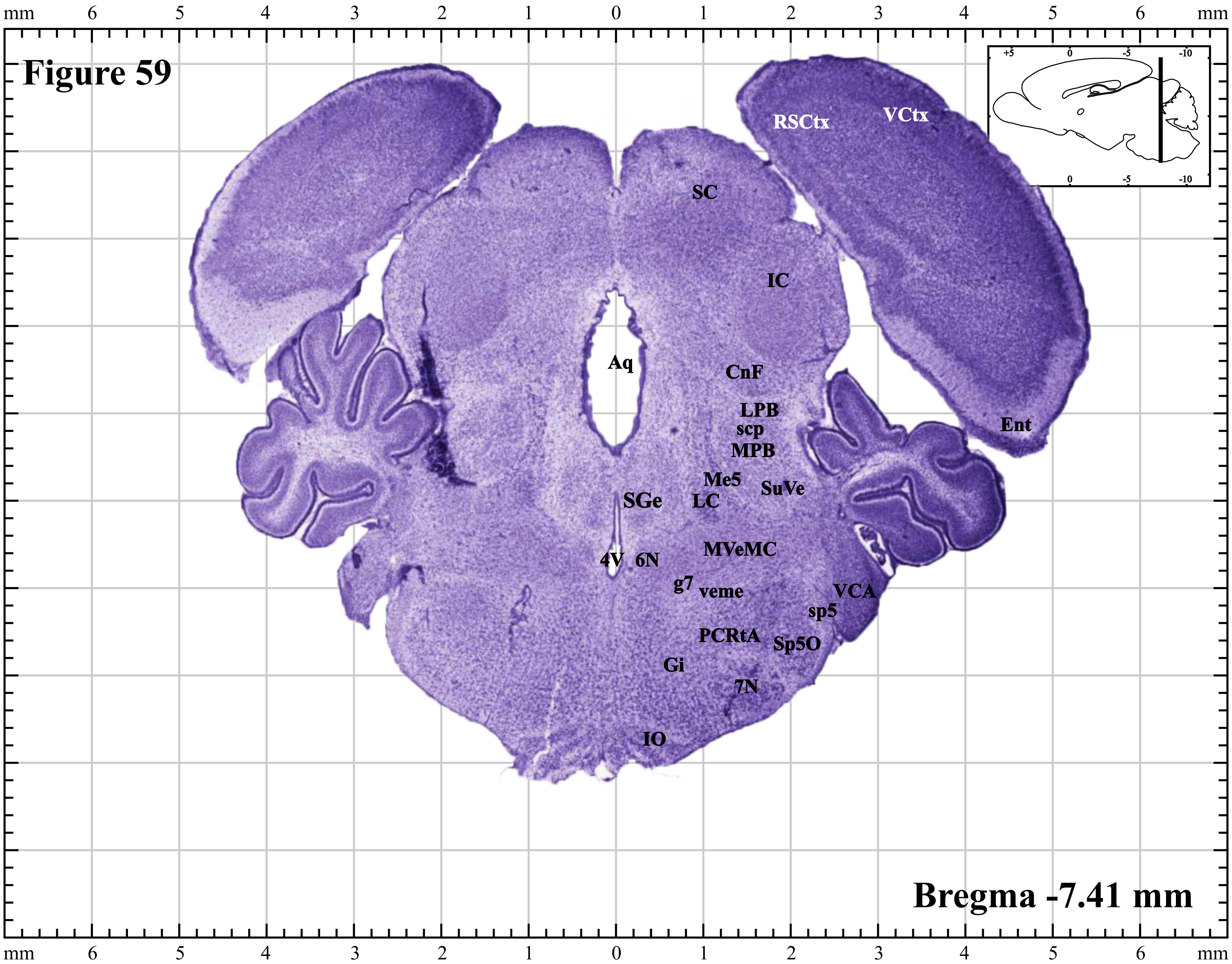

Bregma -7.41 mm

Aq aqueduct
4V 4th ventricle
6N abducens nucleus
7N facial nuclues
CnF cuneiform nucleus
Ent entorhinal cortex
g7 genu of the facial nerve
Gi granular insular cortex
IC inferior colliculus
IO inferior olive
LC locus coeruleus
LPB lateral parabrachial nucleus
MPB medial parabrachial nucleus
Me5 mesencephalic trigeminal nucleus
MVeMC medial vestibular nucleus, magnocellular part
PCRtA parvicellular reticular nucleus, alpha part
RSCtx retrospenial cortex
sp5 spinal trigeminal tract
SC superior colliculus
scp superior cerebellar peduncle
SGe supragenual nucleus
SuVe superior vestibular nucleus
Sp5O spinal trigeminal nucleus, oral part
veme vestibulomesencephalic tract
VCA ventral cochlear nucleus, anterior part
VCtx visual cortex

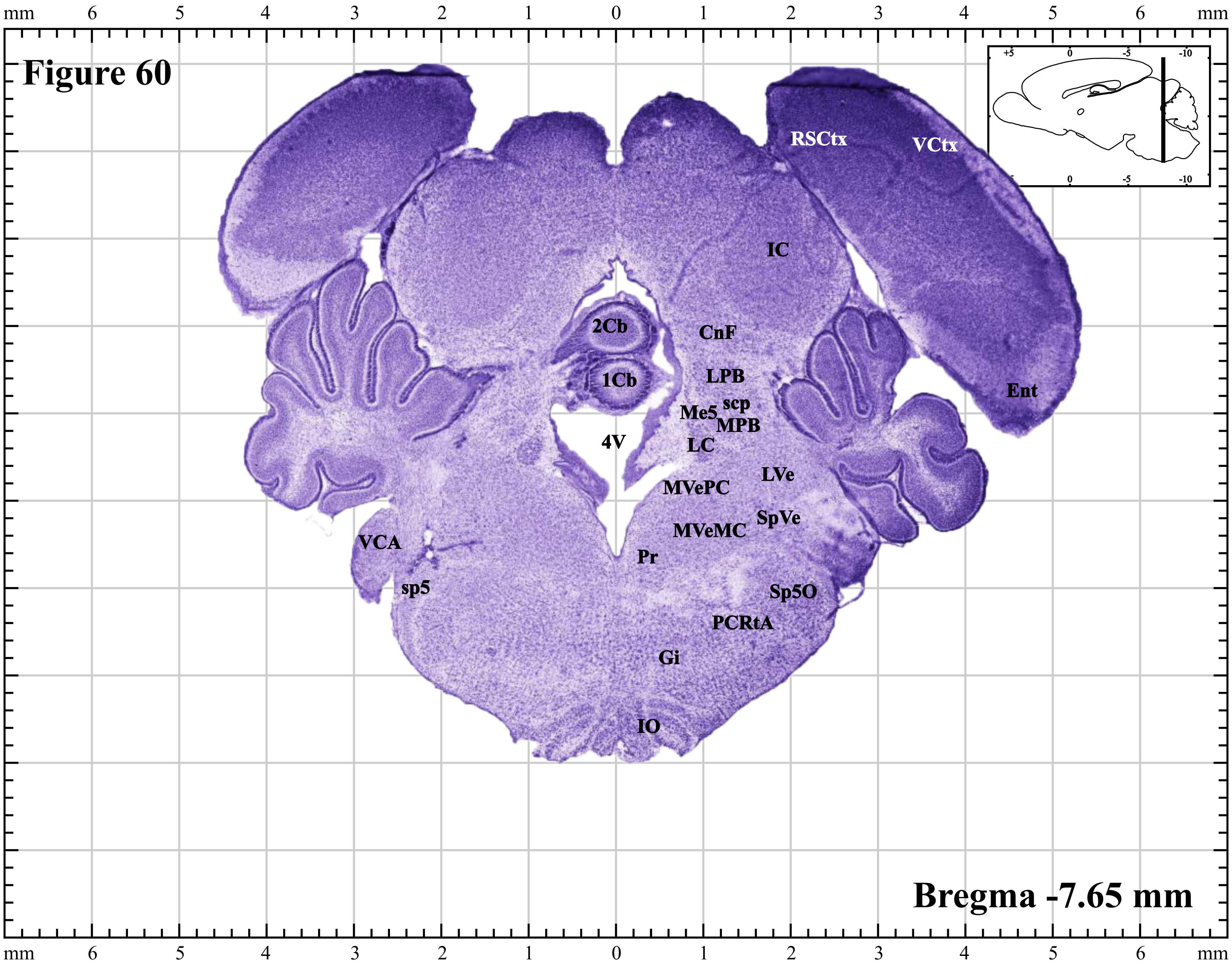

1Cb 1st cerebellar lobule (lingula)
2Cb 2nd cerebellar lobule
4V 4th ventricle
CnF cuneiform nucleus
Ent entorhinal cortex
Gi granular insular cortex
IO inferior olive
IC inferior colliculus
LC locus coeruleus
LVe lateral vestibular nucleus
LPB lateral parabrachial nucleus
Me5 mesencephalic trigeminal nucleus
MPB medial parabrachial nucleus
MVeMC medial vestibular nucleus, magnocellular part
MVePC medial vestibular nucleus, parvicellular part
PCRtA parvicellular reticular nucleus, alpha part
Pr prepositus nucleus
RSCtx retrospenial cortex
scp superior cerebellar peduncle
sp5 spinal trigeminal tract
SpVe spinal vestibular nucleus
Sp5O spinal trigeminal nucleus, oral part
VCA ventral cochlear nucleus, anterior part
VCtx visual cortex

Figure 61

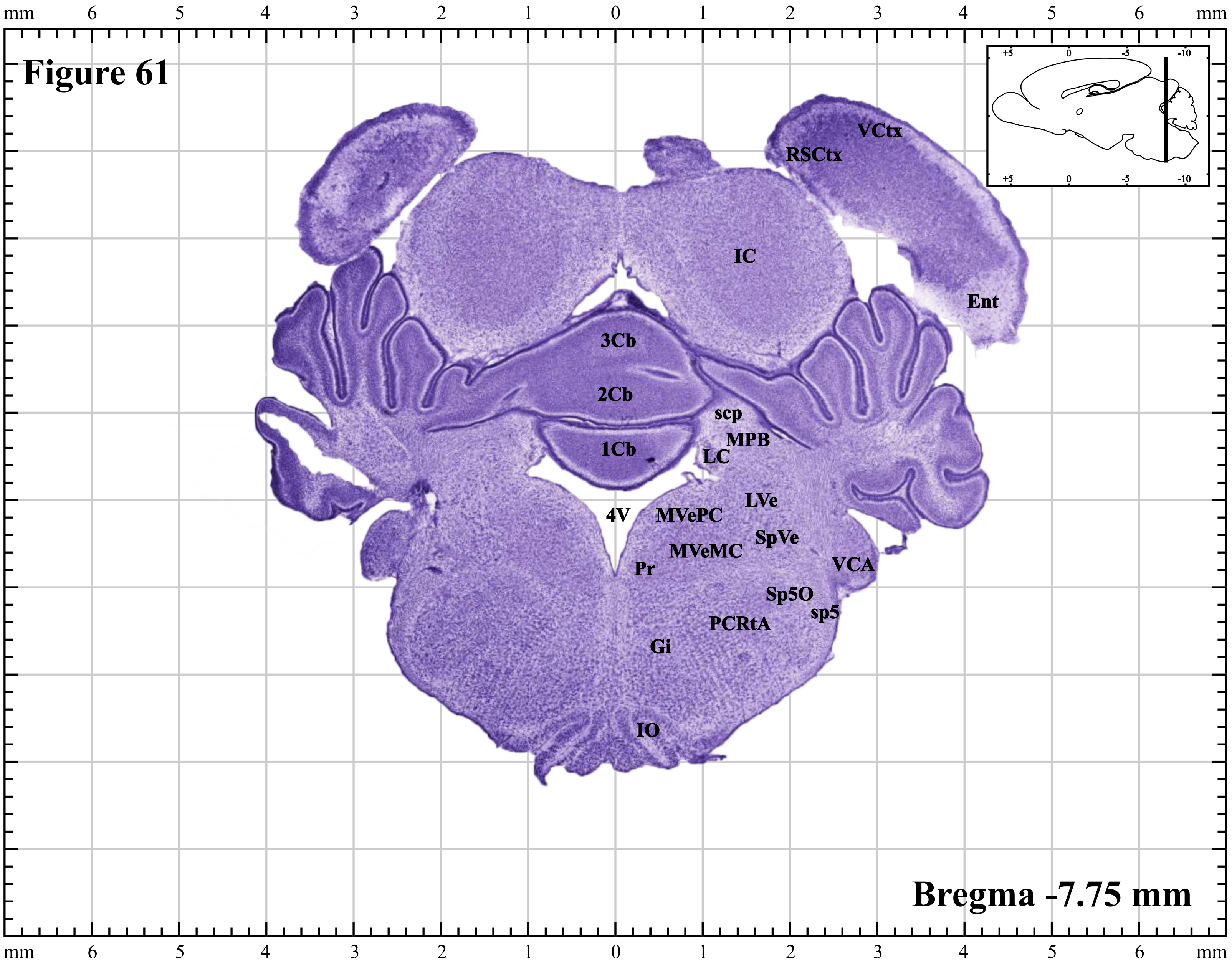

Bregma -7.75 mm

1Cb 1st cerebellar lobule (lingula)
2Cb 2nd cerebellar lobule
3Cb 3rd cerebellar lobule
4V 4th ventricle
7N facial nuclues
Ent entorhinal cortex
Gi granular insular cortex
IO inferior olive
IC inferior colliculus
LC locus coeruleus
LVe lateral vestibular nucleus
Me5 mesencephalic trigeminal nucleus
MPB medial parabrachial nucleus
MVeMC medial vestibular nucleus, magnocellular part
MVePC medial vestibular nucleus, parvicellular part
RSCtx retrospenial cortex
PCRtA parvicellular reticular nucleus, alpha part
Pr prepositus nucleus
scp superior cerebellar peduncle
sp5 spinal trigeminal tract
SpVe spinal vestibular nucleus
Sp5O spinal trigeminal nucleus, oral part
VCA ventral cochlear nucleus, anterior part
VCtx visual cortex

Figure 62

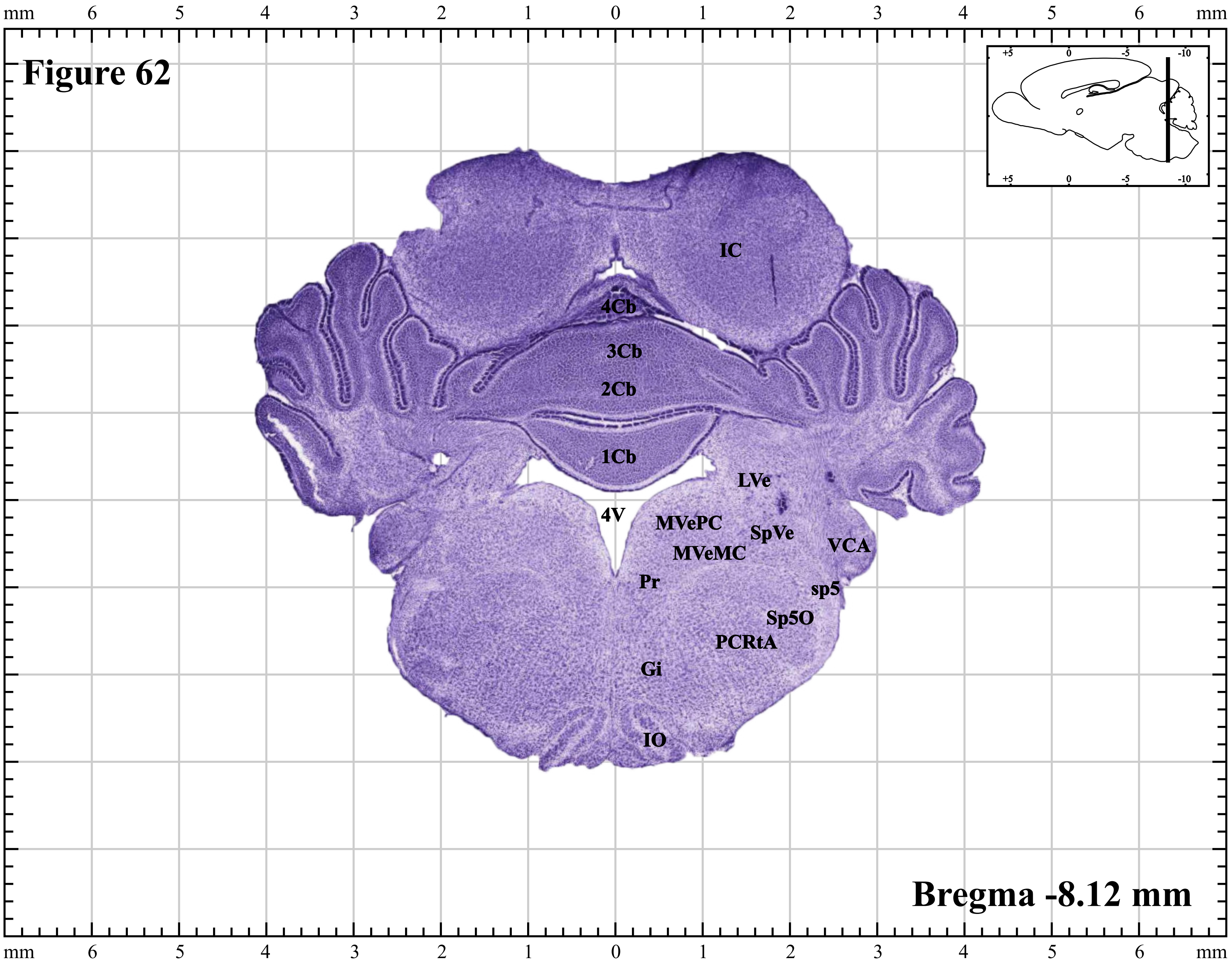

1Cb 1st cerebellar lobule (lingula)
2Cb 2nd cerebellar lobule
3Cb 3rd cerebellar lobule
4Cb 4th cerebellar lobule
4V 4th ventricle
7N facial nuclues
Gi granular insular cortex
IO inferior olive
IC inferior colliculus
LVe lateral vestibular nucleus
Me5 mesencephalic trigeminal nucleus
MVeMC medial vestibular nucleus, magnocellular part
MVePC medial vestibular nucleus, parvicellular part
PCRtA parvicellular reticular nucleus, alpha part
Pr prepositus nucleus
SpVe spinal vestibular nucleus
sp5 spinal trigeminal tract
VCA ventral cochlear nucleus, anterior part

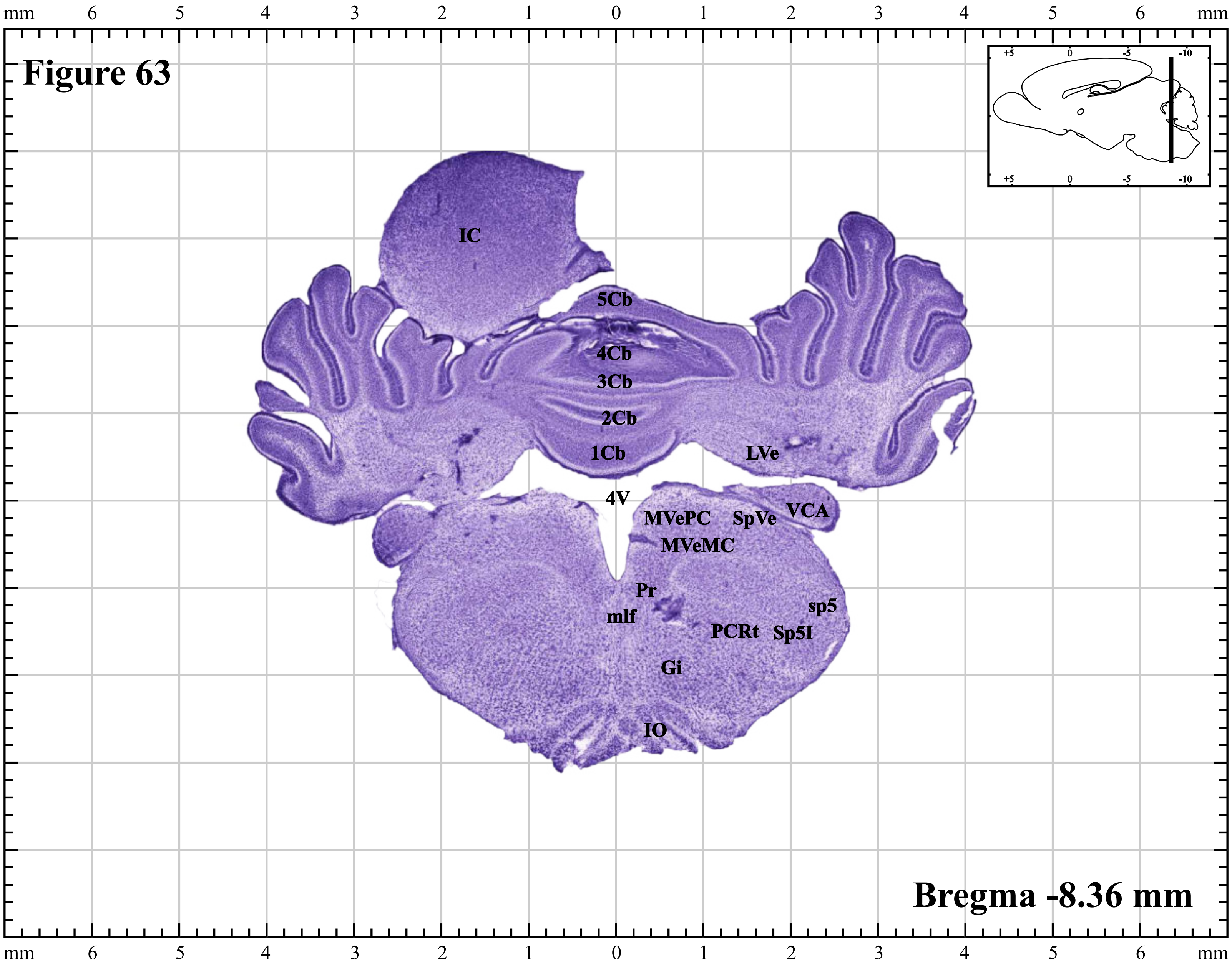

1Cb 1st cerebellar lobule (lingula)
2Cb 2nd cerebellar lobule
3Cb 3rd cerebellar lobule
4Cb 4th cerebellar lobule
4V 4th ventricle
5Cb 5th cerebellar lobule
7N facial nuclues
Gi granular insular cortex
IO inferior olive
IC inferior colliculus
LVe lateral vestibular nucleus
Me5 mesencephalic trigeminal nucleus
MVeMC medial vestibular nucleus, magnocellular part
MVePC medial vestibular nucleus, parvicellular part
mlf medial longitudinal fasciculus
PCRt parvicellular reticular nucleus
Pr prepositus nucleus
SpVe spinal vestibular nucleus
Sp5I spinal trigeminal nucleus, interpolar part
sp5 spinal trigeminal tract
VCA ventral cochlear nucleus, anterior part

Figure 64

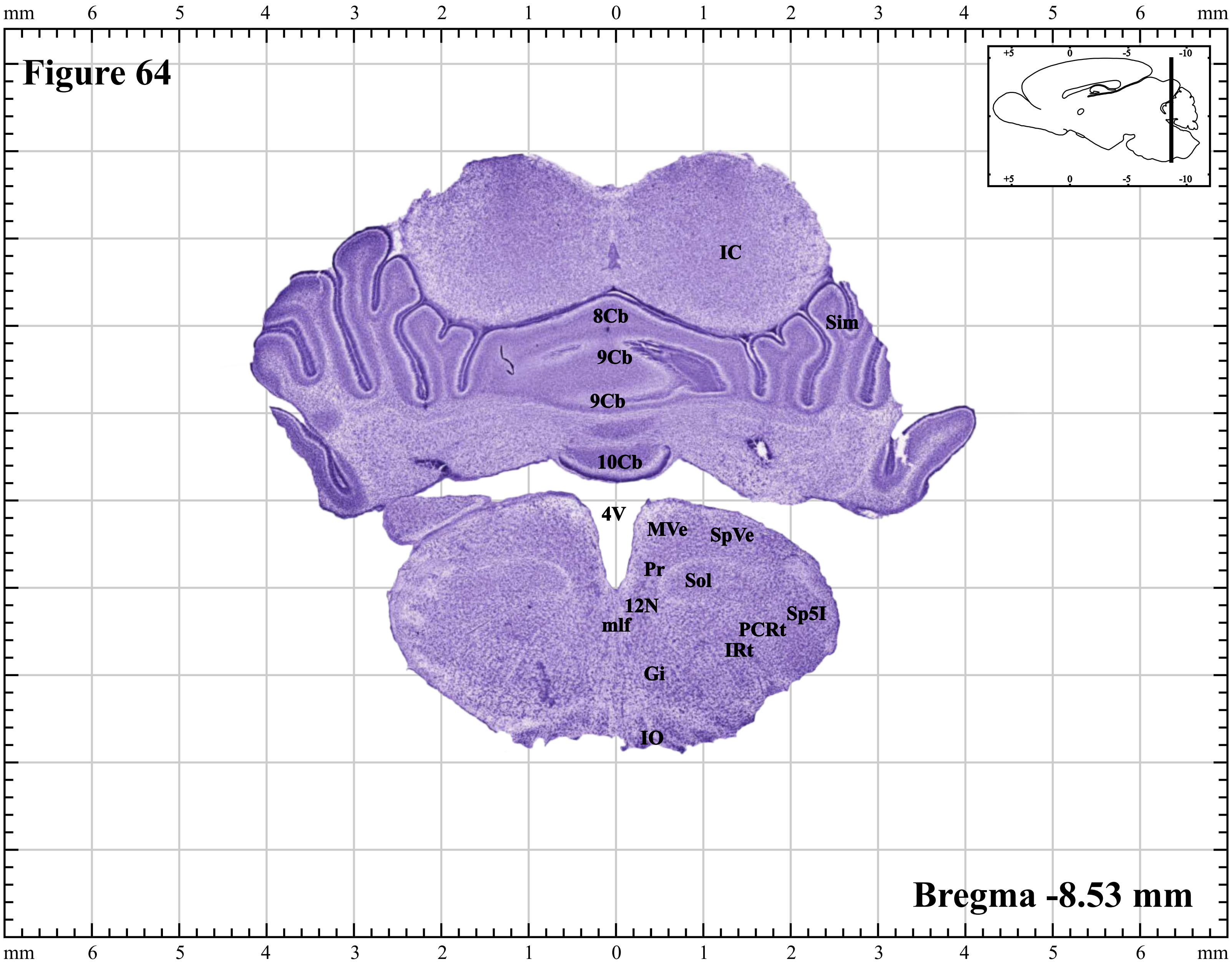

Bregma -8.53 mm

4V 4th ventricle
8Cb 8th cerebellar lobule
9Cb 9th cerebellar lobules
10Cb 10th cerebellar lobule
12N hypoglossal nucleus
Gi granular insular cortex
IO inferior olive
IRt intermediate reticular nucleus
IC inferior colliculus
mlf medial longitudinal fasciculus
MVe medial vestibular nucleus
PCRt parvicellular reticular nucleus
Pr prepositus nucleus
Sol nucleus of the solitary tract
Sim simple lobule
SpVe spinal vestibular nucleus
Sp5I spinal trigeminal nucleus, interpolar part

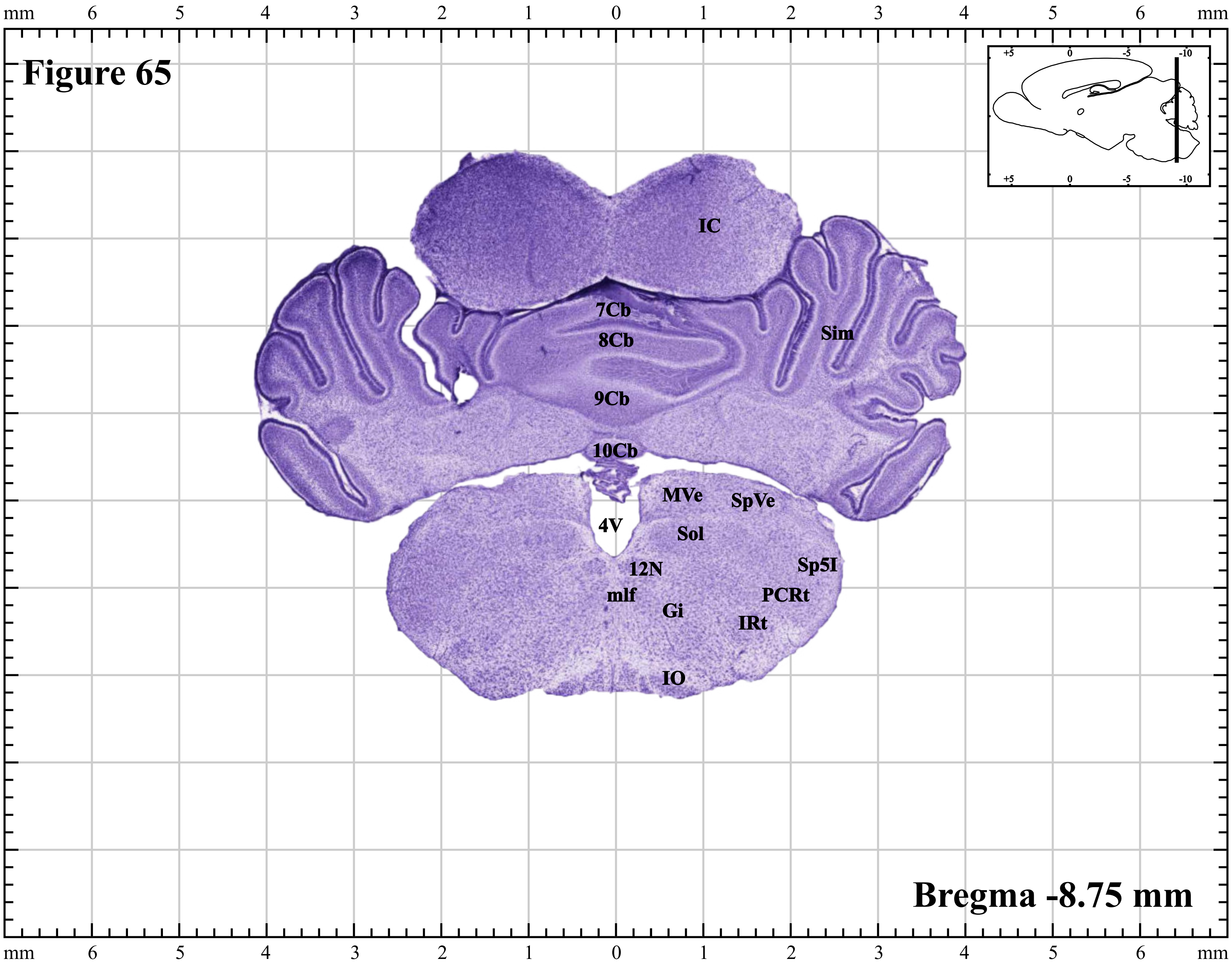

4V 4th ventricle
7Cb 7th cerebellar lobule (lingula)
8Cb 8th cerebellar lobule
9Cb 9th cerebellar lobules
10Cb 10th cerebellar lobule
12N hypoglossal nucleus
Gi granular insular cortex
IO inferior olive
IRt intermediate reticular nucleus
IC inferior colliculus
mlf medial longitudinal fasciculus
MVe medial vestibular nucleus
PCRt parvicellular reticular nucleus
Sol nucleus of the solitary tract
Sim simple lobule
SpVe spinal vestibular nucleus
Sp5I spinal trigeminal nucleus, interpolar part

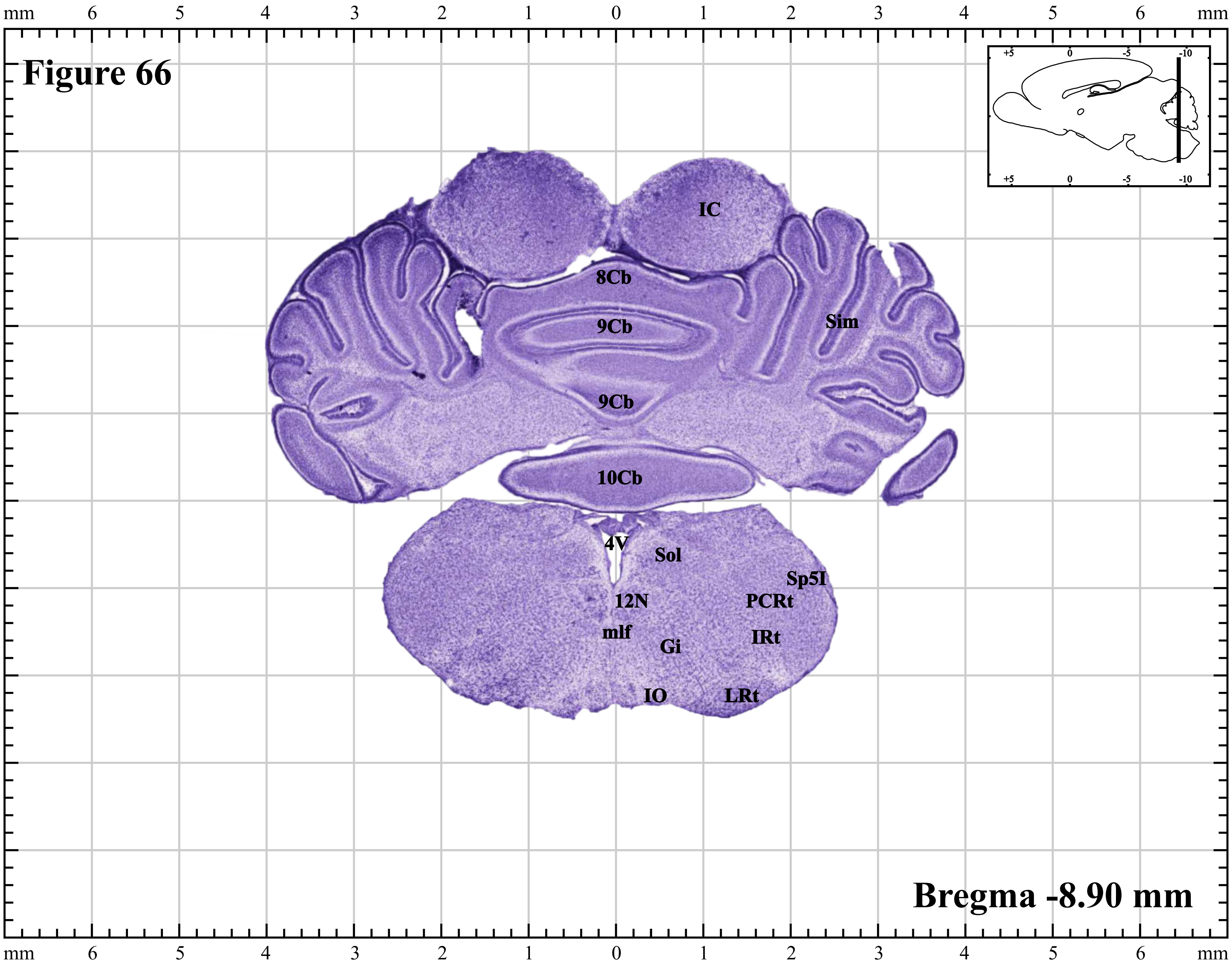

4V 4th ventricle
8Cb 8th cerebellar lobule
9Cb 9th cerebellar lobules
10Cb 10th cerebellar lobule
12N hypoglossal nucleus
Gi granular insular cortex
IO inferior olive
IC inferior colliculus
IRt intermediate reticular nucleus
LRt lateral reticular nucleus
mlf medial longitudinal fasciculus
PCRt parvicellular reticular nucleus
Sol nucleus of the solitary tract
Sim simple lobule
Sp5I spinal trigeminal nucleus, interpolar part

Figure 67

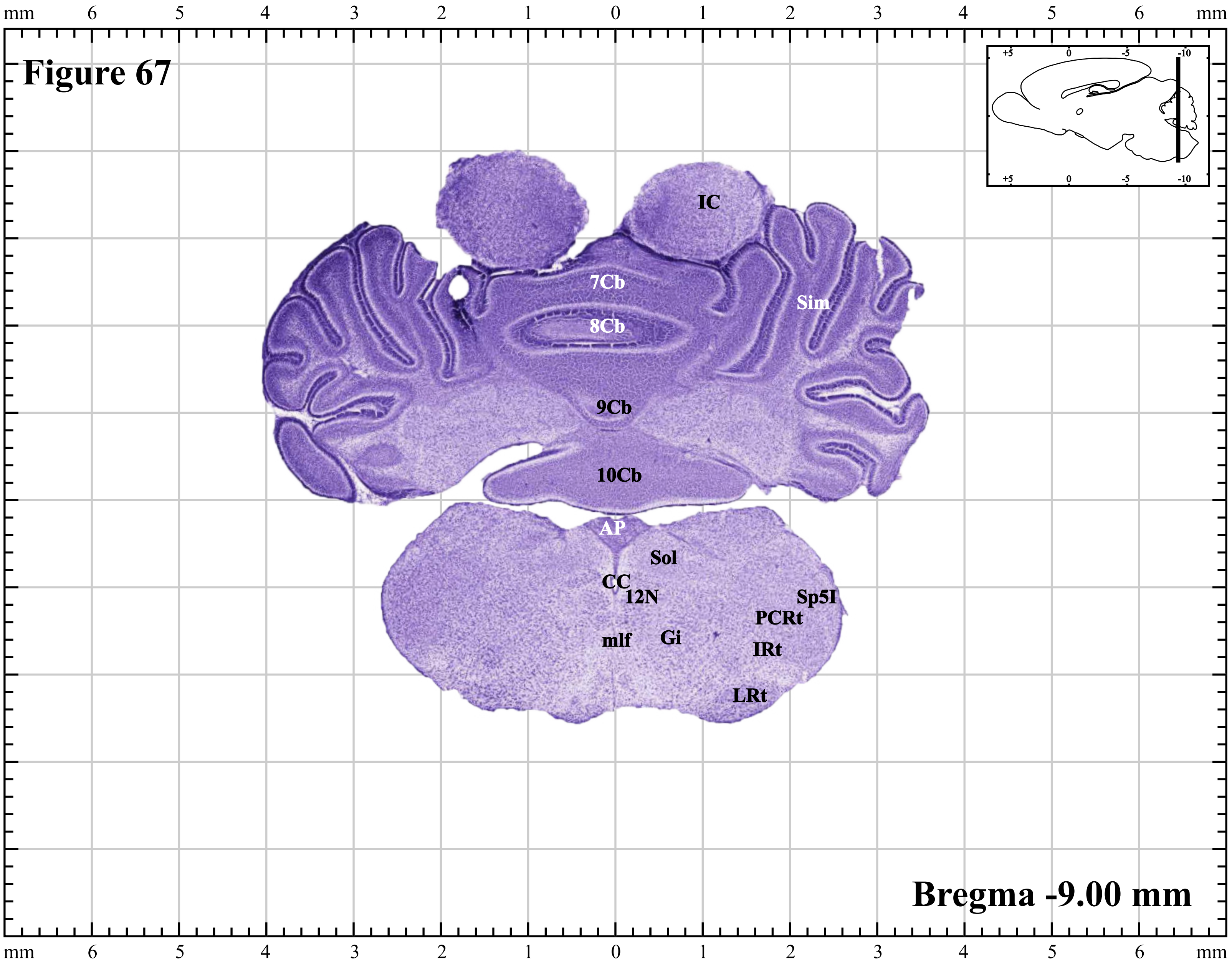

7Cb 7th cerebellar lobule
8Cb 8th cerebellar lobule
9Cb 9th cerebellar lobules
10Cb 10th cerebellar lobule
12N hypoglossal nucleus
AP area postrema
CC central canal
IC inferior colliculus
Gi granular insular cortex
IRt intermediate reticular nucleus
LRt lateral reticular nucleus
mlf medial longitudinal fasciculus
PCRt parvicellular reticular nucleus
Sol nucleus of the solitary tract
Sim simple lobule
Sp5I spinal trigeminal nucleus, interpolar part

Figure 68

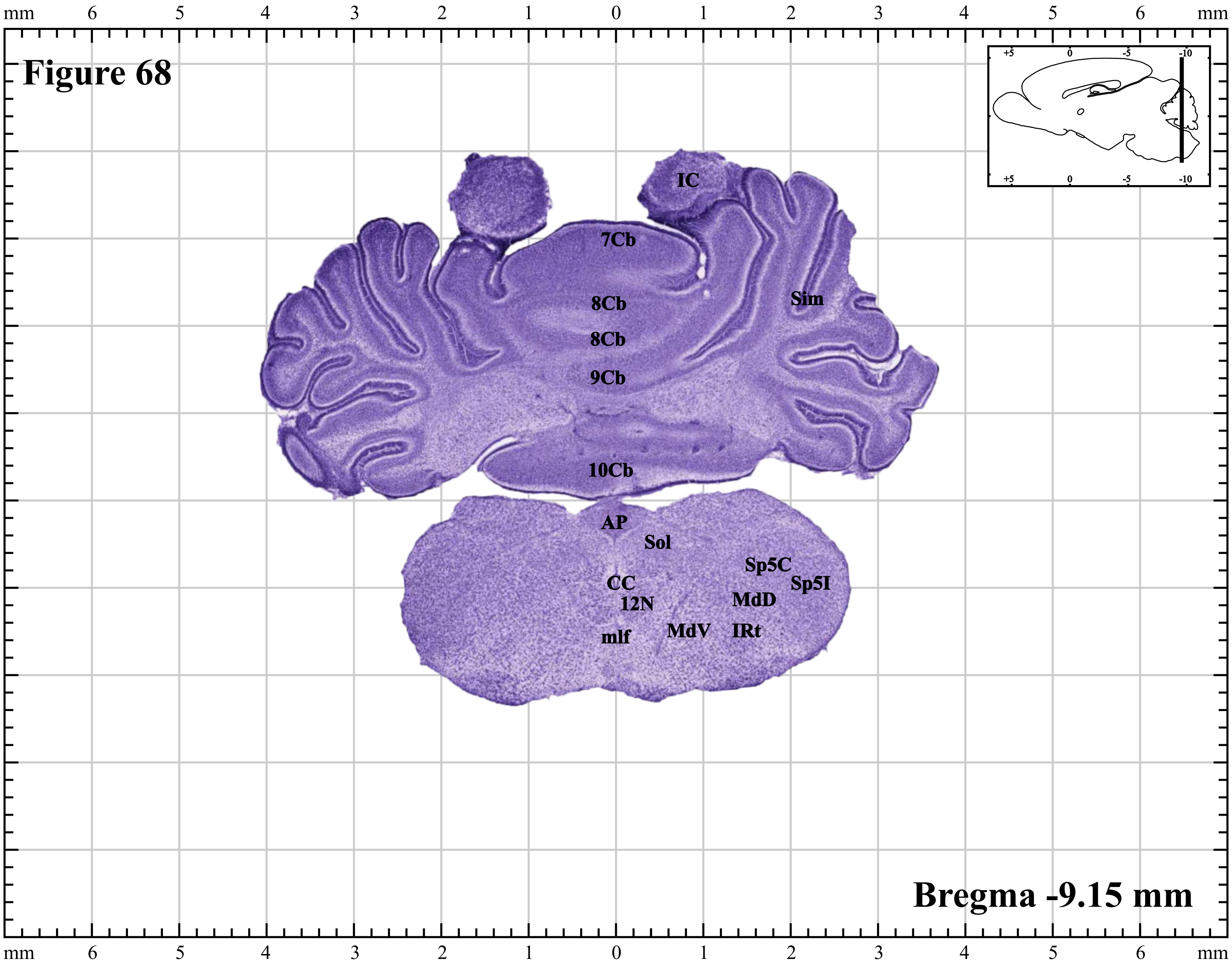

Bregma -9.15 mm

7Cb 7th cerebellar lobule
8Cb 8th cerebellar lobule
9Cb 9th cerebellar lobules
10Cb 10th cerebellar lobule
12N hypoglossal nucleus
AP area postrema
CC central canal
IC inferior colliculus
Gi granular insular cortex
IRt intermediate reticular nucleus
mlf medial longitudinal fasciculus
MdD medullary reticular nucleus, dorsal part
MdV medullary reticular nucleus, ventral part
Sol nucleus of the solitary tract
Sim simple lobule
Sp5I spinal trigeminal nucleus, interpolar part
Sp5C spinal trigeminal nucleus, caudal part

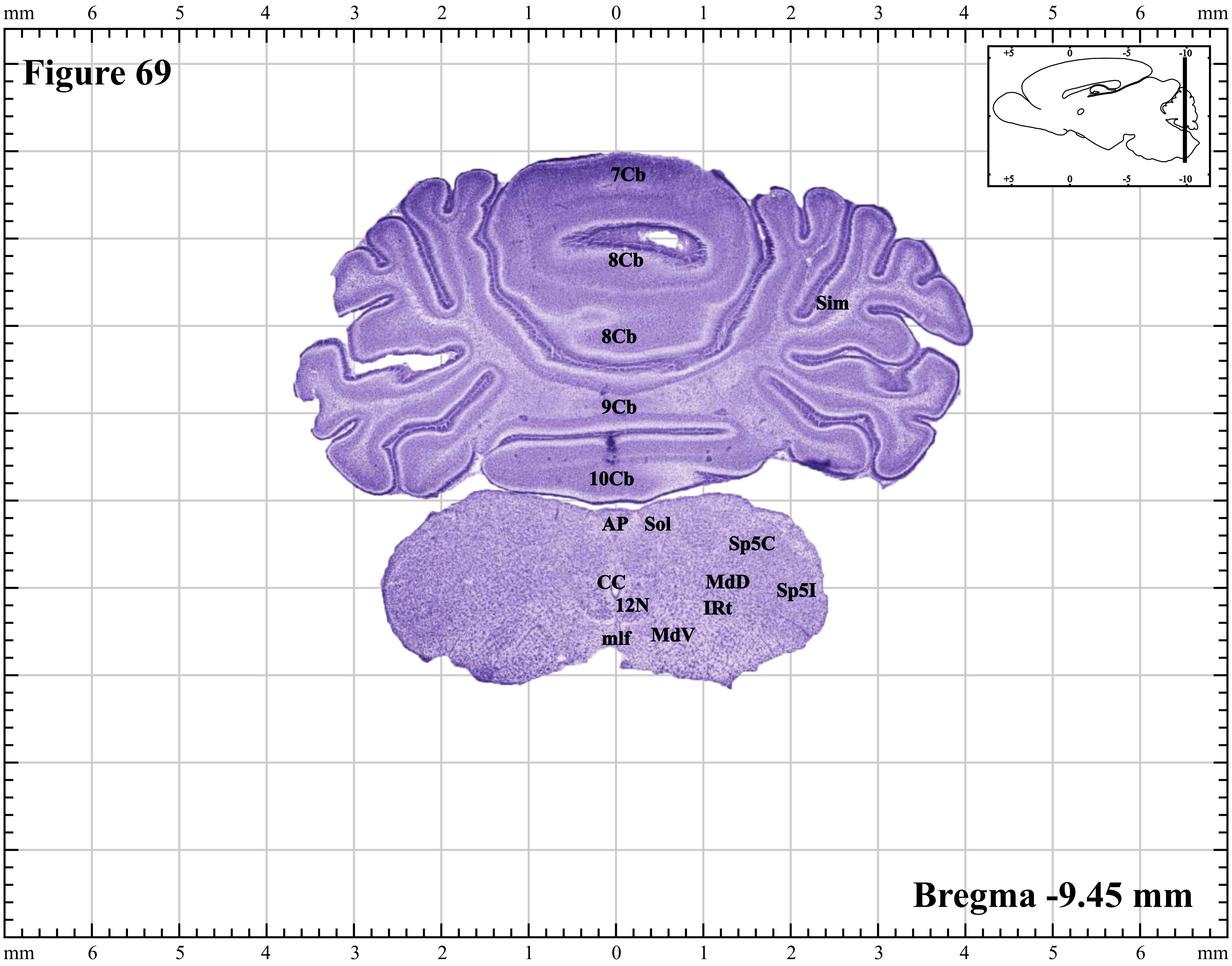

7Cb 7th cerebellar lobule
8Cb 8th cerebellar lobule
9Cb 9th cerebellar lobules
10Cb 10th cerebellar lobule
12N hypoglossal nucleus
AP area postrema
CC central canal
Gi granular insular cortex
IRt intermediate reticular nucleus
mlf medial longitudinal fasciculus
MdD medullary reticular nucleus, dorsal part
MdV medullary reticular nucleus, ventral part
Sol nucleus of the solitary tract
Sim simple lobule
Sp5I spinal trigeminal nucleus, interpolar part
Sp5C spinal trigeminal nucleus, caudal part

Figure 70

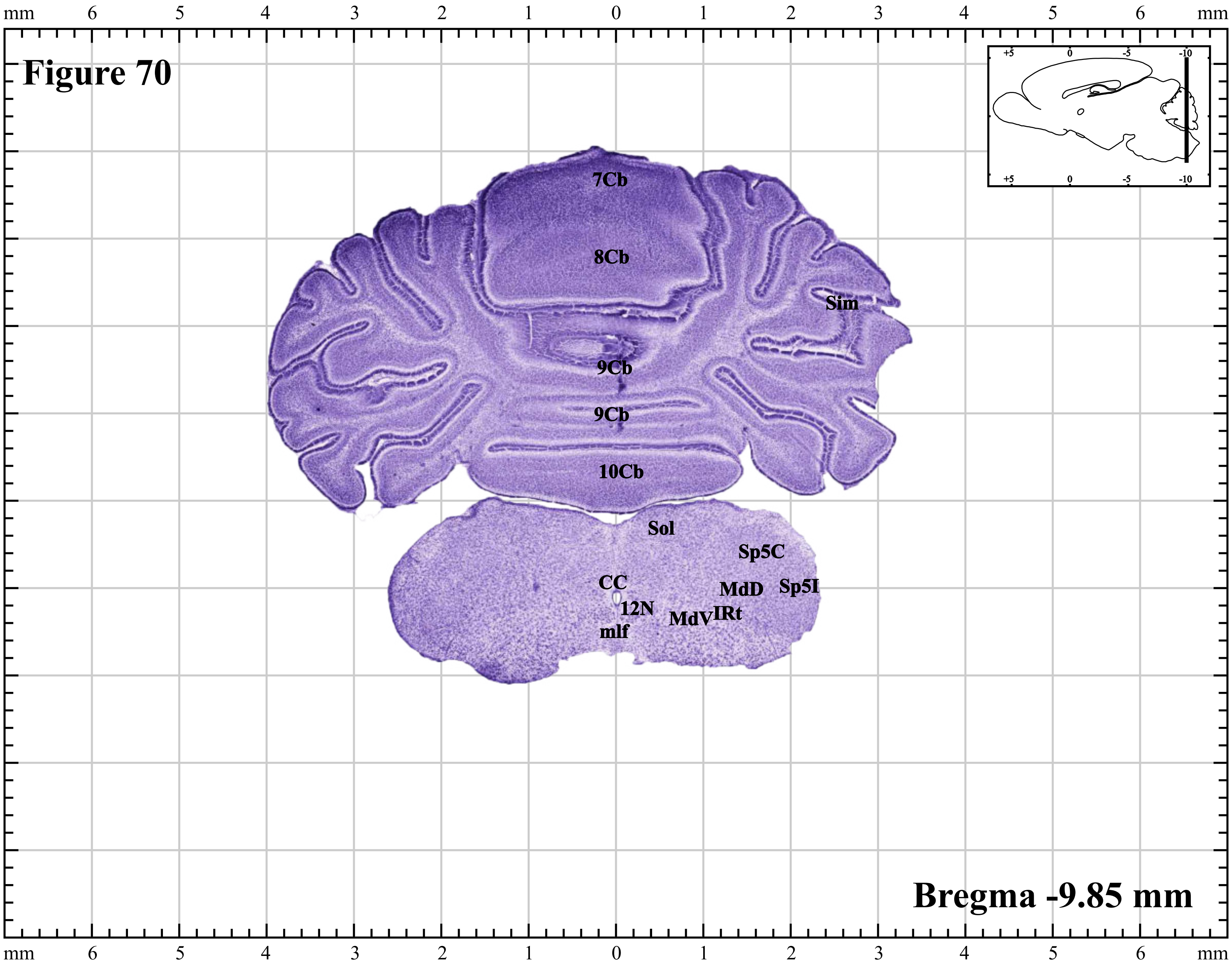

Bregma -9.85 mm

7Cb 7th cerebellar lobule
8Cb 8th cerebellar lobule
9Cb 9th cerebellar lobules
10Cb 10th cerebellar lobule
12N hypoglossal nucleus
CC central canal
Gi granular insular cortex
IRt intermediate reticular nucleus
mlf medial longitudinal fasciculus
MdD medullary reticular nucleus, dorsal part
MdV medullary reticular nucleus, ventral part
Sol nucleus of the solitary tract
Sim simple lobule
Sp5I spinal trigeminal nucleus, interpolar part
Sp5C spinal trigeminal nucleus, caudal part
