# Supplementary material for: Stereotaxic atlas of the infant rat brain at postnatal days 7–13
Source: Front Neuroanat. 2022 Aug 12;16:968320. doi: 10.3389/fnana.2022.968320 (PMC9412974; doi:10.3389/fnana.2022.968320)
Supplement: Supplementary file 6 [file Data_Sheet_6.PDF]

Supplementary Material 6

Stereotaxic Atlas of the Infant Rat Brain

P12 (# G-12-2, 30.0 g)

Yu-Nong Chen[1], Xin Zheng[1], Hai-Lin Chen[1], Jin-Xian Gao[1], Xin-Xuan Li[1], Jun-Fan Xie[1], Yu-Ping Xie[3], Karen Spruyt[4], Yu-Feng Shao[1,2]* and Yi-Ping Hou[1,2]*

[1]Departments of Neuroscience, Anatomy, Histology, and Embryology, Key Laboratory of Preclinical Study for New Drugs of Gansu Province, School of Basic Medical Sciences, Lanzhou University, Lanzhou, China
[2]Key Lab of Neurology of Gansu Province, Lanzhou University, Lanzhou, China
[3]Sleep Medicine Center of Gansu Provincial Hospital, Lanzhou, China
[4]Université de Paris, NeuroDiderot – INSERM, Paris, France.

* Correspondence: Yu-Feng Shao (shaoyf@lzu.edu.cn); Yi-Ping Hou (houyiping@lzu.edu.cn)

Figure 01

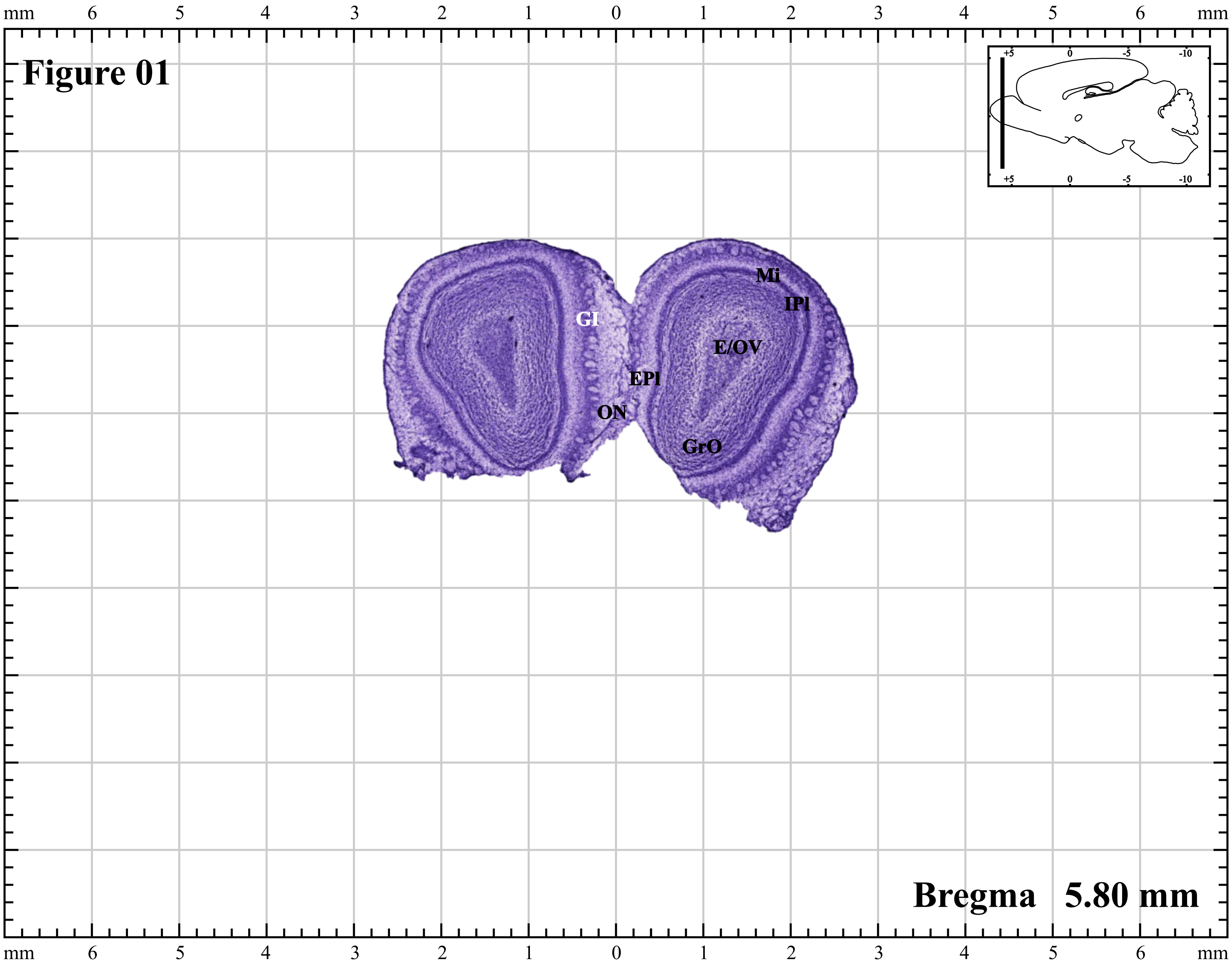

Bregma 5.80 mm

E/OV ependymal and subendymal layer/olfactory ventricle
EPl external plexiform layer of the olfactory bulb
GrO granular cell layer of the olfactory bulb
GI granular insular cortex
IPl internal plexiform layer of the olfactory bulb
Mi mitral cell layer of the olfactory bulb
ON olfactory nerve layer

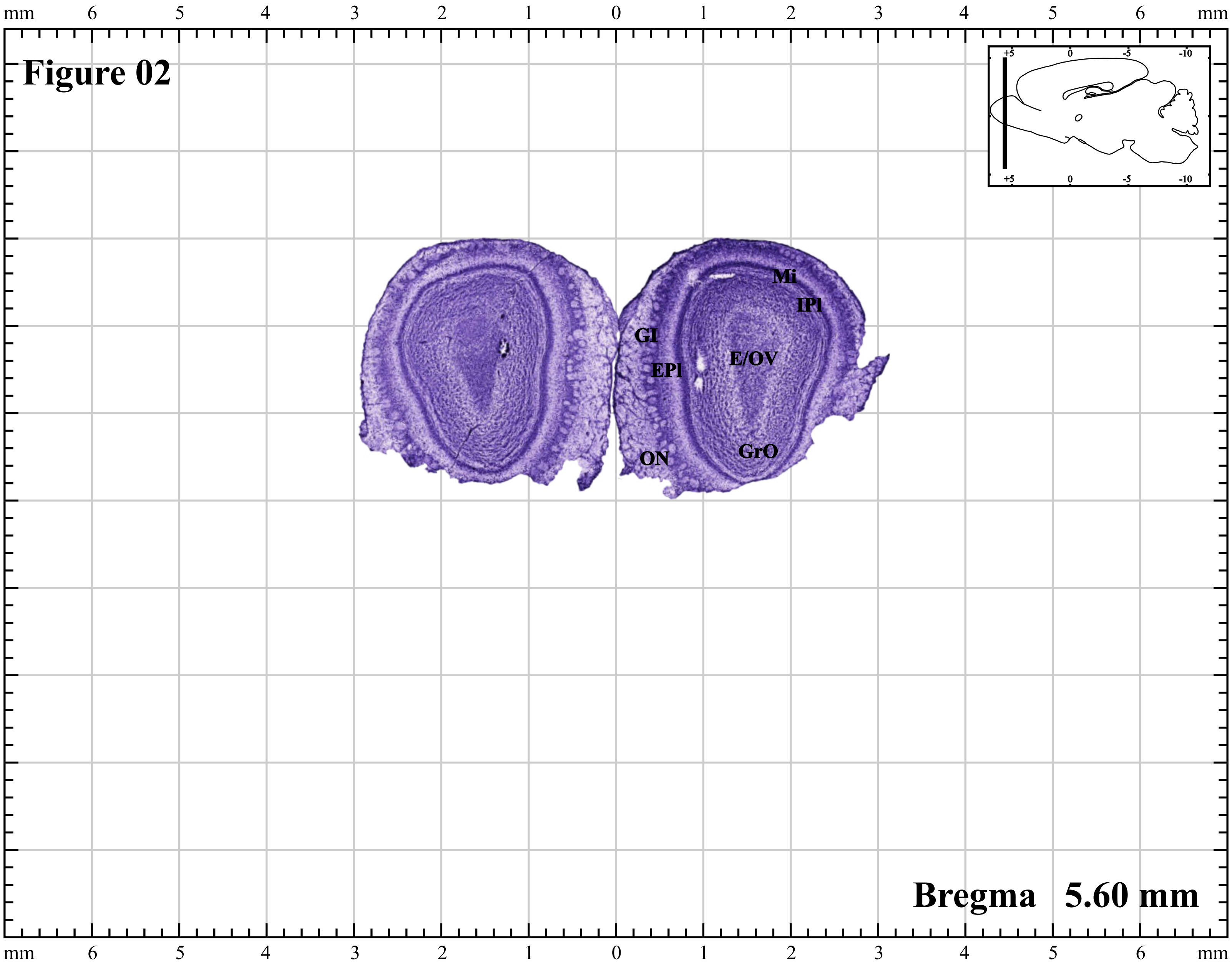

E/OV ependymal and subendymal layer/olfactory ventricle
EPl external plexiform layer of the olfactory bulb
GrO granular cell layer of the olfactory bulb
GI granular insular cortex
IPl internal plexiform layer of the olfactory bulb
Mi mitral cell layer of the olfactory bulb
ON olfactory nerve layer

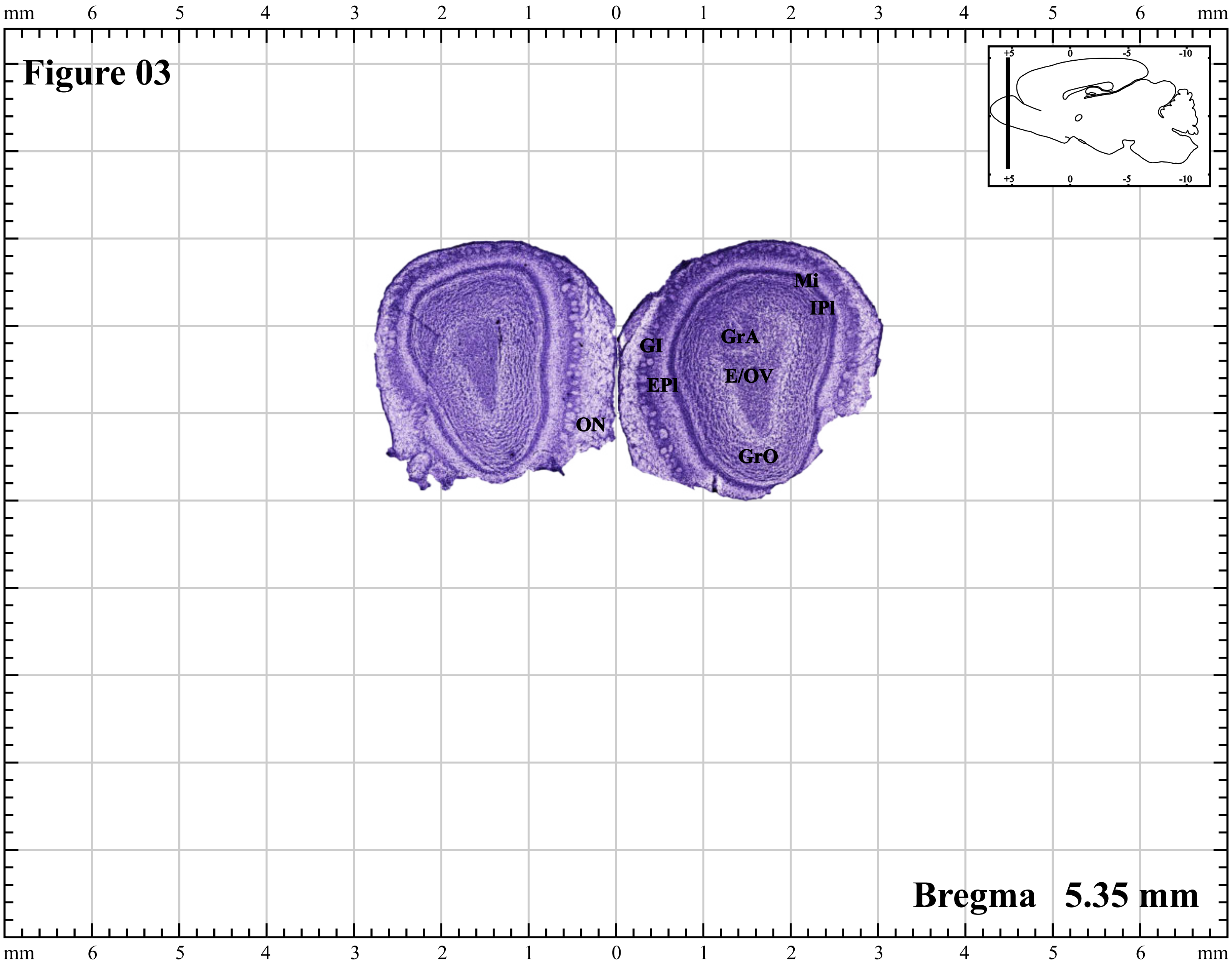

E/OV ependymal and subendymal layer/olfactory ventricle
EPl external plexiform layer of the olfactory bulb
GrO granular cell layer of the olfactory bulb
GI granular insular cortex
GrA granule cell layer of the accessory olfactory bulb
IPl internal plexiform layer of the olfactory bulb
Mi mitral cell layer of the olfactory bulb
ON olfactory nerve layer

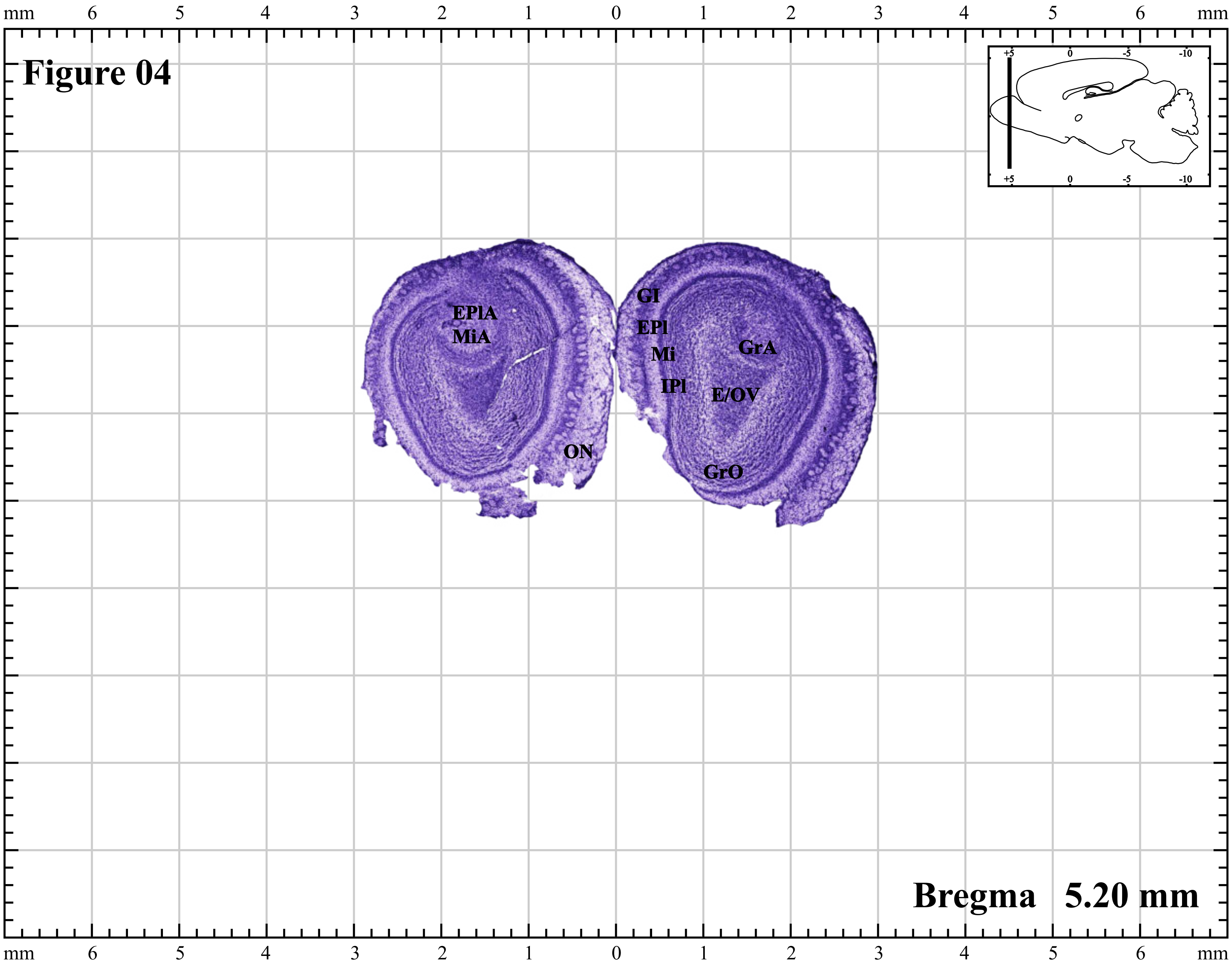

EPl external plexiform layer of the olfactory bulb

E/OV ependymal and subendymal layer/olfactory ventricle

EPlA external plexiform layer of the accessory olfactory bulb

GrO granular cell layer of the olfactory bulb

GI granular insular cortex

GrA granule cell layer of the accessory olfactory bulb

IPl internal plexiform layer of the olfactory bulb

Mi mitral cell layer of the olfactory bulb

MiA mitral cell layer of the accessory olfactory bulb

ON olfactory nerve layer

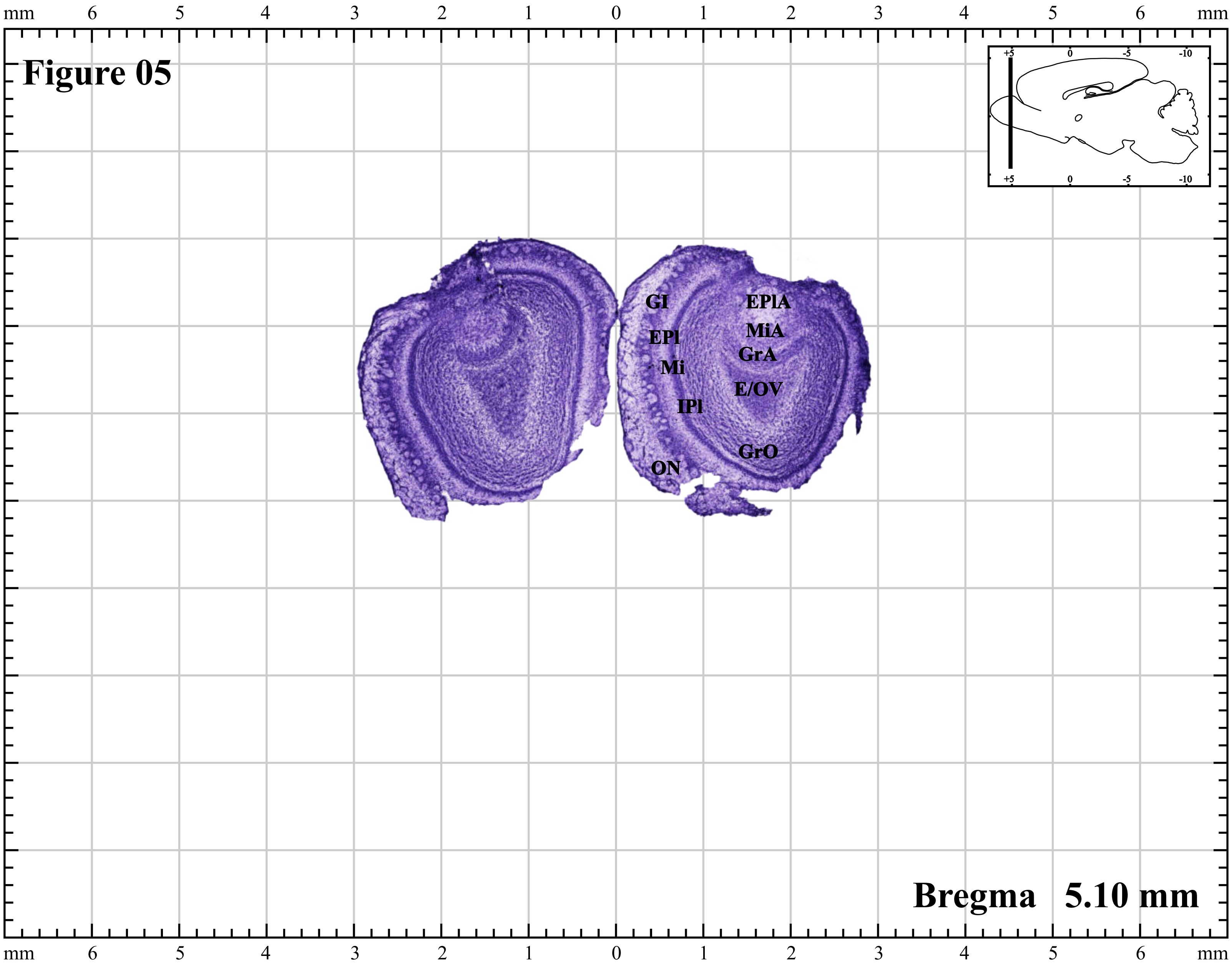

AOL anterior olfactory nucleus, lateral part
EPl external plexiform layer of the olfactory bulb
E/OV ependymal and subendymal layer/olfactory ventricle
EPlA external plexiform layer of the accessory olfactory bulb
GI granular insular cortex
GrO granular cell layer of the olfactory bulb
GrA granule cell layer of the accessory olfactory bulb
IPl internal plexiform layer of the olfactory bulb
Mi mitral cell layer of the olfactory bulb
MiA mitral cell layer of the accessory olfactory bulb
ON olfactory nerve layer

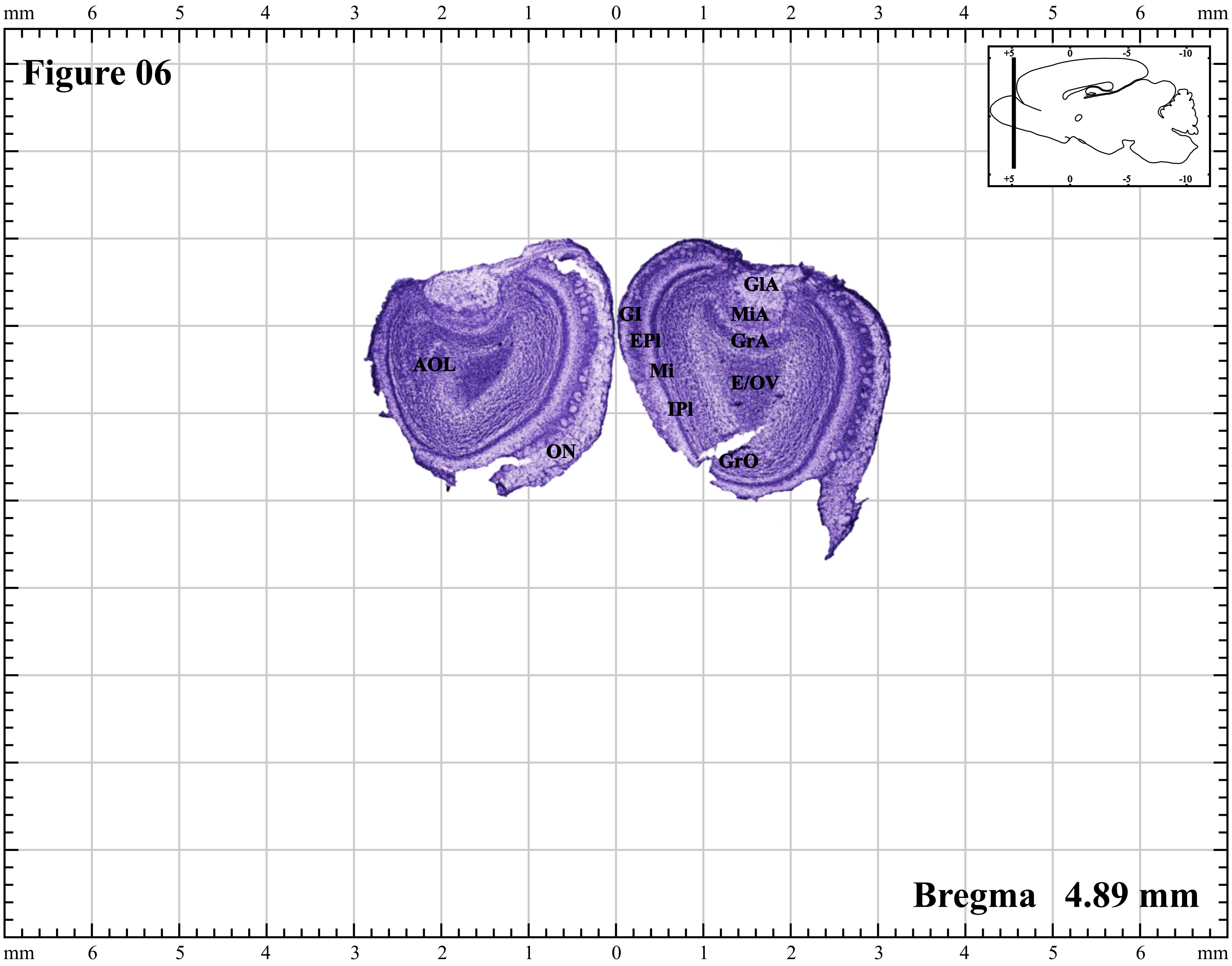

AOL anterior olfactory nucleus, lateral part
EPl external plexiform layer of the olfactory bulb
E/OV ependymal and subendymal layer/olfactory ventricle
GlA glomerular layer of the accessory olfactory bulb
GrO granular cell layer of the olfactory bulb
GI granular insular cortex
GrA granule cell layer of the accessory olfactory bulb
IPl internal plexiform layer of the olfactory bulb
MiA mitral cell layer of the accessory olfactory bulb
Mi mitral cell layer of the olfactory bulb
ON olfactory nerve layer

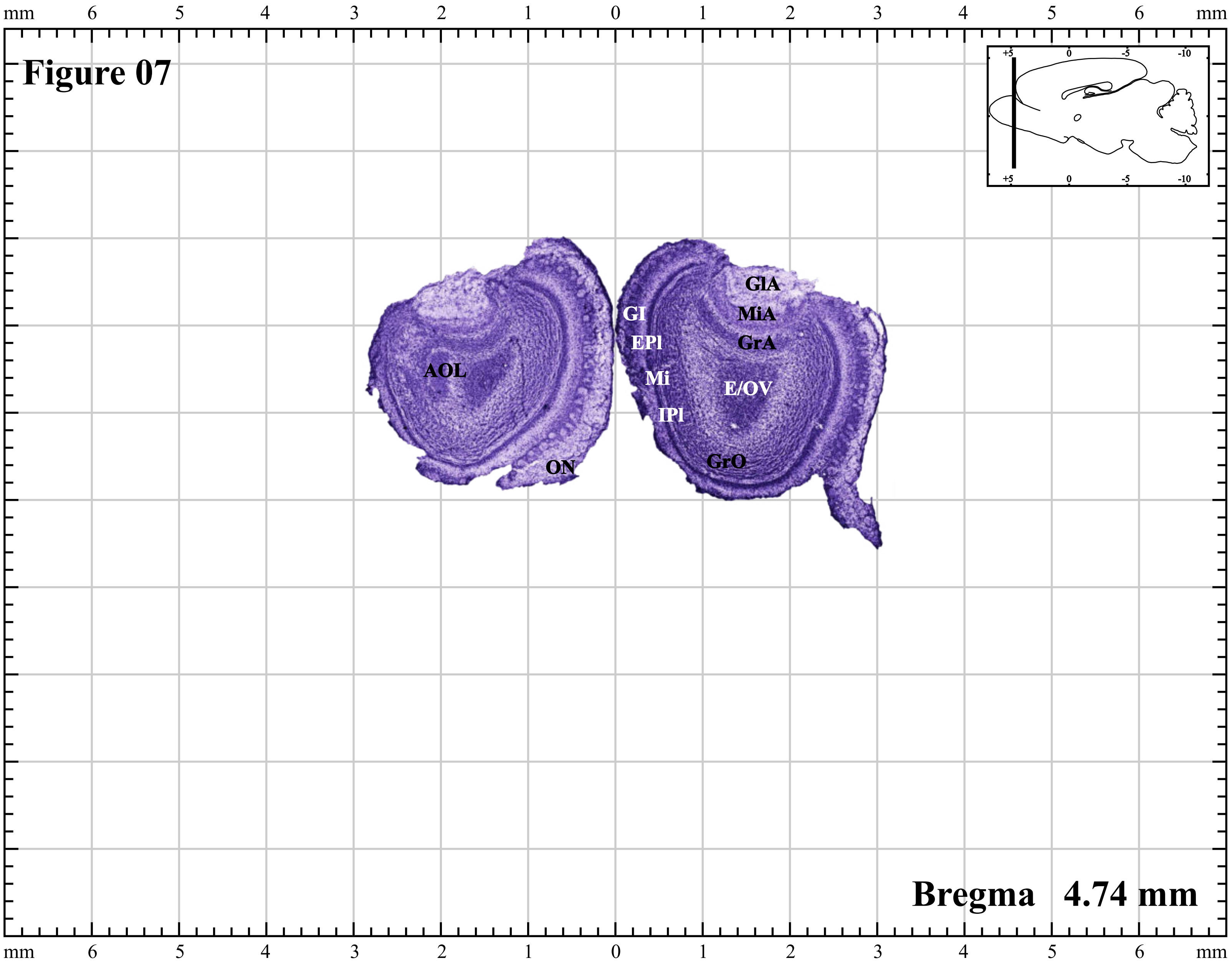

AOL anterior olfactory nucleus, lateral part

EPl external plexiform layer of the olfactory bulb

E/OV ependymal and subendymal layer/olfactory ventricle

GlA glomerular layer of the accessory olfactory bulb

GI granular insular cortex

GrO granular cell layer of the olfactory bulb

GrA granule cell layer of the accessory olfactory bulb

IPl internal plexiform layer of the olfactory bulb

Mi mitral cell layer of the olfactory bulb

MiA mitral cell layer of the accessory olfactory bulb

ON olfactory nerve layer

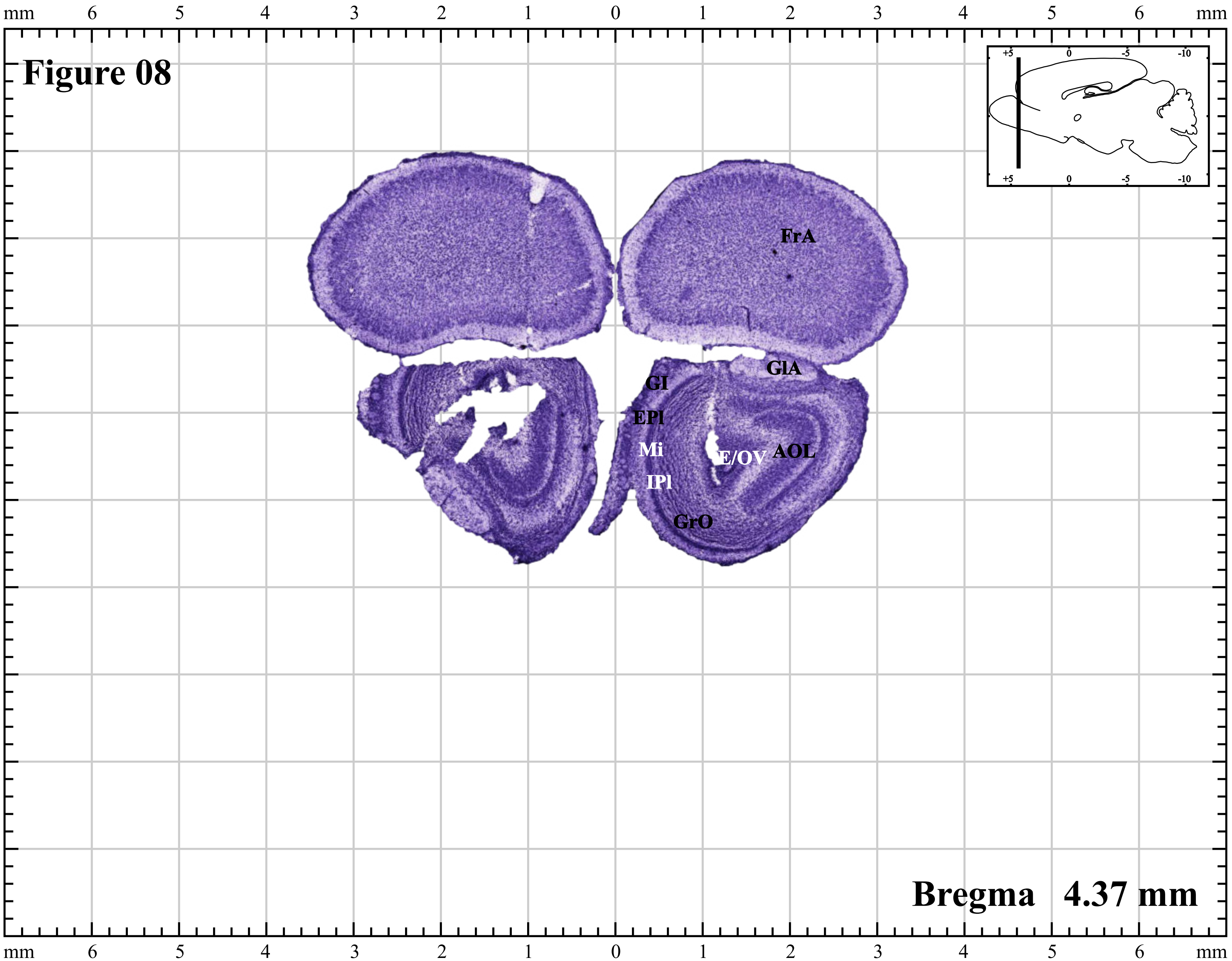

AOL anterior olfactory nucleus, lateral part
EPl external plexiform layer of the olfactory bulb
E/OV ependymal and subendymal layer/olfactory ventricle
FrA frontal assocn cortex
GlA glomerular layer of the accessory olfactory bulb
GrO granular cell layer of the olfactory bulb
GI granular insular cortex
IPl internal plexiform layer of the olfactory bulb
Mi mitral cell layer of the olfactory bulb

Figure 09

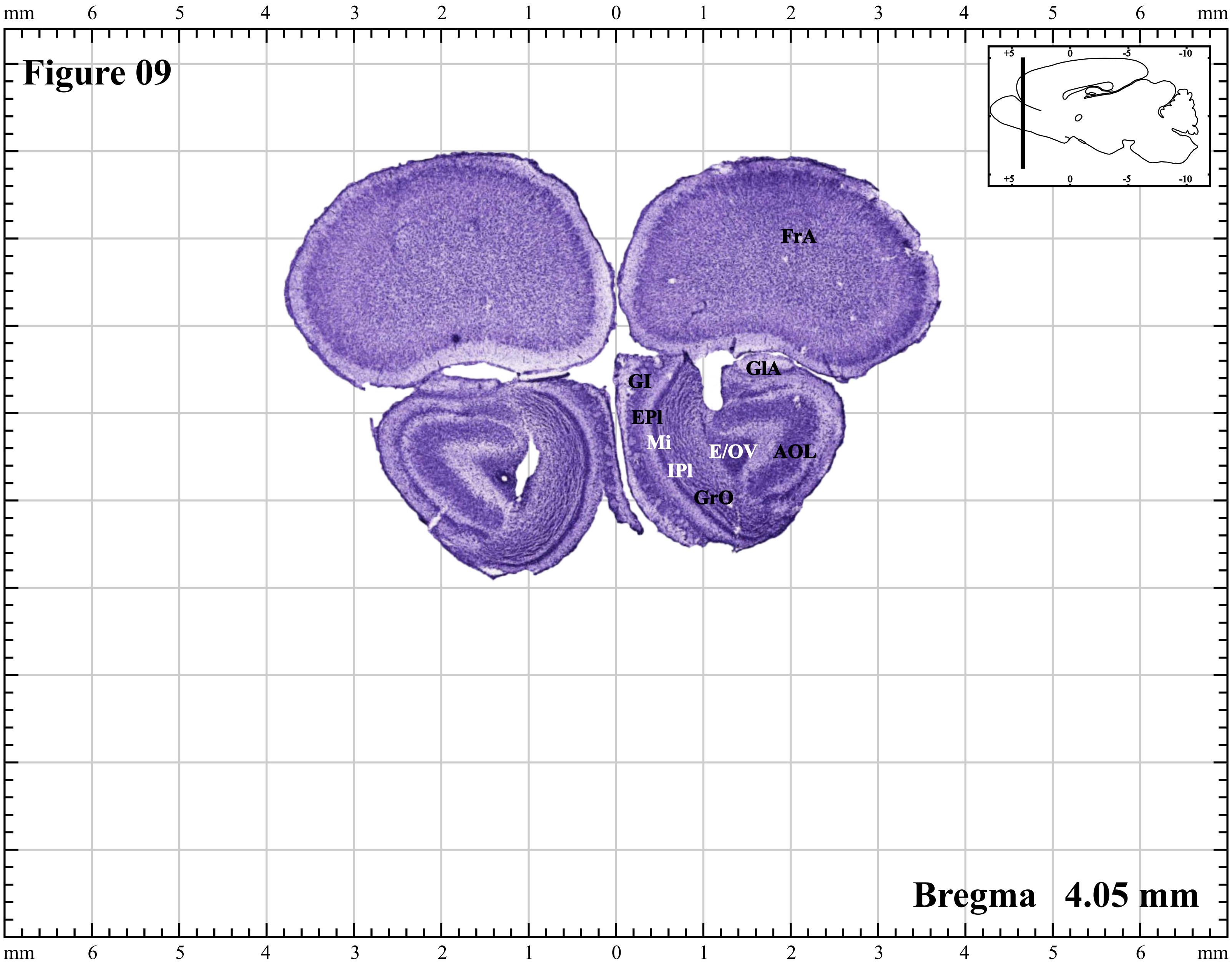

Bregma 4.05 mm

AOL anterior olfactory nucleus, lateral part
EPl external plexiform layer of the olfactory bulb
E/OV ependymal and subendymal layer/olfactory ventricle
FrA frontal assocn cortex
GlA glomerular layer of the accessory olfactory bulb
GrO granular cell layer of the olfactory bulb
GI granular insular cortex
IPl internal plexiform layer of the olfactory bulb
Mi mitral cell layer of the olfactory bulb

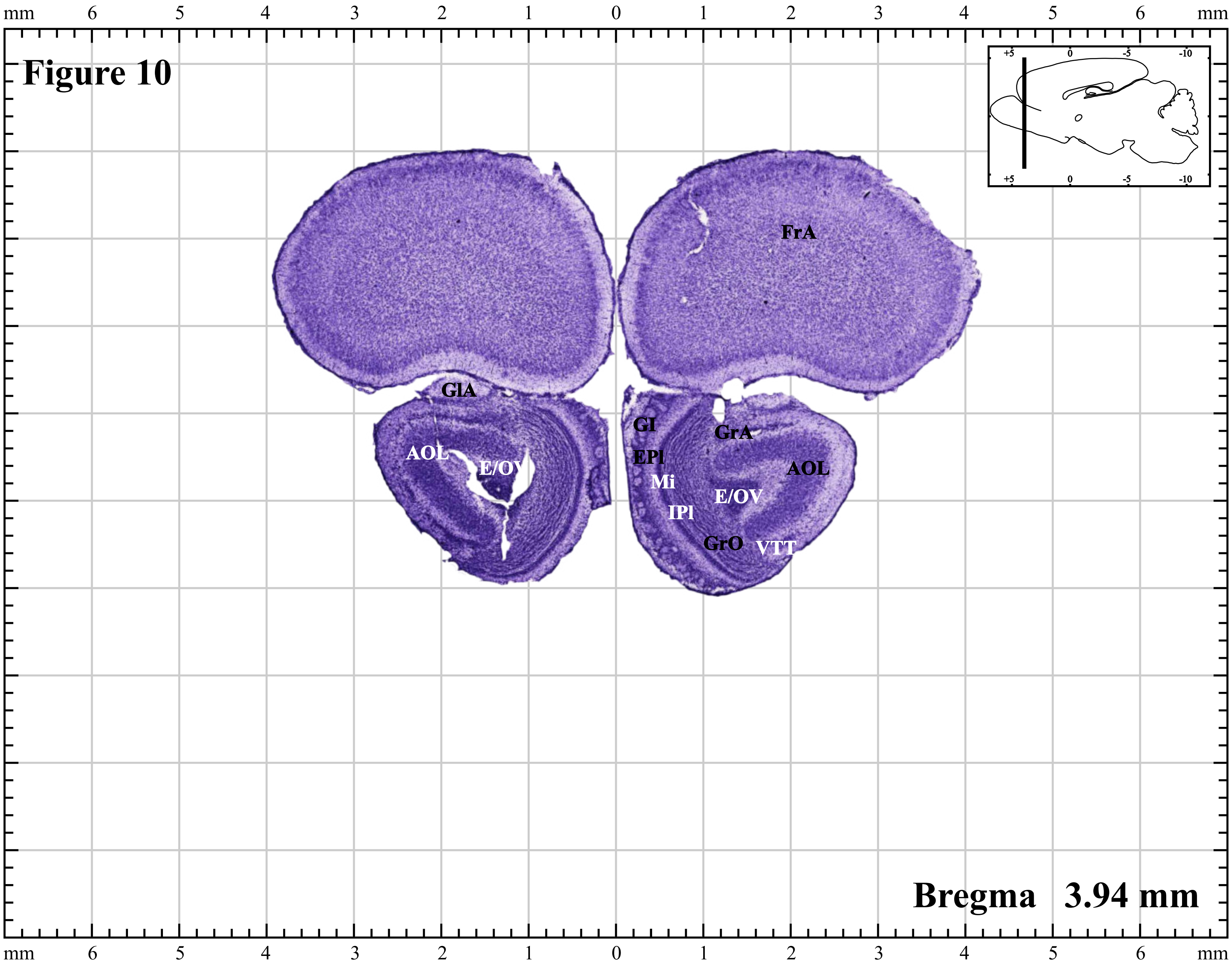

AOL anterior olfactory nucleus, lateral part

EPl external plexiform layer of the olfactory bulb

E/OV ependymal and subendymal layer/olfactory ventricle

FrA frontal assocn cortex

GrA granule cell layer of the accessory olfactory bulb

GlA glomerular layer of the accessory olfactory bulb

GrO granular cell layer of the olfactory bulb

GI granular insular cortex

IPl internal plexiform layer of the olfactory bulb

Mi mitral cell layer of the olfactory bulb

VTT ventral tenia tecta

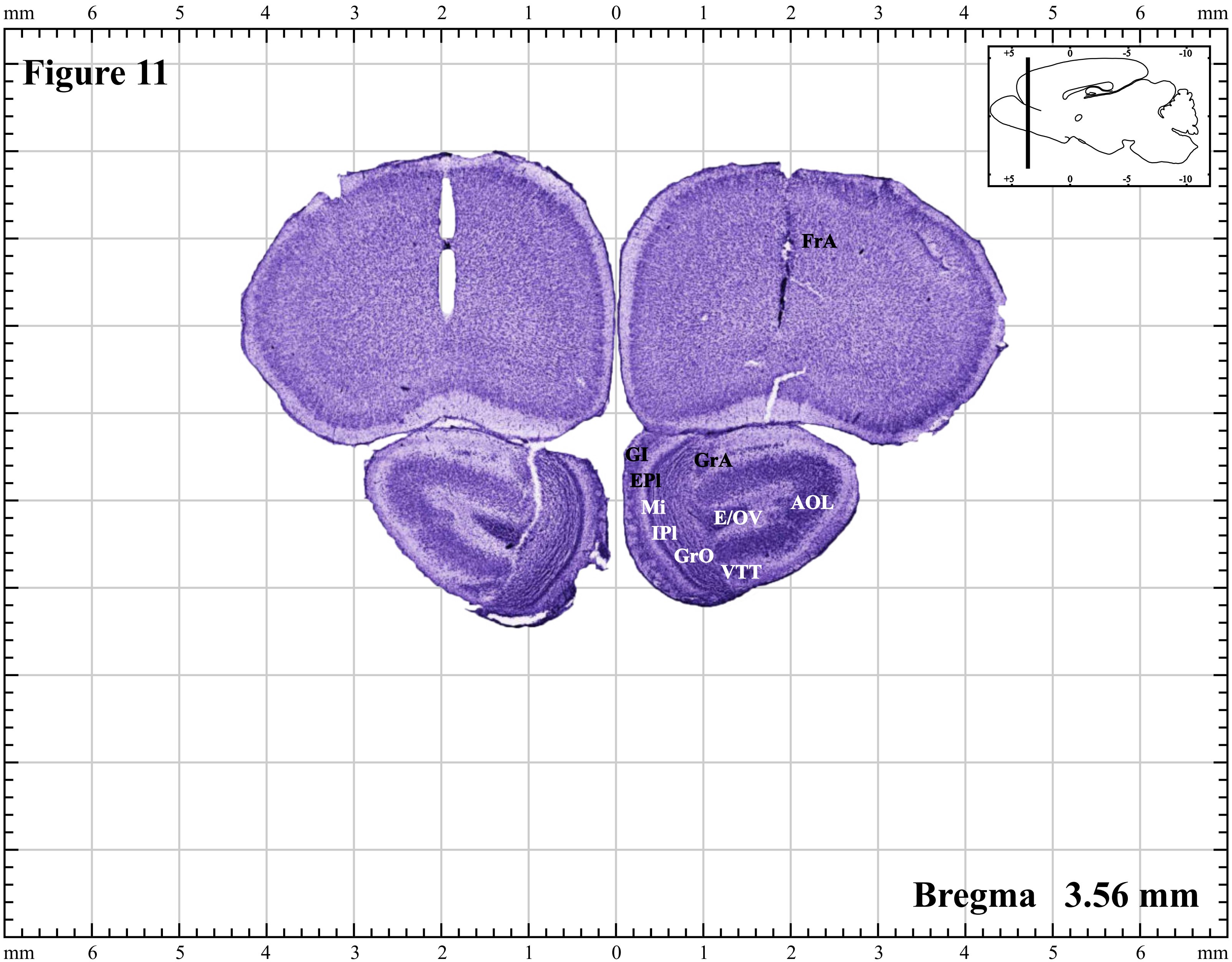

AOL anterior olfactory nucleus, lateral part
EPl external plexiform layer of the olfactory bulb
E/OV ependymal and subendymal layer/olfactory ventricle
FrA frontal assocn cortex
GrA granule cell layer of the accessory olfactory bulb
GrO granular cell layer of the olfactory bulb
GI granular insular cortex
IPl internal plexiform layer of the olfactory bulb
Mi mitral cell layer of the olfactory bulb
VTT ventral tenia tecta

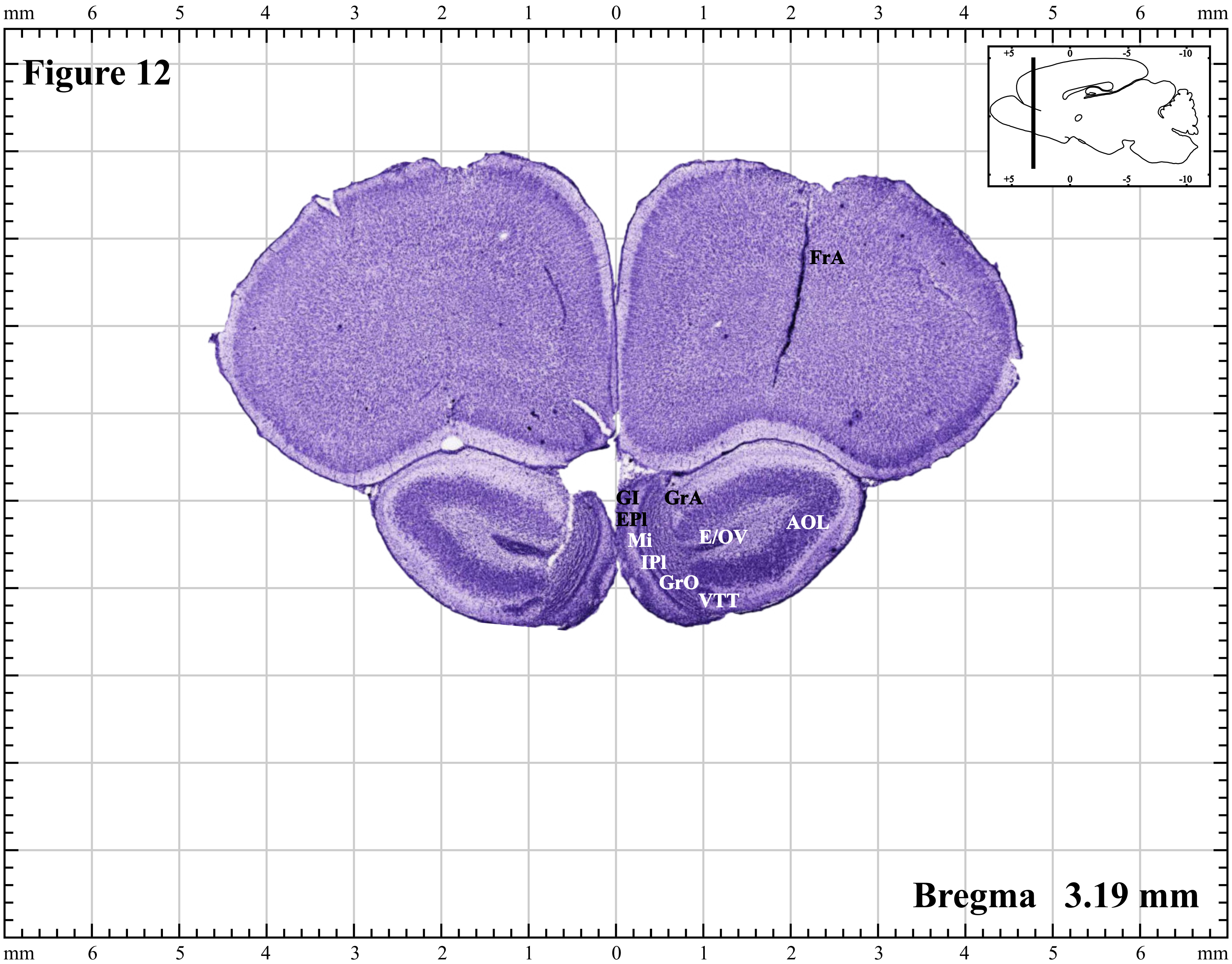

AOL anterior olfactory nucleus, lateral part

EPl external plexiform layer of the olfactory bulb

E/OV ependymal and subendymal layer/olfactory ventricle

FrA frontal assocn cortex

GrA granule cell layer of the accessory olfactory bulb

GrO granular cell layer of the olfactory bulb

GI granular insular cortex

MiA mitral cell layer of the accessory olfactory bulb

ON olfactory nerve layer

VTT ventral tenia tecta

IPl internal plexiform layer of the olfactory bulb

Mi mitral cell layer of the olfactory bulb

Figure 13

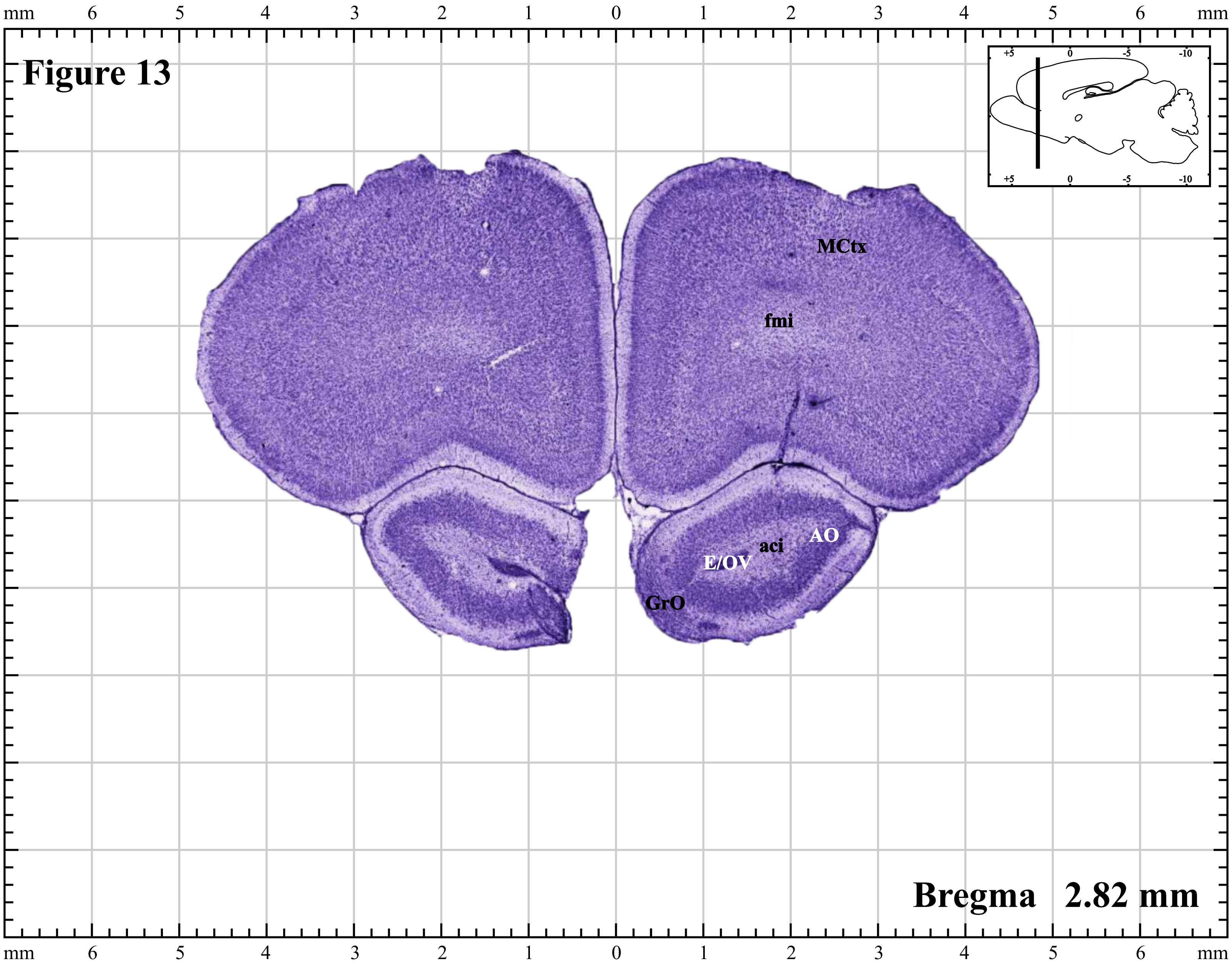

Bregma 2.82 mm

aci anterior commissure, intrabulbar part
AO anterior olfactory nuclues
E/OV ependymal and subendymal layer/ olfactory ventricle
GrO granular cell layer of the olfactory bulb
fmi forceps major of corpus callosum
MCtx motor cortex

Figure 14

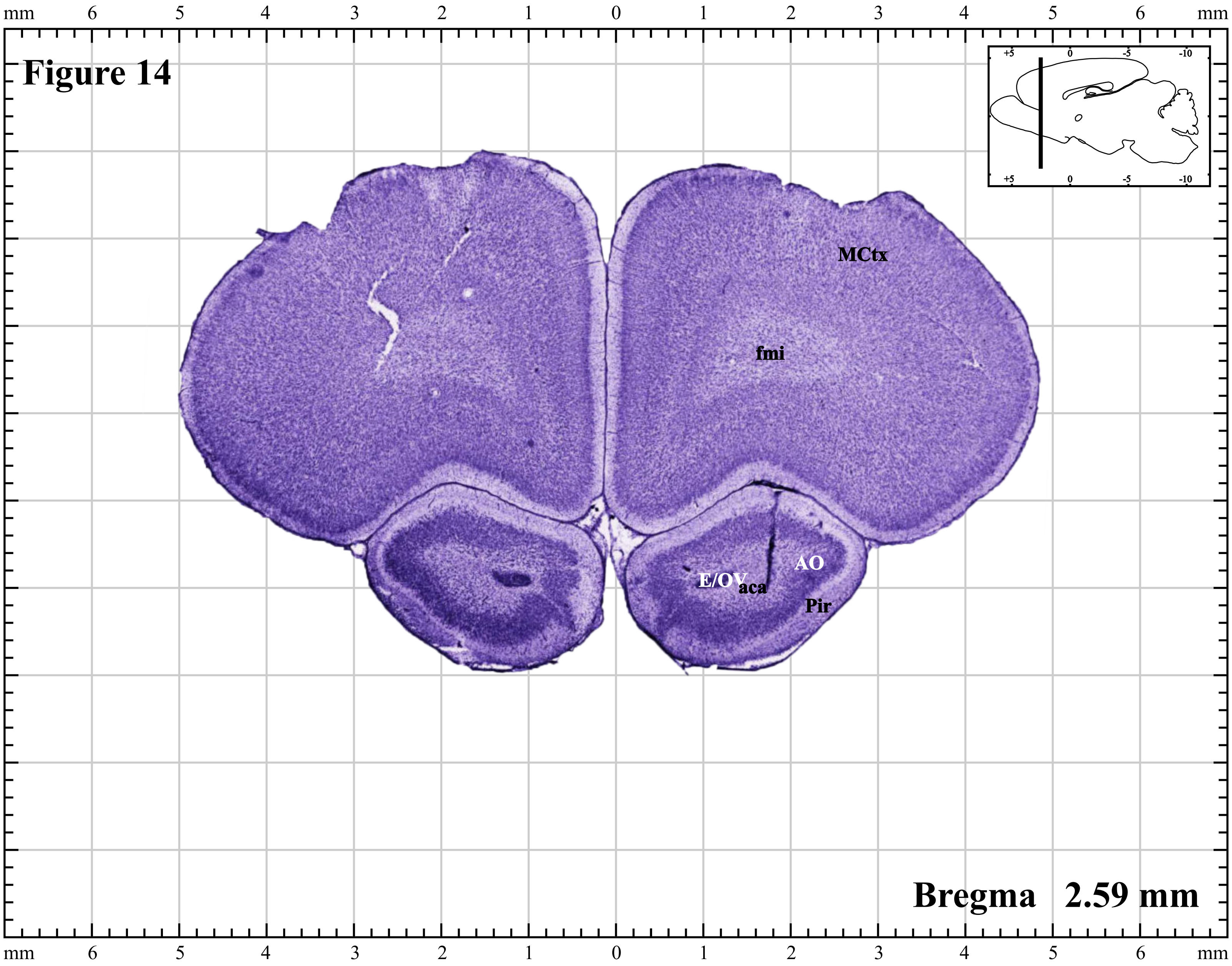

Bregma 2.59 mm

aca anterior commissure, anterior part
AO anterior olfactory nuclues
E/OV ependyma and subependymal layer /olfactory ventricle
fmi forceps major of corpus callosum
MCtx motor cortex
Pir piriform cortex

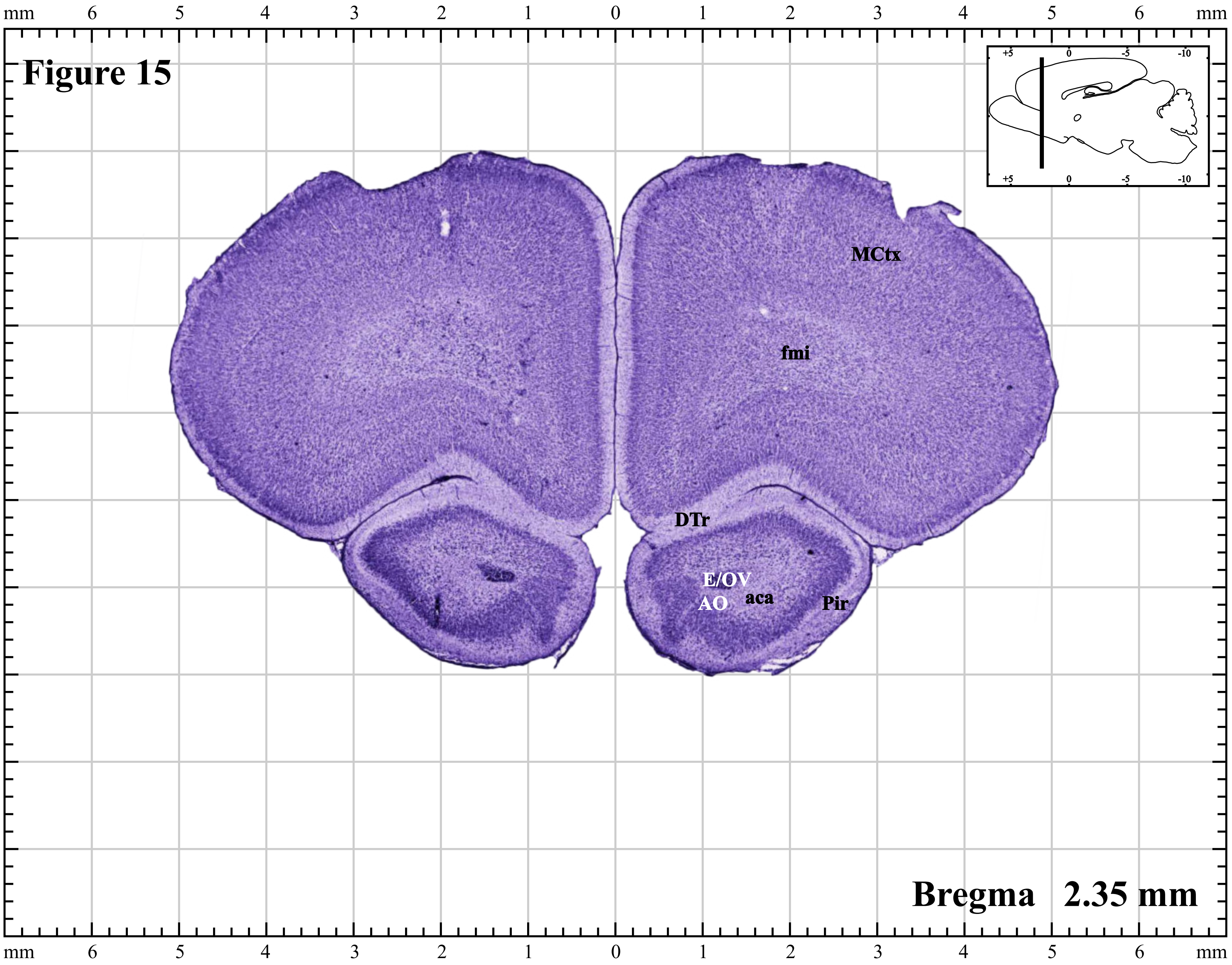

aca anterior commissure, anterior part
AO anterior olfactory nuclues
DTr dorsal transition zone
E/OV ependyma and subependymal layer /olfactory ventricle
fmi forceps major of corpus callosum
MCtx motor cortex
Pir piriform cortex

Figure 16

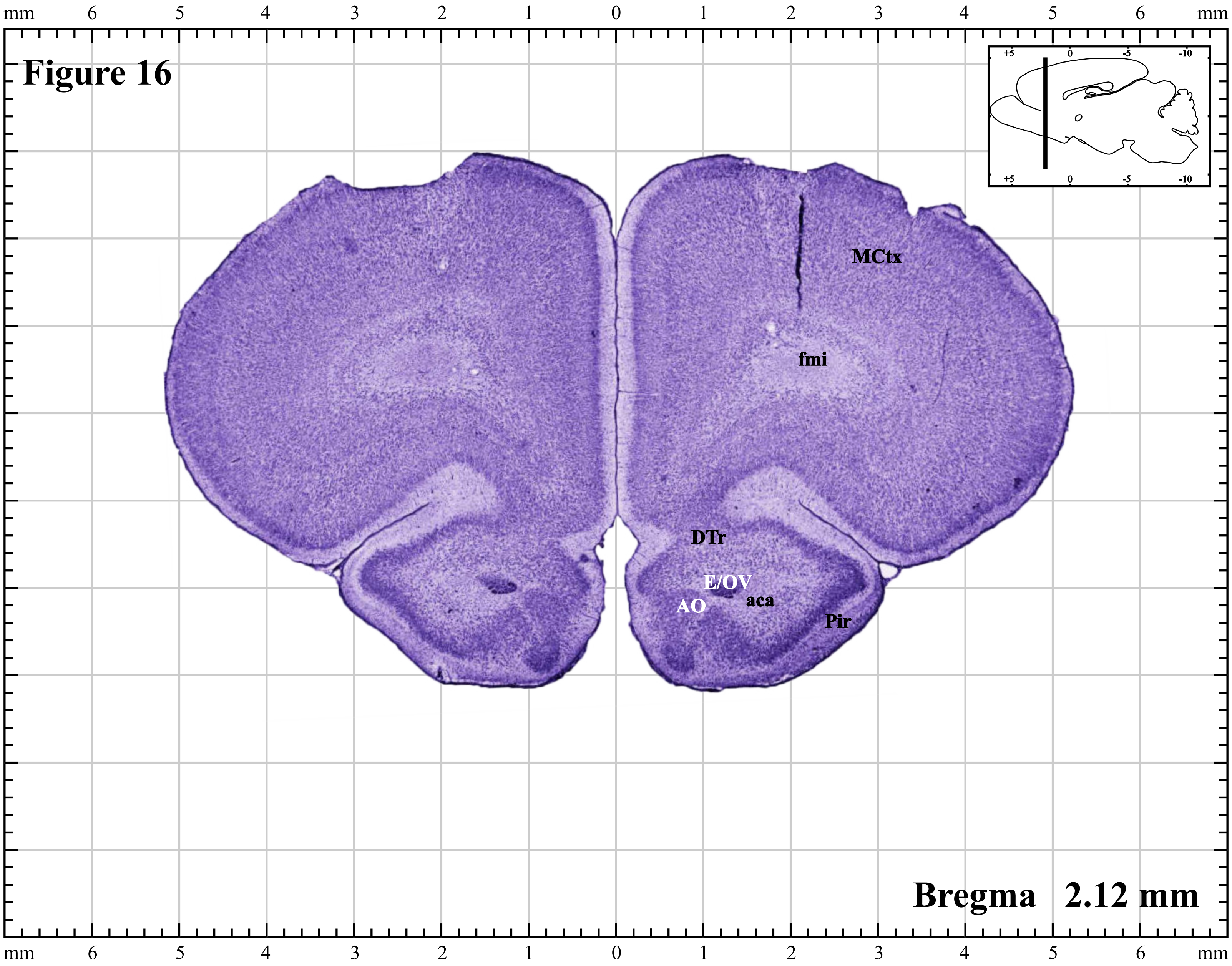

Bregma 2.12 mm

aca anterior commissure, anterior part
AO anterior olfactory nuclues
DTr dorsal transition zone
E/OV ependyma and subependymal layer
/olfactory ventricle
fmi forceps major of corpus callosum
MCtx motor cortex
Pir piriform cortex

Figure 17

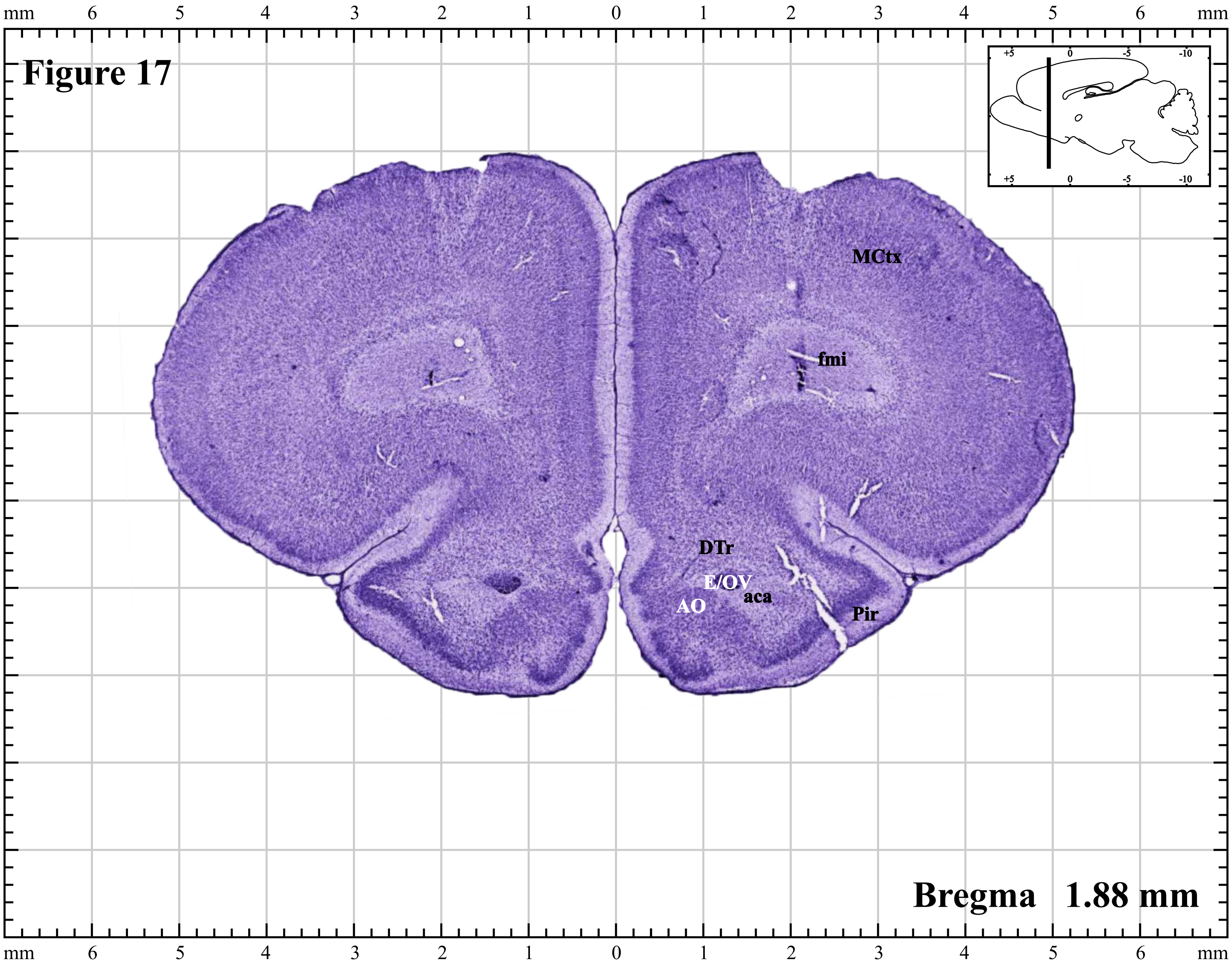

Bregma 1.88 mm

aca anterior commissure, anterior part
AO anterior olfactory nuclues
DTr dorsal transition zone
E/OV ependyma and subependymal layer
/olfactory ventricle
fmi forceps major of corpus callosum
MCtx motor cortex
Pir piriform cortex

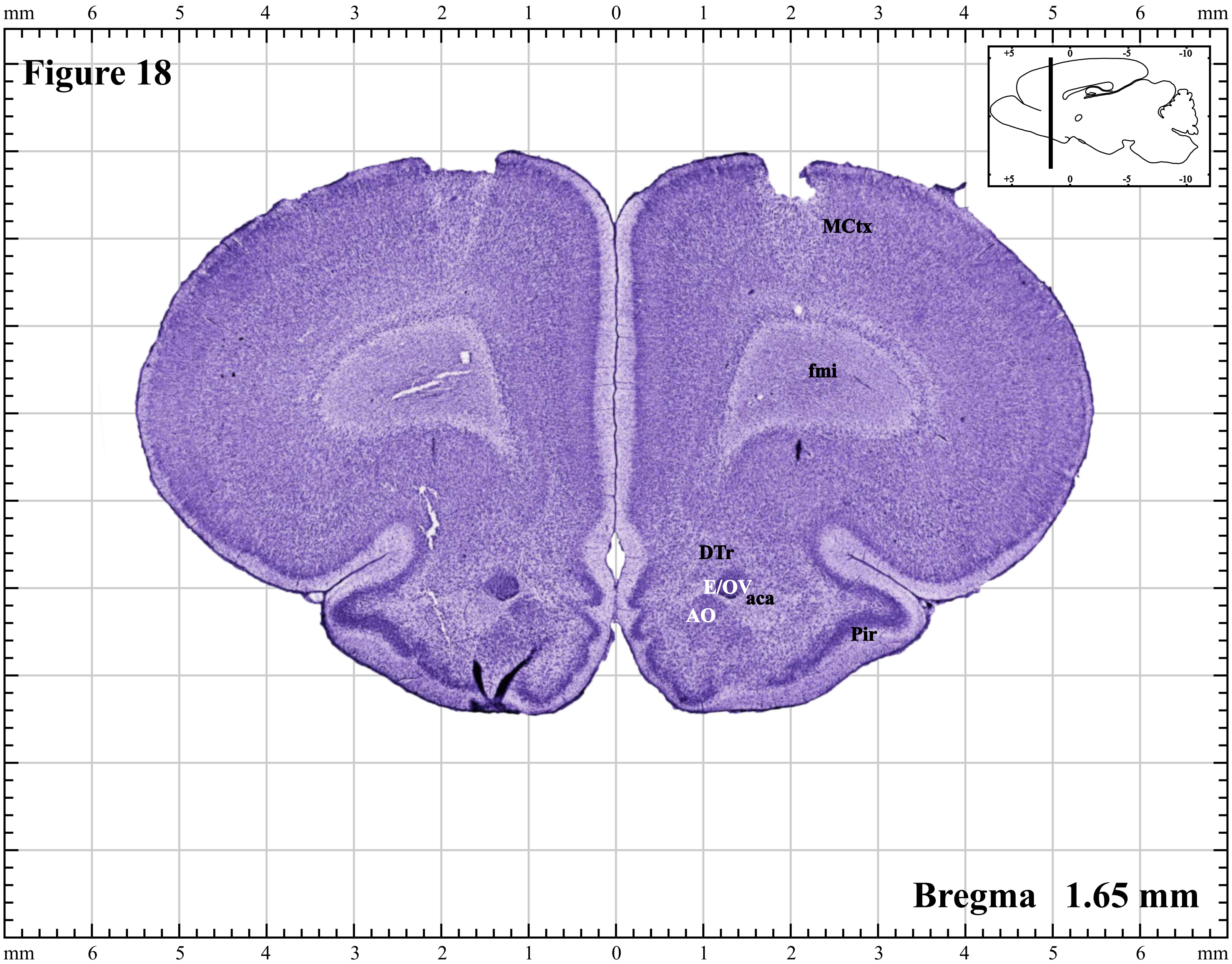

aca anterior commissure, anterior part
AO anterior olfactory nuclues
DTr dorsal transition zone
E/OV ependyma and subependymal layer /olfactory ventricle
fmi forceps major of corpus callosum
MCtx motor cortex
Pir piriform cortex

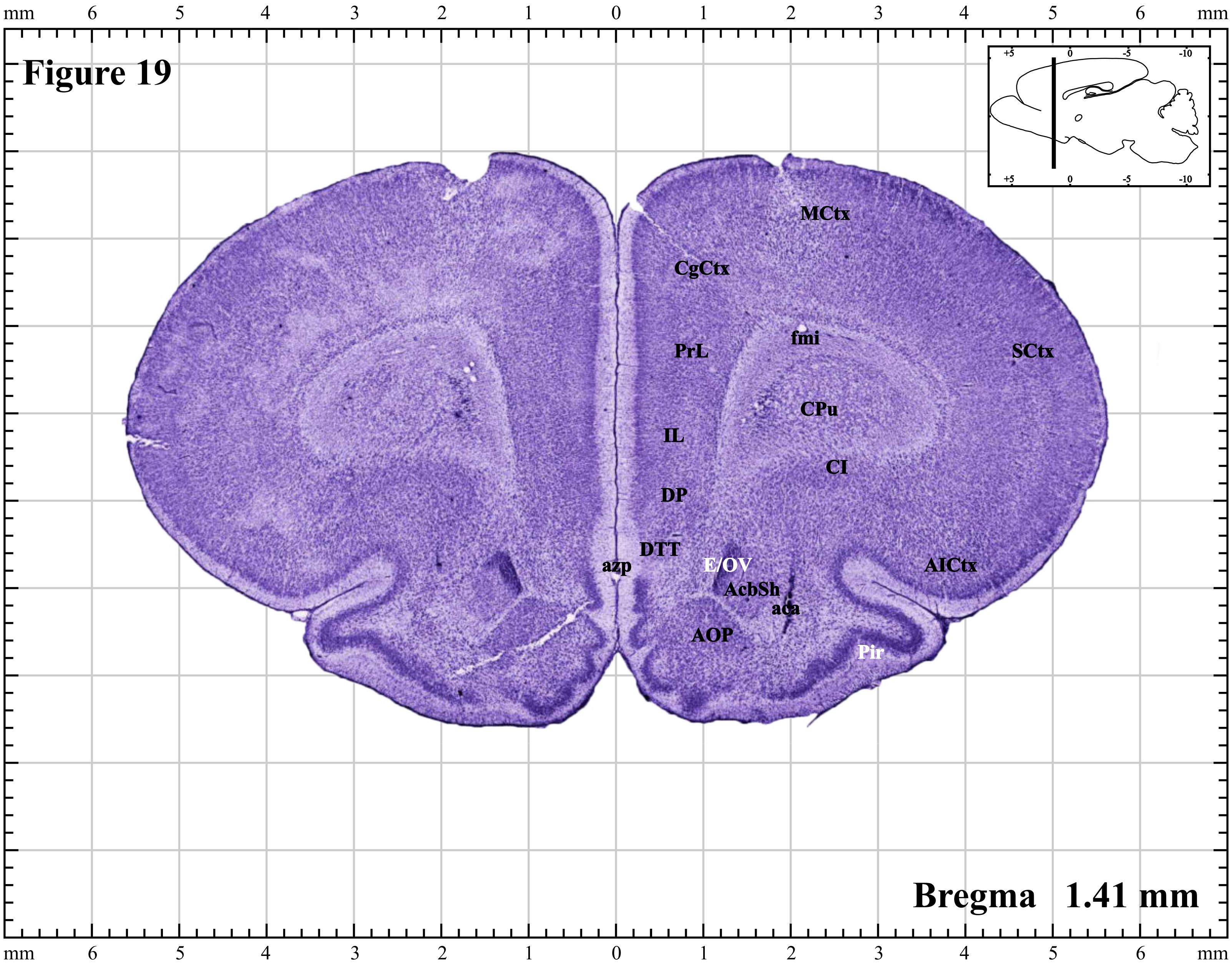

aca anterior commissure, anterior part
azp azygous pericallosal artery
AcbSh accumbens shell
AOP anterior olfactory nucleus, posterior part
AICtx agranular insular cortex
CgCtx cingulate cortex
CI claustrum
CPu caudate putamen (striatum)
DP dorsal peduncular cortex
DTT dorsal tenia tecta
E/OV ependyma and subependymal layer/ olfactory ventricle
fmi forceps major of corpus callosum
IL infralimbic cortex
MCtx motor cortex
Pir piriform cortex
PrL prelimbic cortex
SCtx somatosensory cortex

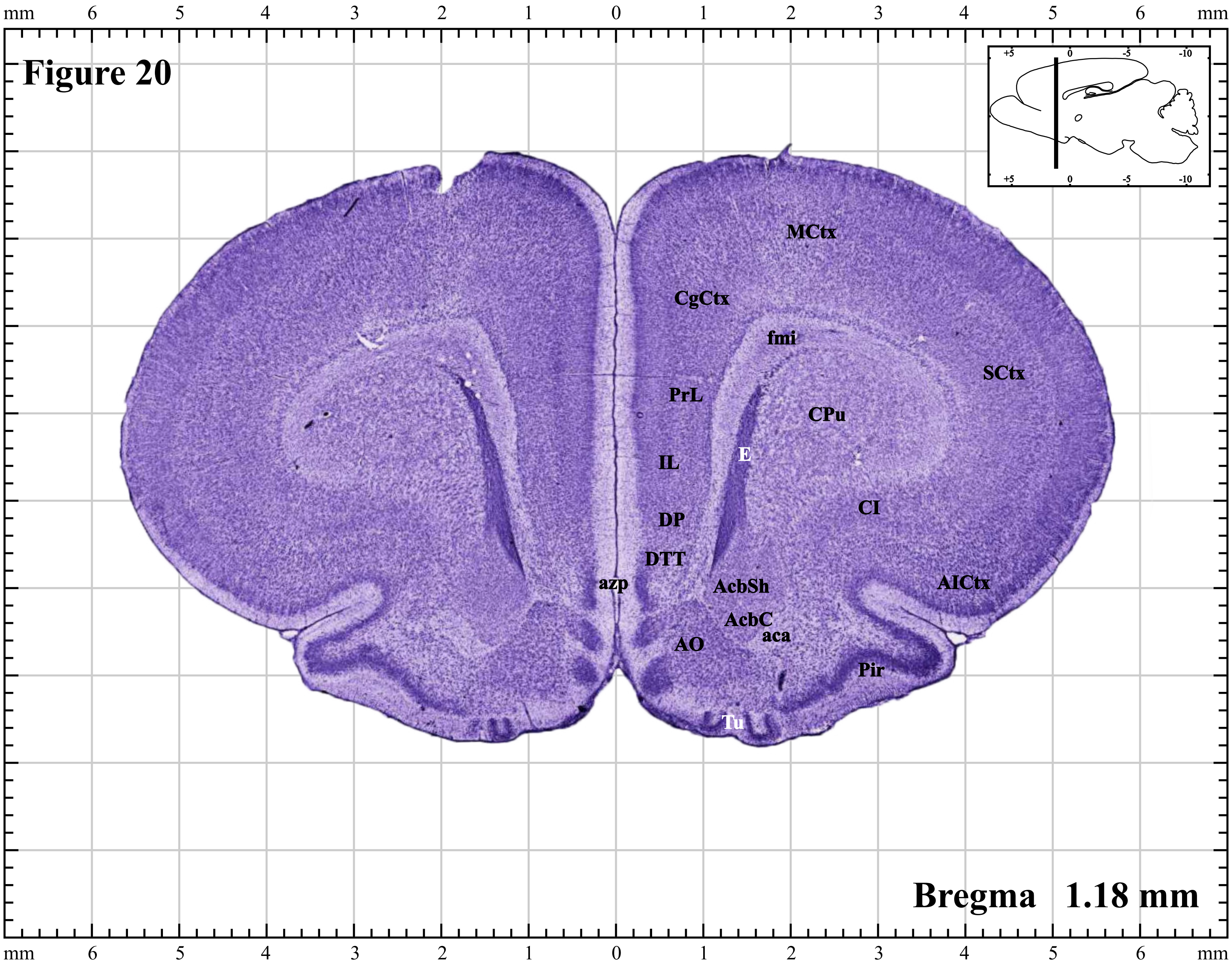

azp azygous pericallosal artery
aca anterior commissure, anterior part
AcbC accumbens nucleus, core
AcbSh accumbens shell
AO anterior olfactory nucleus
AICtx agranular insular cortex
CgCtx cingulate cortex
CI claustrum
CPu caudate putamen (striatum)
DP dorsal peduncular cortex
DTT dorsal tenia tecta
IL infralimbic cortex
E ependyma and subependymal layer
fmi forceps major of corpus callosum
MCtx motor cortex
Pir piriform cortex
PrL prelimbic cortex
SCtx somatosensory cortex
Tu olfactory tubercle

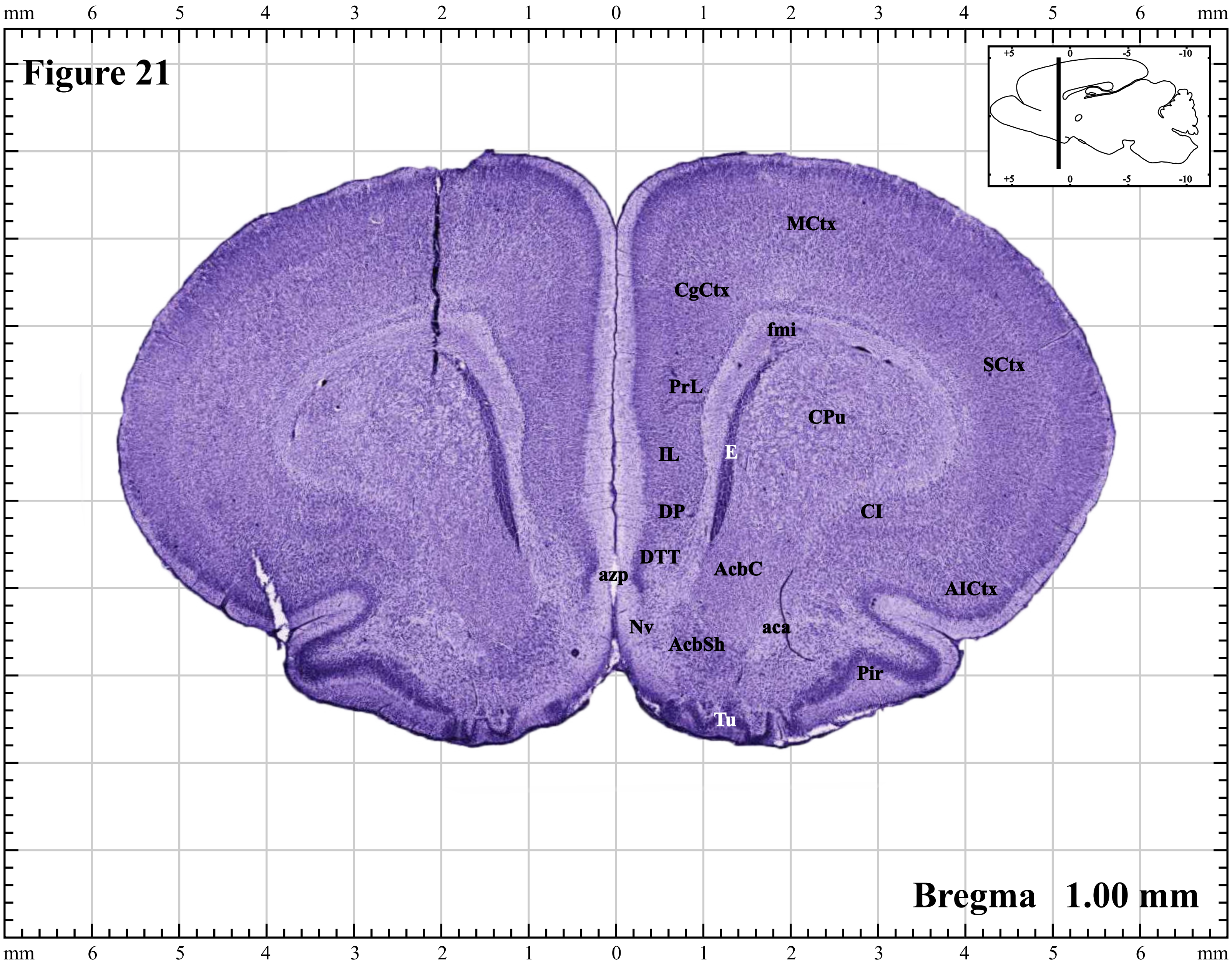

azp azygous pericallosal artery
aca anterior commissure, anterior part
AcbC accumbens nucleus, core
AcbSh accumbens shell
AICtx agranular insular cortex
CgCtx cingulate cortex
CI claustrum
CPu caudate putamen (striatum)
DP dorsal peduncular cortex
DTT dorsal tenia tecta
IL infralimbic cortex
E ependyma and subependymal layer
fmi forceps major of corpus callosum
MCtx motor cortex
Nv navicular nucleus of the basal forebrain
Pir piriform cortex
PrL prelimbic cortex
SCtx somatosensory cortex
Tu olfactory tubercle

Figure 22

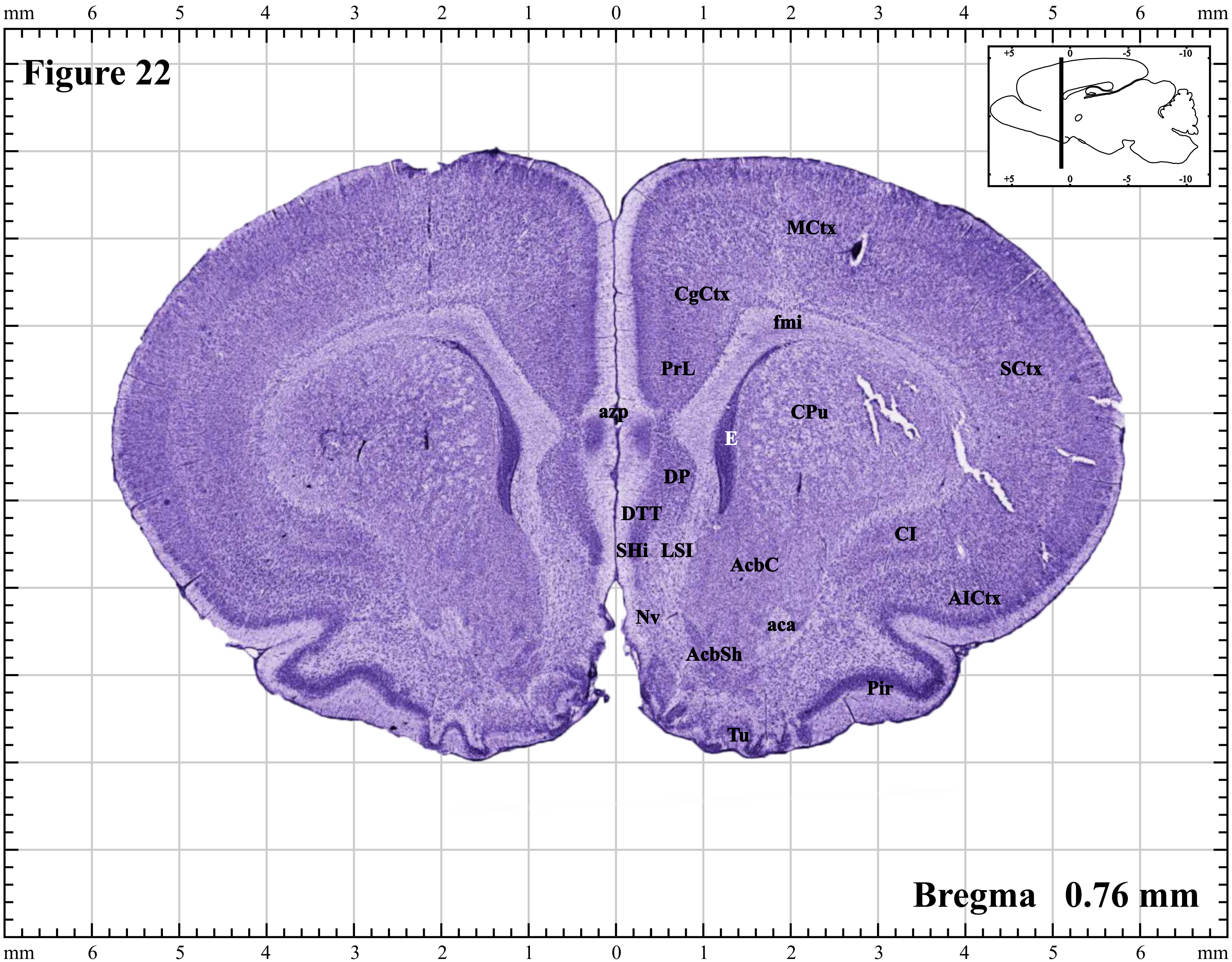

aca anterior commissure, anterior part
azp azygous pericallosal artery
AcbC accumbens nucleus, core
AcbSh accumbens shell
AICtx agranular insular cortex
CgCtx cingulate cortex
CI claustrum
CPu caudate putamen (striatum)
DP dorsal peduncular cortex
DTT dorsal tenia tecta
E ependyma and subependymal layer
fmi forceps major of corpus callosum
LSI lateral septal nucleus, intermediate part
MCtx motor cortex
Nv navicular nucleus of the basal forebrain
Pir piriform cortex
PrL prelimbic cortex
SHi septohippocampal nucleus
SCtx somatosensory cortex
Tu olfactory tubercle

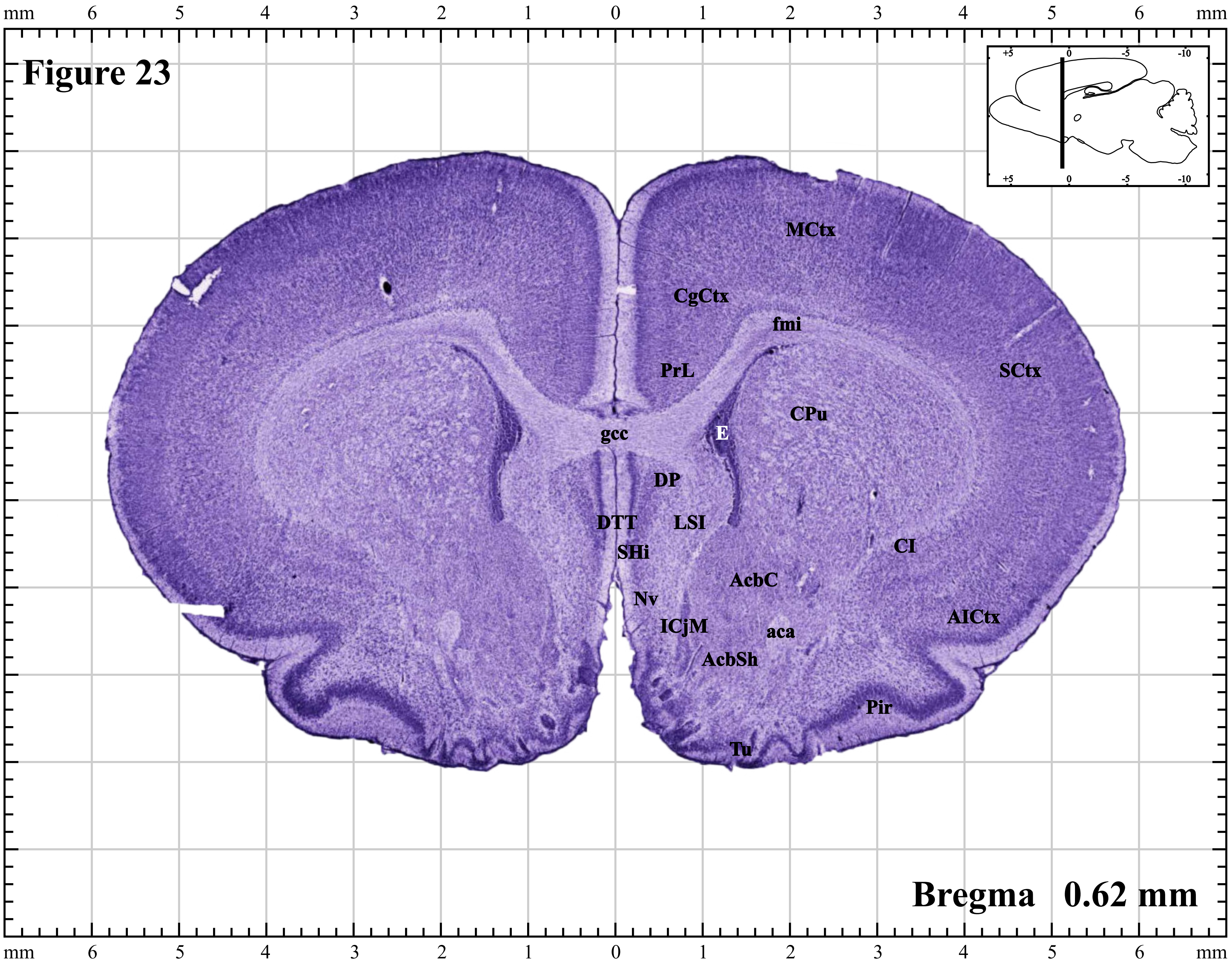

Figure 23

Bregma 0.62 mm

azp azygous pericallosal artery
AcbC accumbens nucleus, core
AcbSh accumbens shell
AICtx agranular insular cortex
CgCtx cingulate cortex
CI claustrum
CPu caudate putamen (striatum)
DP dorsal peduncular cortex
DTT dorsal tenia tecta
E ependyma and subependymal layer
fmi forceps major of corpus callosum
gcc genu of the corpus callosum
ICjM islands of Calleja, major island
LSI lateral septal nucleus, intermediate part
MCtx motor cortex
Nv navicular nucleus of the basal forebrain
Pir piriform cortex
PrL prelimbic cortex
SHi septohippocampal nucleus
SCtx somatosensory cortex
Tu olfactory tubercle

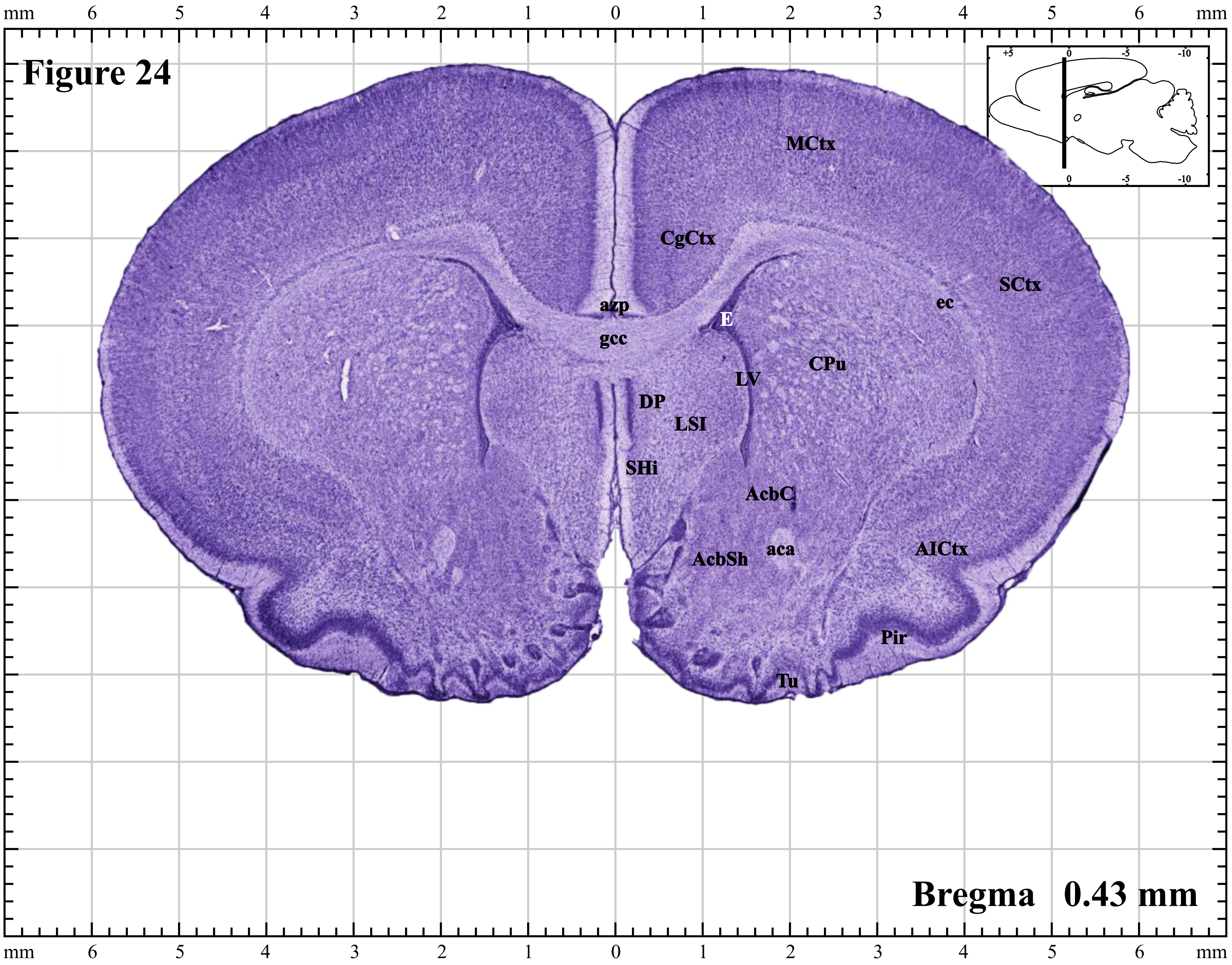

aca anterior commissure, anterior part
azp azygous pericallosal artery
AcbC accumbens nucleus, core
AcbSh accumbens shell
AICtx agranular insular cortex
CgCtx cingulate cortex
CPu caudate putamen (striatum)
DP dorsal peduncular cortex
ec external capsule
E ependyma and subependymal layer
gcc genu of the corpus callosum
fmi forceps major of corpus callosum
LV lateral ventricle
LSI lateral septal nucleus, intermediate part
MCtx motor cortex
Pir piriform cortex
SCtx somatosensory cortex
SHi septohippocampal nucleus
Tu olfactory tubercle

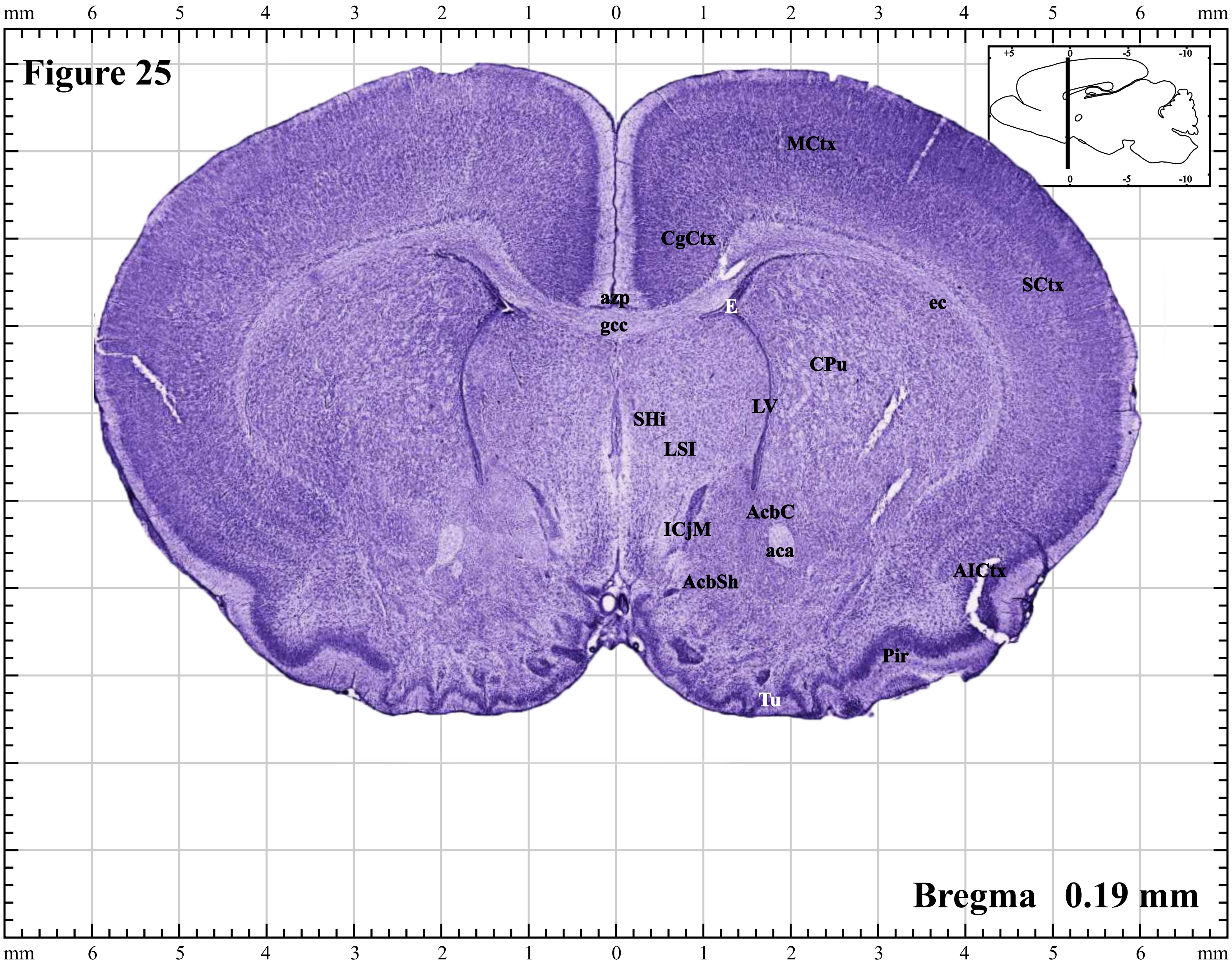

aca anterior commissure, anterior part
azp azygous pericallosal artery
AcbC accumbens nucleus, core
AcbSh accumbens shell
AICtx agranular insular cortex
CgCtx cingulate cortex
CPu caudate putamen (striatum)
ec external capsule
E ependyma and subependymal layer
gcc genu of the corpus callosum
ICjM islands of Calleja, major island
LV lateral ventricle
LSI lateral septal nucleus, intermediate part
MCtx motor cortex
Pir piriform cortex
SCtx somatosensory cortex
SHi septohippocampal nucleus
Tu olfactory tubercle

Figure 26

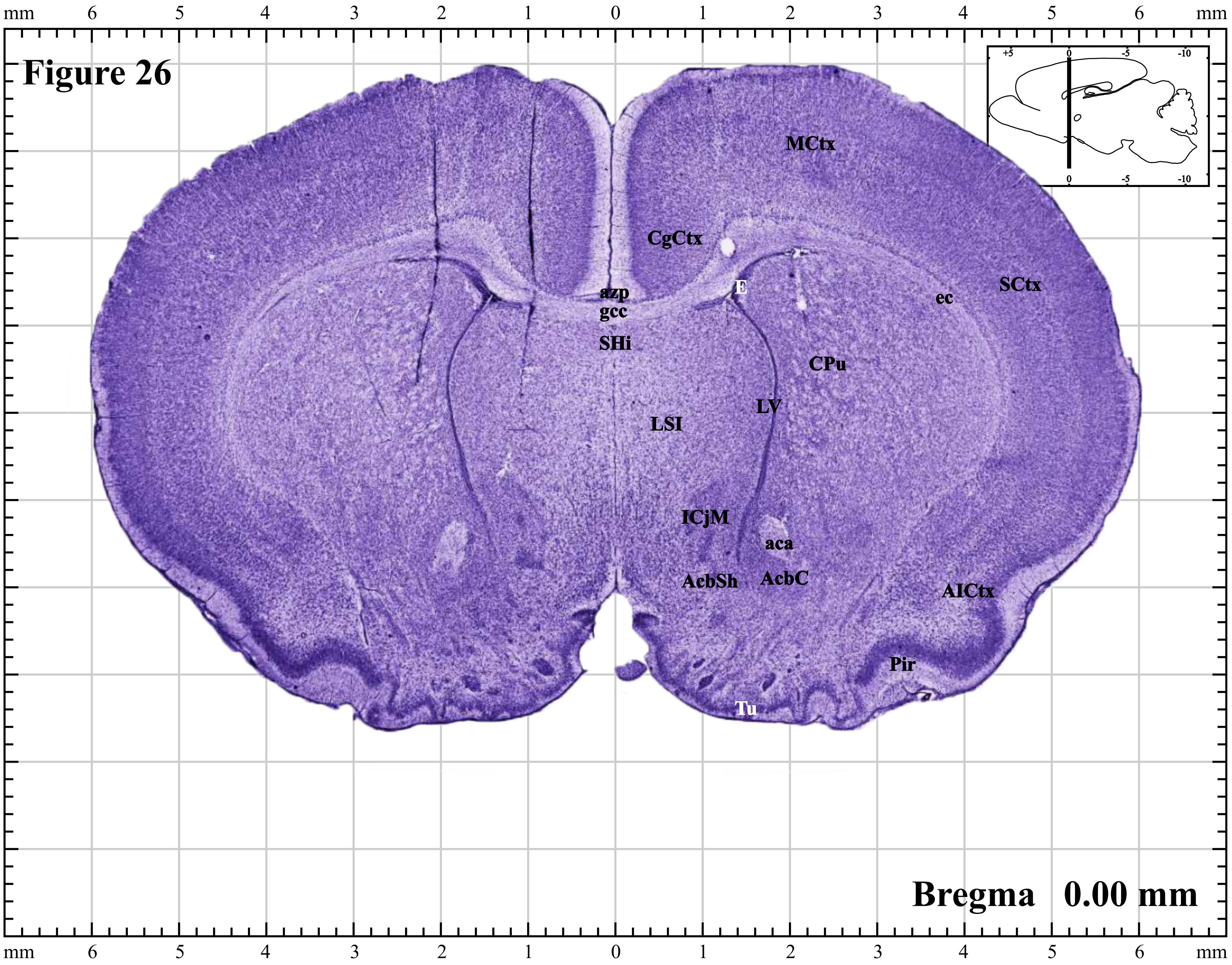

Bregma 0.00 mm

- **aca** anterior commissure, anterior part
- **azp** azygous pericallosal artery
- **AcbC** accumbens nucleus, core
- **AcbSh** accumbens shell
- **AICtx** agranular insular cortex
- **CgCtx** cingulate cortex
- **CPu** caudate putamen (striatum)
- **ec** external capsule
- **E** ependyma and subependymal layer
- **gcc** genu of the corpus callosum
- **ICjM** islands of Calleja, major island
- **LV** lateral ventricle
- **LSI** lateral septal nucleus, intermediate part
- **MCtx** motor cortex
- **Pir** piriform cortex
- **SCtx** somatosensory cortex
- **SHi** septohippocampal nucleus
- **Tu** olfactory tubercle

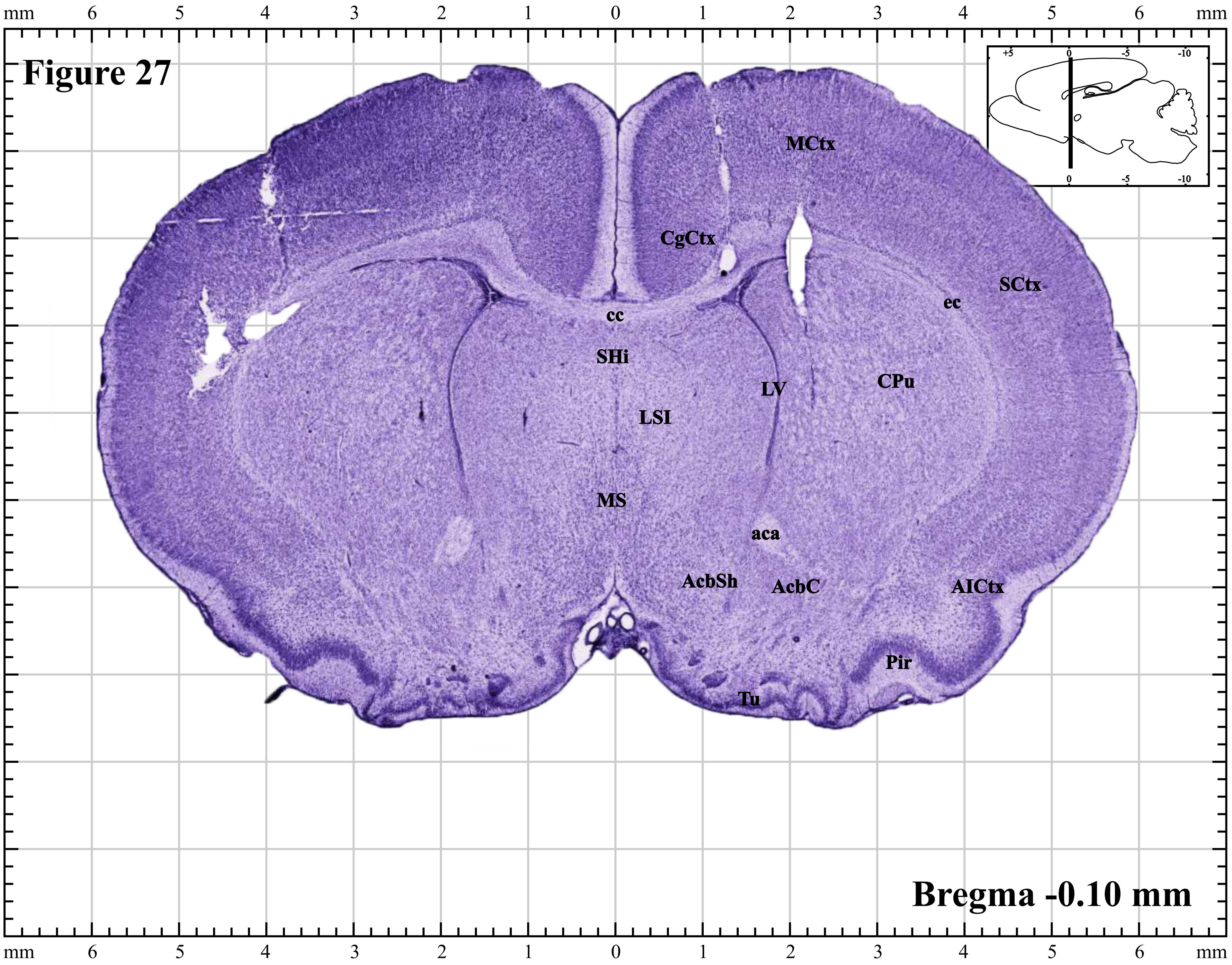

aca anterior commissure, anterior part
AcbC accumbens nucleus, core
AcbSh accumbens shell
AICtx agranular insular cortex
CgCtx cingulate cortex
CPu caudate putamen (striatum)
ec external capsule
cc corpus callosum
LV lateral ventricle
LSI lateral septal nucleus, intermediate part
MCtx motor cortex
MS medial septal nucleus
Pir piriform cortex
SCtx somatosensory cortex
SHi septohippocampal nucleus
Tu olfactory tubercle

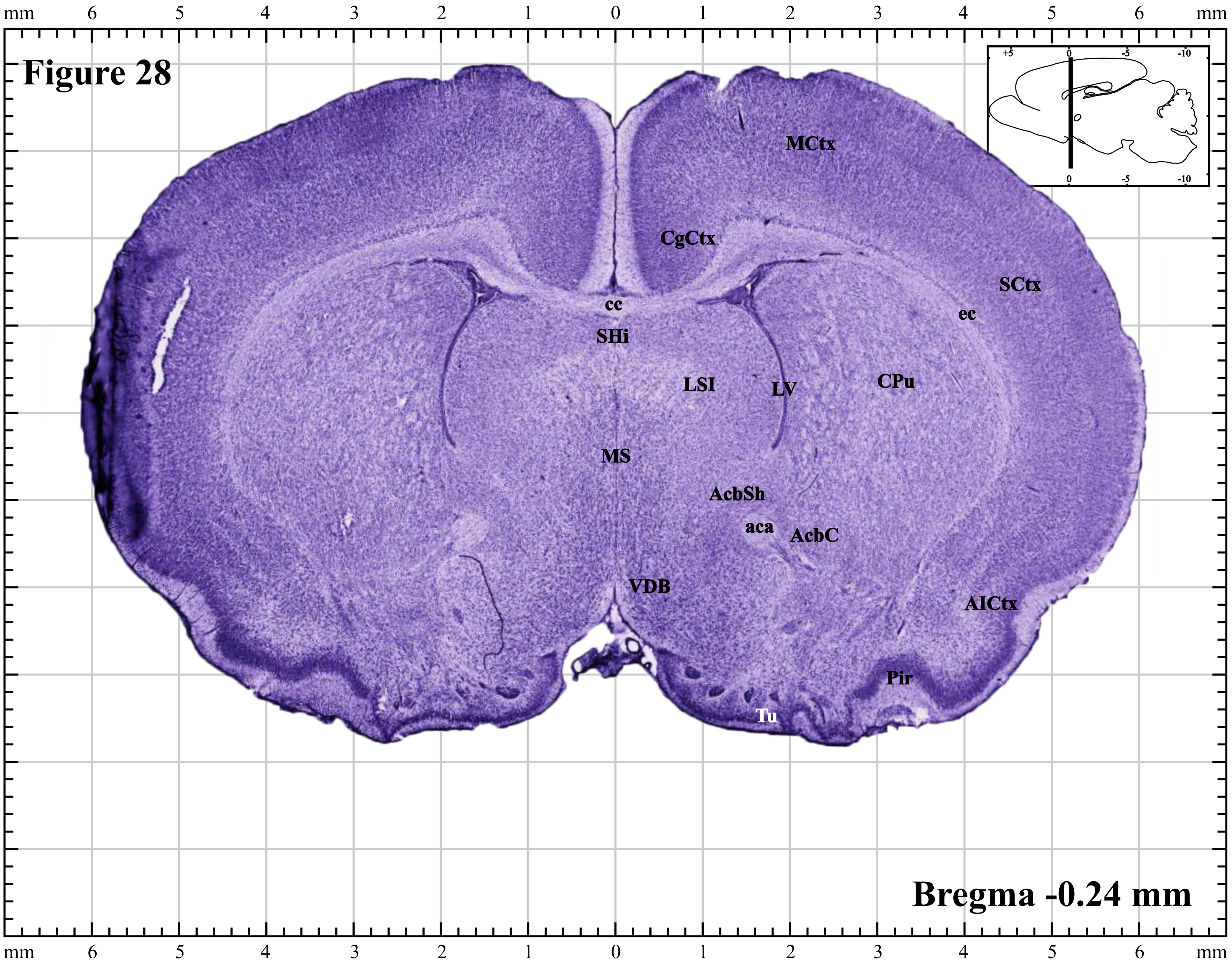

aca anterior commissure, anterior part
AcbC accumbens nucleus, core
AcbSh accumbens shell
AICtx agranular insular cortex
CgCtx cingulate cortex
CPu caudate putamen (striatum)
cc corpus callosum
ec external capsule
LV lateral ventricle
LSI lateral septal nucleus, intermediate part
MCtx motor cortex
MS medial septal nucleus
Pir piriform cortex
SCtx somatosensory cortex
SHi septohippocampal nucleus
VDB nucleus of the vertical limb of the diagonal band
Tu olfactory tubercle

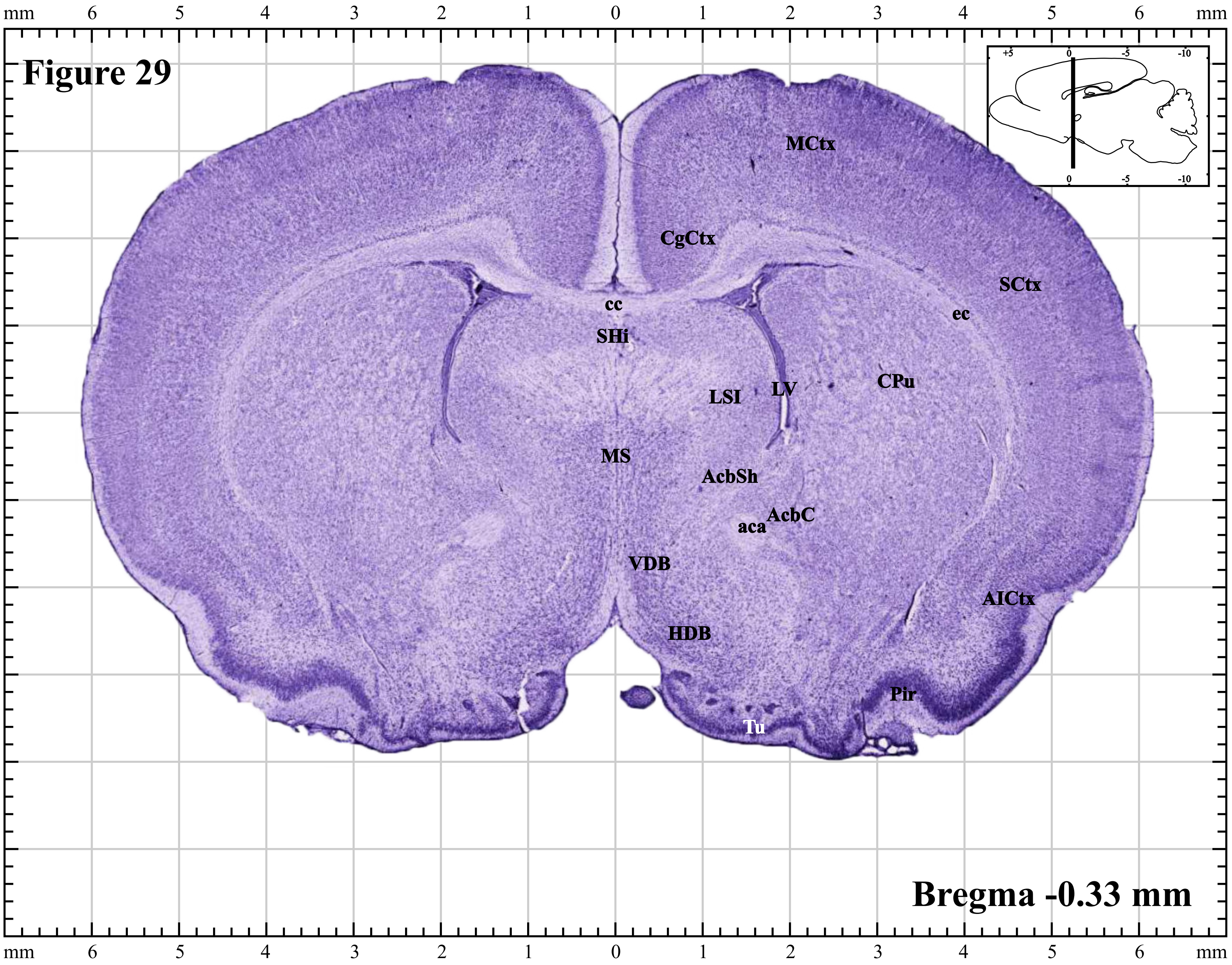

aca anterior commissure, anterior part
AcbC accumbens nucleus, core
AcbSh accumbens shell
AICtx agranular insular cortex
CgCtx cingulate cortex
CPu caudate putamen (striatum)
cc corpus callosum
ec external capsule
HDB nucleus of the horizontal limb of the diagonal band
LV lateral ventricle
LSI lateral septal nucleus, intermediate part
MCtx motor cortex
MS medial septal nucleus
Pir piriform cortex
SCtx somatosensory cortex
SHi septohippocampal nucleus
VDB nucleus of the vertical limb of the diagonal band
Tu olfactory tubercle

Figure 30

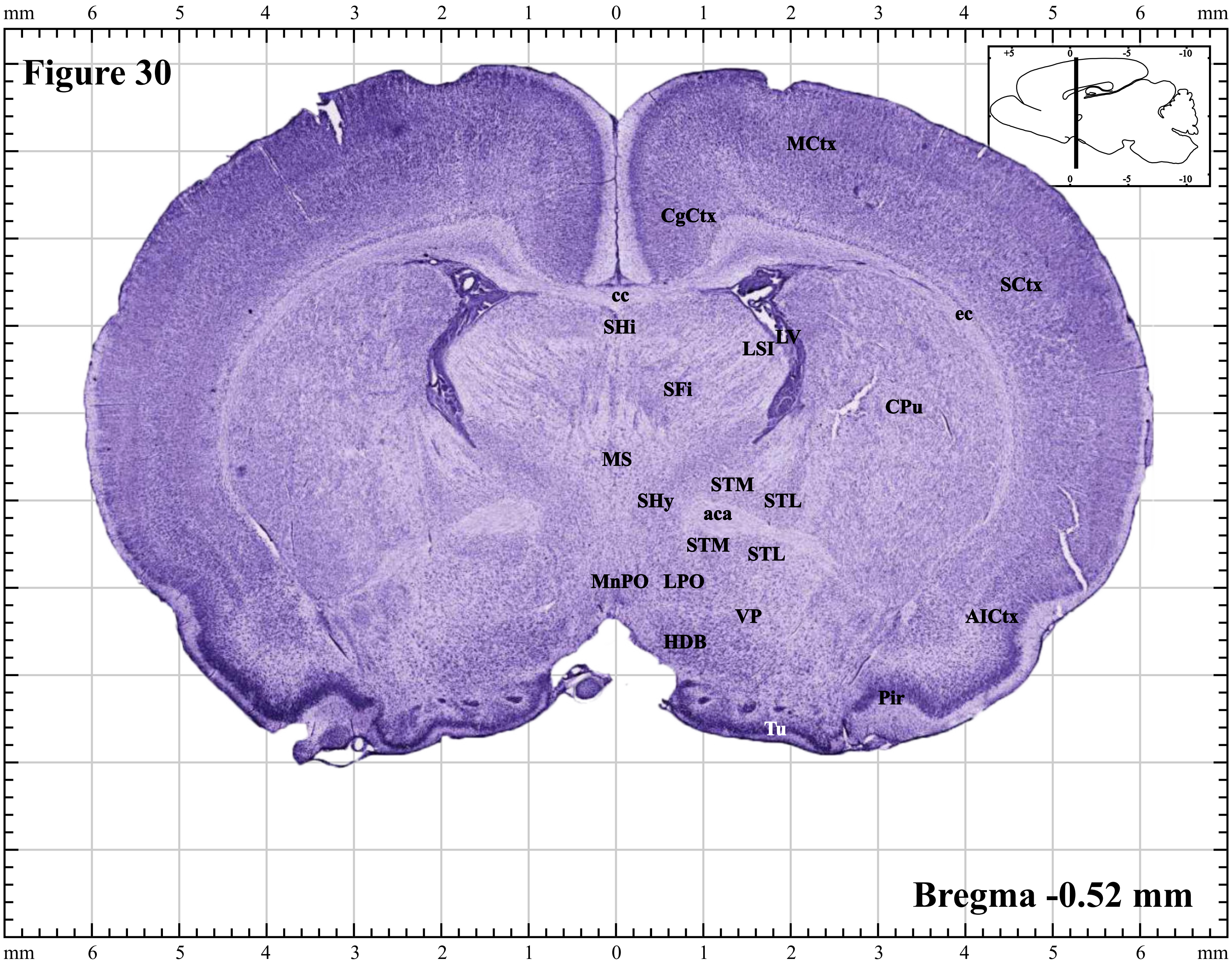

Bregma -0.52 mm

aca anterior commissure, anterior part
AICtx agranular insular cortex
cc corpus callosum
CPu caudate putamen
Cgctx cingulate cortex
ec external capsule
HDB nucleus of the horizontal limb of the diagonal band
LPO lateral preoptic area
LSI lateral septal nucleus, intermediate part
LV lateral ventricle
MnPO median preoptic nucleus
MCtx motor cortex
MS medial septal nucleus
Pir piriform cortex
SHi septohippocampal nucleus
SCtx somatosensory cortex
SHy septohypothalamic nucleus
SFi septofimbrial nucleus
STM bed nucleus of the stria terminalis, medial division
STL bed nucleus of the stria terminalis, lateral division
VP ventral pallidum
Tu olfactory tubercle

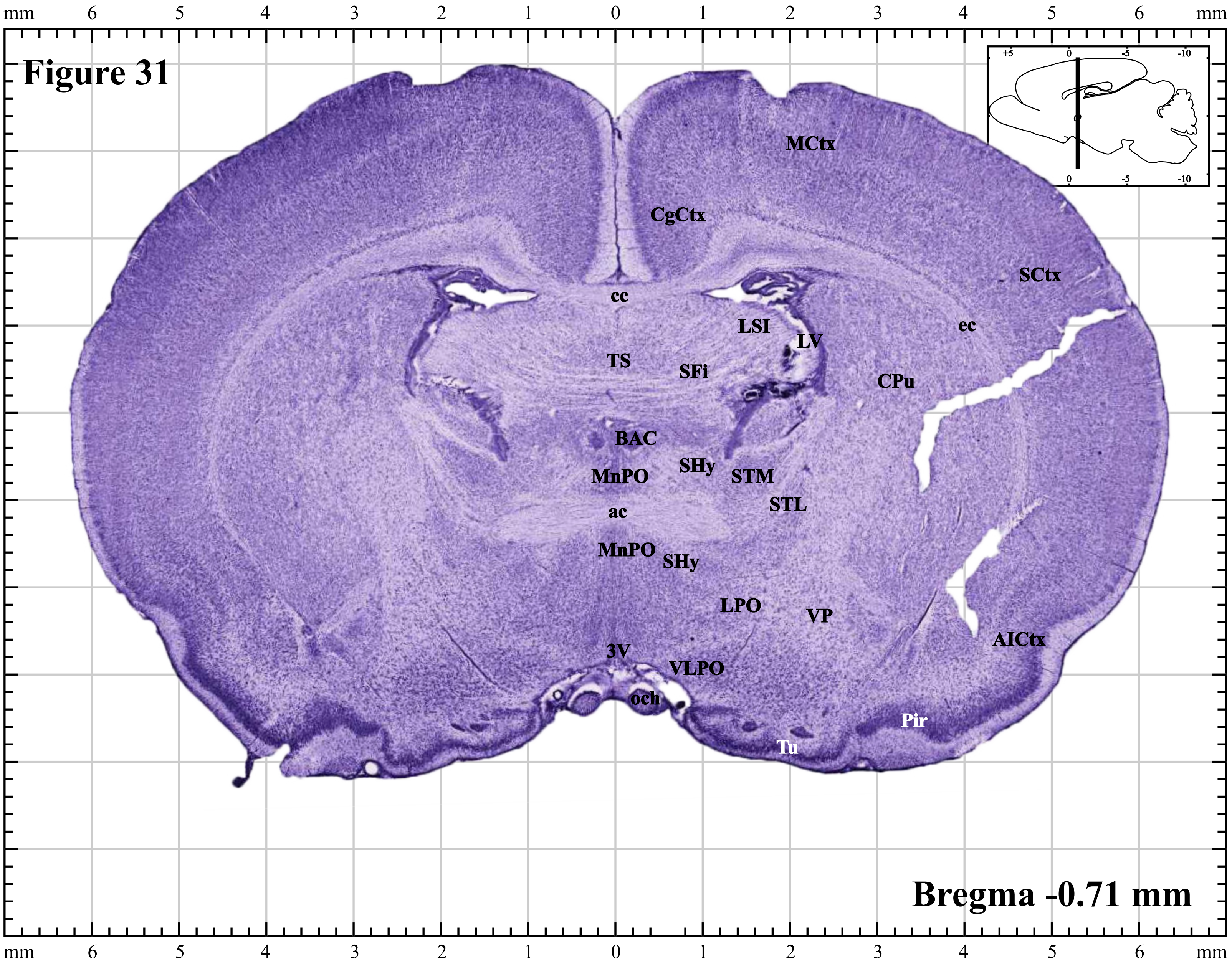

3V 3rd ventricle
ac anterior commissure
AICtx agranular insular cortex
BAC bed nucleus of the anterior commissure
cc corpus callosum
CPu caudate putamen
Cgctx cingulate cortex
ec external capsule
LPO lateral preoptic area
LSI lateral septal nucleus, intermediate part
LV lateral ventricle
MnPO median preoptic nucleus
MCtx motor cortex
MS medial septal nucleus
och optic chiasm
Pir piriform cortex
SCtx somatosensory cortex
SHy septohypothalamic nucleus
SFi septofimbrial nucleus
STL bed nucleus of the stria terminalis, lateral division
STM bed nucleus of the stria terminalis, medial division
TS triangular septal nucleus
Tu olfactory tubercle
VLPO ventrolateral preoptic nucleus
VP ventral pallidum

Figure 32

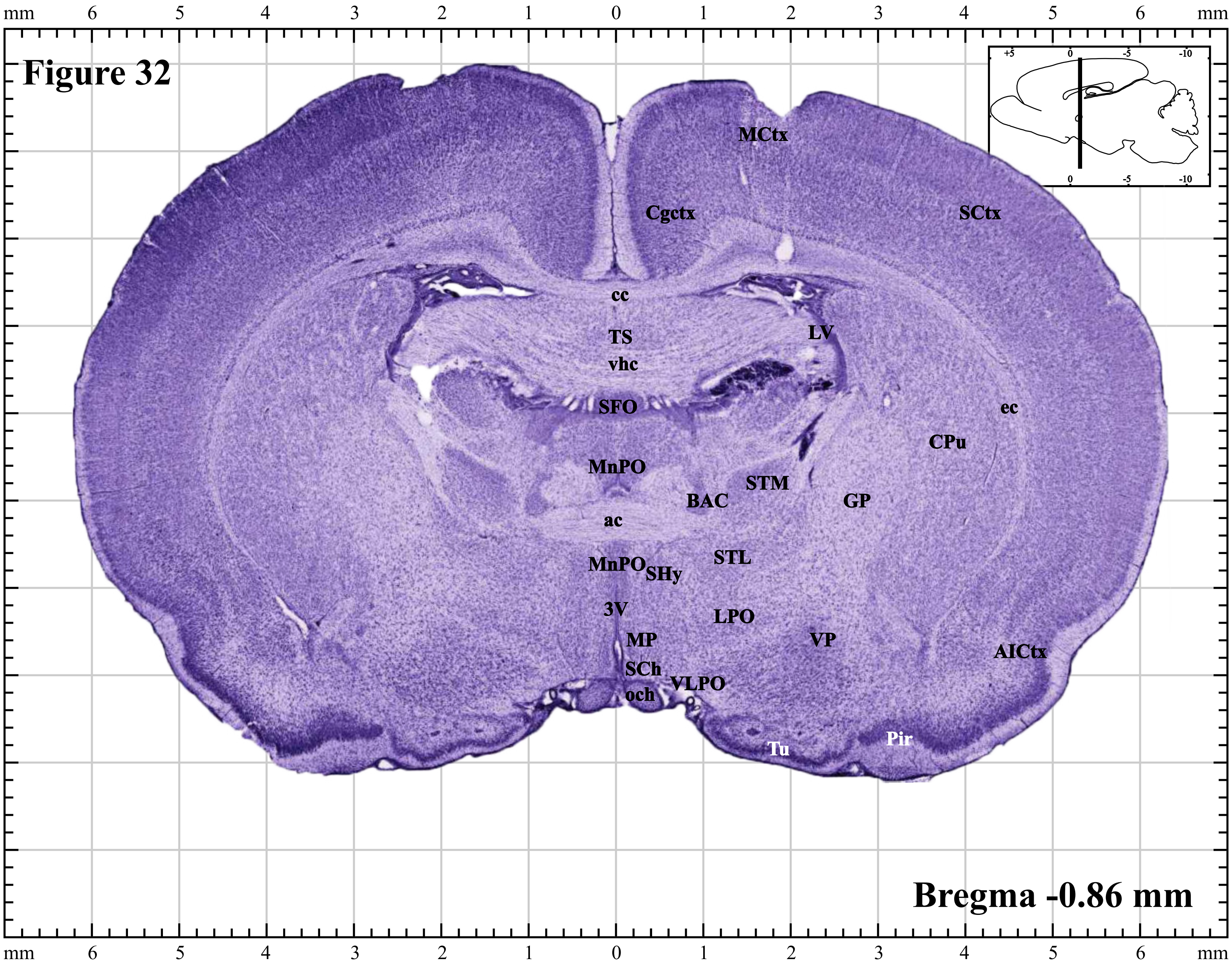

3V 3rd ventricle
ac anterior commissure
AICtx agranular insular cortex
BAC bed nucleus of the anterior commissure
cc corpus callosum
CPu caudate putamen
Cgctx cingulate cortex
ec external capsule
GP globus pallidus
LPO lateral preoptic area
LV lateral ventricle
MCtx motor cortex
MP medial preoptic nucleus
MnPO median preoptic nucleus
och optic chiasm
Pir piriform cortex
SCtx somatosensory cortex
SCh suprachiasmatic nucleus
STM bed nucleus of the stria terminalis, medial division
STL bed nucleus of the stria terminalis, lateral division
SHy septohypothalamic nucleus
SFO subfornical organ
TS triangular septal nucleus
VLPO ventrolateral preoptic nucleus
VP ventral pallidum
vhc ventral hippocampal commissure
Tu olfactory tubercle

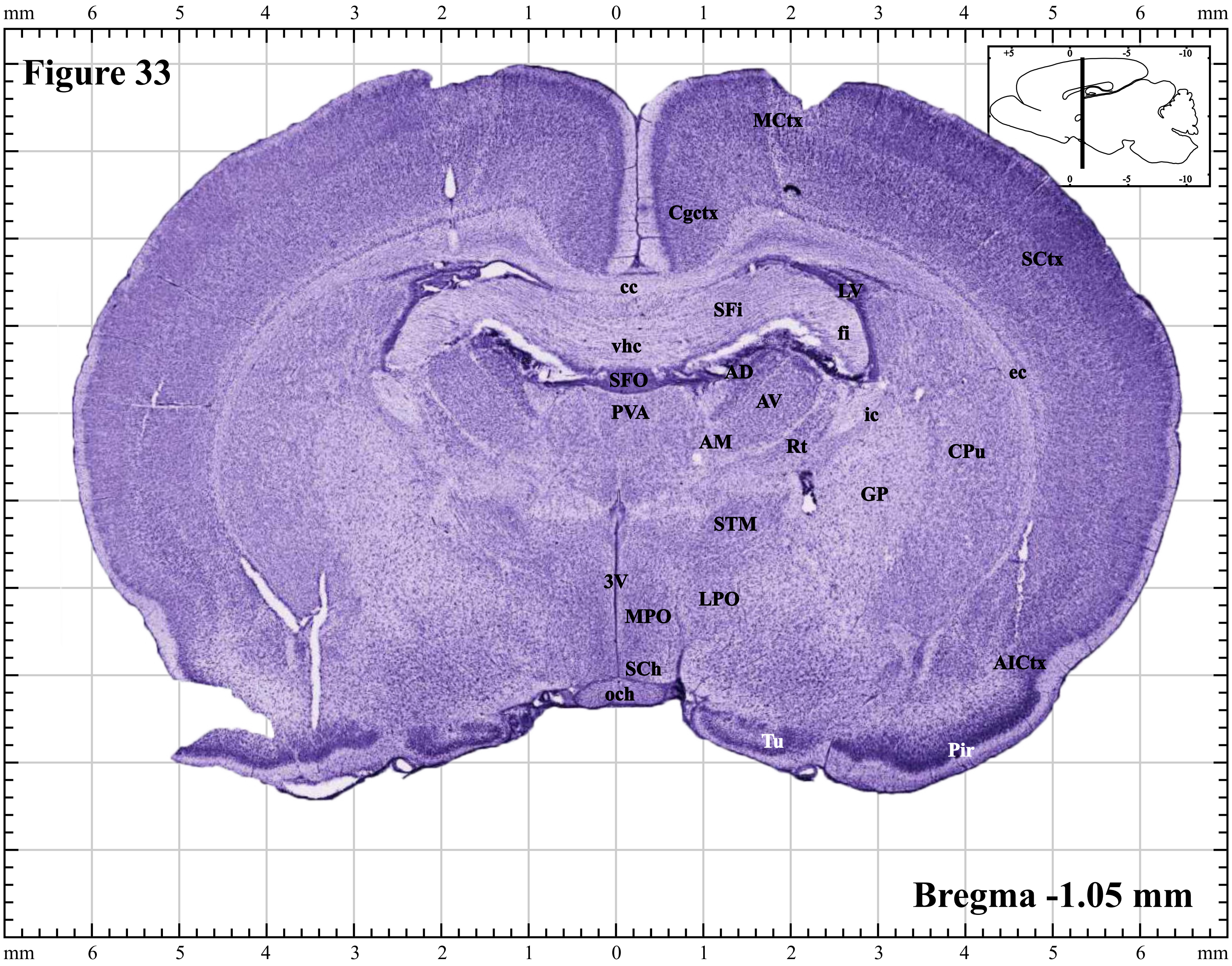

Figure 33

Bregma -1.05 mm

3V 3rd ventricle
AD anterodorsal thalamic nucleus
AM anteromedial thalamic nucleus
AICtx agranular insular cortex
AV anteroventral thalamic nucleus
cc corpus callosum
CPu caudate putamen
Cgctx cingulate cortex
ec external capsule
GP globus pallidus
ic internal capsule
LPO lateral preoptic area
LV lateral ventricle
MPO medial preoptic nucleus
MCtx motor cortex
Rt reticular thalamic nucleus
och optic chiasm
PVA paraventricular thalamic nucleus, anterior part
Pir piriform cortex
SCh suprachiasmatic nucleus
SCtx somatosensory cortex
SFi septofimbrial nucleus
SFO subfornical organ
STM bed nucleus of the stria terminalis, medial division
vhc ventral hippocampal commissure
Tu olfactory tubercle

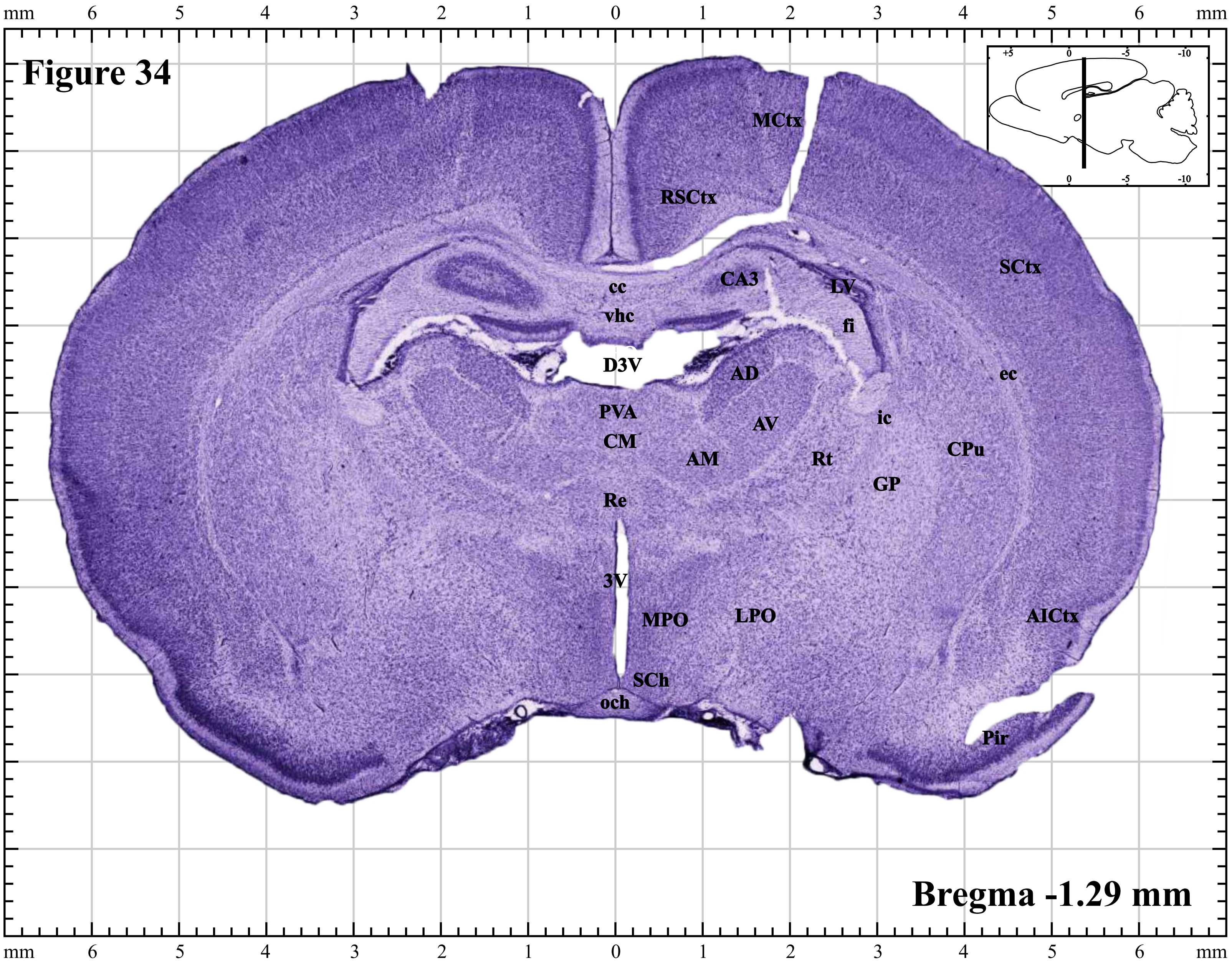

3V 3rd ventricle
AICtx agranular insular cortex
AD anterodorsal thalamic nucleus
AM anteromedial thalamic nucleus
AV anteroventral thalamic nucleus
cc corpus callosum
CPu caudate putamen
CA3 field CA3 of the hippocampus
CM central medial thalamic nucleus
D3V dorsal 3rd ventricle
ec external capsule
fi fimbria of the hippocampus
GP globus pallidus
ic internal capsule
LPO lateral preoptic area
LV lateral ventricle
MCtx motor cortex
MPO medial preoptic nucleus
och optic chiasm
PVA paraventricular thalamic nucleus, anterior part
Pir piriform cortex
Rt reticular thalamic nucleus
Re reuniens thalamic nucleus
RSCtx retrospenial cortex
SCtx somatosensory cortex
SFO subfornical organ
SCh suprachiasmatic nucleus
vhc ventral hippocampal commissure

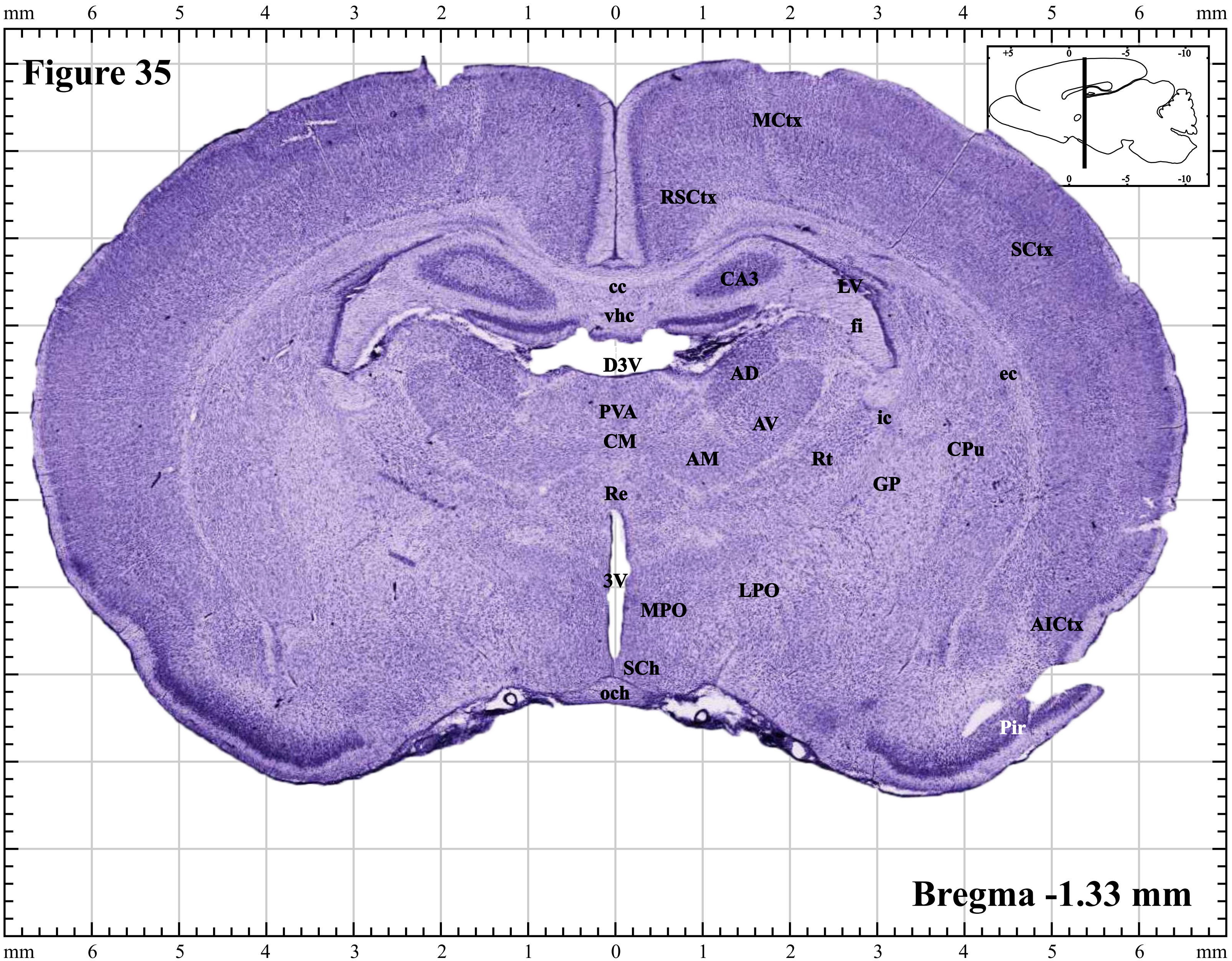

3V 3rd ventricle
AICtx agranular insular cortex
AD anterodorsal thalamic nucleus
AM anteromedial thalamic nucleus
AV anteroventral thalamic nucleus
cc corpus callosum
CPu caudate putamen
CA3 field CA3 of the hippocampus
CM central medial thalamic nucleus
D3V dorsal 3rd ventricle
ec external capsule
fi fimbria of the hippocampus
GP globus pallidus
ic internal capsule
LPO lateral preoptic area
LV lateral ventricle
MCtx motor cortex
MPO medial preoptic nucleus
och optic chiasm
PVA paraventricular thalamic nucleus, anterior part
Pir piriform cortex
Rt reticular thalamic nucleus
Re reuniens thalamic nucleus
RSCtx retrospenial cortex
SCtx somatosensory cortex
SFO subfornical organ
SCh suprachiasmatic nucleus
vhc ventral hippocampal commissure

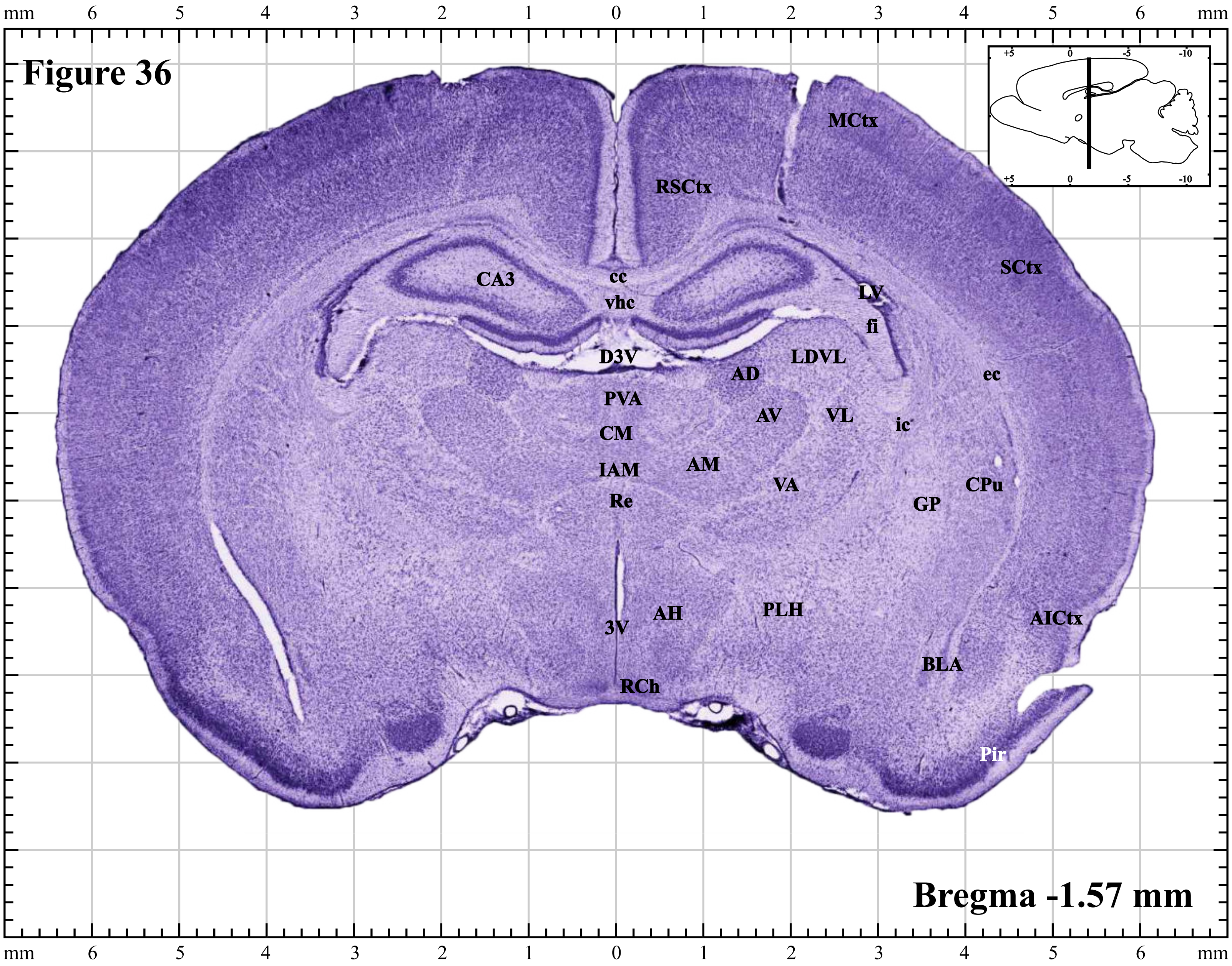

3V 3rd ventricle
AD anterodorsal thalamic nucleus
AH anterior hypothalamic area
AM anteromedial thalamic nucleus
AV anteroventral thalamic nucleus
AICtx agranular insular cortex
BLA basolateral amygdaloid nucleus, anterior part
cc corpus callosum
CPu caudate putamen
CA3 field CA3 of the hippocampus
CM central medial thalamic nucleus
D3V dorsal 3rd ventricle
ec external capsule
fi fimbria of the hippocampus
GP globus pallidus
ic internal capsule
IAM interanteromedial thalamic nucleus
LDVL laterodorsal thalamic nucleus, ventrolateral part
LV lateral ventricle
MCtx motor cortex
Pir piriform cortex
PVA paraventricular thalamic nucleus, anterior part
PLH peduncular part of lateral hypothalamus
RCh retrochiasmatic area
Re reuniens thalamic nucleus
RSCtx retrospenial cortex
SCtx somatosensory cortex
vhc ventral hippocampal commissure
VA ventral anterior thalamic nucleus
VL ventrolateral thalamic nucleus

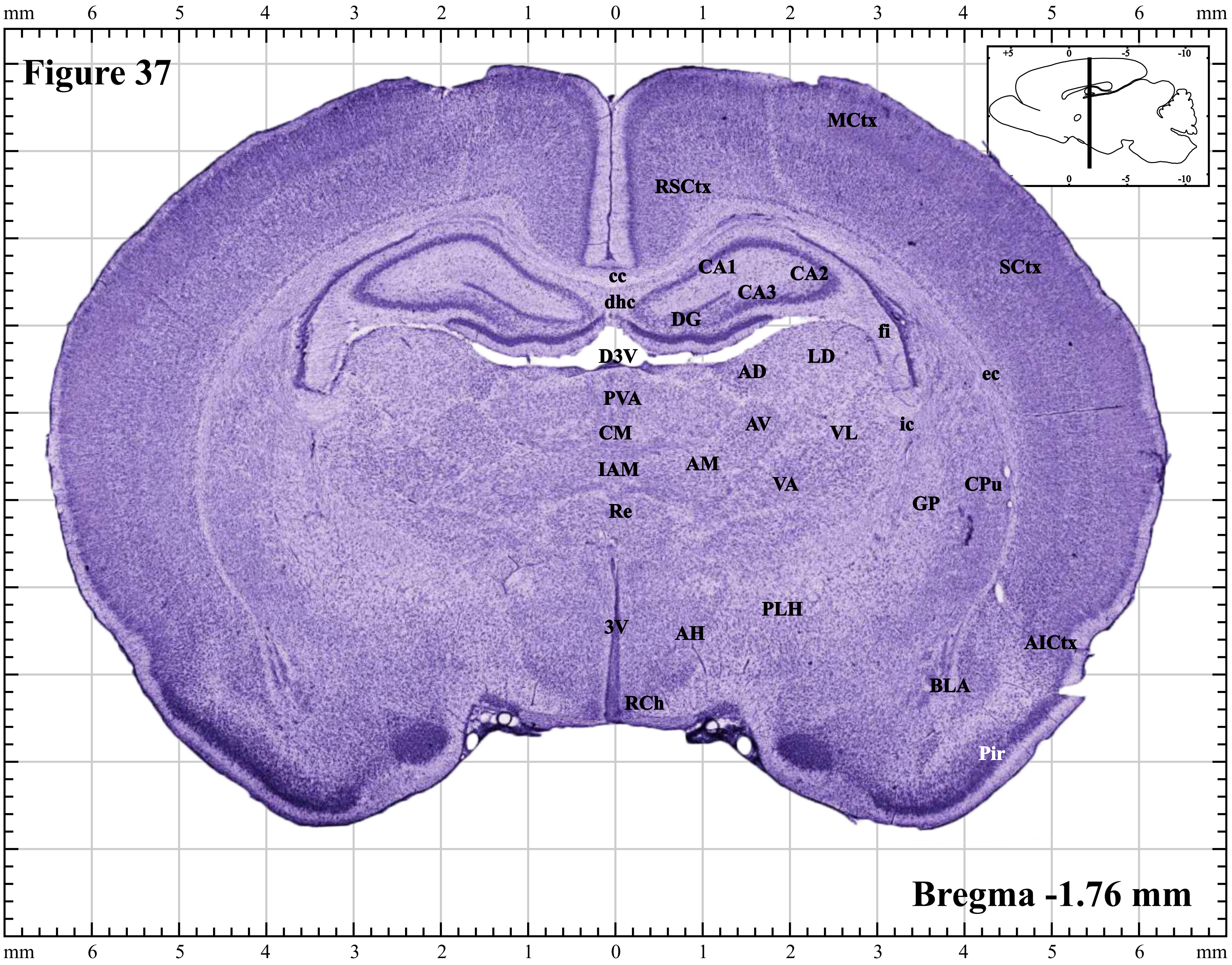

3V 3rd ventricle
AD anterodorsal thalamic nucleus
AH anterior hypothalamic area
AM anteromedial thalamic nucleus
AV anteroventral thalamic nucleus
AICtx agranular insular cortex
BLA basolateral amygdaloid nucleus, anterior part
cc corpus callosum
CPu caudate putamen
CA1 field CA1 of the hippocampus
CA2 field CA2 of the hippocampus
CA3 field CA3 of the hippocampus
CM central medial thalamic nucleus
D3V dorsal 3rd ventricle
DG dentate gyrus
ec external capsule
fi fimbria of the hippocampus
GP globus pallidus
ic internal capsule
IAM interanteromedial thalamic nucleus
LD laterodorsal thalamic nucleus
MCtx motor cortex
Pir piriform cortex
PVA paraventricular thalamic nucleus, anterior part
PLH peduncular part of lateral hypothalamus
RCh retrochiasmatic area
Re reuniens thalamic nucleus
RSCtx retrospenial cortex
SCtx somatosensory cortex
VA ventral anterior thalamic nucleus
VL ventrolateral thalamic nucleus
dhc dorsal hippocampal commissure

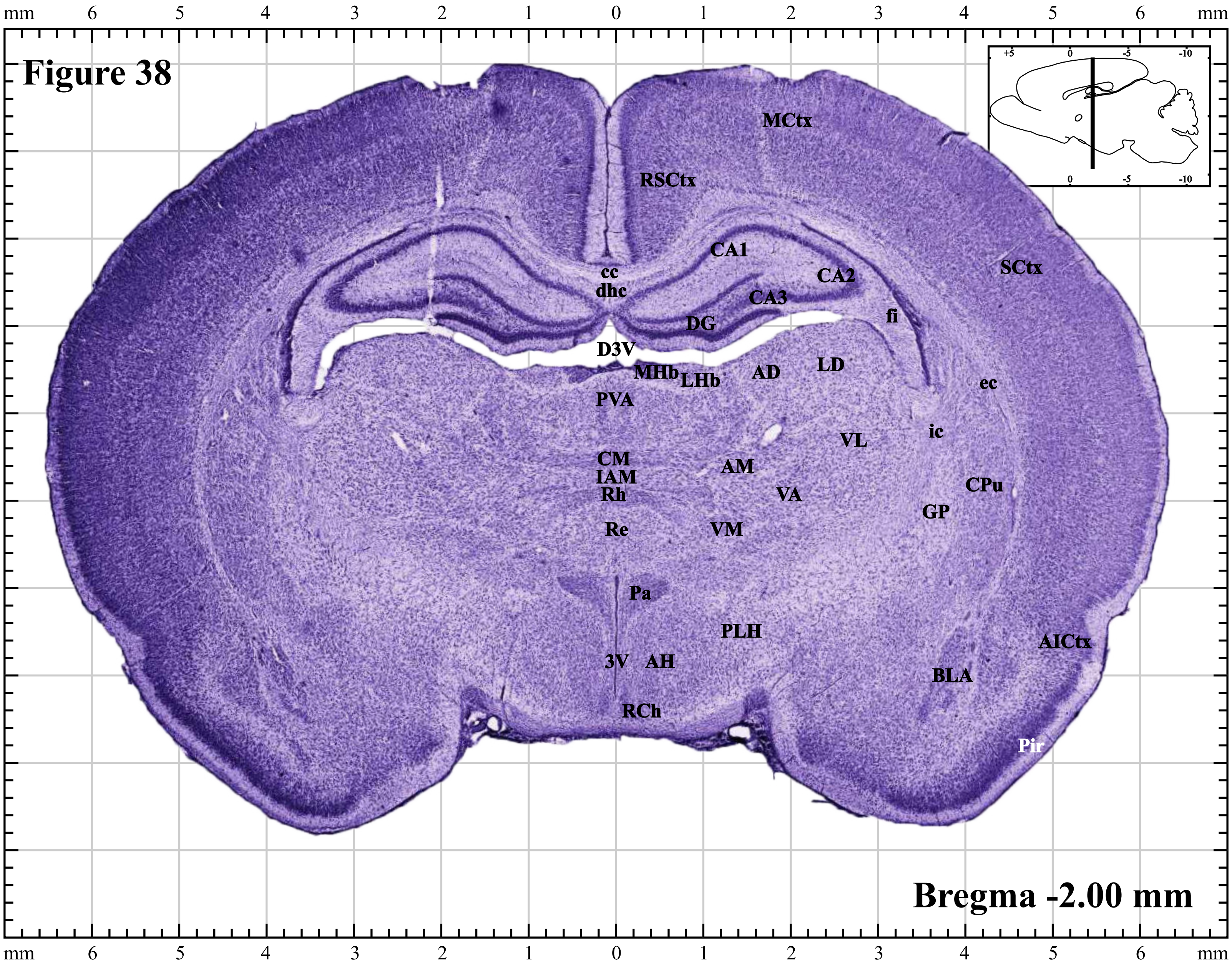

3V 3rd ventricle
AD anterodorsal thalamic nucleus
AH anterior hypothalamic area
AM anteromedial thalamic nucleus
AICtx agranular insular cortex
BLA basolateral amygdaloid nucleus, anterior part
cc corpus callosum
CA1 field CA1 of the hippocampus
CA2 field CA2 of the hippocampus
CA3 field CA3 of the hippocampus
CPu caudate putamen
CM central medial thalamic nucleus
dhc dorsal hippocampal commissure
D3V dorsal 3rd ventricle
ec external capsule
fi fimbria of the hippocampus
GP globus pallidus
ic internal capsule
DG dentate gyrus
IAM interanteromedial thalamic nucleus
LHb lateral habenular nucleus
LD laterodorsal thalamic nucleus,
MHb medial habenular nucleus
MCtx motor cortex
Pir piriform cortex
PVA paraventricular thalamic nucleus, anterior part
Pa paraventricular hypoth nucleus
PLH peduncular part of lateral hypothalamus
Rh rhomboid thalamic nucleus
RCh retrochiasmatic area
Re reuniens thalamic nucleus
RSCtx retrospenial cortex
SCtx somatosensory cortex
VA ventral anterior thalamic nucleus
VM ventromedial thalamic nucleus
VL ventrolateral thalamic nucleus

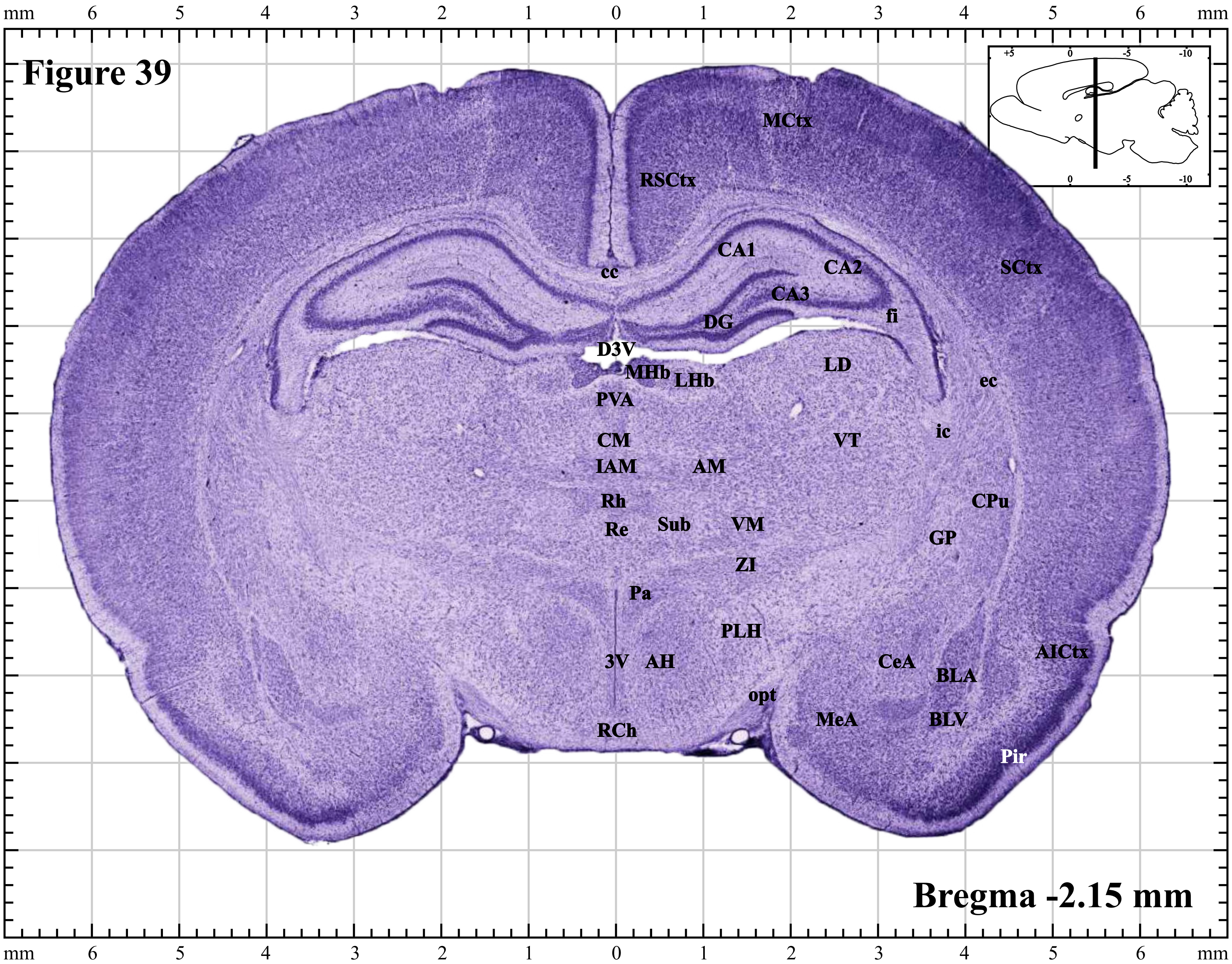

- **3V** 3rd ventricle
- **AH** anterior hypothalamic area
- **AM** anteromedial thalamic nucleus
- **AICtx** agranular insular cortex
- **BLA** basolateral amygdaloid nucleus, anterior part
- **BLV** basolateral amygdaloid nucleus, ventral part
- **cc** corpus callosum
- **CA1** field CA1 of the hippocampus
- **CA2** field CA2 of the hippocampus
- **CA3** field CA3 of the hippocampus
- **CPu** caudate putamen
- **CM** central medial thalamic nucleus
- **CeA** central amygdaloid nucleus
- **D3V** dorsal 3rd ventricle
- **ec** external capsule
- **fi** fimbria of the hippocampus
- **GP** globus pallidus
- **ic** internal capsule
- **DG** dentate gyrus
- **IAM** interanteromedial thalamic nucleus
- **LHb** lateral habenular nucleus
- **LD** laterodorsal thalamic nucleus,
- **MHb** medial habenular nucleus
- **MCtx** motor cortex
- **MeA** medial amygdaloid nucleus
- **Pir** piriform cortex
- **PVA** paraventricular thalamic nucleus, anterior part
- **Pa** paraventricular hypoth nucleus
- **PLH** peduncular part of lateral hypothalamus
- **Rh** rhomboid thalamic nucleus
- **RCh** retrochiasmatic area
- **Re** reuniens thalamic nucleus
- **RSCtx** retrospenial cortex
- **SCtx** somatosensory cortex
- **Sub** submedius thalamic nucleus
- **VT** ventral thalamus
- **VM** ventromedial thalamic nucleus
- **ZI** zona incerta

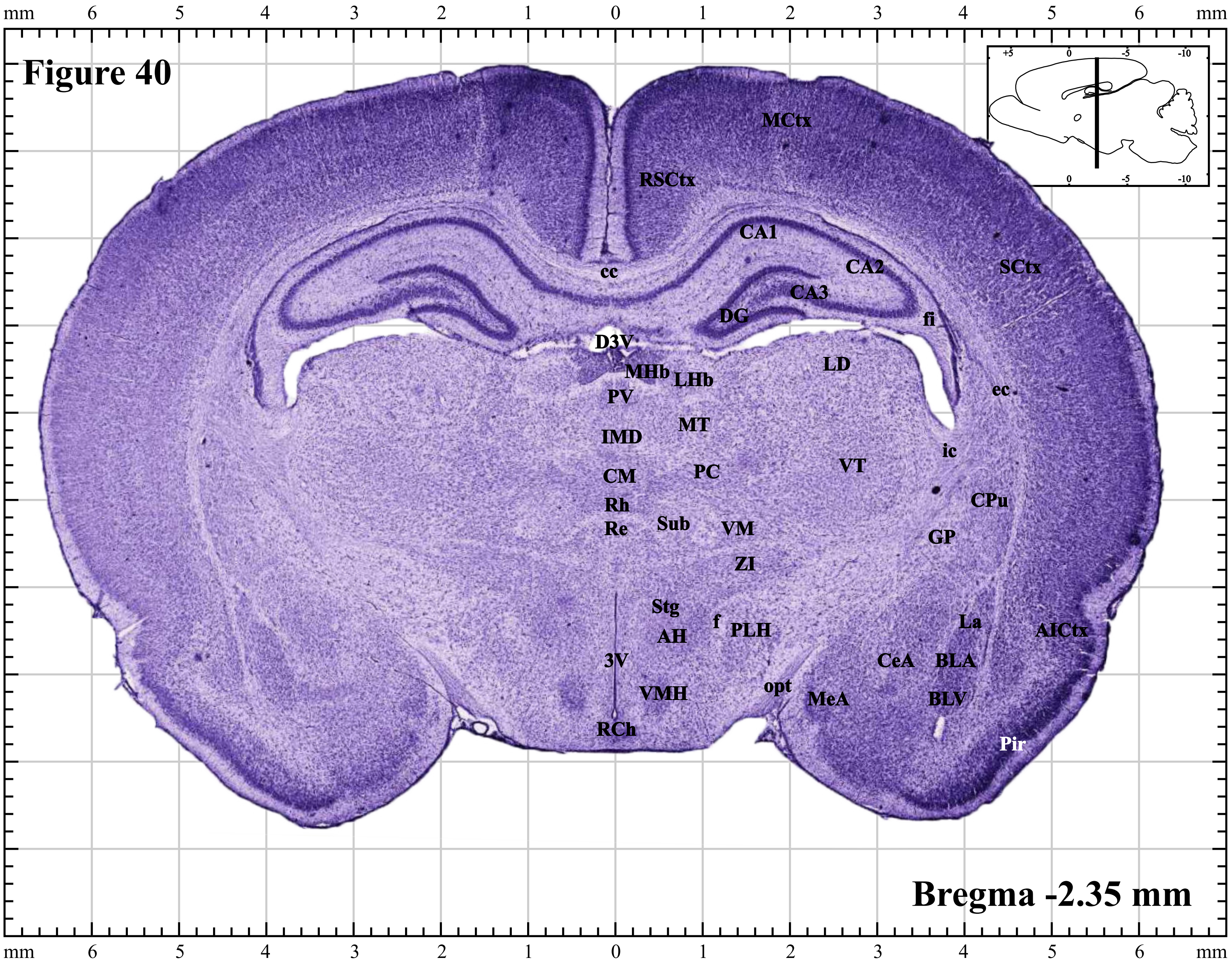

3V 3rd ventricle
AH anterior hypothalamic area
AICtx agranular insular cortex
BLA basolateral amygdaloid nucleus, anterior part
BLV basolateral amygdaloid nucleus, ventral part
cc corpus callosum
CPu caudate putamen
CA1 field CA1 of the hippocampus
CA2 field CA2 of the hippocampus
CA3 field CA3 of the hippocampus
CM central medial thalamic nucleus
CeA central amygdaloid nucleus
D3V dorsal 3rd ventricle
DG dentate gyrus
ec external capsule
f fornix
fi fimbria of the hippocampus
GP globus pallidus
ic internal capsule
IMD intermediodorsal thalamic nucleus
LHb lateral habenular nucleus
LD laterodorsal thalamic nucleus,
MT medial thalamus
MHb medial habenular nucleus
MCtx motor cortex
MeA medial amygdaloid nucleus
opt optic tract
Pir piriform cortex
PC paracentral thalamic nucleus
PLH peduncular part of lateral hypothalamus
Rh rhomboid thalamic nucleus
RCh retrochiasmatic area
Re reuniens thalamic nucleus
RSCtx retrospenial cortex
SCtx somatosensory cortex
Sub submedius thalamic nucleus
Stg stigmoid hypothalamic nucleus
VT ventral thalamus
VM ventromedial thalamic nucleus
VMH ventromedial hypothalamic nucleus
ZI zona incerta

Figure 41

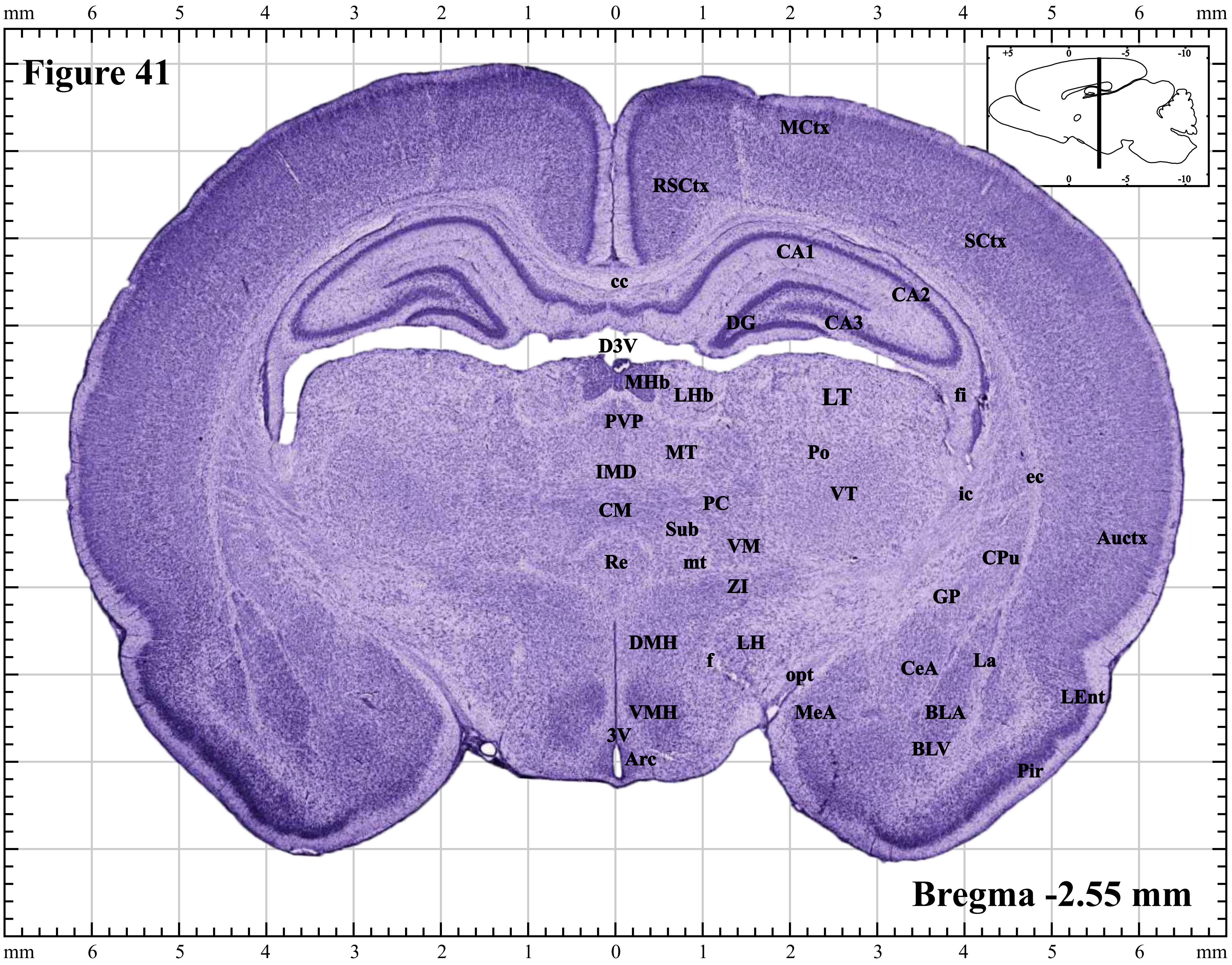

Bregma -2.55 mm

- **3V** medial longitudinal fasciculus
- **Arc** arcuate hypothalamic nucleus
- **Auctx** auditory cortex
- **BLA** basolateral amygdaloid nucleus, anterior part
- **BLV** basolateral amygdaloid nucleus, ventral part
- **CA1** field CA1 of the hippocampus
- **CA2** field CA2 of the hippocampus
- **CA3** field CA3 of the hippocampus
- **cc** corpus callosum
- **CeA** central amygdaloid nucleus
- **CM** central medial thalamic nucleus
- **CPu** caudate putamen
- **D3V** dorsal 3rd ventricle
- **DMH** dorsomedial hypothalamic nucleus
- **DG** dentate gyrus
- **ec** external capsule
- **f** fornix
- **fi** fimbria of the hippocampus
- **GP** globus pallidus
- **ic** internal capsule
- **IMD** intermediodorsal thalamic nucleus
- **La** lat amygdaloid nucleus
- **LEnt** lateral entorhinal cortex
- **LHb** lateral habenular nucleus
- **LH** lateral hypothalamic area
- **LT** lateral thalamus
- **mt** mammillothalamic tract
- **MeA** medial amygdaloid nucleus
- **MHb** medial habenular nucleus
- **MT** medial thalamus
- **opt** optic tract
- **MCtx** motor cortex
- **PC** paracentral thalamic nucleus
- **Pir** piriform cortex
- **PVP** paraventricular thalamic nucleus, posterior part
- **Po** posterior thalamic nuclear group
- **Re** reuniens thalamic nucleus
- **RSCtx** retrospenial cortex
- **SCtx** somatosensory cortex
- **Sub** submedius thalamic nucleus
- **VM** ventromedial thalamic nucleus
- **VMH** ventromedial hypothalamic nucleus
- **VT** ventral thalamus
- **ZI** zona incerta

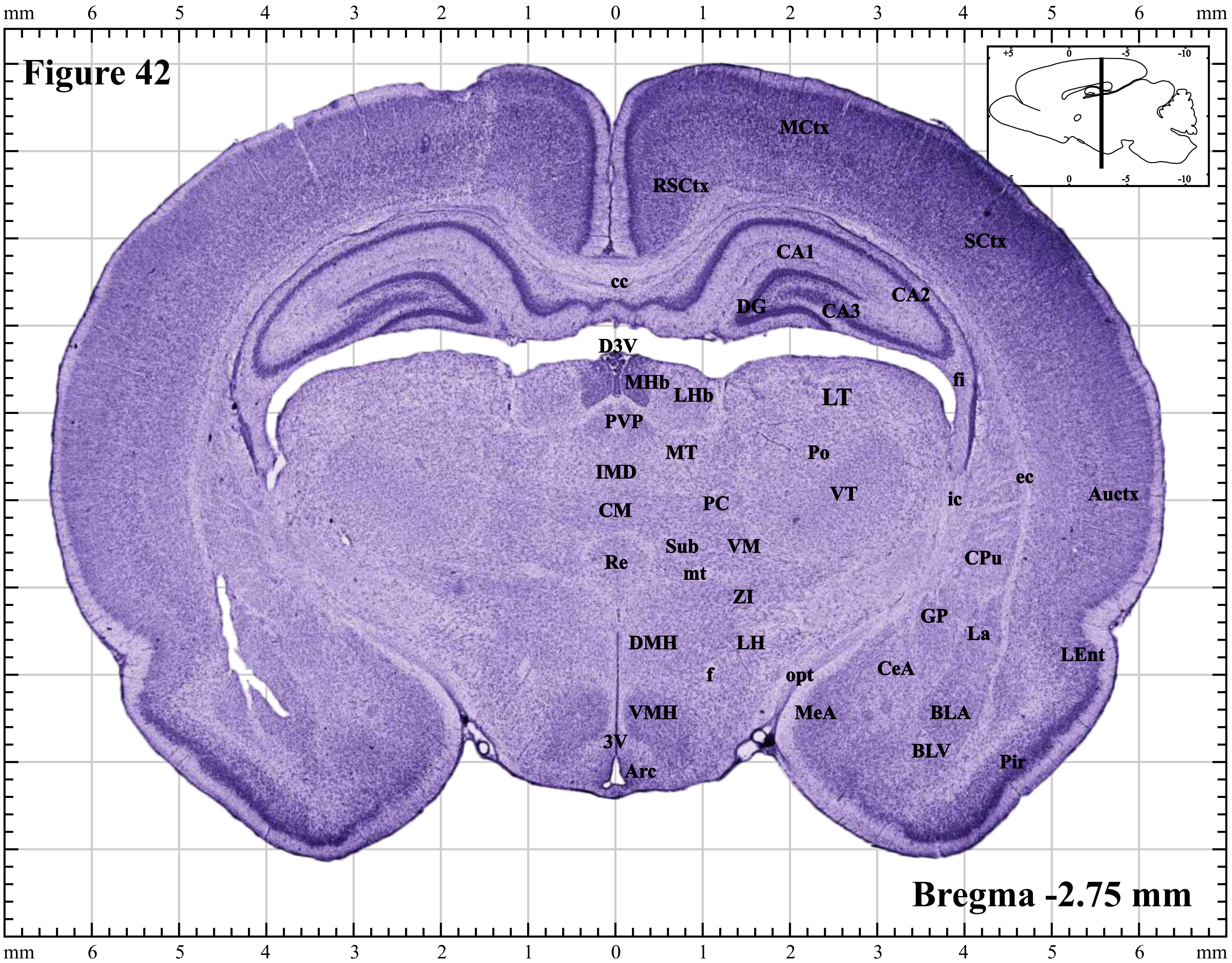

3V medial longitudinal fasciculus
Arc arcuate hypothalamic nucleus
Auctx auditory cortex
BLA basolateral amygdaloid nucleus, anterior part
BLV basolateral amygdaloid nucleus, ventral part
CA1 field CA1 of the hippocampus
CA2 field CA2 of the hippocampus
CA3 field CA3 of the hippocampus
cc corpus callosum
CeA central amygdaloid nucleus
CM central medial thalamic nucleus
CPu caudate putamen
D3V dorsal 3rd ventricle
DMH dorsomedial hypothalamic nucleus
DG dentate gyrus
ec external capsule
f fornix
fi fimbria of the hippocampus
GP globus pallidus
ic internal capsule
IMD intermediodorsal thalamic nucleus
La lat amygdaloid nucleus
LEnt lateral entorhinal cortex
LHb lateral habenular nucleus
LH lateral hypothalamic area
LT lateral thalamus
mt mammillothalamic tract
MeA medial amygdaloid nucleus
MHb medial habenular nucleus
MT medial thalamus
opt optic tract
MCtx motor cortex
PC paracentral thalamic nucleus
Pir piriform cortex
PVP paraventricular thalamic nucleus, posterior part
Po posterior thalamic nuclear group
Re reuniens thalamic nucleus
RSCtx retrospenial cortex
SCtx somatosensory cortex
Sub submedius thalamic nucleus
VM ventromedial thalamic nucleus
VMH ventromedial hypothalamic nucleus
VT ventral thalamus
ZI zona incerta

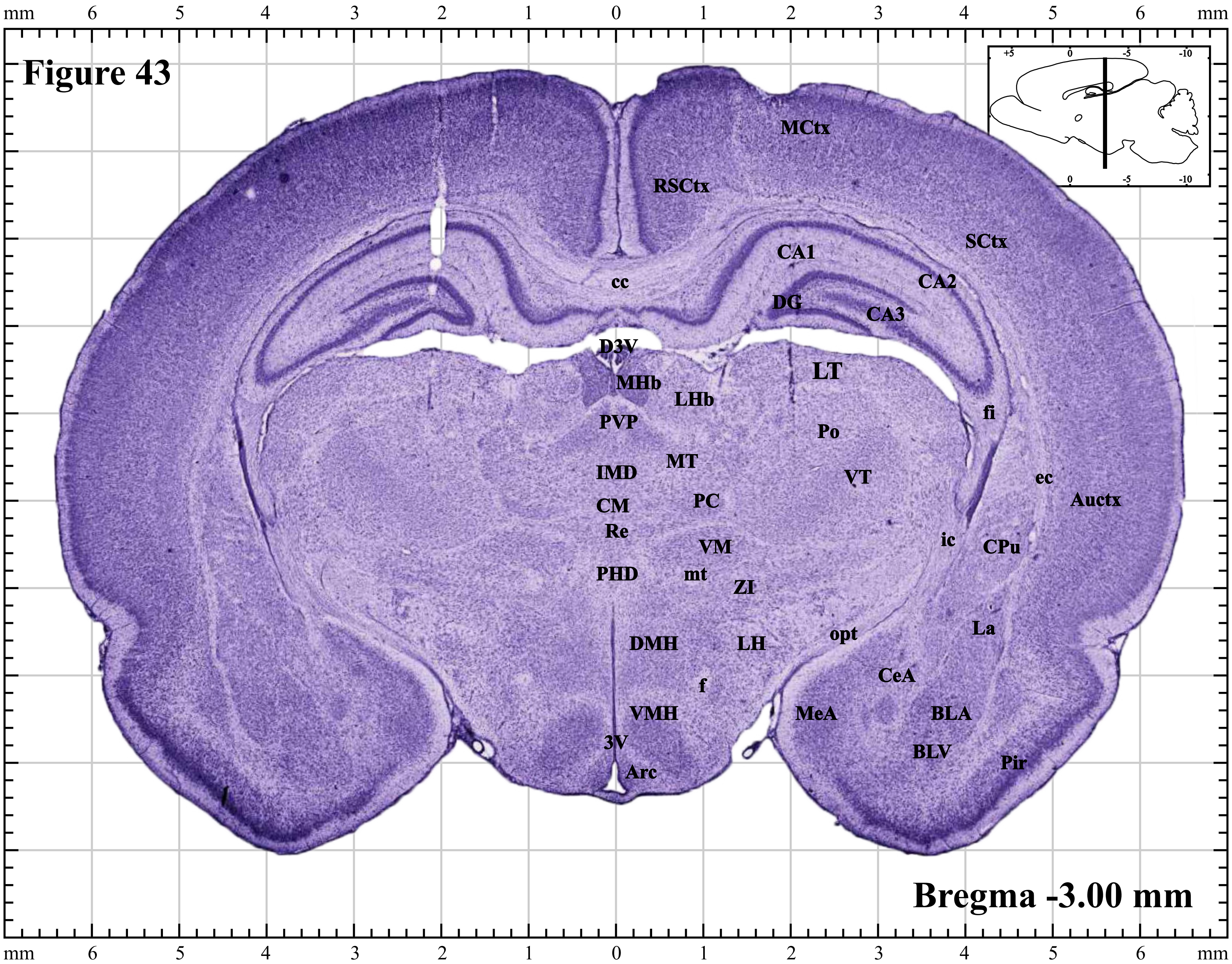

- **3V** medial longitudinal fasciculus
- **Arc** arcuate hypothalamic nucleus
- **Auctx** auditory cortex
- **BLA** basolateral amygdaloid nucleus, anterior part
- **BLV** basolateral amygdaloid nucleus, ventral part
- **CA1** field CA1 of the hippocampus
- **CA2** field CA2 of the hippocampus
- **CA3** field CA3 of the hippocampus
- **cc** corpus callosum
- **CeA** central amygdaloid nucleus
- **CM** central medial thalamic nucleus
- **CPu** caudate putamen
- **D3V** dorsal 3rd ventricle
- **DMH** dorsomedial hypothalamic nucleus
- **DG** dentate gyrus
- **ec** external capsule
- **f** fornix
- **fi** fimbria of the hippocampus
- **ic** internal capsule
- **IMD** intermediodorsal thalamic nucleus
- **La** lat amygdaloid nucleus
- **LHb** lateral habenular nucleus
- **LH** lateral hypothalamic area
- **LT** lateral thalamus
- **MeA** medial amygdaloid nucleus
- **mt** mammillothalamic tract
- **MHb** medial habenular nucleus
- **MT** medial thalamus
- **opt** optic tract
- **MCtx** motor cortex
- **PC** paracentral thalamic nucleus
- **Pir** piriform cortex
- **PVP** paraventricular thalamic nucleus, posterior part
- **Po** posterior thalamic nuclear group
- **PHD** posterior hypothalamic area, dorsal part
- **Re** reuniens thalamic nucleus
- **RSCtx** retrospenial cortex
- **SCtx** somatosensory cortex
- **VM** ventromedial thalamic nucleus
- **VMH** ventromedial hypothalamic nucleus
- **VT** ventral thalamus
- **ZI** zona incerta

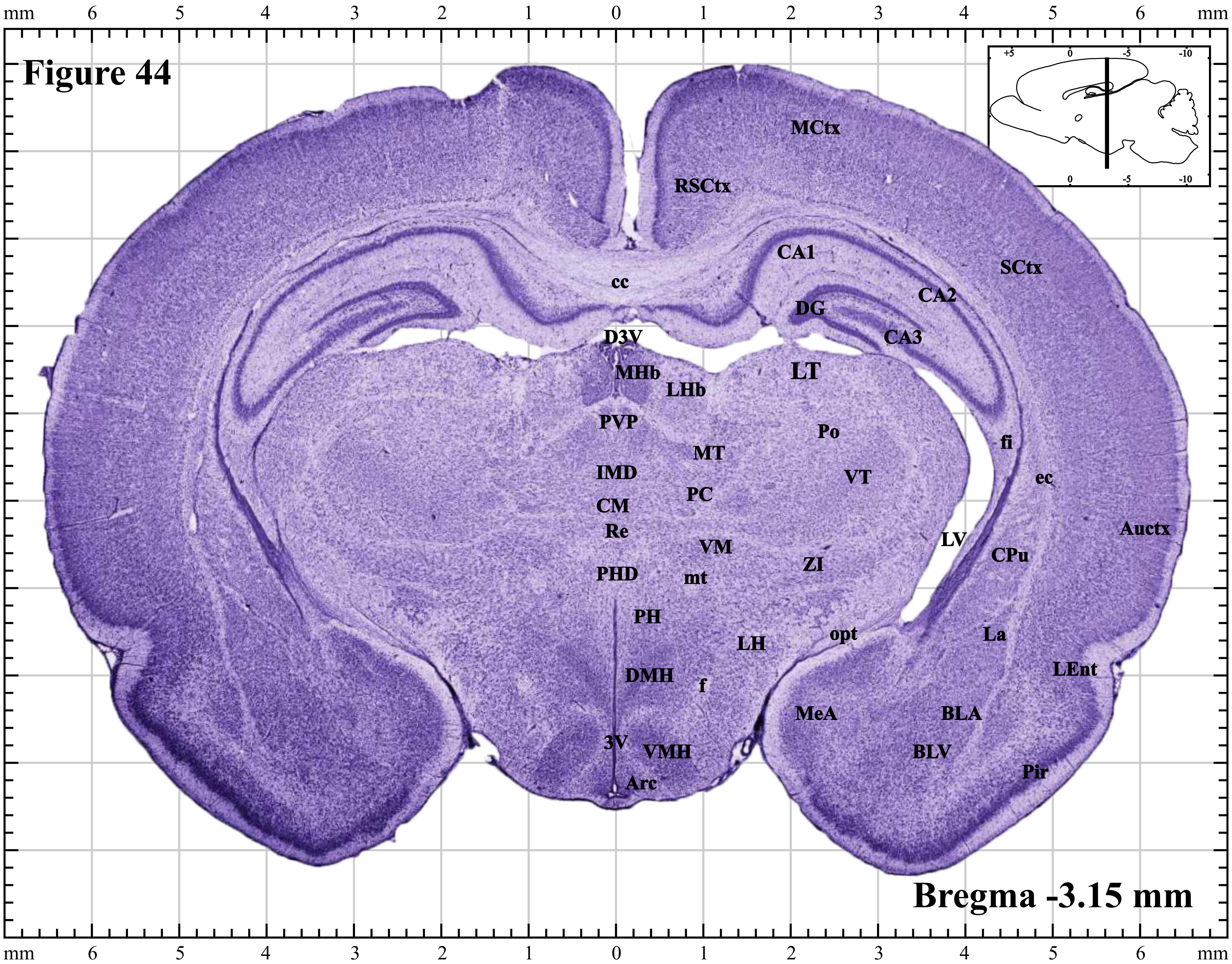

Figure 44

Bregma -3.15 mm

3V medial longitudinal fasciculus
Arc arcuate hypothalamic nucleus
Auctx auditory cortex
BLA basolateral amygdaloid nucleus, anterior part
BLV basolateral amygdaloid nucleus, ventral part
CA1 field CA1 of the hippocampus
CA2 field CA2 of the hippocampus
CA3 field CA3 of the hippocampus
cc corpus callosum
CeA central amygdaloid nucleus
CM central medial thalamic nucleus
CPu caudate putamen
D3V dorsal 3rd ventricle
DMH dorsomedial hypothalamic nucleus
DG dentate gyrus
ec external capsule
f fornix
fi fimbria of the hippocampus
ic internal capsule
IMD intermediodorsal thalamic nucleus
La lat amygdaloid nucleus
LEnt lateral entorhinal cortex
LHb lateral habenular nucleus
LH lateral hypothalamic area
LT lateral thalamus
LV lateral ventricle
mt mammillothalamic tract
MeA medial amygdaloid nucleus
MHb medial habenular nucleus
MT medial thalamus
opt optic tract
MCtx motor cortex
PC paracentral thalamic nucleus
Pir piriform cortex
PVP paraventricular thalamic nucleus, posterior part
Po posterior thalamic nuclear group
PH posterior hypothalamic nucleus
PHD posterior hypothalamic area, dorsal part
Re reuniens thalamic nucleus
RSCtx retrospenial cortex
SCtx somatosensory cortex
VM ventromedial thalamic nucleus
VMH ventromedial hypothalamic nucleus
VT ventral thalamus
ZI zona incerta

Figure 45

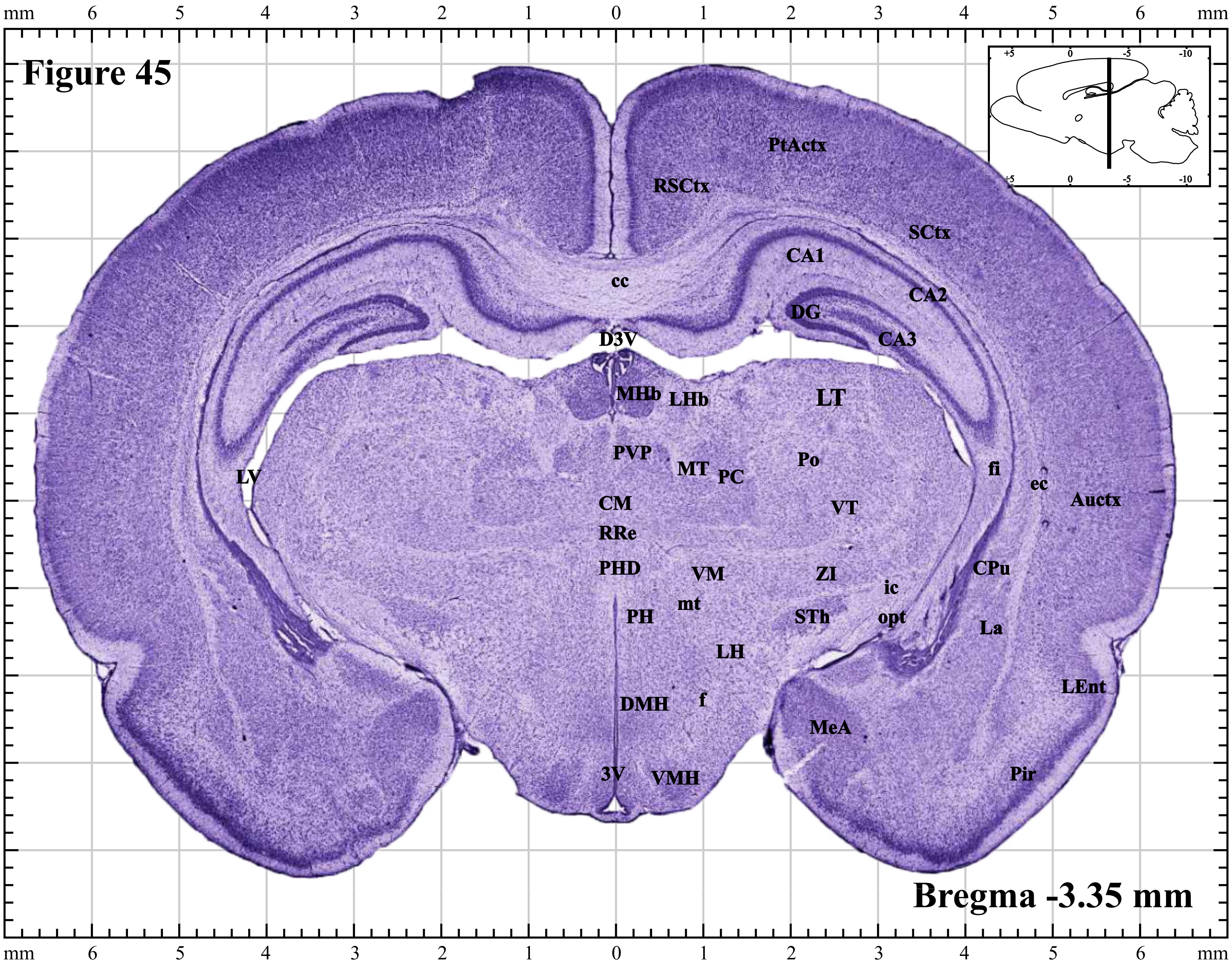

Bregma -3.35 mm

3V medial longitudinal fasciculus
Auctx auditory cortex
CA1 field CA1 of the hippocampus
CA2 field CA2 of the hippocampus
CA3 field CA3 of the hippocampus
cc corpus callosum
CeA central amygdaloid nucleus
CM central medial thalamic nucleus
CPu caudate putamen

D3V dorsal 3rd ventricle
DMH dorsomedial hypothalamic nucleus
DG dentate gyrus
ec external capsule
f fornix
fi fimbria of the hippocampus
ic internal capsule
IMD intermediodorsal thalamic nucleus

La lat amygdaloid nucleus
LHb lateral habenular nucleus
LH lateral hypothalamic area
LT lateral thalamus
LV lateral ventricle
LEnt lateral entorhinal cortex
mt mammillothalamic tract
MeA medial amygdaloid nucleus
MHb medial habenular nucleus
MT medial thalamus

opt optic tract
PC paracentral thalamic nucleus
Pir piriform cortex
PVP paraventricular thalamic nucleus, posterior part
Po posterior thalamic nuclear group
PH posterior hypothalamic nucleus
PHD posterior hypothalamic area, dorsal part

PtActx parietal association cortex
RRe retrouniens area
RSCtx retrospenial cortex
SCtx somatosensory cortex
STh subthalamic nucleus
VM ventromedial thalamic nucleus
VT ventral thalamus
VMH ventromedial hypothalamic nucleus
ZI zona incerta

Figure 46

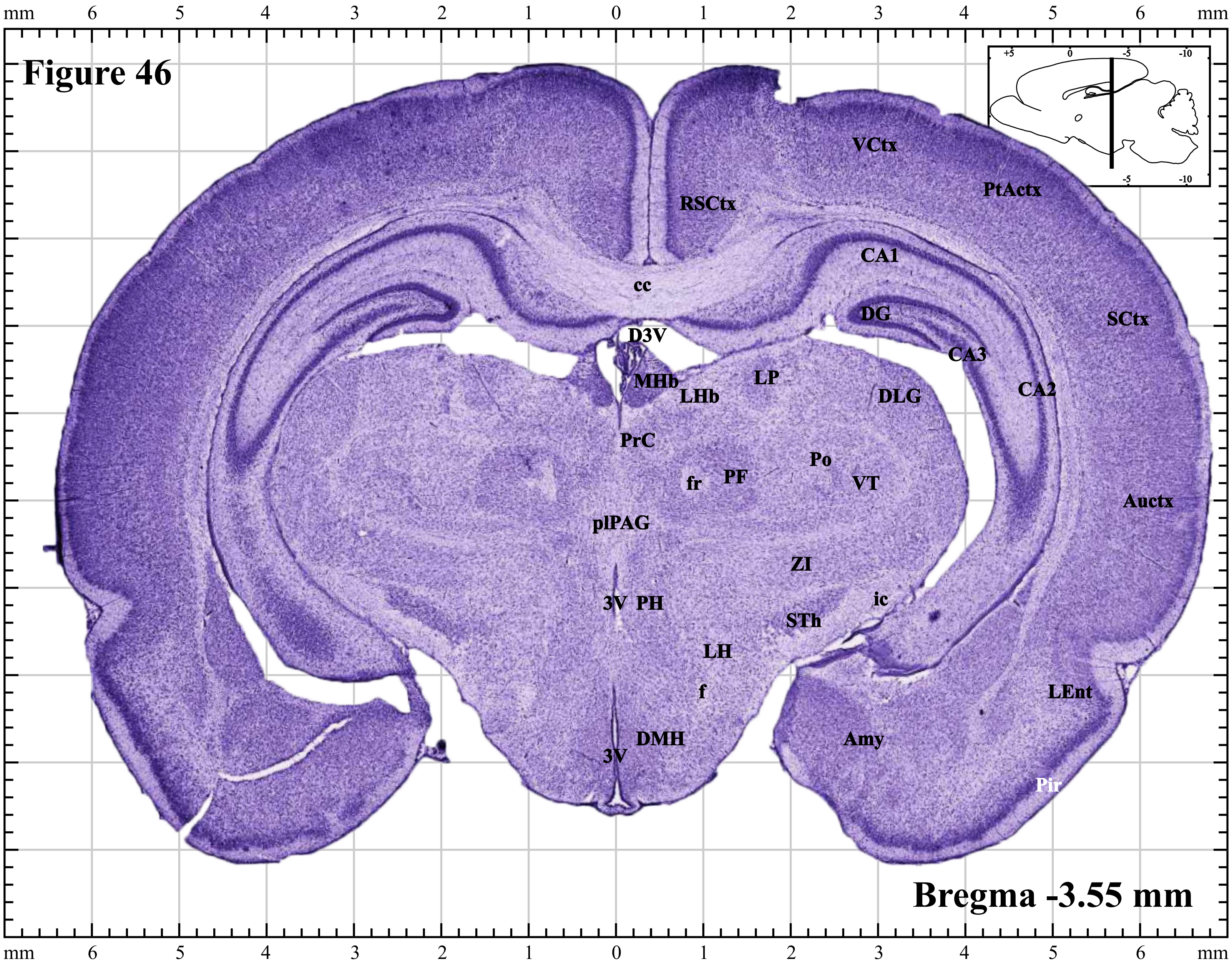

Bregma -3.55 mm

3V medial longitudinal fasciculus
Auctx auditory cortex
Amy amygdaloid nuclei
CA1 field CA1 of the hippocampus
CA2 field CA2 of the hippocampus
CA3 field CA3 of the hippocampus
cc corpus callosum
DMH dorsomedial hypothalamic nucleus
DLG dorsal lateral geniculate nucleus
D3V dorsal 3rd ventricle
DG dentate gyrus
f fornix
fr fasciculus retroflexus
ic internal capsule
LHb lateral habenular nucleus
LH lateral hypothalamic area
LP lateral posterior thalamic nucleus
LEnt lateral entorhinal cortex
mt mammillothalamic tract
MHb medial habenular nucleus
PrC precommissural nucleus
Pir piriform cortex
Po posterior thalamic nuclear group
plPAG pleomorphic part of periaqueductai gray
PF parafascicular thalamic nucleus
PH posterior hypothalamic nucleus
PtActx parietal association cortex
RSCtx retrospenial cortex
SCtx somatosensory cortex
STh subthalamic nucleus
VCtx visual cortex
VT ventral thalamus
ZI zona incerta

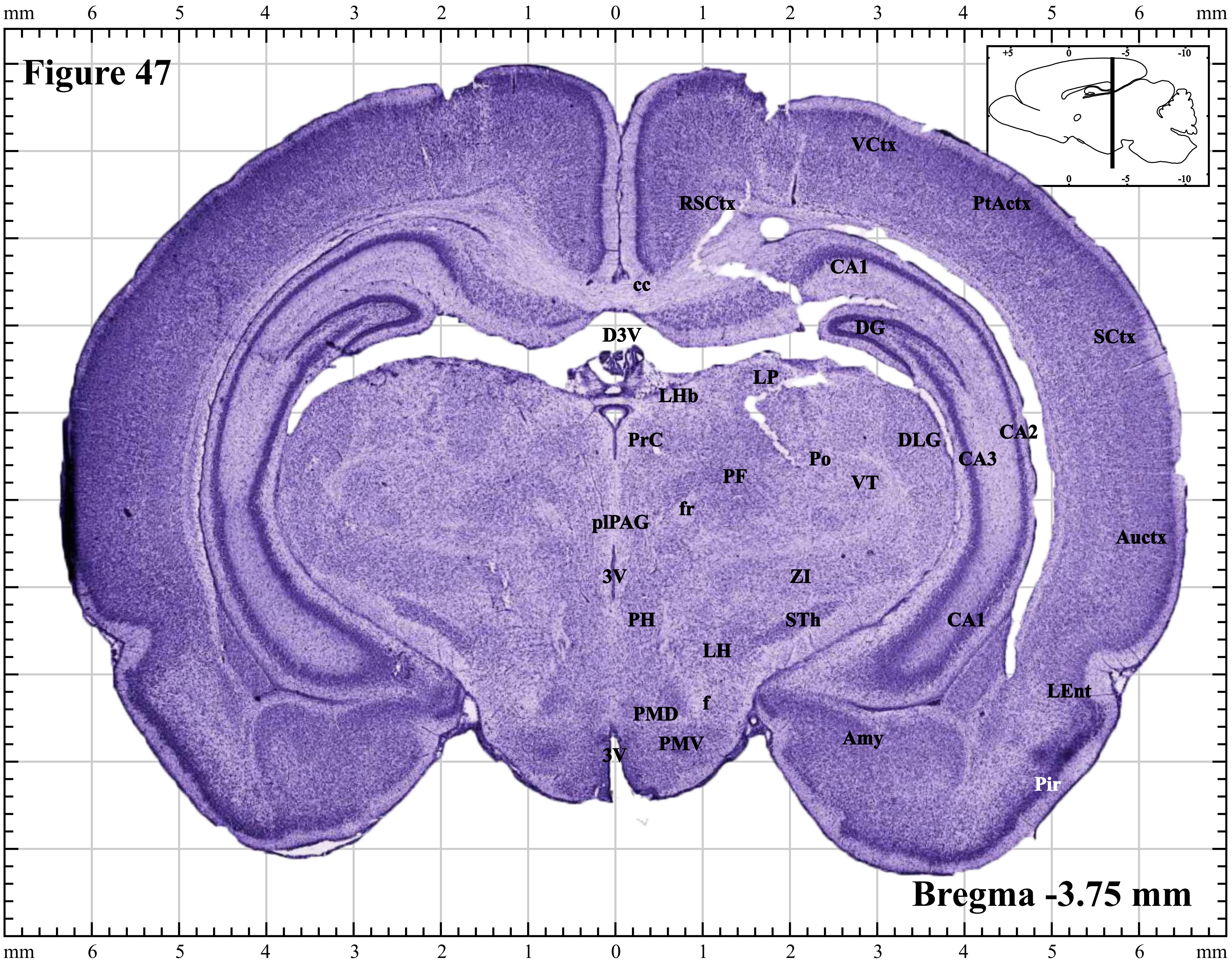

3V medial longitudinal fasciculus
Auctx auditory cortex
Amy amygdaloid nuclei
CA1 field CA1 of the hippocampus
CA2 field CA2 of the hippocampus
CA3 field CA3 of the hippocampus
cc corpus callosum
DLG dorsal lateral geniculate nucleus
D3V dorsal 3rd ventricle
DG dentate gyrus
f fornix
fr fasciculus retroflexus
ic internal capsule
LHb lateral habenular nucleus
LH lateral hypothalamic area
LP lateral posterior thalamic nucleus
LEnt lateral entorhinal cortex
mt mammillothalamic tract
PMV premammillary nucleus, ventral part
PMD premammillary nucleus, dorsal part
PrC precommissural nucleus
Pir piriform cortex
Po posterior thalamic nuclear group
plPAG pleomorphic part of periaqueductai gray
PF parafascicular thalamic nucleus
PH posterior hypothalamic nucleus
PtActx parietal association cortex
RSCtx retrospenial cortex
SCtx somatosensory cortex
STh subthalamic nucleus
VCtx visual cortex
VT ventral thalamus
ZI zona incerta

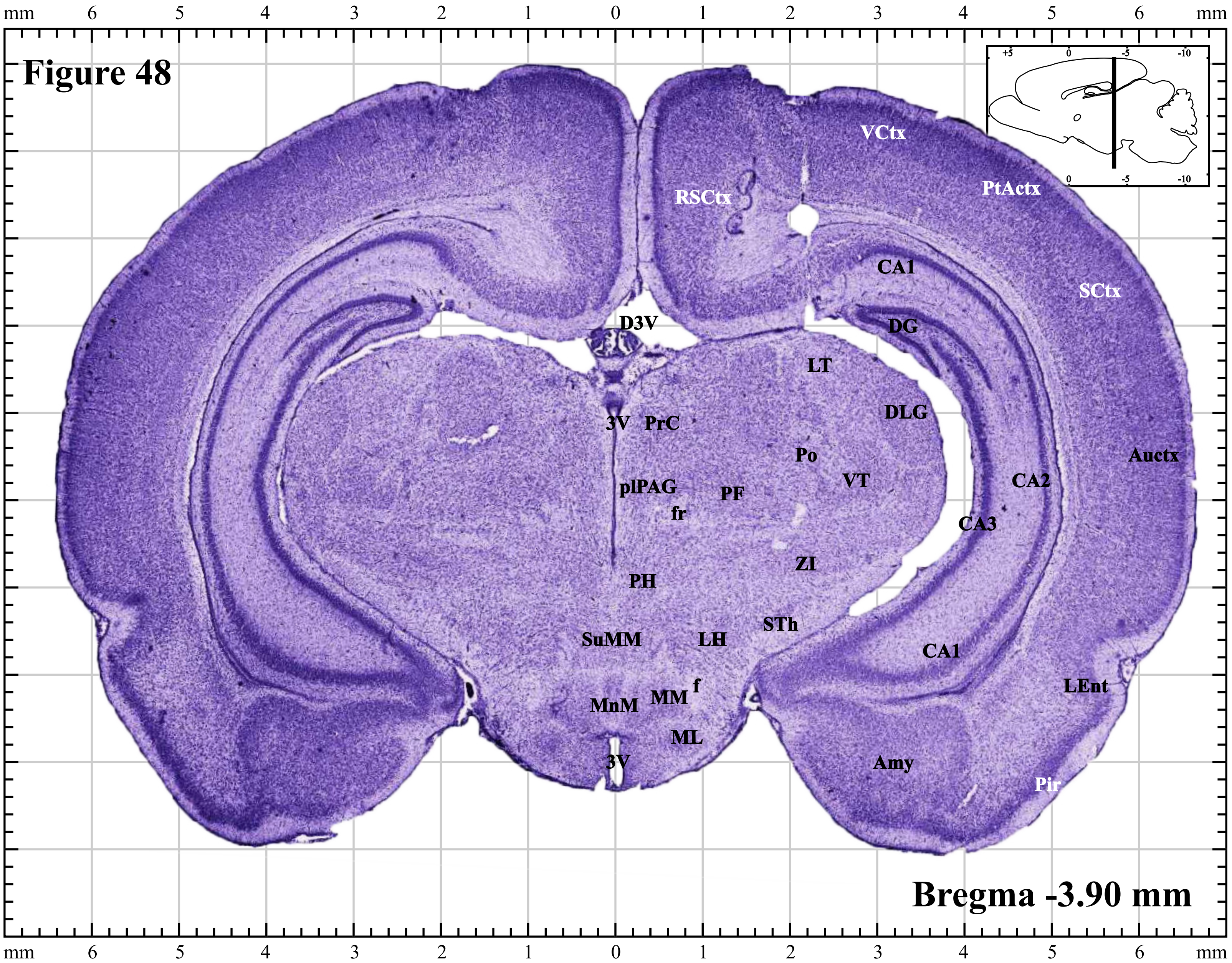

3V medial longitudinal fasciculus
Auctx auditory cortex
Amy amygdaloid nuclei
CA1 field CA1 of the hippocampus
CA2 field CA2 of the hippocampus
CA3 field CA3 of the hippocampus
D3V dorsal 3rd ventricle
DG dentate gyrus
DLG dorsal lateral geniculate nucleus
f fornix
fr fasciculus retroflexus
LH lateral hypothalamic area
LT lateral thalamus
LEnt lateral entorhinal cortex
ML medial mammillary nucleus, lateral part
MnM medial mammillary nucleus, median part
MM medial mammillary nucleus, medial part
PF parafascicular thalamic nucleus
PrC precommissural nucleus
Pir piriform cortex
Po posterior thalamic nuclear group
PH posterior hypothalamic nucleus
plPAG pleomorphic part of periaqueductai gray
PtActx parietal association cortex
RSCtx retrospenial cortex
SCtx somatosensory cortex
SuMM supramammillary nucleus, medial part
VCtx visual cortex
VT ventral thalamus
ZI zona incerta

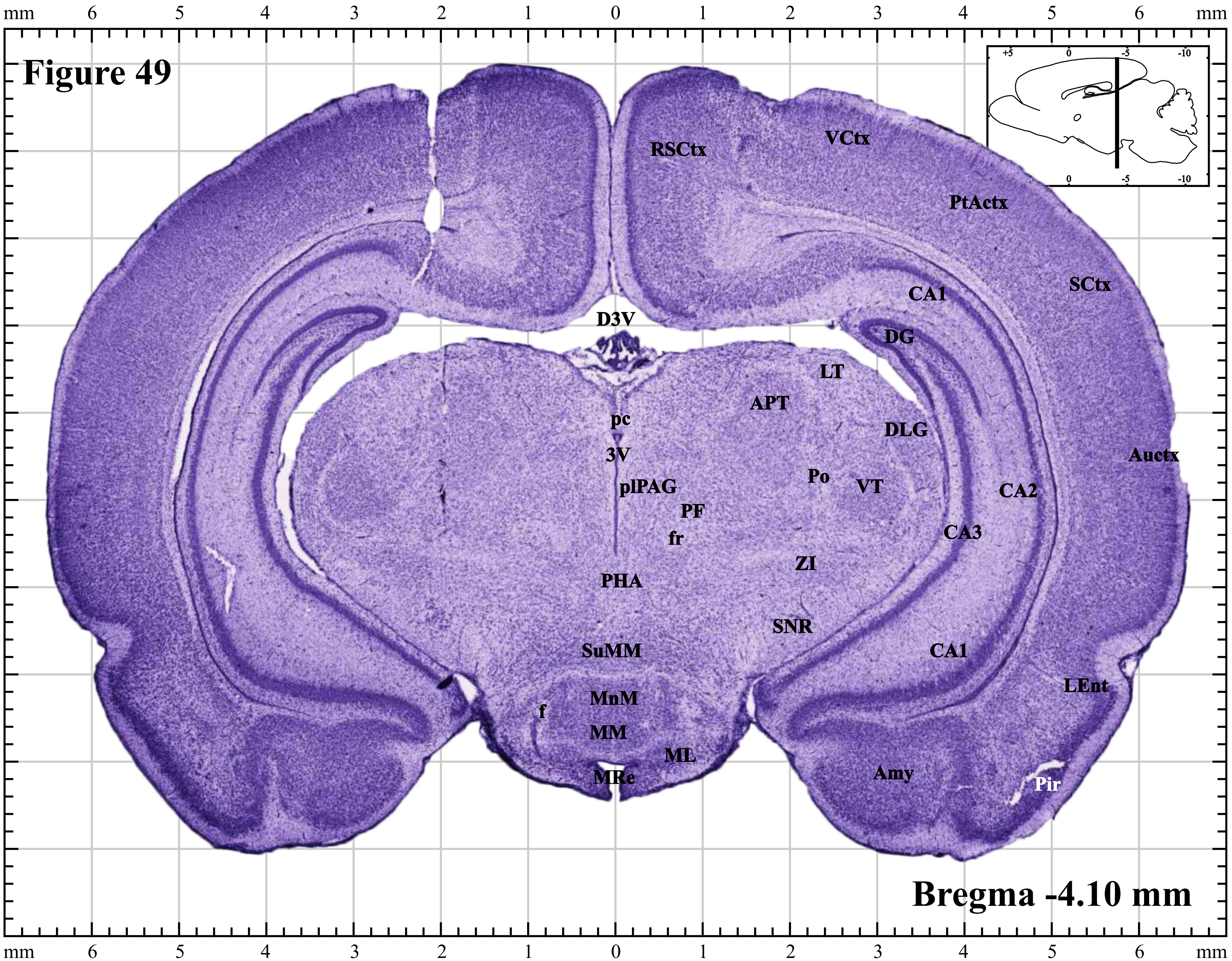

3V medial longitudinal fasciculus
Auctx auditory cortex
Amy amygdaloid nuclei
APT anterior pretectal nucleus
CA1 field CA1 of the hippocampus
CA2 field CA2 of the hippocampus
CA3 field CA3 of the hippocampus
D3V dorsal 3rd ventricle
DG dentate gyrus
DLG dorsal lateral geniculate nucleus
f fornix
fr fasciculus retroflexus
LT lateral thalamus
LEnt lateral entorhinal cortex
ML medial mammillary nucleus, lateral part
MnM medial mammillary nucleus, median part
MM medial mammillary nucleus, medial part
MRe mammillary recess of the 3rd ventricle
pc posterior commissure
PF parafascicular thalamic nucleus
Po posterior thalamic nuclear group
PHA posterior hypothalamic area
plPAG pleomorphic part of periaqueductai gray
Pir piriform cortex
PtActx parietal association cortex
RSCtx retrospenial cortex
SCtx somatosensory cortex
SNR substantia nigra, reticular part
SuMM supramammillary nucleus, medial part
VCtx visual cortex
VT ventral thalamus
ZI zona incerta

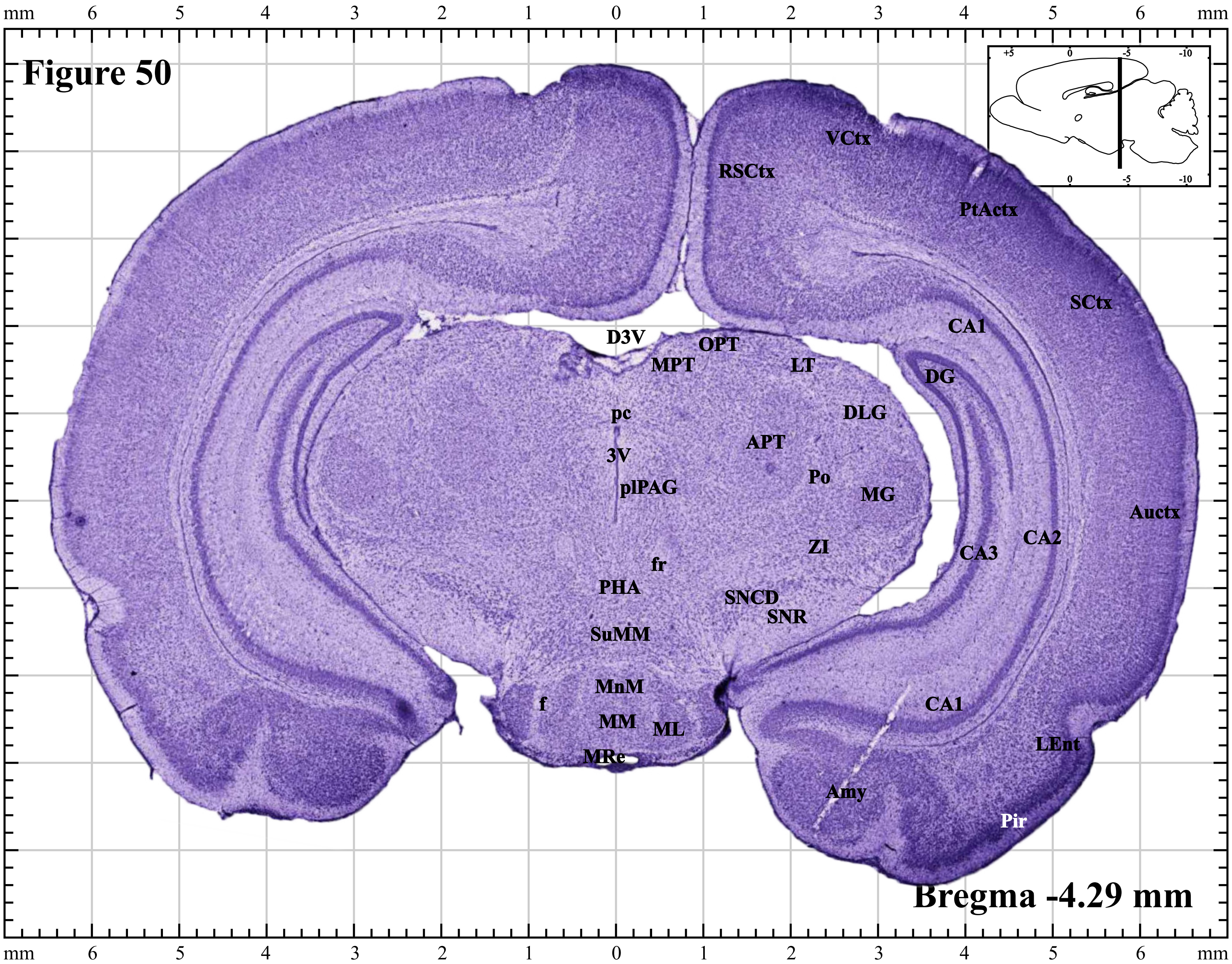

3V medial longitudinal fasciculus
Auctx auditory cortex
Amy amygdaloid nuclei
APT anterior pretectal nucleus
CA1 field CA1 of the hippocampus
CA2 field CA2 of the hippocampus
CA3 field CA3 of the hippocampus
D3V dorsal 3rd ventricle
DG dentate gyrus
DLG dorsal lateral geniculate nucleus
f fornix
fr fasciculus retroflexus
LT lateral thalamus
LEnt lateral entorhinal cortex
ML medial mammillary nucleus, lateral part
MnM medial mammillary nucleus, median part
MM medial mammillary nucleus, medial part
MRe mammillary recess of the 3rd ventricle
MG medial geniculate nucleus
MPT medial pretectal nucleus
OPT olivary pretectal nucleus
Pir piriform cortex
Po posterior thalamic nuclear group
PHA posterior hypothalamic area
plPAG pleomorphic part of periaqueductai gray
PtActx parietal association cortex
RSCtx retrospenial cortex
SCtx somatosensory cortex
SNR substantia nigra, reticular part
SuMM supramammillary nucleus, medial part
SNCD substantia nigra, compact part, dorsal tier
ZI zona incerta
VCtx visual cortex

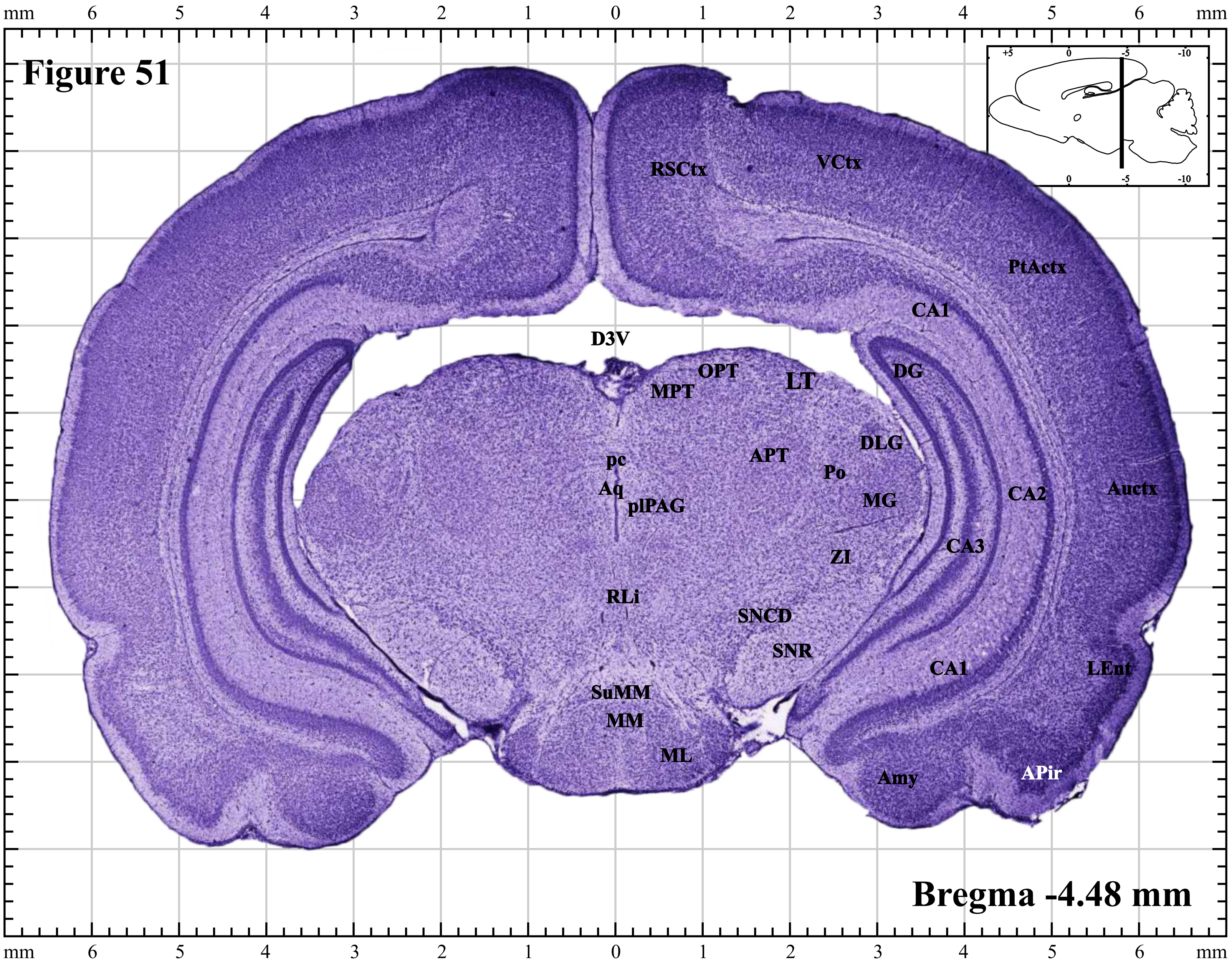

Aq aqueduct
Auctx auditory cortex
Amy amygdaloid nuclei
APT anterior pretectal nucleus
APir amygdalopiriform transition area
CA1 field CA1 of the hippocampus
CA2 field CA2 of the hippocampus
CA3 field CA3 of the hippocampus
D3V dorsal 3rd ventricle
DLG dorsal lateral geniculate nucleus
DG dentate gyrus
LT lateral thalamus
LEnt lateral entorhinal cortex
ML medial mammillary nucleus, lateral part
MM medial mammillary nucleus, medial part
MG medial geniculate nucleus
MPT medial pretectal nucleus
OPT olivary pretectal nucleus
pc posterior commissure
plPAG pleomorphic part of periaqueductai gray
PtActx parietal association cortex
Po posterior thalamic nuclear group
RSCtx retrospenial cortex
RLi rostral linear nucleus of the raphe
SuMM supramammillary nucleus, medial part
SNR substantia nigra, reticular part
SNCD substantia nigra, compact part, dorsal tier
VCtx visual cortex
ZI zona incerta

Figure 52

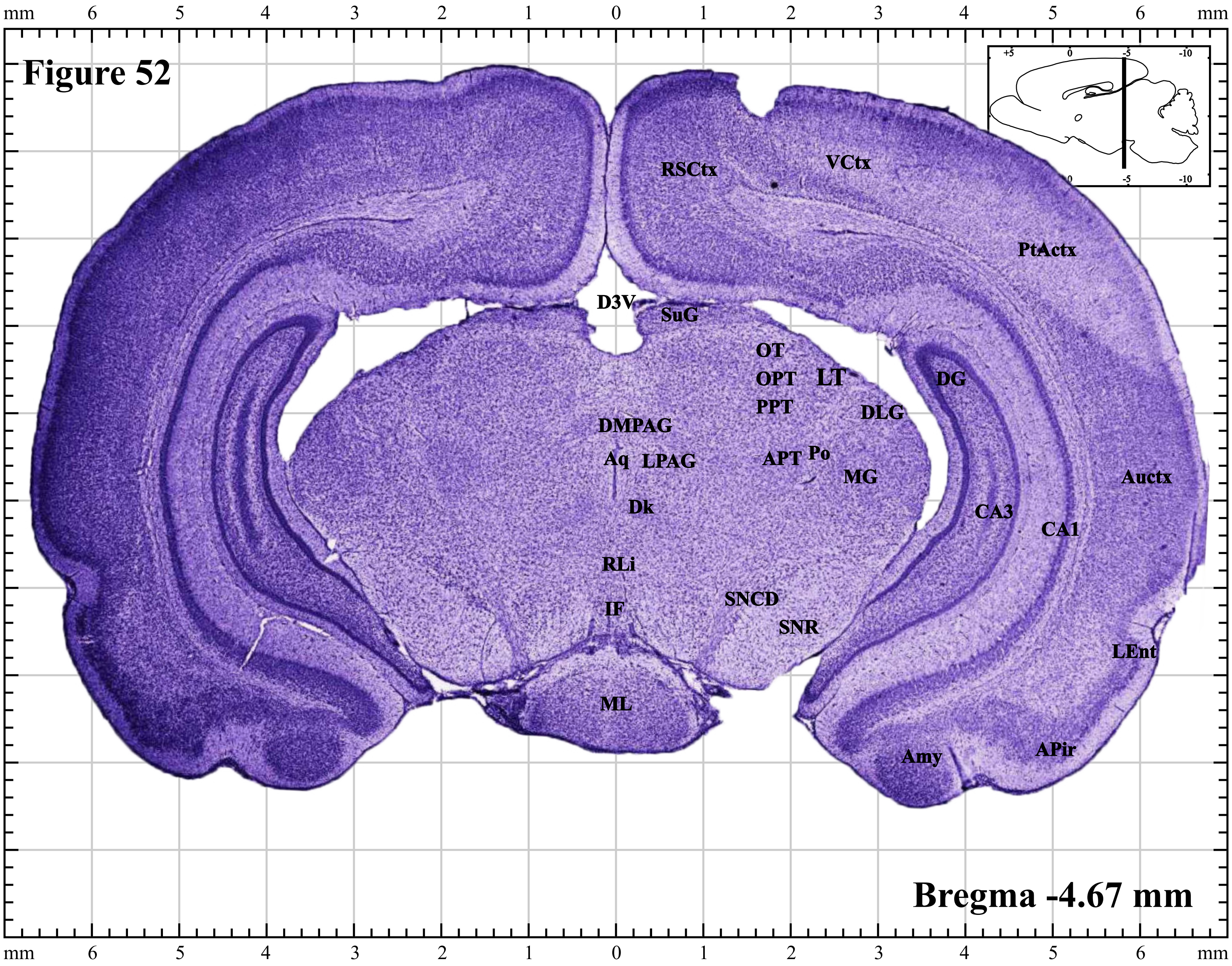

Aq aqueduct
Auctx auditory cortex
Amy amygdaloid nuclei
APT anterior pretectal nucleus
APir amygdalopiriform transition area
CA1 field CA1 of the hippocampus
CA3 field CA3 of the hippocampus
D3V dorsal 3rd ventricle
DG dentate gyrus
Dk nucleus of Darkschewitsch
DLG dorsal lateral geniculate nucleus
DMPAG dorsomedial periaqueductal gray
IF interfascicular nucleus
LT lateral thalamus
LEnt lateral entorhinal cortex
LPAG lateral periaqueductal gray
ML medial mammillary nucleus, lateral part
MG medial geniculate nucleus
OT nucleus of the optic
OPT olivary pretectal nucleus
PtActx parietal association cortex
Po posterior thalamic nuclear group
PPT posterior pretectal nucleus
RSCtx retrospenial cortex
SNR substantia nigra, reticular part
SNCD substantia nigra, compact part, dorsal tier
SuG superficial gray layer of the superior colliculus
VCtx visual cortex

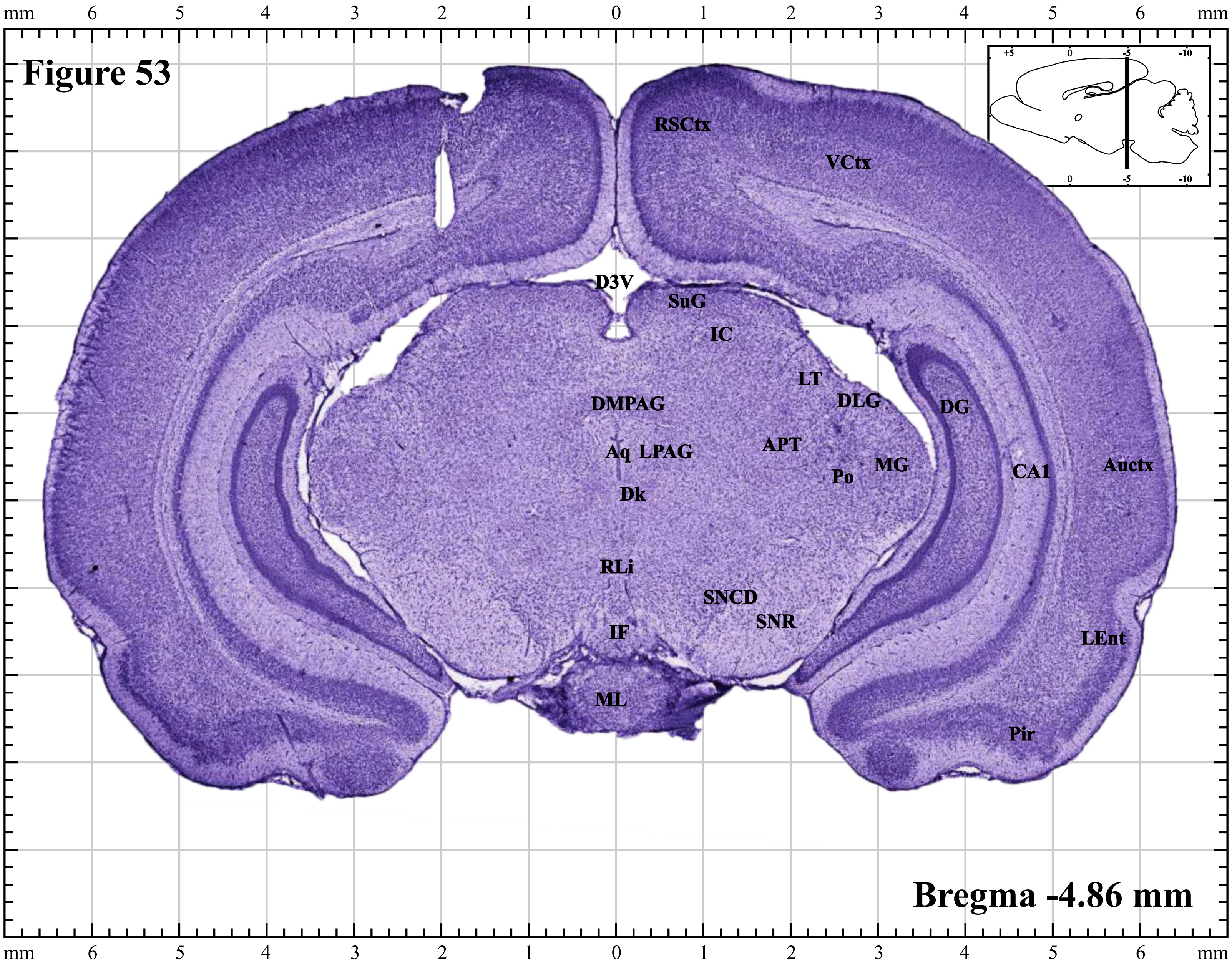

Aq aqueduct
Auctx auditory cortex
APT anterior pretectal nucleus
APir amygdalopiriform transition area
CA1 field CA1 of the hippocampus
CA3 field CA3 of the hippocampus
D3V dorsal 3rd ventricle
DG dentate gyrus
DLG dorsal lateral geniculate nucleus
Dk nucleus of Darkschewitsch
DMPAG dorsomedial periaqueductal gray
IF interfascicular nucleus
IC inferior colliculus
LPAG lateral periaqueductal gray
LT lateral thalamus
LEnt lateral entorhinal cortex
ML medial mammillary nucleus, lateral part
MG medial geniculate nucleus
OT nucleus of the optic
pc posterior commissure
Po posterior thalamic nuclear group
RSCtx retrospenial cortex
RLi rostral linear nucleus of the raphe
SuG superficial gray layer of the superior colliculus
SNR substantia nigra, reticular part
SNCD substantia nigra, compact part, dorsal tier
VCtx visual cortex

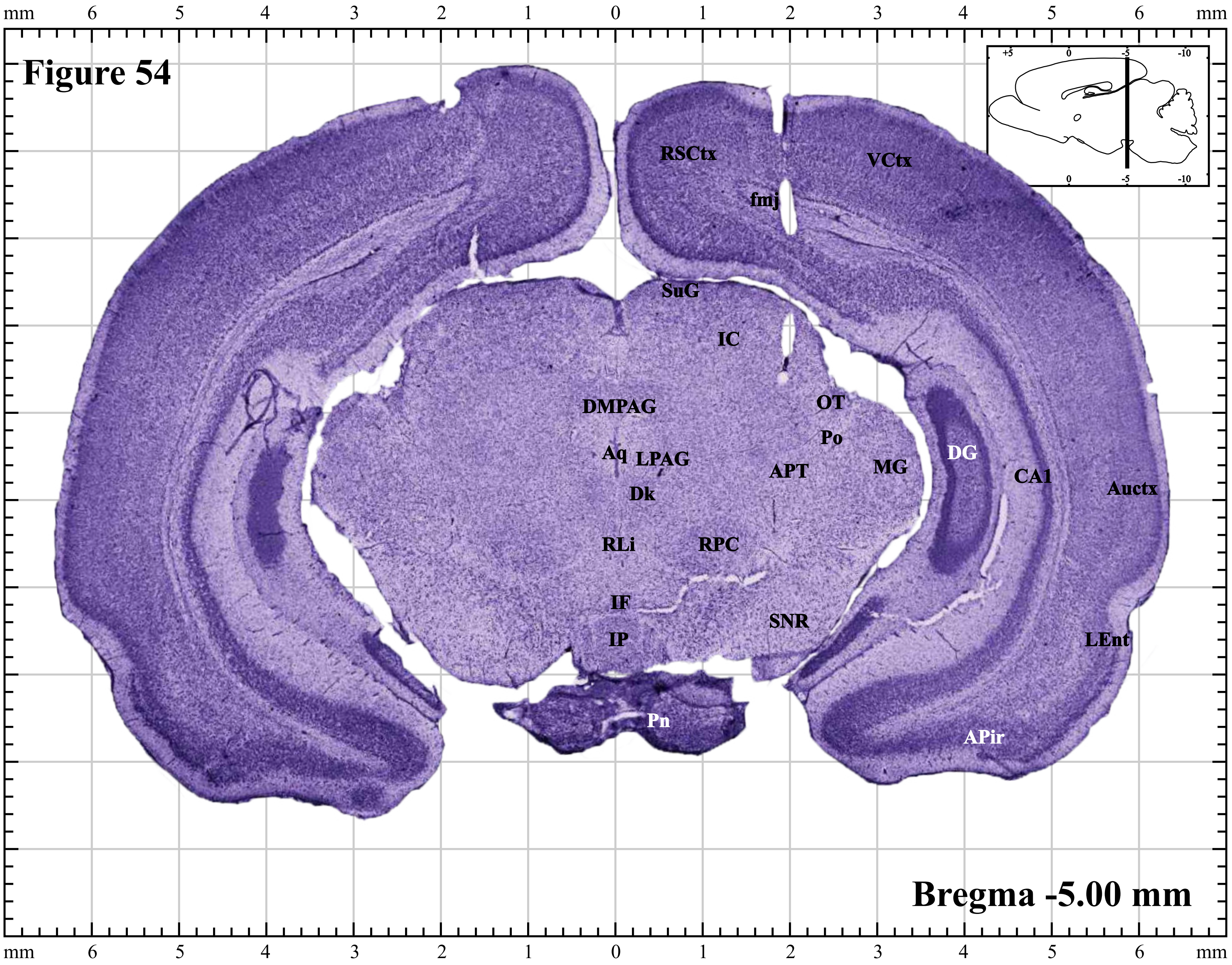

Aq aqueduct
Auctx auditory cortex
APT anterior pretectal nucleus
APir amygdalopiriform transition area
CA1 field CA1 of the hippocampus
DMPAG dorsomedial periaqueductal gray
DG dentate gyrus
Dk nucleus of Darkschewitsch
fmj forceps major of the corpus callosum
IC inferior colliculus
IF interfascicular nucleus
IP interpeduncular nucleus
LEnt lateral entorhinal cortex
LPAG lateral periaqueductal gray
MG medial geniculate nucleus
OT nucleus of the optic
Pn pontine nuclei
Po posterior thalamic nuclear group
RSCtx retrospenial cortex
RLi rostral linear nucleus of the raphe
RPC red nucleus, parvicellular part
SuG superficial gray layer of the superior colliculus
SNR substantia nigra, reticular part
VCtx visual cortex

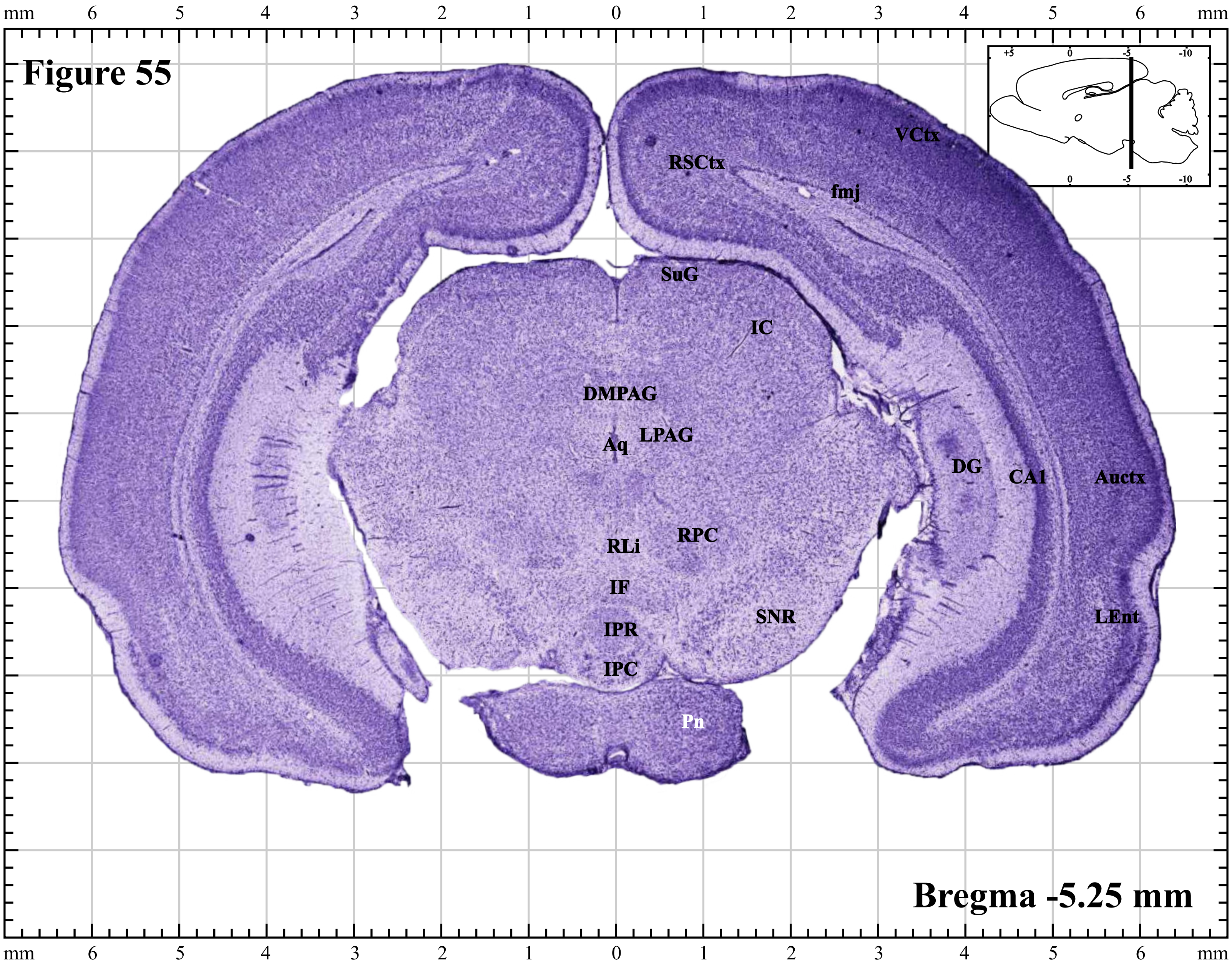

Aq aqueduct
Auctx auditory cortex
CA1 field CA1 of the hippocampus
DMPAG dorsomedial periaqueductal gray
DG dentate gyrus
fmj forceps major of the corpus callosum
IC inferior colliculus
IF interfascicular nucleus
IPC interpeduncular nucleus, caudal subnucleus
IPR interpeduncular nucleus, rostral subnucleus
LEnt lateral entorhinal cortex
LPAG lateral periaqueductal gray
Pn pontine nuclei
RSCtx retrospenial cortex
RLi rostral linear nucleus of the raphe
RPC red nucleus, parvicellular part
SuG superficial gray layer of the superior colliculus
SNR substantia nigra, reticular part
VCtx visual cortex

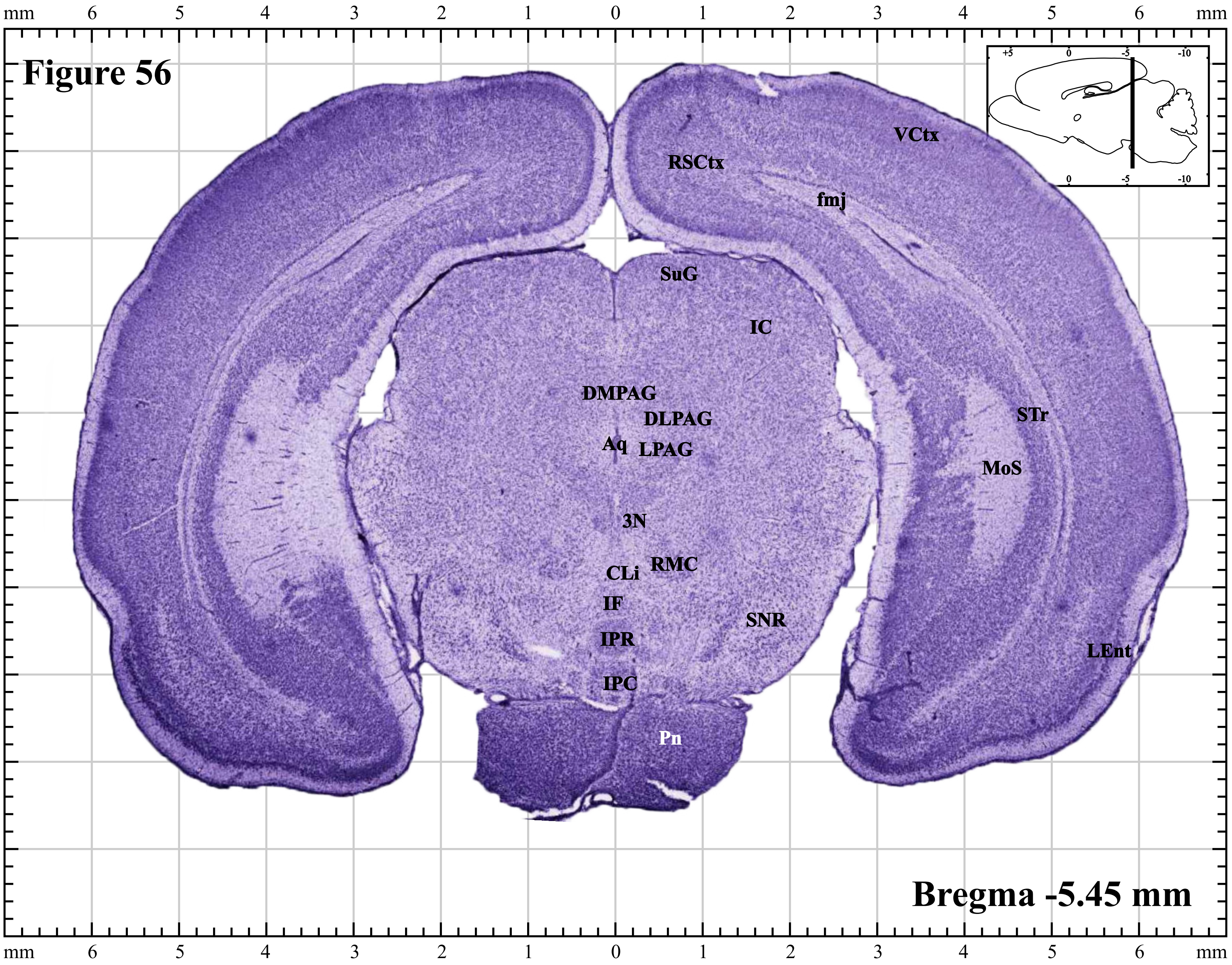

3N oculomotor nucleus
Aq aqueduct
CLi caudal linear nucleus of the raphe
DMPAG dorsomedial periaqueductal gray
DLPAG dorsolateral periaqueductal gray
fmj forceps major of the corpus callosum
IC inferior colliculus
IF interfascicular nucleus
IPC interpeduncular nucleus, caudal subnucleus
IPR interpeduncular nucleus, rostral subnucleus
LPAG lateral periaqueductal gray
LEnt lateral entorhinal cortex
MoS molecular layer of the subiculum
Pn pontine nuclei
RSCtx retrospenial cortex
RMC red nucleus, magnocellular part
STr subiculum, transition area
SuG superficial gray layer of the superior colliculus
SNR substantia nigra, reticular part
VCtx visual cortex

Figure 57

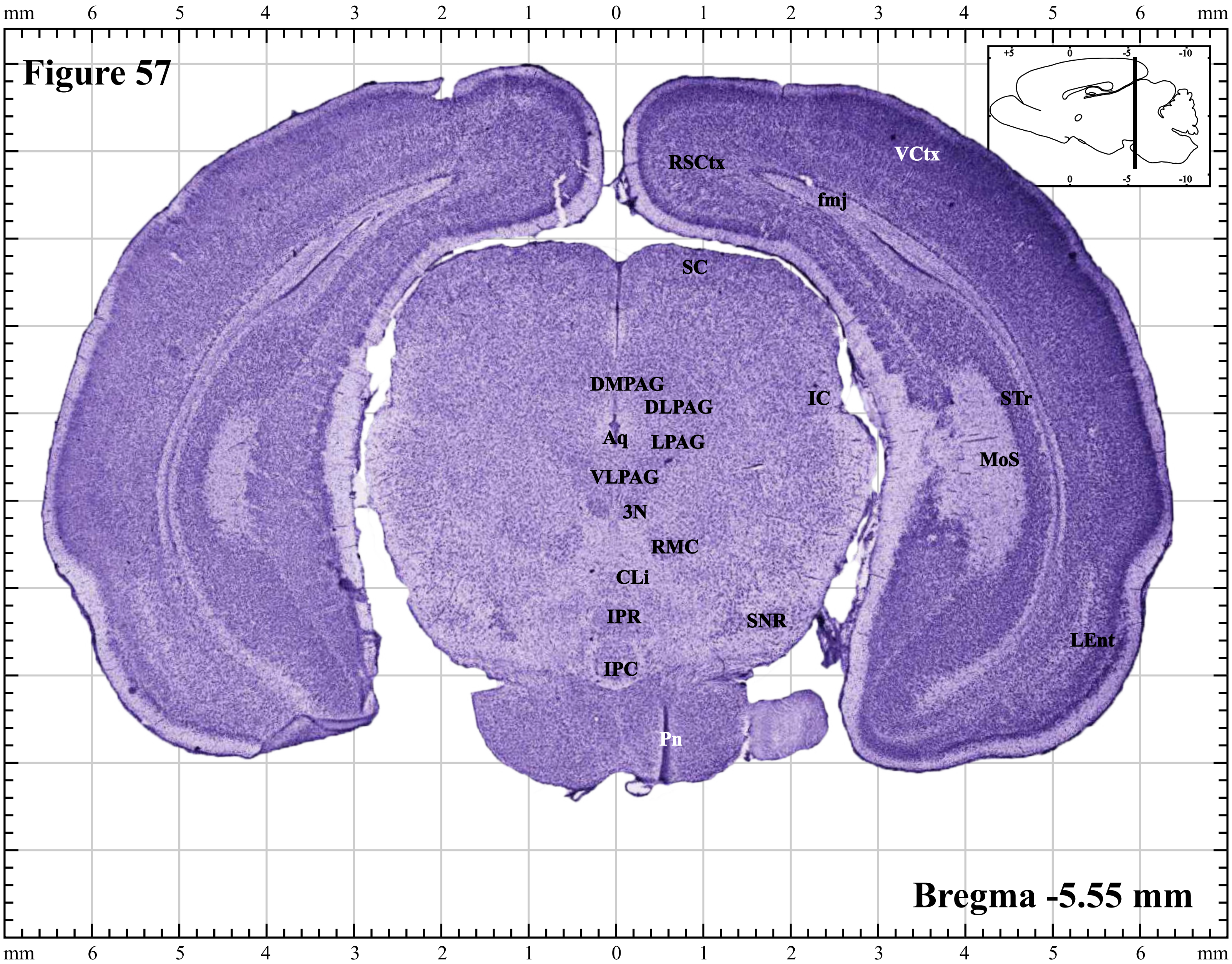

Bregma -5.55 mm

3N oculomotor nucleus
Aq aqueduct
CLi caudal linear nucleus of the raphe
DMPAG dorsomedial periaqueductal gray
DLPAG dorsolateral periaqueductal gray
fmj forceps major of the corpus callosum
IC inferior colliculus
IF interfascicular nucleus
IPC interpeduncular nucleus, caudal subnucleus
IPR interpeduncular nucleus, rostral subnucleus
LEnt lateral entorhinal cortex
LPAG lateral periaqueductal gray
MoS molecular layer of the subiculum
Pn pontine nuclei
RSCtx retrospenial cortex
RMC red nucleus, magnocellular part
STr subiculum, transition area
SuG superficial gray layer of the superior colliculus
SNR substantia nigra, reticular part
VCtx visual cortex
VLPAG ventrolateral periaqueductal gray

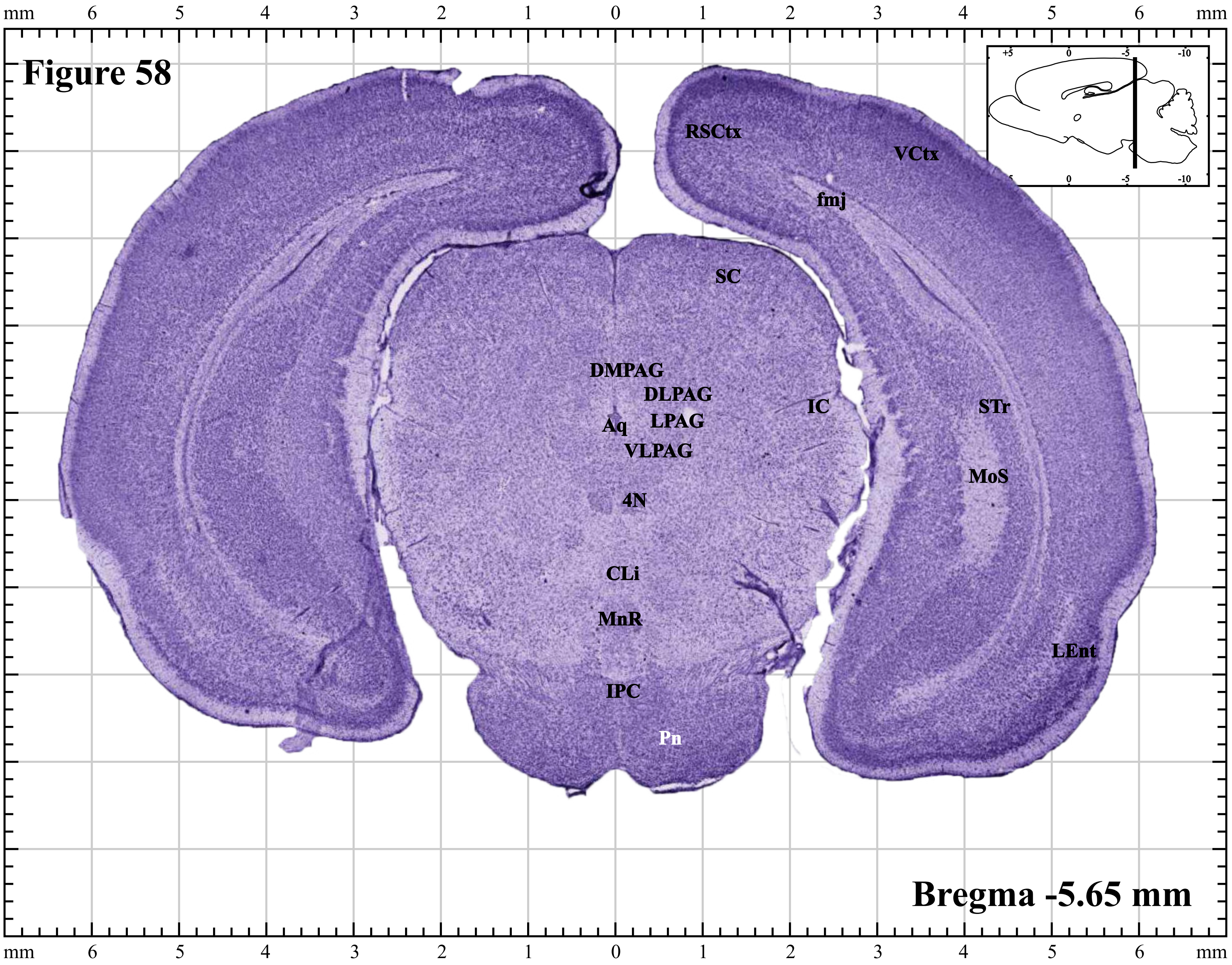

4N trochlear nucleus
Aq aqueduct
CLi caudal linear nucleus of the raphe
DMPAG dorsomedial periaqueductal gray
DLPAG dorsolateral periaqueductal gray
fmj forceps major of the corpus callosum
IC inferior colliculus
IF interfascicular nucleus
IPC interpeduncular nucleus, caudal subnucleus
LEnt lateral entorhinal cortex
LPAG lateral periaqueductal gray
MnR median raphe nucleus
MoS molecular layer of the subiculum
Pn pontine nuclei
RSCtx retrospenial cortex
STr subiculum, transition area
SC superior colliculus
VCtx visual cortex
VLPAG ventrolateral periaqueductal gray

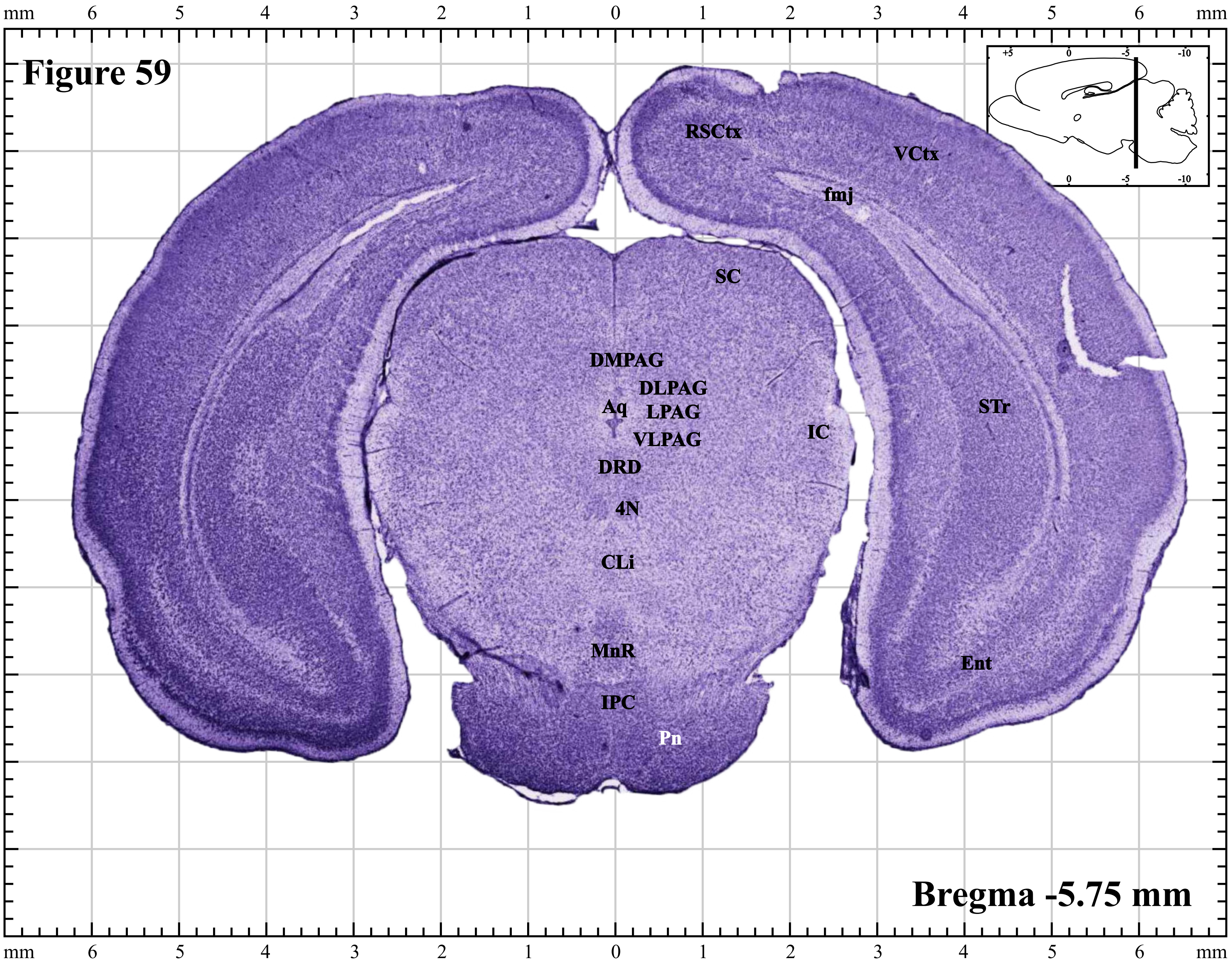

- **4N** trochlear nucleus
- **Aq** aqueduct
- **CLi** caudal linear nucleus of the raphe
- **DMPAG** dorsomedial periaqueductal gray
- **DLPAG** dorsolateral periaqueductal gray
- **DRD** dorsomedial hypothalamic nucleus, dorsal part
- **Ent** entorhinal cortex
- **fmj** forceps major of the corpus callosum
- **IC** inferior colliculus
- **IF** interfascicular nucleus
- **IPC** interpeduncular nucleus, caudal subnucleus
- **LPAG** lateral periaqueductal gray
- **MnR** median raphe nucleus
- **Pn** pontine nuclei
- **RSCtx** retrospenial cortex
- **STr** subiculum, transition area
- **SC** superior colliculus
- **VCtx** visual cortex
- **VLPAG** ventrolateral periaqueductal gray

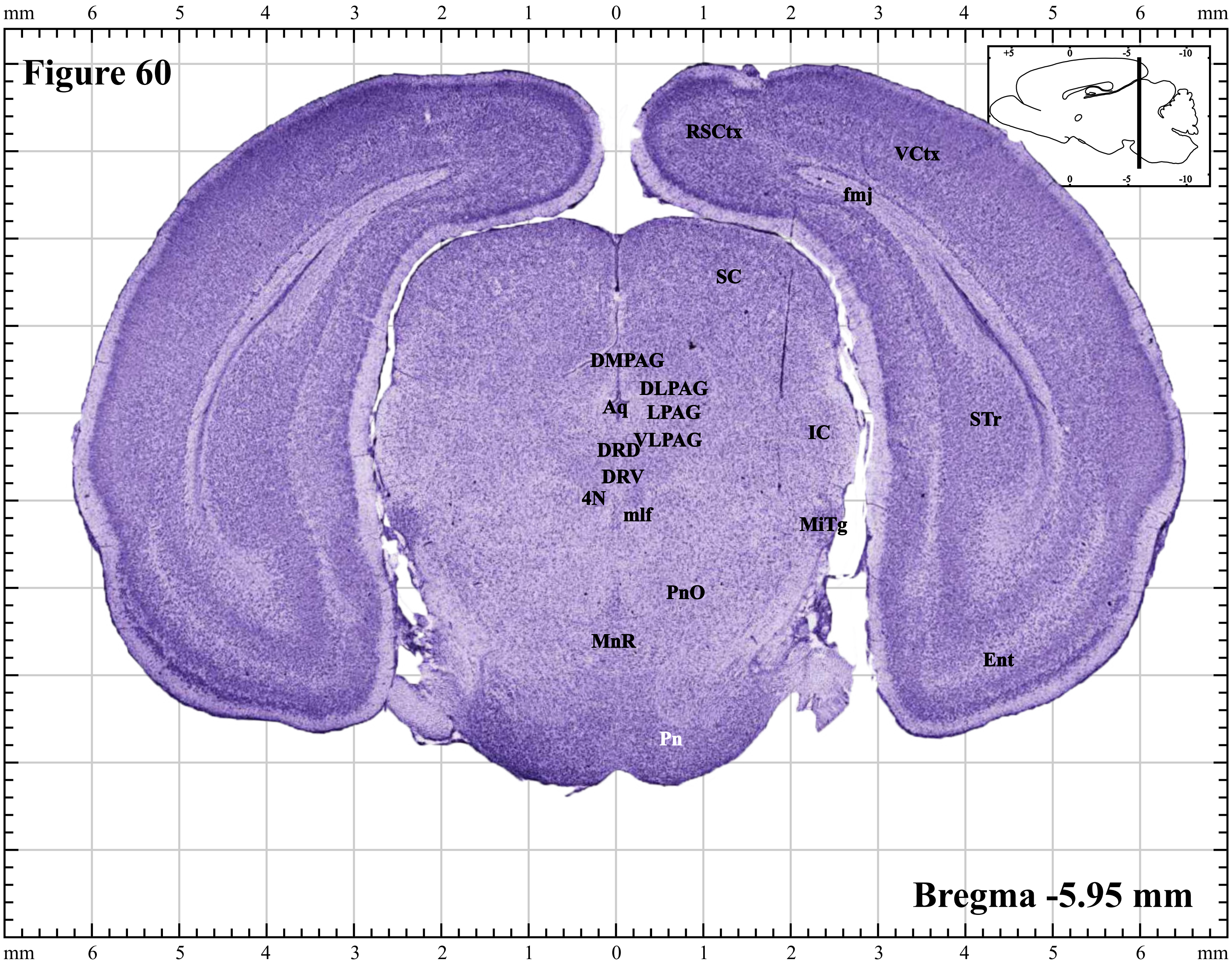

4N trochlear nucleus
Aq aqueduct
DMPAG dorsomedial periaqueductal gray
DLPAG dorsolateral periaqueductal gray
DRD dorsomedial hypothalamic nucleus, dorsal part
DRV dorsomedial hypothalamic nucleus, ventral part
Ent entorhinal cortex
fmj forceps major of the corpus callosum
IC inferior colliculus
LPAG lateral periaqueductal gray
mlf medial longitudinal fasciculus
MnR median raphe nucleus
MiTg microcellular tegmental nucleus
PnO pontine reticular nucleus, oral part
Pn pontine nuclei
RSCtx retrospenial cortex
STr subiculum, transition area
SC superior colliculus
VCtx visual cortex
VLPAG ventrolateral periaqueductal gray

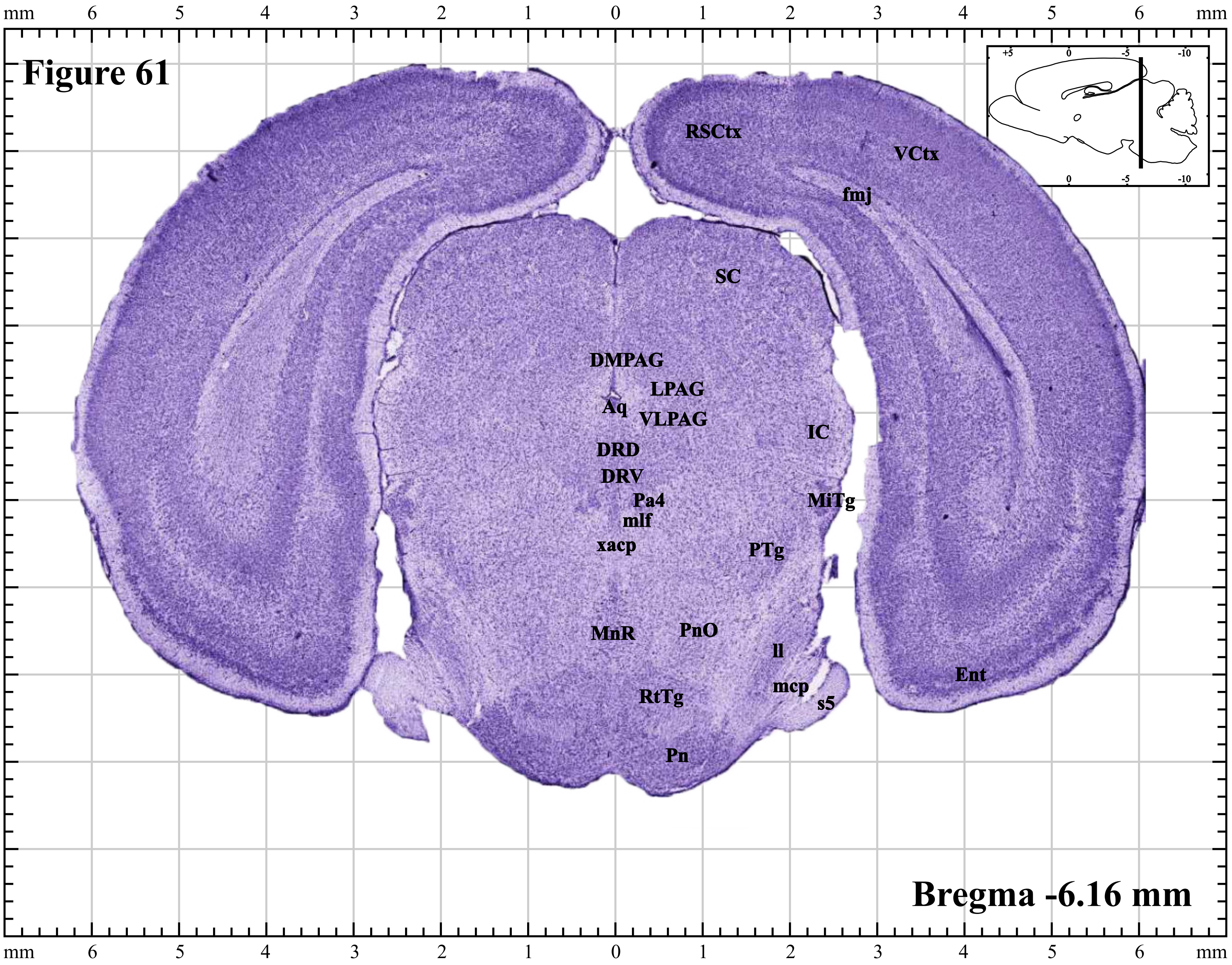

Aq aqueduct
DMPAG dorsomedial periaqueductal gray
DRD dorsomedial hypothalamic nucleus, dorsal part
DRV dorsomedial hypothalamic nucleus, ventral part
Ent entorhinal cortex
fmj forceps major of the corpus callosum
IC inferior colliculus
ll lateral lemniscus
LPAG lateral periaqueductal gray
MnR median raphe nucleus
mlf medial longitudinal fasciculus
mcp middle cerebellar peduncle
MiTg microcellular tegmental nucleus
PnO pontine reticular nucleus, oral part
PTg pedunculopontine tegmental nucleus
Pn pontine nuclei
Pa4 paratrochlear nucleus
RtTg reticulotegmental nucleus of the pons
RSCtx retrospenial cortex
s5 sensory root of the trigeminal nerve
SC superior colliculus
VCtx visual cortex
VLPAG ventrolateral periaqueductal gray
xscp decussation of the superior cerebellar peduncle

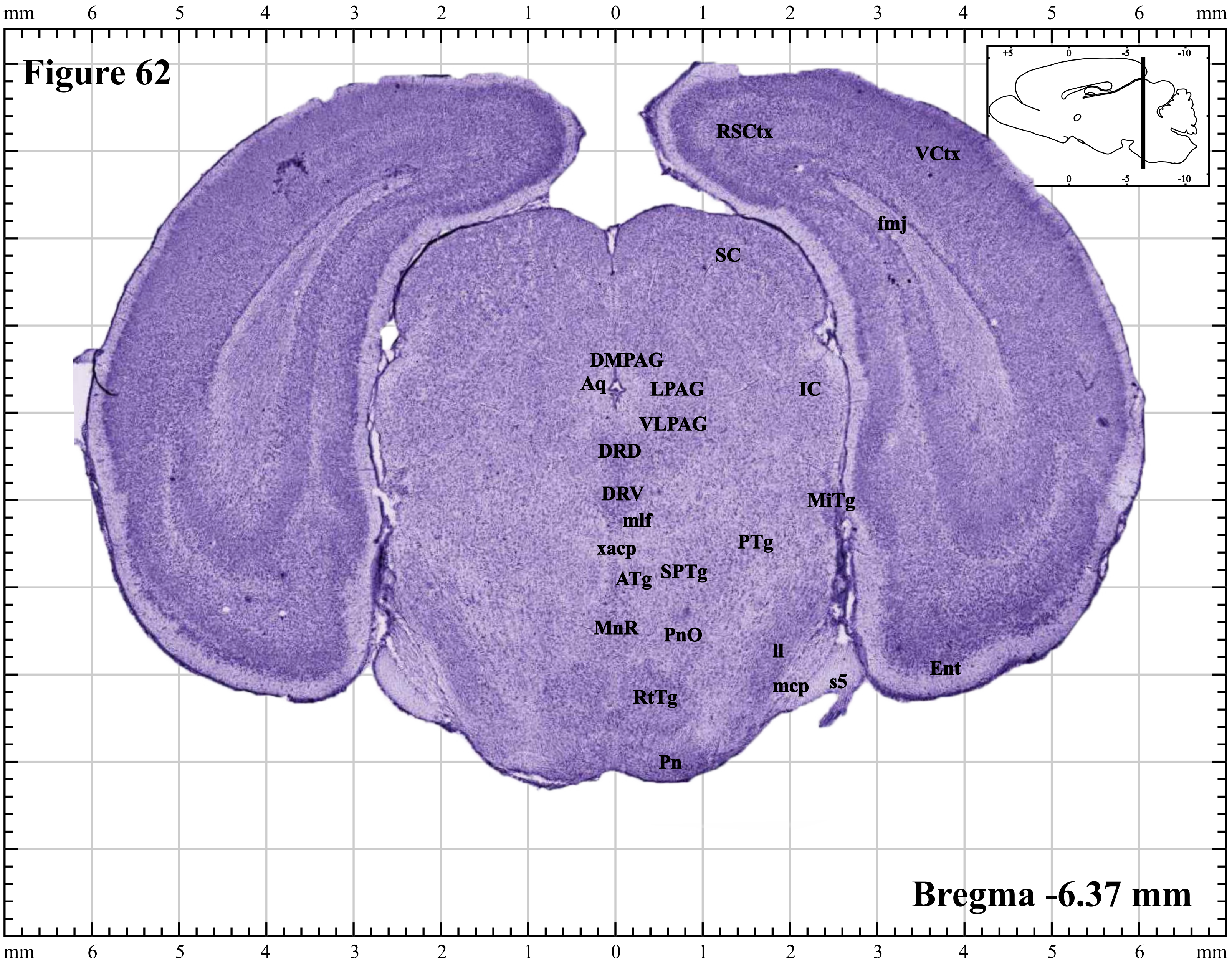

Aq aqueduct
ATg anterior tegmental nucleus
DMPAG dorsomedial periaqueductal gray
DRD dorsomedial hypothalamic nucleus, dorsal part
DRV dorsomedial hypothalamic nucleus, ventral part
Ent entorhinal cortex
fmj forceps major of the corpus callosum
IC inferior colliculus
ll lateral lemniscus
LPAG lateral periaqueductal gray
mlf medial longitudinal fasciculus
mcp middle cerebellar peduncle
MnR median raphe nucleus
MiTg microcellular tegmental nucleus
PnO pontine reticular nucleus, oral part
Pn pontine nuclei
PTg pedunculopontine tegmental nucleus
RtTg reticulotegmental nucleus of the pons
RSCtx retrospenial cortex
s5 sensory root of the trigeminal nerve
SC superior colliculus
SPTg subpeduncular tegmental nucleus
VCtx visual cortex
VLPAG ventrolateral periaqueductal gray
xscp decussation of the superior cerebellar peduncle

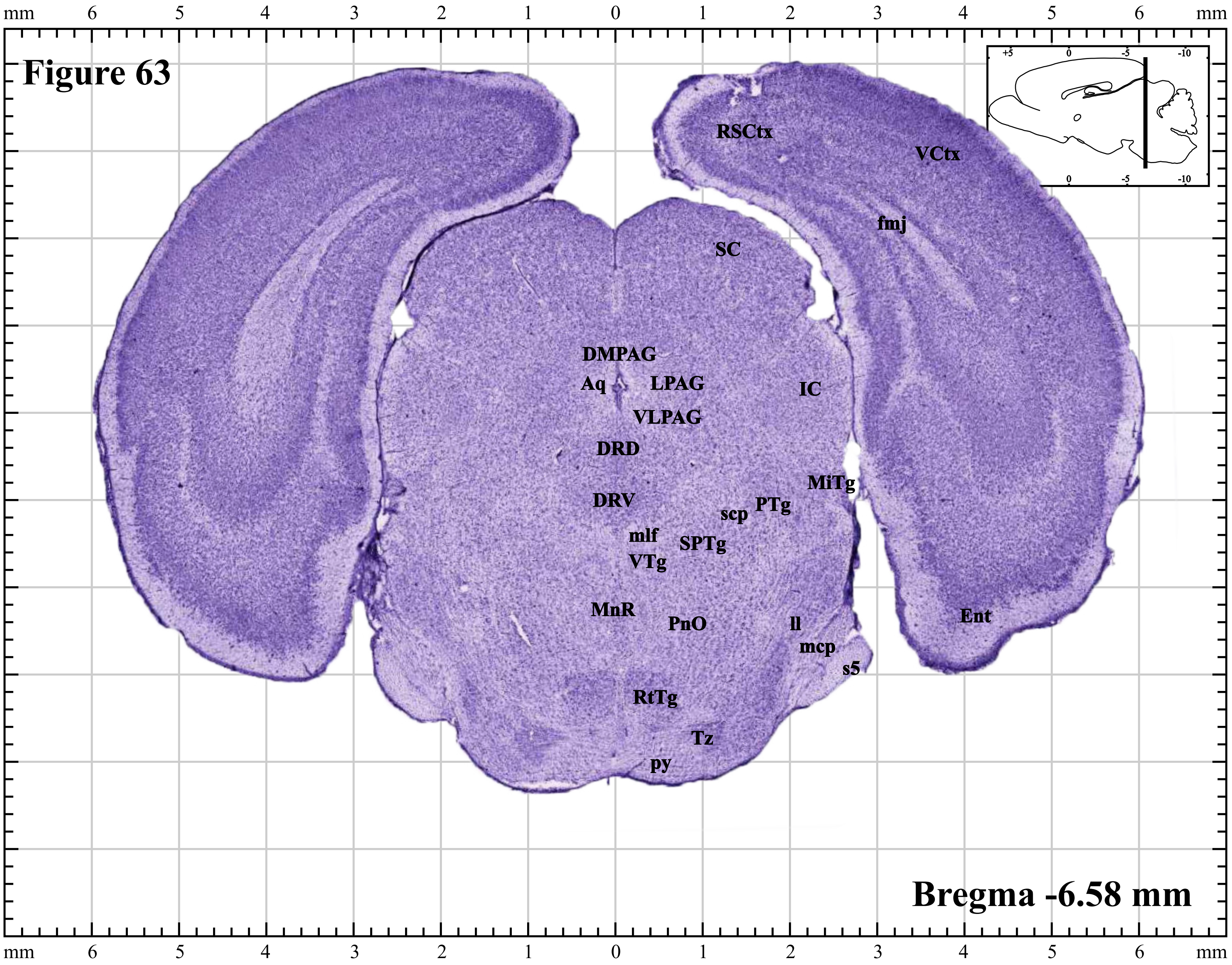

Aq aqueduct
DMPAG dorsomedial periaqueductal gray
DRD dorsomedial hypothalamic nucleus, dorsal part
DRV dorsomedial hypothalamic nucleus, ventral part
Ent entorhinal cortex
fmj forceps major of the corpus callosum
IC inferior colliculus
ll lateral lemniscus
LPAG lateral periaqueductal gray
MnR median raphe nucleus
mlf medial longitudinal fasciculus
mcp middle cerebellar peduncle
py pyramidal tract
PnO pontine reticular nucleus, oral part
PTg pedunculopontine tegmental nucleus
RSCtx retrospenial cortex
RtTg reticulotegmental nucleus of the pons
s5 sensory root of the trigeminal nerve
scp superior cerebellar peduncle
SC superior colliculus
SPTg subpeduncular tegmental nucleus
Tz nucleus of the trapezoid body
VCtx visual cortex
VLPAG ventrolateral periaqueductal gray
VTg ventral tegmental nucleus

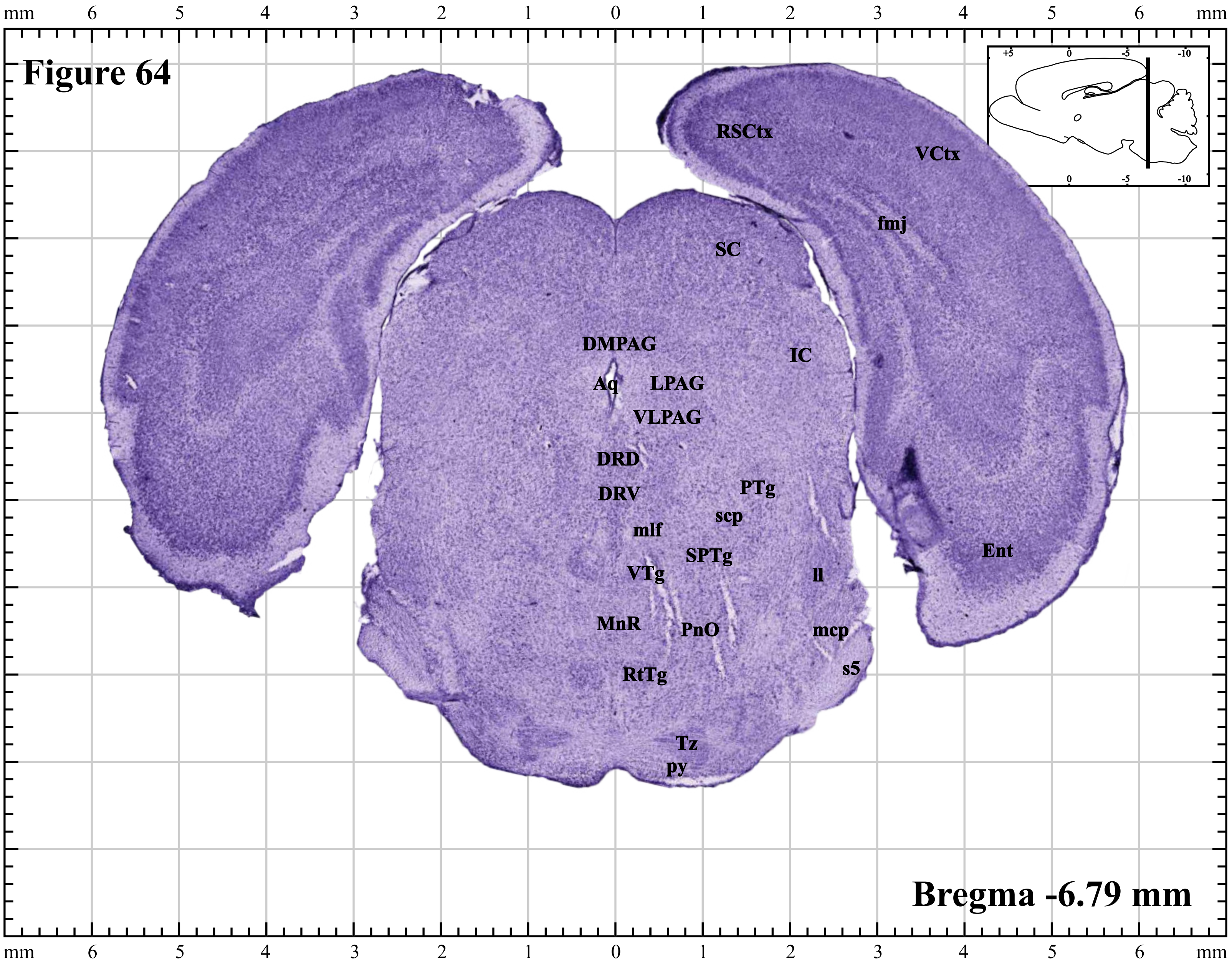

Aq aqueduct
DMPAG dorsomedial periaqueductal gray
DRD dorsomedial hypothalamic nucleus, dorsal part
DRV dorsomedial hypothalamic nucleus, ventral part
Ent entorhinal cortex
fmj forceps major of the corpus callosum
IC inferior colliculus
ll lateral lemniscus
LPAG lateral periaqueductal gray
MnR median raphe nucleus
mlf medial longitudinal fasciculus
mcp middle cerebellar peduncle
py pyramidal tract
PTg pedunculopontine tegmental nucleus
PnO pontine reticular nucleus, oral part
RSCtx retrospenial cortex
RtTg reticulotegmental nucleus of the pons
scp superior cerebellar peduncle
s5 sensory root of the trigeminal nerve
SC superior colliculus
SPTg subpeduncular tegmental nucleus
Tz nucleus of the trapezoid body
VCtx visual cortex
VLPAG ventrolateral periaqueductal gray
VTg ventral tegmental nucleus

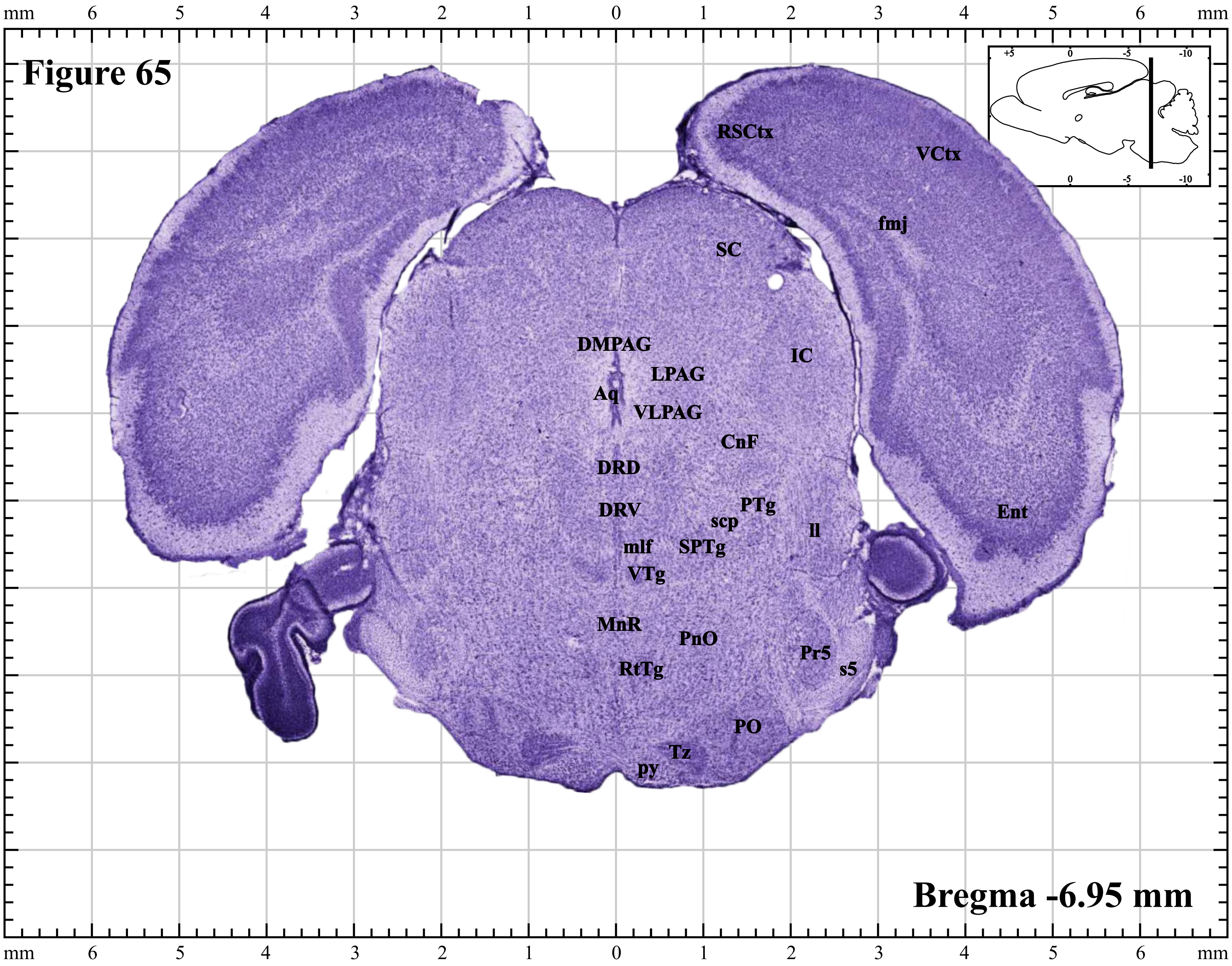

Aq aqueduct
CnF cuneiform nucleus
DMPAG dorsomedial periaqueductal gray
DRD dorsomedial hypothalamic nucleus, dorsal part
DRV dorsomedial hypothalamic nucleus, ventral part
Ent entorhinal cortex
fmj forceps major of the corpus callosum
IC inferior colliculus
ll lateral lemniscus
LPAG lateral periaqueductal gray
MnR median raphe nucleus
mlf medial longitudinal fasciculus
py pyramidal tract
PnO pontine reticular nucleus, oral part
Pr5 principal sensory trigeminal nucleus
PTg pedunculopontine tegmental nucleus
RSCtx retrospenial cortex
RtTg reticulotegmental nucleus of the pons
s5 sensory root of the trigeminal nerve
scp superior cerebellar peduncle
SC superior colliculus
SPTg subpeduncular tegmental nucleus
Tz nucleus of the trapezoid body
VCtx visual cortex
VLPAG ventrolateral periaqueductal gray
VTg ventral tegmental nucleus

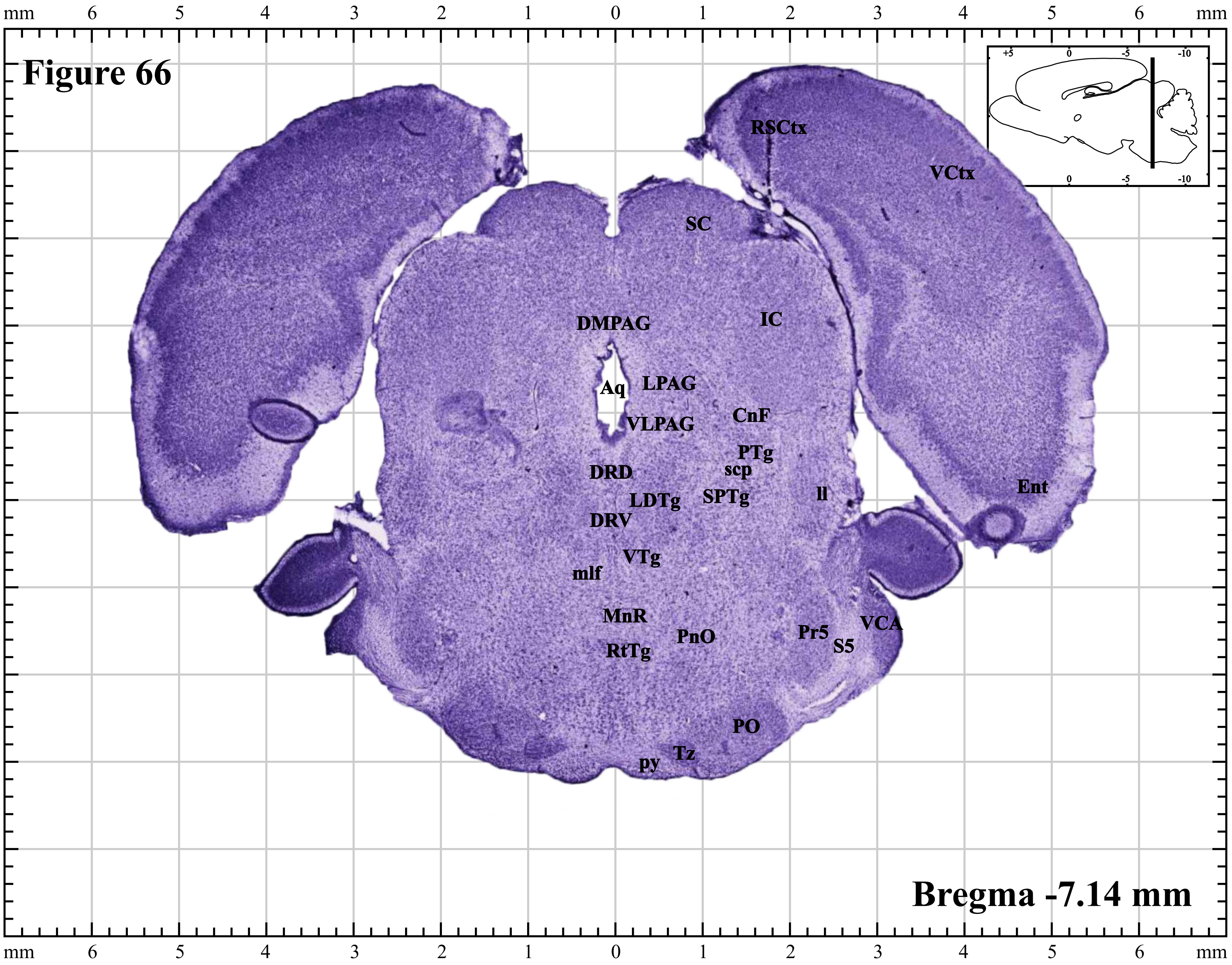

Aq aqueduct
CnF cuneiform nucleus
DMPAG dorsomedial periaqueductal gray
DRD dorsomedial hypothalamic nucleus, dorsal part
DRV dorsomedial hypothalamic nucleus, ventral part
Ent entorhinal cortex
IC inferior colliculus
ll lateral lemniscus
LDTg laterodorsal tegmental nucleus
LPAG lateral periaqueductal gray
MnR median raphe nucleus
mlf medial longitudinal fasciculus
py pyramidal tract
PTg pedunculopontine tegmental nucleus
PnO pontine reticular nucleus, oral part
PO periolivary nucleus
RtTg reticulotegmental nucleus of the pons
RSCtx retrospenial cortex
s5 sensory root of the trigeminal nerve
scp superior cerebellar peduncle
SPTg subpeduncular tegmental nucleus
SC superior colliculus
Tz nucleus of the trapezoid body
VCA ventral cochlear nucleus, anterior part
VCtx visual cortex
VLPAG ventrolateral periaqueductal gray
VTg ventral tegmental nucleus

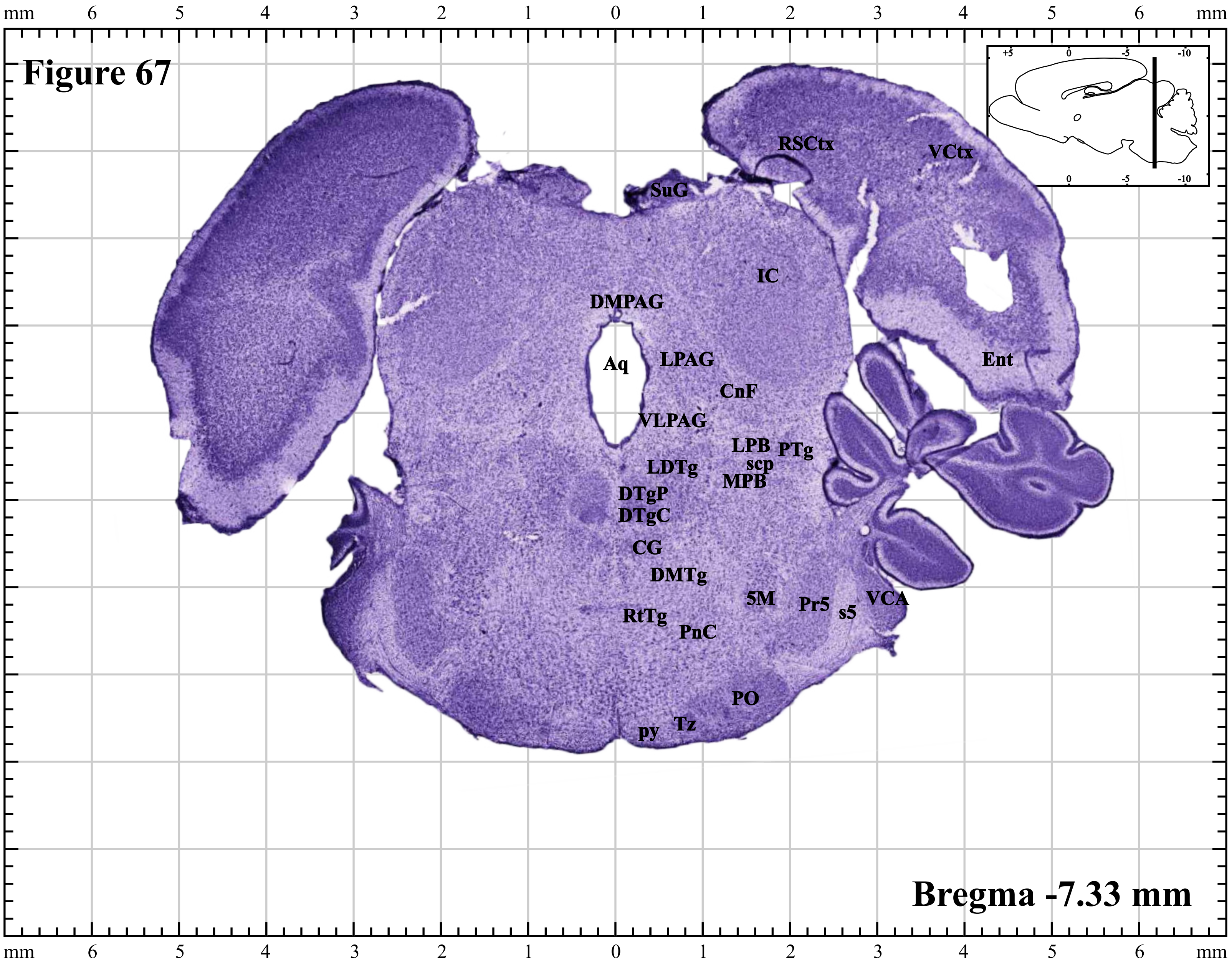

- **5M** motor trigeminal nucleus
- **Aq** aqueduct
- **CG** central gray
- **CnF** cuneiform nucleus
- **DMPAG** dorsomedial periaqueductal gray
- **DTgP** dorsal tegmental nucleus, pericentral part
- **DTgC** dorsal tegmental nucleus, central part
- **Ent** entorhinal cortex
- **IC** inferior colliculus
- **LDTg** laterodorsal tegmental nucleus
- **LPAG** lateral periaqueductal gray
- **LPB** lateral parabrachial nucleus
- **Me5** mesencephalic trigeminal nucleus
- **DMTg** dorsomedial tegmental area
- **MPB** medial parabrachial nucleus
- **py** pyramidal tract
- **PnC** pontine reticular nucleus,caudal part
- **Pr5** principal sensory trigeminal nucleus
- **PO** periolivary nucleus
- **PTg** pedunculopontine tegmental nucleus
- **RSCtx** retrospenial cortex
- **RtTg** reticulotegmental nucleus of the pons
- **s5** sensory root of the trigeminal nerve
- **scp** superior cerebellar peduncle
- **SuG** superficial gray layer of the superior colliculus
- **Tz** nucleus of the trapezoid body
- **VLPAG** ventrolateral periaqueductal gray
- **VCA** ventral cochlear nucleus, anterior part
- **VCtx** visual cortex

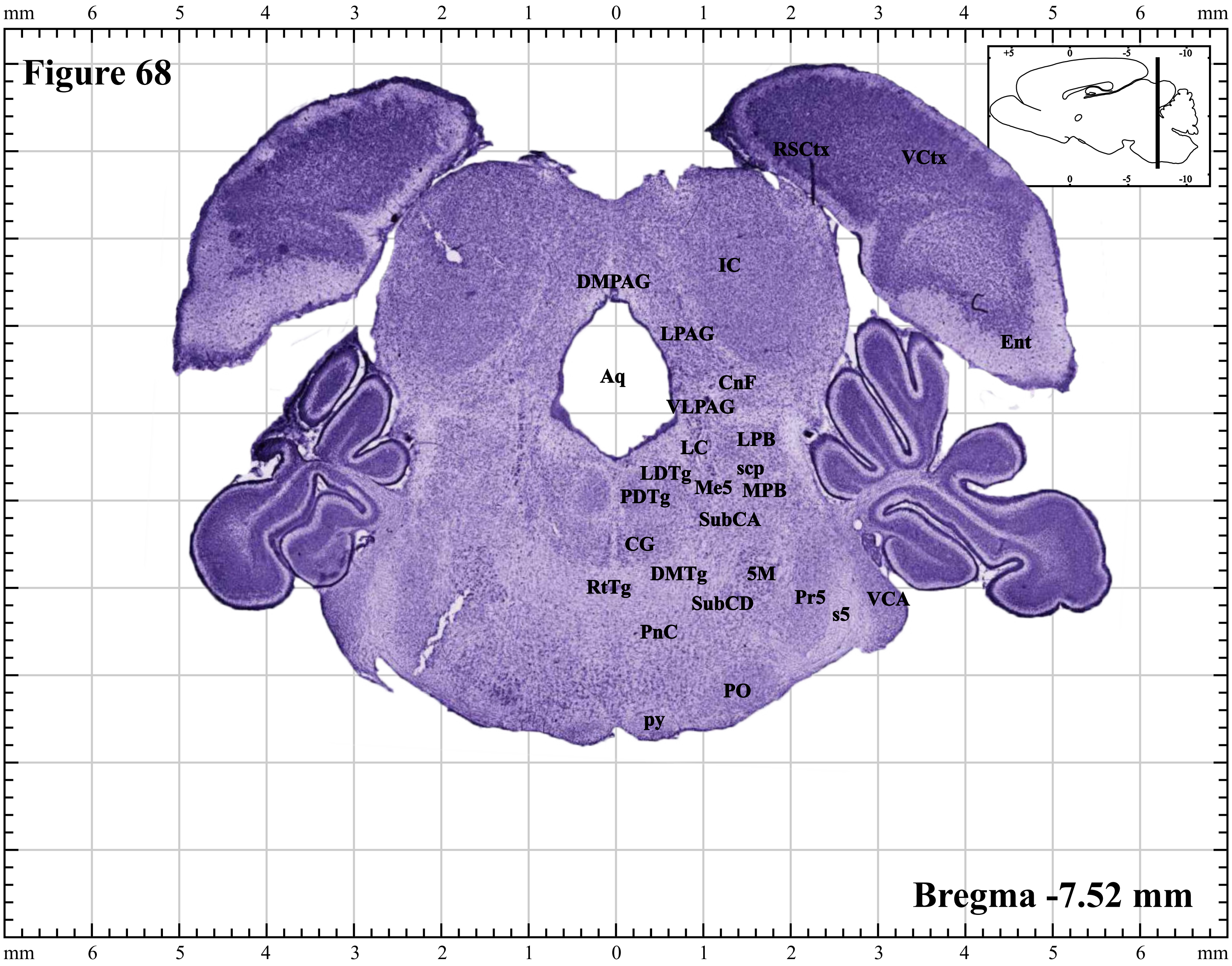

- **5M** motor trigeminal nucleus
- **Aq** aqueduct
- **CG** central gray
- **CnF** cuneiform nucleus
- **DMTg** dorsomedial tegmental area
- **DMPAG** dorsomedial periaqueductal gray
- **DTgP** dorsal tegmental nucleus, pericentral part
- **Ent** entorhinal cortex
- **IC** inferior colliculus
- **LC** locus coeruleus
- **LDTg** laterodorsal tegmental nucleus
- **LPAG** lateral periaqueductal gray
- **LPB** lateral parabrachial nucleus
- **MPB** medial parabrachial nucleus
- **Me5** mesencephalic trigeminal nucleus
- **py** pyramidal tract
- **PO** periolivary nucleus
- **PnC** pontine reticular nucleus,caudal part
- **Pr5** principal sensory trigeminal nucleus
- **PDTg** posterodorsal tegmental nucleus
- **RSCtx** retrospenial cortex
- **RtTg** reticulotegmental nucleus of the pons
- **s5** sensory root of the trigeminal nerve
- **scp** superior cerebellar peduncle
- **SubCA** subcoeruleus nucleus, alpha part
- **SubCD** subcoeruleus nucleus, dorsal part
- **VCtx** visual cortex
- **VLPAG** ventrolateral periaqueductal gray
- **VCA** ventral cochlear nucleus, anterior part
- **VTg** ventral tegmental nucleus

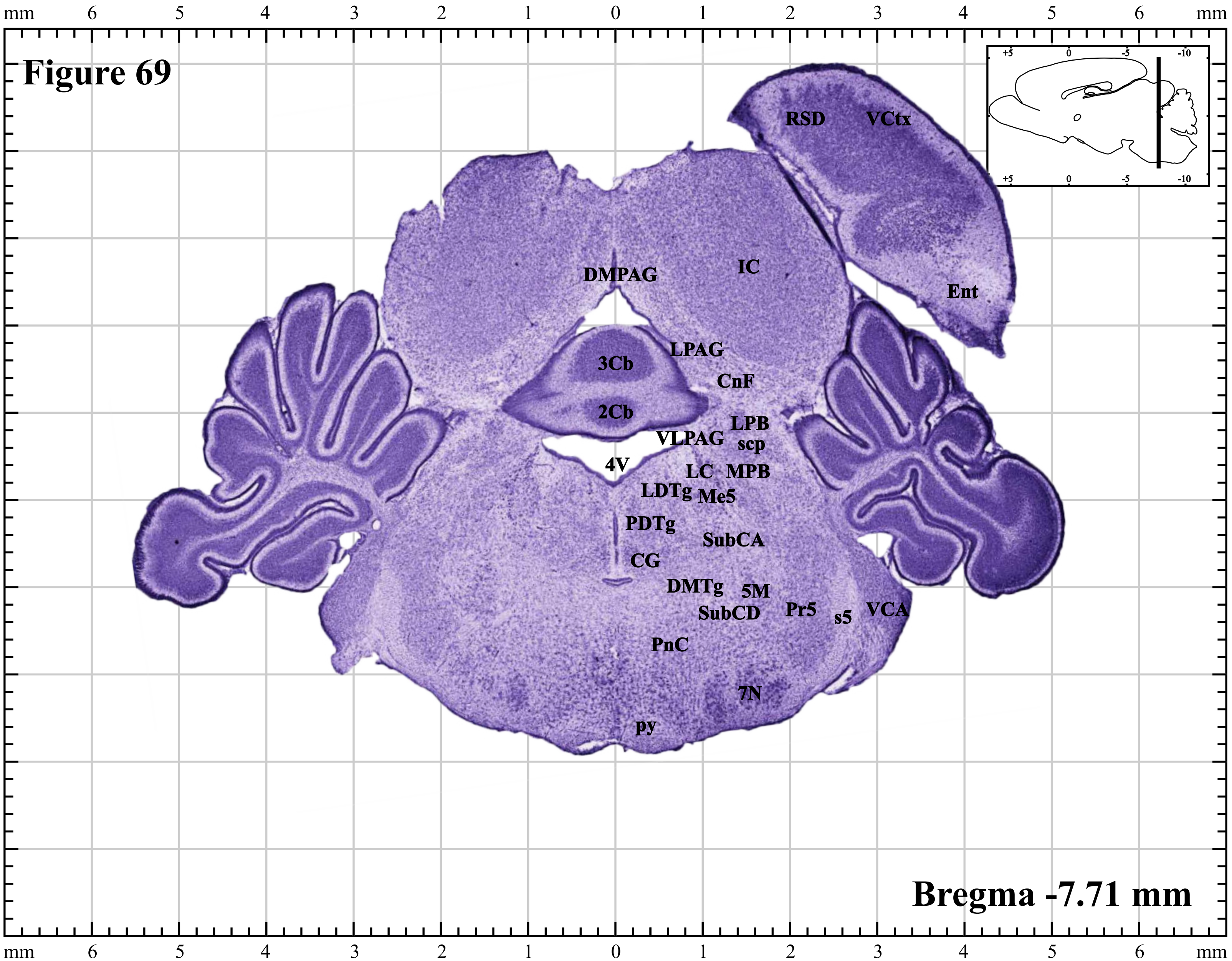

2Cb 2nd cerebellar lobule
3Cb 3rd cerebellar lobule
4V 4th ventricle
5M motor trigeminal nucleus
7N facial nuclues
CG central gray
CnF cuneiform nucleus
DMPAG dorsomedial periaqueductal gray
Ent entorhinal cortex
DMTg dorsomedial tegmental area
IC inferior colliculus
LPAG lateral periaqueductal gray
LC locus coeruleus
LPB lateral parabrachial nucleus
MPB medial parabrachial nucleus
Me5 mesencephalic trigeminal nucleus
py pyramidal tract
PnC pontine reticular nucleus, caudal part
Pr5 principal sensory trigeminal nucleus
RSCtx retrospenial cortex
s5 sensory root of the trigeminal nerve
SubCA subcoeruleus nucleus, alpha part
SubCD subcoeruleus nucleus, dorsal part
VCtx visual cortex
VLPAG ventrolateral periaqueductal gray
VCA ventral cochlear nucleus, anterior part

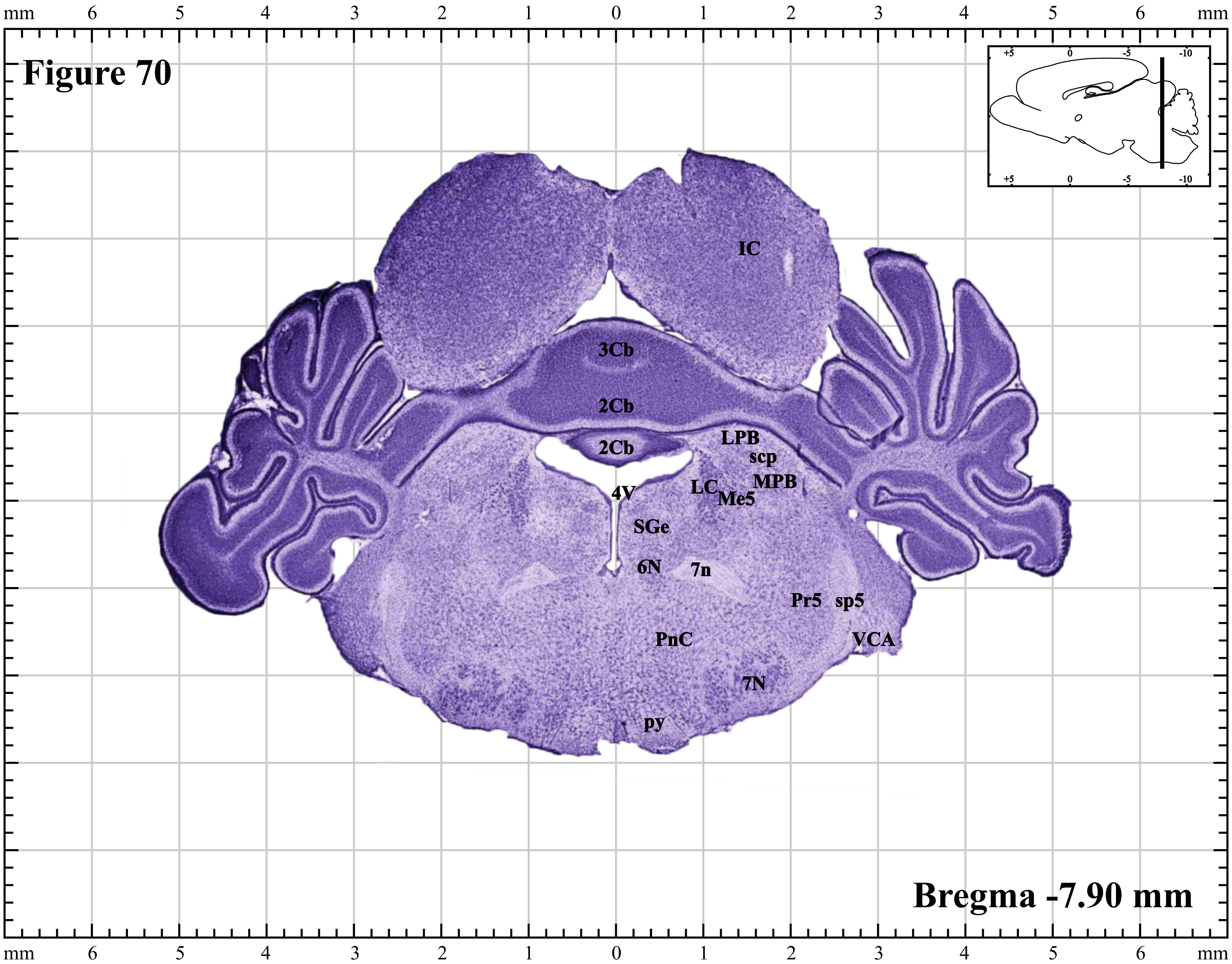

2Cb 2nd cerebellar lobule
3Cb 3rd cerebellar lobule
4V 4th ventricle
6N abducens nucleus
7n facial nerve
7N facial nuclues
IC inferior colliculus
LC locus coeruleus
LPB lateral parabrachial nucleus
Me5 mesencephalic trigeminal nucleus
MPB medial parabrachial nucleus
py pyramidal tract
PnC pontine reticular nucleus, caudal part
Pr5 principal sensory trigeminal nucleus
sp5 spinal trigeminal tract
scp superior cerebellar peduncle
SGe supragenual nucleus
SubCD subcoeruleus nucleus, dorsal part
VCA ventral cochlear nucleus, anterior part

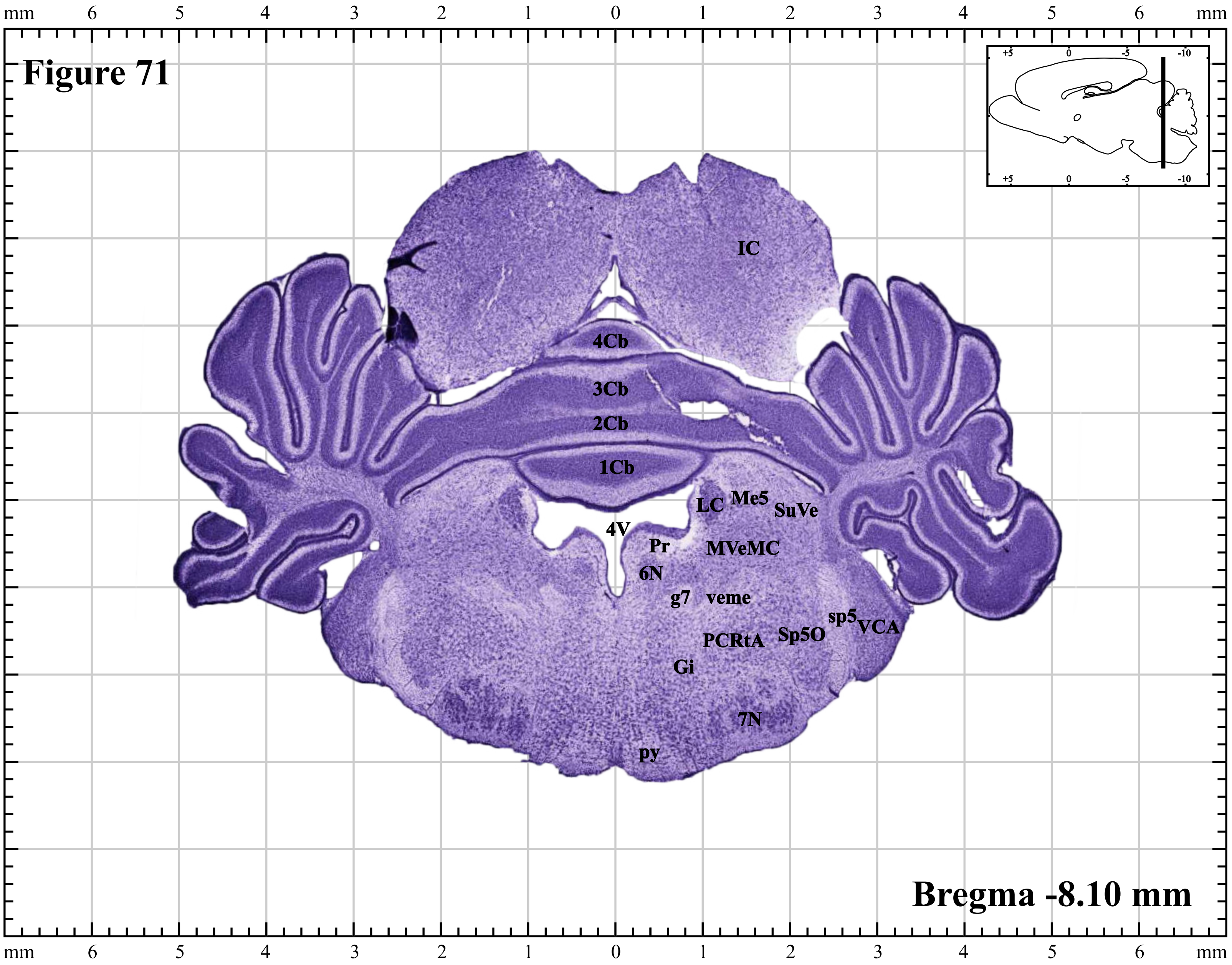

1Cb 1st cerebellar lobule (lingula)
2Cb 2nd cerebellar lobule
3Cb 3rd cerebellar lobule
4Cb 4th cerebellar lobule
4V 4th ventricle
6N abducens nucleus
7N facial nuclues
g7 genu of the facial nerve
Gi granular insular cortex
IC inferior colliculus
LC locus coeruleus
Me5 mesencephalic trigeminal nucleus
MVeMC medial vestibular nucleus, magnocellular part
PCRtA parvicellular reticular nucleus, alpha part
Pr prepositus nucleus
SuVe superior vestibular nucleus
Sp5O spinal trigeminal nucleus, oral part
SubCD subcoeruleus nucleus, dorsal part
sp5 spinal trigeminal tract
VCA ventral cochlear nucleus, anterior part
veme vestibulomesencephalic tract

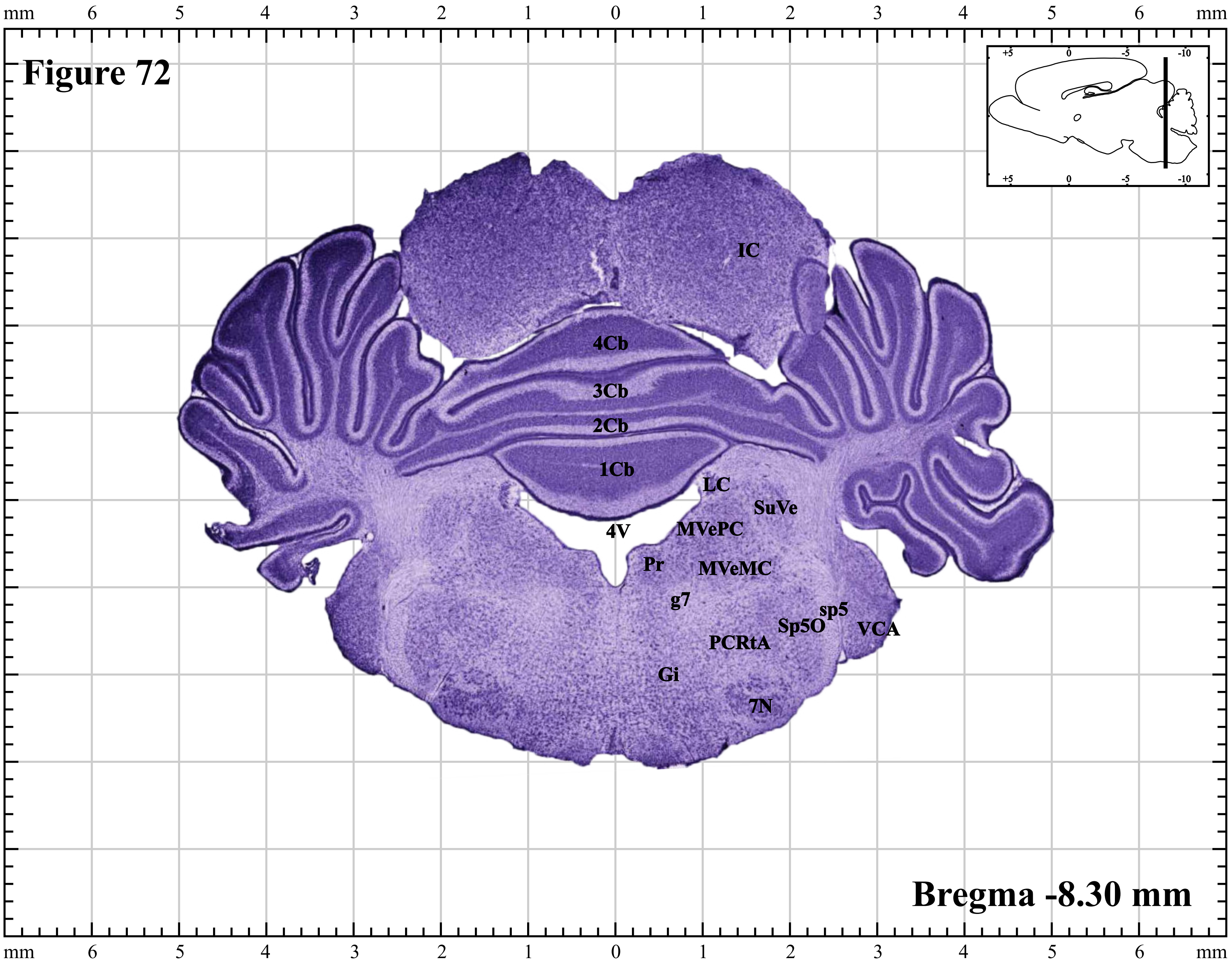

Figure 72

Bregma -8.30 mm

1Cb 1st cerebellar lobule (lingula)
2Cb 2nd cerebellar lobule
3Cb 3rd cerebellar lobule
4Cb 4th cerebellar lobule
4V 4th ventricle
7N facial nuclues
g7 genu of the facial nerve
Gi granular insular cortex
IC inferior colliculus
LC locus coeruleus
Me5 mesencephalic trigeminal nucleus
MVeMC medial vestibular nucleus, magnocellular part
MVePC medial vestibular nucleus, parvicellular part
PCRtA parvicellular reticular nucleus, alpha part
Pr prepositus nucleus
SuVe superior vestibular nucleus
Sp5O spinal trigeminal nucleus, oral part
SubCD subcoeruleus nucleus, dorsal part
sp5 spinal trigeminal tract
VCA ventral cochlear nucleus, anterior part

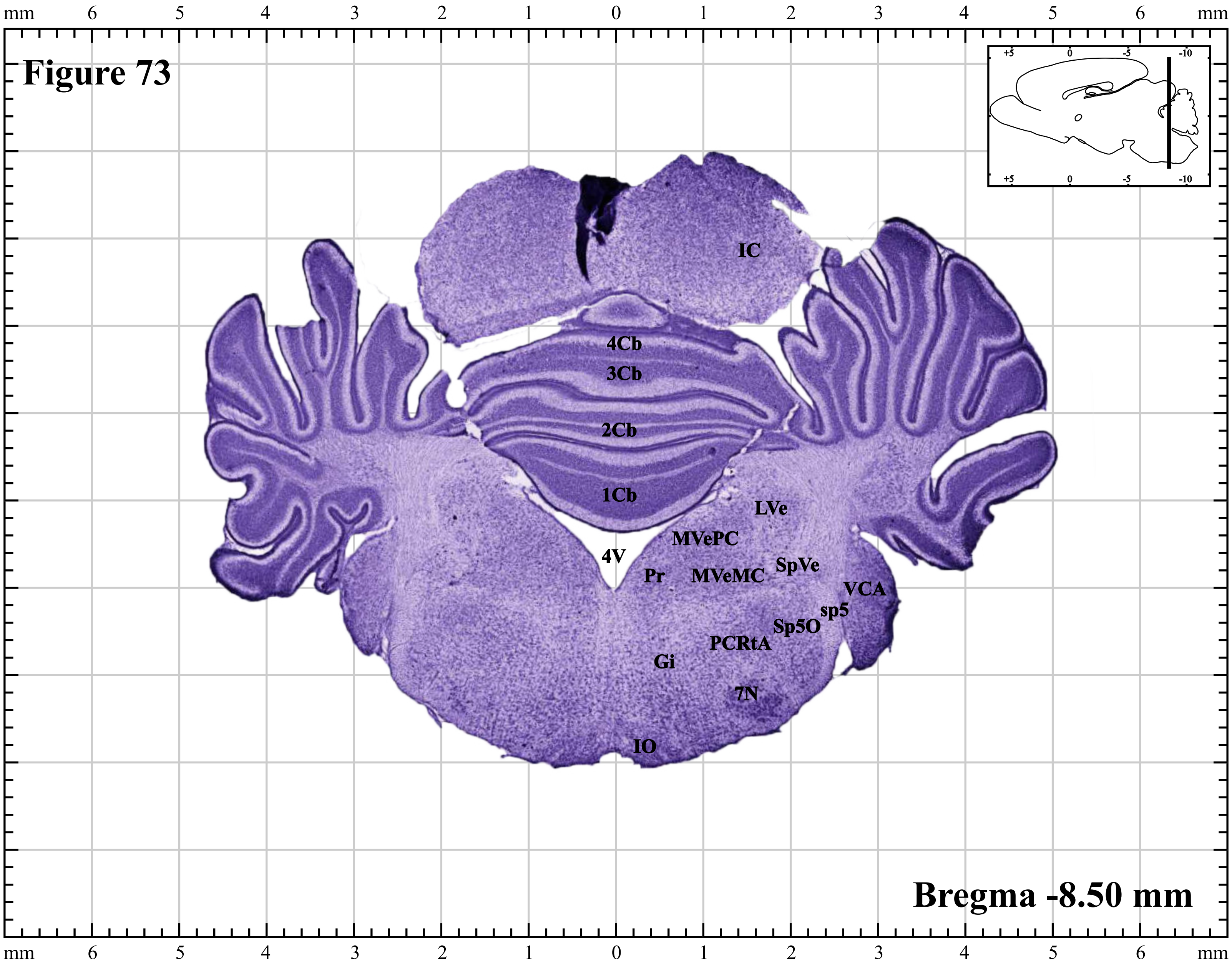

1Cb 1st cerebellar lobule (lingula)
2Cb 2nd cerebellar lobule
3Cb 3rd cerebellar lobule
4Cb 4th cerebellar lobule
4V 4th ventricle
7N facial nuclues
Gi granular insular cortex
IC inferior colliculus
IO inferior olive
LVe lateral vestibular nucleus
Me5 mesencephalic trigeminal nucleus
MVeMC medial vestibular nucleus, magnocellular part
MVePC medial vestibular nucleus, parvicellular part
PCRtA parvicellular reticular nucleus, alpha part
Pr prepositus nucleus
sp5 spinal trigeminal tract
SpVe spinal vestibular nucleus
Sp5O spinal trigeminal nucleus, oral part
SubCD subcoeruleus nucleus, dorsal part
VCA ventral cochlear nucleus, anterior part

Figure 74

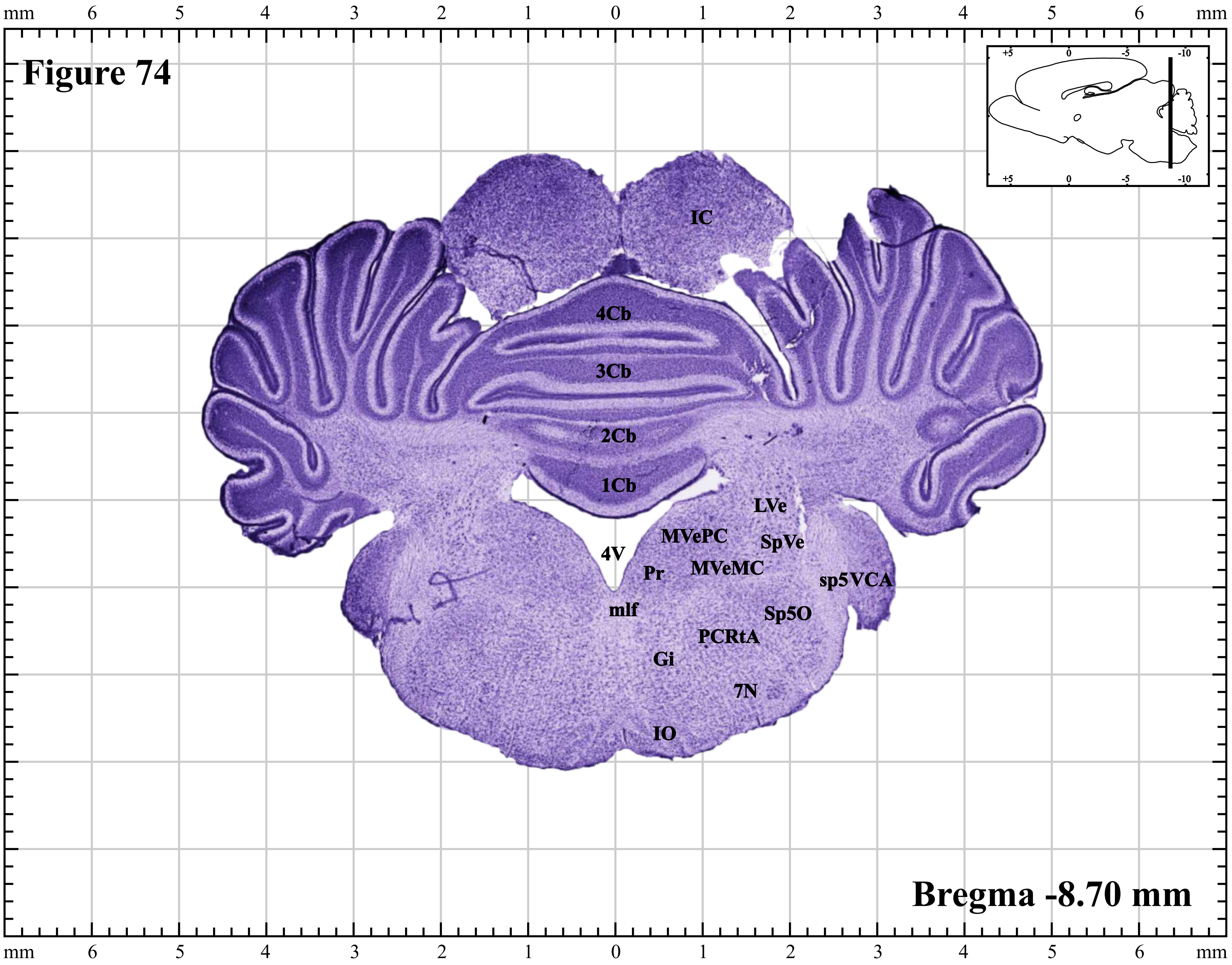

Bregma -8.70 mm

1Cb 1st cerebellar lobule (lingula)
2Cb 2nd cerebellar lobule
3Cb 3rd cerebellar lobule
4Cb 4th cerebellar lobule
4V 4th ventricle
7N facial nuclues
Gi granular insular cortex
IO inferior olive
IC inferior colliculus
LVe lateral vestibular nucleus
Me5 mesencephalic trigeminal nucleus
MVeMC medial vestibular nucleus, magnocellular part
MVePC medial vestibular nucleus, parvicellular part
mlf medial longitudinal fasciculus
PCRtA parvicellular reticular nucleus, alpha part
Pr prepositus nucleus
sp5 spinal trigeminal tract
SpVe spinal vestibular nucleus
Sp5O spinal trigeminal nucleus, oral part
SubCD subcoeruleus nucleus, dorsal part
VCA ventral cochlear nucleus, anterior part

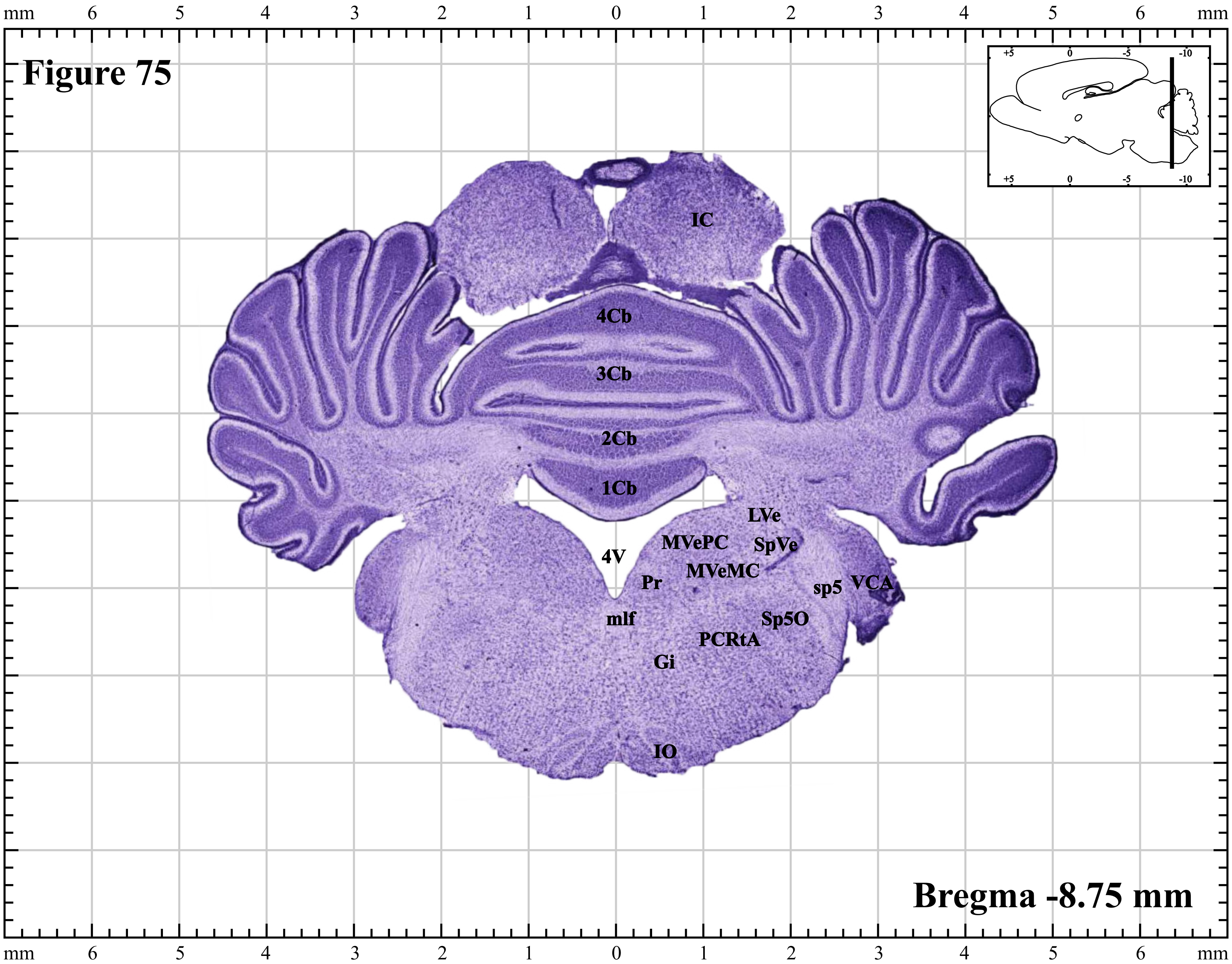

1Cb 1st cerebellar lobule (lingula)
2Cb 2nd cerebellar lobule
3Cb 3rd cerebellar lobule
4Cb 4th cerebellar lobule
4V 4th ventricle
Gi granular insular cortex
IO inferior olive
IC inferior colliculus
LVe lateral vestibular nucleus
Me5 mesencephalic trigeminal nucleus
MVeMC medial vestibular nucleus, magnocellular part
MVePC medial vestibular nucleus, parvicellular part
mlf medial longitudinal fasciculus
PCRtA parvicellular reticular nucleus, alpha part
Pr prepositus nucleus
sp5 spinal trigeminal tract
SpVe spinal vestibular nucleus
SubCD subcoeruleus nucleus, dorsal part
Sp5O spinal trigeminal nucleus, oral part
VCA ventral cochlear nucleus, anterior part

Figure 76

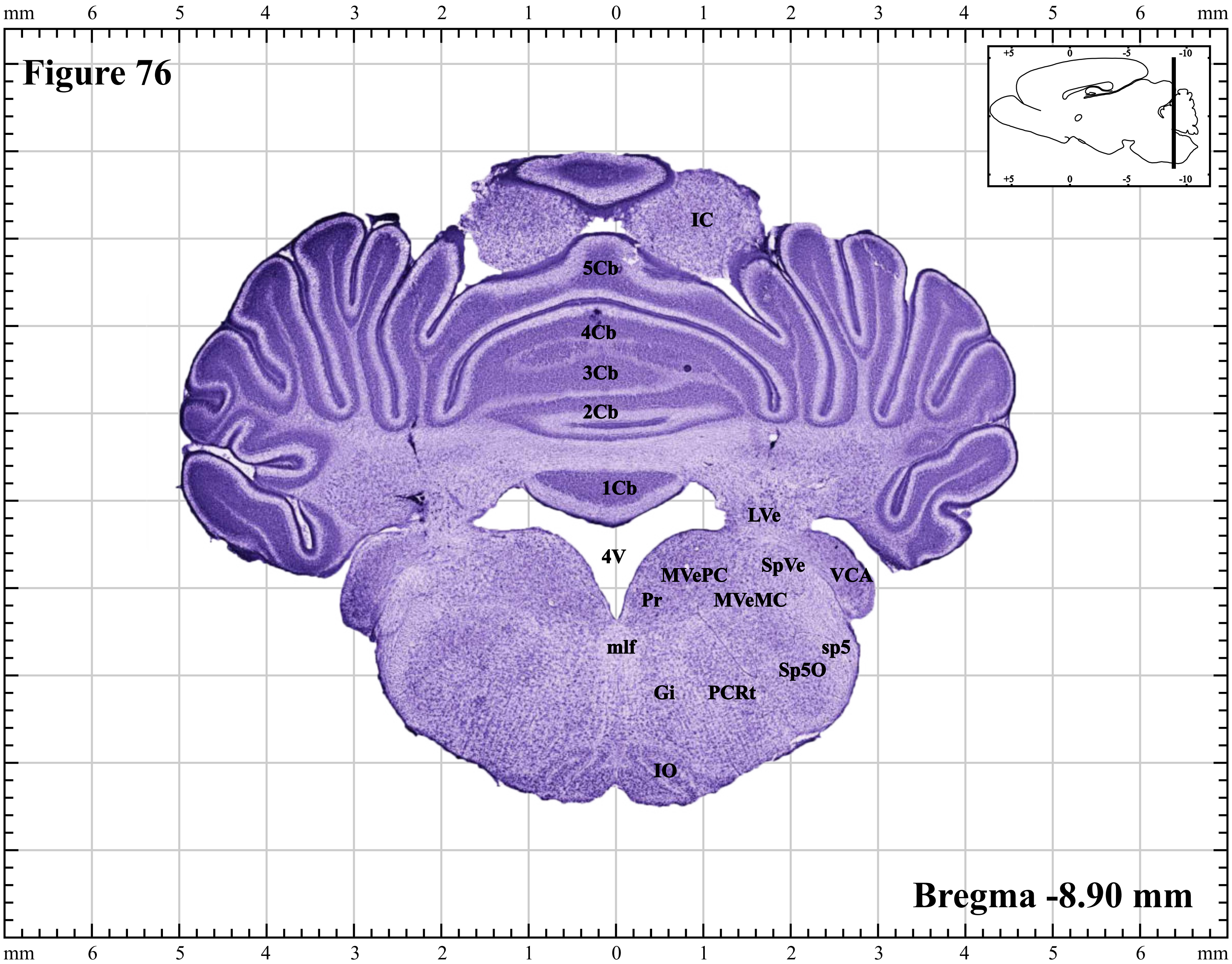

Bregma -8.90 mm

1Cb 1st cerebellar lobule (lingula)
2Cb 2nd cerebellar lobule
3Cb 3rd cerebellar lobule
4Cb 4th cerebellar lobule
4V 4th ventricle
5Cb 5th cerebellar lobule
Gi granular insular cortex
IO inferior olive
IC inferior colliculus
LVe lateral vestibular nucleus
Me5 mesencephalic trigeminal nucleus
MVeMC medial vestibular nucleus, magnocellular part
MVePC medial vestibular nucleus, parvicellular part
mlf medial longitudinal fasciculus
PCRt parvicellular reticular nucleus
Pr prepositus nucleus
sp5 spinal trigeminal tract
SpVe spinal vestibular nucleus
Sp5O spinal trigeminal nucleus, oral part
SubCD subcoeruleus nucleus, dorsal part
VCA ventral cochlear nucleus, anterior part

Figure 77

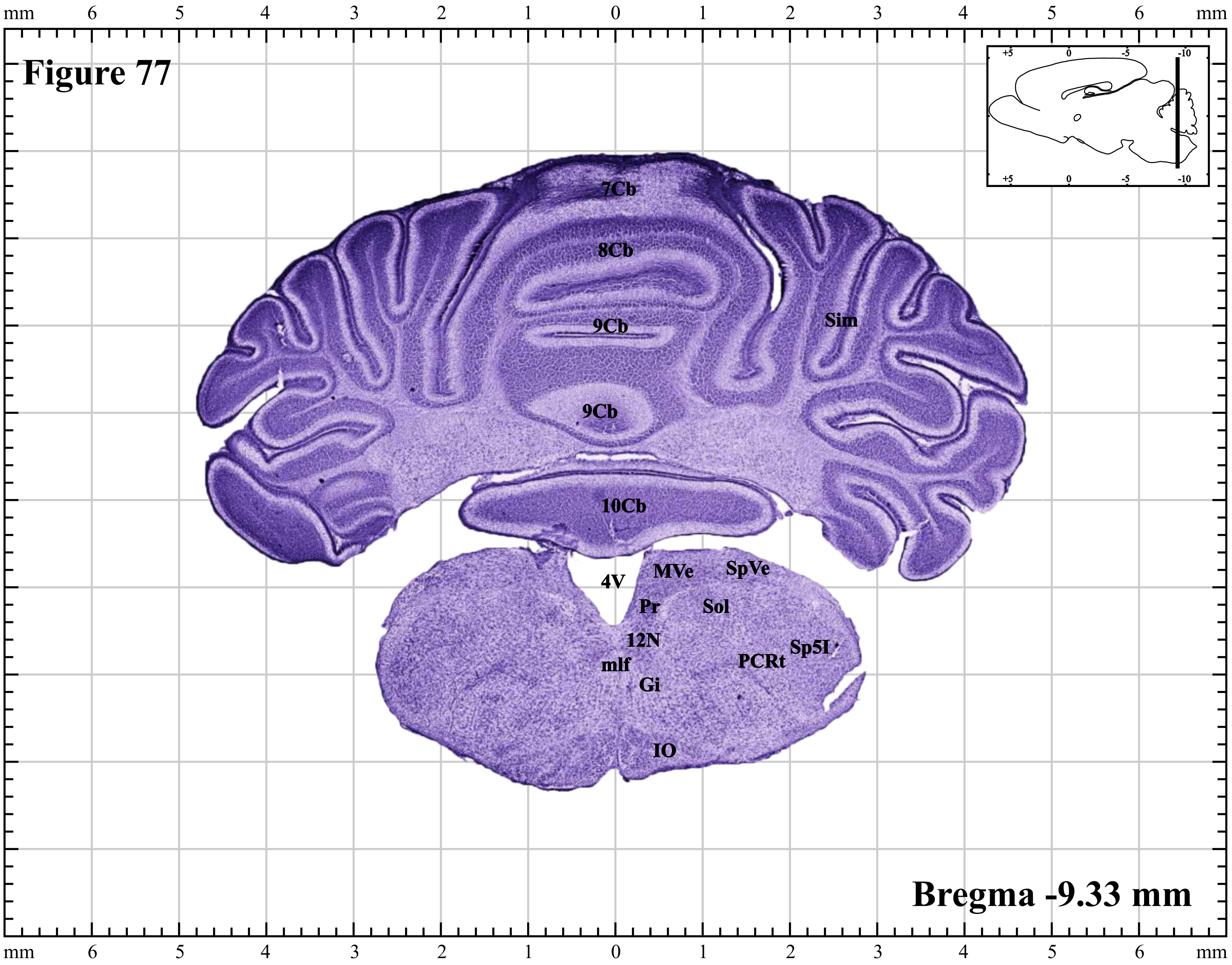

Bregma -9.33 mm

4V 4th ventricle
7Cb 7th cerebellar lobule (lingula)
8Cb 8th cerebellar lobule
9Cb 9th cerebellar lobules
10Cb 10th cerebellar lobule
12N hypoglossal nucleus
Gi granular insular cortex
IO inferior olive
mlf medial longitudinal fasciculus
MVe medial vestibular nucleus
PCRt parvicellular reticular nucleus
Pr prepositus nucleus
Sol nucleus of the solitary tract
Sim simple lobule
SpVe spinal vestibular nucleus
Sp5I spinal trigeminal nucleus, interpolar part

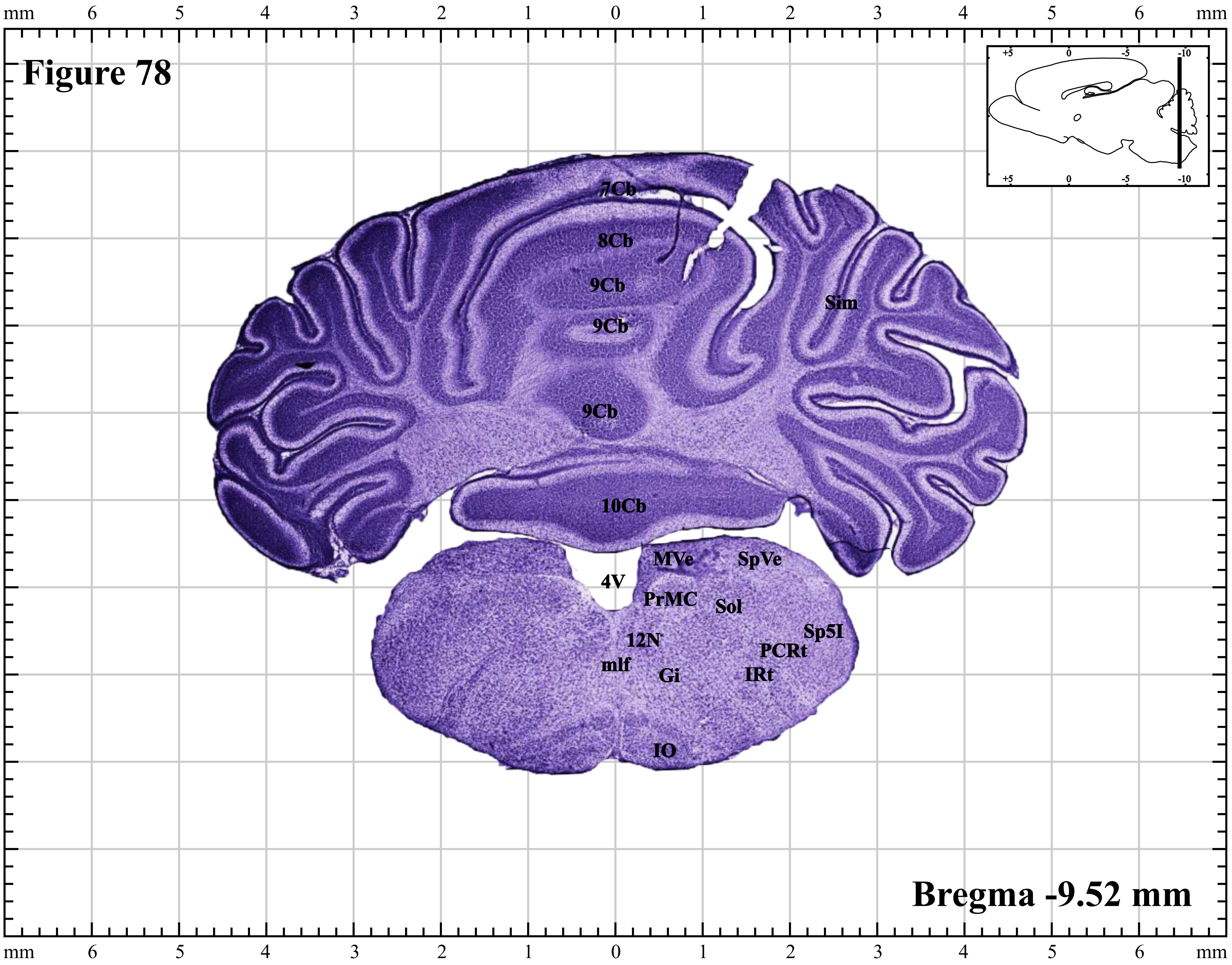

4V 4th ventricle
7Cb 7th cerebellar lobule (lingula)
8Cb 8th cerebellar lobule
9Cb 9th cerebellar lobules
10Cb 10th cerebellar lobule
12N hypoglossal nucleus
Gi granular insular cortex
IO inferior olive
IRt intermediate reticular nucleus
mlf medial longitudinal fasciculus
MVe medial vestibular nucleus
PCRt parvicellular reticular nucleus
PrMC prepositus nucleus,magnocellul
Sol nucleus of the solitary tract
Sim simple lobule
SpVe spinal vestibular nucleus
Sp5I spinal trigeminal nucleus, interpolar part

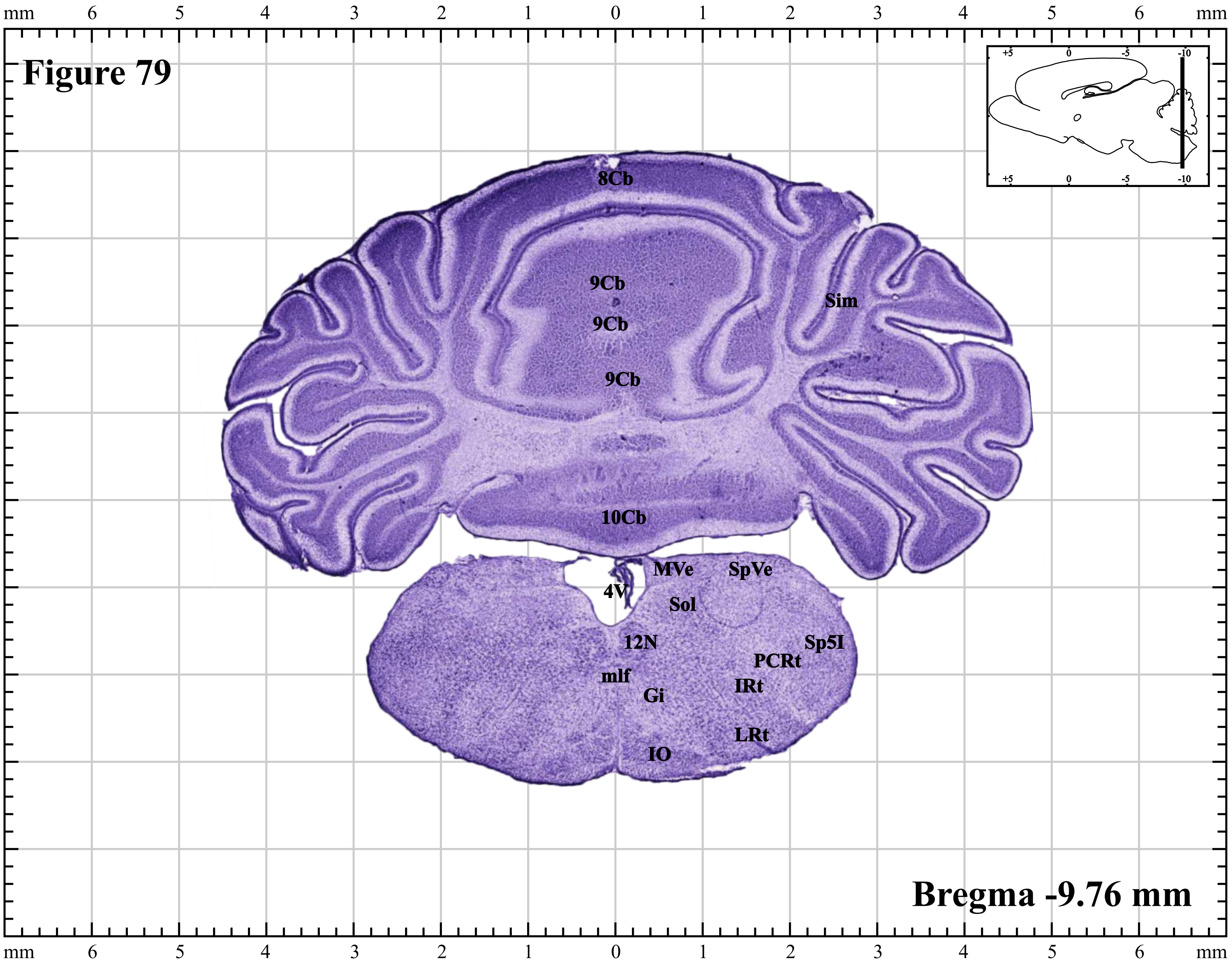

4V 4th ventricle
8Cb 8th cerebellar lobule
9Cb 9th cerebellar lobules
10Cb 10th cerebellar lobule
12N hypoglossal nucleus
Gi granular insular cortex
IO inferior olive
IRt intermediate reticular nucleus
LRt lateral reticular nucleus
mlf medial longitudinal fasciculus
MVe medial vestibular nucleus
PCRt parvicellular reticular nucleus
Sol nucleus of the solitary tract
Sim simple lobule
SpVe spinal vestibular nucleus
Sp5I spinal trigeminal nucleus, interpolar part

Figure 80

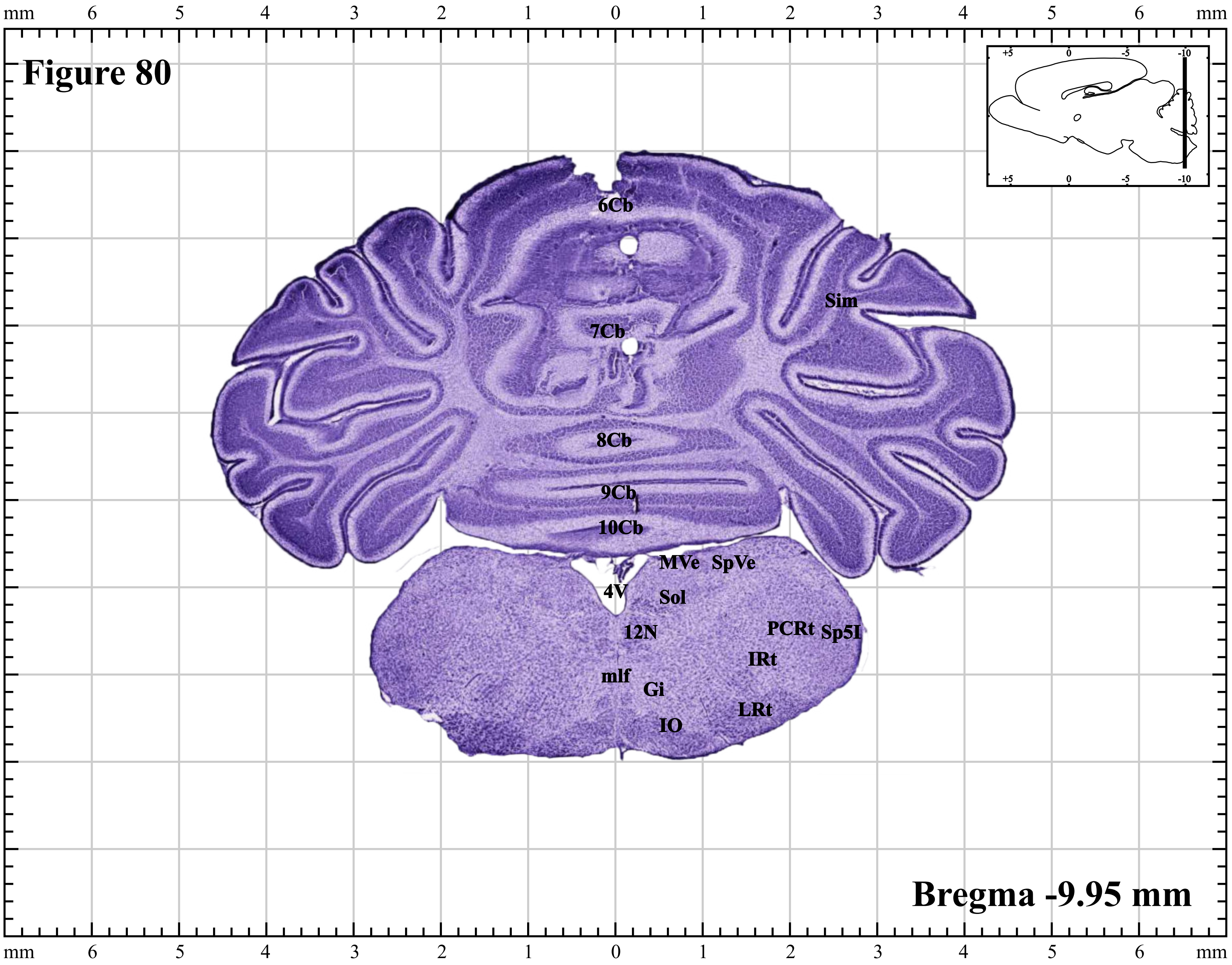

Bregma -9.95 mm

4V 4th ventricle
6Cb 6th cerebellar lobule
7Cb 7th cerebellar lobule
8Cb 8th cerebellar lobule
9Cb 9th cerebellar lobules
Gi granular insular cortex
12N hypoglossal nucleus
10Cb 10th cerebellar lobule
IO inferior olive
IRt intermediate reticular nucleus
LRt lateral reticular nucleus
mlf medial longitudinal fasciculus
MVe medial vestibular nucleus
PCRt parvicellular reticular nucleus
Sol nucleus of the solitary tract
Sim simple lobule
SpVe spinal vestibular nucleus
Sp5I spinal trigeminal nucleus, interpolar part

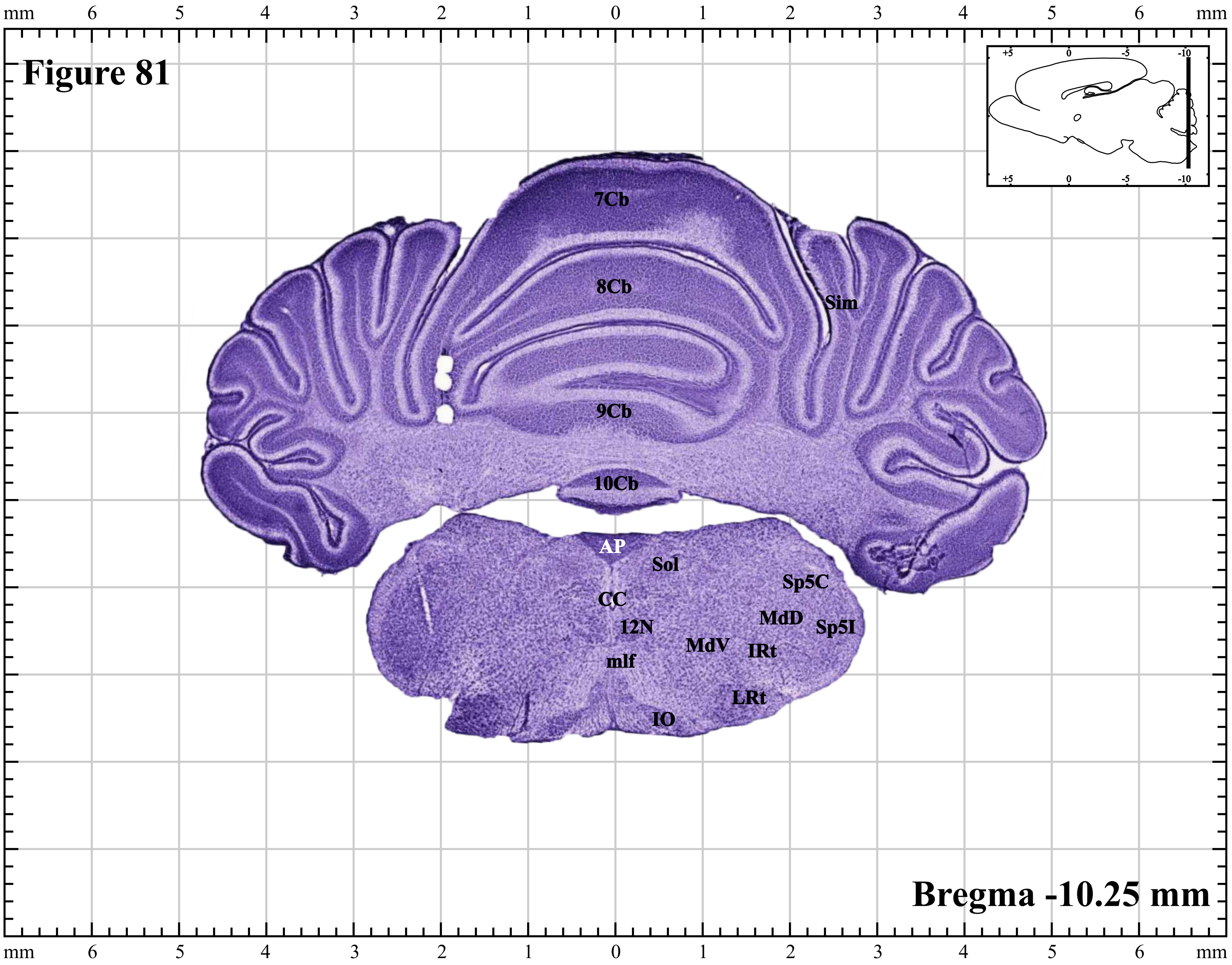

4V 4th ventricle
7Cb 7th cerebellar lobule
8Cb 8th cerebellar lobule
9Cb 9th cerebellar lobules
10Cb 10th cerebellar lobule
12N hypoglossal nucleus
AP area postrema
CC central canal
IO inferior olive
IRt intermediate reticular nucleus
LRt lateral reticular nucleus
mlf medial longitudinal fasciculus
MdD medullary reticular nucleus, dorsal part
MdV medullary reticular nucleus, ventral part
Sol nucleus of the solitary tract
Sim simple lobule
Sp5I spinal trigeminal nucleus, interpolar part
Sp5C spinal trigeminal nucleus, caudal part
