# Supplementary material for: Stereotaxic atlas of the infant rat brain at postnatal days 7–13
Source: Front Neuroanat. 2022 Aug 12;16:968320. doi: 10.3389/fnana.2022.968320 (PMC9412974; doi:10.3389/fnana.2022.968320)
Supplement: Supplementary file 7 [file Data_Sheet_7.PDF]

frontiers

Supplementary Material 7

Stereotaxic Atlas of the Infant Rat Brain

P13 (# G-12-3, 33.8 g)

Yu-Nong Chen[1], Xin Zheng[1], Hai-Lin Chen[1], Jin-Xian Gao[1], Xin-Xuan Li[1], Jun-Fan Xie[1], Yu-Ping Xie[3], Karen Spruyt[4], Yu-Feng Shao[1,2]* and Yi-Ping Hou[1,2]*

[1]Departments of Neuroscience, Anatomy, Histology, and Embryology, Key Laboratory of Preclinical Study for New Drugs of Gansu Province, School of Basic Medical Sciences, Lanzhou University, Lanzhou, China
[2]Key Lab of Neurology of Gansu Province, Lanzhou University, Lanzhou, China
[3]Sleep Medicine Center of Gansu Provincial Hospital, Lanzhou, China
[4]Université de Paris, NeuroDiderot – INSERM, Paris, France.

* Correspondence: Yu-Feng Shao (shaoyf@lzu.edu.cn); Yi-Ping Hou (houyiping@lzu.edu.cn)

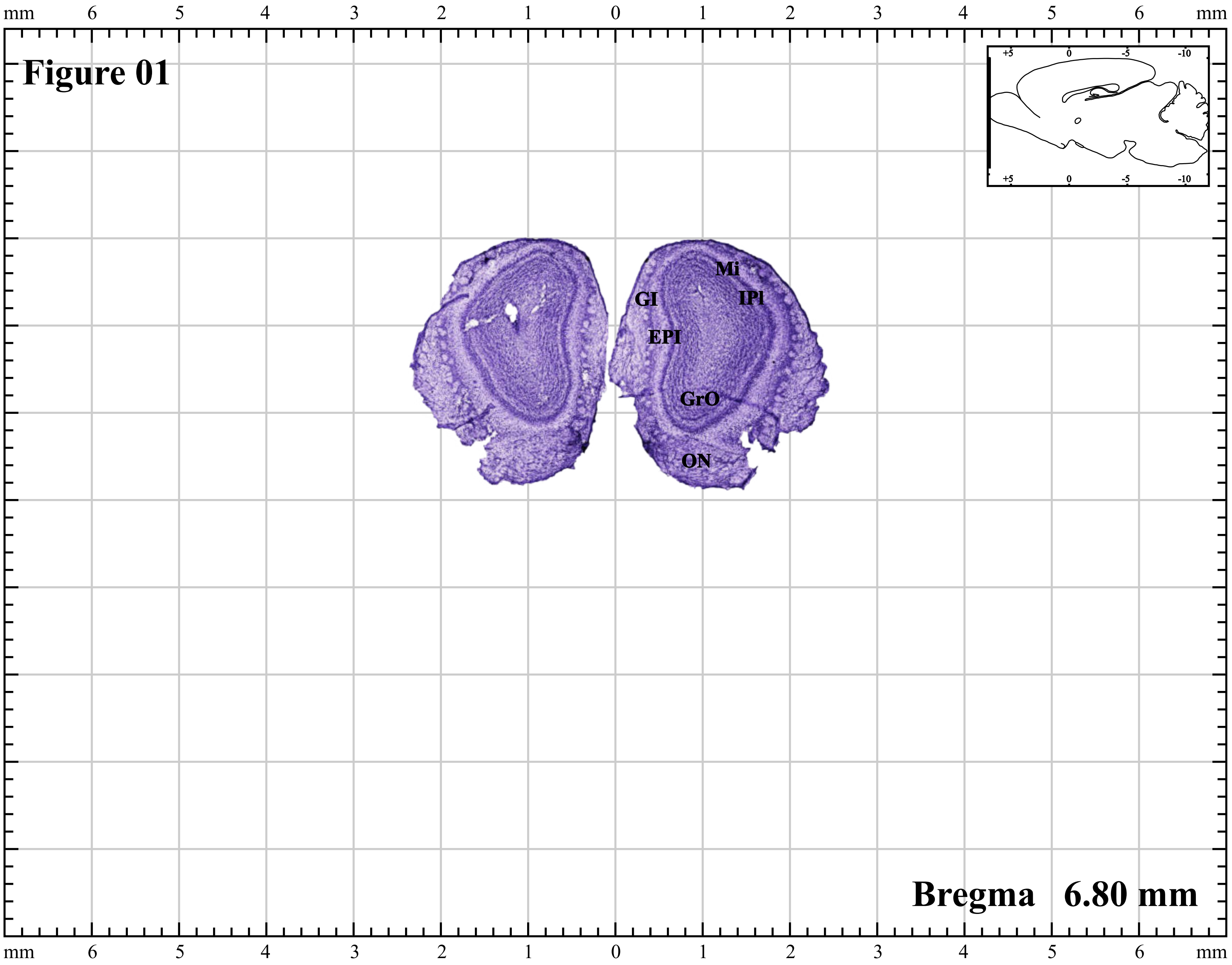

EPl external plexiform layer
of the olfactory bulb
GrO granular cell layer of
the olfactory bulb
GI granular insular cortex
ON olfactory nerve layer
IPl internal plexiform layer of
the olfactory bulb
Mi mitral cell layer of the olfactory bulb

Figure 02

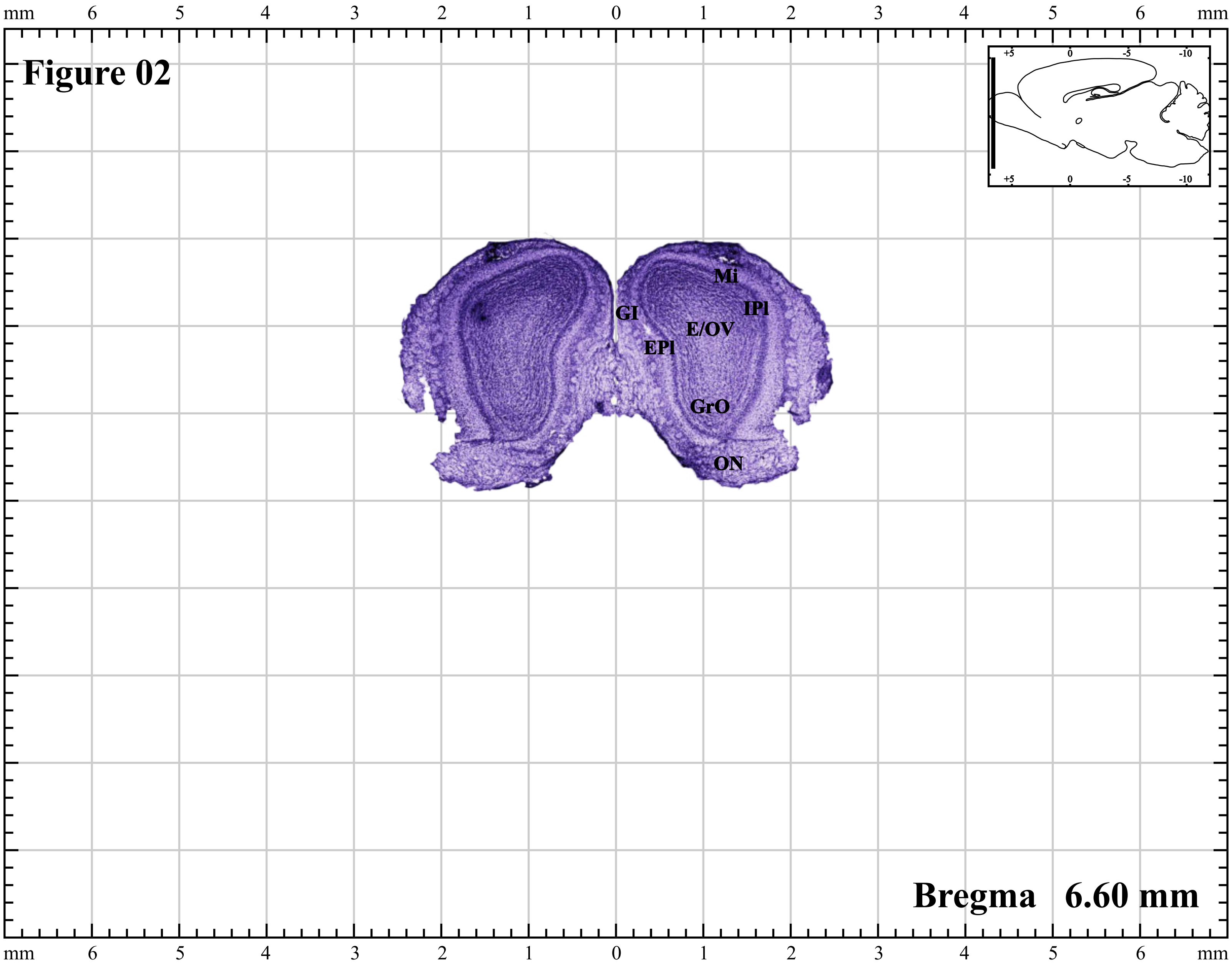

Bregma 6.60 mm

E/OV ependymal and subendymal layer/olfactory ventricle
EPl external plexiform layer of the olfactory bulb
GrO granular cell layer of the olfactory bulb
GI granular insular cortex
IPl internal plexiform layer of the olfactory bulb
Mi mitral cell layer of the olfactory bulb
ON olfactory nerve layer

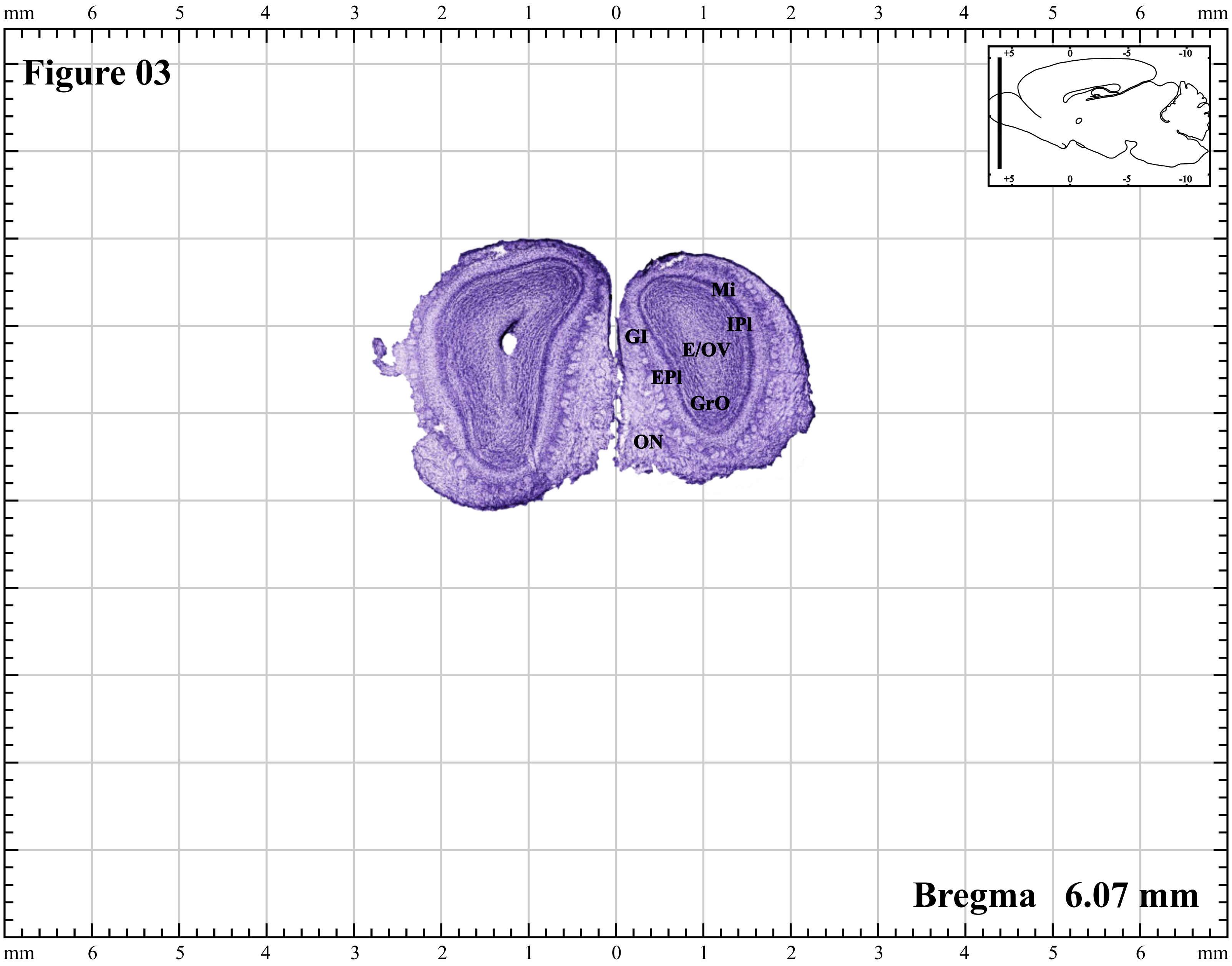

E/OV ependymal and subendymal layer/olfactory ventricle
EPl external plexiform layer of the olfactory bulb
GrO granular cell layer of the olfactory bulb
GI granular insular cortex
IPl internal plexiform layer of the olfactory bulb
Mi mitral cell layer of the olfactory bulb
ON olfactory nerve layer

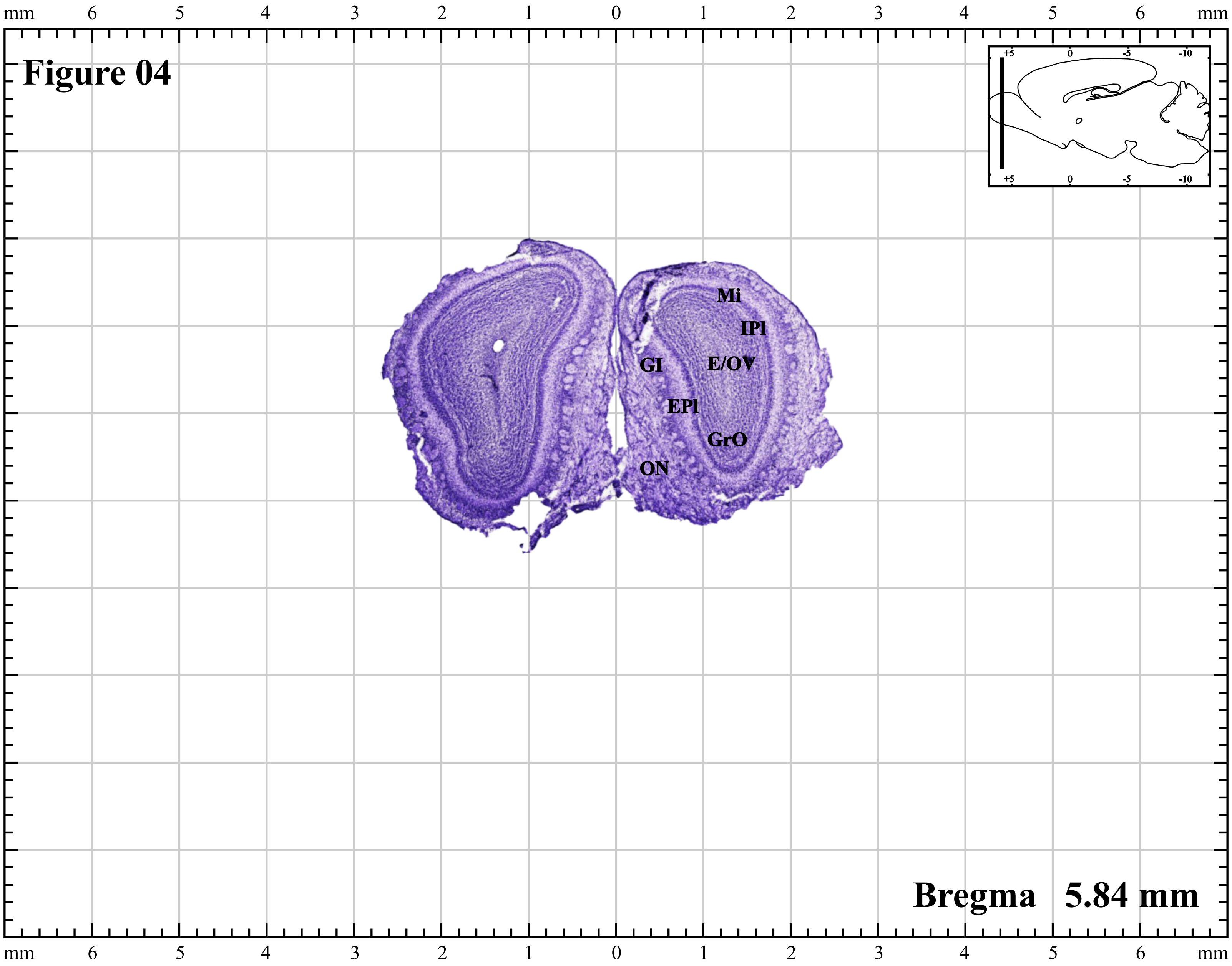

E/OV ependymal and subendymal layer/olfactory ventricle

EPl external plexiform layer of the olfactory bulb

GrO granular cell layer of the olfactory bulb

GI granular insular cortex

IPl internal plexiform layer of the olfactory bulb

Mi mitral cell layer of the olfactory bulb

ON olfactory nerve layer

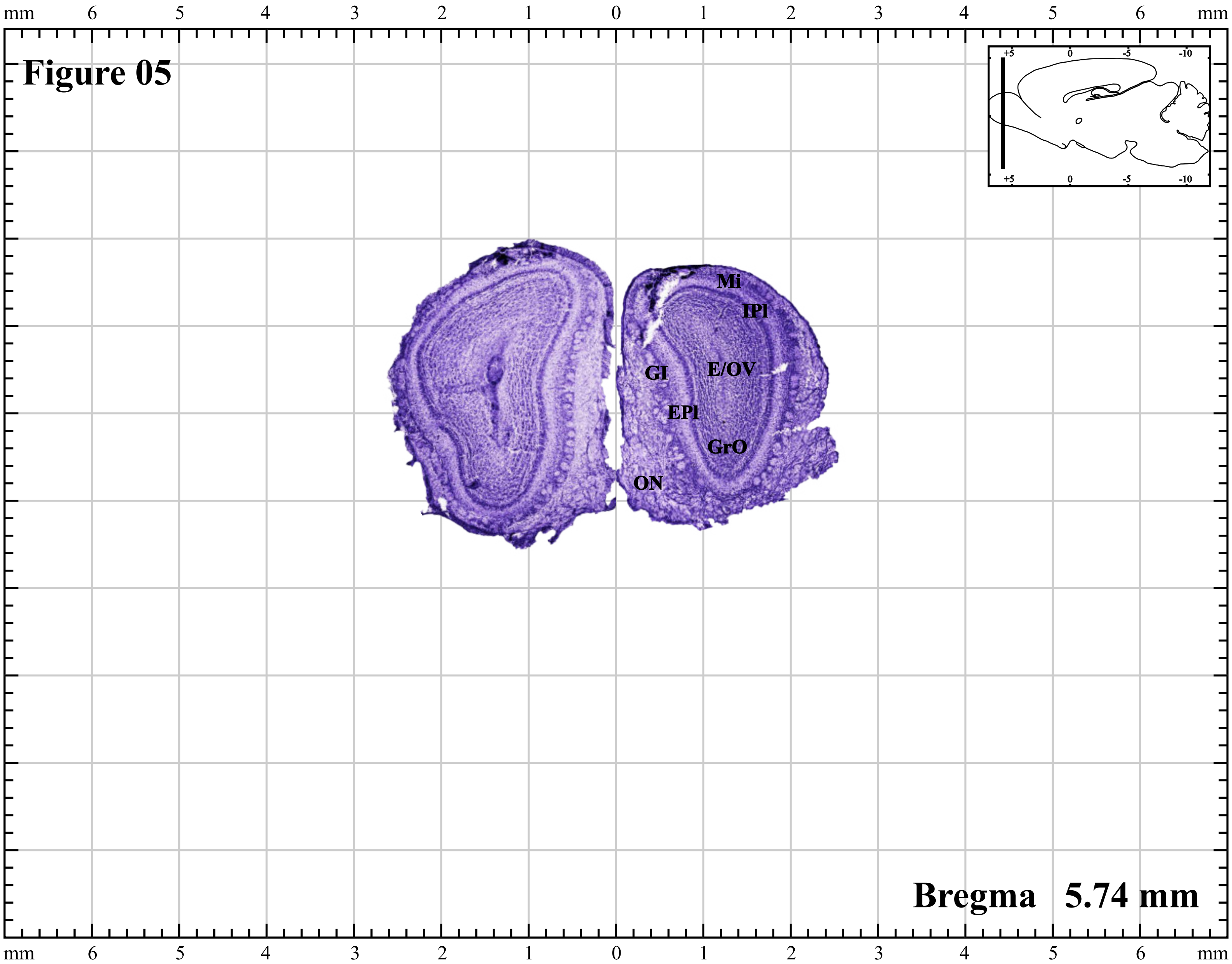

E/OV ependymal and subendymal layer/olfactory ventricle
EPl external plexiform layer of the olfactory bulb
GrO granular cell layer of the olfactory bulb
GI granular insular cortex
IPl internal plexiform layer of the olfactory bulb
Mi mitral cell layer of the olfactory bulb
ON olfactory nerve layer

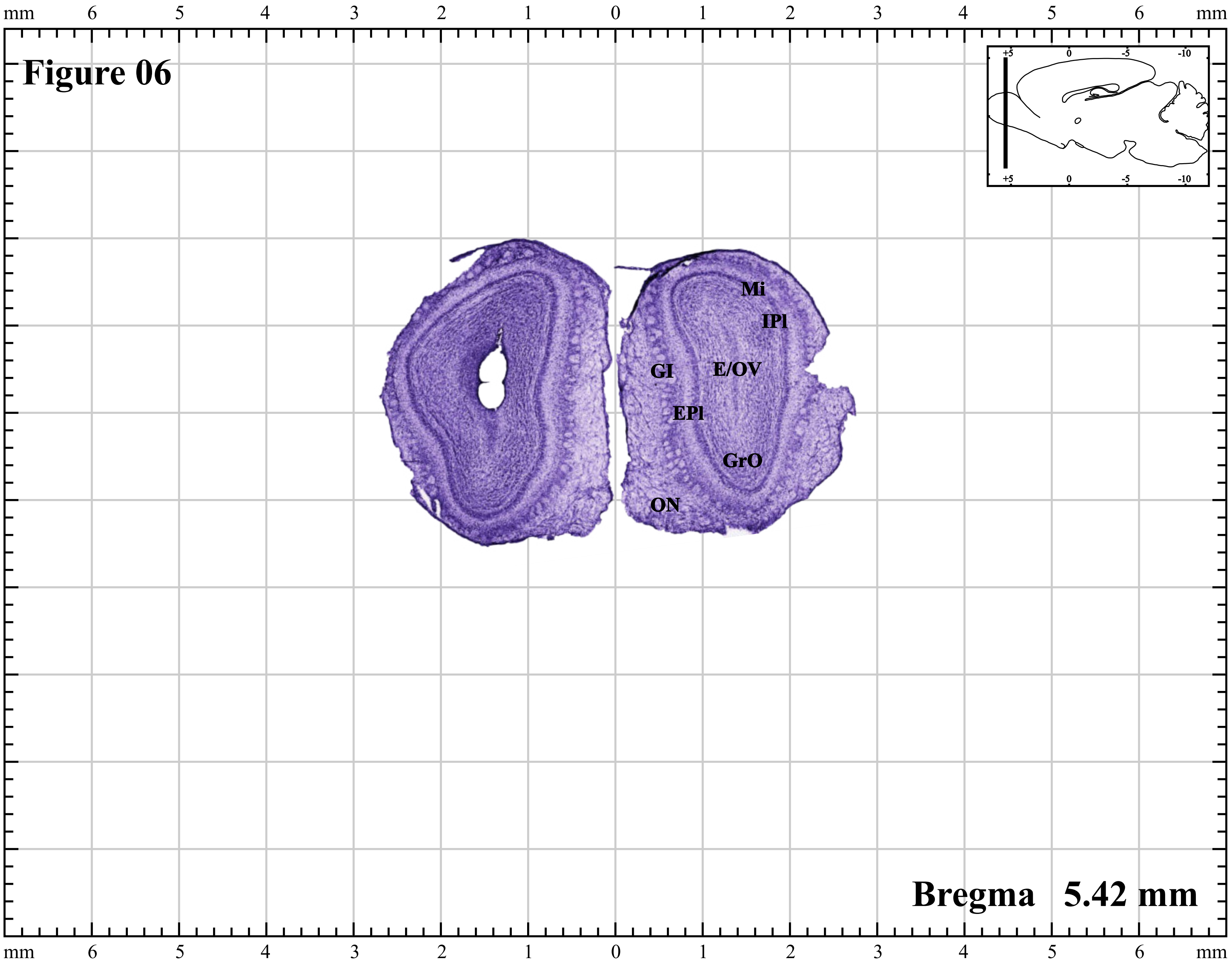

E/OV ependymal and subendymal layer/olfactory ventricle

EPl external plexiform layer of the olfactory bulb

GrO granular cell layer of the olfactory bulb

GI granular insular cortex

IPl internal plexiform layer of the olfactory bulb

Mi mitral cell layer of the olfactory bulb

ON olfactory nerve layer

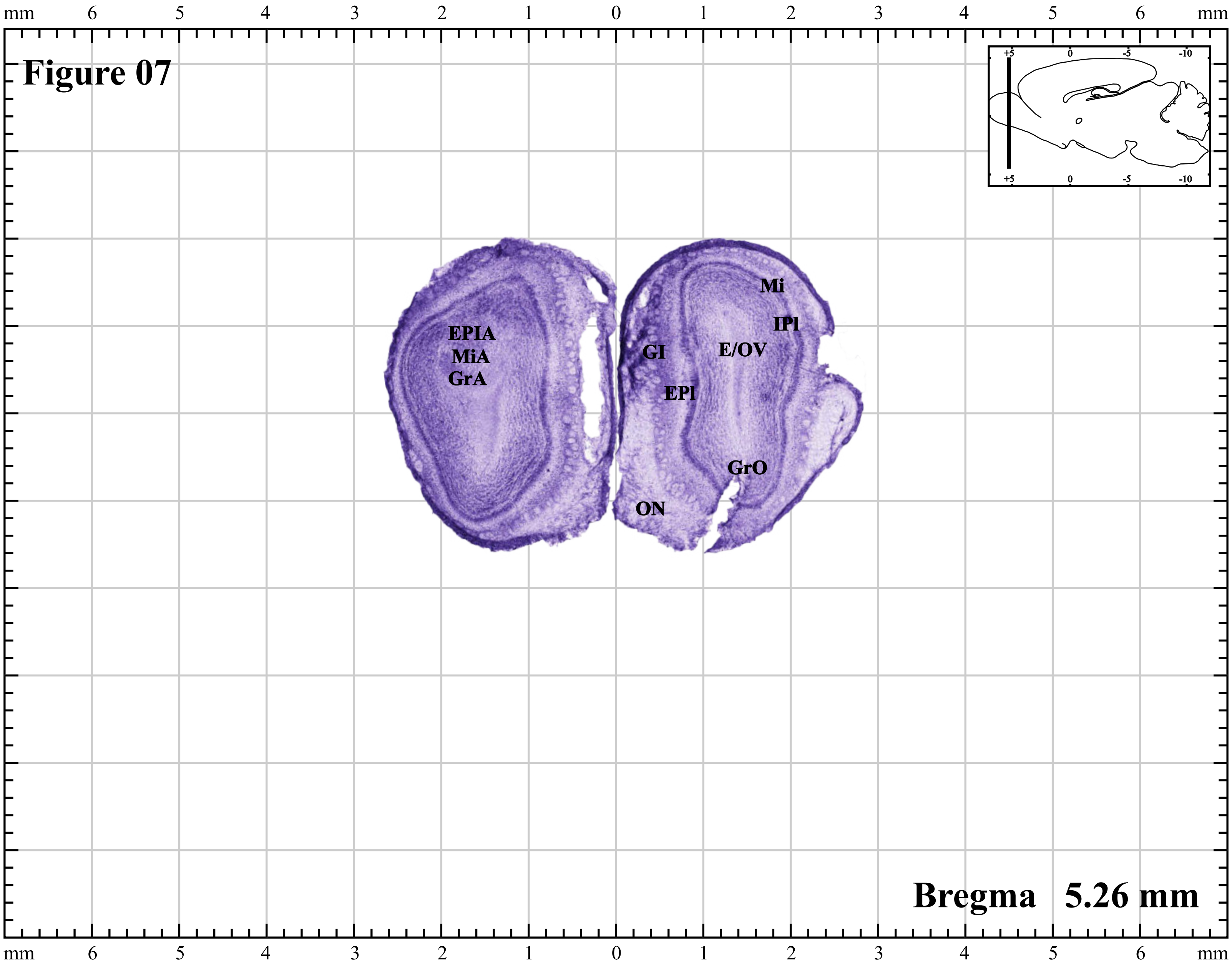

EPl external plexiform layer of the olfactory bulb
E/OV ependymal and subendymal layer/olfactory ventricle
EPlA external plexiform layer of the accessory olfactory bulb
GrO granular cell layer of the olfactory bulb
GrA granule cell layer of the accessory olfactory bulb
GI granular insular cortex
IPl internal plexiform layer of the olfactory bulb
Mi mitral cell layer of the olfactory bulb
MiA mitral cell layer of the accessory olfactory bulb
ON olfactory nerve layer

Figure 08

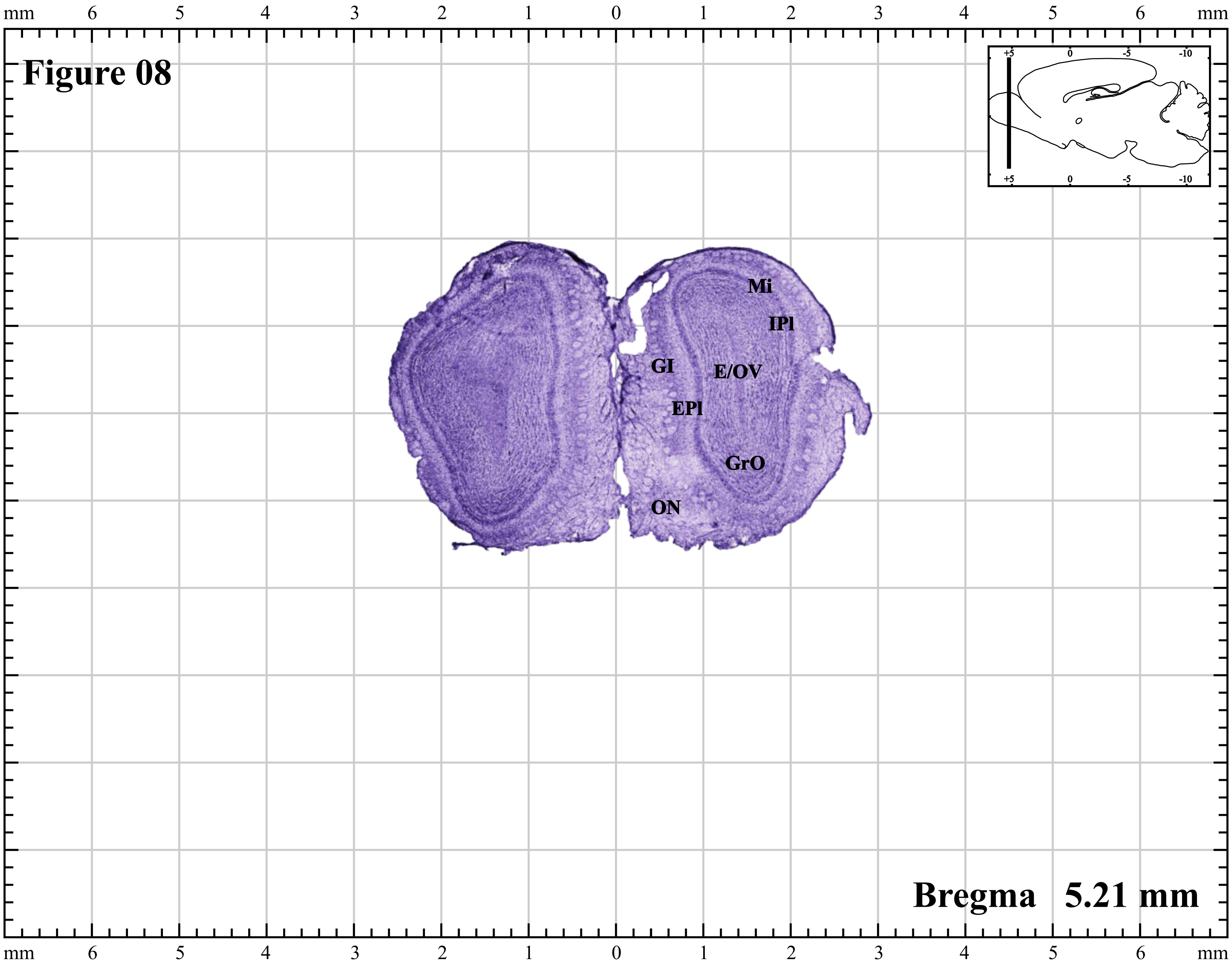

Bregma 5.21 mm

E/OV ependymal and subendymal layer/olfactory ventricle
EPl external plexiform layer of the olfactory bulb
GrO granular cell layer of the olfactory bulb
GI granular insular cortex
IPl internal plexiform layer of the olfactory bulb
Mi mitral cell layer of the olfactory bulb
ON olfactory nerve layer

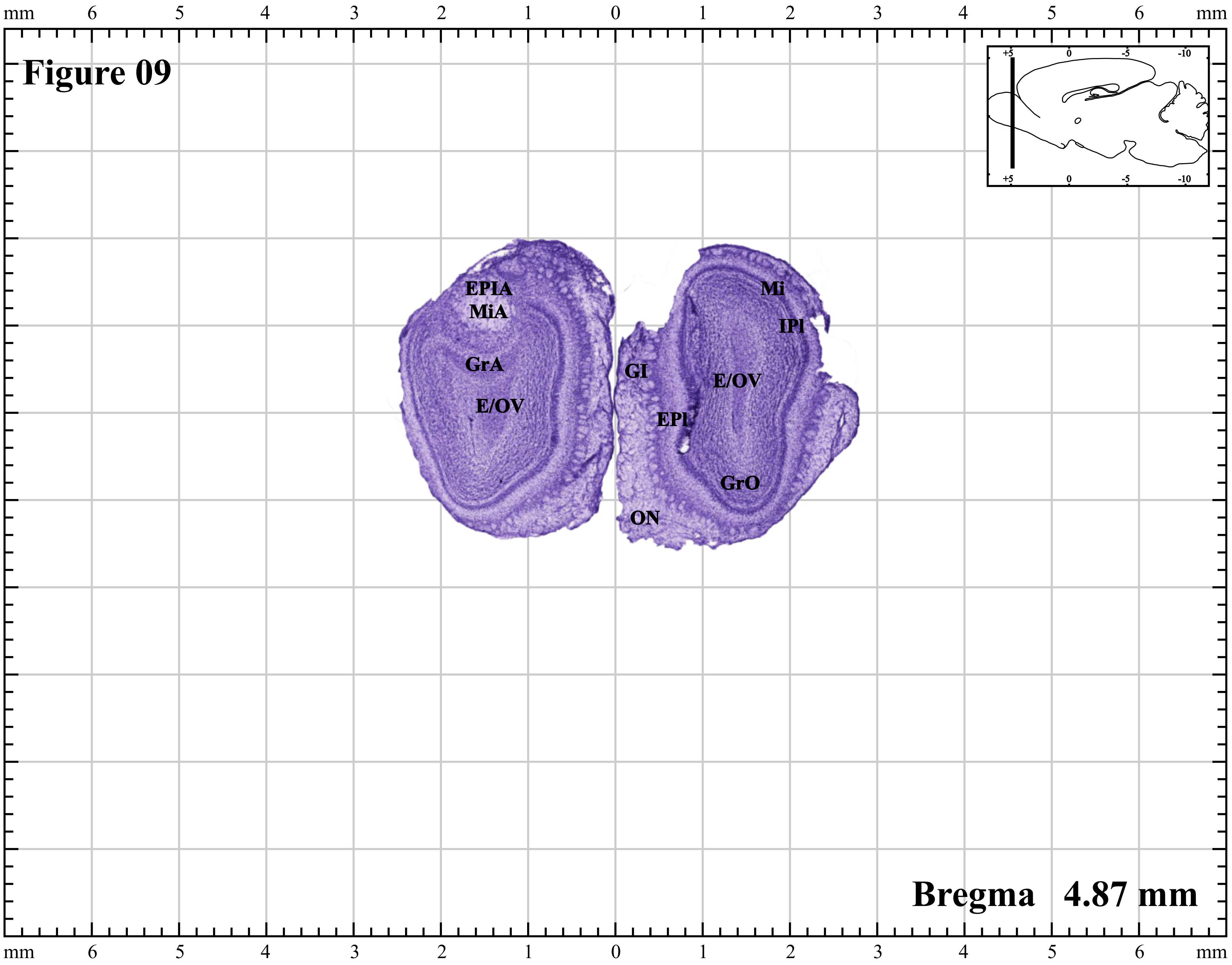

EPl external plexiform layer of the olfactory bulb
E/OV ependymal and subendymal layer/olfactory ventricle
EPlA external plexiform layer of the accessory olfactory bulb
FrA frontal assocn cortex
GI granular insular cortex
GlA glomerular layer of the accessory olfactory bulb
GrO granular cell layer of the olfactory bulb
GrA granule cell layer of the accessory olfactory bulb
IPl internal plexiform layer of the olfactory bulb
MiA mitral cell layer of the accessory olfactory bulb
Mi mitral cell layer of the olfactory bulb
ON olfactory nerve layer

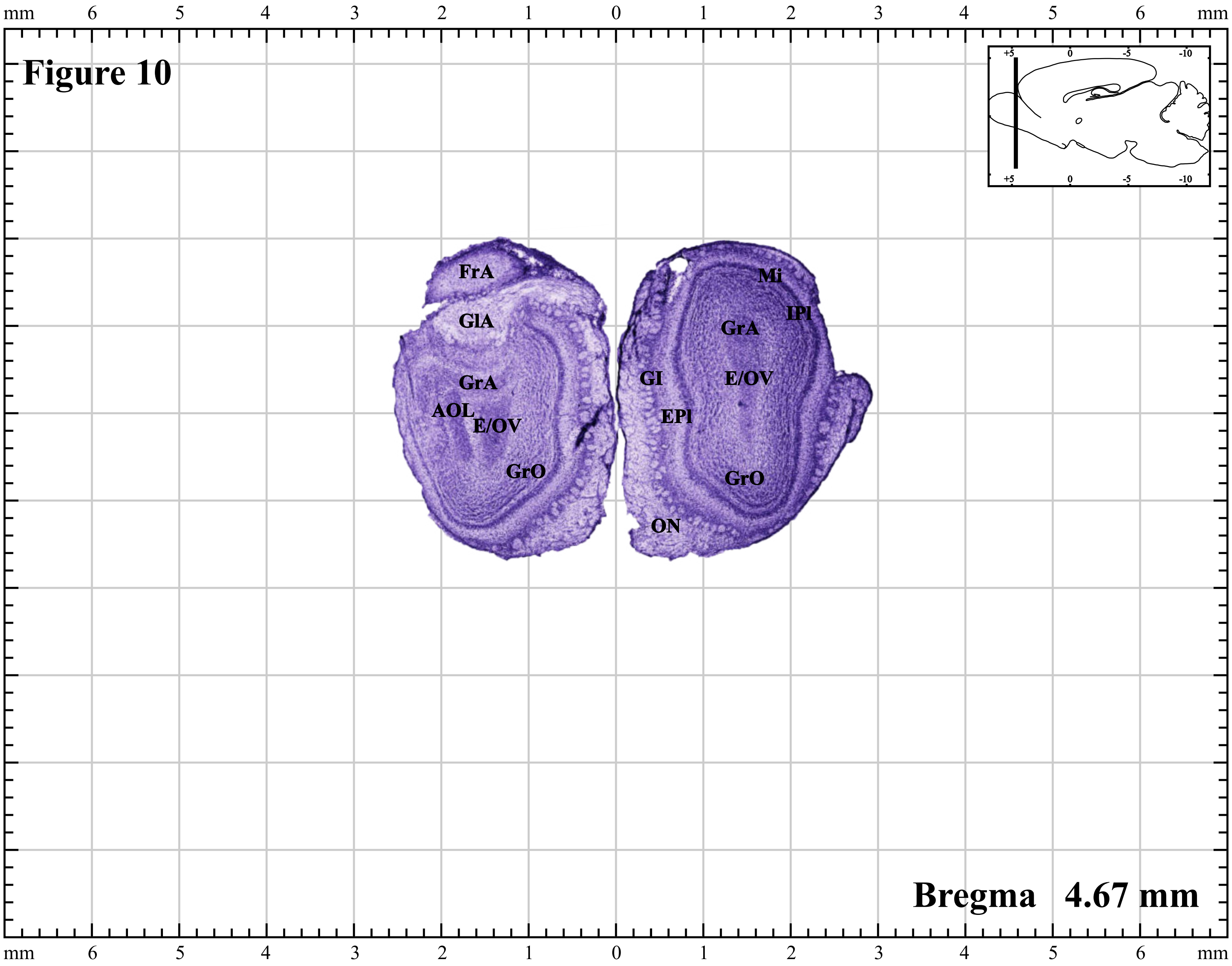

AOL anterior olfactory nucleus, lateral part
E/OV ependymal and subendymal layer/olfactory ventricle
EPl external plexiform layer of the olfactory bulb
FrA frontal assocn cortex
GlA glomerular layer of the accessory olfactory bulb
GrA granule cell layer of the accessory olfactory bulb
GrO granular cell layer of the olfactory bulb
GI granular insular cortex
IPl internal plexiform layer of the olfactory bulb
Mi mitral cell layer of the olfactory bulb
ON olfactory nerve layer

Figure 11

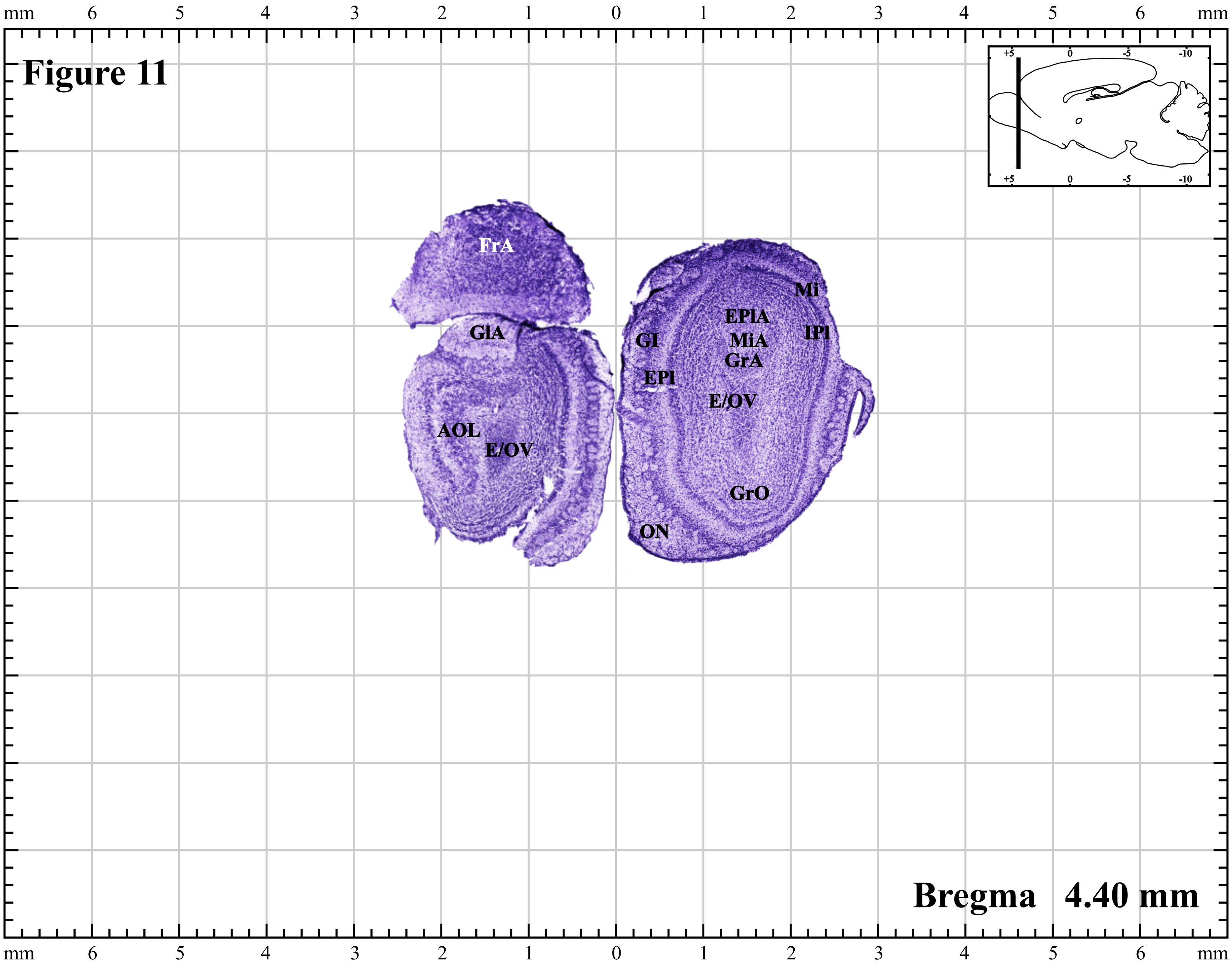

AOL anterior olfactory nucleus, lateral part
EPl external plexiform layer of the olfactory bulb
E/OV ependymal and subendymal layer/olfactory ventricle
EPlA external plexiform layer of the accessory olfactory bulb
FrA frontal assocn cortex
GlA glomerular layer of the accessory olfactory bulb
GrO granular cell layer of the olfactory bulb
GI granular insular cortex
GrA granule cell layer of the accessory olfactory bulb
IPl internal plexiform layer of the olfactory bulb
Mi mitral cell layer of the olfactory bulb
MiA mitral cell layer of the accessory olfactory bulb
ON olfactory nerve layer

Figure 12

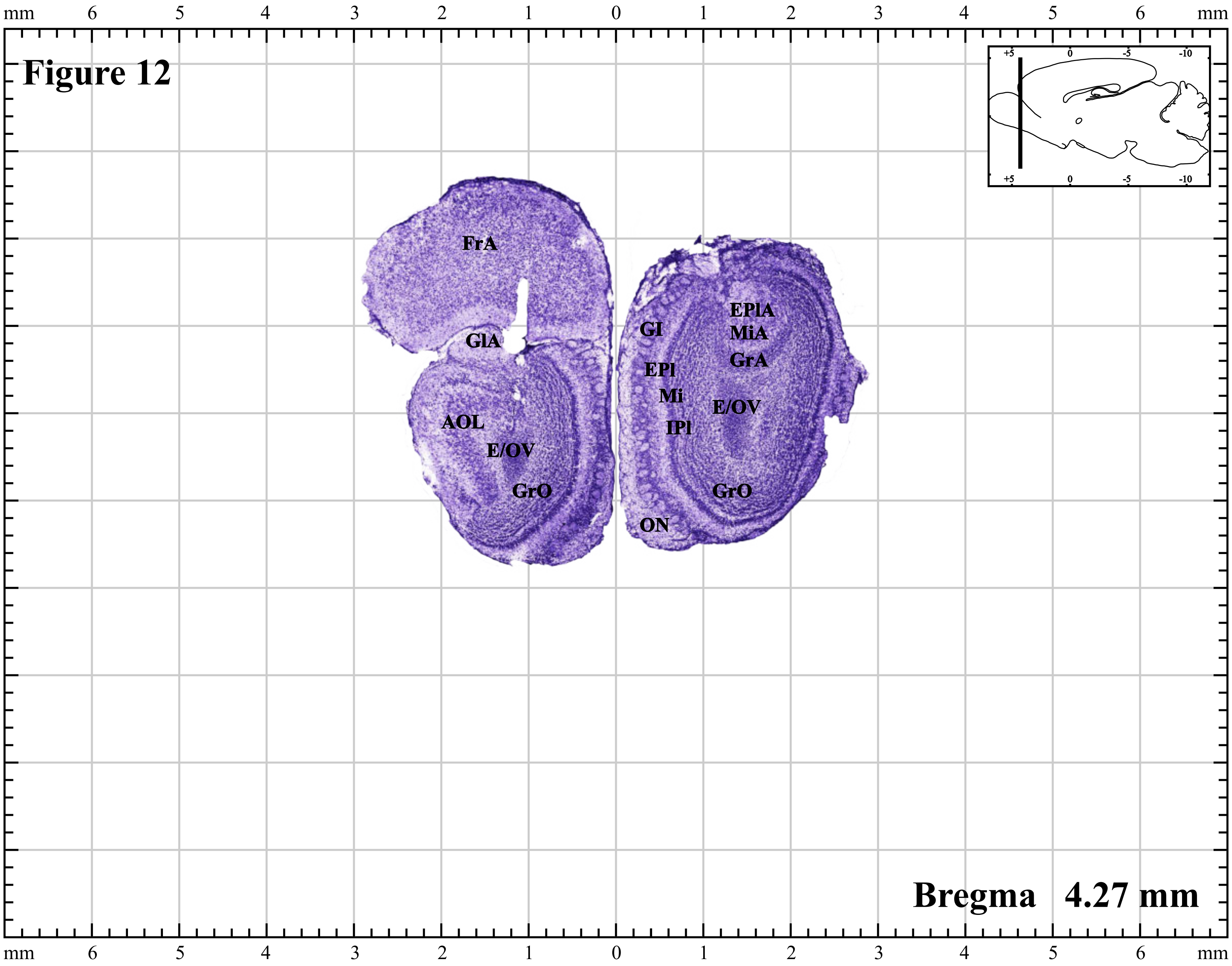

Bregma 4.27 mm

AOL anterior olfactory nucleus, lateral part
EPl external plexiform layer of the olfactory bulb
E/OV ependymal and subendymal layer/olfactory ventricle
EPlA external plexiform layer of the accessory olfactory bulb
FrA frontal assocn cortex
GlA glomerular layer of the accessory olfactory bulb
GrO granular cell layer of the olfactory bulb
GI granular insular cortex
GrA granule cell layer of the accessory olfactory bulb
IPl internal plexiform layer of the olfactory bulb
Mi mitral cell layer of the olfactory bulb
MiA mitral cell layer of the accessory olfactory bulb
ON olfactory nerve layer

Figure 13

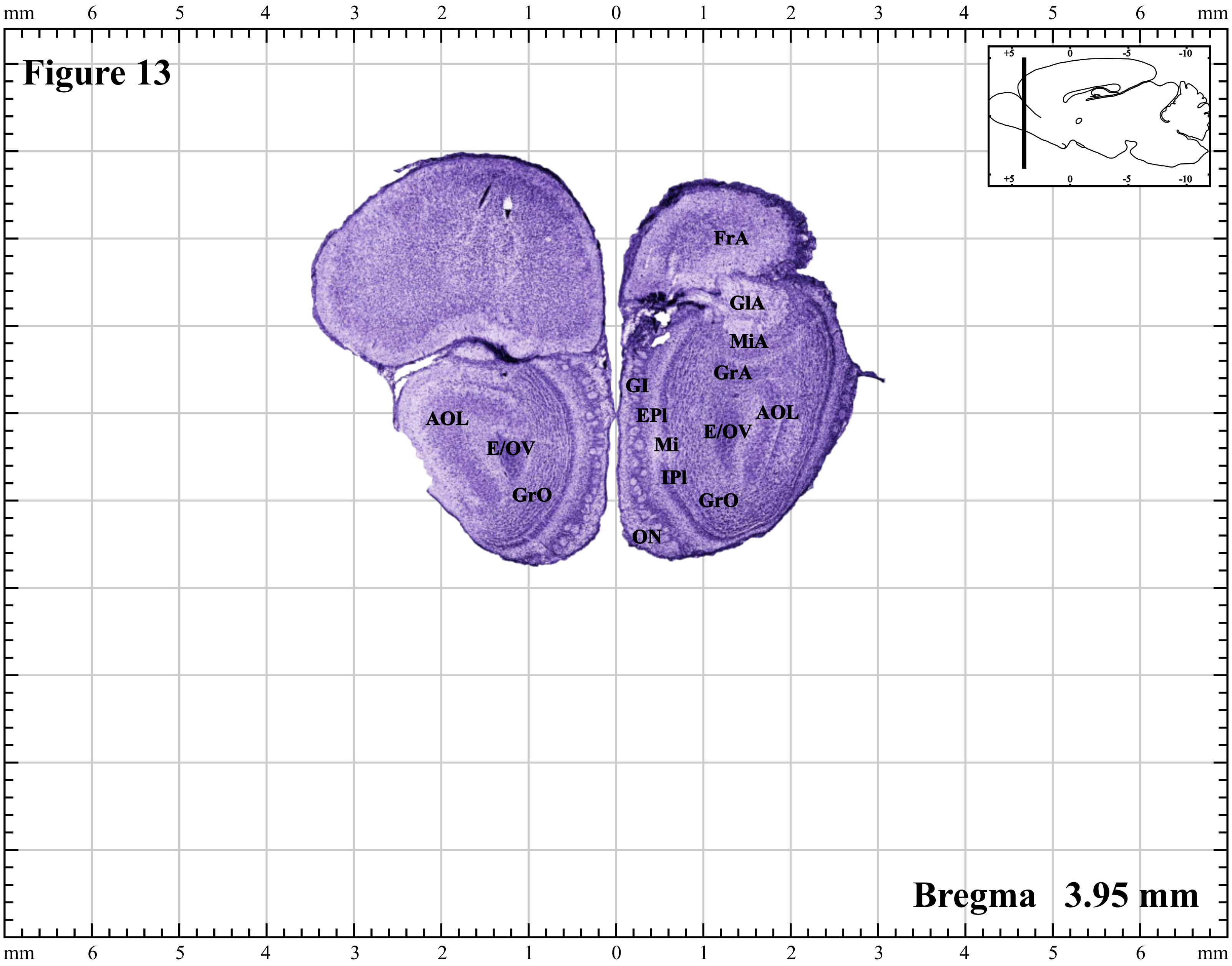

AOL anterior olfactory nucleus, lateral part
EPl external plexiform layer of the olfactory bulb
E/OV ependymal and subendymal layer/olfactory ventricle
FrA frontal assocn cortex
GrA granule cell layer of the accessory olfactory bulb
GlA glomerular layer of the accessory olfactory bulb
GrO granular cell layer of the olfactory bulb
GI granular insular cortex
IPl internal plexiform layer of the olfactory bulb
Mi mitral cell layer of the olfactory bulb
MiA mitral cell layer of the accessory olfactory bulb
ON olfactory nerve layer

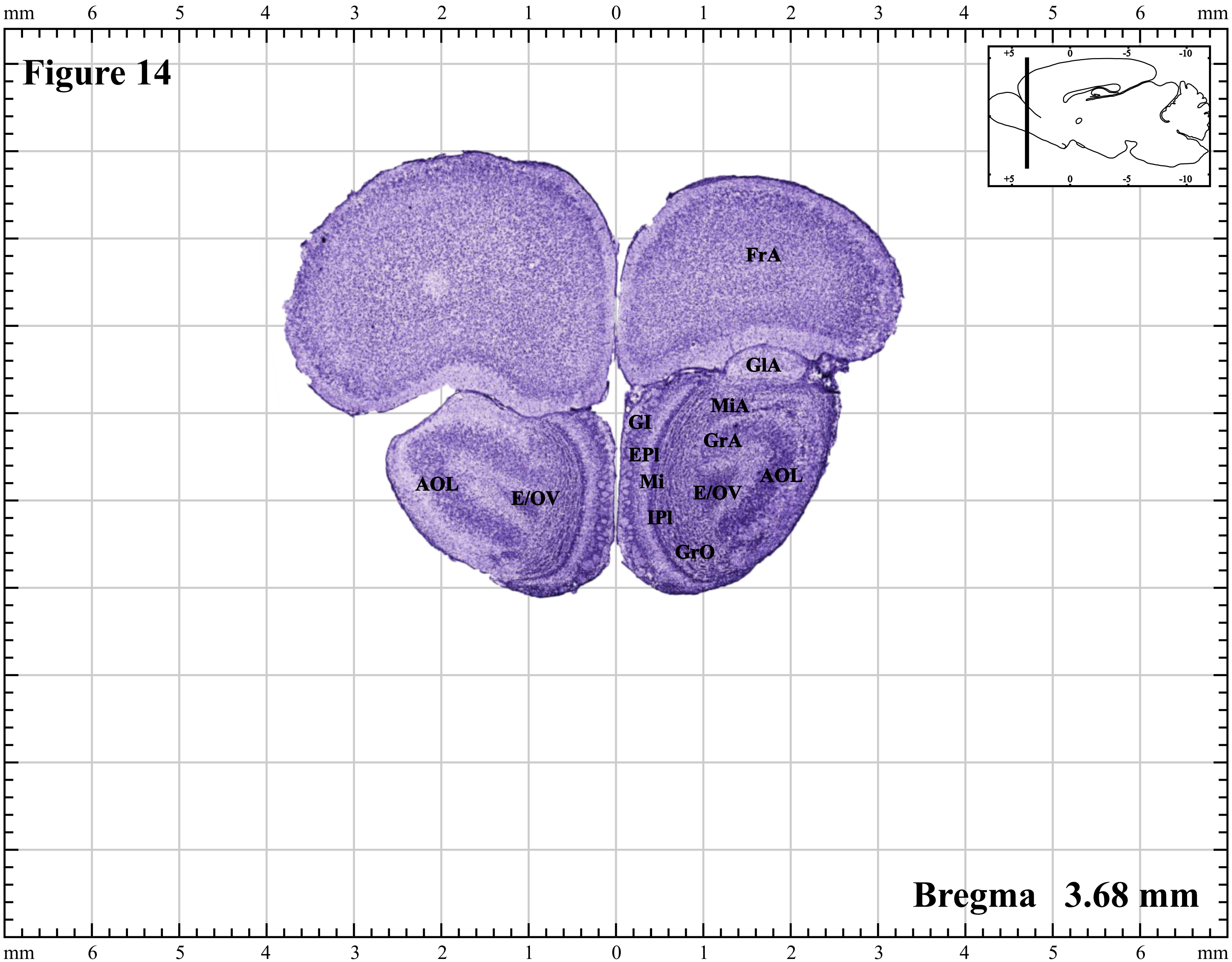

AOL anterior olfactory nucleus, lateral part
EPl external plexiform layer of the olfactory bulb
E/OV ependymal and subendymal layer/olfactory ventricle
FrA frontal assocn cortex
GrA granule cell layer of the accessory olfactory bulb
GlA glomerular layer of the accessory olfactory bulb
GrO granular cell layer of the olfactory bulb
GI granular insular cortex
IPl internal plexiform layer of the olfactory bulb
Mi mitral cell layer of the olfactory bulb
MiA mitral cell layer of the accessory olfactory bulb

Figure 15

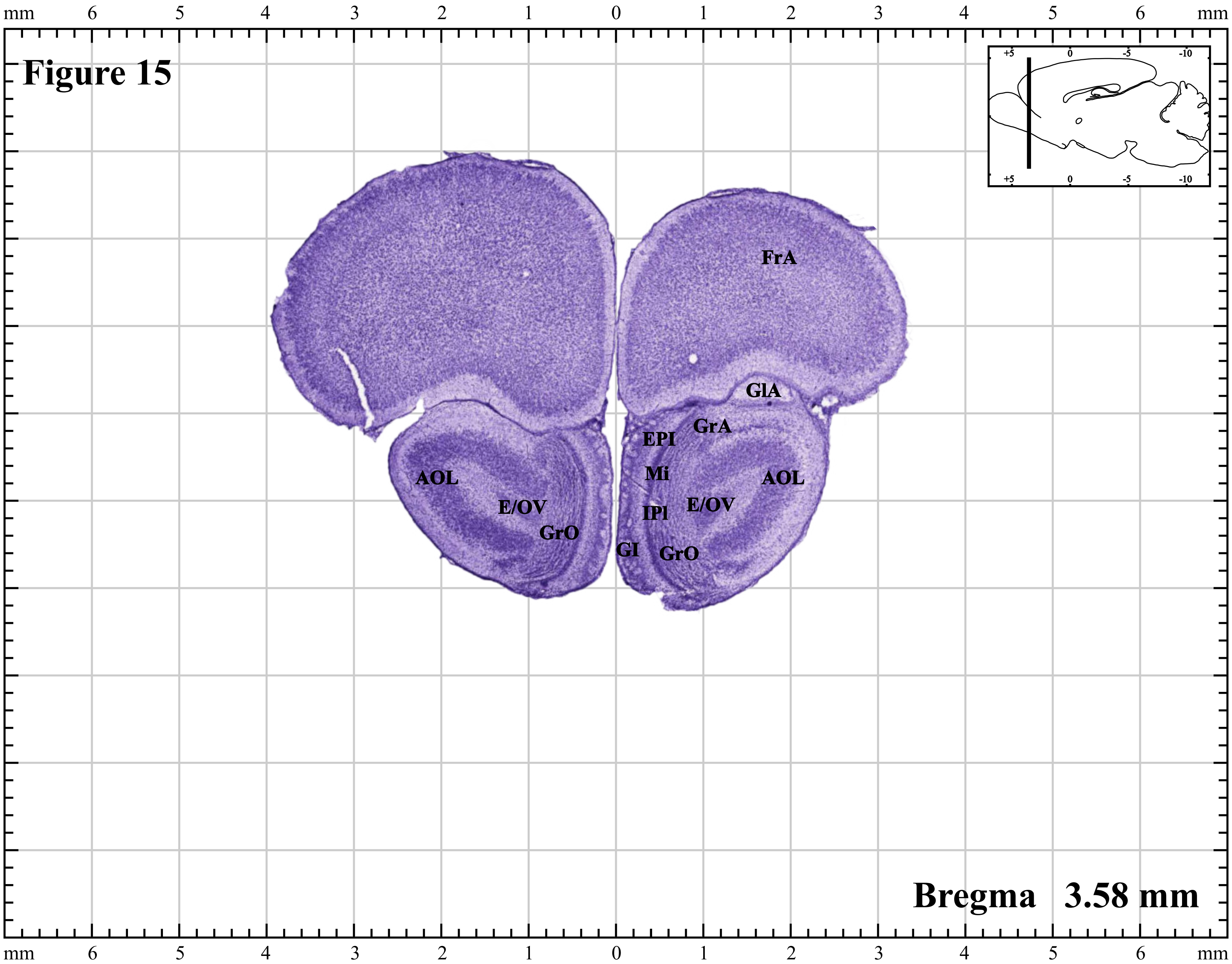

Bregma 3.58 mm

AOL anterior olfactory nucleus, lateral part
EPl external plexiform layer of the olfactory bulb
E/OV ependymal and subendymal layer/olfactory ventricle
FrA frontal assocn cortex
GrA granule cell layer of the accessory olfactory bulb
GlA glomerular layer of the accessory olfactory bulb
GrO granular cell layer of the olfactory bulb
GI granular insular cortex
IPl internal plexiform layer of the olfactory bulb
Mi mitral cell layer of the olfactory bulb
MiA mitral cell layer of the accessory olfactory bulb
ON olfactory nerve layer
VTT ventral tenia tecta

Figure 16

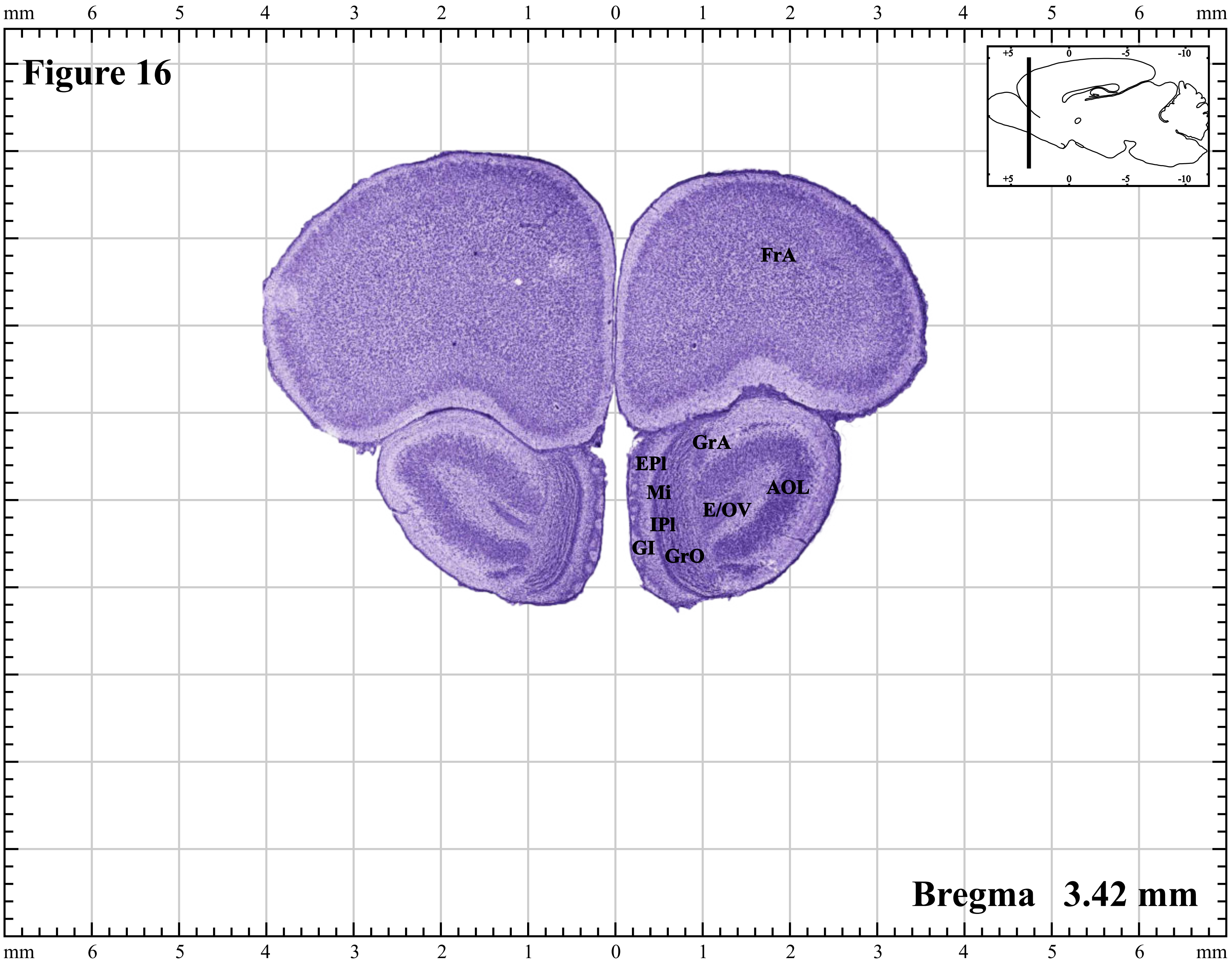

Bregma 3.42 mm

AOL anterior olfactory nucleus, lateral part
EPl external plexiform layer of the olfactory bulb
E/OV ependymal and subendymal layer/olfactory ventricle
FrA frontal assocn cortex
GrA granule cell layer of the accessory olfactory bulb
GrO granular cell layer of the olfactory bulb
GI granular insular cortex
IPl internal plexiform layer of the olfactory bulb
Mi mitral cell layer of the olfactory bulb
MiA mitral cell layer of the accessory olfactory bulb
ON olfactory nerve layer
VTT ventral tenia tecta

Figure 17

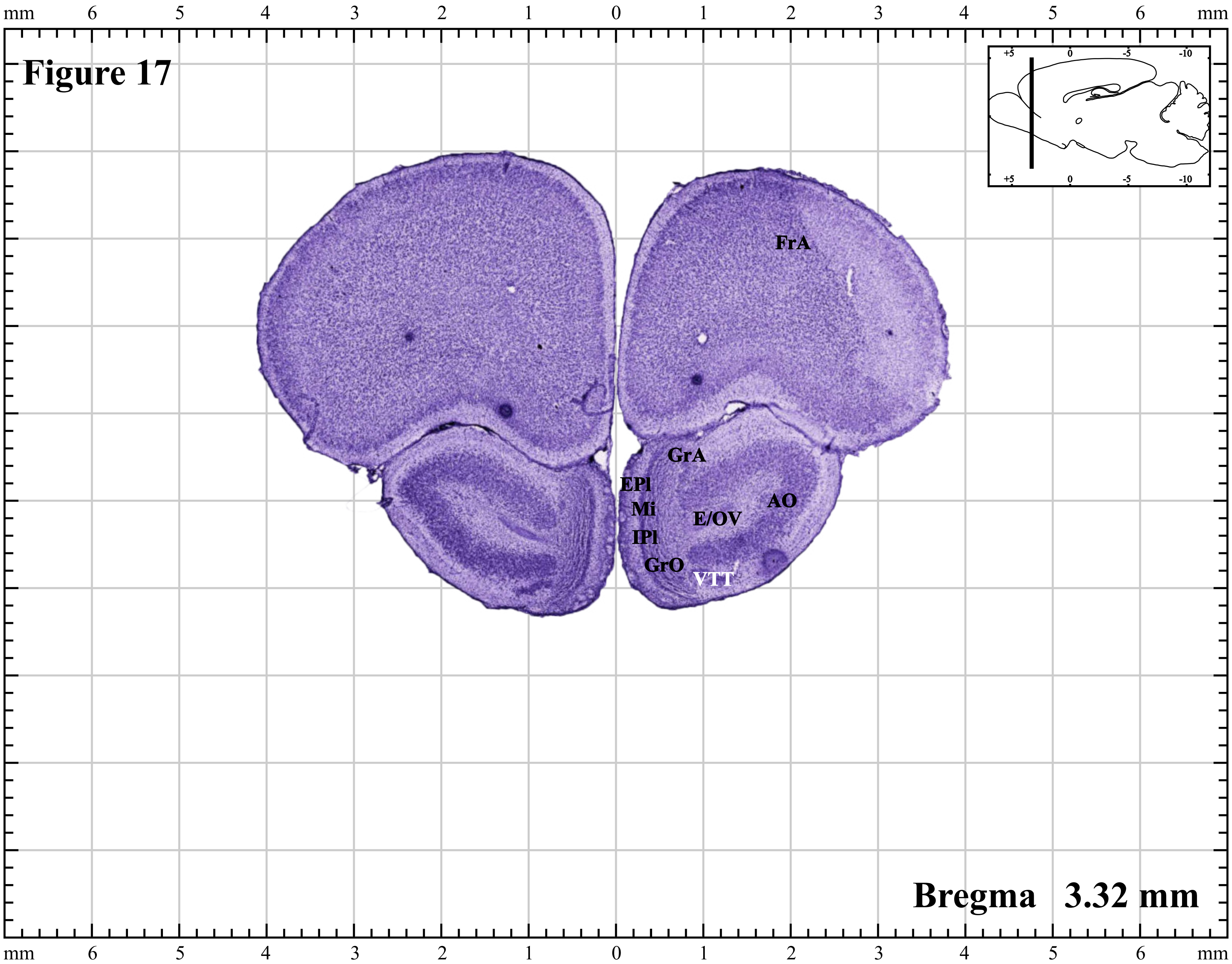

Bregma 3.32 mm

AO anterior olfactory nucleus
EPl external plexiform layer of the olfactory bulb
E/OV ependymal and subendymal layer/olfactory ventricle
FrA frontal assocn cortex
GrA granule cell layer of the accessory olfactory bulb
GrO granular cell layer of the olfactory bulb
IPl internal plexiform layer of the olfactory bulb
Mi mitral cell layer of the olfactory bulb
VTT ventral tenia tecta

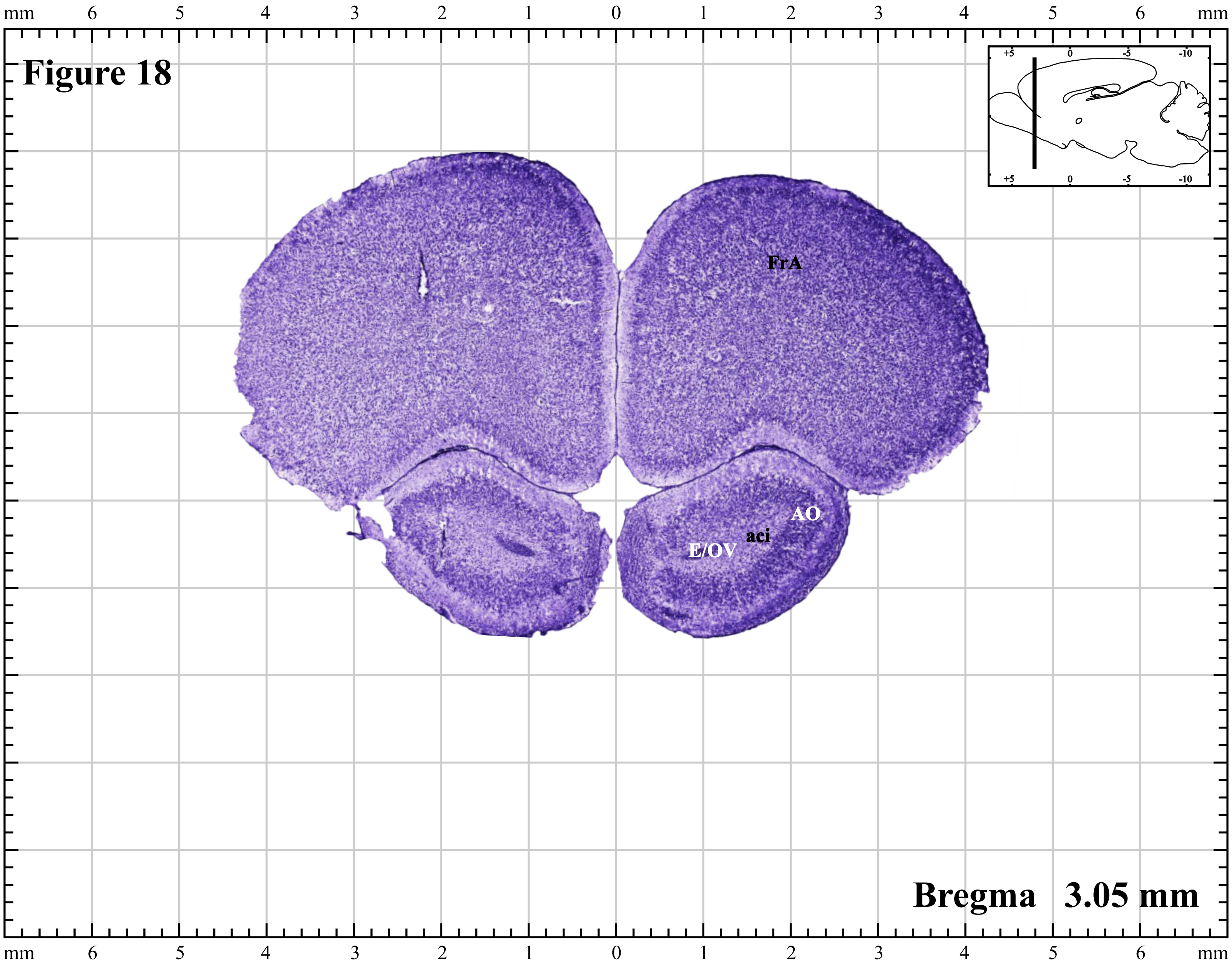

aci anterior commissure, intrabulbar part
AO anterior olfactory nuclues
E/OV ependymal and subendymal layer /olfactory ventricle
FrA frontal assocn cortex

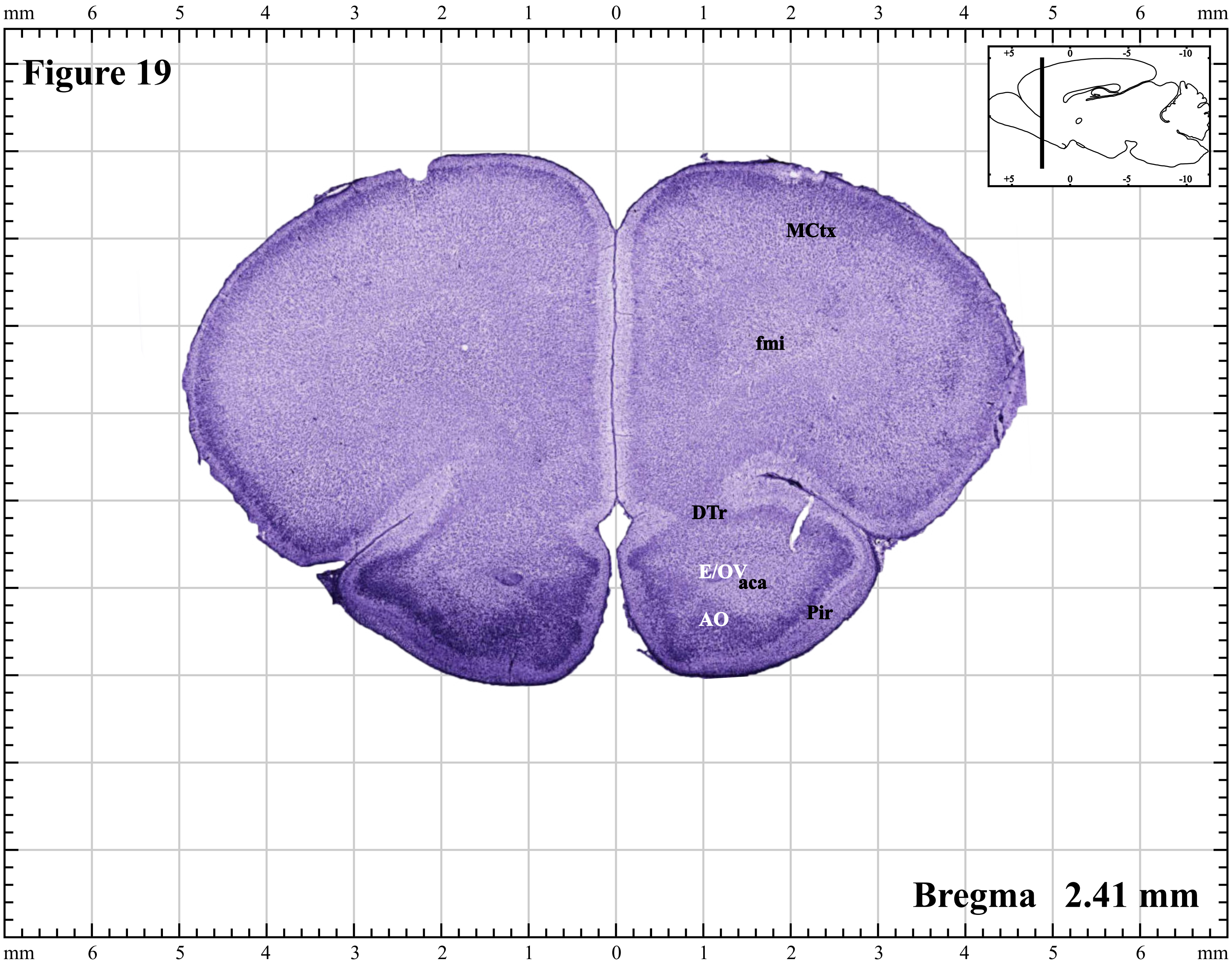

aca anterior commissure, anterior part
AO anterior olfactory nuclues
DTr dorsal transition zone
E/OV ependyma and subependymal layer /olfactory ventricle
fmi forceps major of corpus callosum
MCtx motor cortex
Pir piriform cortex

Figure 20

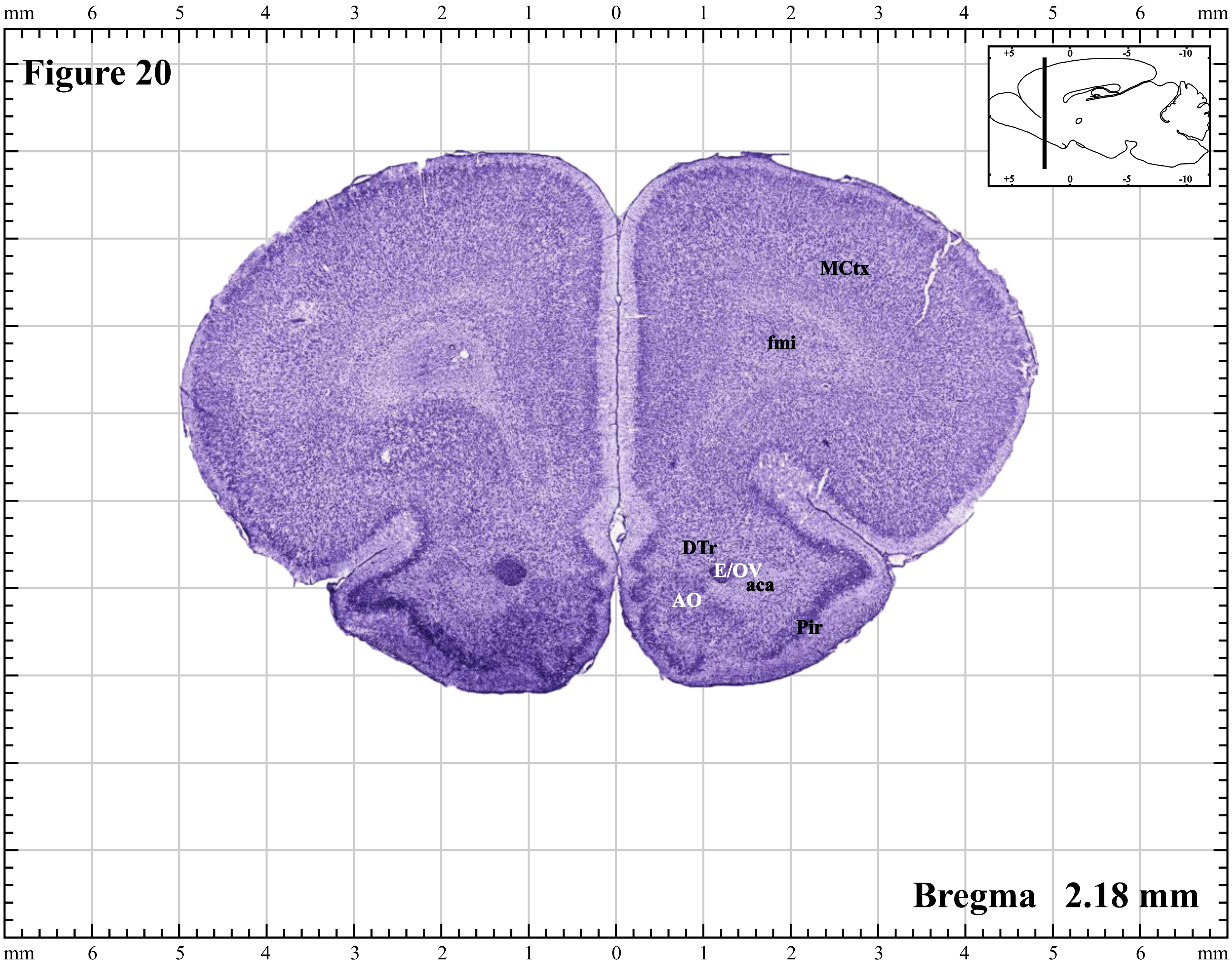

aca anterior commissure, anterior part
AO anterior olfactory nuclues
DTr dorsal transition zone
E/OV ependyma and subependymal layer
/olfactory ventricle
fmi forceps major of corpus callosum
MCtx motor cortex
Pir piriform cortex

Figure 21

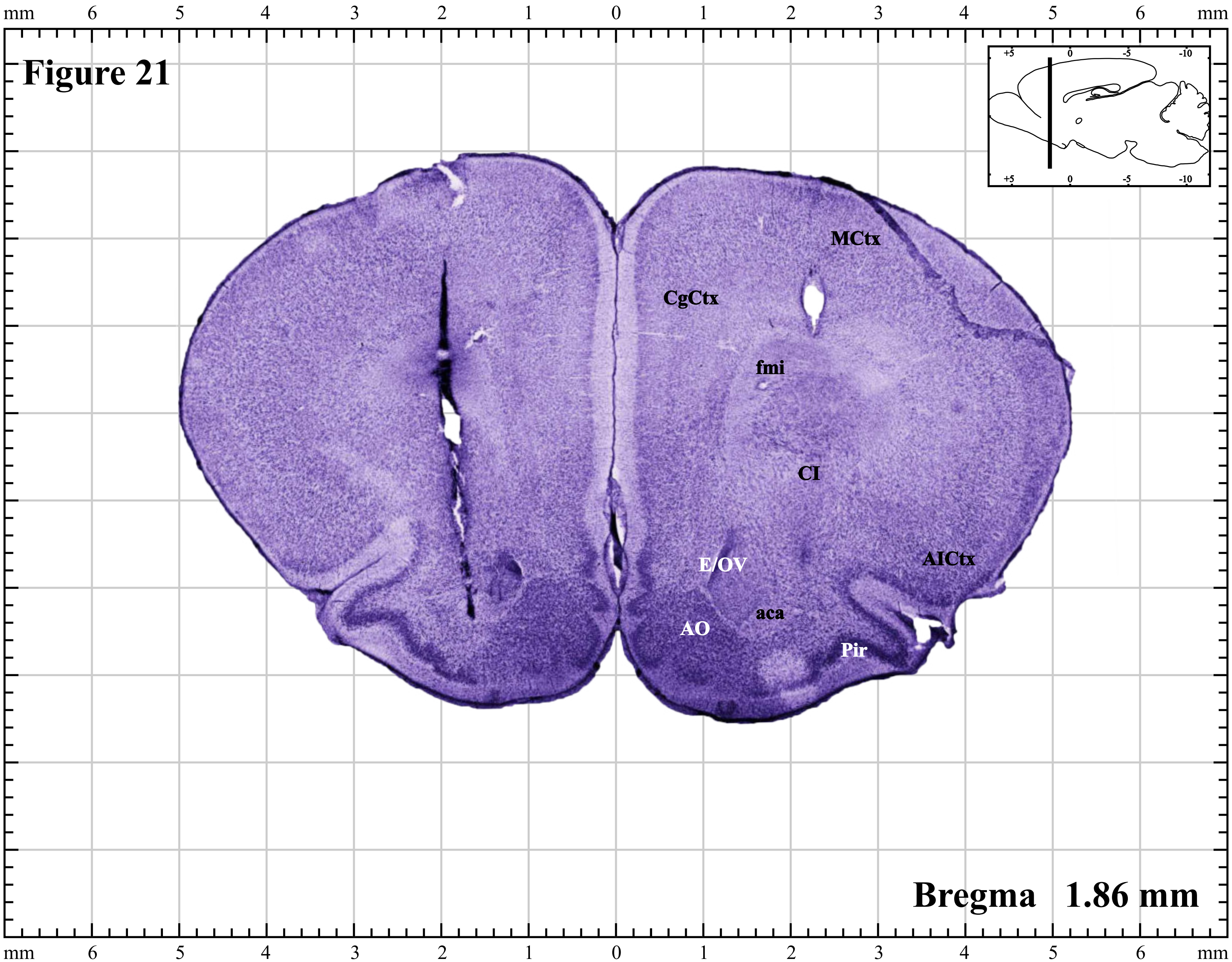

Bregma 1.86 mm

aca anterior commissure, anterior part
AO anterior olfactory nuclues
AICtx agranular insular cortex
CgCtx cingulate cortex
CI claustrum
E/OV ependyma and subependymal layer /olfactory ventricle
fmi forceps major of corpus callosum
MCtx motor cortex
Pir piriform cortex

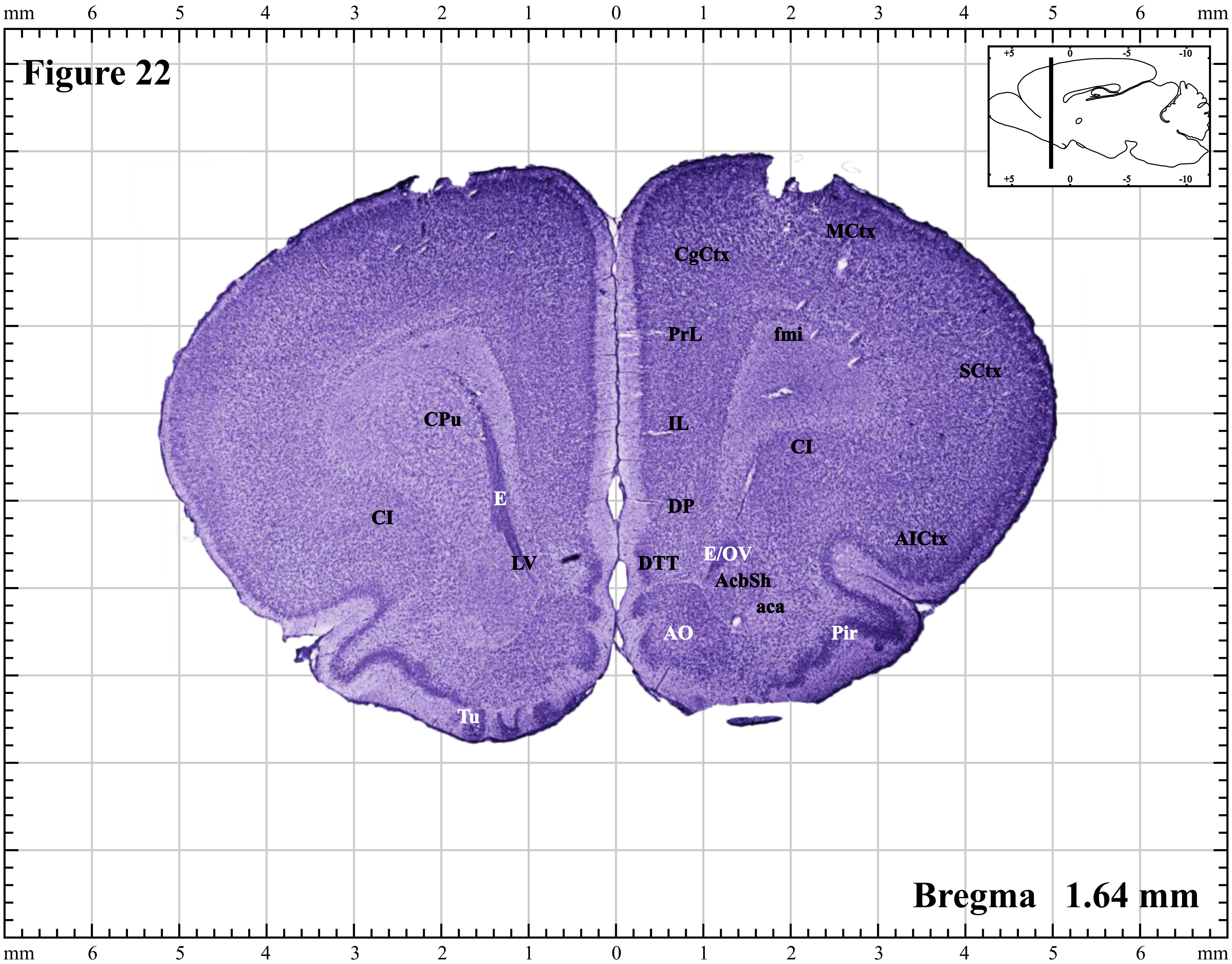

aca anterior commissure, anterior part
AcbSh accumbens shell
AO anterior olfactory nuclues
AICtx agranular insular cortex
CgCtx cingulate cortex
CI claustrum
CPu caudate putamen (striatum)
DTT dorsal tenia tecta
DP dorsal peduncular cortex
E/OV ependyma and subependymal layer /olfactory ventricle
fmi forceps major of corpus callosum
IL infralimbic cortex
LV lateral ventricle
MCtx motor cortex
Pir piriform cortex
PrL prelimbic cortex
SCtx somatosensory cortex
Tu olfactory tubercle

Figure 23

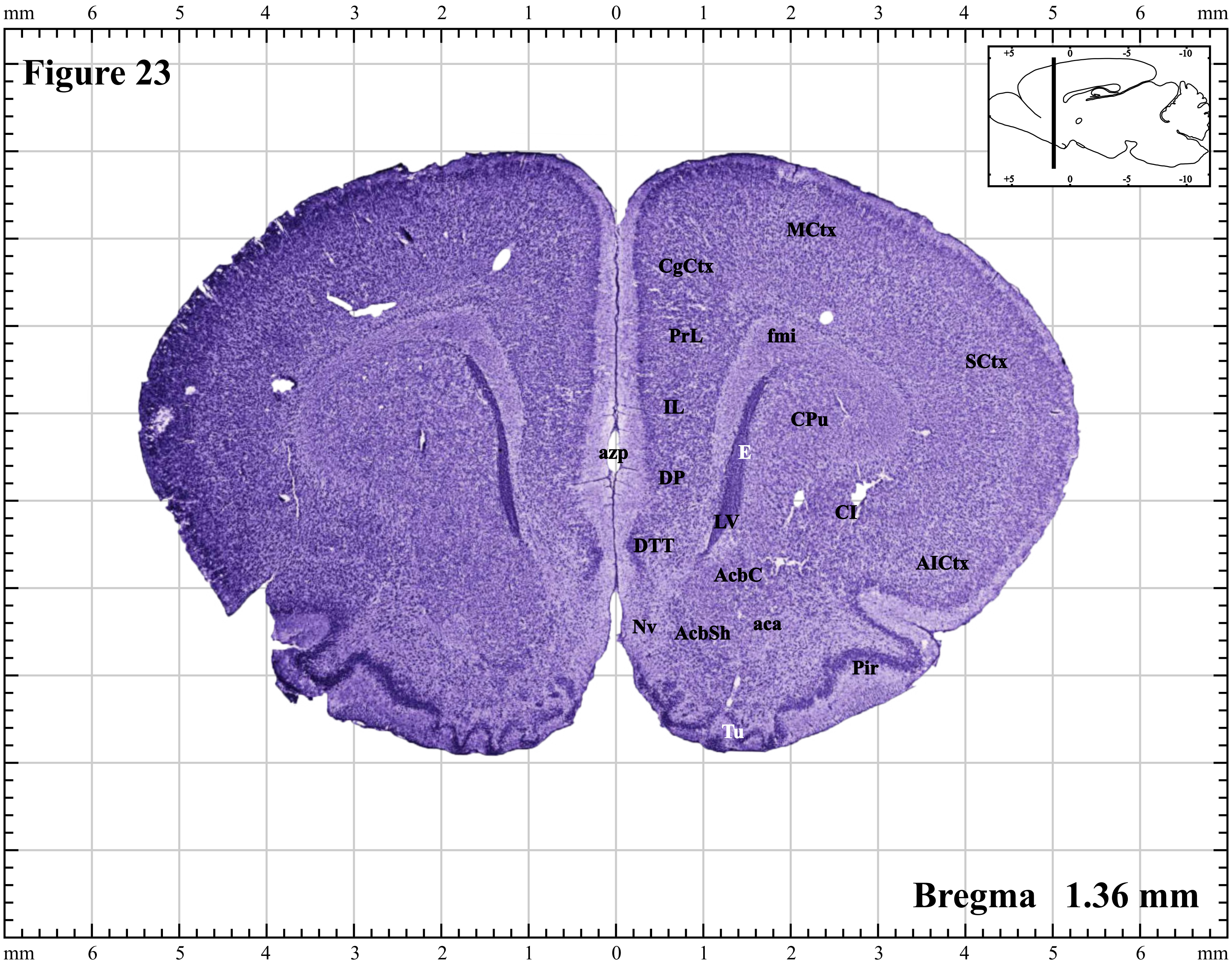

Bregma 1.36 mm

aca anterior commissure, anterior part
azp azygous pericallosal artery
AcbC accumbens nucleus, core
AcbSh accumbens shell
AICtx agranular insular cortex
CgCtx cingulate cortex
CI claustrum
CPu caudate putamen (striatum)
DP dorsal peduncular cortex
DTT dorsal tenia tecta
IL infralimbic cortex
E ependyma and subependymal layer
fmi forceps major of corpus callosum
LV lateral ventricle
MCtx motor cortex
Nv navicular nucleus of the basal forebrain
Pir piriform cortex
PrL prelimbic cortex
SCtx somatosensory cortex
Tu olfactory tubercle

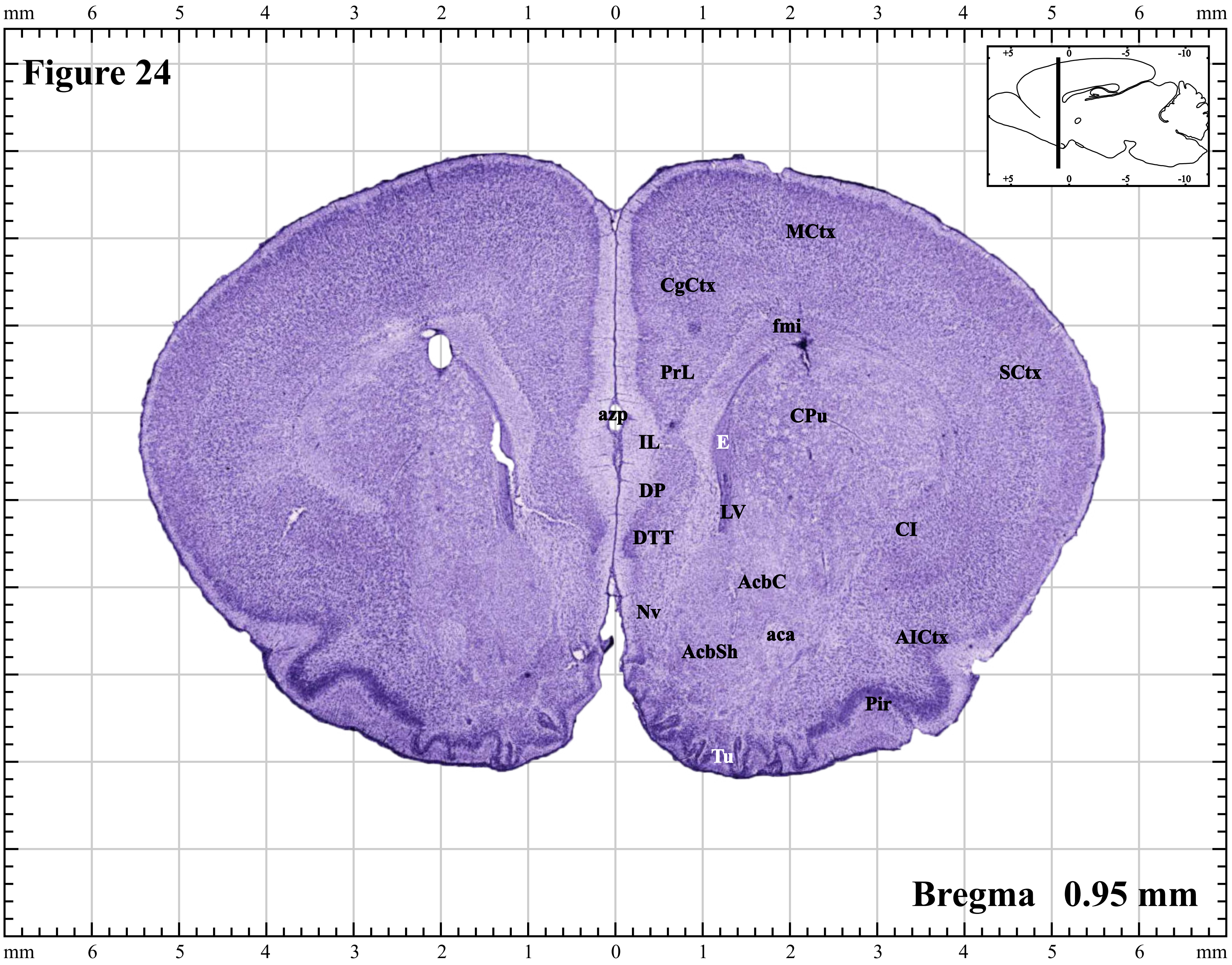

aca anterior commissure, anterior part
azp azygous pericallosal artery
AcbC accumbens nucleus, core
AcbSh accumbens shell
AICtx agranular insular cortex
CgCtx cingulate cortex
CI claustrum
CPu caudate putamen (striatum)
DP dorsal peduncular cortex
DTT dorsal tenia tecta
IL infralimbic cortex
E ependyma and subependymal layer
fmi forceps major of corpus callosum
LV lateral ventricle
MCtx motor cortex
Nv navicular nucleus of the basal forebrain
Pir piriform cortex
PrL prelimbic cortex
SCtx somatosensory cortex
Tu olfactory tubercle

Figure 25

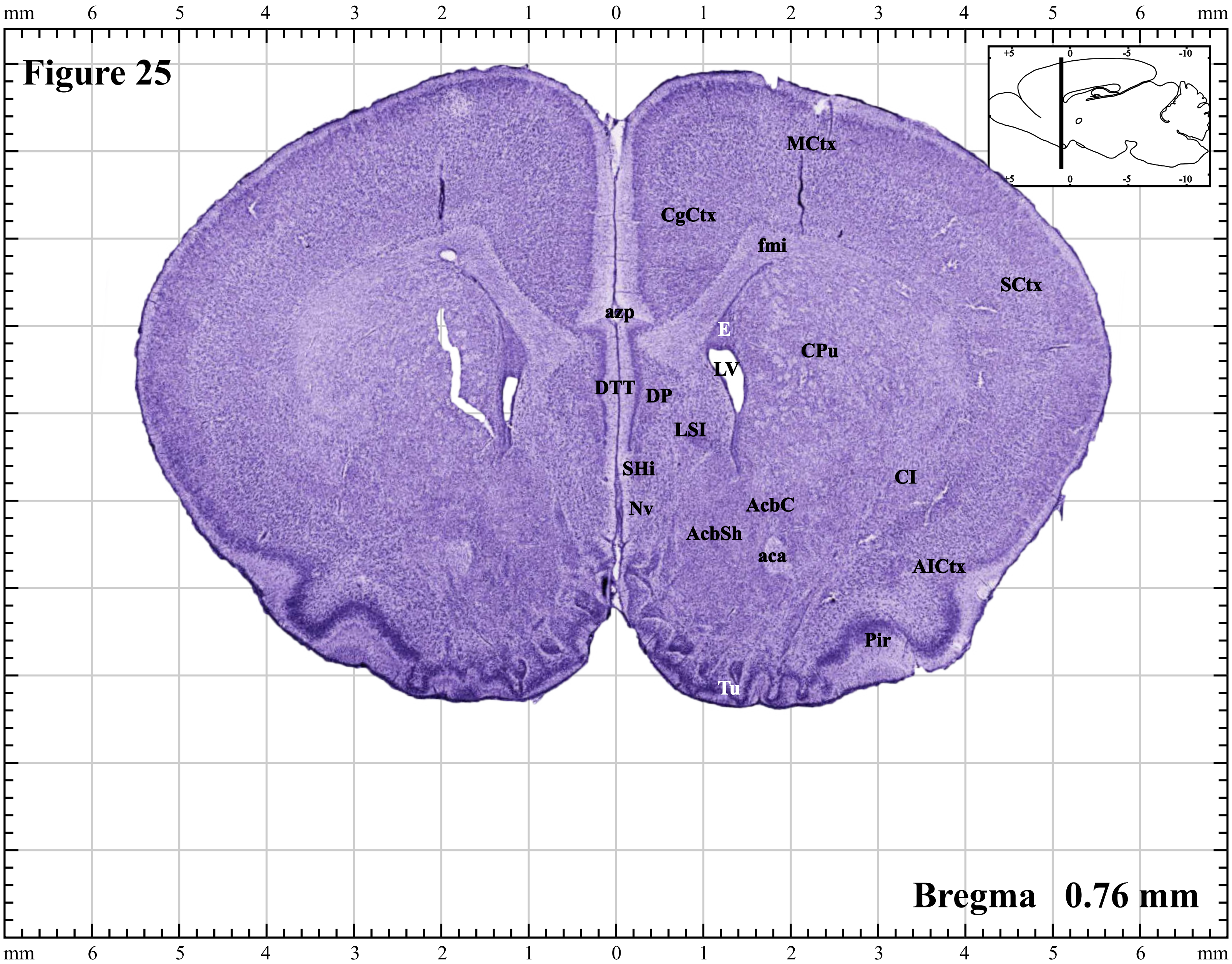

Bregma 0.76 mm

aca anterior commissure, anterior part
azp azygous pericallosal artery
AcbC accumbens nucleus, core
AcbSh accumbens shell
AICtx agranular insular cortex
CgCtx cingulate cortex
CI claustrum
CPu caudate putamen (striatum)
DP dorsal peduncular cortex
DTT dorsal tenia tecta
E ependyma and subependymal layer
fmi forceps major of corpus callosum
LV lateral ventricle
LSI lateral septal nucleus, intermediate part
MCtx motor cortex
Nv navicular nucleus of the basal forebrain
Pir piriform cortex
SCtx somatosensory cortex
SHi septohippocampal nucleus
Tu olfactory tubercle

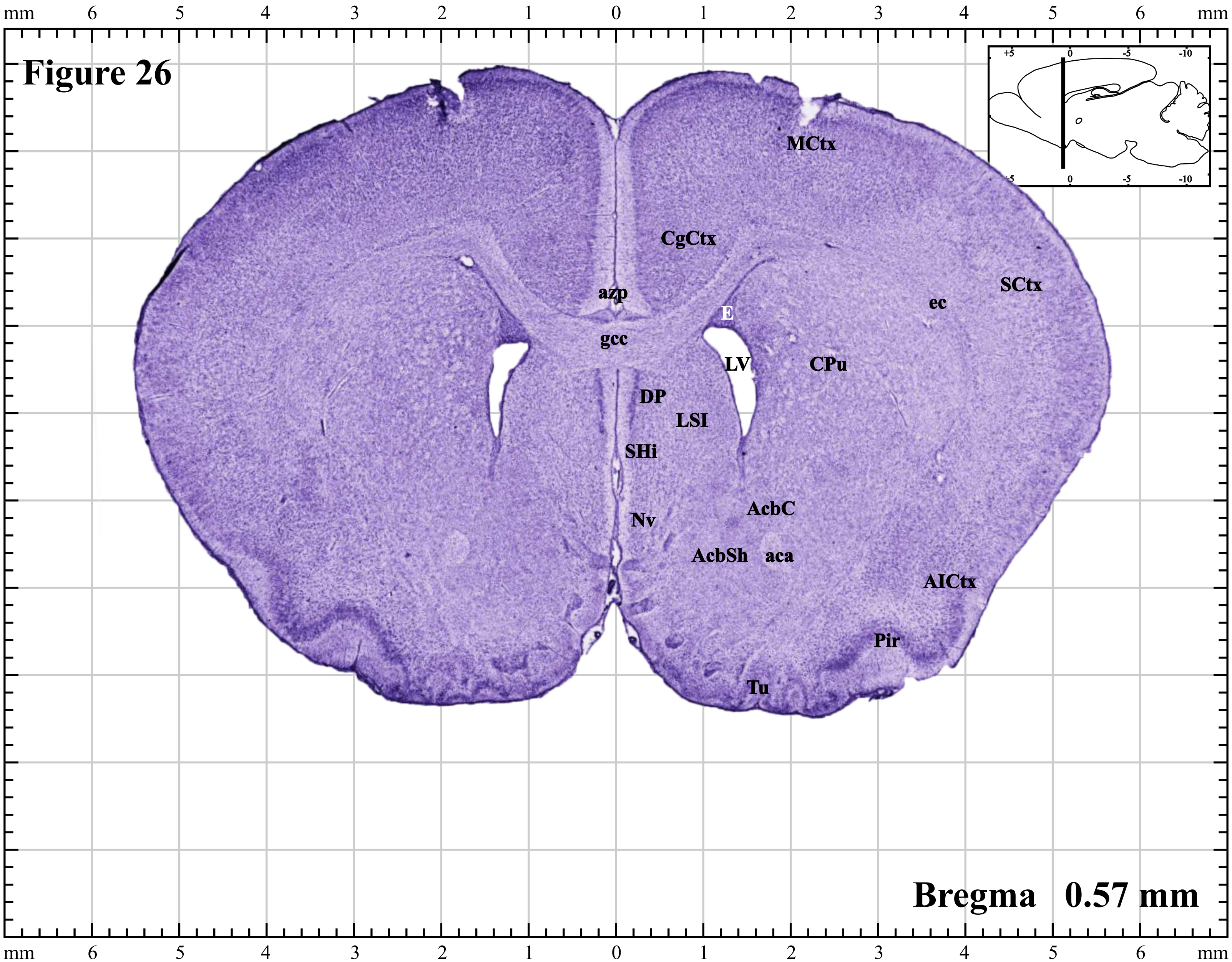

aca anterior commissure, anterior part
azp azygous pericallosal artery
AcbC accumbens nucleus, core
AcbSh accumbens shell
AICtx agranular insular cortex
CgCtx cingulate cortex
CPu caudate putamen (striatum)
DP dorsal peduncular cortex
ec external capsule
E ependyma and subependymal layer
gcc genu of the corpus callosum
fmi forceps major of corpus callosum
LV lateral ventricle
LSI lateral septal nucleus, intermediate part
MCtx motor cortex
Nv navicular nucleus of the basal forebrain
Pir piriform cortex
SCtx somatosensory cortex
SHi septohippocampal nucleus
Tu olfactory tubercle

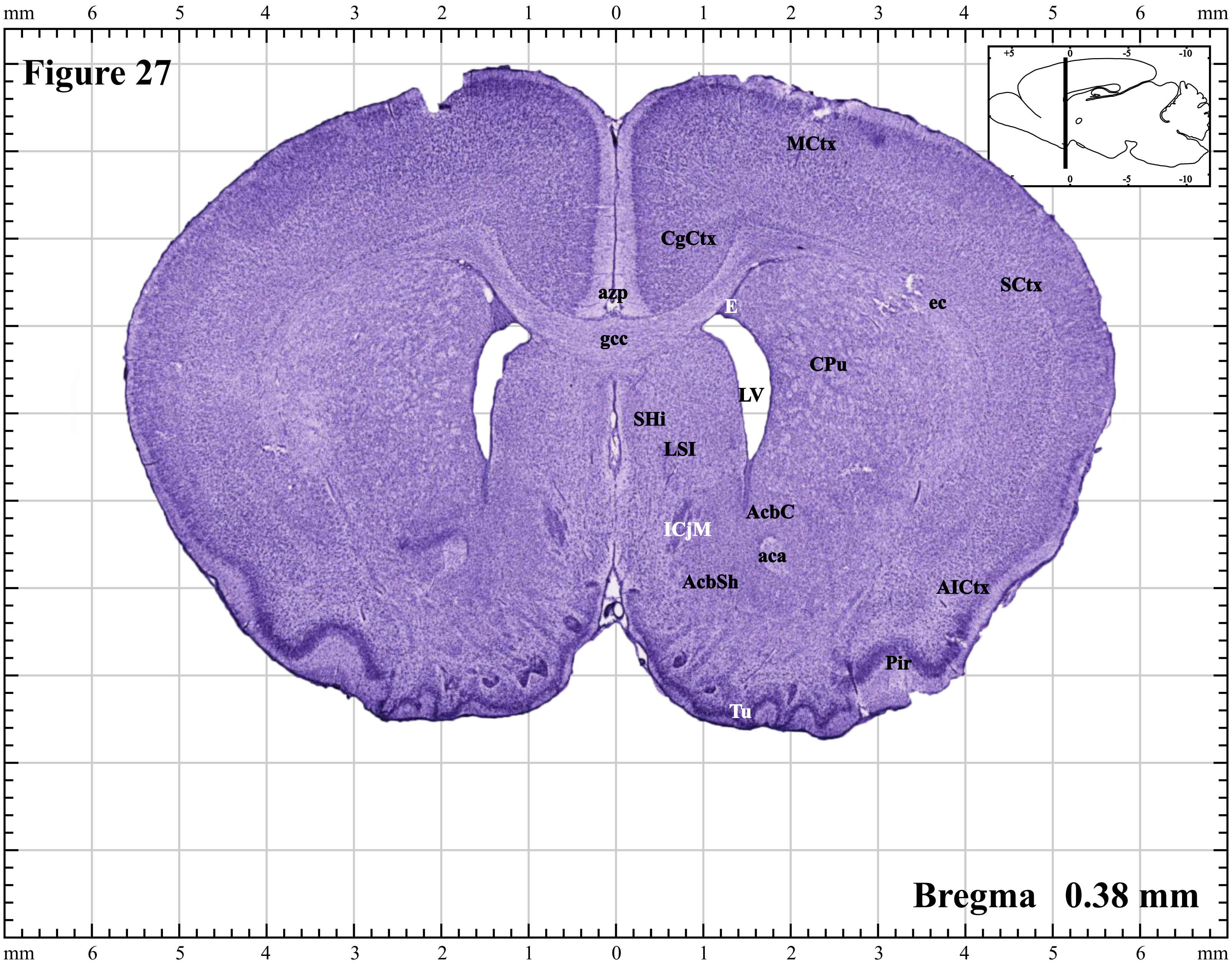

aca anterior commissure, anterior part
azp azygous pericallosal artery
AcbC accumbens nucleus, core
AcbSh accumbens shell
AICtx agranular insular cortex
CgCtx cingulate cortex
CPu caudate putamen (striatum)
ec external capsule
E ependyma and subependymal layer
gcc genu of the corpus callosum
ICjM islands of Calleja, major island
LV lateral ventricle
LSI lateral septal nucleus, intermediate part
MCtx motor cortex
Pir piriform cortex
SCtx somatosensory cortex
SHi septohippocampal nucleus
Tu olfactory tubercle

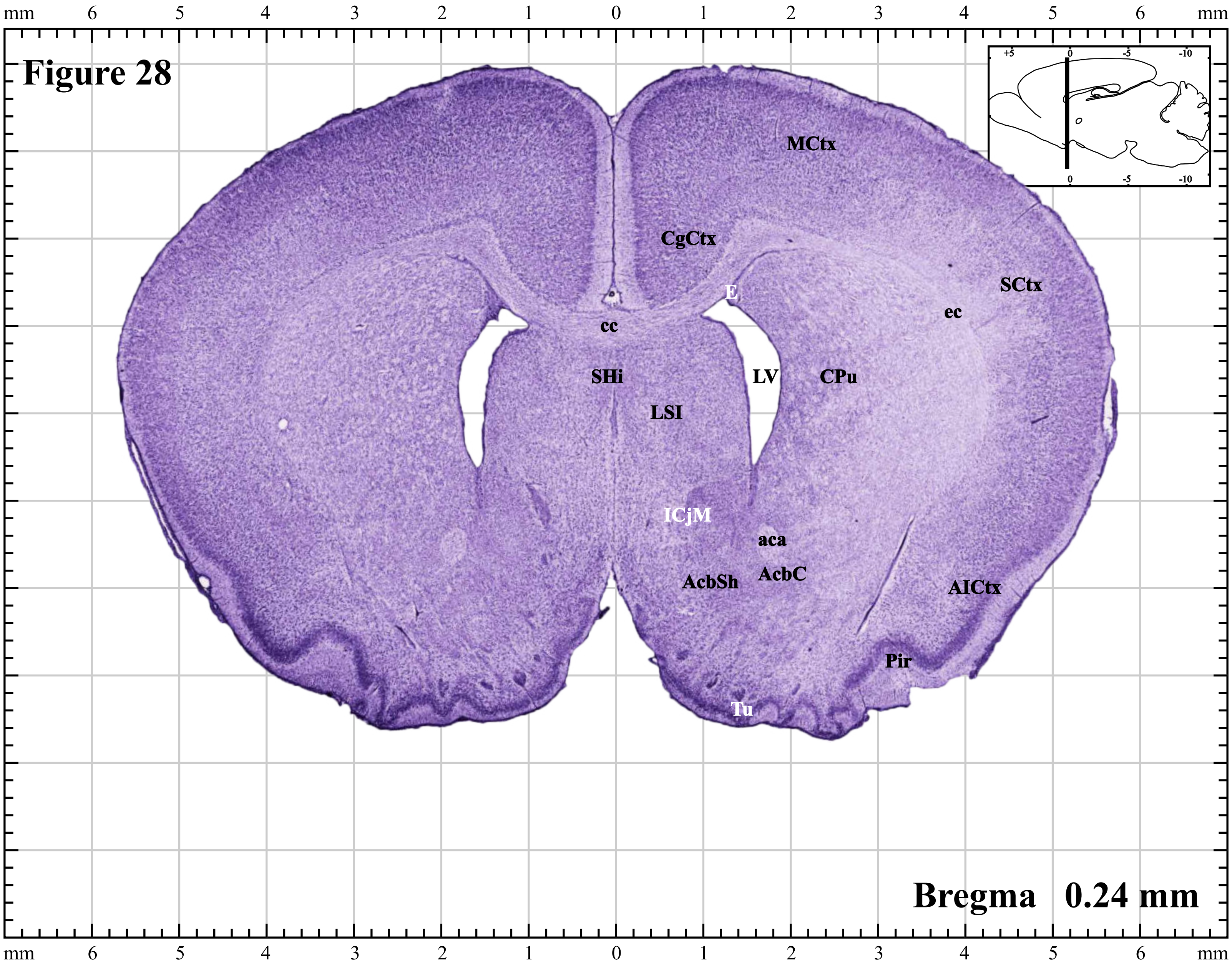

aca anterior commissure, anterior part
AcbC accumbens nucleus, core
AcbSh accumbens shell
AICtx agranular insular cortex
CgCtx cingulate cortex
CPu caudate putamen (striatum)
cc corpus callosum
ec external capsule
E ependyma and subependymal layer
ICjM islands of Calleja, major island
LV lateral ventricle
LSI lateral septal nucleus, intermediate part
MCtx motor cortex
Pir piriform cortex
SCtx somatosensory cortex
SHi septohippocampal nucleus
Tu olfactory tubercle

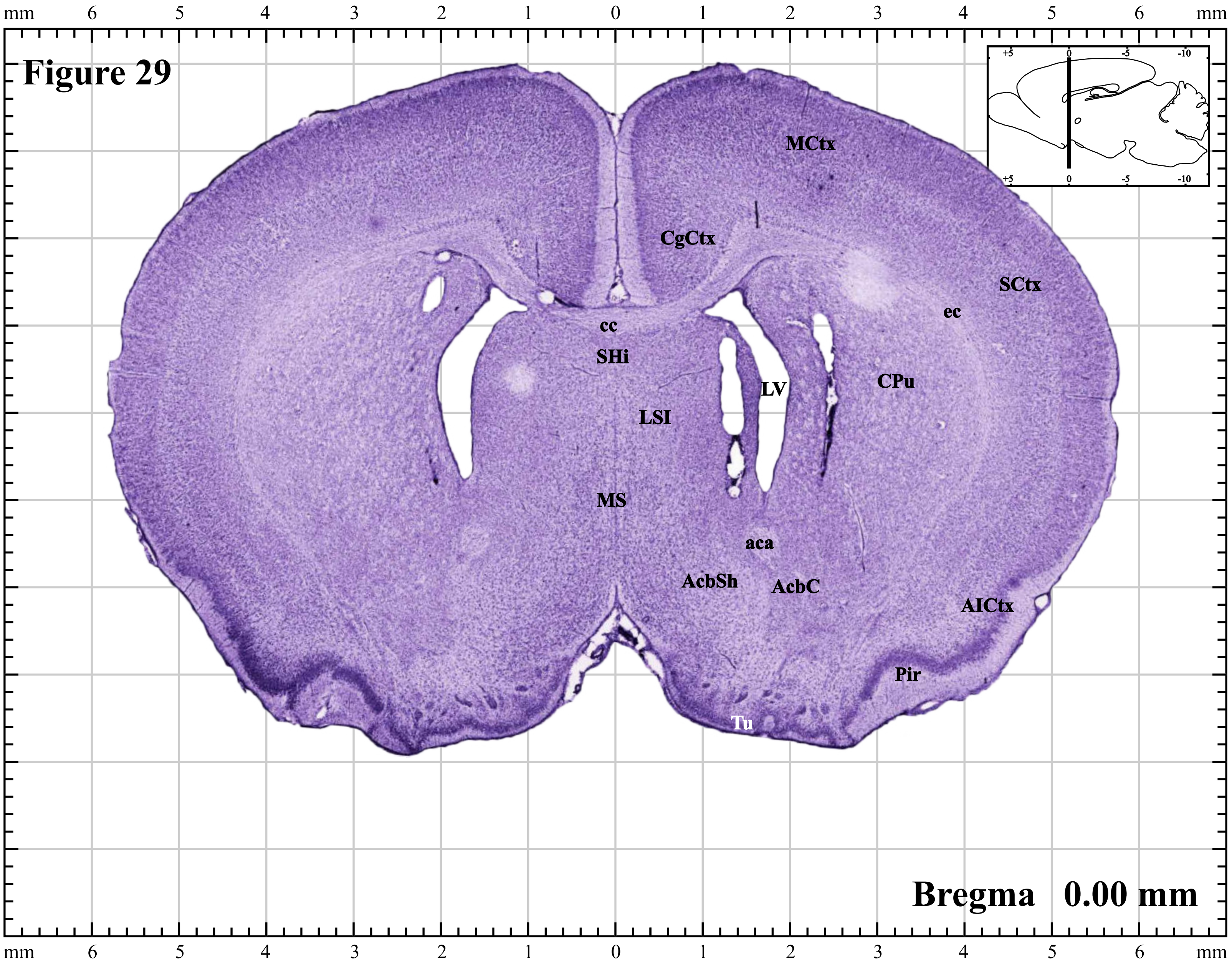

aca anterior commissure, anterior part
AcbC accumbens nucleus, core
AcbSh accumbens shell
AICtx agranular insular cortex
CgCtx cingulate cortex
CPu caudate putamen (striatum)
ec external capsule
cc corpus callosum
LV lateral ventricle
LSI lateral septal nucleus, intermediate part
MCtx motor cortex
MS medial septal nucleus
Pir piriform cortex
SCtx somatosensory cortex
SHi septohippocampal nucleus
Tu olfactory tubercle

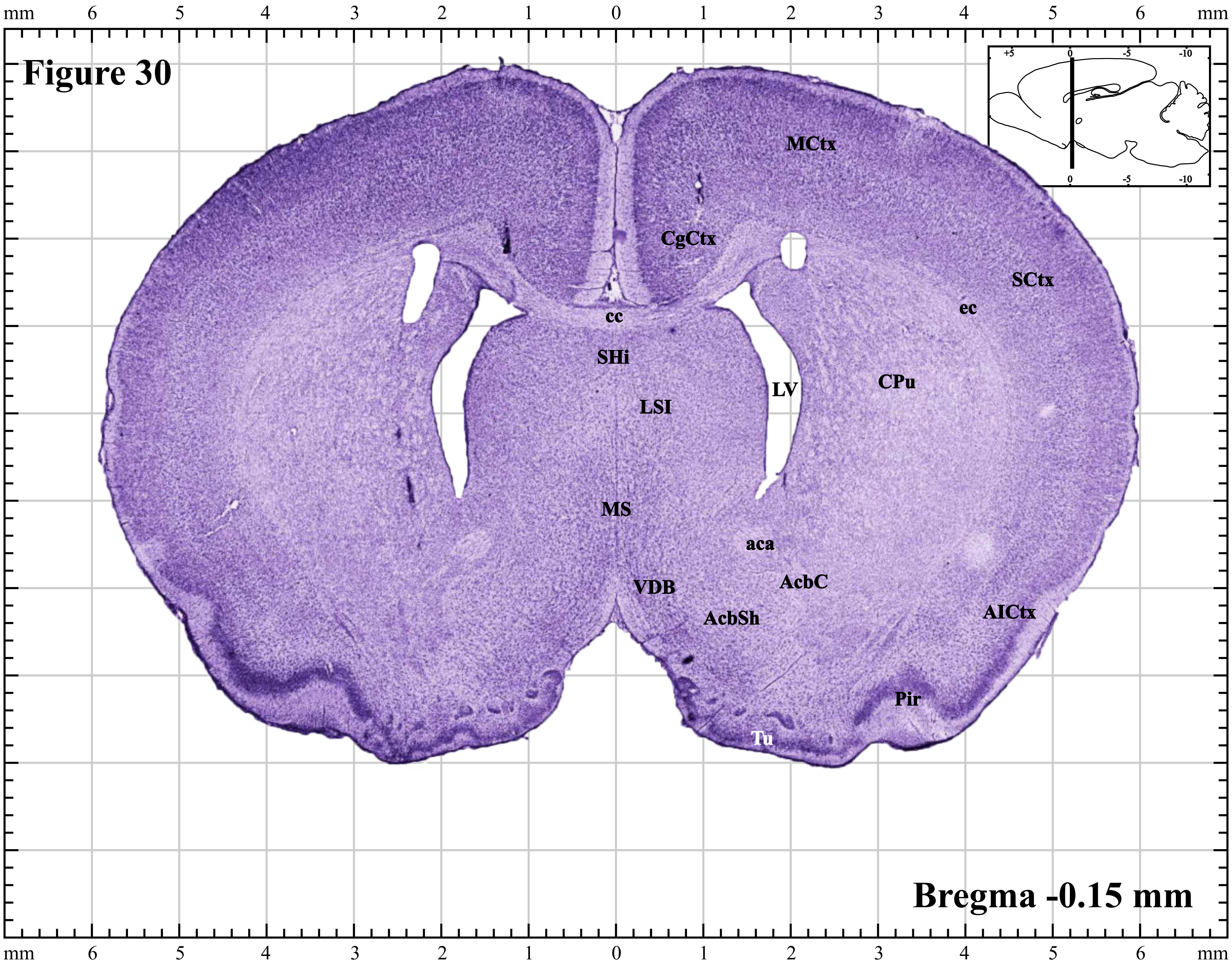

aca anterior commissure, anterior part
AcbC accumbens nucleus, core
AcbSh accumbens shell
AICtx agranular insular cortex
CgCtx cingulate cortex
CPu caudate putamen (striatum)
cc corpus callosum
ec external capsule
LV lateral ventricle
LSI lateral septal nucleus, intermediate part
MCtx motor cortex
MS medial septal nucleus
Pir piriform cortex
SCtx somatosensory cortex
SHi septohippocampal nucleus
VDB nucleus of the vertical limb of the diagonal band
Tu olfactory tubercle

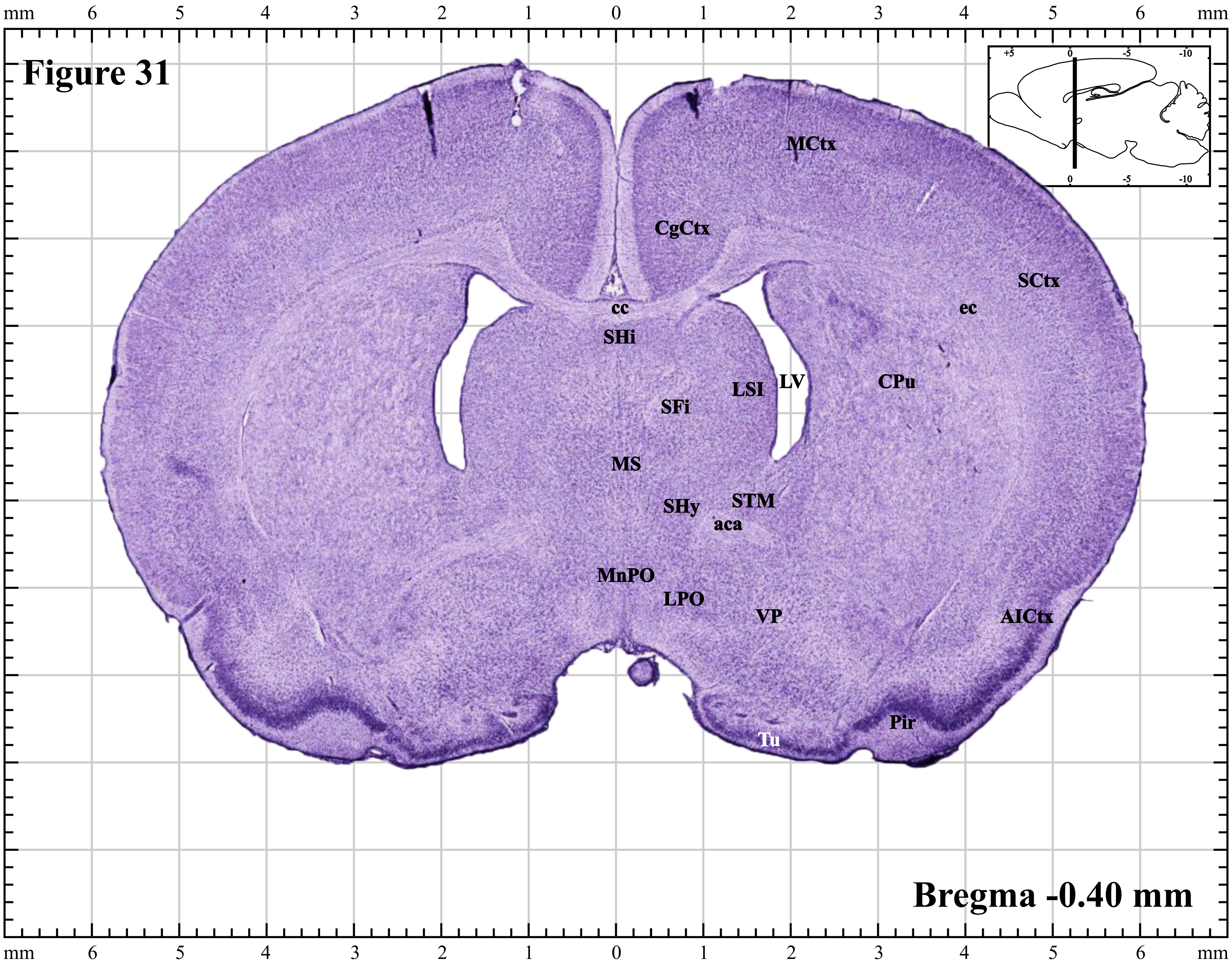

aca anterior commissure, anterior part
AICtx agranular insular cortex
cc corpus callosum
CPu caudate putamen
Cgctx cingulate cortex
ec external capsule
LPO lateral preoptic area
LSI lateral septal nucleus, intermediate part
LV lateral ventricle
MnPO median preoptic nucleus
MCtx motor cortex
MS medial septal nucleus
Pir piriform cortex
SHi septohippocampal nucleus
SCtx somatosensory cortex
SHy septohypothalamic nucleus
SFi septofimbrial nucleus
STM bed nucleus of the stria terminalis, medial division
VP ventral pallidum
Tu olfactory tubercle

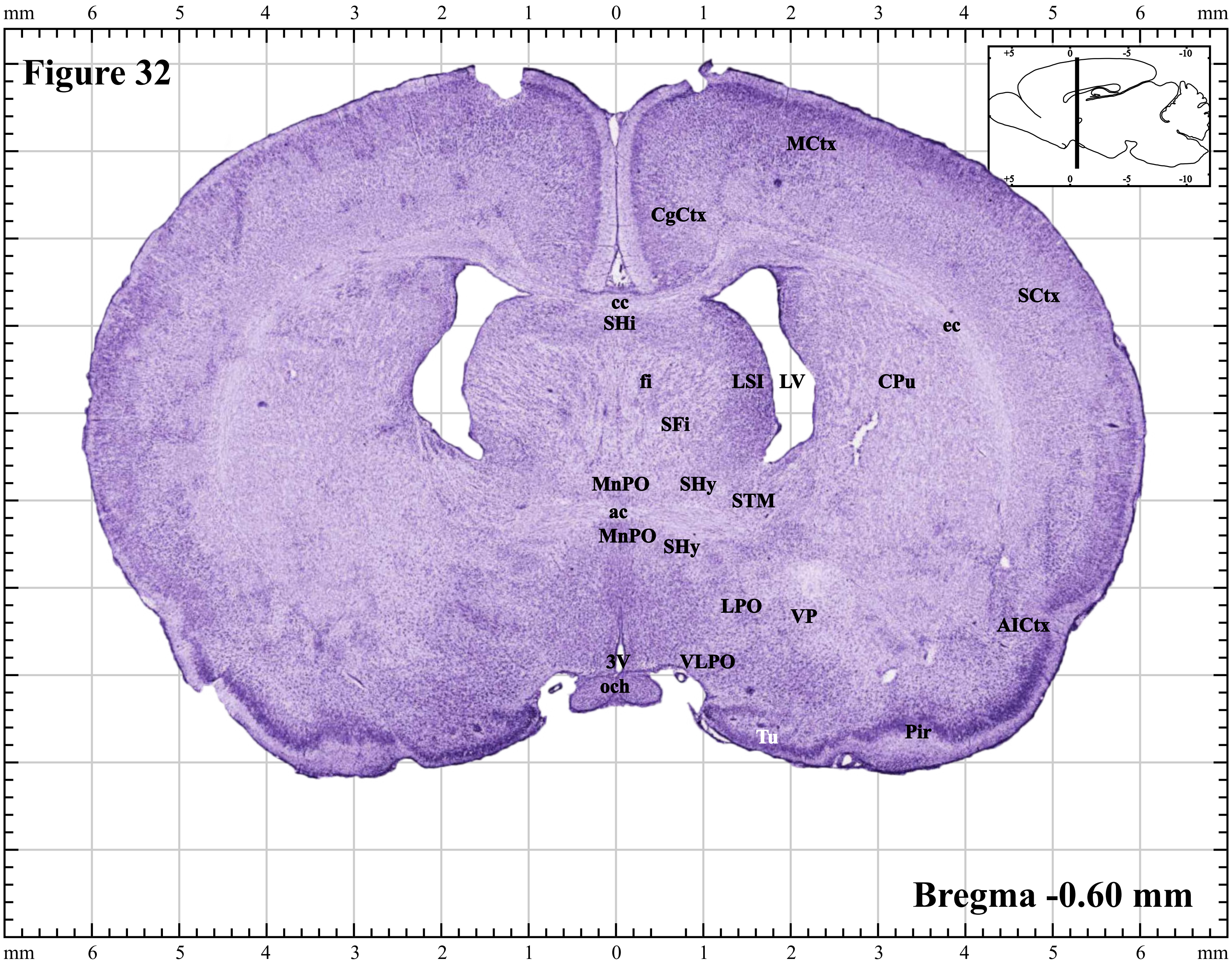

3V 3rd ventricle
ac anterior commissure
AICtx agranular insular cortex
cc corpus callosum
CPu caudate putamen
Cgctx cingulate cortex
ec external capsule
LPO lateral preoptic area
LSI lateral septal nucleus, intermediate part
LV lateral ventricle
MnPO median preoptic nucleus
MCtx motor cortex
MS medial septal nucleus
och optic chiasm
Pir piriform cortex
SHi septohippocampal nucleus
SCtx somatosensory cortex
SHy septohypothalamic nucleus
SFi septofimbrial nucleus
STM bed nucleus of the stria terminalis, medial division
VP ventral pallidum
VLPO ventrolateral preoptic nucleus
Tu olfactory tubercle

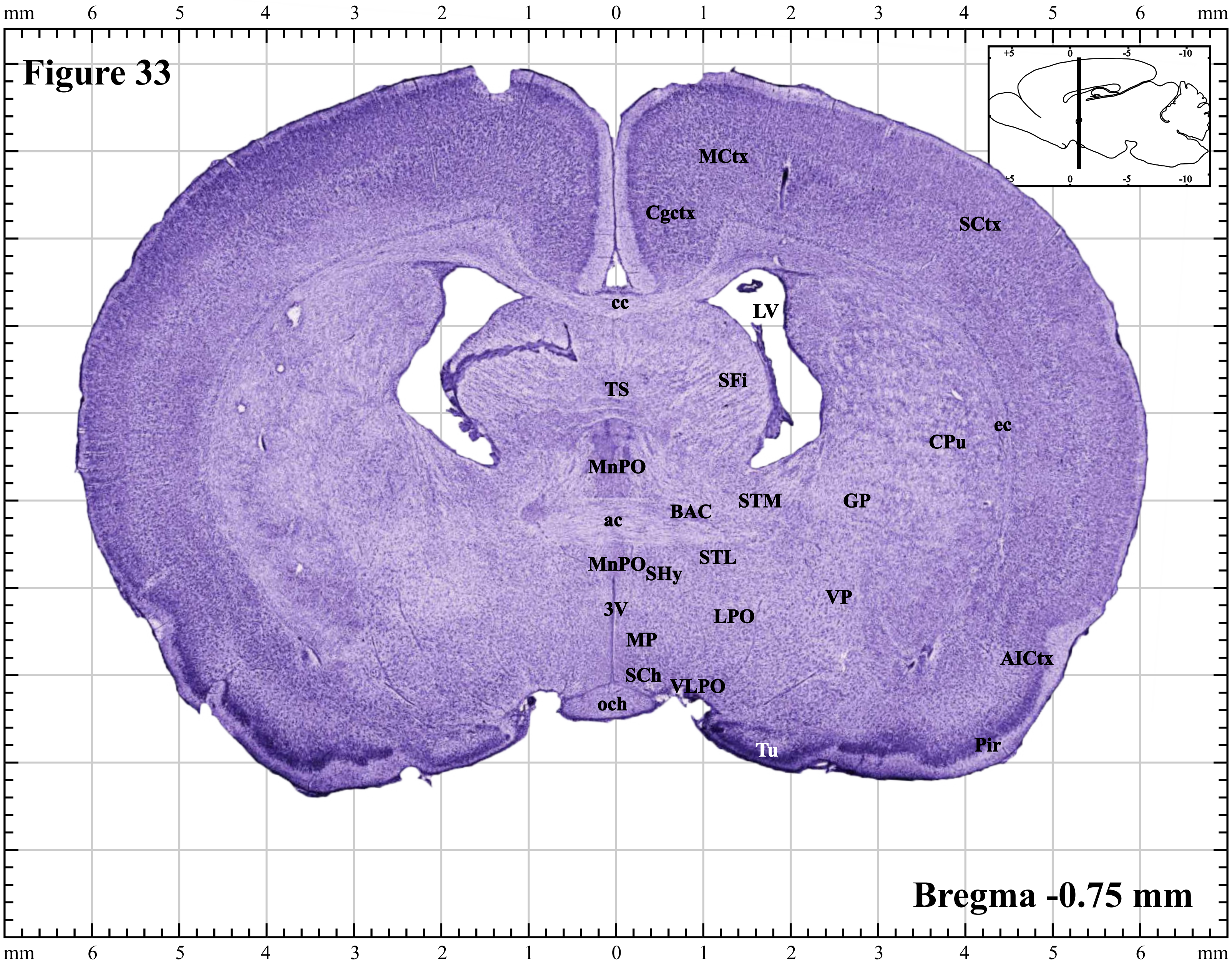

3V 3rd ventricle
ac anterior commissure
AICtx agranular insular cortex
BAC bed nucleus of the anterior commissure
cc corpus callosum
CPu caudate putamen
Cgctx cingulate cortex
ec external capsule
GP globus pallidus
LPO lateral preoptic area
LV lateral ventricle
MCtx motor cortex
MP medial preoptic nucleus
MnPO median preoptic nucleus
och optic chiasm
Pir piriform cortex
SCtx somatosensory cortex
SCh suprachiasmatic nucleus
STM bed nucleus of the stria terminalis, medial division
STL bed nucleus of the stria terminalis, lateral division
SHy septohypothalamic nucleus
SFi septofimbrial nucleus
TS triangular septal nucleus
VLPO ventrolateral preoptic nucleus
VP ventral pallidum
Tu olfactory tubercle

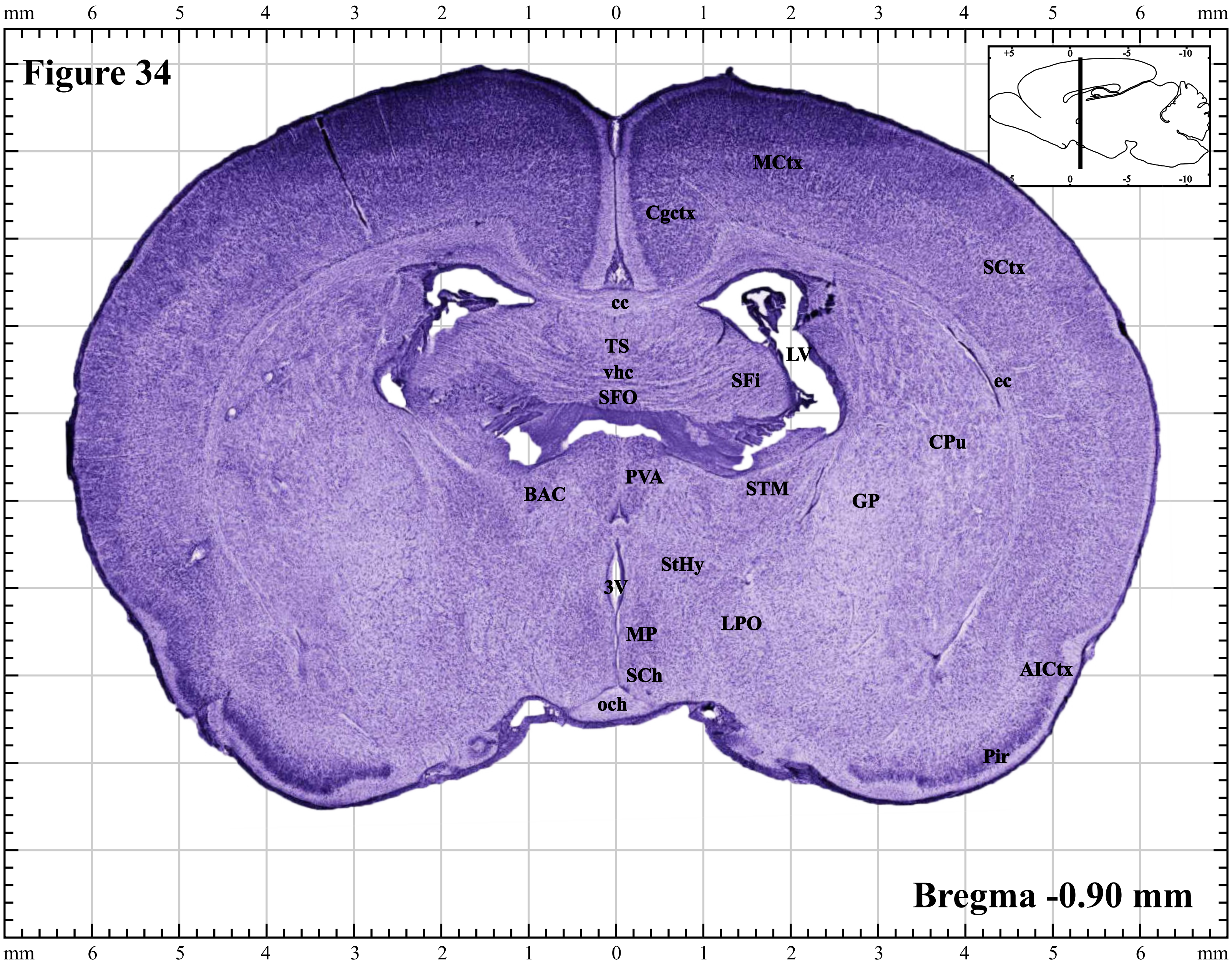

Figure 34

3V 3rd ventricle
AICtx agranular insular cortex
BAC bed nucleus of the anterior commissure
cc corpus callosum
CPu caudate putamen
Cgctx cingulate cortex
ec external capsule
GP globus pallidus
LPO lateral preoptic area
LV lateral ventricle
MCtx motor cortex
MP medial preoptic nucleus
MnPO median preoptic nucleus
och optic chiasm
PVA paraventricular thalamic nucleus, anterior part
Pir piriform cortex
SCtx somatosensory cortex
STM bed nucleus of the stria terminalis, medial division
StHy striohypothalamic nucleus
SFi septofimbrial nucleus
SFO subfornical organ
TS triangular septal nucleus
vhc ventral hippocampal commissure

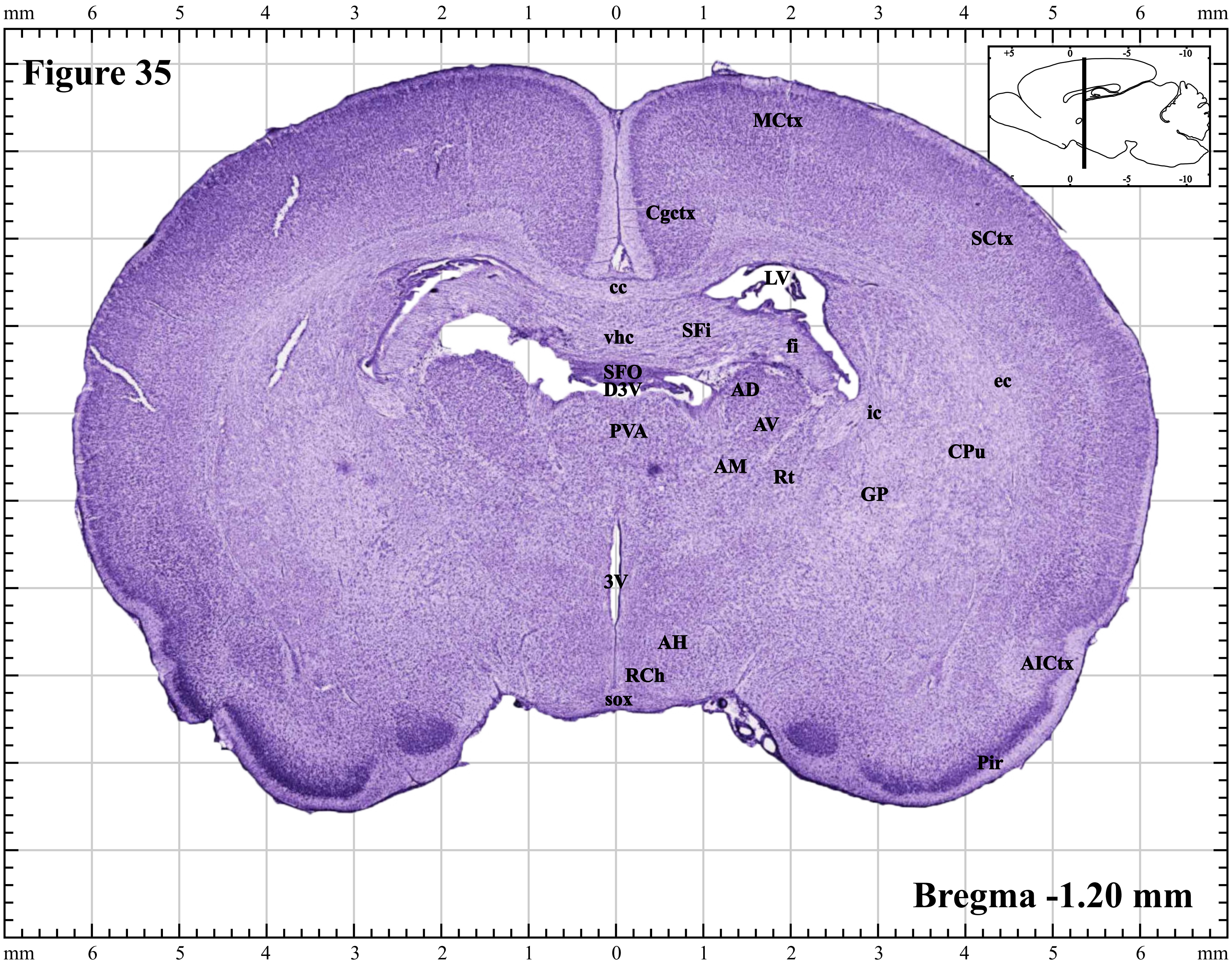

3V 3rd ventricle
AD anterodorsal thalamic nucleus
AH anterior hypothalamic area
AICtx agranular insular cortex
AM anteromedial thalamic nucleus
AV anteroventral thalamic nucleus
cc corpus callosum
CPu caudate putamen
Cgctx cingulate cortex
D3V dorsal 3rd ventricle
ec external capsule
fi fimbria of the hippocampus
GP globus pallidus
ic internal capsule
LV lateral ventricle
MCtx motor cortex
Rt reticular thalamic nucleus
RCh retrochiasmatic area
och optic chiasm
PVA paraventricular thalamic nucleus, anterior part
Pir piriform cortex
SCtx somatosensory cortex
SFi septofimbrial nucleus
SFO subfornical organ
vhc ventral hippocampal commissure
sox supraoptic decussation

Figure 36

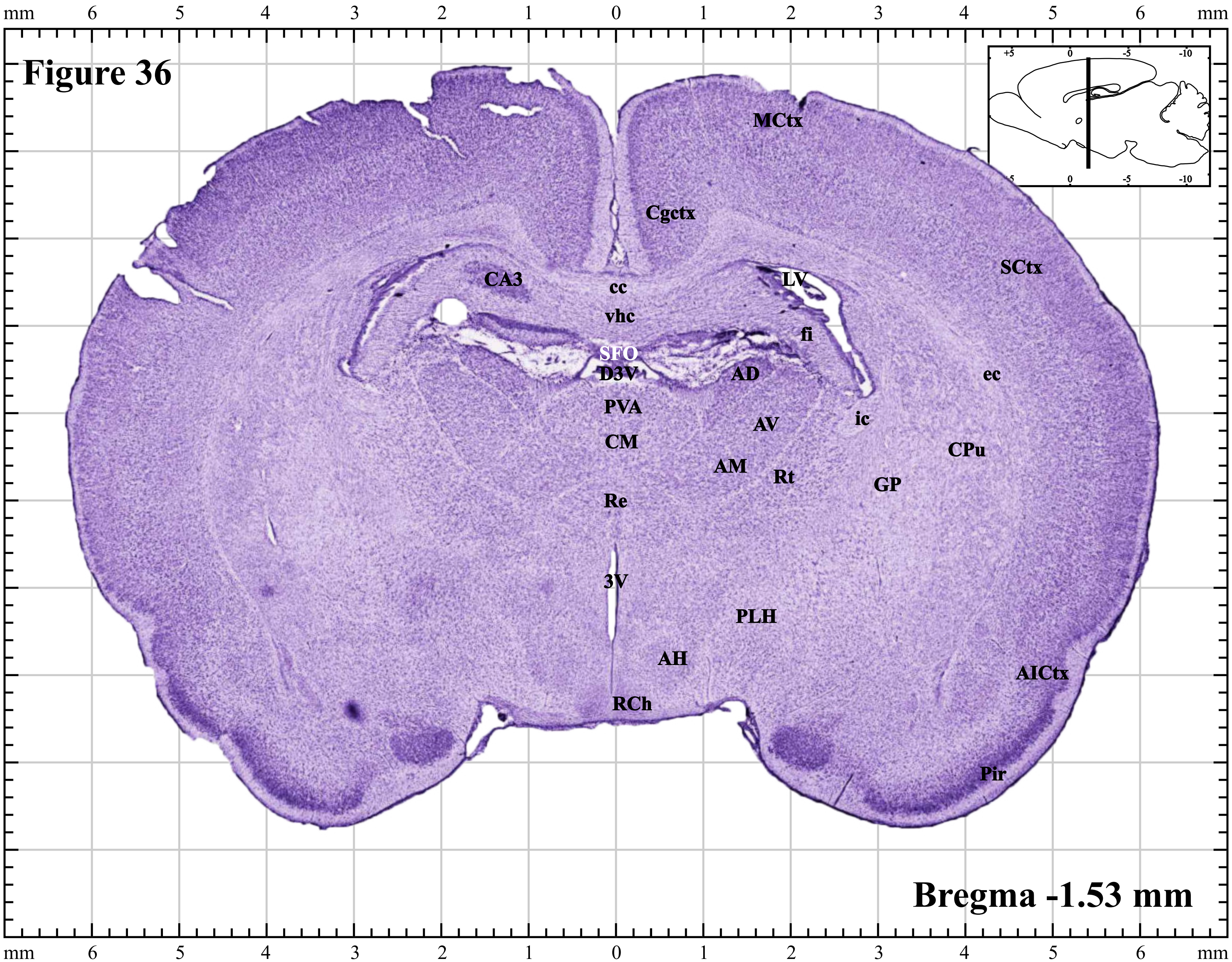

3V 3rd ventricle
AD anterodorsal thalamic nucleus
AH anterior hypothalamic area
AICtx agranular insular cortex
AM anteromedial thalamic nucleus
AV anteroventral thalamic nucleus
cc corpus callosum
CPu caudate putamen
Cgctx cingulate cortex
CA3 field CA3 of the hippocampus
CM central medial thalamic nucleus
D3V dorsal 3rd ventricle
ec external capsule
fi fimbria of the hippocampus
GP globus pallidus
ic internal capsule
LV lateral ventricle
MCtx motor cortex
Pir piriform cortex
PVA paraventricular thalamic nucleus, anterior part
PLH peduncular part of lateral hypothalamus
Rt reticular thalamic nucleus
RCh retrochiasmatic area
Re reuniens thalamic nucleus
SCtx somatosensory cortex
SFO subfornical organ
vhc ventral hippocampal commissure

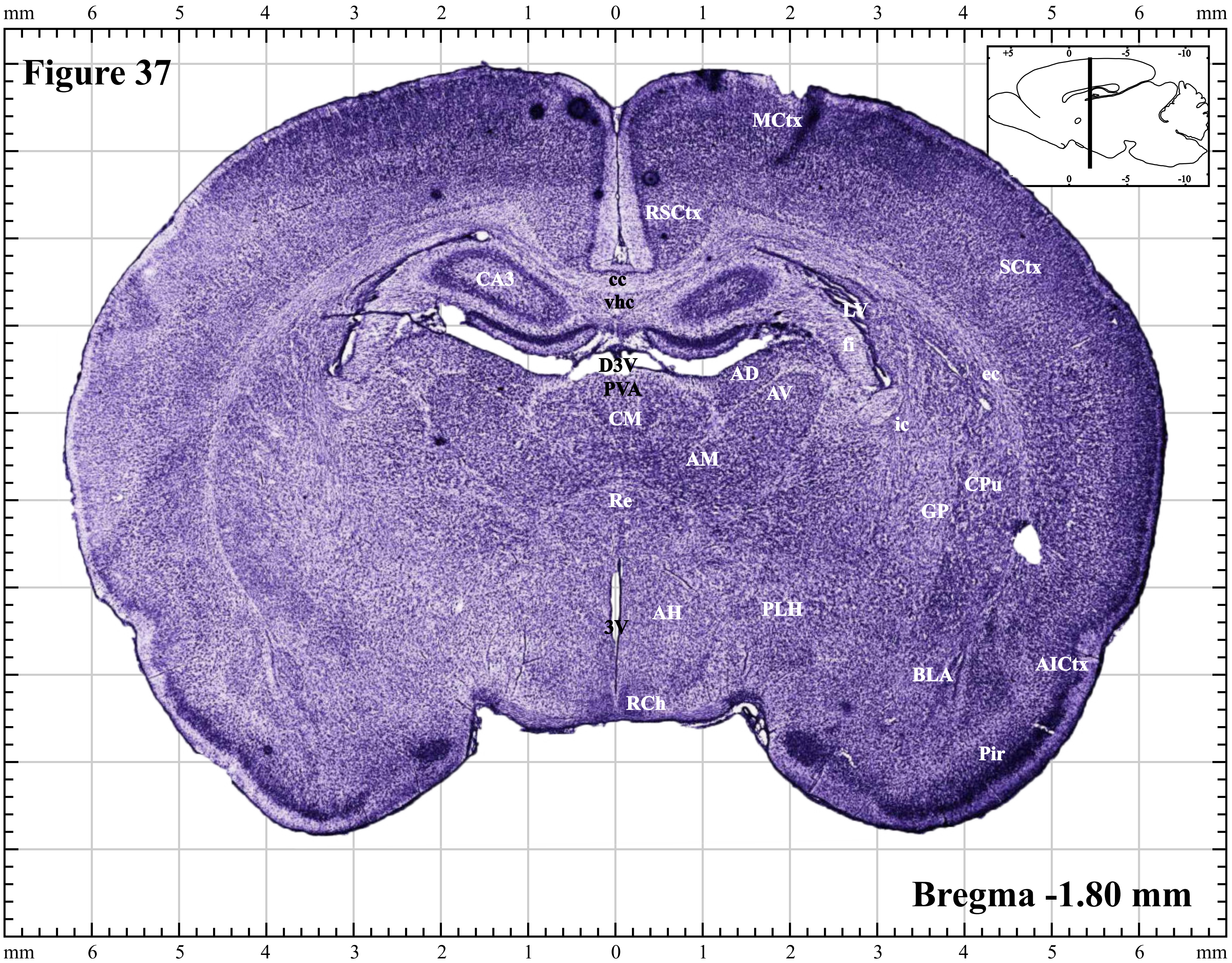

3V 3rd ventricle
AD anterodorsal thalamic nucleus
AH anterior hypothalamic area
AM anteromedial thalamic nucleus
AV anteroventral thalamic nucleus
AICtx agranular insular cortex
BLA basolateral amygdaloid nucleus, anterior part
cc corpus callosum
CPu caudate putamen
CA3 field CA3 of the hippocampus
CM central medial thalamic nucleus
D3V dorsal 3rd ventricle
ec external capsule
fi fimbria of the hippocampus
GP globus pallidus
ic internal capsule
LV lateral ventricle
MCtx motor cortex
Pir piriform cortex
PVA paraventricular thalamic nucleus, anterior part
PLH peduncular part of lateral hypothalamus
RCh retrochiasmatic area
Re reuniens thalamic nucleus
RSCtx retrospenial cortex
SCtx somatosensory cortex
vhc ventral hippocampal commissure

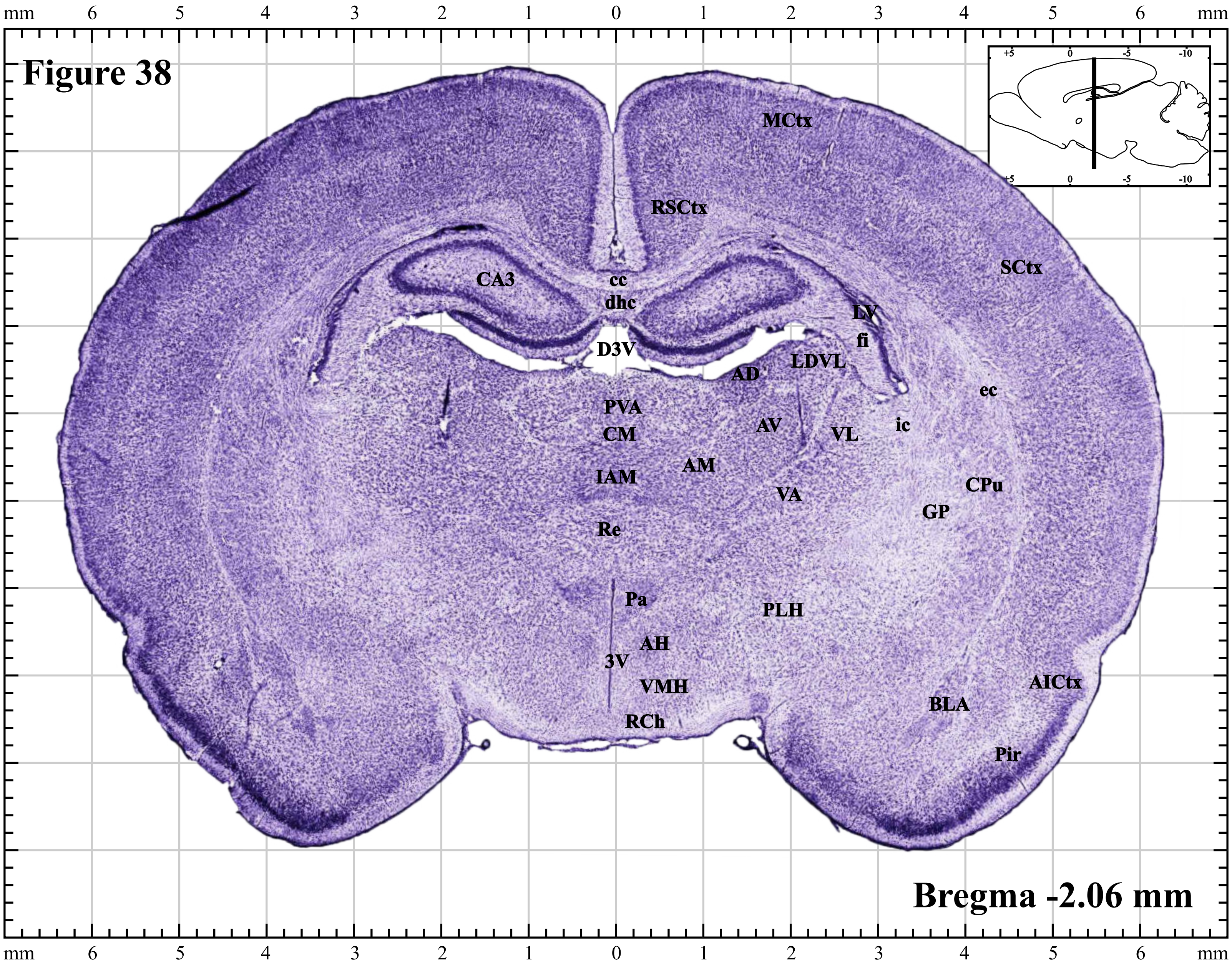

3V 3rd ventricle
AD anterodorsal thalamic nucleus
AH anterior hypothalamic area
AM anteromedial thalamic nucleus
AV anteroventral thalamic nucleus
AICtx agranular insular cortex
BLA basolateral amygdaloid nucleus, anterior part
cc corpus callosum
CPu caudate putamen
CA3 field CA3 of the hippocampus
CM central medial thalamic nucleus
dhc dorsal hippocampal commissure
D3V dorsal 3rd ventricle
ec external capsule
fi fimbria of the hippocampus
GP globus pallidus
ic internal capsule
IAM interanteromedial thalamic nucleus
LV lateral ventricle
LDVL laterodorsal thalamic nucleus, ventrolateral part
MCtx motor cortex
Pir piriform cortex
PVA paraventricular thalamic nucleus, anterior part
PLH peduncular part of lateral hypothalamus
Pa paraventricular hypoth nucleus
RCh retrochiasmatic area
Re reuniens thalamic nucleus
RSCtx retrospenial cortex
SCtx somatosensory cortex
VA ventral anterior thalamic nucleus
VMH ventromedial hypothalamic nucleus
VL ventrolateral thalamic nucleus

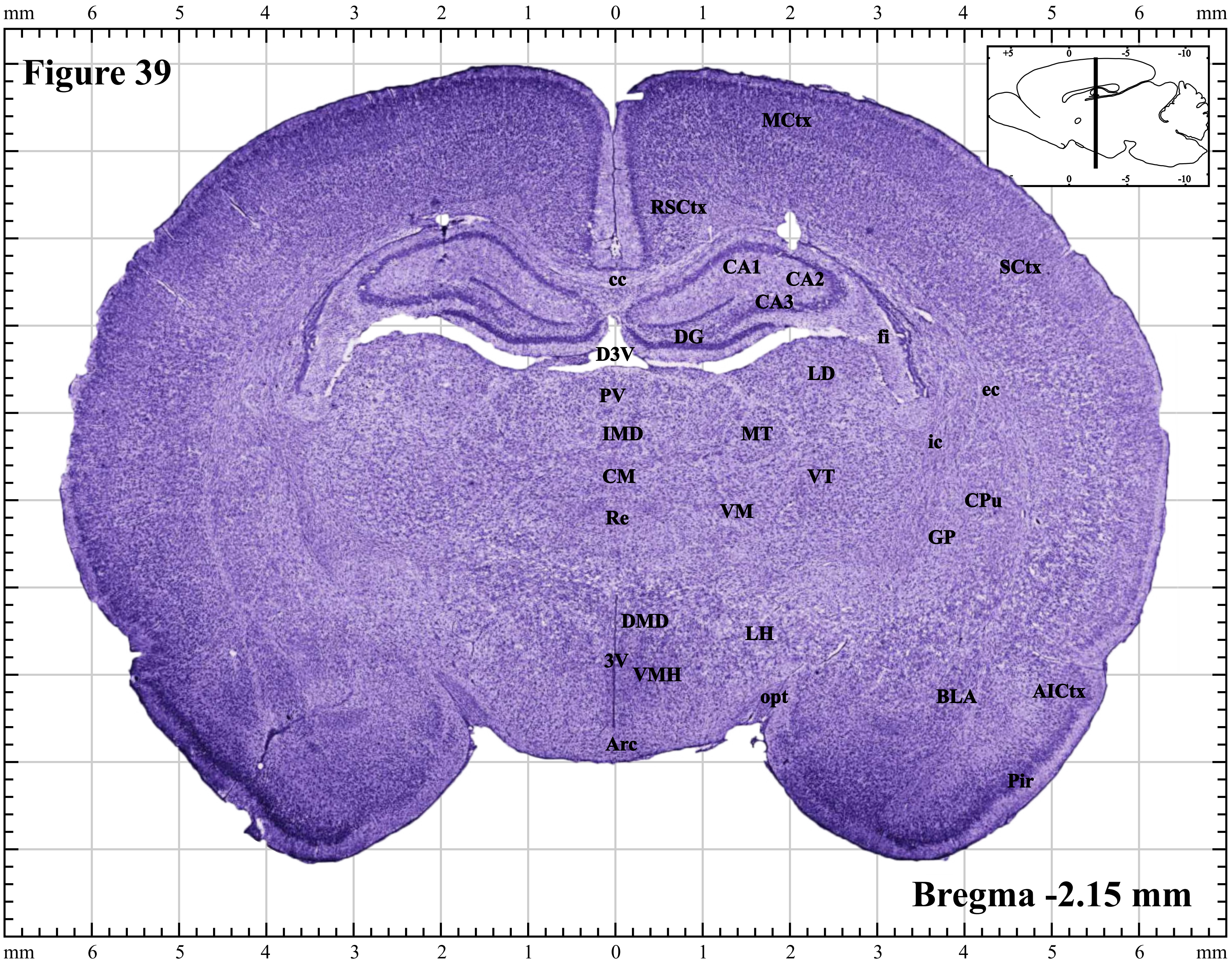

- **3V** 3rd ventricle
- **Arc** arcuate hypothalamic nucleus
- **AICtx** agranular insular cortex
- **BLA** basolateral amygdaloid nucleus, anterior part
- **cc** corpus callosum
- **CA1** field CA1 of the hippocampus
- **CA2** field CA2 of the hippocampus
- **CA3** field CA3 of the hippocampus
- **CPu** caudate putamen
- **Cgctx** cingulate cortex
- **CM** central medial thalamic nucleus
- **DMD** dorsomedial hypothalamic nucleus, dorsal part
- **D3V** dorsal 3rd ventricle
- **DG** dentate gyrus
- **ec** external capsule
- **fi** fimbria of the hippocampus
- **GP** globus pallidus
- **ic** internal capsule
- **IAM** interanteromedial
- **IMD** intermediodorsal thalamic nucleus
- **LH** lateral hypothalamic area
- **LV** lateral ventricle
- **LD** laterodorsal thalamic nucleus
- **MCtx** motor cortex
- **MT** medial thalamus
- **opt** optic tract
- **PV** paraventricular thalamic
- **Pir** piriform cortex
- **Re** reuniens thalamic nucleus
- **RSCtx** retrospenial cortex
- **SCtx** somatosensory cortex
- **VM** ventromedial thalamic nucleus
- **VMH** ventromedial hypothalamic nucleus
- **VT** ventral thalamus

Figure 40

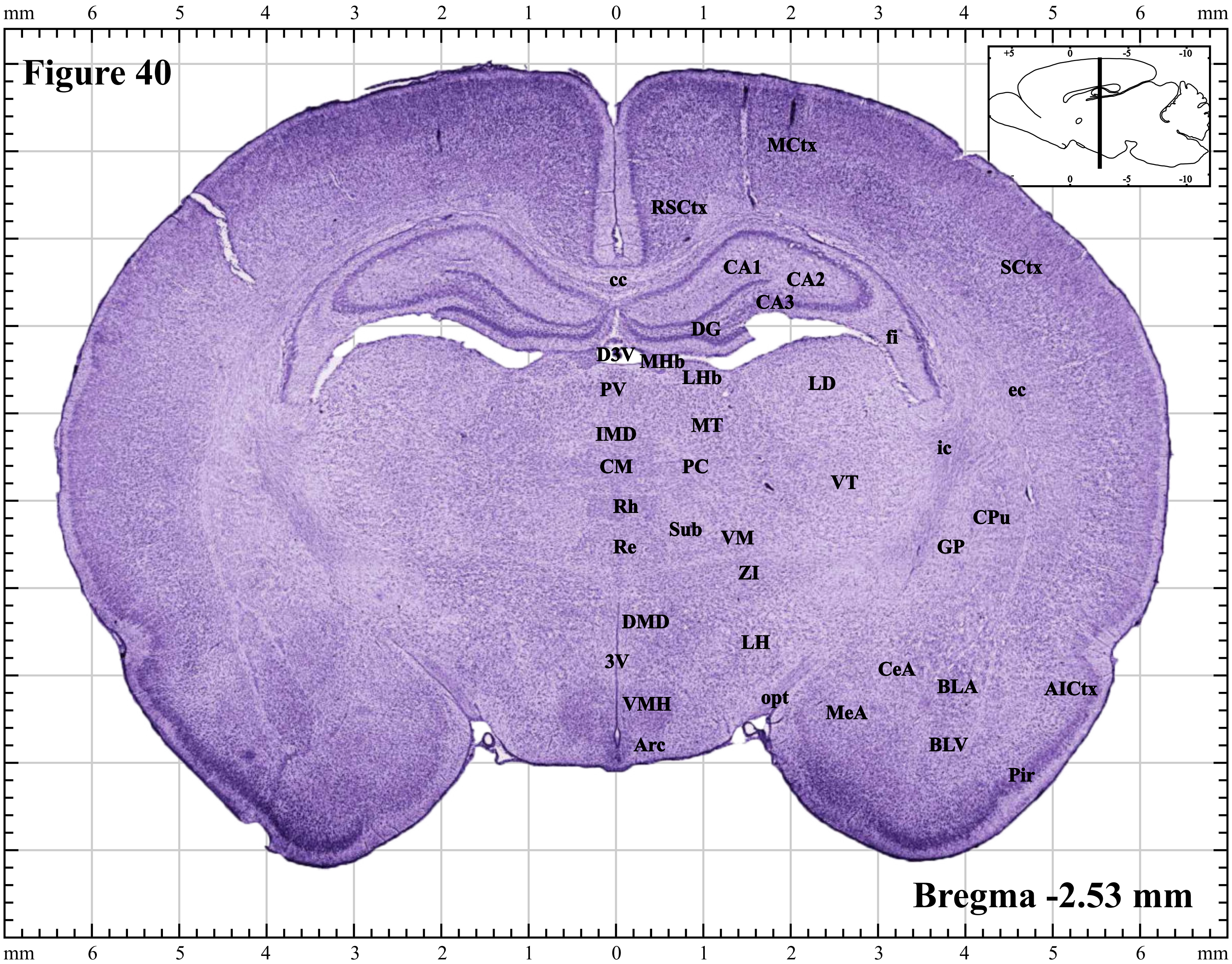

Bregma -2.53 mm

3V medial longitudinal fasciculus
AICtx agranular insular cortex
Arc arcuate hypothalamic nucleus
BLA basolateral amygdaloid nucleus, anterior part
BLV basolateral amygdaloid nucleus, ventral part
CA1 field CA1 of the hippocampus
CA2 field CA2 of the hippocampus
CA3 field CA3 of the hippocampus
cc corpus callosum
CeA central amygdaloid nucleus
CM central medial thalamic nucleus
CPu caudate putamen
D3V dorsal 3rd ventricle
DMD dorsomedial hypothalamic nucleus, dorsal part
DG dentate gyrus
ec external capsule
fi fimbria of the hippocampus
GP globus pallidus
ic internal capsule
IMD intermediodorsal thalamic nucleus
LHb lateral habenular nucleus
LH lateral hypothalamic area
LD laterodorsal thalamic nucleus
MeA medial amygdaloid nucleus
MT medial thalamus
MHb medial habenular nucleus
opt optic tract
PC paracentral thalamic nucleus
Pir piriform cortex
PVP paraventricular thalamic
PtActx parietal association cortex
Rh rhomboid thalamic nucleus
Re reuniens thalamic nucleus
RSCtx retrospenial cortex
Sub submedius thalamic nucleus
SCtx somatosensory cortex
VM ventromedial thalamic nucleus
VMH ventromedial hypothalamic nucleus
VT ventral thalamus
ZI zona incerta

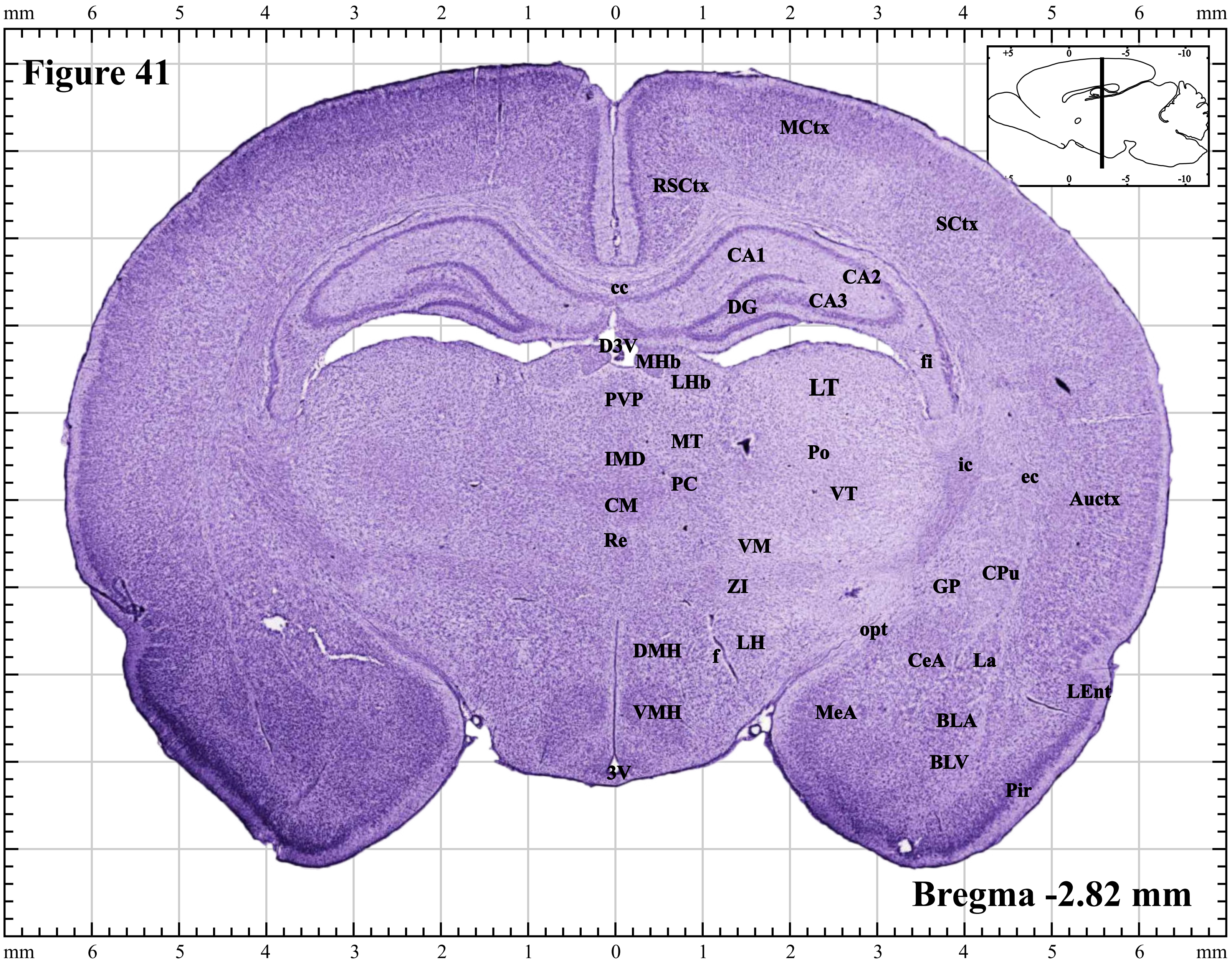

3V medial longitudinal fasciculus
Auctx auditory cortex
BLA basolateral amygdaloid nucleus, anterior part
BLV basolateral amygdaloid nucleus, ventral part
CA1 field CA1 of the hippocampus
CA2 field CA2 of the hippocampus
CA3 field CA3 of the hippocampus
cc corpus callosum
CeA central amygdaloid nucleus
CM central medial thalamic nucleus
CPu caudate putamen
D3V dorsal 3rd ventricle
DMH dorsomedial hypothalamic nucleus
DG dentate gyrus
ec external capsule
f fornix
fi fimbria of the hippocampus
GP globus pallidus
ic internal capsule
IMD intermediodorsal thalamic nucleus
La lat amygdaloid nucleus
LEnt lateral entorhinal cortex
LHb lateral habenular nucleus
LH lateral hypothalamic area
LT lateral thalamus
MeA medial amygdaloid nucleus
MHb medial habenular nucleus
MT medial thalamus
opt optic tract
MCtx motor cortex
PC paracentral thalamic nucleus
Pir piriform cortex
PVP paraventricular thalamic nucleus, posterior part
Po posterior thalamic nuclear group
Re reuniens thalamic nucleus
RSCtx retrospenial cortex
SCtx somatosensory cortex
VM ventromedial thalamic nucleus
VMH ventromedial hypothalamic nucleus
VT ventral thalamus
ZI zona incerta

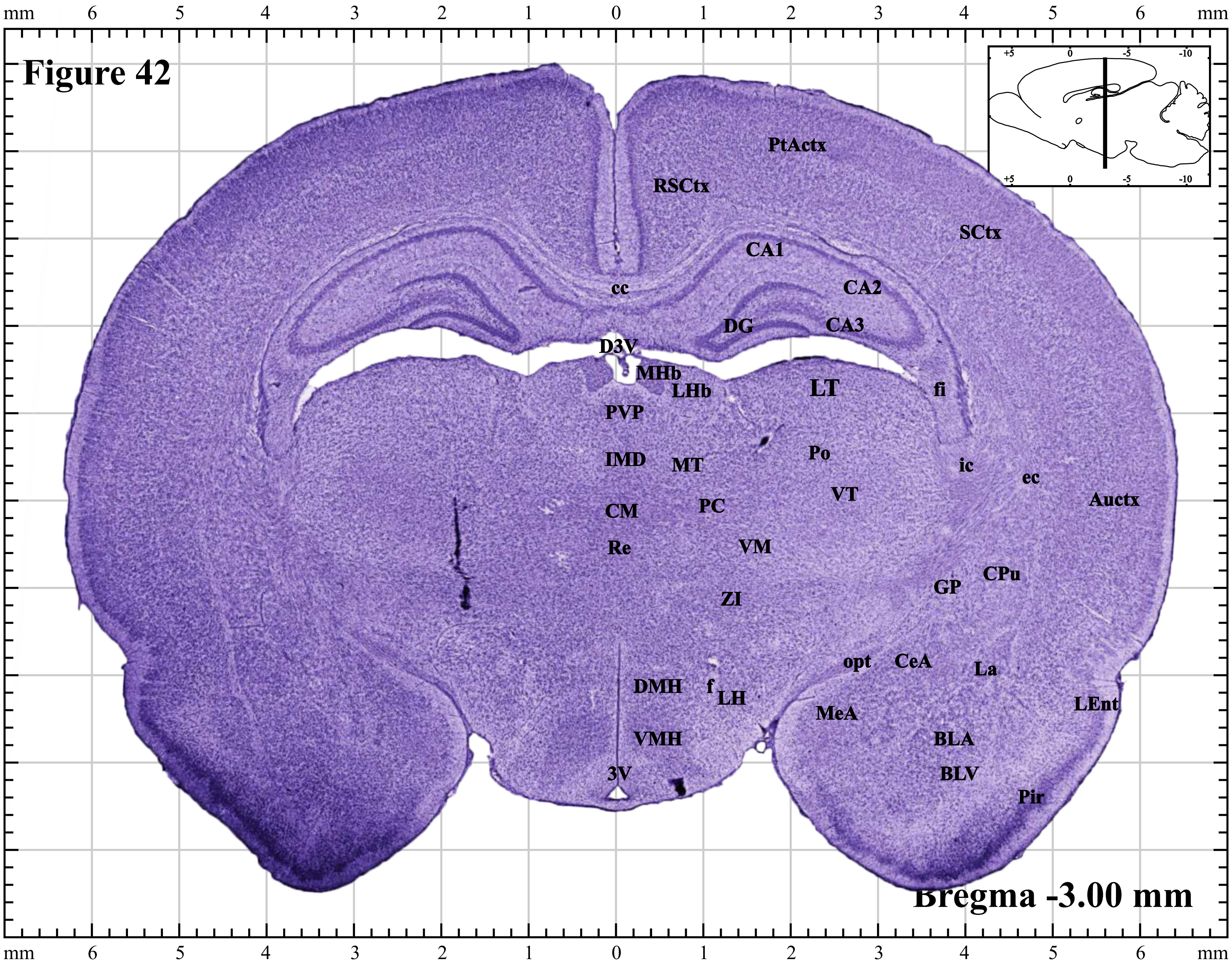

3V medial longitudinal fasciculus
Auctx auditory cortex
BLA basolateral amygdaloid nucleus, anterior part
BLV basolateral amygdaloid nucleus, ventral part
CA1 field CA1 of the hippocampus
CA2 field CA2 of the hippocampus
CA3 field CA3 of the hippocampus
cc corpus callosum
CeA central amygdaloid nucleus
CM central medial thalamic nucleus
CPu caudate putamen
D3V dorsal 3rd ventricle
DMH dorsomedial hypothalamic nucleus
DG dentate gyrus
ec external capsule
f fornix
fi fimbria of the hippocampus
GP globus pallidus
ic internal capsule
IMD intermediodorsal thalamic nucleus
La lat amygdaloid nucleus
LEnt lateral entorhinal cortex
LHb lateral habenular nucleus
LH lateral hypothalamic area
LT lateral thalamus
MeA medial amygdaloid nucleus
MHb medial habenular nucleus
MT medial thalamus
opt optic tract
PC paracentral thalamic nucleus
Pir piriform cortex
PVP paraventricular thalamic nucleus, posterior part
Po posterior thalamic nuclear group
PtActx parietal association cortex
Re reuniens thalamic nucleus
RSCtx retrospenial cortex
SCtx somatosensory cortex
VM ventromedial thalamic nucleus
VMH ventromedial hypothalamic nucleus
VT ventral thalamus
ZI zona incerta

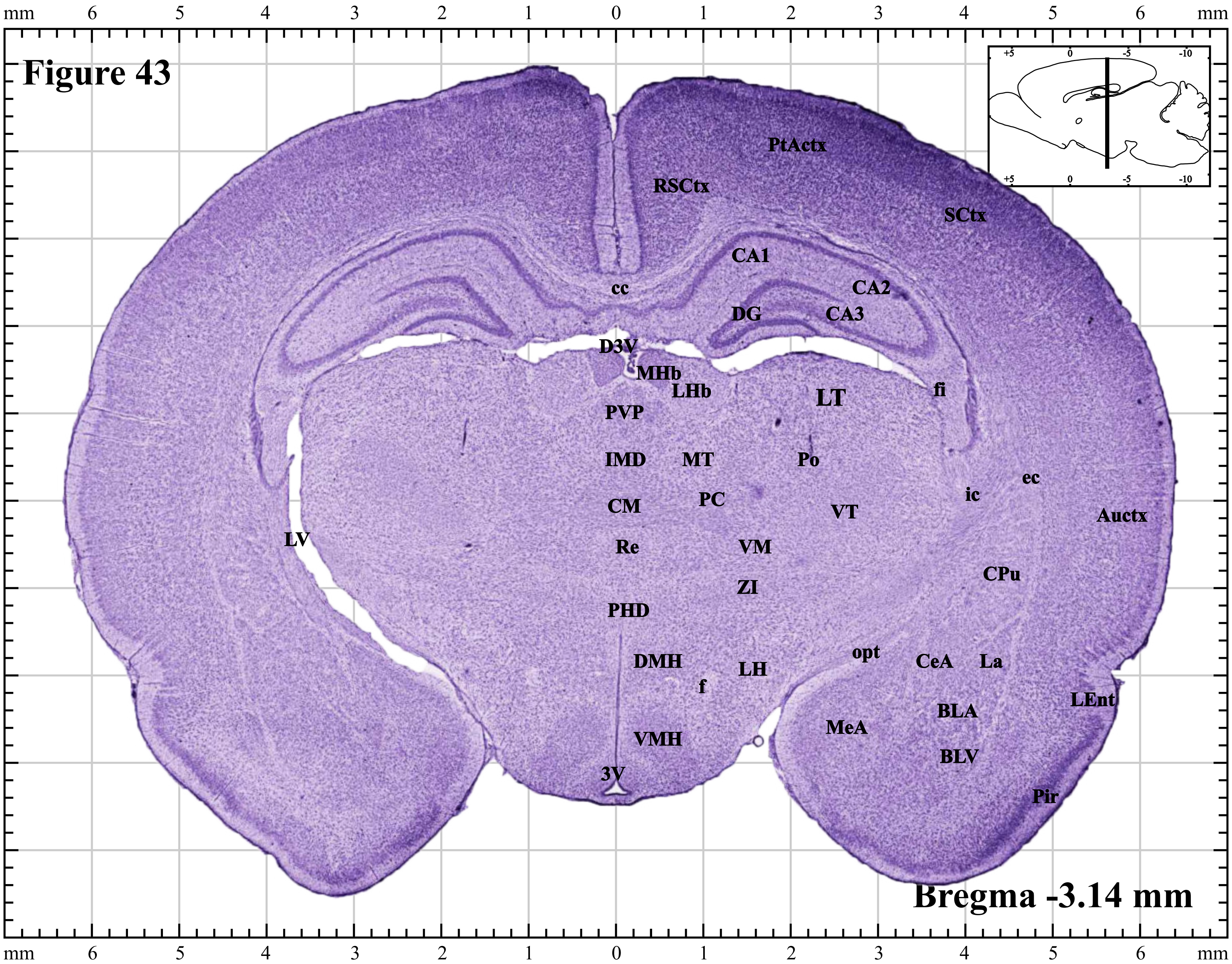

3V medial longitudinal fasciculus
Auctx auditory cortex
BLA basolateral amygdaloid nucleus, anterior part
BLV basolateral amygdaloid nucleus, ventral part
CA1 field CA1 of the hippocampus
CA2 field CA2 of the hippocampus
CA3 field CA3 of the hippocampus
cc corpus callosum
CeA central amygdaloid nucleus
CM central medial thalamic nucleus
CPu caudate putamen
D3V dorsal 3rd ventricle
DMH dorsomedial hypothalamic nucleus
DG dentate gyrus
ec external capsule
f fornix
fi fimbria of the hippocampus
ic internal capsule
IMD intermediodorsal thalamic nucleus
La lat amygdaloid nucleus
LEnt lateral entorhinal cortex
LHb lateral habenular nucleus
LH lateral hypothalamic area
LT lateral thalamus
LV lateral ventricle
MeA medial amygdaloid nucleus
MHb medial habenular nucleus
MT medial thalamus
opt optic tract
PC paracentral thalamic nucleus
Pir piriform cortex
PVP paraventricular thalamic nucleus, posterior part
Po posterior thalamic nuclear group
PHD posterior hypothalamic area, dorsal part
PtActx parietal association cortex
Re reuniens thalamic nucleus
RSCtx retrospenial cortex
SCtx somatosensory cortex
VM ventromedial thalamic nucleus
VMH ventromedial hypothalamic nucleus
VT ventral thalamus
ZI zona incerta

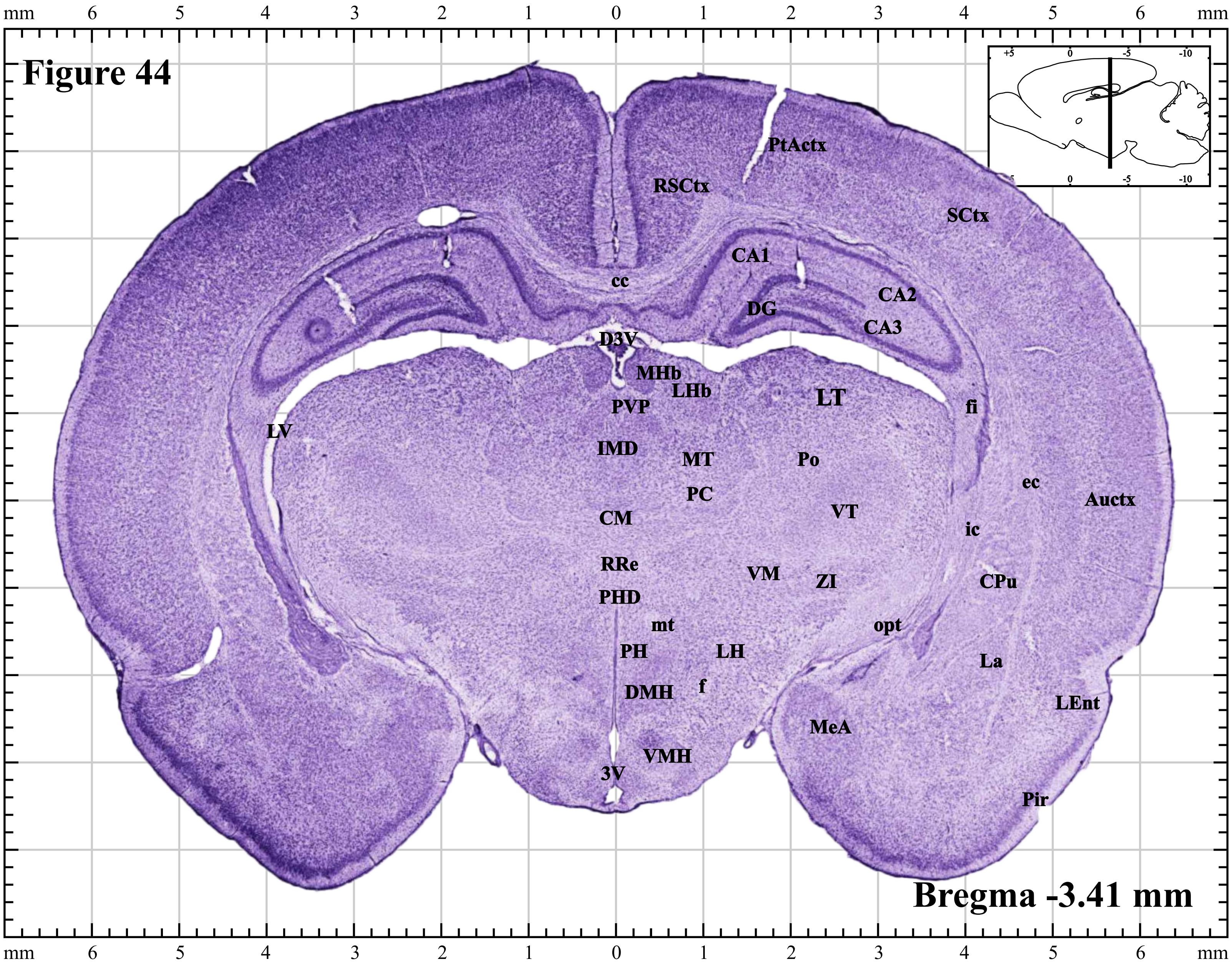

3V medial longitudinal fasciculus
Auctx auditory cortex
CA1 field CA1 of the hippocampus
CA2 field CA2 of the hippocampus
CA3 field CA3 of the hippocampus
cc corpus callosum
CeA central amygdaloid nucleus
CM central medial thalamic nucleus
CPu Caudate putamen
D3V dorsal 3rd ventricle
DMH dorsomedial hypothalamic nucleus
DG dentate gyrus
ec external capsule
f fornix
fi fimbria of the hippocampus
ic internal capsule
IMD intermediodorsal thalamic nucleus
La lat amygdaloid nucleus
LEnt lateral entorhinal cortex
LHb lateral habenular nucleus
LH lateral hypothalamic area
LT lateral thalamus
LV lateral ventricle
mt mammillothalamic tract
MeA medial amygdaloid nucleus
MHb medial habenular nucleus
MT medial thalamus
opt optic tract
PC paracentral thalamic nucleus
Pir piriform cortex
PVP paraventricular thalamic nucleus, posterior part
Po posterior thalamic nuclear group
PH posterior hypothalamic nucleus
PHD posterior hypothalamic area, dorsal part
PtActx parietal association cortex
RRe retrouniens area
RSCtx retrospenial cortex
SCtx somatosensory cortex
VM ventromedial thalamic nucleus
VT ventral thalamus
VMH ventromedial hypothalamic nucleus
ZI zona incerta

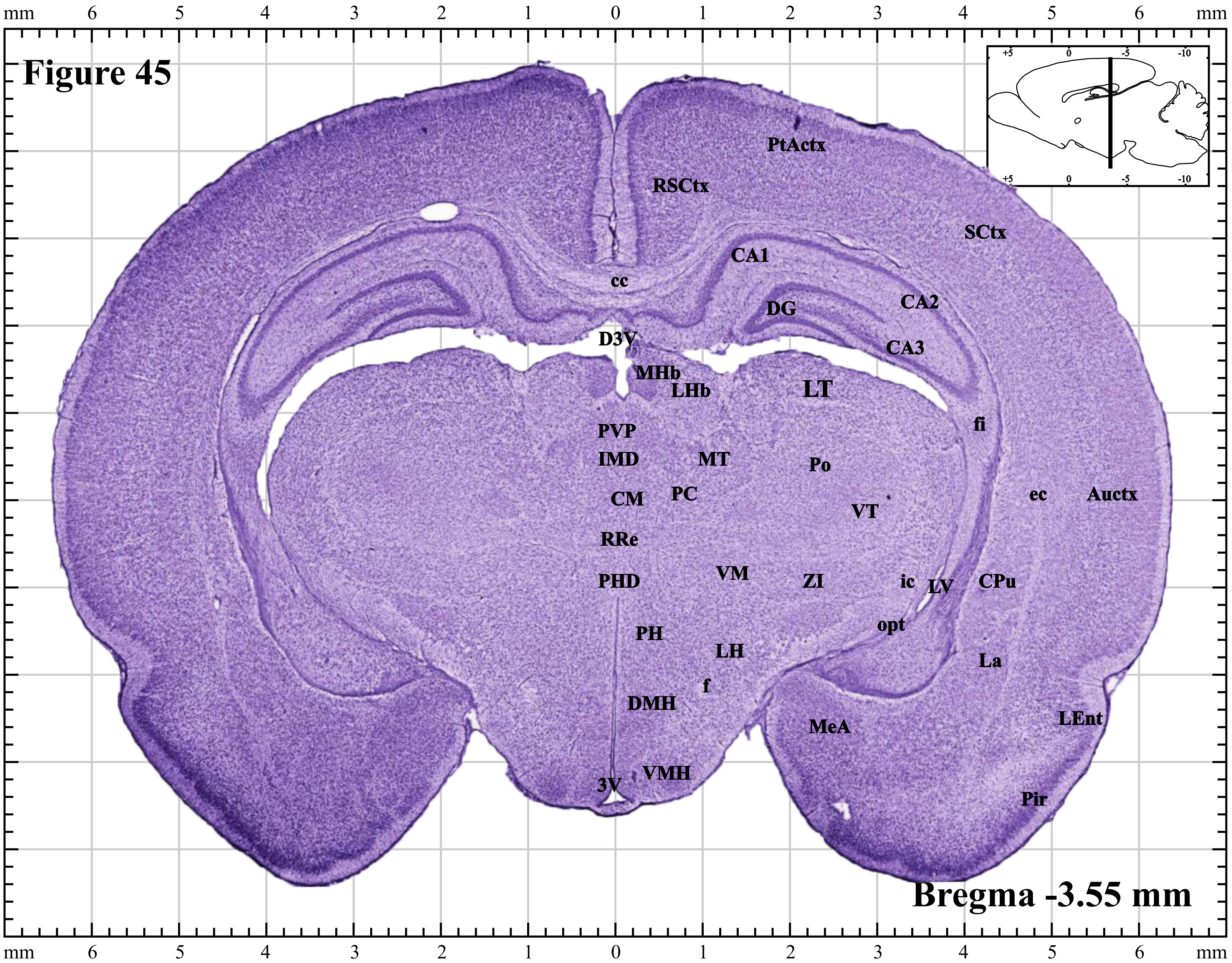

3V medial longitudinal fasciculus
Auctx auditory cortex
CA1 field CA1 of the hippocampus
CA2 field CA2 of the hippocampus
CA3 field CA3 of the hippocampus
cc corpus callosum
CeA central amygdaloid nucleus
CM central medial thalamic nucleus
CPu caudate putamen
D3V dorsal 3rd ventricle
DMH dorsomedial hypothalamic nucleus
DG dentate gyrus
ec external capsule
f fornix
fi fimbria of the hippocampus
ic internal capsule
IMD intermediodorsal thalamic nucleus
La lat amygdaloid nucleus
LEnt lateral entorhinal cortex
LHb lateral habenular nucleus
LH lateral hypothalamic area
LT lateral thalamus
LV lateral ventricle
mt mammillothalamic tract
MeA medial amygdaloid nucleus
MHb medial habenular nucleus
MT medial thalamus
opt optic tract
PC paracentral thalamic nucleus
Pir piriform cortex
PVP paraventricular thalamic nucleus, posterior part
Po posterior thalamic nuclear group
PH posterior hypothalamic nucleus
PHD posterior hypothalamic area, dorsal part
PtActx parietal association cortex
RRe retrouniens area
RSCtx retrospenial cortex
SCtx somatosensory cortex
VM ventromedial thalamic nucleus
VT ventral thalamus
VMH ventromedial hypothalamic nucleus
ZI zona incerta

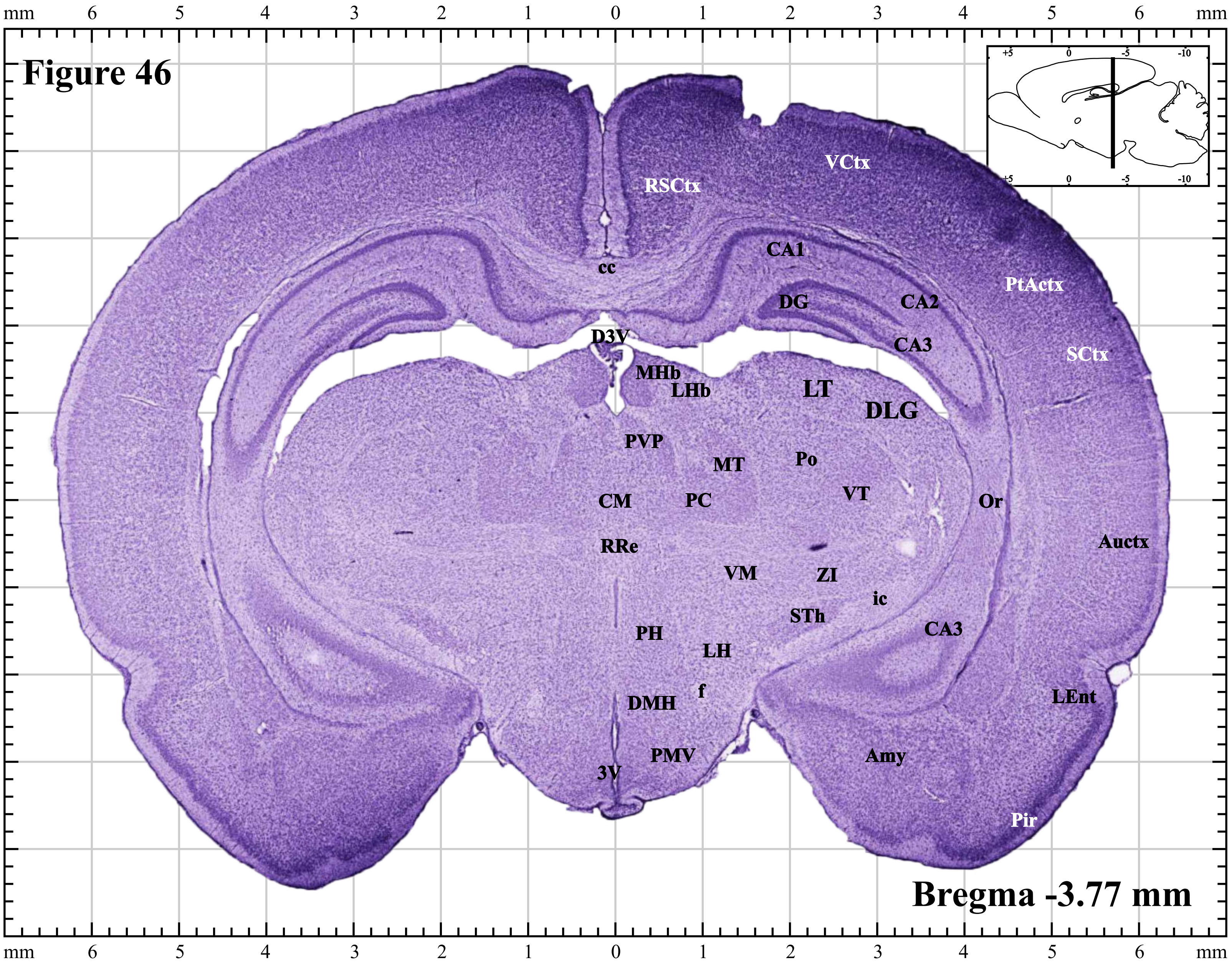

3V medial longitudinal fasciculus
Auctx auditory cortex
Amy amygdaloid nuclei
CA1 field CA1 of the hippocampus
CA2 field CA2 of the hippocampus
CA3 field CA3 of the hippocampus
cc corpus callosum
CM central medial thalamic nucleus
D3V dorsal 3rd ventricle
DMH dorsomedial hypothalamic nucleus
DG dentate gyrus
DLG dorsal lateral geniculate nucleus
f fornix
ic internal capsule
IMD intermediodorsal thalamic nucleus
La lat amygdaloid nucleus
LHb lateral habenular nucleus
LH lateral hypothalamic area
LT lateral thalamus
LEnt lateral entorhinal cortex
mt mammillothalamic tract
MHb medial habenular nucleus
MT medial thalamus
Or oriens layer of the hippocampus
PC paracentral thalamic nucleus
Pir piriform cortex
PVP paraventricular thalamic
Po posterior thalamic nuclear group nucleus, posterior part
PH posterior hypothalamic nucleus
PMV premammillary nucleus, ventral part
PtActx parietal association cortex
RRe retrouniens area
RSCtx retrospenial cortex
SCtx somatosensory cortex
STh subthalamic nucleus
VCtx visual cortex
VM ventromedial thalamic nucleus
VT ventral thalamus
ZI zona incerta

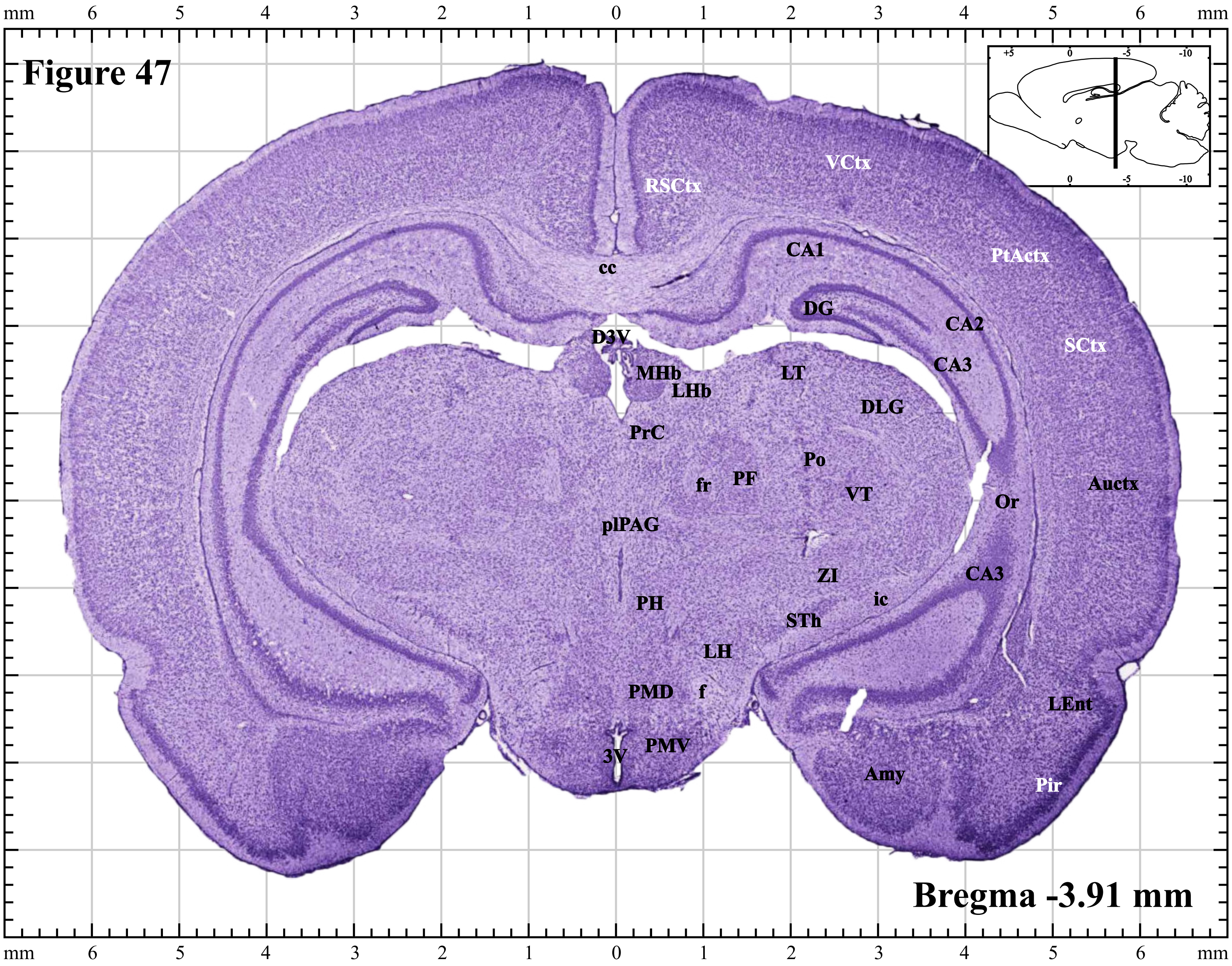

3V medial longitudinal fasciculus
Auctx auditory cortex
Amy amygdaloid nuclei
CA1 field CA1 of the hippocampus
CA2 field CA2 of the hippocampus
CA3 field CA3 of the hippocampus
cc corpus callosum
D3V dorsal 3rd ventricle
DG dentate gyrus

DLG dorsal lateral geniculate nucleus
f fornix
fr fasciculus retroflexus
ic internal capsule
IMD intermediodorsal thalamic nucleus
La lat amygdaloid nucleus
LHb lateral habenular nucleus
LH lateral hypothalamic area
LT lateral thalamus

LEnt lateral entorhinal cortex
mt mammillothalamic tract
MHb medial habenular nucleus
Or oriens layer of the hippocampus
PC paracentral thalamic nucleus
Pir piriform cortex
Po posterior thalamic nuclear group
PMD premammillary nucleus, dorsal part

PVP paraventricular thalamic nucleus, posterior part
PH posterior hypothalamic nucleus
PMV premammillary nucleus, ventral part
PtActx parietal association cortex
PF parafascicular thalamic nucleus
plPAG pleomorphic part of periaqueductai gray

PrC precommissural nucleus
RSCtx retrospenial cortex
SCtx somatosensory cortex
STh subthalamic nucleus
VCtx visual cortex
VM ventromedial thalamic nucleus
VT ventral thalamus
ZI zona incerta

Figure 48

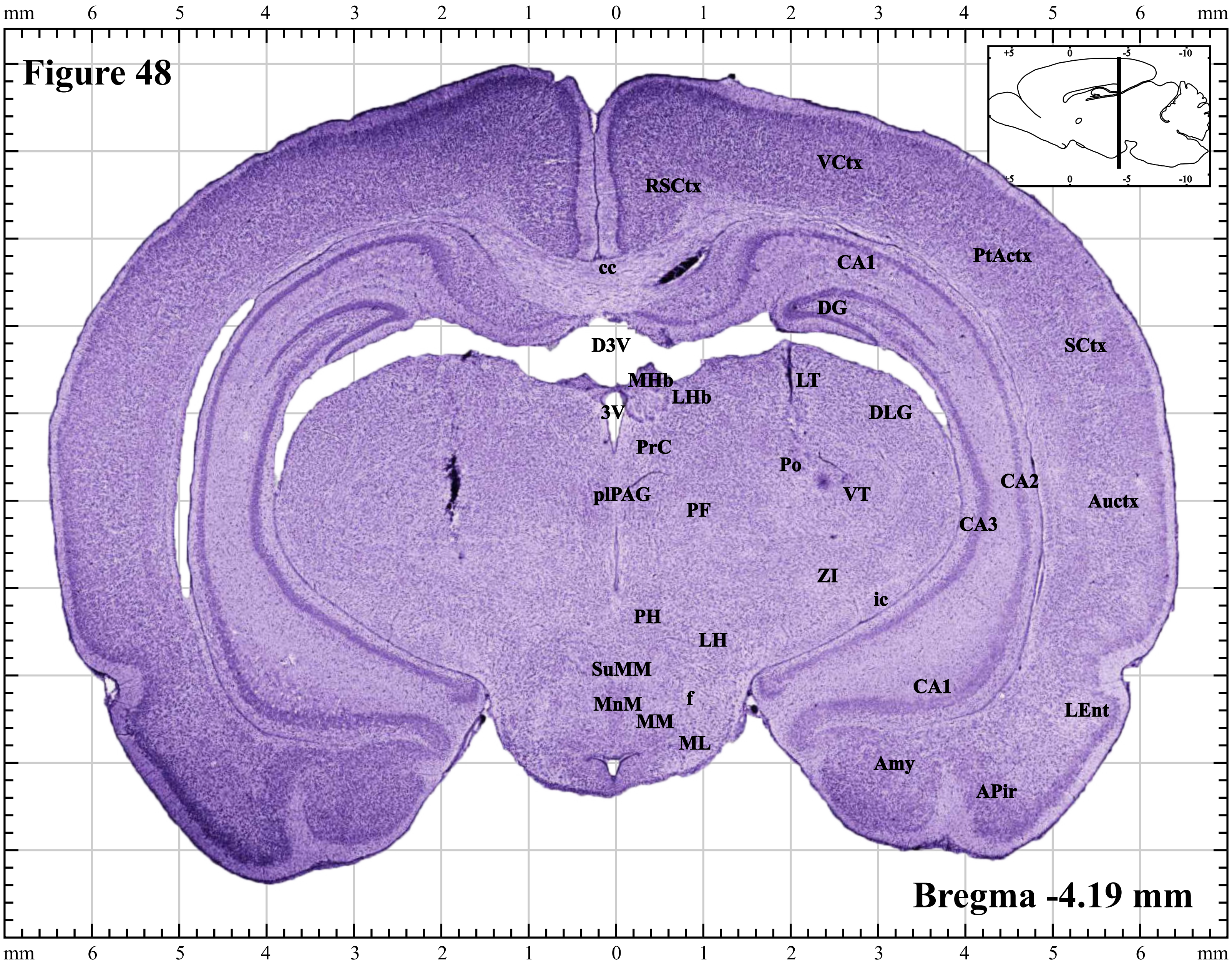

Bregma -4.19 mm

3V medial longitudinal fasciculus
Auctx auditory cortex
Amy amygdaloid nuclei
APir amygdalopiriform transition area
CA1 field CA1 of the hippocampus
CA2 field CA2 of the hippocampus
CA3 field CA3 of the hippocampus
cc corpus callosum
D3V dorsal 3rd ventricle
DG dentate gyrus
DLG dorsal lateral geniculate nucleus
f fornix
ic internal capsule
LHb lateral habenular nucleus
LH lateral hypothalamic area
LT lateral thalamus
LEnt lateral entorhinal cortex
ML medial mammillary nucleus, lateral part
MHb medial habenular nucleus
MnM medial mammillary nucleus, median part
MM medial mammillary nucleus, medial part
PF parafascicular thalamic nucleus
PrC precommissural nucleus
Po posterior thalamic nuclear group
PH posterior hypothalamic nucleus
plPAG pleomorphic part of periaqueductai gray
PtActx parietal association cortex
RSCtx retrospenial cortex
SCtx somatosensory cortex
SuMM supramammillary nucleus, medial part
VCtx visual cortex
VT ventral thalamus
ZI zona incerta

Figure 49

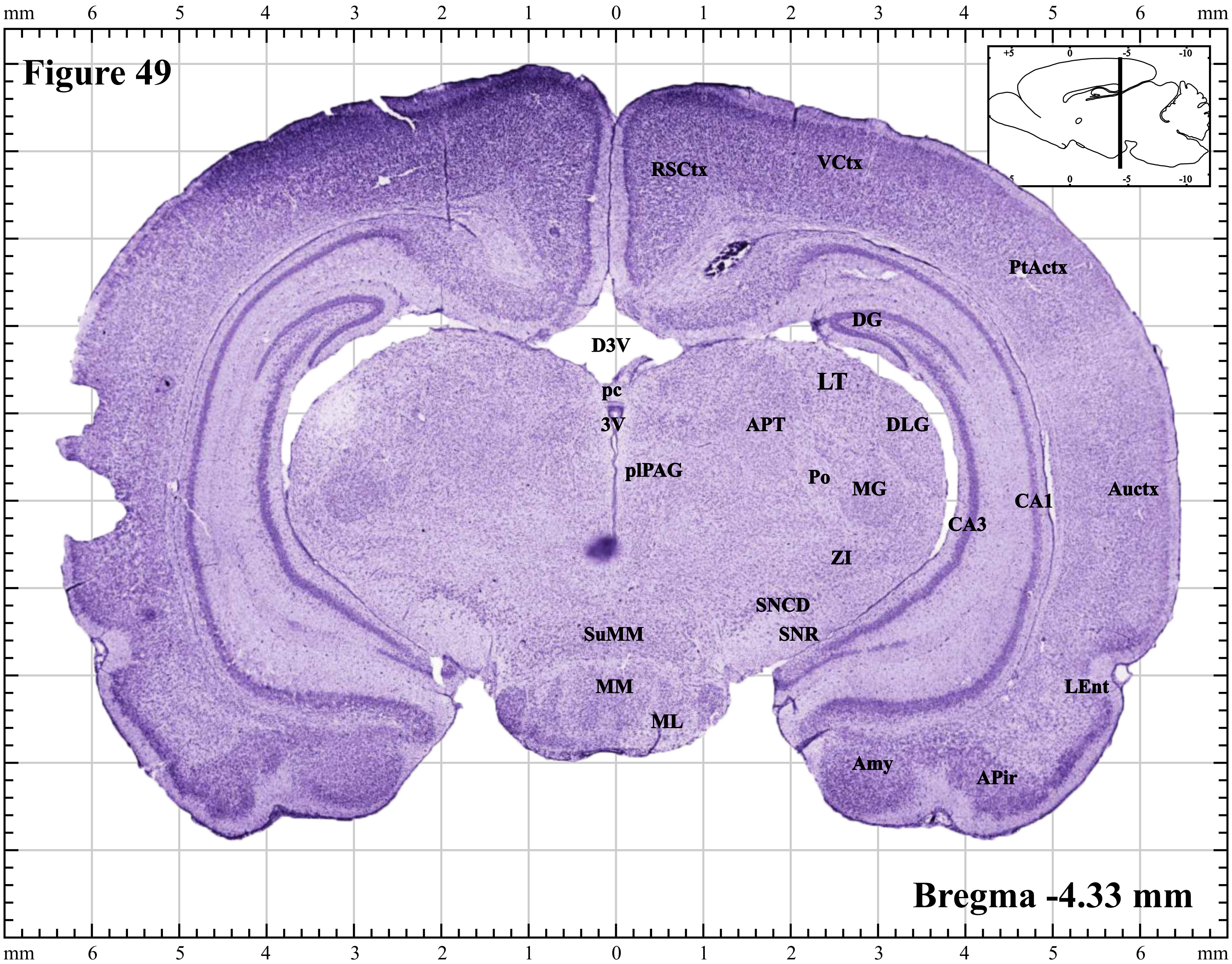

Bregma -4.33 mm

3V medial longitudinal fasciculus
Auctx auditory cortex
Amy amygdaloid nuclei
APir amygdalopiriform transition area
APT anterior pretectal nucleus
CA1 field CA1 of the hippocampus
CA2 field CA2 of the hippocampus
CA3 field CA3 of the hippocampus
D3V dorsal 3rd ventricle
DG dentate gyrus
DLG dorsal lateral geniculate nucleus
f fornix
LT lateral thalamus
LEnt lateral entorhinal cortex
ML medial mammillary nucleus, lateral part
MM medial mammillary nucleus, medial part
MG medial geniculate nucleus
pc posterior commissure
plPAG pleomorphic part of periaqueductai gray
PtActx parietal association cortex
Po posterior thalamic nuclear group
RSCtx retrospenial cortex
SuMM supramammillary nucleus, medial part
SNR substantia nigra, reticular part
SNCD substantia nigra, compact part, dorsal tier
VCtx visual cortex
ZI zona incerta

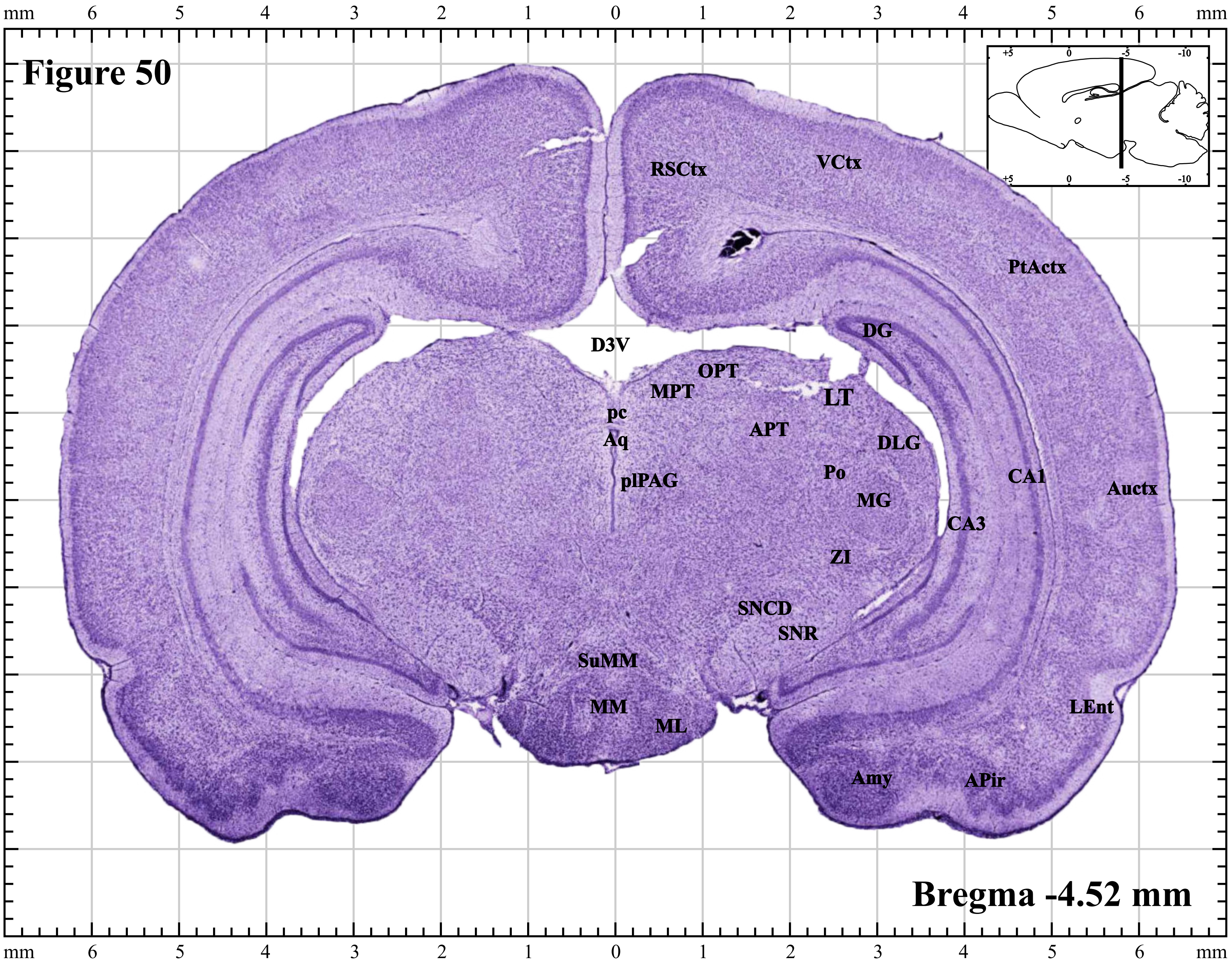

APir amygdalopiriform transition area
Aq aqueduct
Auctx auditory cortex
Amy amygdaloid nuclei
APT anterior pretectal nucleus
CA1 field CA1 of the hippocampus
CA2 field CA2 of the hippocampus
CA3 field CA3 of the hippocampus
D3V dorsal 3rd ventricle
DG dentate gyrus
DLG dorsal lateral geniculate nucleus
f fornix
LT lateral thalamus
LEnt lateral entorhinal cortex
ML medial mammillary nucleus, lateral part
MM medial mammillary nucleus, medial part
MG medial geniculate nucleus
MPT medial pretectal nucleus
OPT olivary pretectal nucleus
pc posterior commissure
plPAG pleomorphic part of periaqueductai gray
PtActx parietal association cortex
Po posterior thalamic nuclear group
RSCtx retrospenial cortex
SuMM supramammillary nucleus, medial part
SNR substantia nigra, reticular part
SNCD substantia nigra, compact part, dorsal tier
VCtx visual cortex
ZI zona incerta

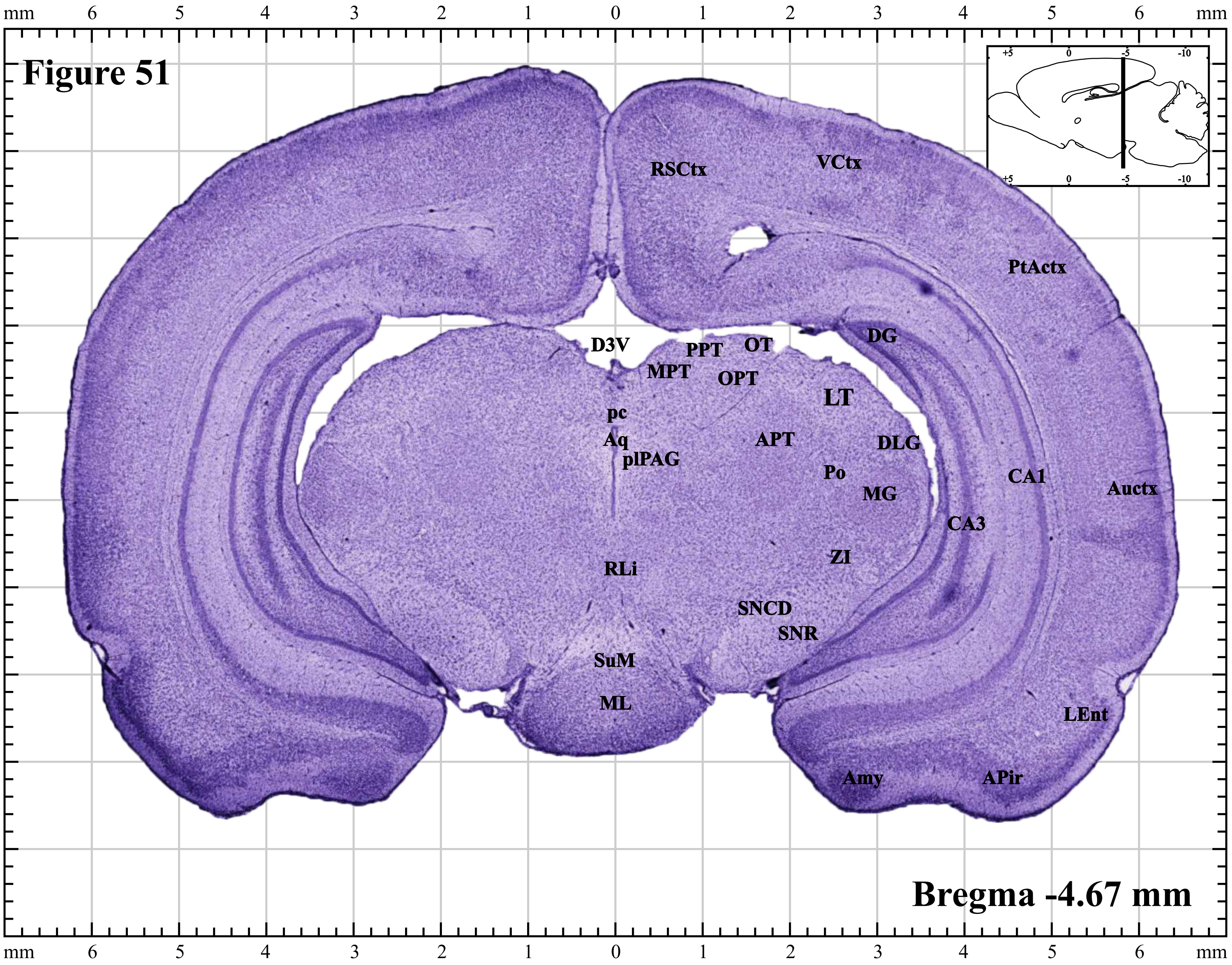

APir amygdalopiriform transition area
Aq aqueduct
Auctx auditory cortex
Amy amygdaloid nuclei
APT anterior pretectal nucleus
CA1 field CA1 of the hippocampus
CA2 field CA2 of the hippocampus
CA3 field CA3 of the hippocampus
D3V dorsal 3rd ventricle
DG dentate gyrus
DLG dorsal lateral geniculate nucleus
f fornix
LT lateral thalamus
LEnt lateral entorhinal cortex
ML medial mammillary nucleus, lateral part
MG medial geniculate nucleus
MPT medial pretectal nucleus
OT nucleus of the optic
OPT olivary pretectal nucleus
pc posterior commissure
plPAG pleomorphic part of periaqueductai gray
PtActx parietal association cortex
Po posterior thalamic nuclear group
PPT posterior pretectal nucleus
RSCtx retrospenial cortex
SuM supramammillary nucleus
SuM supramammillary nucleus
SNR substantia nigra, reticular part
SNCD substantia nigra, compact part, dorsal tier
VCtx visual cortex
ZI zona incerta

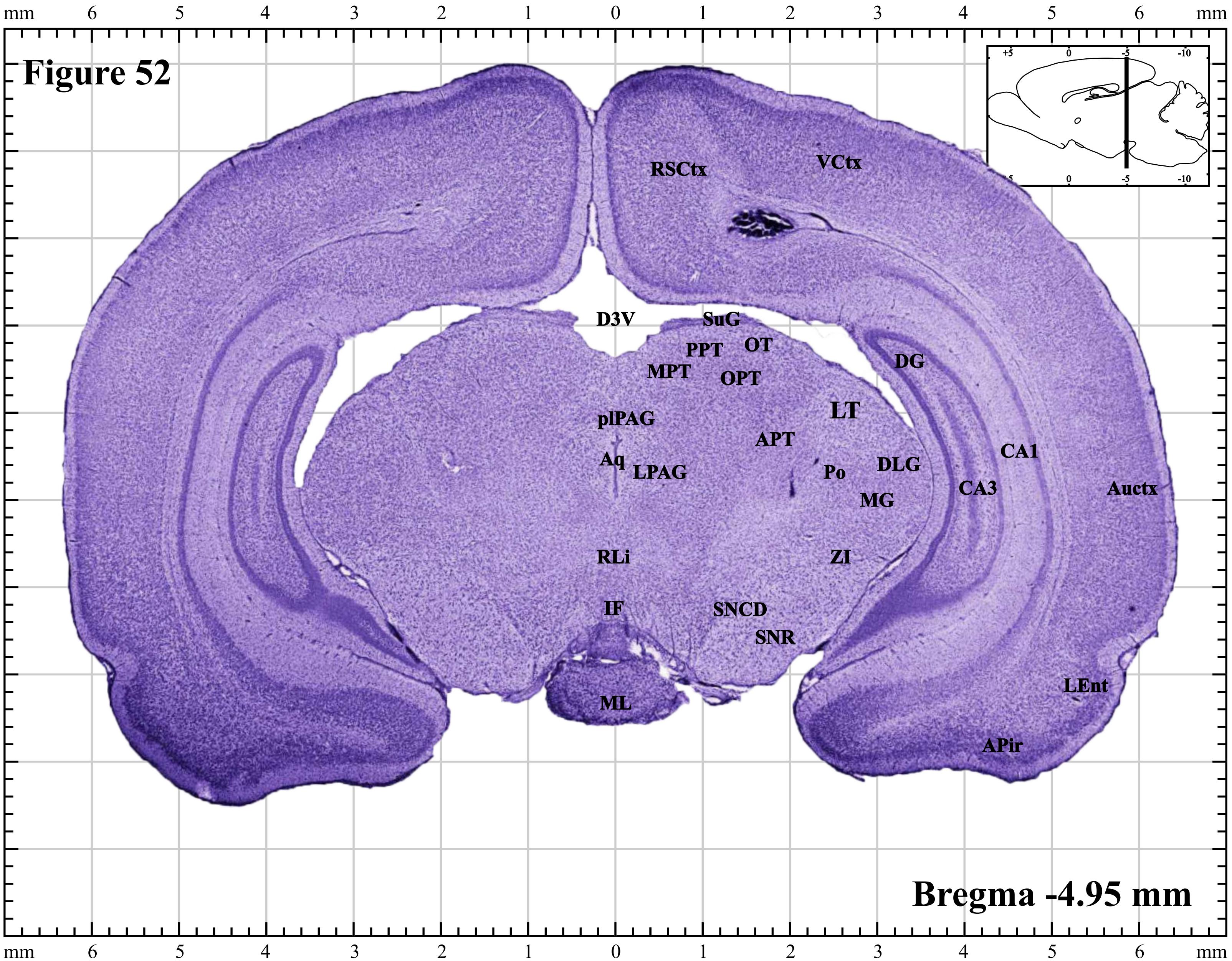

APir amygdalopiriform transition area
Aq aqueduct
Auctx auditory cortex
APT anterior pretectal nucleus
CA1 field CA1 of the hippocampus
CA3 field CA3 of the hippocampus
D3V dorsal 3rd ventricle
DG dentate gyrus
DLG dorsal lateral geniculate nucleus
LPAG lateral periaqueductal gray
IF interfascicular nucleus
LT lateral thalamus
LEnt lateral entorhinal cortex
ML medial mammillary nucleus, lateral part
MG medial geniculate nucleus
MPT medial pretectal nucleus
OT nucleus of the optic
OPT olivary pretectal nucleus
pc posterior commissure
plPAG pleomorphic part of periaqueductai gray
Po posterior thalamic nuclear group
PPT posterior pretectal nucleus
RSCtx retrospenial cortex
RLi rostral linear nucleus of the raphe
SuM supramammillary nucleus
SuG superficial gray layer of the superior colliculus
SNR substantia nigra, reticular part
SNCD substantia nigra, compact part, dorsal tier
VCtx visual cortex
ZI zona incerta

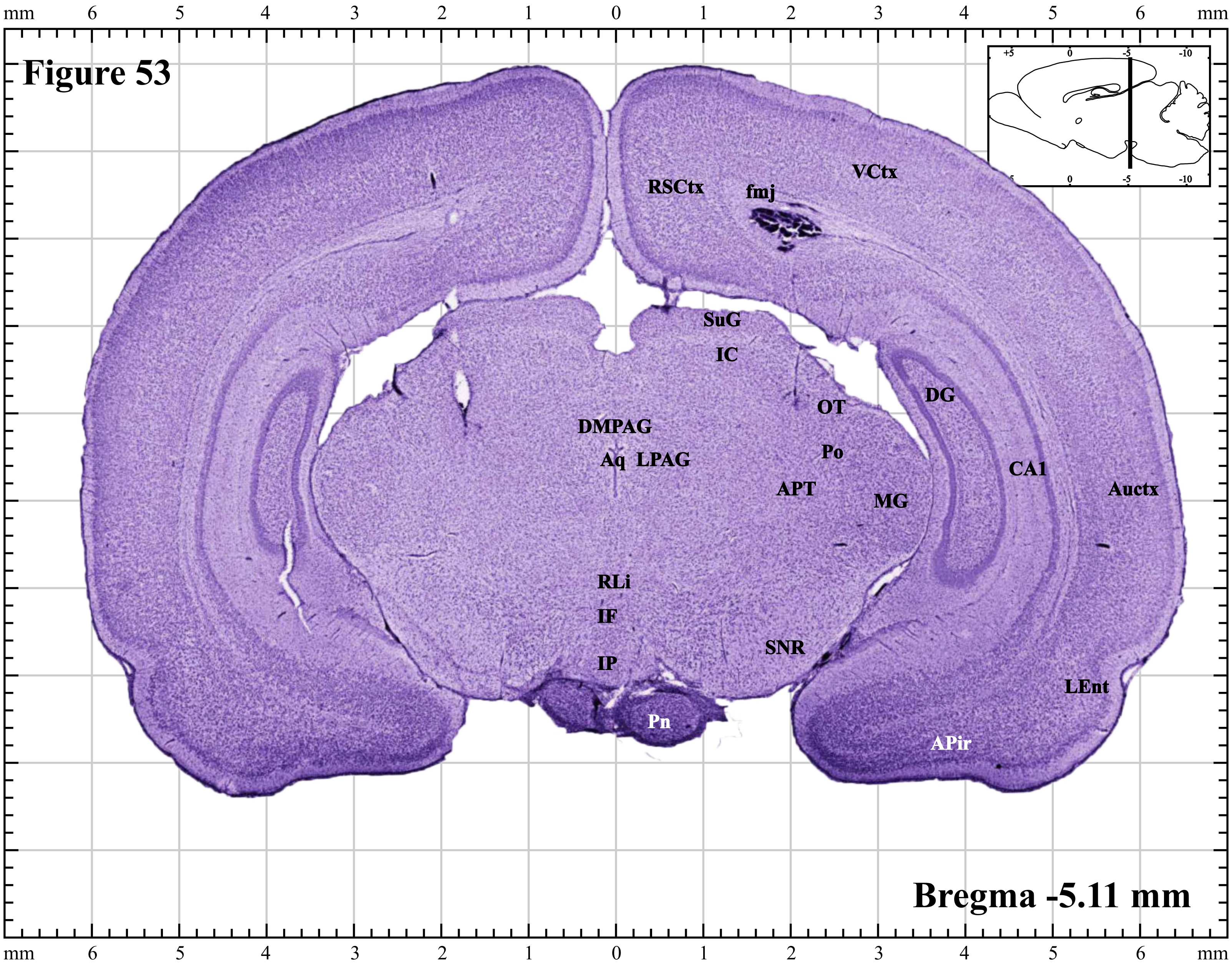

APir amygdalopiriform transition area
Aq aqueduct
Auctx auditory cortex
APT anterior pretectal nucleus
CA1 field CA1 of the hippocampus
DMPAG dorsomedial periaqueductal gray
DG dentate gyrus
fmj forceps major of the corpus callosum
IC inferior colliculus
IF interfascicular nucleus
IP interpeduncular nucleus
LEnt lateral entorhinal cortex
LPAG lateral periaqueductal gray
MG medial geniculate nucleus
OT nucleus of the optic
Po posterior thalamic nuclear group
Pn pontine nuclei
RSCtx retrospenial cortex
RLi rostral linear nucleus of the raphe
SuG superficial gray layer of the superior colliculus
SNR substantia nigra, reticular part
VCtx visual cortex

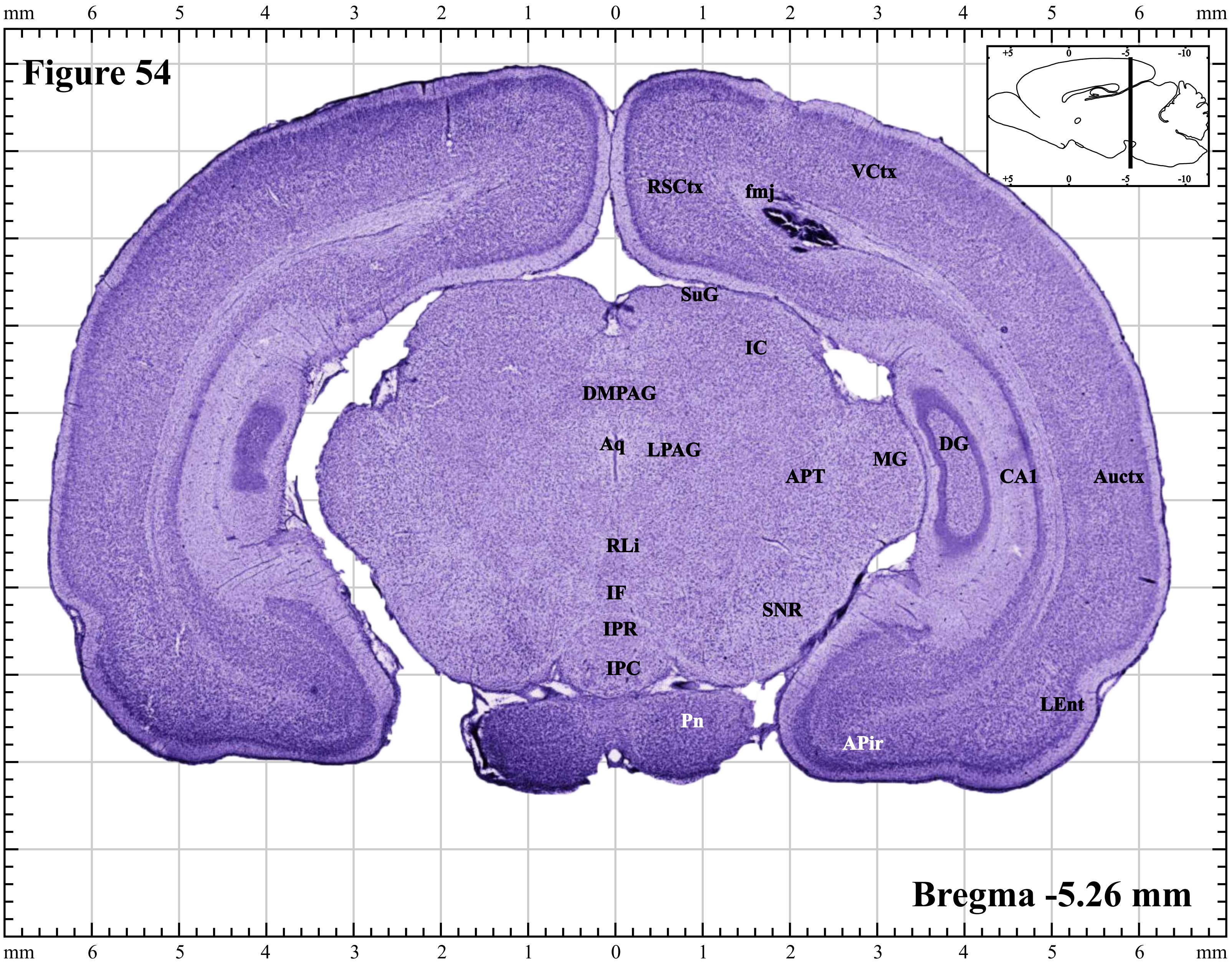

APir amygdalopiriform transition area
Aq aqueduct
Auctx auditory cortex
APT anterior pretectal nucleus
CA1 field CA1 of the hippocampus
DMPAG dorsomedial periaqueductal gray
DG dentate gyrus
fmj forceps major of the corpus callosum
IC inferior colliculus
IF interfascicular nucleus
IPC interpeduncular nucleus, caudal subnucleus
IPR interpeduncular nucleus, rostral subnucleus
LEnt lateral entorhinal cortex
LPAG lateral periaqueductal gray
MG medial geniculate nucleus
Pn pontine nuclei
RSCtx retrospenial cortex
RLi rostral linear nucleus of the raphe
SuG superficial gray layer of the superior colliculus
SNR substantia nigra, reticular part
VCtx visual cortex

Figure 55

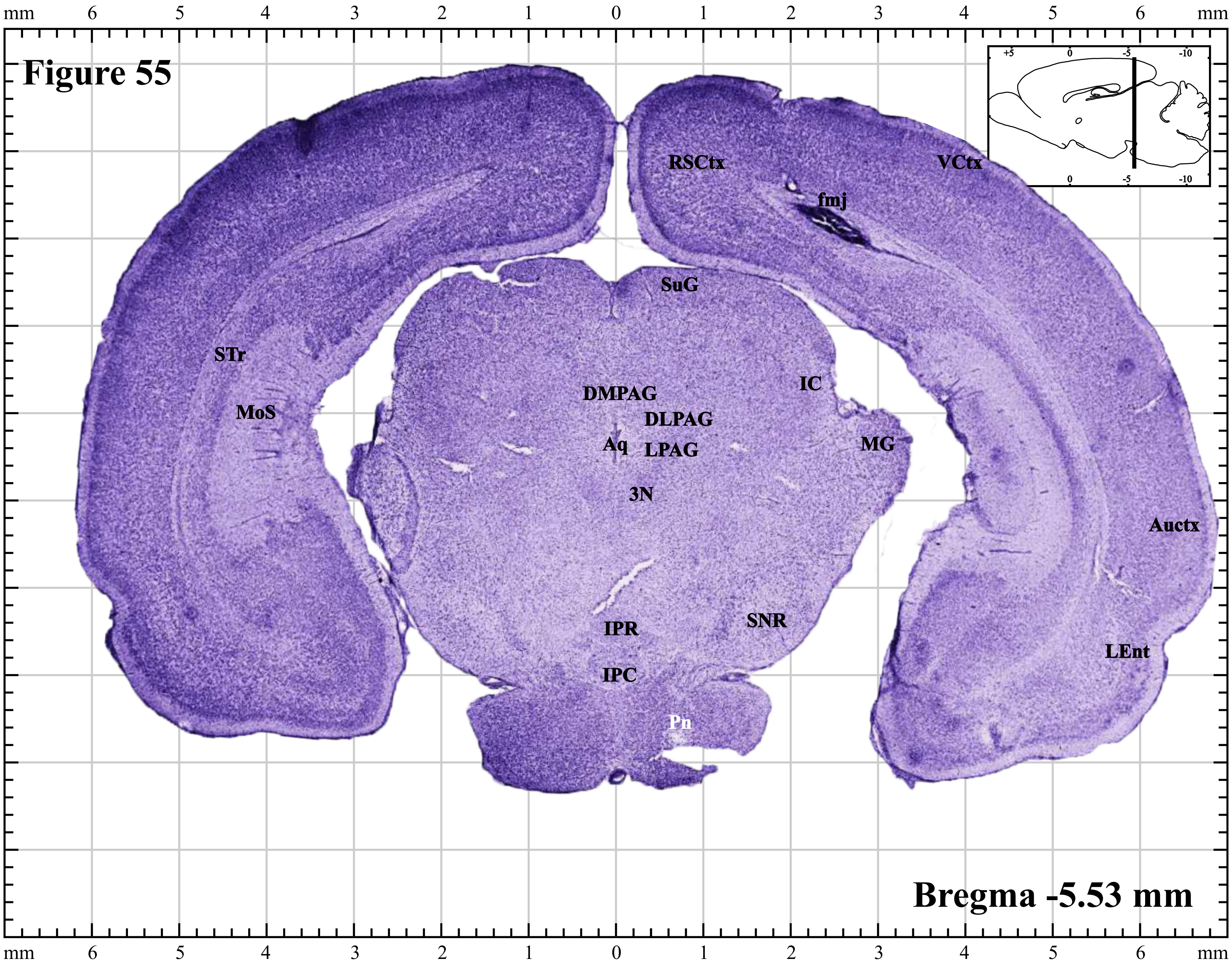

Bregma -5.53 mm

3N oculomotor nucleus
Aq aqueduct
Auctx auditory cortex
DMPAG dorsomedial periaqueductal gray
DLPAG dorsolateral periaqueductal gray
fmj forceps major of the corpus callosum
IC inferior colliculus
IPC interpeduncular nucleus, caudal subnucleus
IPR interpeduncular nucleus, rostral subnucleus
LPAG lateral periaqueductal gray
LEnt lateral entorhinal cortex
MoS molecular layer of the subiculum
MG medial geniculate nucleus
Pn pontine nuclei
RSCtx retrospenial cortex
STr subiculum, transition area
SuG superficial gray layer of the superior colliculus
SNR substantia nigra, reticular part
VCtx visual cortex

Figure 56

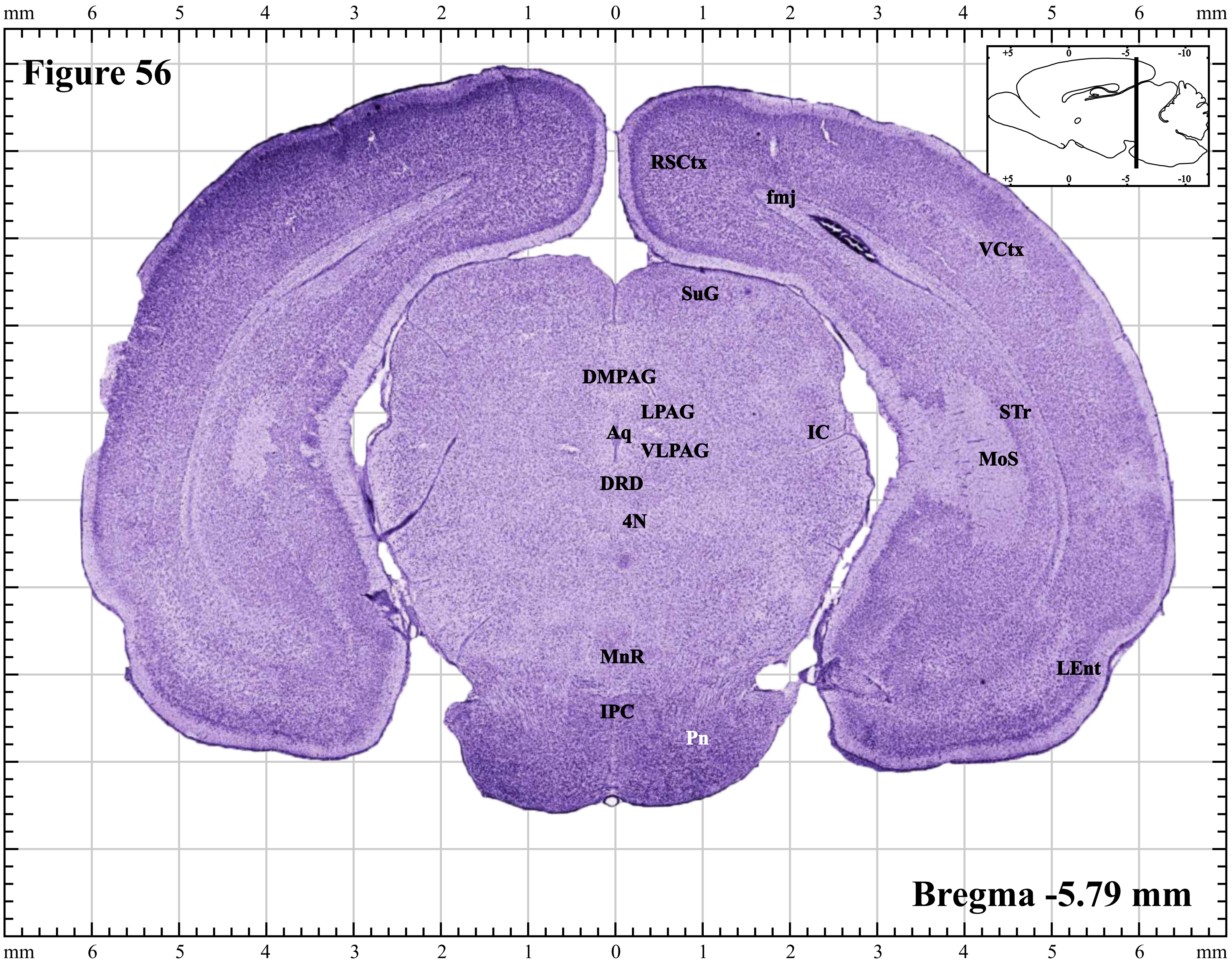

Aq aqueduct
4N trochlear nucleus
DMPAG dorsomedial periaqueductal gray
DRD dorsomedial hypothalamic nucleus, dorsal part
fmj forceps major of the corpus callosum
IC inferior colliculus
IPC interpeduncular nucleus, caudal subnucleus
LEnt lateral entorhinal cortex
LPAG lateral periaqueductal gray
MnR median raphe nucleus
MoS molecular layer of the subiculum
PnO pontine reticular nucleus, oral part
Pn pontine nuclei
RSCtx retrospenial cortex
STr subiculum, transition area
SuG superficial gray layer of the superior colliculus
VCtx visual cortex
VLPAG ventrolateral periaqueductal gray

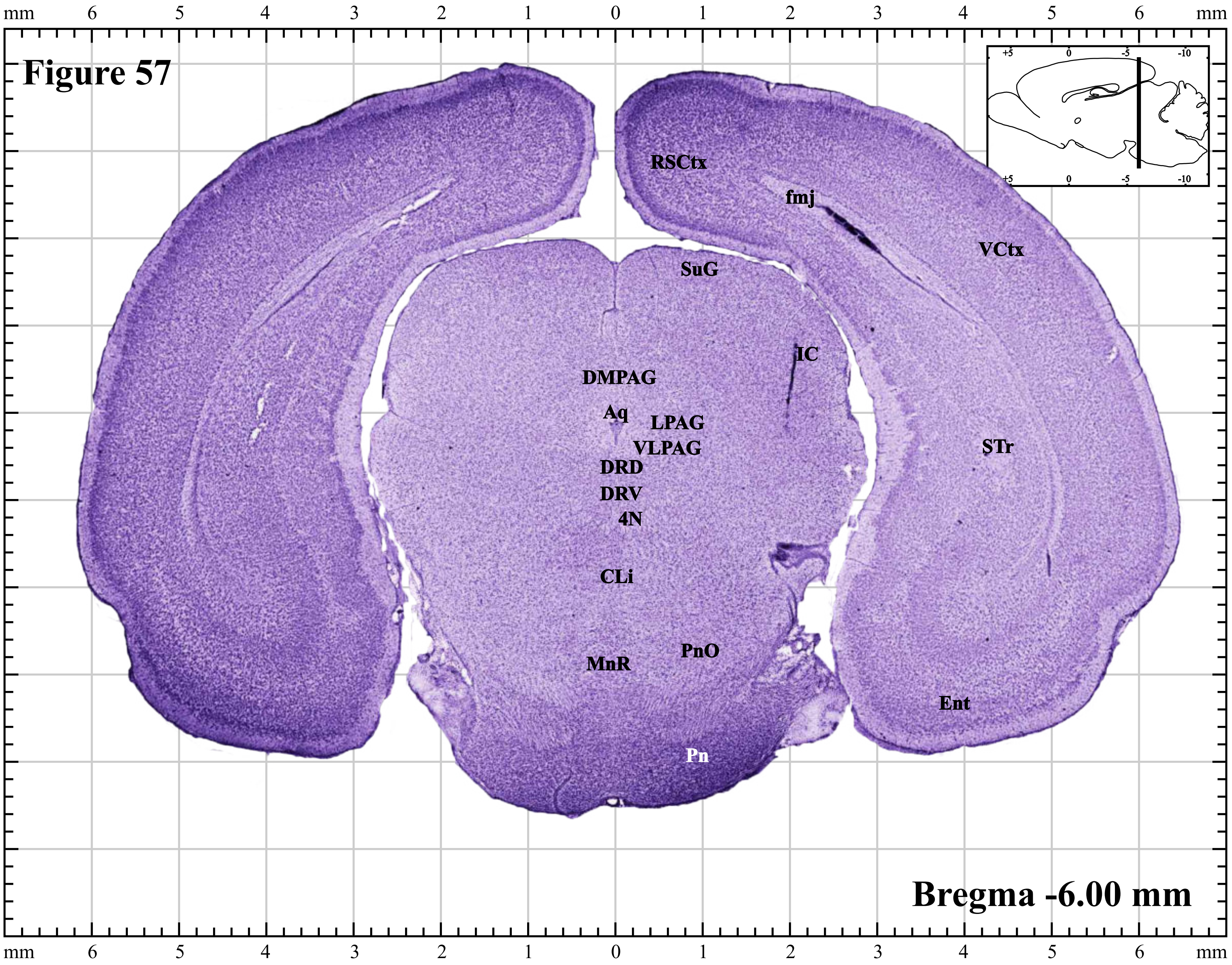

4N trochlear nucleus
Aq aqueduct
CLi caudal linear nucleus of the raphe
DMPAG dorsomedial periaqueductal gray
DRD dorsomedial hypothalamic nucleus, dorsal part
DRV dorsomedial hypothalamic nucleus, ventral part
Ent entorhinal cortex
fmj forceps major of the corpus callosum
IC inferior colliculus
LPAG lateral periaqueductal gray
MnR median raphe nucleus
PnO pontine reticular nucleus, oral part
Pn pontine nuclei
RSCtx retrospenial cortex
STr subiculum, transition area
SuG superficial gray layer of the superior colliculus
VCtx visual cortex
VLPAG ventrolateral periaqueductal gray

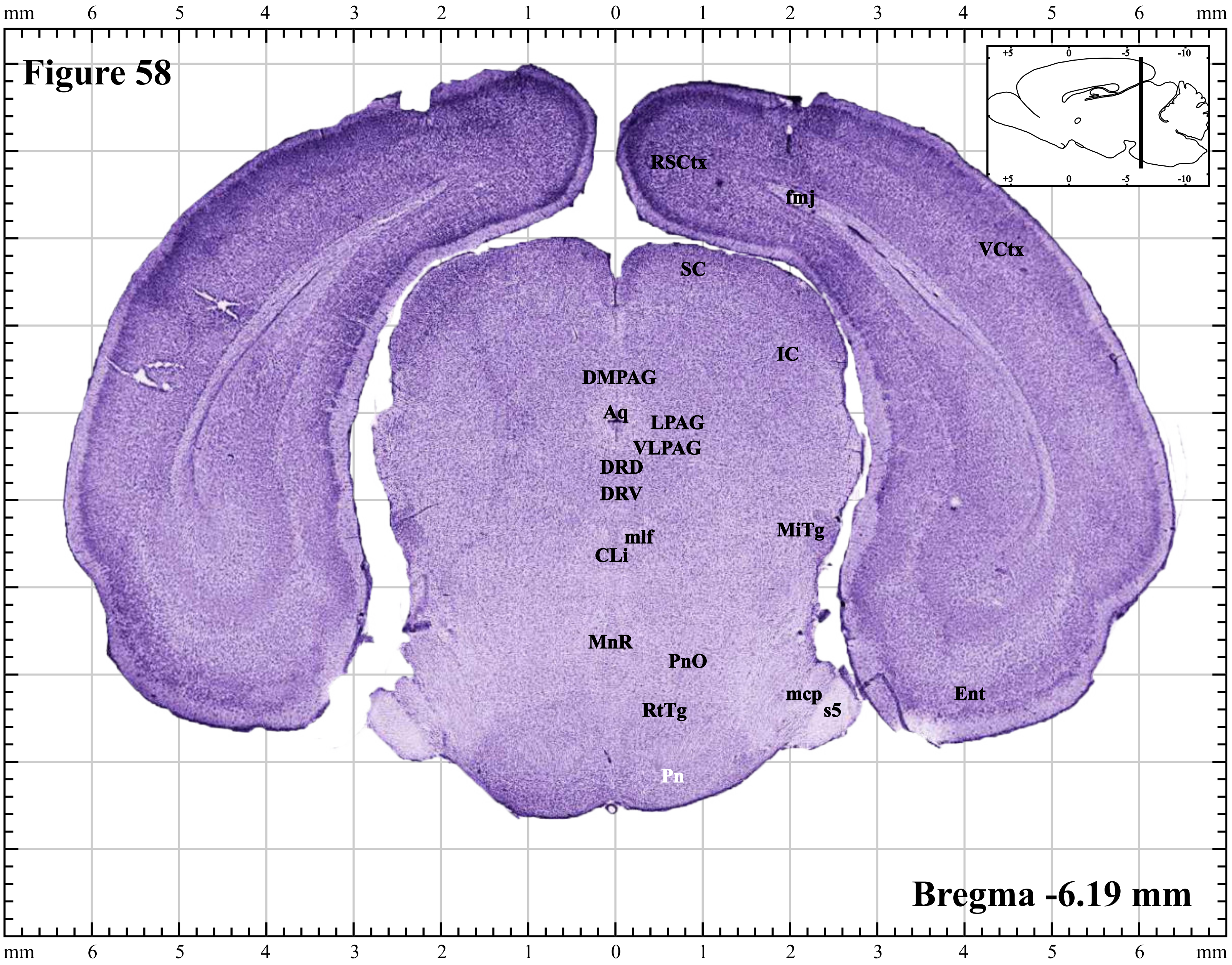

- **Aq** aqueduct
- **CLi** caudal linear nucleus of the raphe
- **DMPAG** dorsomedial periaqueductal gray
- **DRD** dorsomedial hypothalamic nucleus, dorsal part
- **DRV** dorsomedial hypothalamic nucleus, ventral part
- **Ent** entorhinal cortex
- **fmj** forceps major of the corpus callosum
- **IC** inferior colliculus
- **LPAG** lateral periaqueductal gray
- **MnR** median raphe nucleus
- **mlf** medial longitudinal fasciculus
- **mcp** middle cerebellar peduncle
- **MiTg** microcellular tegmental nucleus
- **PnO** pontine reticular nucleus, oral part
- **Pn** pontine nuclei
- **RSCtx** retrospenial cortex
- **RtTg** reticulotegmental nucleus of the pons
- **s5** sensory root of the trigeminal nerve
- **SC** superior colliculus
- **VCtx** visual cortex
- **VLPAG** ventrolateral periaqueductal gray

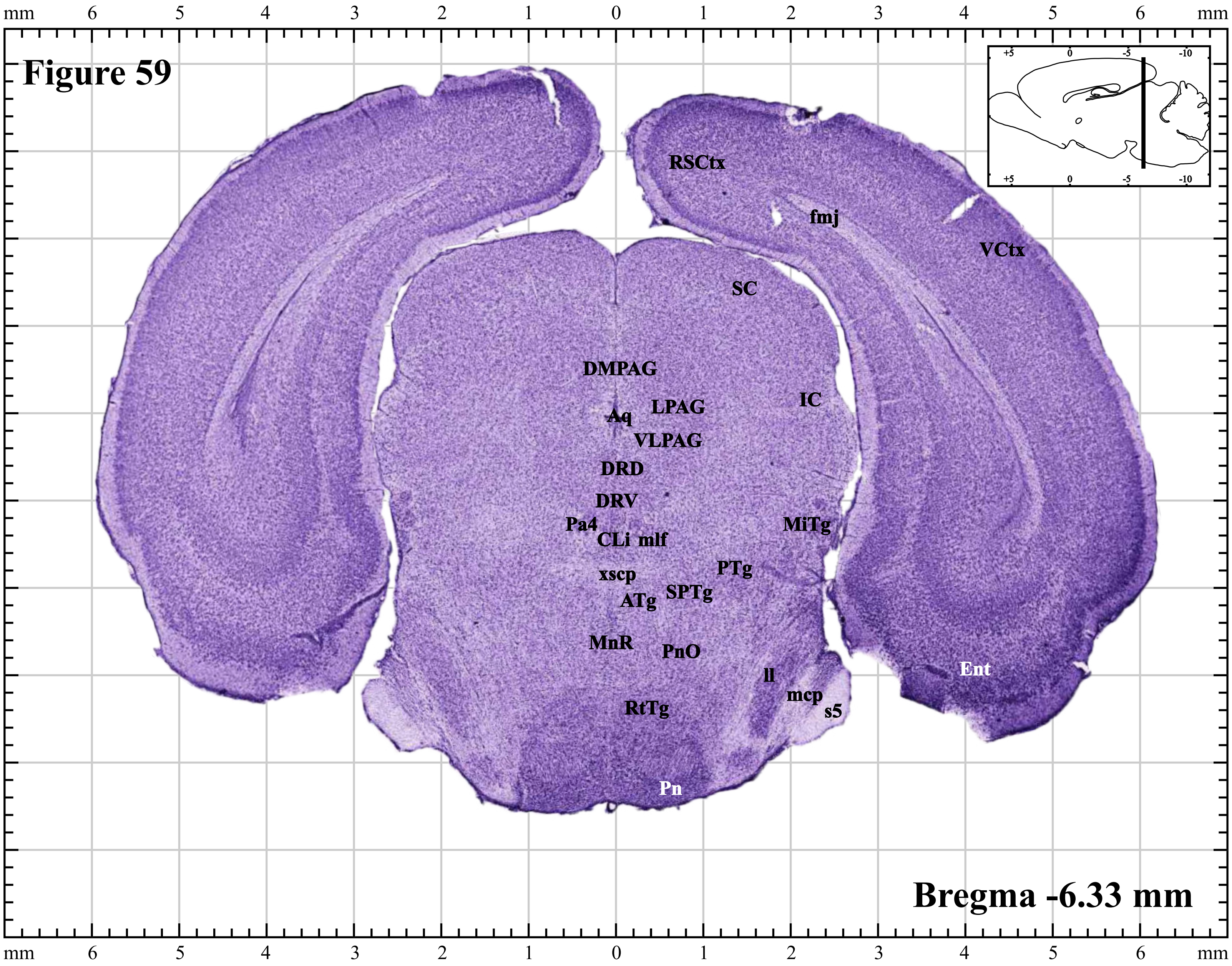

ATg anterior tegmental nucleus
Aq aqueduct
CLi caudal linear nucleus of the raphe
DMPAG dorsomedial periaqueductal gray
DRD dorsomedial hypothalamic nucleus, dorsal part
DRV dorsomedial hypothalamic nucleus, ventral part
Ent entorhinal cortex
fmj forceps major of the corpus callosum
IC inferior colliculus
ll lateral lemniscus
LPAG lateral periaqueductal gray
MnR median raphe nucleus
mlf medial longitudinal fasciculus
mcp middle cerebellar peduncle
MiTg microcellular tegmental nucleus
PnO pontine reticular nucleus, oral part
Pn pontine nuclei
PTg pedunculopontine tegmental nucleus
Pa4 paratrochlear nucleus
RSCtx retrospenial cortex
RtTg reticulotegmental nucleus of the pons
s5 sensory root of the trigeminal nerve
SC superior colliculus
SPTg subpeduncular tegmental nucleus
VCtx visual cortex
VLPAG ventrolateral periaqueductal gray
xscp decussation of the superior cerebellar peduncle

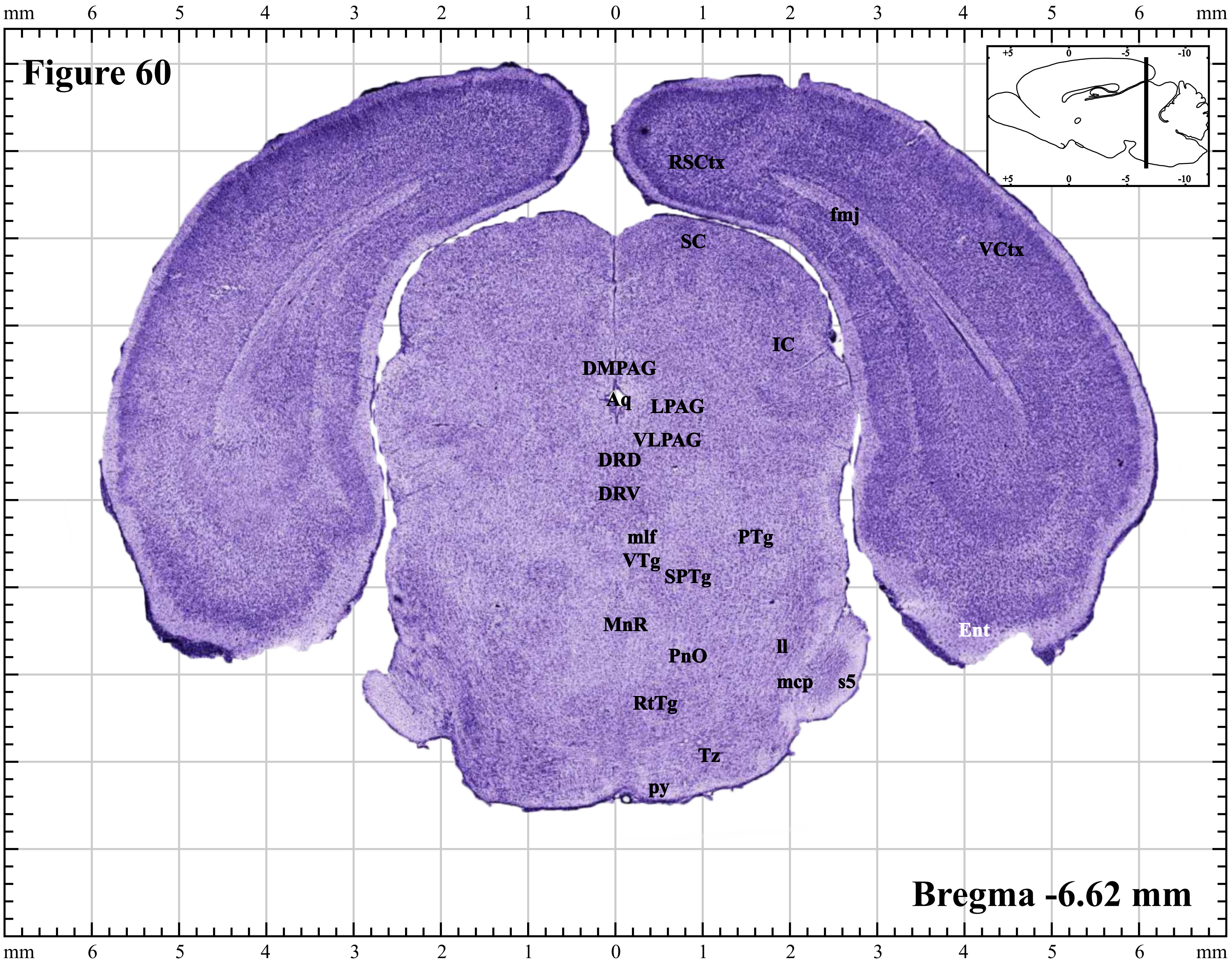

Aq aqueduct
DMPAG dorsomedial periaqueductal gray
DRD dorsomedial hypothalamic nucleus, dorsal part
DRV dorsomedial hypothalamic nucleus, ventral part
Ent entorhinal cortex
fmj forceps major of the corpus callosum
IC inferior colliculus
ll lateral lemniscus
LPAG lateral periaqueductal gray
mlf medial longitudinal fasciculus
mcp middle cerebellar peduncle
MnR median raphe nucleus
py pyramidal tract
PTg pedunculopontine tegmental nucleus
PnO pontine reticular nucleus, oral part
Pn pontine nuclei
RtTg reticulotegmental nucleus of the pons
RSCtx retrospenial cortex
s5 sensory root of the trigeminal nerve
SC superior colliculus
SPTg subpeduncular tegmental nucleus
Tz nucleus of the trapezoid body
VCtx visual cortex
VLPAG ventrolateral periaqueductal gray
VTg ventral tegmental nucleus

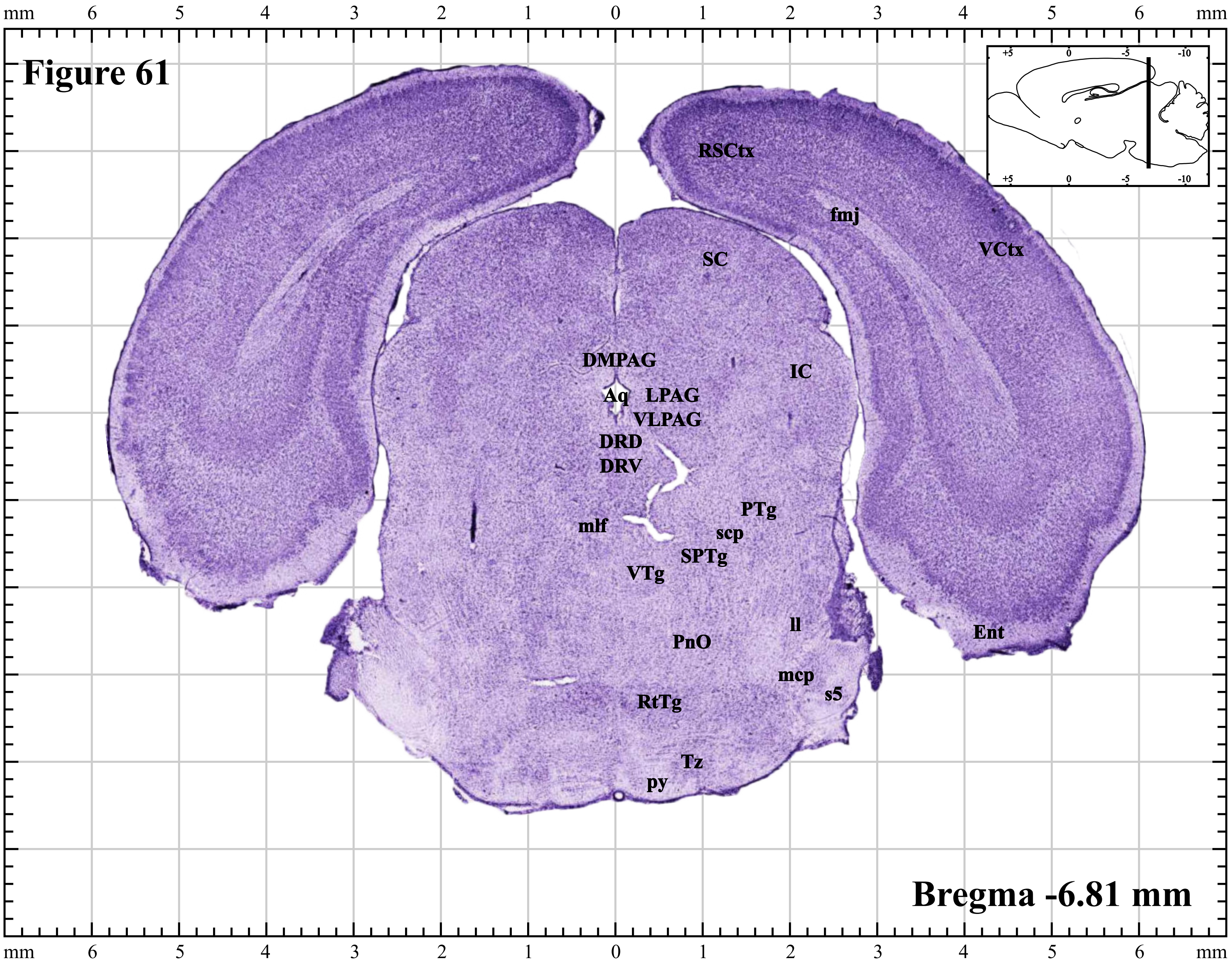

Aq aqueduct
DMPAG dorsomedial periaqueductal gray
DRD dorsomedial hypothalamic nucleus, dorsal part
DRV dorsomedial hypothalamic nucleus, ventral part
Ent entorhinal cortex
fmj forceps major of the corpus callosum
IC inferior colliculus
ll lateral lemniscus
LPAG lateral periaqueductal gray
mlf medial longitudinal fasciculus
mcp middle cerebellar peduncle
py pyramidal tract
PTg pedunculopontine tegmental nucleus
PnO pontine reticular nucleus, oral part
RSCtx retrospenial cortex
s5 sensory root of the trigeminal nerve
scp superior cerebellar peduncle
SPTg subpeduncular tegmental nucleus
SC superior colliculus
RtTg reticulotegmental nucleus of the pons
Tz nucleus of the trapezoid body
VCtx visual cortex
VLPAG ventrolateral periaqueductal gray
VTg ventral tegmental nucleus

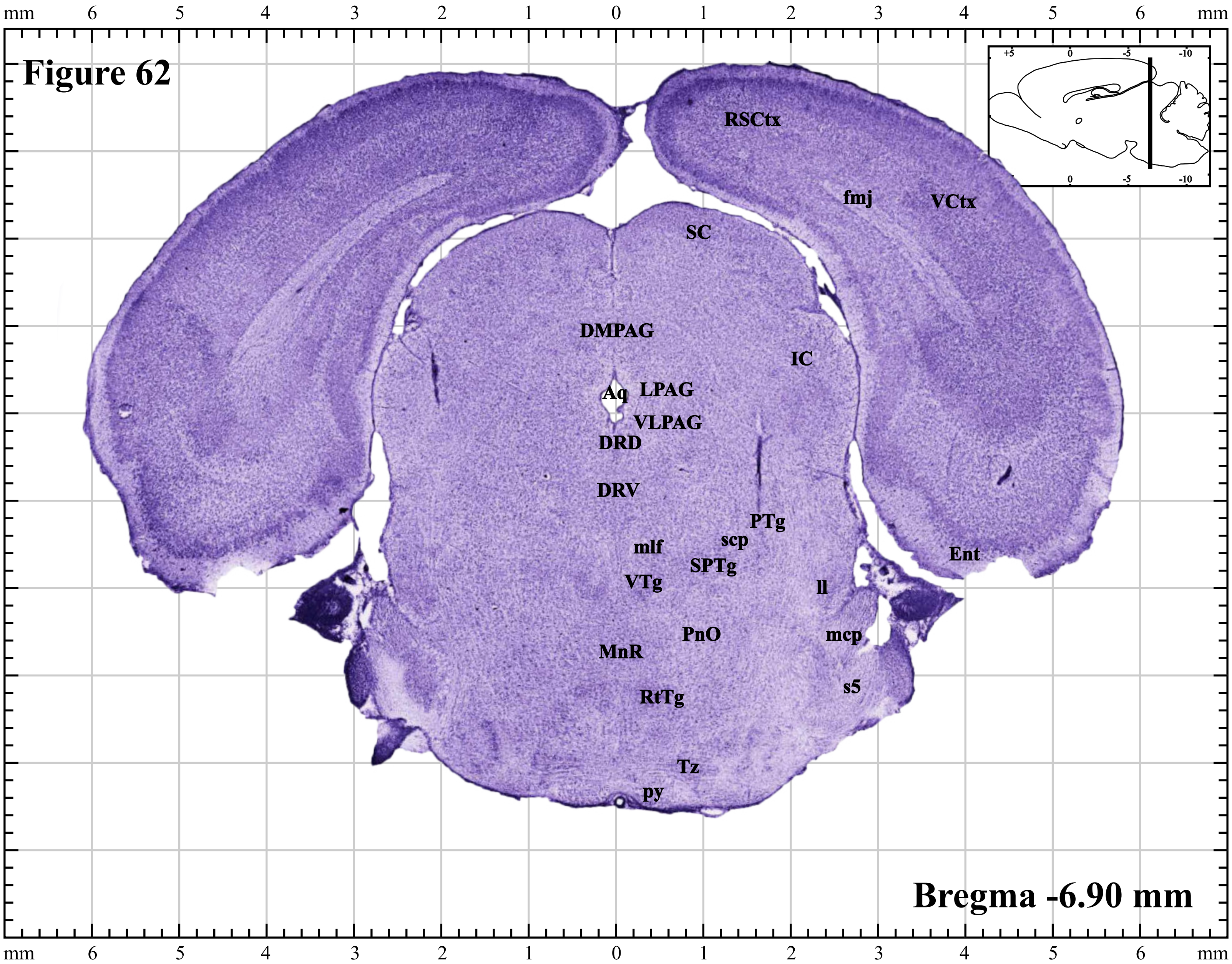

Figure 62

Bregma -6.90 mm

Aq aqueduct
DMPAG dorsomedial periaqueductal gray
DRD dorsomedial hypothalamic nucleus, dorsal part
DRV dorsomedial hypothalamic nucleus, ventral part
Ent entorhinal cortex
fmj forceps major of the corpus callosum
IC inferior colliculus
ll lateral lemniscus
LPAG lateral periaqueductal gray
mlf medial longitudinal fasciculus
mcp middle cerebellar peduncle
MnR median raphe nucleus
py pyramidal tract
PnO pontine reticular nucleus, oral part
PTg pedunculopontine tegmental nucleus
RSCtx retrospenial cortex
RtTg reticulotegmental nucleus of the pons
s5 sensory root of the trigeminal nerve
scp superior cerebellar peduncle
SC superior colliculus
SPTg subpeduncular tegmental nucleus
Tz nucleus of the trapezoid body
VCtx visual cortex
VLPAG ventrolateral periaqueductal gray
VTg ventral tegmental nucleus

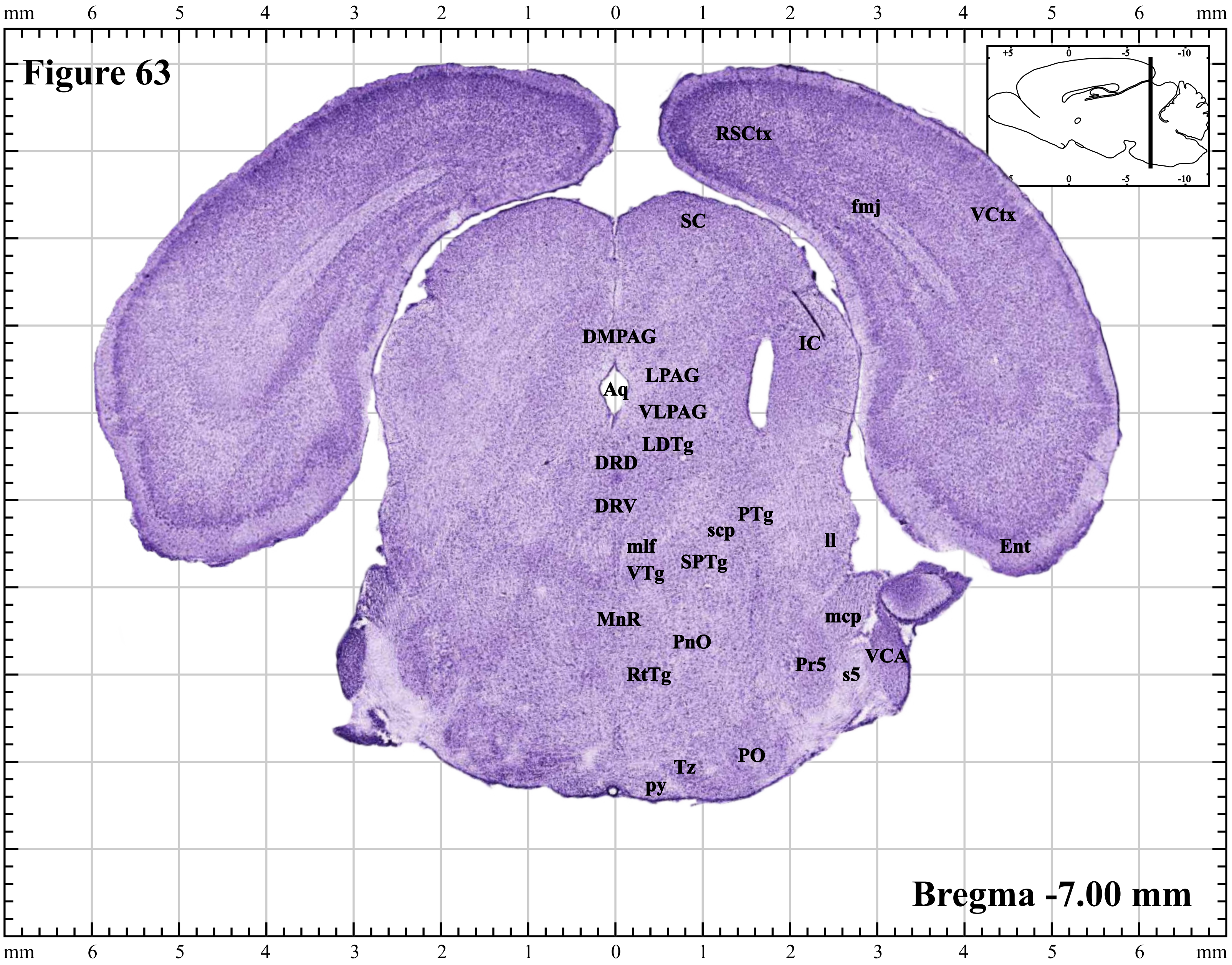

Aq aqueduct
DMPAG dorsomedial periaqueductal gray
DRD dorsomedial hypothalamic nucleus, dorsal part
DRV dorsomedial hypothalamic nucleus, ventral part
Ent entorhinal cortex
fmj forceps major of the corpus callosum
IC inferior colliculus
ll lateral lemniscus
LDTg laterodorsal tegmental nucleus
LPAG lateral periaqueductal gray
mlf medial longitudinal fasciculus
mcp middle cerebellar peduncle
MnR median raphe nucleus
py pyramidal tract
PO paraolivary nucleus
PTg pedunculopontine tegmental nucleus
PnO pontine reticular nucleus, oral part
Pr5 principal sensory trigeminal nucleus
RtTg reticulotegmental nucleus of the pons
RSCtx retrospenial cortex
s5 sensory root of the trigeminal nerve
scp superior cerebellar peduncle
SC superior colliculus
SPTg subpeduncular tegmental nucleus
Tz nucleus of the trapezoid body
VCtx visual cortex
VLPAG ventrolateral periaqueductal gray
VCA ventral cochlear nucleus, anterior part
VTg ventral tegmental nucleus

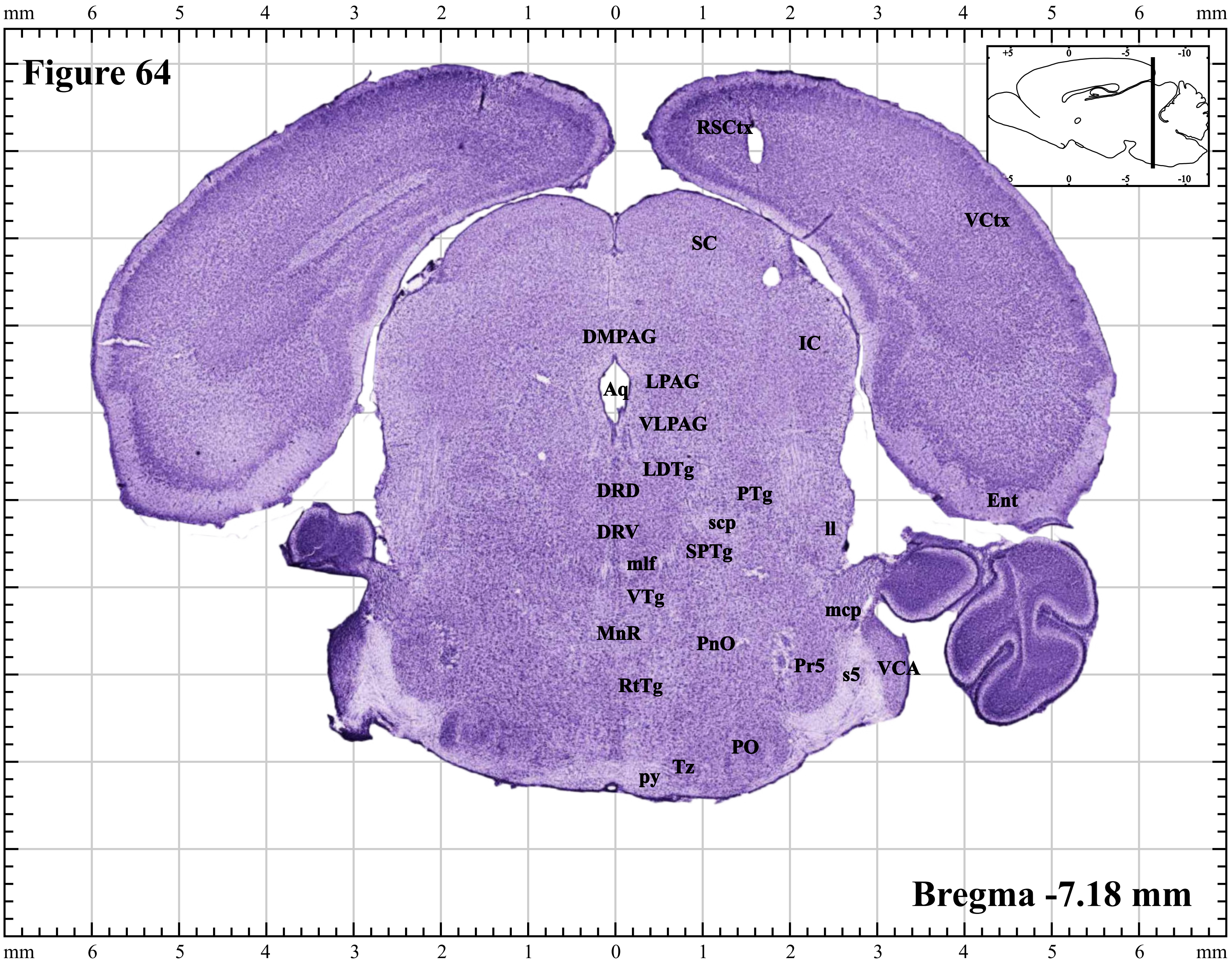

Aq aqueduct
DMPAG dorsomedial periaqueductal gray
DRD dorsomedial hypothalamic nucleus, dorsal part
DRV dorsomedial hypothalamic nucleus, ventral part
Ent entorhinal cortex
IC inferior colliculus
ll lateral lemniscus
LDTg laterodorsal tegmental nucleus
LPAG lateral periaqueductal gray
mlf medial longitudinal fasciculus
mcp middle cerebellar peduncle
MnR median raphe nucleus
py pyramidal tract
Pn pontine nuclei
PnO pontine reticular nucleus, oral part
Pr5 principal sensory trigeminal nucleus
PO paraolivary nucleus
PTg pedunculopontine tegmental nucleus
RSCtx retrospenial cortex
RtTg reticulotegmental nucleus of the pons
s5 sensory root of the trigeminal nerve
scp superior cerebellar peduncle
SPTg subpeduncular tegmental nucleus
SC superior colliculus
Tz nucleus of the trapezoid body
VCtx visual cortex
VLPAG ventrolateral periaqueductal gray
VCA ventral cochlear nucleus, anterior part
VTg ventral tegmental nucleus

Figure 65

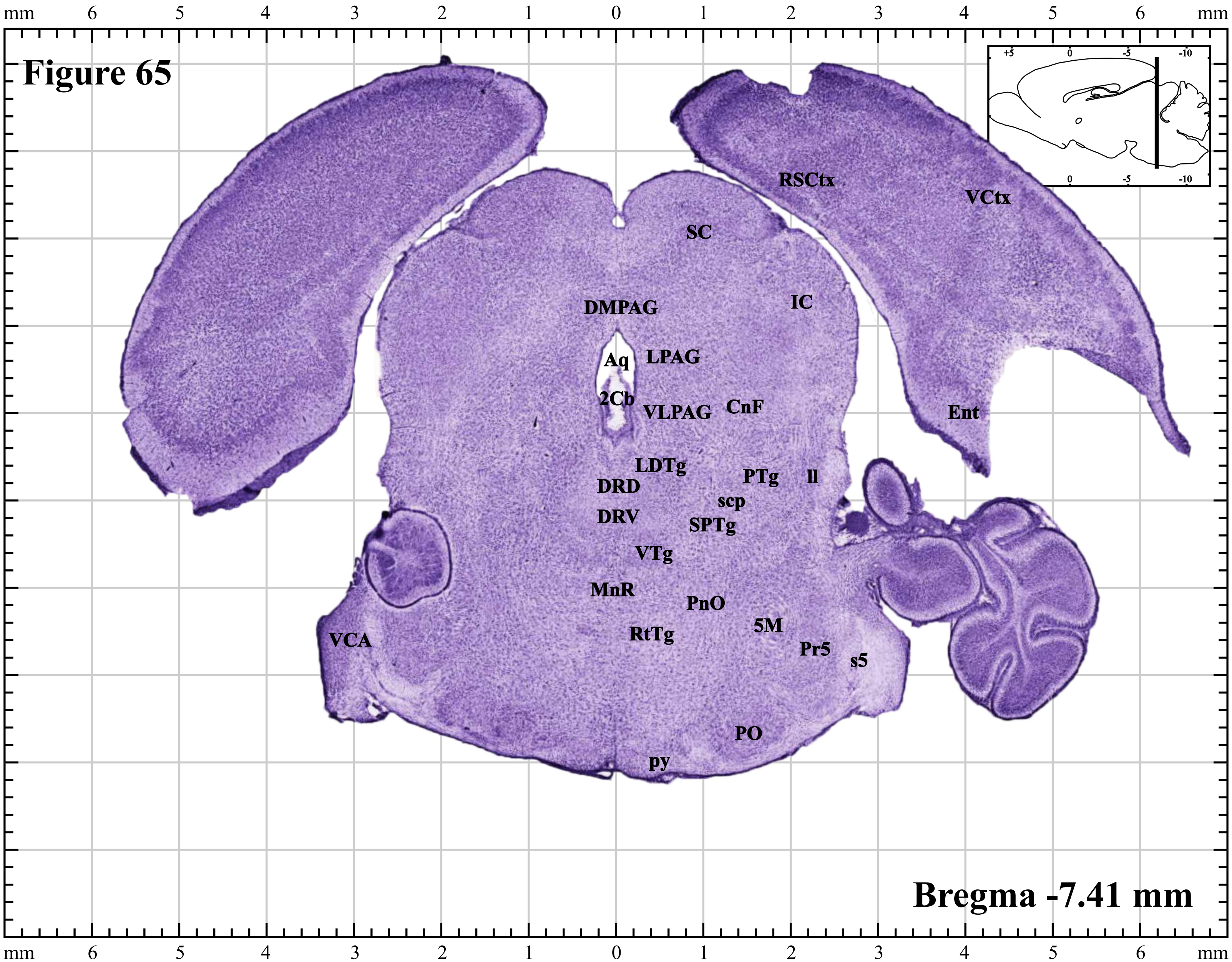

Bregma -7.41 mm

5M motor trigeminal nucleus
2Cb 2nd cerebellar lobule
Aq aqueduct
CnF cuneiform nucleus
DMPAG dorsomedial periaqueductal gray
DRD dorsomedial hypothalamic nucleus, dorsal part
DRV dorsomedial hypothalamic nucleus, ventral part
Ent entothinal cortex
IC inferior colliculus
ll lateral lemniscus
LDTg laterodorsal tegmental nucleus
LPAG lateral periaqueductal gray
MnR median raphe nucleus
py pyramidal tract
Pr5 principal sensory trigeminal nucleus
PO paraolivary nucleus
PnO pontine reticular nucleus, oral part
PTg pedunculopontine tegmental nucleus
RSCtx retrospenial cortex
RtTg reticulotegmental nucleus of the pons
scp superior cerebellar peduncle
s5 sensory root of the trigeminal nerve
SC superior colliculus
SPTg subpeduncular tegmental nucleus
VCtx visual cortex
VLPAG ventrolateral periaqueductal gray
VCA ventral cochlear nucleus, anterior part
VTg ventral tegmental nucleus

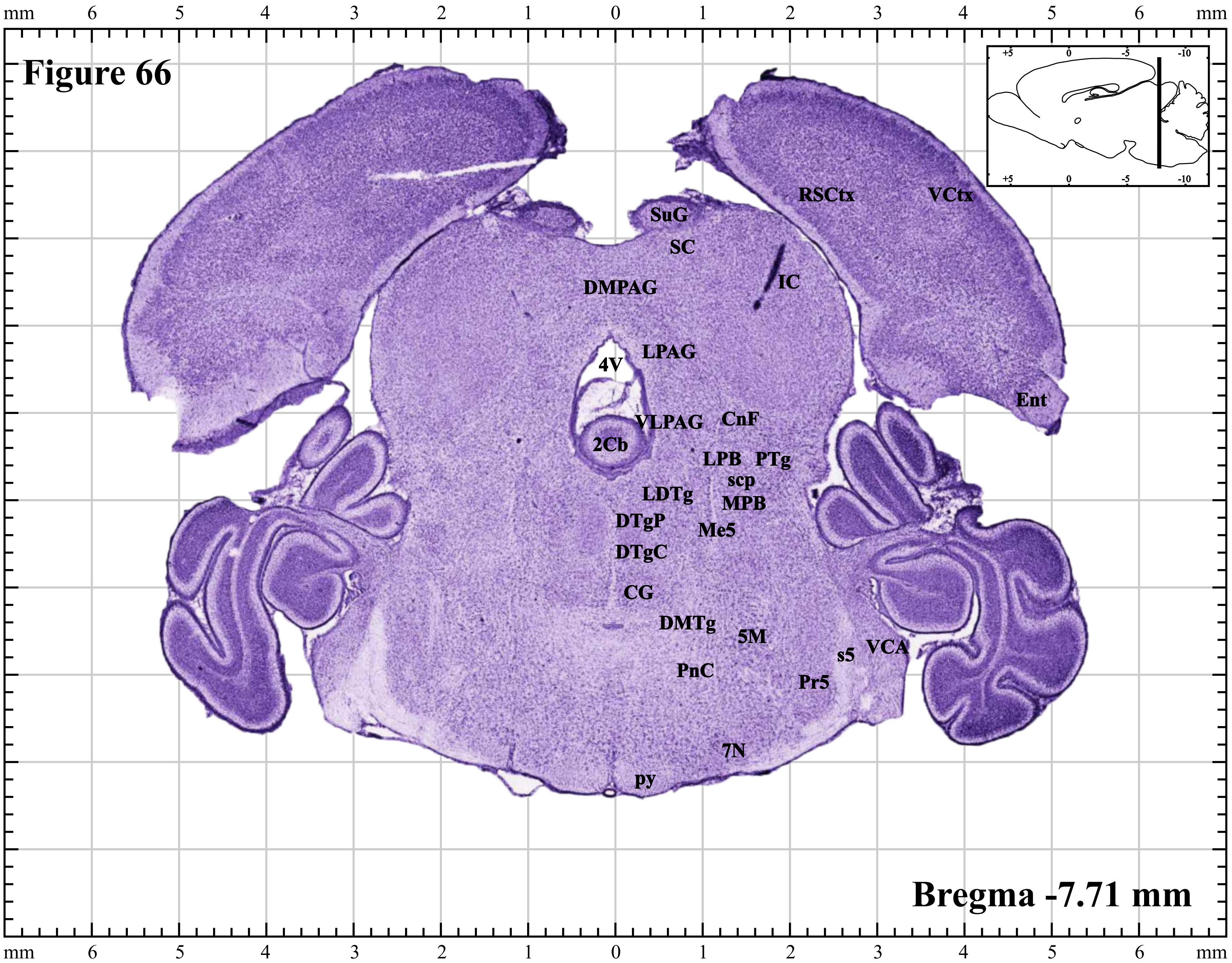

5M motor trigeminal nucleus
7N facial nuclues
2Cb 2nd cerebellar lobule
4V 4th ventricle
CG central gray
CnF cuneiform nucleus
DMTg dorsomedial tegmental area
DMPAG dorsomedial periaqueductal gray
DTgP dorsal tegmental nucleus, pericentral part
DTgC dorsal tegmental nucleus, central part
Ent entorhinal cortex
IC inferior colliculus
ll lateral lemniscus
LDTg laterodorsal tegmental nucleus
LPAG lateral periaqueductal gray
LPB lateral parabrachial nucleus
MPB medial parabrachial nucleus
Me5 mesencephalic trigeminal nucleus
py pyramidal tract
PTg pedunculopontine tegmental nucleus
PnC pontine reticular nucleus,caudal part
Pr5 principal sensory trigeminal nucleus
RSCtx retrospenial cortex
scp superior cerebellar peduncle
s5 sensory root of the trigeminal nerve
SuG superficial gray layer of the superior colliculus
SC superior colliculus
VCtx visual cortex
VLPAG ventrolateral periaqueductal gray
VCA ventral cochlear nucleus, anterior part

Figure 67

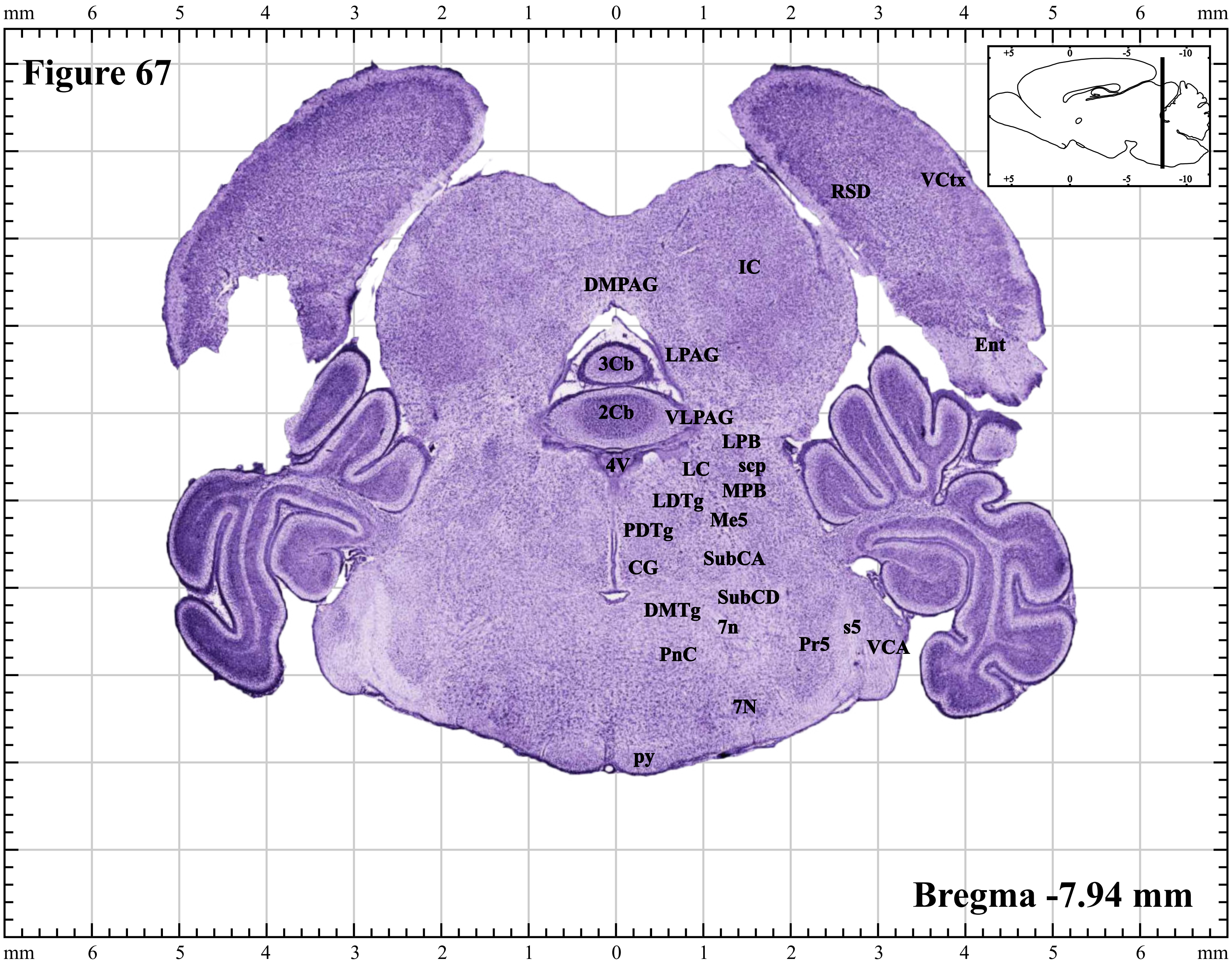

Bregma -7.94 mm

7n facial nerve
7N facial nuclues
2Cb 2nd cerebellar lobule
3Cb 3rd cerebellar lobule
4V 4th ventricle
CG central gray
DMPAG dorsomedial periaqueductal gray
DMTg dorsomedial tegmental area
Ent entorhinal cortex
IC inferior colliculus
LC locus coeruleus
LDTg laterodorsal tegmental nucleus
LPAG lateral periaqueductal gray
LPB lateral parabrachial nucleus
Me5 mesencephalic trigeminal nucleus
MPB medial parabrachial nucleus
py pyramidal tract
PnC pontine reticular nucleus, caudal part
PDTg posterodorsal tegmental nucleus
Pr5 principal sensory trigeminal nucleus
RSCtx retrospenial cortex
s5 sensory root of the trigeminal nerve
SubCA subcoeruleus nucleus, alpha part
SubCD subcoeruleus nucleus, dorsal part
VCtx visual cortex
VLPAG ventrolateral periaqueductal gray
VCA ventral cochlear nucleus, anterior part

Figure 68

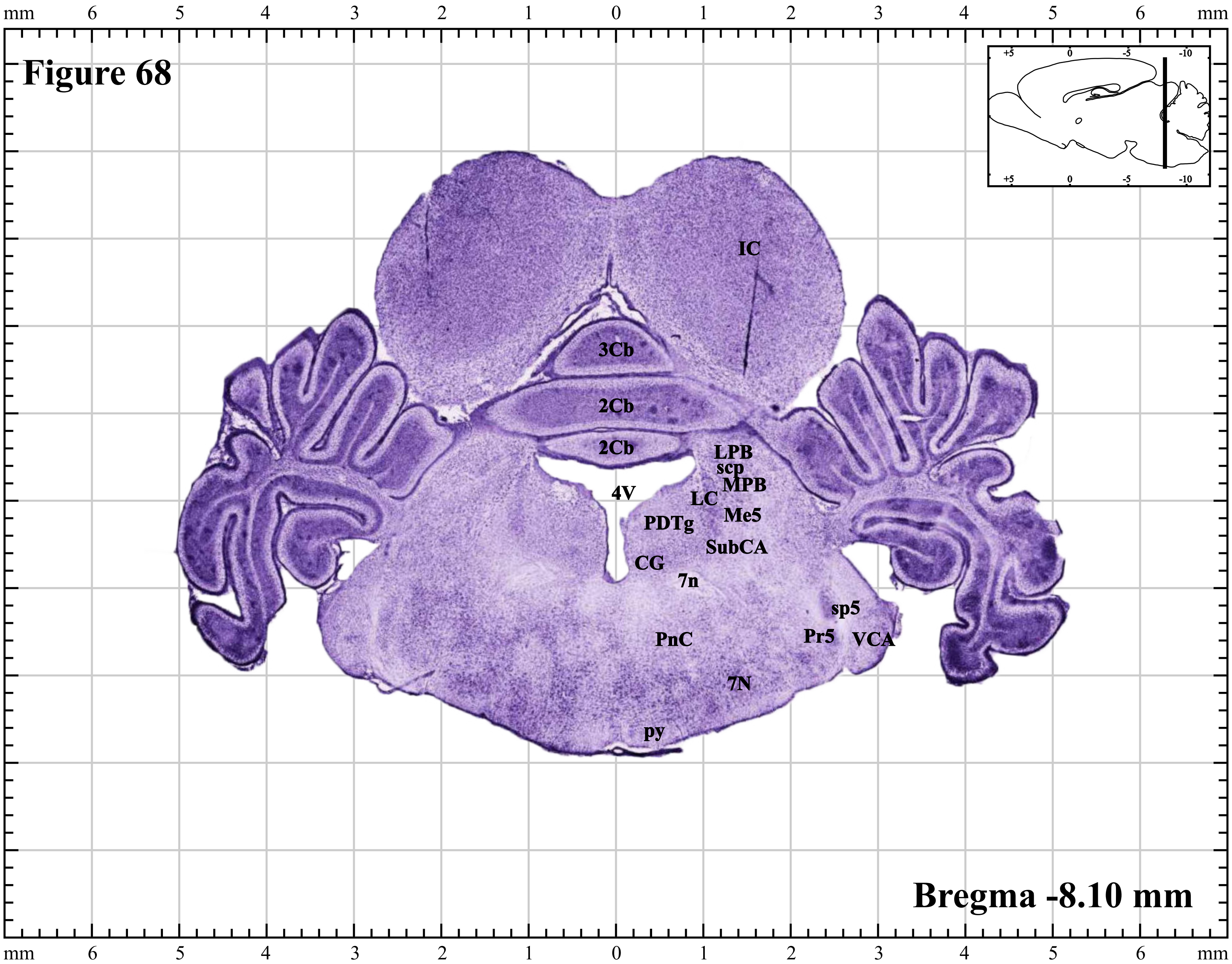

Bregma -8.10 mm

2Cb 2nd cerebellar lobule
3Cb 3rd cerebellar lobule
4V 4th ventricle
7n facial nerve
7N facial nuclues
CG central gray
IC inferior colliculus
LC locus coeruleus
LPB lateral parabrachial nucleus
Me5 mesencephalic trigeminal nucleus
MPB medial parabrachial nucleus
py pyramidal tract
Pr5 principal sensory trigeminal nucleus
PDTg posterodorsal tegmental nucleus
PnC pontine reticular nucleus, caudal part
sp5 spinal trigeminal tract
scp superior cerebellar peduncle
SubCA subcoeruleus nucleus, alpha part
SubCD subcoeruleus nucleus, dorsal part
VCA ventral cochlear nucleus, anterior part

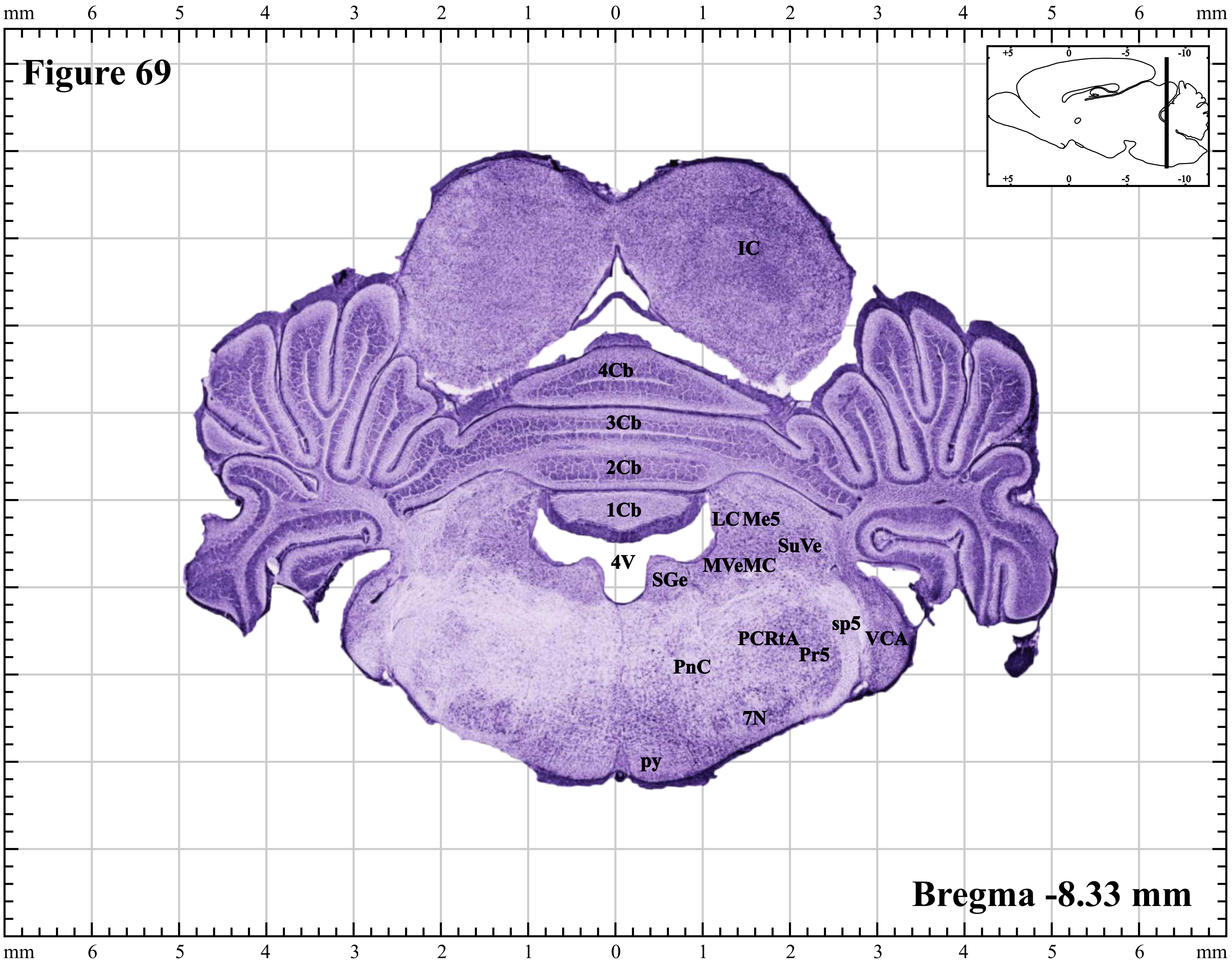

1Cb 1st cerebellar lobule (lingula)
2Cb 2nd cerebellar lobule
3Cb 3rd cerebellar lobule
4Cb 4th cerebellar lobule
4V 4th ventricle
7N facial nuclues
IC inferior colliculus
LC locus coeruleus
Me5 mesencephalic trigeminal nucleus
MVeMC medial vestibular nucleus, magnocellular part
py pyramidal tract
PCRtA parvicellular reticular nucleus, alpha part
PnC pontine reticular nucleus,caudal part
Pr5 principal sensory trigeminal nucleus
scp superior cerebellar peduncle
sp5 spinal trigeminal tract
SGe supragenual nucleus
SuVe superior vestibular nucleus
VCA ventral cochlear nucleus, anterior part

Figure 70

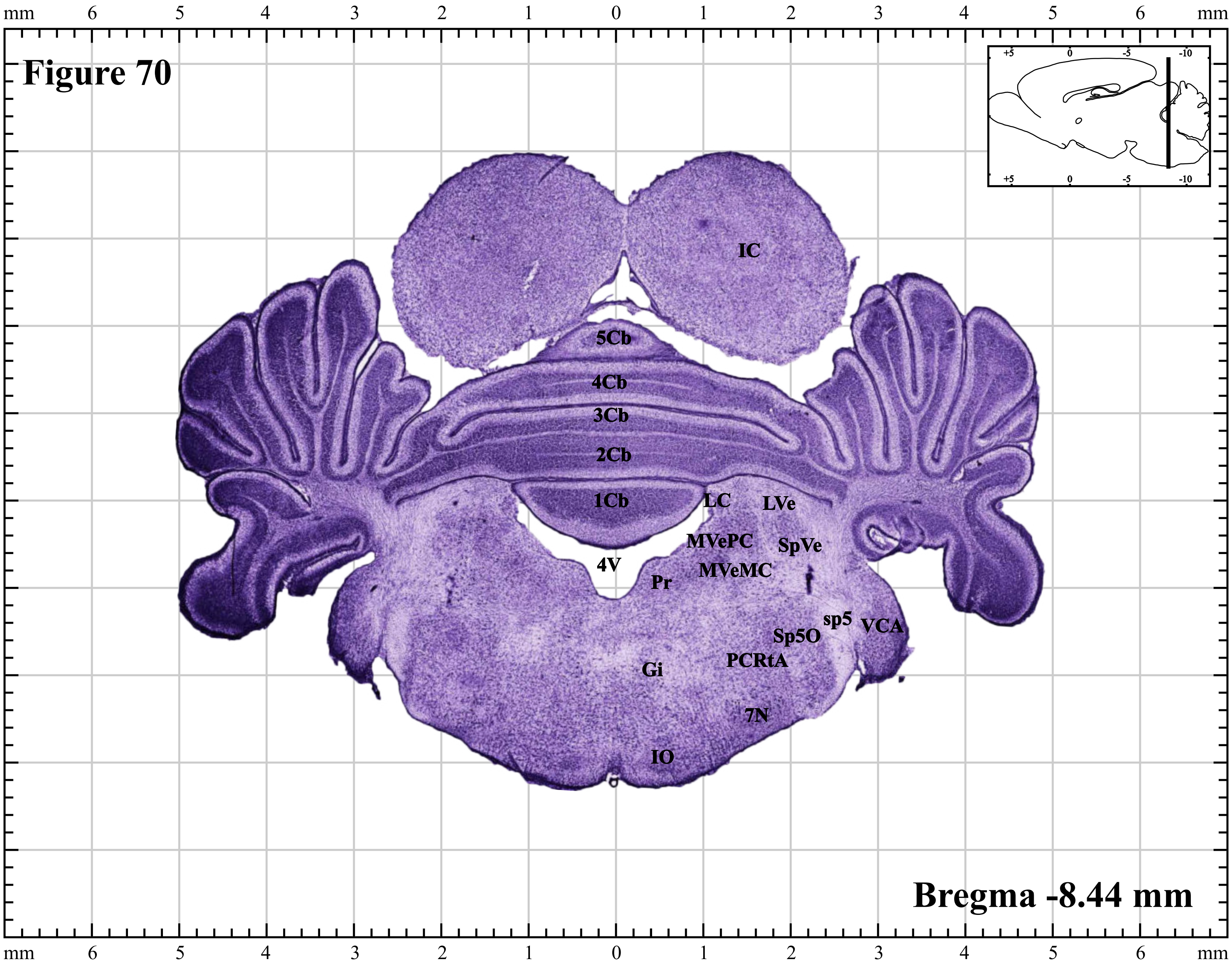

Bregma -8.44 mm

1Cb 1st cerebellar lobule (lingula)
2Cb 2nd cerebellar lobule
3Cb 3rd cerebellar lobule
4Cb 4th cerebellar lobule
4V 4th ventricle
5Cb 5th cerebellar lobule
7N facial nuclues
Gi granular insular cortex
IO inferior olive
IC inferior colliculus
LC locus coeruleus
LVe lateral vestibular nucleus
Me5 mesencephalic trigeminal nucleus
MVeMC medial vestibular nucleus, magnocellular part
MVePC medial vestibular nucleus, parvicellular part
PCRtA parvicellular reticular nucleus, alpha part
Pr prepositus nucleus
sp5 spinal trigeminal tract
SpVe spinal vestibular nucleus
Sp5O spinal trigeminal nucleus, oral part
VCA ventral cochlear nucleus, anterior part

Figure 71

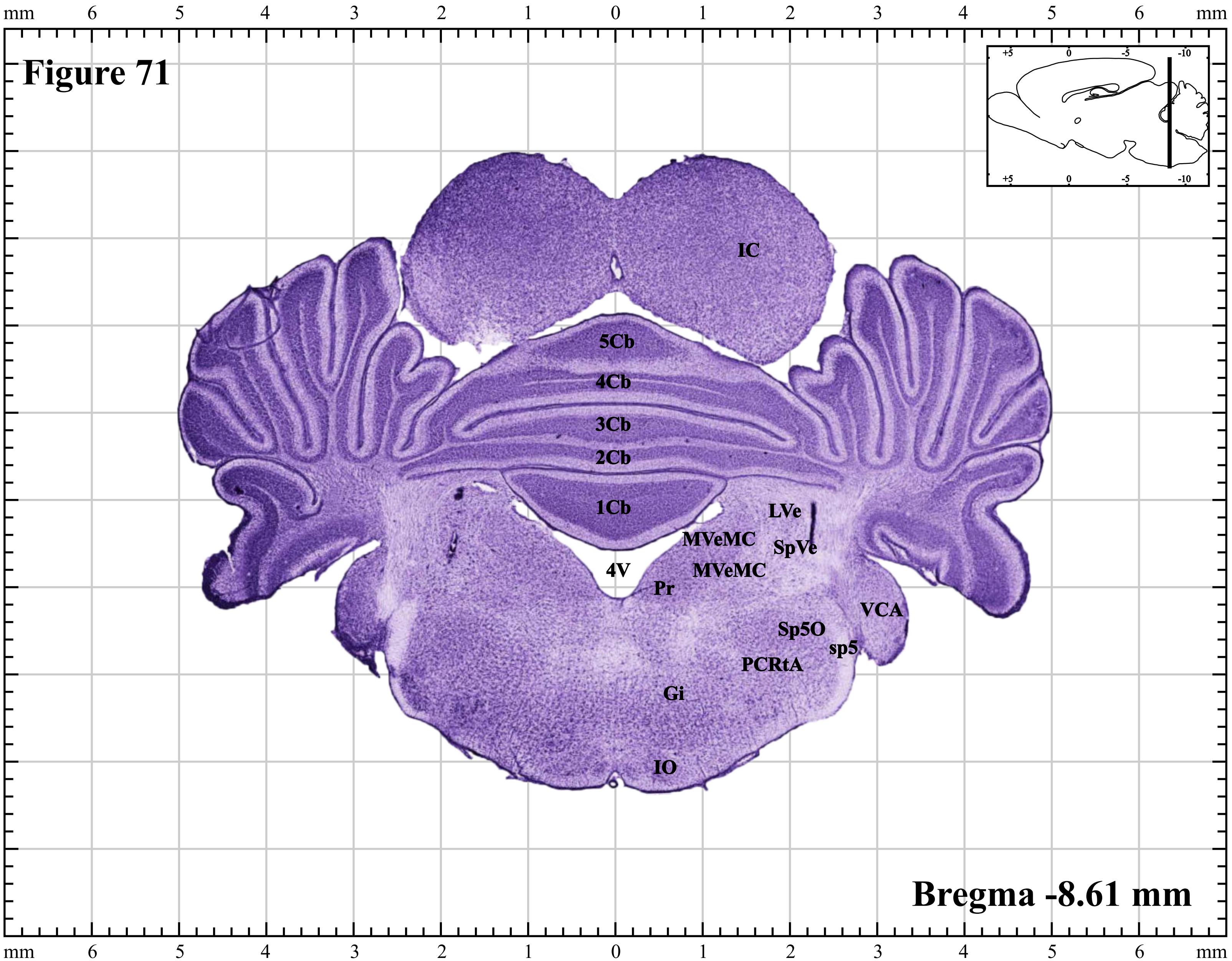

Bregma -8.61 mm

1Cb 1st cerebellar lobule (lingula)
2Cb 2nd cerebellar lobule
3Cb 3rd cerebellar lobule
4Cb 4th cerebellar lobule
4V 4th ventricle
5Cb 5th cerebellar lobule
Gi granular insular cortex
LC locus coeruleus
IO inferior olive
IC inferior colliculus
LVe lateral vestibular nucleus
Me5 mesencephalic trigeminal nucleus
MVeMC medial vestibular nucleus, magnocellular part
MVePC medial vestibular nucleus, parvicellular part
PCRtA parvicellular reticular nucleus, alpha part
Pr prepositus nucleus
sp5 spinal trigeminal tract
SpVe spinal vestibular nucleus
Sp5O spinal trigeminal nucleus, oral part
VCA ventral cochlear nucleus, anterior part

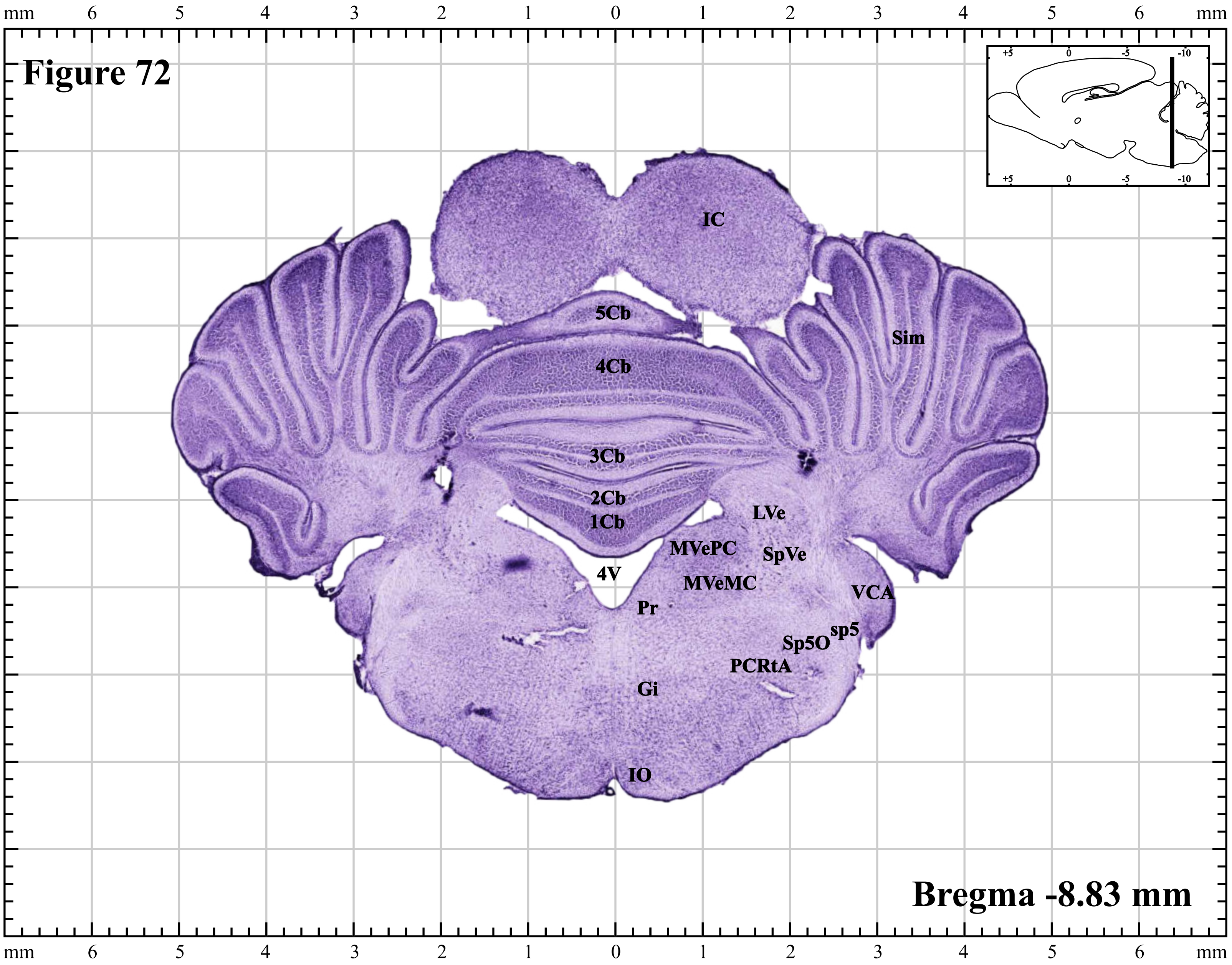

1Cb 1st cerebellar lobule (lingula)
2Cb 2nd cerebellar lobule
3Cb 3rd cerebellar lobule
4Cb 4th cerebellar lobule
4V 4th ventricle
5Cb 5th cerebellar lobule
Gi granular insular cortex
IO inferior olive
IC inferior colliculus
LVe lateral vestibular nucleus
Me5 mesencephalic trigeminal nucleus
MVeMC medial vestibular nucleus, magnocellular part
MVePC medial vestibular nucleus, parvicellular part
PCRtA parvicellular reticular nucleus, alpha part
Pr prepositus nucleus
sp5 spinal trigeminal tract
Sp5O spinal trigeminal nucleus, oral part
SpVe spinal vestibular nucleus
Sim simple lobule
VCA ventral cochlear nucleus, anterior part

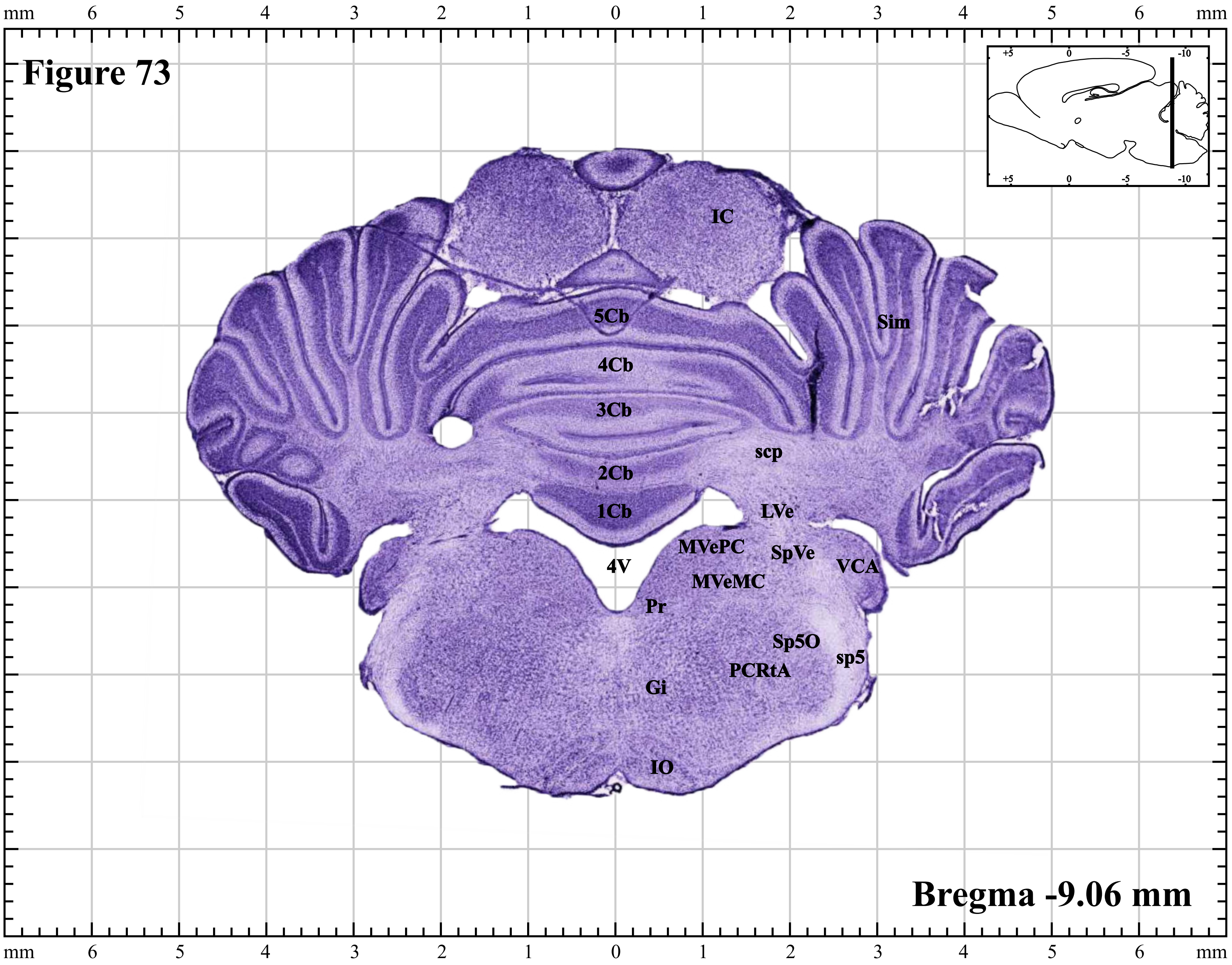

1Cb 1st cerebellar lobule (lingula)
2Cb 2nd cerebellar lobule
3Cb 3rd cerebellar lobule
4Cb 4th cerebellar lobule
4V 4th ventricle
5Cb 5th cerebellar lobule
Gi granular insular cortex
IO inferior olive
IC inferior colliculus
LVe lateral vestibular nucleus
Me5 mesencephalic trigeminal nucleus
MVeMC medial vestibular nucleus, magnocellular part
MVePC medial vestibular nucleus, parvicellular part
PCRtA parvicellular reticular nucleus, alpha part
Pr prepositus nucleus
scp superior cerebellar peduncle
sp5 spinal trigeminal tract
Sp5O spinal trigeminal nucleus, oral part
SpVe spinal vestibular nucleus
Sim simple lobule
VCA ventral cochlear nucleus, anterior part

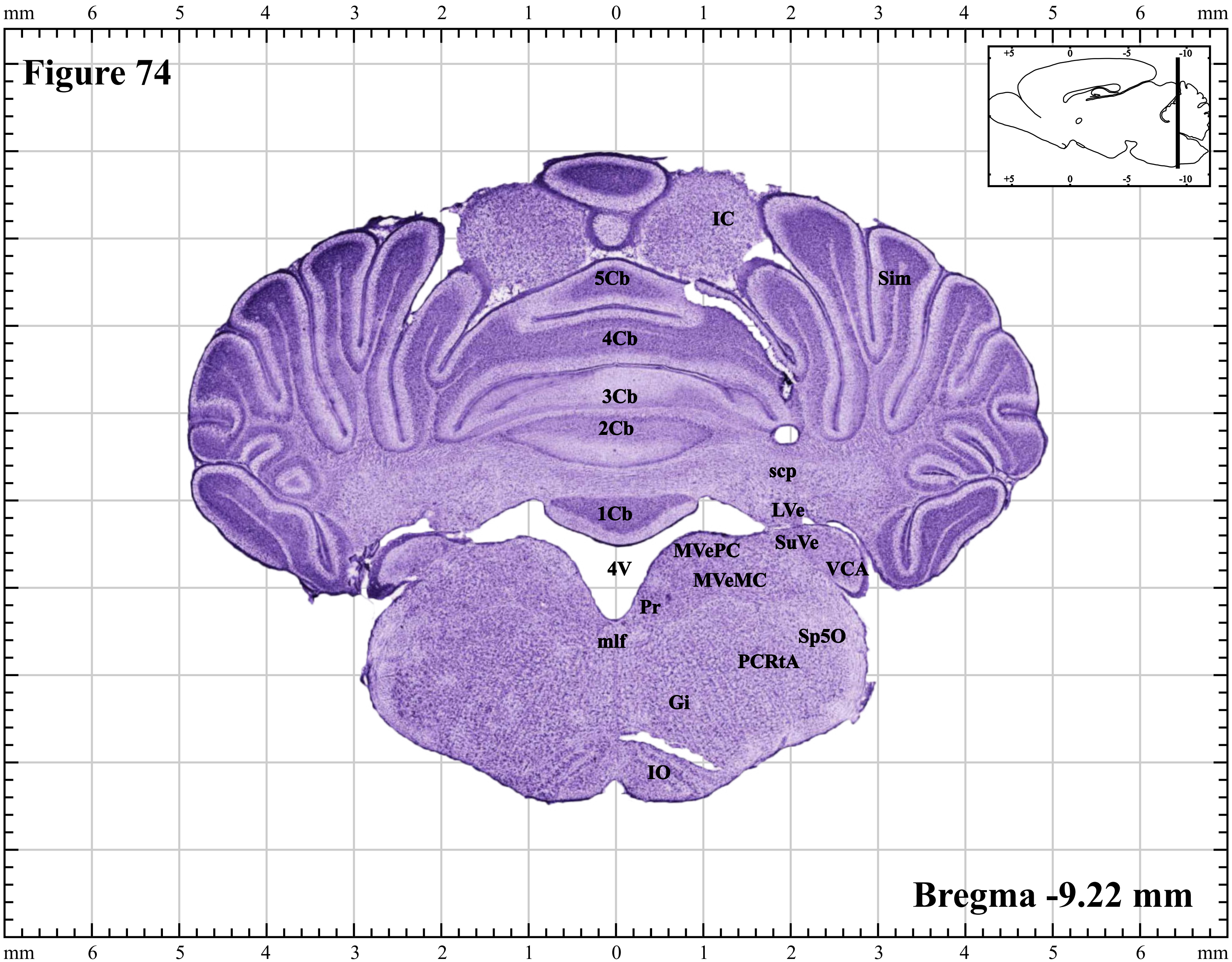

1Cb 1st cerebellar lobule (lingula)
2Cb 2nd cerebellar lobule
3Cb 3rd cerebellar lobules
4Cb 4th cerebellar lobule
4V 4th ventricle
5Cb 5th cerebellar lobule
Gi gigantocellular reticular nucleus
IC inferior colliculus
IO inferior olive
LVe lateral vestibular nucleus
mlf medial longitudinal fasciculus
MVeMC medial vestibular nucleus, magnocellular part
MVePC medial vestibular nucleus, parvicellular part
Pr prepositus nucleus
PCRtA parvicellular reticular nucleus, alpha part
scp superior cerebellar peduncle
Sp5O spinal trigeminal nucleus, oral part
SuVe superior vestibular nucleus
Sim simple lobule
VCA ventral cochlear nucleus, anterior part

Figure 75

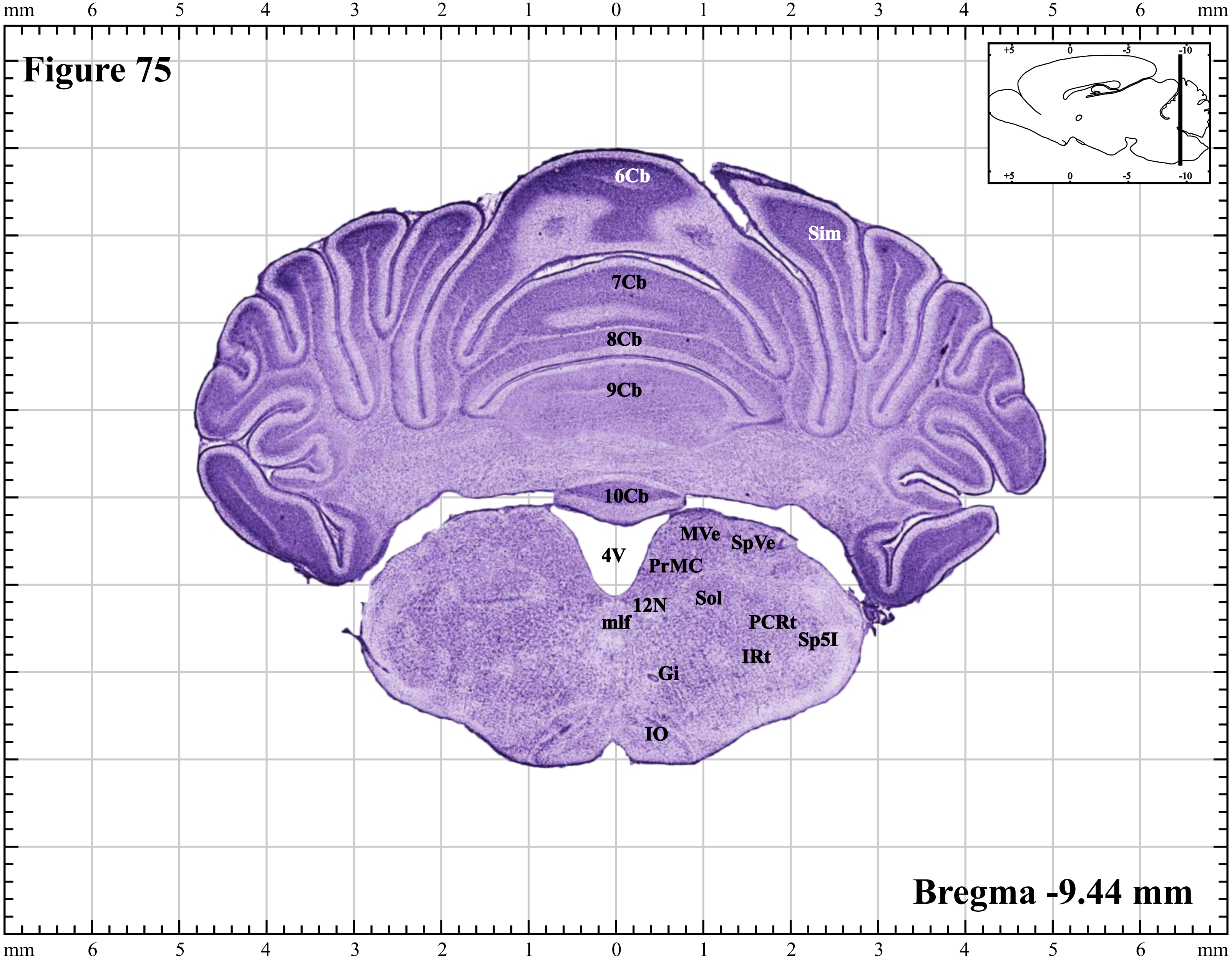

Bregma -9.44 mm

4V 4th ventricle
6Cb 6th cerebellar lobule (lingula)
7Cb 7th cerebellar lobule
8Cb 8th cerebellar lobule
9Cb 9th cerebellar lobules
10Cb 10th cerebellar lobule
12N hypoglossal nucleus
Gi granular insular cortex
IO inferior olive
IRt intermediate reticular nucleus
mlf medial longitudinal fasciculus
MVe medial vestibular nucleus
PCRt parvicellular reticular nucleus
PrMC prepositus nucleus,magnocellul
Sol nucleus of the solitary tract
Sim simple lobule
SpVe spinal vestibular nucleus
Sp5I spinal trigeminal nucleus, interpolar part

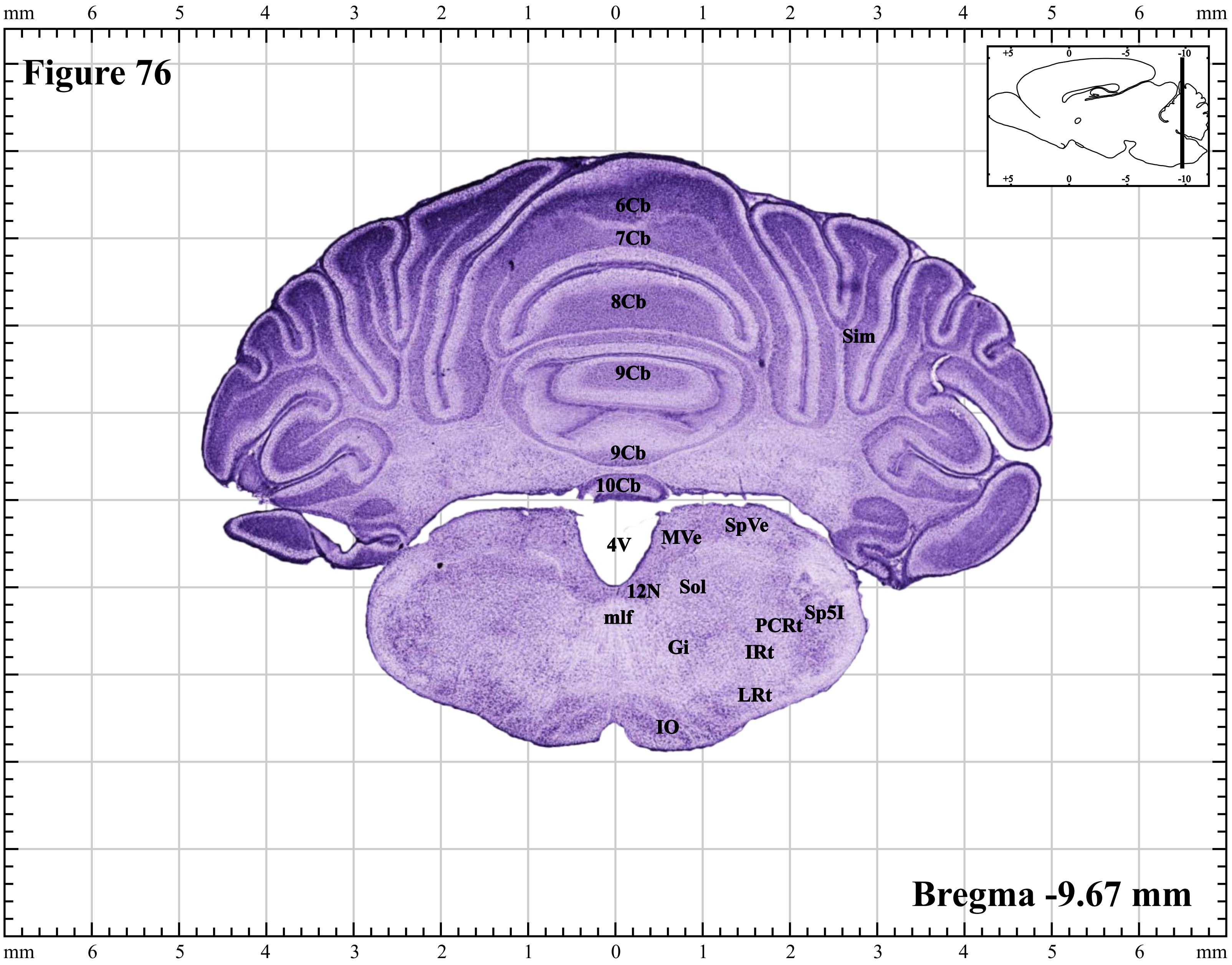

4V 4th ventricle
6Cb 6th cerebellar lobule (lingula)
7Cb 7th cerebellar lobule
8Cb 8th cerebellar lobule
9Cb 9th cerebellar lobules
10Cb 10th cerebellar lobule
12N hypoglossal nucleus
Gi granular insular cortex
IO inferior olive
IRt intermediate reticular nucleus
LRt lateral reticular nucleus
mlf medial longitudinal fasciculus
MVe medial vestibular nucleus
PCRt parvicellular reticular nucleus
Sol nucleus of the solitary tract
Sim simple lobule
SpVe spinal vestibular nucleus
Sp5I spinal trigeminal nucleus, interpolar part

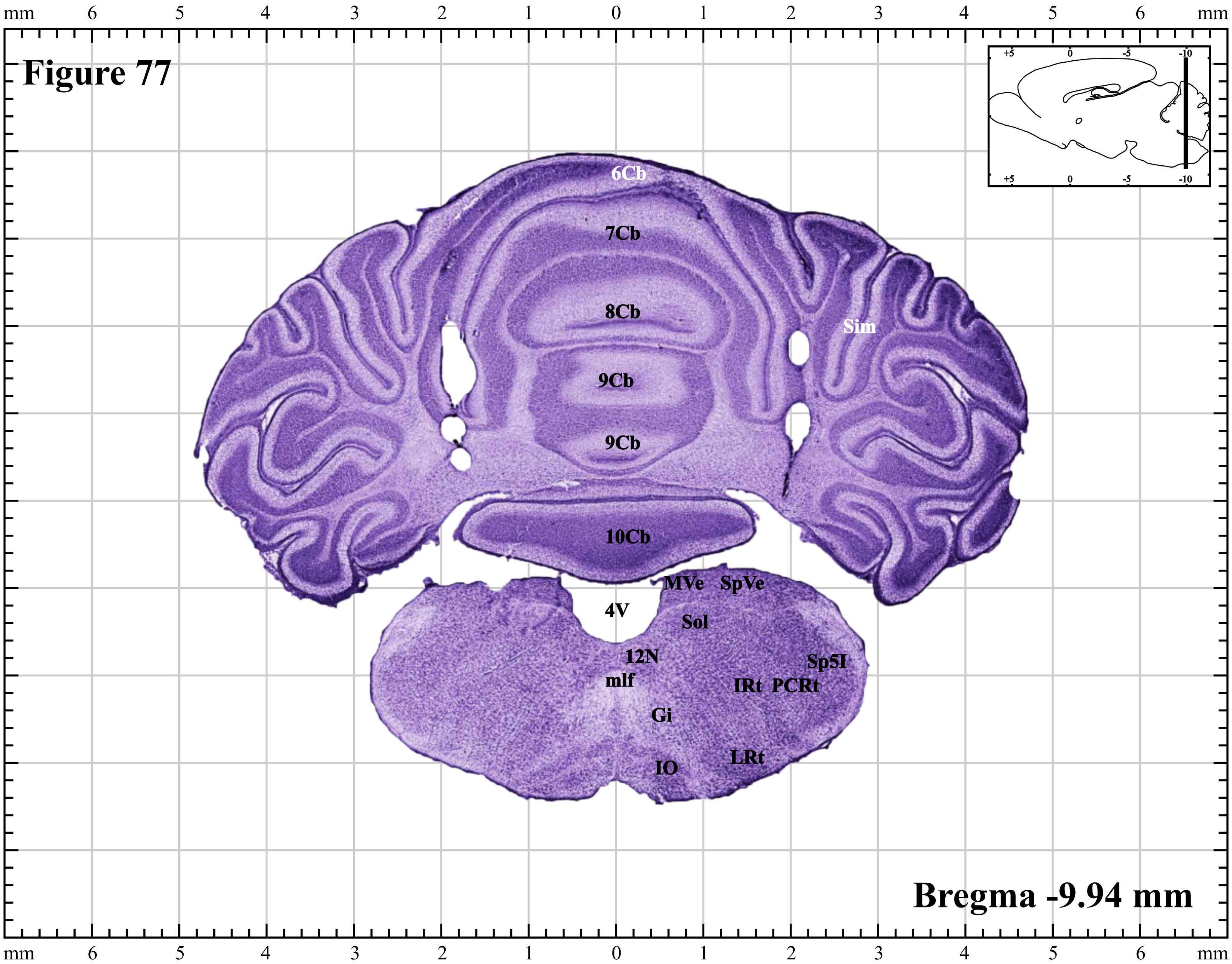

- **4V** 4th ventricle
- **6Cb** 6th cerebellar lobule (lingula)
- **7Cb** 7th cerebellar lobule
- **8Cb** 8th cerebellar lobule
- **9Cb** 9th cerebellar lobules
- **10Cb** 10th cerebellar lobule
- **12N** hypoglossal nucleus
- **Gi** granular insular cortex
- **IO** inferior olive
- **IRt** intermediate reticular nucleus
- **LRt** lateral reticular nucleus
- **mlf** medial longitudinal fasciculus
- **MVe** medial vestibular nucleus
- **PCRt** parvicellular reticular nucleus
- **Sol** nucleus of the solitary tract
- **Sim** simple lobule
- **SpVe** spinal vestibular nucleus
- **Sp5I** spinal trigeminal nucleus, interpolar part

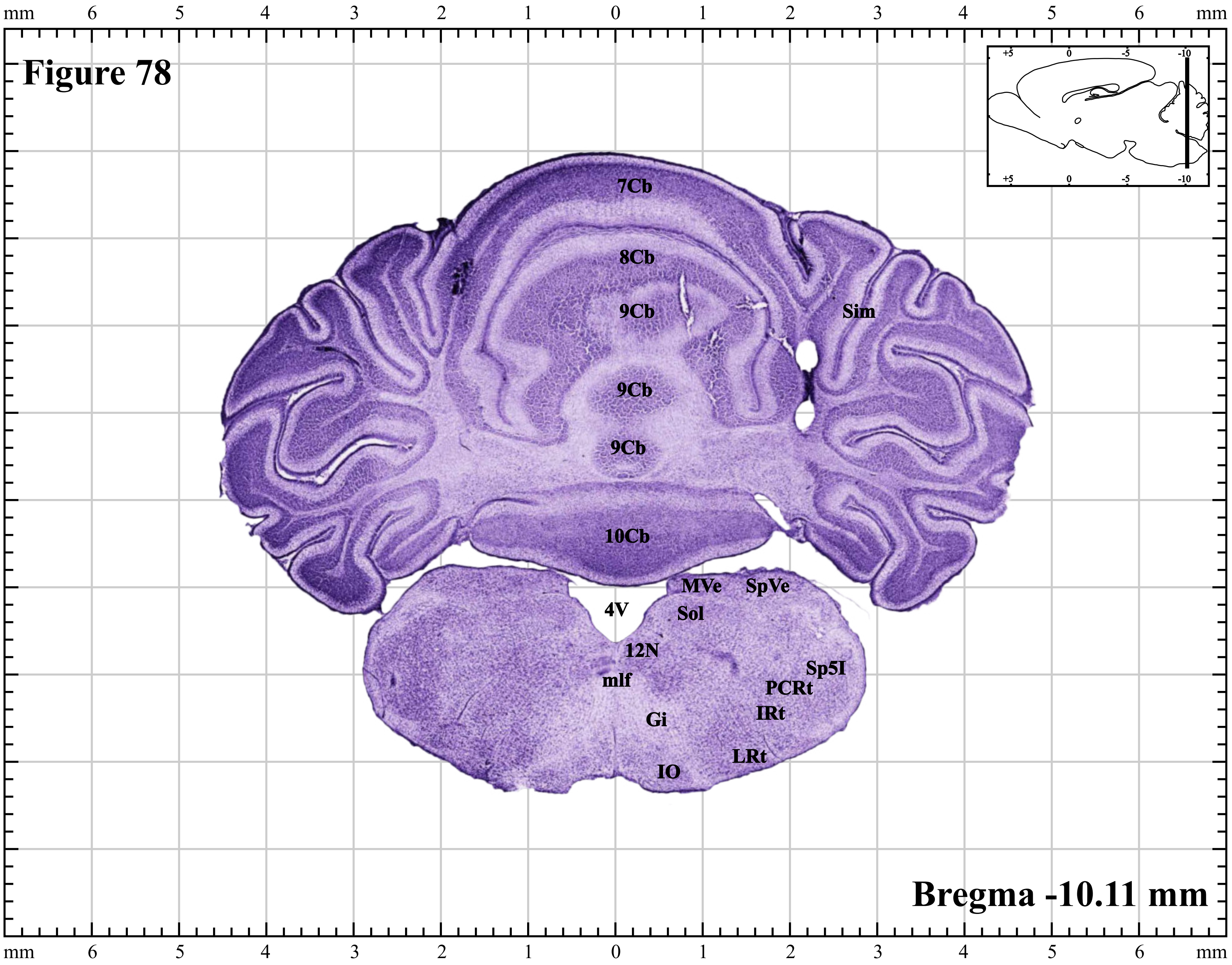

4V 4th ventricle
7Cb 7th cerebellar lobule
8Cb 8th cerebellar lobule
9Cb 9th cerebellar lobules
10Cb 10th cerebellar lobule
12N hypoglossal nucleus
Gi granular insular cortex
IO inferior olive
IRt intermediate reticular nucleus
LRt lateral reticular nucleus
mlf medial longitudinal fasciculus
MVe medial vestibular nucleus
PCRt parvicellular reticular nucleus
Sol nucleus of the solitary tract
Sim simple lobule
SpVe spinal vestibular nucleus
Sp5I spinal trigeminal nucleus, interpolar part

Figure 79

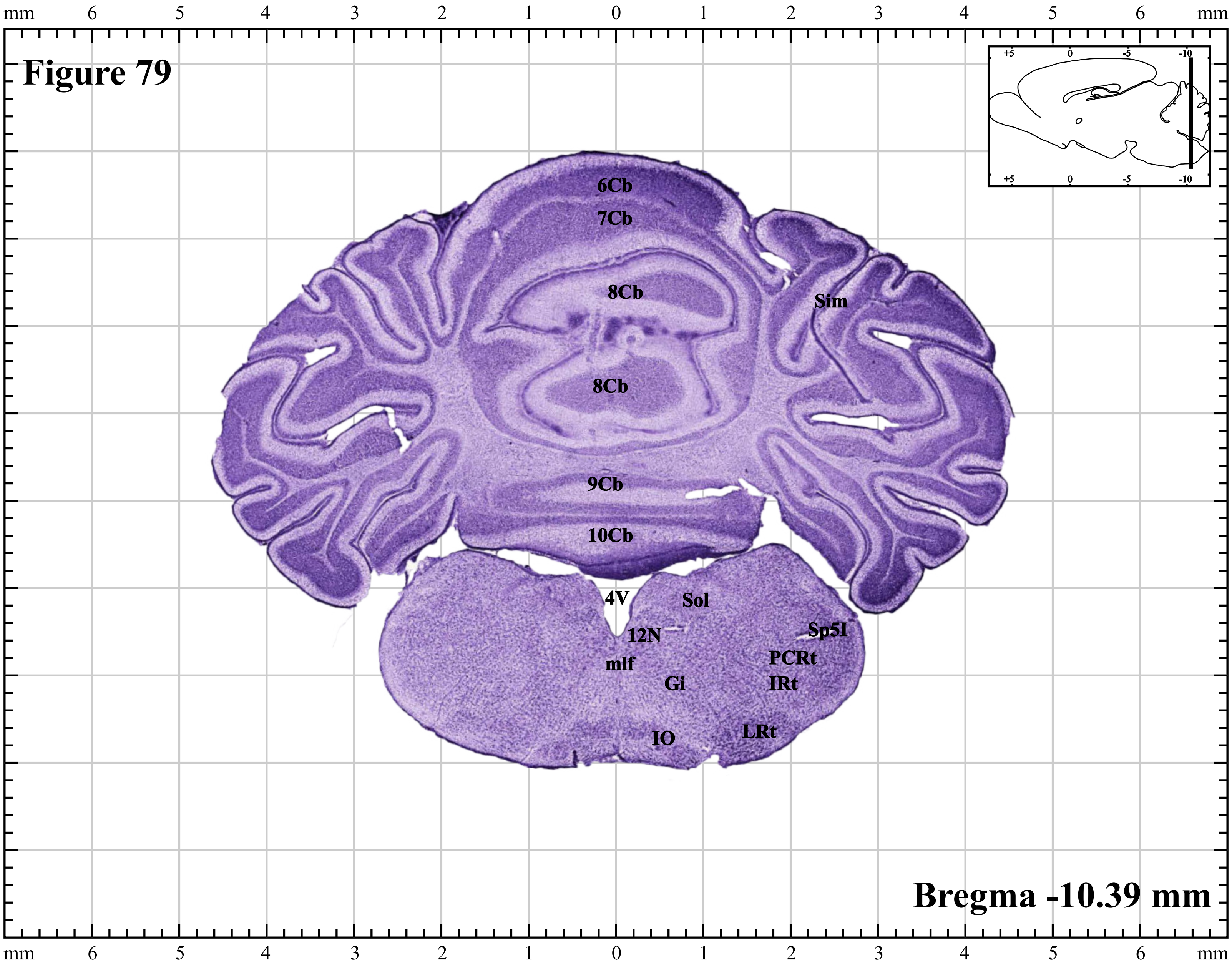

Bregma -10.39 mm

4V 4th ventricle
6Cb 6th cerebellar lobule
7Cb 7th cerebellar lobule
8Cb 8th cerebellar lobule
9Cb 9th cerebellar lobules
10Cb 10th cerebellar lobule
12N hypoglossal nucleus
Gi granular insular cortex
IO inferior olive
IRt intermediate reticular nucleus
LRt lateral reticular nucleus
mlf medial longitudinal fasciculus
PCRt parvicellular reticular nucleus
Sol nucleus of the solitary tract
Sim simple lobule
Sp5I spinal trigeminal nucleus, interpolar part

Figure 80

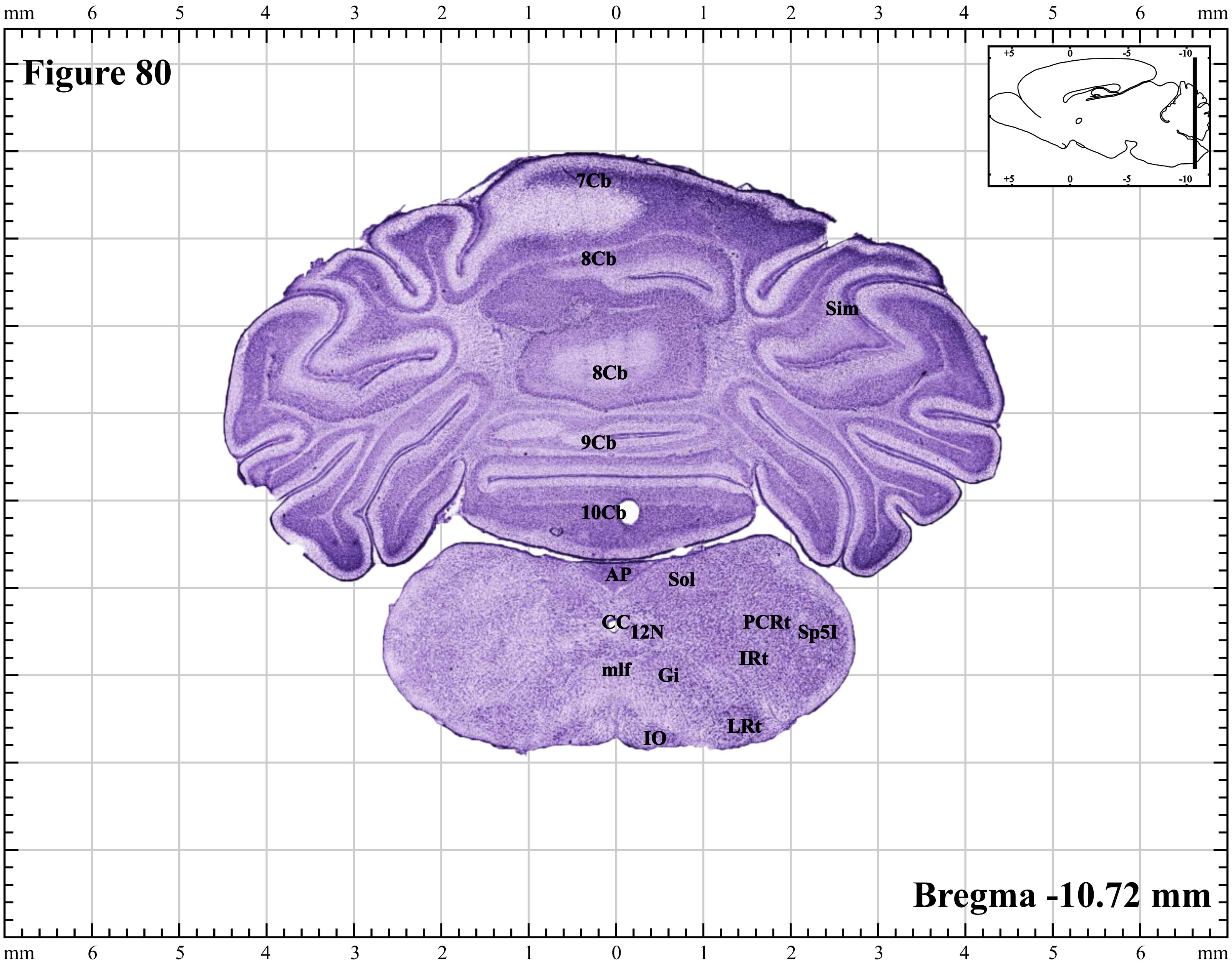

Bregma -10.72 mm

7Cb 7th cerebellar lobule
8Cb 8th cerebellar lobule
9Cb 9th cerebellar lobules
10Cb 10th cerebellar lobule
12N hypoglossal nucleus
AP area postrema
CC central canal
Gi granular insular cortex
IO inferior olive
IRt intermediate reticular nucleus
LRt lateral reticular nucleus
mlf medial longitudinal fasciculus
PCRt parvicellular reticular nucleus
Sol nucleus of the solitary tract
Sim simple lobule
Sp5I spinal trigeminal nucleus, interpolar part

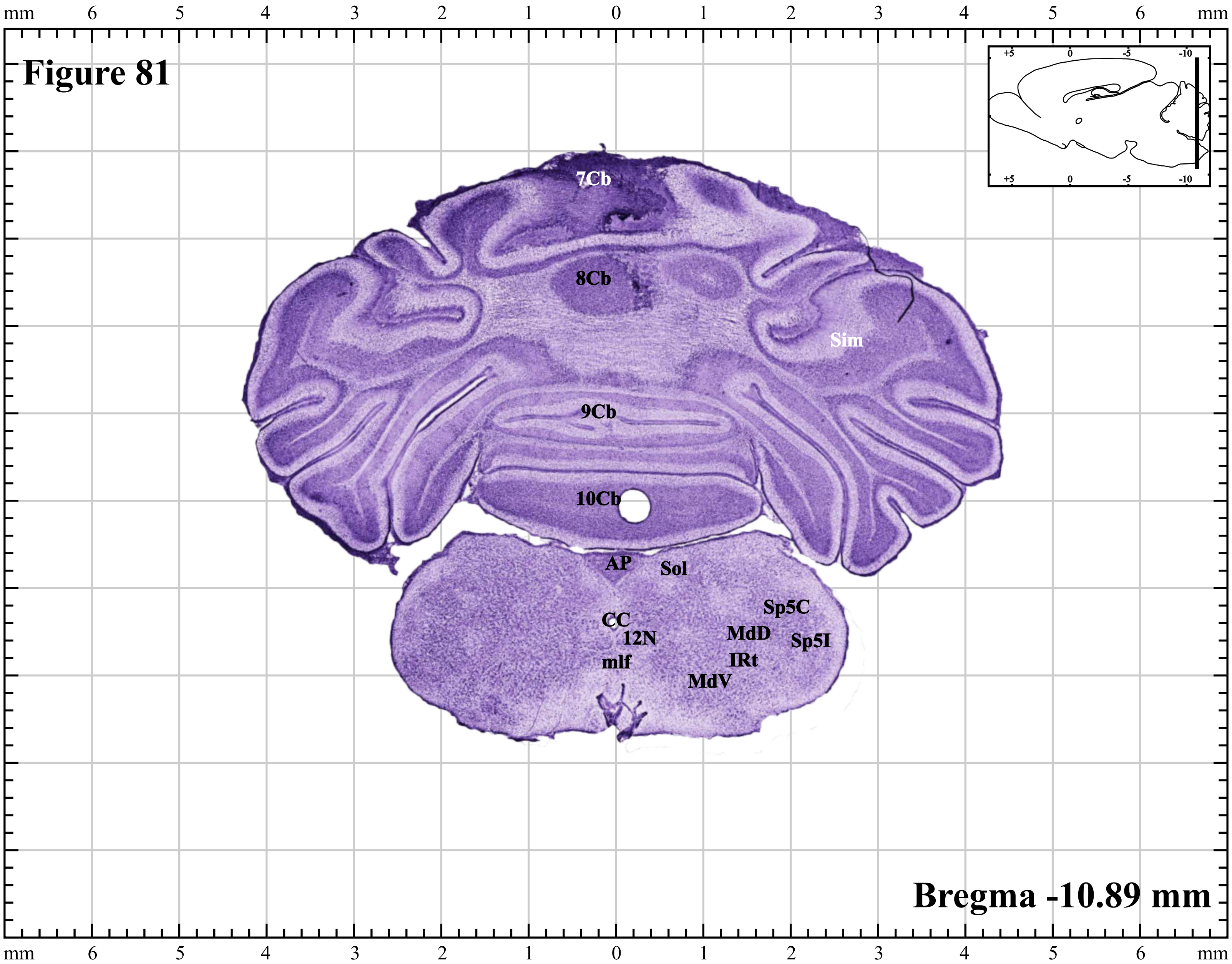

7Cb 7th cerebellar lobule
8Cb 8th cerebellar lobule
9Cb 9th cerebellar lobules
10Cb 10th cerebellar lobule
12N hypoglossal nucleus
AP area postrema
CC central canal
Gi granular insular cortex
IRt intermediate reticular nucleus
mlf medial longitudinal fasciculus
MdD medullary reticular nucleus, dorsal part
MdV medullary reticular nucleus, ventral part
Sol nucleus of the solitary tract
Sim simple lobule
Sp5I spinal trigeminal nucleus, interpolar part
Sp5C spinal trigeminal nucleus, caudal part

Figure 82

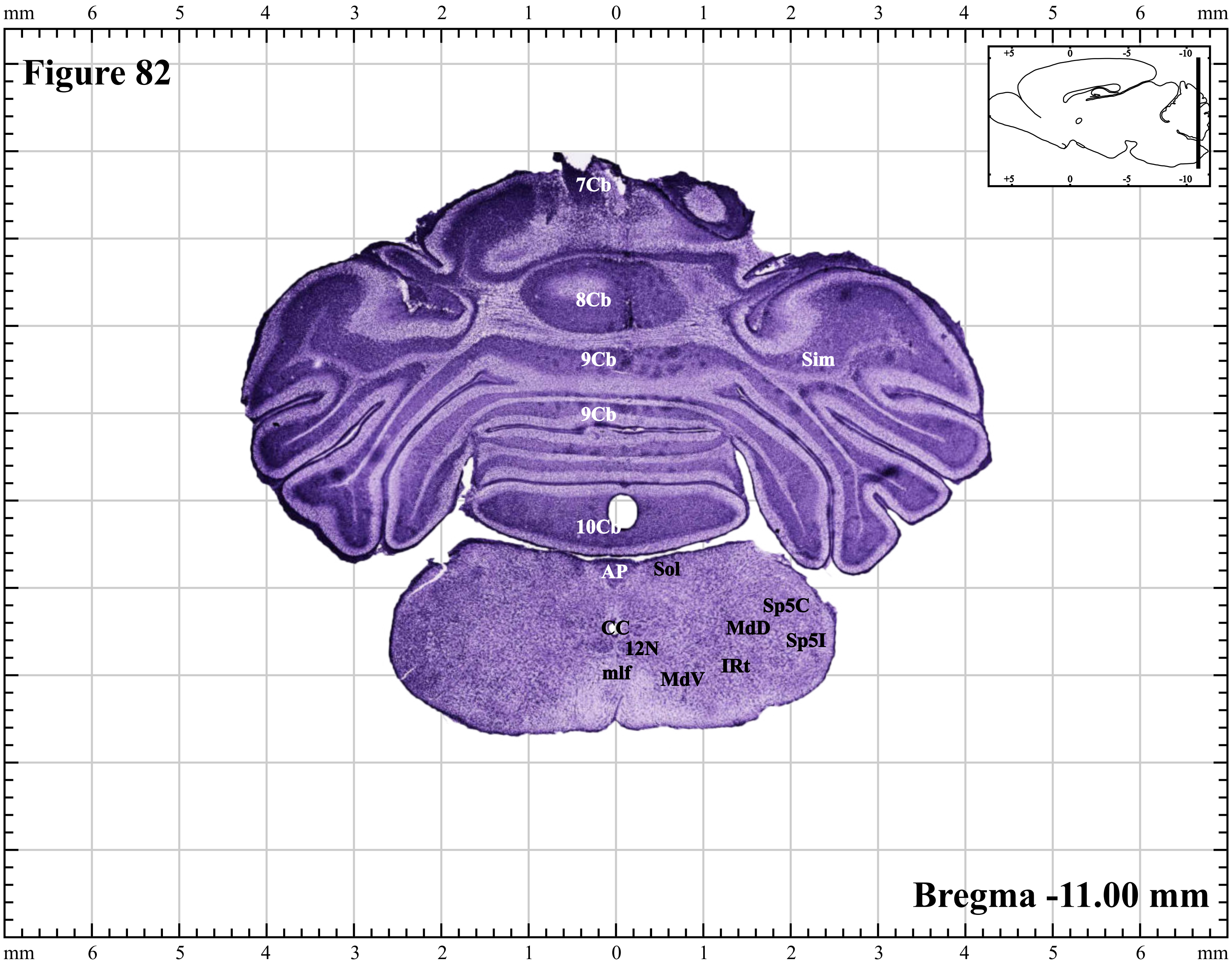

Bregma -11.00 mm

7Cb 7th cerebellar lobule
8Cb 8th cerebellar lobule
9Cb 9th cerebellar lobules
10Cb 10th cerebellar lobule
12N hypoglossal nucleus
AP area postrema
CC central canal
Gi granular insular cortex
IRt intermediate reticular nucleus
mlf medial longitudinal fasciculus
MdD medullary reticular nucleus, dorsal part
MdV medullary reticular nucleus, ventral part
Sol nucleus of the solitary tract
Sim simple lobule
Sp5I spinal trigeminal nucleus, interpolar part
Sp5C spinal trigeminal nucleus, caudal part
